# Effect of alirocumab on cataracts in patients with acute coronary syndromes

**DOI:** 10.1186/s12886-023-03012-1

**Published:** 2023-06-16

**Authors:** Gaspard Suc, Gregory G. Schwartz, Shaun G. Goodman, J. Wouter Jukema, Garen Manvelian, Yann Poulouin, Robert Pordy, Michel Scemama, Michael Szarek, Ph. Gabriel Steg, ODYSSEY OUTCOMES Investigators

**Affiliations:** 1 Université Paris-Cité, INSERM_U1148/LVTS, Paris, France; 2 Assistance Publique-Hôpitaux de Paris, Hôpital Bichat, Paris, France; 3 Division of Cardiology, University of Colorado School of Medicine, Aurora, CO USA; 4 Canadian VIGOUR Centre, University of Alberta, Edmonton, AB Canada; 5 St. Michael’s Hospital, University of Toronto, Toronto, ON Canada; 6 Department of Cardiology, Leiden University Medical Center, Leiden, the Netherlands; 7 Netherlands Heart Institute, Utrecht, the Netherlands; 8 Regeneron Pharmaceuticals, Tarrytown, NY USA; 9 IT&M Stats, Neuilly-sur-Seine, France; 10 Sanofi, Chilly-Mazarin, France; 11 Downstate School of Public Health, State University of New York, Brooklyn, NY USA; 12 CPC Clinical Research and Division of Cardiology, University of Colorado School of Medicine, Aurora, CO USA; 13 Institut Universitaire de France, Paris, France; 14 FACT (French Alliance for Cardiovascular Trials), INSERM U-1148, Paris, France; 15 Département de Cardiologie, AP-HP Hôpital Bichat, 46 Rue Henri Huchard, Paris, 75018 France

**Keywords:** Acute coronary syndrome, Cataracts, Alirocumab, PCSK9 inhibitor

## Abstract

**Background:**

Some data suggest that low levels of low-density lipoprotein cholesterol (LDL-C) are associated with risk of cataracts. Proprotein convertase subtilisin–kexin type 9 (PCSK9) inhibitors reduce LDL-C below levels achieved with statins alone. We determined whether the incidence of cataracts was influenced by treatment with the PCSK9 inhibitor alirocumab versus placebo, and whether that incidence was affected by achieved LDL-C levels.

**Methods:**

The ODYSSEY OUTCOMES trial (NCT01663402) compared alirocumab with placebo in 18,924 patients with recent acute coronary syndrome receiving high-intensity or maximum-tolerated statin. Incident cataracts were pre-specified events of interest. In multivariable analysis using propensity score-matching on characteristics including cataract risk factors, incident cataracts were compared in the alirocumab and placebo groups according to LDL-C levels achieved with alirocumab.

**Results:**

Over median follow-up of 2.8 years (interquartile range 2.3 − 3.4), the incidence of cataracts was similar with alirocumab (127/9462 [1.3%]) versus placebo (134/9462 [1.4%]); hazard ratio [HR] 0.94, 95% confidence interval [CI] 0.74 − 1.20). In patients treated with alirocumab with ≥ 2 LDL-C values < 25 mg/dL (0.65 mmol/L), the incidence of cataracts was 71/4305 (1.6%), versus 60/4305 (1.4%) in propensity score-matched patients from the placebo group (HR 1.10, CI 95% 0.78 − 1.55). In patients treated with alirocumab with ≥ 2 LDL-C values < 15 mg/dL (0.39 mmol/L), the incidence of cataracts was 13/782 (1.7%), versus 36/2346 (1.5%) in matched patients from the placebo group (HR 1.03, CI 95% 0.54 − 1.94).

**Conclusion:**

Treatment with alirocumab versus placebo, added to statin, did not influence the incidence of cataracts, even when achieved LDL-C levels on alirocumab were very low. Longer follow-up studies might be necessary to exclude the long-term effects on the incidence or progression of cataracts.

**Trial registration:**

ClinicalTrials.gov Identifier: NCT01663402.

**Supplementary Information:**

The online version contains supplementary material available at 10.1186/s12886-023-03012-1.

## Introduction

Age-related cataract is the most common cause of visual impairment and blindness worldwide [1]. The lens fibers that represent the majority of the volume of the lens have a high cholesterol concentration [2]. The avascular lens grows through life, by accumulating fiber cells surrounded by a very rich cholesterol plasma membrane [3, 4]. Hence, concerns have been raised that low levels of low-density lipoprotein cholesterol (LDL-C) may increase the risk of cataracts [5], but clinical trial data have not confirmed this association with statins [6, 7]. Proprotein convertase subtilisin–kexin type 9 (PCSK9) inhibitors are potent cholesterol-lowering drugs with the potential to reduce LDL-C to levels well below those achievable with statins [8]. Their safety profile, and especially the risk of cataracts in patients achieving very low LDL C remains debated [9–15].

The aim of this analysis is to describe the incidence of cataracts in alirocumab- and placebo-treated patients, and to assess whether patients achieving very low LDL-C levels (< 25 or 15 mg/dL [0.65 mmol/L or 0.39 mmol/L]) on treatment experienced a higher incidence of cataracts than those who did not.

## Methods

The ODYSSEY OUTCOMES trial [16, 17] (NCT01663402; 08/08/2012; Additional file 1: Appendix) was a randomized, double-blind trial that evaluated the efficacy and safety of alirocumab versus placebo in patients with acute coronary syndrome (ACS) and elevated atherogenic lipoproteins despite high-intensity or maximum-tolerated statin treatment. Patient eligibility criteria are detailed in the Additional file 2: Appendix.

Patients with LDL-C ≥ 70 mg/dL [1.81 mmol/L], non − high-density lipoprotein cholesterol ≥ 100 mg/dL [2.59 mmol/L], or apolipoprotein B ≥ 80 mg/dL) were randomized (1:1) to receive alirocumab 75 mg subcutaneously every 2 weeks or matching placebo starting 1‒12 months after ACS. Randomization, with stratification for country, was performed centrally using an interactive voice-response or web-response system [16, 17]. In the alirocumab group, a blinded, protocol-specified, dose-adjustment algorithm was used to maximize the number of participants achieving an LDL-C level of 25‒50 mg/dL (0.65 − 1.29 mmol/L). Alirocumab 75 mg could be blindly up-titrated to 150 mg if LDL-C was ≥ 50 mg/dL (1.29 mmol/L). Conversely, in patients on the 150 mg dose, the 75 mg was down-titrated if LDL-C was < 15 mg/dL (0.39 mmol/L) on two consecutive measurements. If the LDL-C level was < 15 mg/dL (0.39 mmol/L) on two consecutive measurements on the 75 mg alirocumab dose, placebo was blindly substituted for alirocumab for the rest of the trial. Both patients and investigators were masked to treatment assignment and lipid concentrations.

### Statistical analysis

Cataract-related events (incident cataracts or worsening of pre-existing cataracts) were pre-specified events of special interest. They were assessed prospectively as investigator-reported treatment-emergent adverse effects and grouped under the high-level term cataract conditions from MedDRA coding (see Statistical Analysis Plan [16, 17]).

Categorical variables were compared with the chi-square test. If relevant, 2-sided 95% confidence interval (CI) of proportion is displayed (Wilson method). Hazard ratios (HR) and 95% CIs were estimated using a Cox proportional-hazards model. Multivariable analysis was performed using 2 propensity-score − matched cohorts to compare incidence of cataracts according to LDL-C levels in alirocumab-treated patients. Each alirocumab patient with ≥ 2 consecutive LDL-C values < 15 mg/dL (0.39 mmol/L) was matched with 3 placebo patients paired according to these variables. Each alirocumab patient with ≥ 2 consecutive LDL-C values < 25 mg/dL (0.65 mmol/L) was matched with one placebo-treated patient. Baseline characteristics considered for matching were age, sex, smoking, region, diabetes, glycated hemoglobin A1c, lipoprotein(a), body mass index, percent change in LDL-C from qualifying to baseline, and LDL-C value. The trial was powered for the primary efficacy outcome of major adverse cardiac events but not for safety events. Therefore, the safety analysis presented must be viewed as descriptive only. The follow-up period extended from the first injection of study medication (alirocumab or placebo) until 70 days after the final injection of study medication. All analyses were performed using SAS® version 9.4, (SAS Institute, Inc., Cary, North Carolina).

## Results

**Fig. 1:**
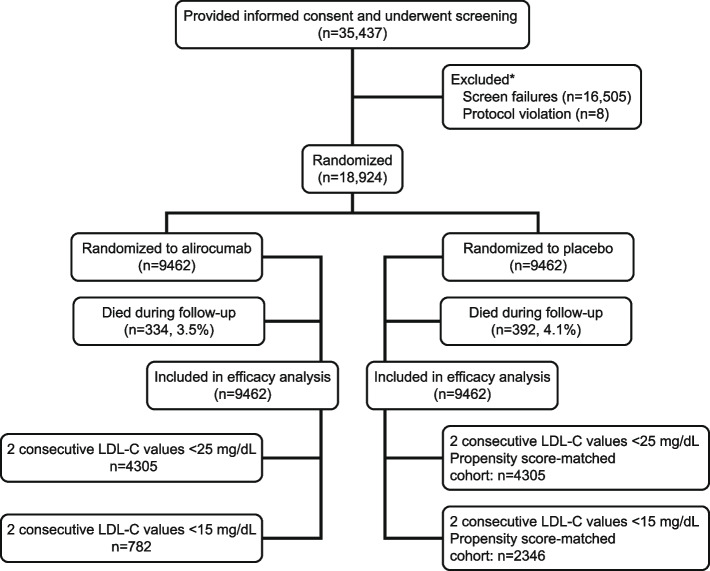
Consort diagram. *The most common reasons for screen failure during the run-in period were related to lipid criteria (34.1% of patients) or withdrawal of consent (6.1% of patients)

A total of 18,924 patients were randomized at 1315 sites in 57 countries; 9462 were assigned to alirocumab and 9462 to placebo (Fig. 1).

Outside of China, patients were randomized between November 2012 and November 2015. In China, 613 patients were randomized between May 2016 and February 2017. At baseline, mean ± SD LDL-C was 92 ± 31 mg/dL (2.41 ± 0.80). At 12 months after randomization by intention-to-treat analysis, mean LDL-C was 48 mg/dL (1.24 mmol/L) in the alirocumab group versus 96 mg/dL (2.49 mmol/L) in the placebo group. At baseline, 182 (1.9%) patients in the alirocumab group and 194 (2.1%) in the placebo group had a history of cataracts.

The median (Q1, Q3) duration of exposure to treatment was 30.9 (0.5, 61.0) months in the alirocumab group and 31.8 (0.5, 60.5)months in the placebo group. After a median (Q1, Q3) follow-up of 2.8 (2.3, 3.4) years, the incidence of cataract in the (unmatched) safety population was similar in alirocumab and placebo groups (127 [1.3%] versus 134 [1.4%] patients, respectively; HR 0.94, 95% CI 0.74 − 1.20) (Table 1). Cataracts were reported as serious adverse events in 0.1% of patients in both treatment groups, leading to study-drug discontinuation in 2 alirocumab patients and 1 placebo patient. Among patients with cataract, most reported mild (40.0% with alirocumab; 56.0% with placebo) or moderate symptoms (54.2% and 38.1%, respectively). A small proportion of those with cataract reported severe symptoms (5.8% and 6.0%, respectively).

**Table 1:** Cataract-related treatment-emergent adverse events in the safety population

	**Placebo** **(*N* = 9443)**	**Alirocumab** **(*N* = 9451)**
Any cataract-related treatment-emergent adverse event		
N, % (95% CI)	134, 1.4% (1.2, 1.7)	127, 1.3% (1.1, 1.5)
Event rate per 100 patient-years (95% CI)^a^	0.5 (0.5, 0.6)	0.5 (0.4, 0.6)
Hazard ratio versus placebo (95% CI)^b^		0.93 (0.73, 1.20)
Subcategories of cataract-related treatment-emergent adverse events, N (%)		
Cataract	127 (1.3%)	116 (1.2%)
Atopic cataract	0	1 (< 0.1%)
Cataract cortical	2 (< 0.1%)	1 (< 0.1%)
Cataract nuclear	3 (< 0.1%)	1 (< 0.1%)
Cataract subcapsular	0	1 (< 0.1%)
Lenticular opacities	1 (< 0.1%)	1 (< 0.1%)
Cataract diabetic	1 (< 0.1%)	0

*CI* Confidence interval

^a^ Calculated as number of patients with an event divided by total patient years. For patients with event, number of patient years is calculated up to date of the first event, for patients without event, it corresponds to the length of the treatment-emergent adverse event period

^b^ Calculated using a Cox model

A total of 4305 patients in the alirocumab group had ≥ 2 consecutive LDL-C values < 25 mg/dL (0.65 mmol/L) and were matched to 4305 patients from the placebo group with similar baseline characteristics (Tables 2 and 3). Baseline characteristics of these patients included mean age 59 years, male sex (81%), diabetes (33%); and mean body mass index 28.3 kg/m^2^, LDL-C 2.1 mmol/L, lipoprotein(a) 28.6 mg/dL, and apolipoprotein A1 131.6 mg/dL. A total of 782 patients in the alirocumab group had ≥ 2 consecutive LDL-C values < 15 mg/dL (0.39 mmol/L) and were matched to 2346 patients from the placebo group with similar baseline characteristics (Tables 4 and 5). Baseline characteristics of these patients included mean age 59 years, male sex (81%), diabetes (33%); and mean body mass index 27.3 kg/m^2^, LDL-C 2.0 mmol/L, lipoprotein(a) 18.8 mg/dL, and apolipoprotein A1 129.9 mg/dL.

**Table 2:** Baseline demographics and characteristics of patients in the alirocumab group with ≥ 2 consecutive LDL-C values < 25 mg/dL and propensity score-matched patients from the placebo group

	**All** **(*N* = 8610)**	**Alirocumab** **(*N* = 4305)**	**Placebo** **(*N* = 4305)**
Age, years	58.5 (9.3)	58.6 (9.2)	58.4 (9.4)
< 65 years	6335 (73.6%)	3165 (73.5%)	3170 (73.6%)
65 to < 75 years	1822 (21.2%)	911 (21.2%)	911 (21.2%)
≥ 75 years	453 (5.3%)	229 (5.3%)	224 (5.2%)
Male	6973 (81.0%)	3478 (80.8%)	3495 (81.2%)
Race			
White	6563 (76.2%)	3262 (75.8%)	3301 (76.7%)
Asian	1436 (16.7%)	734 (17.0%)	702 (16.3%)
Other/unknown	611 (7.1%)	309 (7.3%)	302 (7.0%)
Hispanic or Latino	1680 (19.5%)	858 (19.9%)	822 (19.1%)
Weight, kg	82.02 (16.68)	81.88 (16.69)	82.17 (16.68)
BMI, kg/m^2^	28.29 (4.68)	28.30 (4.70)	28.28 (4.66)
≥ 30 kg/m^2^	2721 (31.8%)	1344 (31.4%)	1377 (32.1%)
Region			
Asia	1359 (15.8%)	689 (16.0%)	670 (15.6%)
South America	1443 (16.8%)	734 (17.0%)	709 (16.5%)
Eastern Europe	2290 (26.6%)	1124 (26.1%)	1166 (27.1%)
Western Europe	1869 (21.7%)	921 (21.4%)	948 (22.0%)
North America	1088 (12.6%)	55 (12.9%)	534 (12.4%)
Rest of world	561 (6.5%)	283 (6.6%)	278 (6.5%)

Data are presented as mean ± standard deviation or n (%)

*BMI* Body mass index

**Table 3:** Baseline lipid parameters in patients on alirocumab with ≥ 2 consecutive LDL-C values < 25 mg/dL and propensity score-matched patients on placebo

	**All** **(*N* = 8610)**	**Alirocumab** **(*N* = 4305)**	**Placebo** **(*N* = 4305)**
LDL-C, mmol/L	2.1 ± 0.6	2.1 ± 0.6	2.16 ± 0
Non-HDL-C, mmol/L	2.9 ± 0.7	2.9 ± 0.7	2.9 ± 0.7
Total cholesterol, mmol/L	4.0 ± 0.8	4.0 ± 0.8	4.0 ± 0.8
HDL-C, mmol/L	1.1 ± 0.3	1.1 ± 0.3	1.1 ± 0.3
Fasting triglycerides, mmol/L	1.8 ± 1.0	1.8 ± 1.0	1.8 ± 1.1
Lipoprotein(a), mg/dL	28.6 ± 33.1	28.7 ± 33.3	28.7 ± 33.2
Apolipoprotein B, mg/dL	77.6 ± 17.3	78.1 ± 17.5	77.9 ± 17.4
Apolipoprotein A1, mg/dL	131.6 ± 23.0	131.2 ± 22.5	131.4 ± 22.7
Apolipoprotein B/Apolipoprotein A1 ratio^a^	0.61 ± 0.2	0.61 ± 0.2	0.61 ± 0.2
Total cholesterol/HDL-C ratio^a^	3.8 ± 1.0	3.8 ± 1.0	3.8 ± 1.0

Data are presented as mean ± SD

*HDL-C* High-density lipoprotein cholesterol, *LDL-C* Low-density lipoprotein cholesterol, *Q* Quartile, *SD* Standard deviation

^a^ Ratios were only calculated if the 2 samples were collected at the same visit

**Table 4:** Baseline demographics and characteristics of patients in the alirocumab group with ≥ 2 consecutive LDL-C values < 15 mg/dL and propensity score-matched patients from the placebo group

	**All** **(*N* = 3128)**	**Alirocumab** **(*N* = 782)**	**Placebo** **(*N* = 2346)**
Age, years	58.8 ± 9.5	58.9 ± 9.4	58.7 ± 9.5
< 65	2243 (71.7)	557 (71.2)	1686 (71.9)
65 to < 75	711 (22.7)	184 (23.5)	527 (22.5)
≥ 75	174 (5.6)	41 (5.2)	133 (5.7)
Male	2526 (80.8)	645 (82.5)	1881 (80.2)
Race			
White	1928 (61.6)	461 (59.0)	1467 (62.5)
Asian	951 (30.4)	253 (32.4)	698 (29.8)
Other/unknown	249 (8.0)	68 (8.7)	181 (7.7)
Hispanic or Latino	752 (24.0)	192 (24.6)	560 (23.9)
Weight, kg	77.9 ± 16.5	76.9 ± 16.6	78.2 ± 16.4
BMI, kg/m^2^	27.3 ± 4.5	27.2 ± 4.5	27.4 ± 4.4
≥ 30 kg/m^2^	784 (25.1)	191 (24.4)	593 (25.3)
Region			
Asia	919 (29.4)	240 (30.7)	679 (28.9)
South America	710 (22.7)	178 (22.8)	532 (22.7)
Eastern Europe	665 (21.3)	157 (20.1)	508 (21.7)
Western Europe	343 (11.0)	81 (10.4)	262 (11.2)
North America	336 (10.7)	85 (10.9)	251 (10.7)
Rest of world	155 (5.0)	41 (5.2)	114 (4.9)

Data are presented as mean ± standard deviation or n (%)

*BMI* Body mass index

**Table 5:** Baseline lipid parameters in patients on alirocumab with ≥ 2 consecutive LDL-C values < 15 mg/dL and propensity score-matched patients on placebo

	**All** **(*N* = 3128)**	**Alirocumab** **(*N* = 782)**	**Placebo** **(*N* = 2346)**
LDL-C, mmol/L	2.0 ± 0.5	2.0 ± 0.6	2.0 ± 0.5
Non-HDL-C, mmol/L	2.8 ± 0.7	2.8 ± 0.7	2.8 ± 0.6
Total cholesterol, mmol/L	3.9 ± 0.7	3.9 ± 0.8	3.9 ± 0.7
HDL-C, mmol/L	1.1 ± 0.3	1.1 ± 0.3	1.1 ± 0.3
Fasting triglycerides, mmol/L	1.8 ± 1.1	1.8 ± 0.9	1.8 ± 1.1
Lipoprotein(a), mg/dL	18.8 ± 23.1	19.3 ± 22.4	18.7 ± 23.3
Apolipoprotein B, mg/dL	75.4 ± 16.0	76.0 ± 17.5	75.2 ± 15.4
Apolipoprotein A1, mg/dL	129.9 ± 22.9	129.2 ± 22.3	130.1 ± 23.1
Apolipoprotein B/Apolipoprotein A1 ratio^a^	0.6 ± 0.2	0.6 ± 0.2	0.6 ± 0.2
Total cholesterol/HDL-C ratio^a^	3.7 ± 1.0	3.7 ± 0.9	3.7 ± 1.0

Data are presented as mean ± SD

*HDL-C* High-density lipoprotein cholesterol, *LDL-C* Low-density lipoprotein cholesterol, *Q* Quartile, *SD* Standard deviation

^a^ Ratios were only calculated if the 2 samples were collected at the same visit

**Fig. 2:**
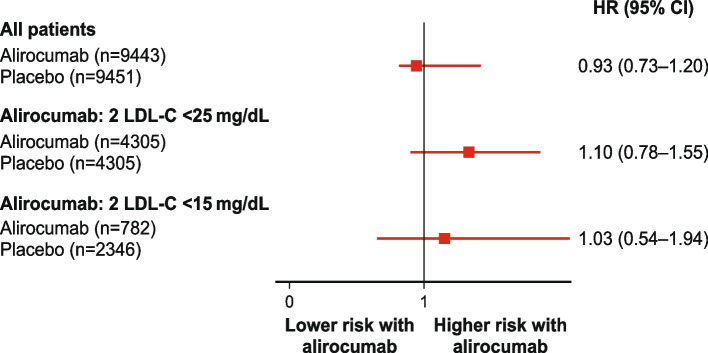
Hazard ratio for incident cataracts in all patients, and in patients achieving LDL-C levels < 25 or < 15 mg/dL with alirocumab, compared with propensity score-matched patients from the placebo group. CI, confidence interval; HR, hazard ratio; LDL-C, low-density lipoprotein cholesterol

As shown in Fig. 2, the incidence of cataracts was similar in patients treated with alirocumab with ≥ 2 LDL-C values < 25 mg/dL (71/4305, 1.6%) and in matched patients from the placebo group (60/4305 [1.4]; HR 1.10, CI 95% 0.78 − 1.55). Corresponding data for patients with ≥ 2 LDL-C values < 15 mg/dL (0.39 mmol/L) with alirocumab and in matched patients from the placebo group were 13/782 (1.7%) and 36/2346 (1.5%), respectively (HR 1.03, CI 95% 0.54 − 1.94). Thus, very low achieved LDL-C levels did not appear to associate with an increased incidence of cataracts.

## Discussion

Age-related cataract is the most common cause of visual impairment and blindness worldwide [1]. Cataract development can be induced by oxidative stress. It was hypothesized that the inhibition of cholesterol biosynthesis by statin medications (by a bidirectional effect on oxidation processes) can prevent proper epithelial cell development within the crystalline lens [4]. Administration of atorvastatin was noted to induce cataract in animal models [18].

Clinical trials of statins have yielded inconsistent findings regarding their effects on the risk of cataract. The HOPE-3 trial showed an increase of cataract surgery in patients treated with 10 mg rosuvastatin per day [19]. However, other sub-studies in patients achieving very low LDL-C did not find a correlation between very low LDL-C and cataract development [20, 21].

PCSK9 inhibitors are potent cholesterol-lowering drugs. They can reduce LDL-C to levels well below those achievable with statin therapy. The role of very low LDL-C levels in the onset of cataracts remains debated [9]. A pooled analysis of data from alirocumab trials showed that the incidence of cataracts was higher in patients with very low LDL C levels [14]. Other studies have not shown association of very low LDL-C levels achieved with statins and incidence of cataracts [21, 22], but did not use a propensity score or other approaches to match patients who achieved very low LDL-C with patients on statin treatment with patients from the corresponding control groups with similar baseline characteristics.

The present analysis demonstrates no overall increased risk of incident cataract with alirocumab versus placebo. Moreover, in analyses limited in the alirocumab group to patients who achieved very low levels of LDL-C, there was no evidence of an increased risk of cataract compared to patients from the placebo group with matched baseline characteristics including established cataract risk factors of age, smoking, diabetes, glycated hemoglobin A1c, and body mass index [9]. The analysis therefore adds to the evidence refuting an increased risk of cataracts in patients with very low LDL-C levels on lipid-lowering therapies.

### Strengths and limitations

This study involved a large, international clinical trial population with a high prevalence of cataract risk factors and clearly demonstrates the absence of risk of cataracts in patients treated with alirocumab. Moreover, to demonstrate the safety of very low LDL-C levels, propensity score-matching was used to assess a potential relationship of achieved LDL-C to risk of cataract [14, 23]. However, it should be stated that the analyses pertaining to very low achieved LDL-C levels did not account for certain cataract risk factors that were not ascertained in the trial, including socioeconomic status, malnutrition, large retinal drusen, prior ocular injury, and radiation exposure [1]. No systematic ophthalmoscopic examination was performed in the trial, and the presence of cataract was determined by clinical visual impairment or non-systematic ophthalmologic examination leading to a diagnosis. It is therefore likely that subclinical cataracts remained undiagnosed in the study population. Moreover, cataracts develop slowly, and the duration of exposure to alirocumab and resulting low LDL-C levels in ODYSSEY OUTCOMES may be too short to exclude a long-term effect on incidence or progression of cataracts. On the other hand, the study cohort had a high prevalence of cataract risk factors including smoking and diabetes and a substantial number of incident cataracts were identified. Lastly, even with the use of propensity score matching, an analysis based on a post-randomization variable such as achieved LDL-C should be considered exploratory.

## Conclusions

There was no increase in the incidence of cataract between patients receiving alirocumab or placebo. Moreover, very low achieved LDL-C levels on alirocumab treatment did not appear to increase the risk of cataracts in comparison to patients from the placebo group with similar baseline characteristics including established cataract risk factors. These results indicate that PCSK9 inhibition and resulting low LDL-C levels do not appear to modify the incidence of cataract over a median observation period of 2.8 years. Longer-term evaluation is indicated to add certainty to this conclusion.

## Supplementary Information

**Additional file 1.**

**Additional file 2.**

## Data Availability

The dataset(s) supporting the conclusions of this article is(are) included within the article.

## Abbreviations

ACS: Acute coronary syndrome
LDL-C: Low-density lipoprotein cholesterol
PCSK9: Proprotein convertase subtilisin–kexin type 9

## References

[1] Liu YC, Wilkins M, Kim T, Malyugin B, Mehta JS (2017). Cataracts. Lancet.

[2] Zelenka PS (1984). Lens lipids. Curr Eye Res.

[3] Cenedella RJ (1987). Inhibitors of cholesterol synthesis and cataracts. JAMA.

[4] Cenedella RJ (1996). Cholesterol and cataracts. Surv Ophthalmol.

[5] Karagiannis AD, Mehta A, Dhindsa DS, Virani SS, Orringer CE, Blumenthal RS, Stone NJ, Sperling LS (2021). How low is safe? The frontier of very low (<30 mg/dL) LDL cholesterol. Eur Heart J.

[6] Mach F, Ray KK, Wiklund O, Corsini A, Catapano AL, Bruckert E, De Backer G, Hegele RA, Hovingh GK, Jacobson TA (2018). Adverse effects of statin therapy: perception vs. the evidence - focus on glucose homeostasis, cognitive, renal and hepatic function, haemorrhagic stroke and cataract. Eur Heart J.

[7] Bentz AJ, Netland PJ, Newman WP, Froemke LL, Miller RR, Schiele RL (2018). Comparison of cardiovascular outcomes between statin monotherapy and fish oil and statin combination therapy in a veteran population. Fed Pract.

[8] Rosenson RS, Hegele RA, Fazio S, Cannon CP (2018). The evolving future of PCSK9 inhibitors. J Am Coll Cardiol.

[9] Dobrzynski JM, Kostis JB, Sargsyan D, Zinonos S, Kostis WJ (2018). Effect of cholesterol lowering with statins or proprotein convertase subtilisin/kexin type 9 antibodies on cataracts: a meta-analysis. J Clin Lipidol.

[10] Li J, Du H, Wang Y, Aertgeerts B, Guyatt G, Hao Q, Shen Y, Li L, Su N, Delvaux N (2022). Safety of proprotein convertase subtilisin/kexin 9 inhibitors: a systematic review and meta-analysis. Heart.

[11] Gaba P, O'Donoghue ML, Park JG, Wiviott SD, Atar D, Kuder JF, Im K, Murphy SA, De Ferrari GM, Gaciong ZA, et al. Association between achieved low-density lipoprotein cholesterol levels and long-term cardiovascular and safety outcomes: an analysis of FOURIER-OLE. Circulation. 2023;147(16):1192-203. 10.1161/CIRCULATIONAHA.122.063399 36779348

[12] Patti G, Spinoni EG, Grisafi L, Mehran R, Mennuni M (2023). Safety and efficacy of very low LDL-cholesterol intensive lowering: a meta-analysis and meta-regression of randomized trials. Eur Heart J Cardiovasc Pharmacother.

[13] Ray KK, Colhoun HM, Szarek M, Baccara-Dinet M, Bhatt DL, Bittner VA, Budaj AJ, Diaz R, Goodman SG, Hanotin C (2019). Effects of alirocumab on cardiovascular and metabolic outcomes after acute coronary syndrome in patients with or without diabetes: a prespecified analysis of the ODYSSEY OUTCOMES randomised controlled trial. Lancet Diabetes Endocrinol.

[14] Robinson JG, Rosenson RS, Farnier M, Chaudhari U, Sasiela WJ, Merlet L, Miller K, Kastelein JJ (2017). Safety of very low low-density lipoprotein cholesterol levels with alirocumab: pooled data from randomized trials. J Am Coll Cardiol.

[15] Yu S, Chu Y, Li G, Ren L, Zhang Q, Wu L (2017). Statin use and the risk of cataracts: a systematic review and meta-analysis. J Am Heart Assoc.

[16] Schwartz GG, Bessac L, Berdan LG, Bhatt DL, Bittner V, Diaz R, Goodman SG, Hanotin C, Harrington RA, Jukema JW (2014). Effect of alirocumab, a monoclonal antibody to PCSK9, on long-term cardiovascular outcomes following acute coronary syndromes: rationale and design of the ODYSSEY outcomes trial. Am Heart J.

[17] Schwartz GG, Steg PG, Szarek M, Bhatt DL, Bittner VA, Diaz R, Edelberg JM, Goodman SG, Hanotin C, Harrington RA (2018). Alirocumab and cardiovascular outcomes after acute coronary syndrome. N Engl J Med.

[18] Zakrzewski P, Milewska J, Czerny K (2002). The eye lens evaluation of the atorvastatin-treated white rat. Ann Univ Mariae Curie Sklodowska Med.

[19] Yusuf S, Bosch J, Dagenais G, Zhu J, Xavier D, Liu L, Pais P, Lopez-Jaramillo P, Leiter LA, Dans A (2016). Cholesterol lowering in intermediate-risk persons without cardiovascular disease. N Engl J Med.

[20] Giugliano RP, Wiviott SD, Blazing MA, De Ferrari GM, Park JG, Murphy SA, White JA, Tershakovec AM, Cannon CP, Braunwald E (2017). Long-term safety and efficacy of achieving very low levels of low-density lipoprotein cholesterol : a prespecified analysis of the IMPROVE-IT trial. JAMA Cardiol.

[21] Everett BM, Mora S, Glynn RJ, MacFadyen J, Ridker PM (2014). Safety profile of subjects treated to very low low-density lipoprotein cholesterol levels (<30 mg/dl) with rosuvastatin 20 mg daily (from JUPITER). Am J Cardiol.

[22] Wiviott SD, Cannon CP, Morrow DA, Ray KK, Pfeffer MA, Braunwald E, Investigators PI-T (2005). Can low-density lipoprotein be too low? The safety and efficacy of achieving very low low-density lipoprotein with intensive statin therapy: a PROVE IT-TIMI 22 substudy. J Am Coll Cardiol.

[23] Elze MC, Gregson J, Baber U, Williamson E, Sartori S, Mehran R, Nichols M, Stone GW, Pocock SJ (2017). Comparison of propensity score methods and covariate adjustment: evaluation in 4 cardiovascular studies. J Am Coll Cardiol.

