# Correction: Effect of alirocumab on cataracts in patients with acute coronary syndromes

**DOI:** 10.1186/s12886-023-03065-2

**Published:** 2023-07-06

**Authors:** Gaspard Suc, Gregory G. Schwartz, Shaun G. Goodman, J. Wouter Jukema, Garen Manvelian, Yann Poulouin, Robert Pordy, Michel Scemama, Michael Szarek, Ph.Gabriel Steg, ODYSSEY OUTCOMES Investigators

**Affiliations:** 1 Université Paris-Cité, INSERM_U1148/LVTS, Paris, France; 2 Assistance Publique-Hôpitaux de Paris, HôpitalBichat, Paris, France; 3 Division of Cardiology, University of Colorado School of Medicine, Aurora, CO USA; 4 Canadian VIGOUR Centre, University of Alberta, Edmonton, AB Canada; 5 St. Michael’s Hospital, University of Toronto, Toronto, ON Canada; 6 Department of Cardiology, Leiden University Medical Center, Leiden, the Netherlands; 7 Netherlands Heart Institute, Utrecht, the Netherlands; 8 Regeneron Pharmaceuticals, Tarrytown, NY USA; 9 IT&M Stats, Neuilly-Sur-Seine, France; 10 Sanofi, Chilly-Mazarin, France; 11 Downstate School of Public Health, State University of New York, Brooklyn, NY USA; 12 CPC Clinical Research and Division of Cardiology, University of Colorado School of Medicine, Aurora, CO USA; 13 Institut Universitaire de France, Paris, France; 14 FACT (French Alliance for Cardiovascular Trials), INSERM U-1148, Paris, France; 15 Département de Cardiologie, AP-HP Hôpital Bichat, 46 Rue Henri Huchard, Paris, 75018 France

**Correction: BMC Ophthalmol 23, 279 (2023)**

**https://doi.org/10.1186/s12886–023-03012-1**

After publication of the original article [1], the author group noticed a calculation error that has affected Tables 3 and 5 as well as the second paragraph of the Results section in page 4.

The original article has been updated and the correct values are also given below in bold.

**Table 3:** Baseline lipid parameters in patients on alirocumab with ≥ 2 consecutive LDL-C values < 25 mg/dL and propensity score-matched patients on placebo

	**All** **(** ***N*** ** = 8610)**	**Alirocumab** **(** ***N*** ** = 4305)**	**Placebo** **(** ***N*** ** = 4305)**
LDL-C, mmol/L	2.1 ± 0.6	2.1 ± 0.6	2.16 ± 0.
Non-HDL-C, mmol/L	2.9 ± 0.7	2.9 ± 0.7	2.9 ± 0.7
Total cholesterol, mmol/L	4.0 ± 0.8	4.0 ± 0.8	4.0 ± 0.8
HDL-C, mmol/L	1.1 ± 0.3	1.1 ± 0.3	1.1 ± 0.3
Fasting triglycerides, mmol/L	1.8 ± 1.0	1.8 ± 1.0	1.8 ± 1.1
**Lipoprotein(a), mg/dL**	**28.6 ± 33.1**	**28.7 ± 33.3**	**28.7 ± 33.2**
**Apolipoprotein B, mg/dL**	**77.6 ± 17.3**	**78.1 ± 17.5**	**77.9 ± 17.4**
**Apolipoprotein A1, mg/dL**	**131.6 ± 23.0**	**131.2 ± 22.5**	**131.4 ± 22.7**
Apolipoprotein B/Apolipoprotein A1 ratio^a^	0.61 ± 0.2	0.61 ± 0.2	0.61 ± 0.2
Total cholesterol/HDL-C ratio^a^	3.8 ± 1.0	3.8 ± 1.0	3.8 ± 1.0

Data are presented as mean ± SD

*HDL-C* high-density lipoprotein cholesterol, *LDL-C* low-density lipoprotein cholesterol, *Q* quartile, *SD* standard deviation

^a^ Ratios were only calculated if the 2 samples were collected at the same visit

**Table 5:** Baseline lipid parameters in patients on alirocumab with ≥ 2 consecutive LDL-C values < 15 mg/dL and propensity score-matched patients on placebo

	**All** **(** ***N*** ** = 3128)**	**Alirocumab** **(** ***N*** ** = 782)**	**Placebo** **(** ***N*** ** = 2346)**
LDL-C, mmol/L	2.0 ± 0.5	2.0 ± 0.6	2.0 ± 0.5
Non-HDL-C, mmol/L	2.8 ± 0.7	2.8 ± 0.7	2.8 ± 0.6
Total cholesterol, mmol/L	3.9 ± 0.7	3.9 ± 0.8	3.9 ± 0.7
HDL-C, mmol/L	1.1 ± 0.3	1.1 ± 0.3	1.1 ± 0.3
Fasting triglycerides, mmol/L	1.8 ± 1.1	1.8 ± 0.9	1.8 ± 1.1
Lipoprotein(a), mg/dL	**18.8 ± 23.1**	**19.3 ± 22.4**	**18.7 ± 23.3**
Apolipoprotein B, mg/dL	**75.4 ± 16.0**	**76.0 ± 17.5**	**75.2 ± 15.4**
Apolipoprotein A1, mg/dL	**129.9 ± 22.9**	**129.2 ± 22.3**	**130.1 ± 23.1**
Apolipoprotein B/Apolipoprotein A1 ratio^a^	0.6 ± 0.2	0.6 ± 0.2	0.6 ± 0.2
Total cholesterol/HDL-C ratio^a^	3.7 ± 1.0	3.7 ± 0.9	3.7 ± 1.0

Data are presented as mean ± SD

*HDL-C* high-density lipoprotein cholesterol, *LDL-C* low-density lipoprotein cholesterol, *Q* quartile, *SD* standard deviation.

^a^ Ratios were only calculated if the 2 samples were collected at the same visit.

The second paragraph in page 4 should read:

A total of 4305 patients in the alirocumab group had ≥ 2 consecutive LDL-C values < 25 mg/dL (0.65 mmol/L) and were matched to 4305 patients from the placebo group with similar baseline characteristics (Tables 2 and 3). Baseline characteristics of these patients included mean age 59 years, male sex (81%), diabetes (33%); and mean body mass index 28.3 kg/m2, LDL-C 2.1 mmol/L, lipoprotein (a) **28.6** mg/dL, and apolipoprotein A1 **131.6** mg/dL. A total of 782 patients in the alirocumab group had ≥ 2 consecutive LDL-C values < 15 mg/dL (0.39 mmol/L) and were matched to 2346 patients from the placebo group with similar baseline characteristics (Tables 4 and 5). Baseline characteristics of these patients included mean age 59 years, male sex (81%), diabetes (33%); and mean body mass index 27.3 kg/m2, LDL-C 2.0 mmol/L, lipoprotein (a) **18.8** mg/dL, and apolipoprotein A1 **129.9** mg/dL.
