# Supplementary material for: Effect of alirocumab on cataracts in patients with acute coronary syndromes
Source: BMC Ophthalmol. 2023 Jun 16;23:279. doi: 10.1186/s12886-023-03012-1 (PMC10276414; doi:10.1186/s12886-023-03012-1)
Supplement: Supplementary file 1 — Additional file 1. [file 12886_2023_3012_MOESM1_ESM.pdf]

ODYSSEY OUTCOMES

ClinicalTrials.gov NCT01663402

This supplement contains the following items:

	Pages
Original protocol*	2-142
Final protocol*	143-314
Summary of changes	315-318
Original statistical analysis plan	319-402
Final statistical analysis plan, including summary of changes (pages 413-416)	403-492

*Proprietary methods for assessment of anti-drug and neutralizing antibodies have been redacted. The EQ-5D questionnaire is proprietary, cannot be reproduced without permission, and is therefore redacted. A copy of the EQ-5D may be obtained from EuroQol, Marten Meesweg 107, 3068 AV Rotterdam, The Netherlands.

CLINICAL TRIAL PROTOCOL

TITLE: A Randomized, Double-Blind, Placebo-Controlled, Parallel-Group Study to Evaluate the Effect of SAR236553/REGN727 on the Occurrence of Cardiovascular Events in Patients Who Have Recently Experienced an Acute Coronary Syndrome

COMPOUND: SAR236553/REGN727

STUDY NUMBER: EFC11570

STUDY NAME: ODYSSEY Outcomes

Version Number:	1	IND Number	IND: 105574
		EudraCT number	2011-005698-21
Date:	19-Jul-2012	Total number of pages:	141

Any and all information presented in this document shall be treated as confidential and shall remain the exclusive property of Sanofi (or any of its affiliated companies). The use of such confidential information must be restricted to the recipient for the agreed purpose and must not be disclosed, published or otherwise communicated to any unauthorized persons, for any reason, in any form whatsoever without the prior written consent of Sanofi (or the concerned affiliated company); 'affiliated company' means any corporation, partnership or other entity which at the date of communication or afterwards (i) controls directly or indirectly Sanofi, (ii) is directly or indirectly controlled by Sanofi, with 'control' meaning direct or indirect ownership of more than 50% of the capital stock or the voting rights in such corporation, partnership or other entity

NAMES AND ADDRESSES OF

**COORDINATING INVESTIGATOR**	Name:<br>Address:<br><br>Tel:<br>Fax:<br>E-mail:	
**MONITORING TEAM'S REPRESENTATIVE**	Name:<br>Address:<br><br>Tel:<br>Fax:<br>E-mail:	
**SPONSOR**	Company:<br>Address:	
**OTHER EMERGENCY TELEPHONE NUMBERS**		

CLINICAL TRIAL SUMMARY

COMPOUND: SAR236553/REGN27 STUDY No: EFC11570	
**TITLE**	A Randomized, Double-Blind, Placebo-Controlled, Parallel-Group Study to Evaluate the Effect of SAR236553/REGN727 on the Occurrence of Cardiovascular Events in Patients Who Have Recently Experienced an Acute Coronary Syndrome
**INVESTIGATOR/TRIAL LOCATION**	Worldwide – multicenter study
**PHASE OF DEVELOPMENT**	IIIb
**STUDY OBJECTIVE(S)**	**Primary objective**<br>The primary objective of this study is to compare the effect of SAR236553 with placebo on the occurrence of cardiovascular events (composite endpoint of coronary heart disease (CHD) death, non-fatal myocardial infarction (MI), fatal and non-fatal ischemic stroke, unstable angina requiring hospitalization) in patients who have experienced an acute coronary syndrome (ACS) event 4 to 16 weeks prior to randomization and are treated with intensive statin therapy (defined as atorvastatin 40 or 80 mg, or rosuvastatin 20 or 40 mg) or at maximally tolerated dose of these given statins, or other non statin LMT(s).<br>**Secondary objective(s)**<br>To compare the efficacy of SAR236553 versus placebo on secondary endpoints (any CHD event, major CHD event, any CV event, composite of all cause mortality/non-fatal MI/non-fatal ischemic stroke, all cause mortality).<br>*A Clinical Events Committee (CEC) will be established to adjudicate, in a blinded fashion, events corresponding to the primary and secondary cardiovascular endpoints as well as all causes of death.*<br>• To evaluate the safety and tolerability of SAR236553 throughout the study.<br>• To evaluate the development of anti-SAR236553 antibodies.<br>• To evaluate the effect of SAR236553 on low-density lipoprotein cholesterol (LDL-C), apolipoprotein B (ApoB), and non-high-density lipoprotein cholesterol (non-HDL-C).
**STUDY DESIGN**	This is a double-blind, randomized, placebo-controlled, parallel-group study, multi-national, multicenter study. Randomization will be stratified according to country.<br>The study will comprise 3 periods:<br>• A run-in period during which the background lipid-modifying therapy (LMT) will be adjusted as needed, to ascertain the patient is receiving the required intensive statin treatment (defined as atorvastatin 40 or 80 mg, or rosuvastatin 20 or 40 mg) or the maximally tolerated doses of these given statins, or other non statin LMTs prior to randomization, and to stabilize this treatment.<br>• A double-blind treatment period during which the background LMT will not be modified (except for safety reasons).

	• A follow-up contact 8 weeks (i.e., 2 months) after the final visit (Visit 30, Month 64) that corresponds to the common study end date. This follow-up contact only applies for patients still on study treatment at the time of the common study end date.<br>**1/ The run-in period** will start by a screening visit during which patients will be evaluated for eligibility to enter this run-in period. This screening visit can occur as soon as the day of the index ACS event, but no later than 12 weeks after the index event. Then a qualifying Visit (V2, Week-2) will be performed within 2 weeks (± 3 days) prior to randomization to obtain the results of the qualifying lipid parameters.<br>*Note: An optional visit (V2b) and central laboratory assessments may occur during the run-in period if modifications to background statin or other LMTs are made after the initial qualifying visit (V2), to ascertain that lipid criteria are fulfilled after these modifications and within 2 weeks prior to randomization.*<br>During this run-in period the following rules will be applied:<br>• All patients will receive atorvastatin or rosuvastatin as follows:<br>- Following the index ACS event, intensive statin therapy as defined by atorvastatin 40 or 80 mg, or rosuvastatin 20 or 40 mg daily will be given to statin naïve patients as well as to patients previously treated with other statins or with lower doses of atorvastatin or rosuvastatin.<br>- However:<br>o An initial dose of atorvastatin lower than 40/ 80 mg or rosuvastatin 20/40mg may be chosen for patients who are known to be intolerant to these doses. The maximum tolerated dose of atorvastatin or rosuvastatin will be determined by the investigator.<br>o An initial dose of atorvastatin lower than 40/ 80 mg or rosuvastatin 20/40mg may be chosen for patients in whom the investigator considers such dose prudent due to advanced age, low body mass index or other concerns. If the initial dose is tolerated, the dose may afterwards be increased up to atorvastatin 40 or 80 mg or rosuvastatin 20 or 40 mg, at the investigator's discretion but no later than 12 weeks after the index ACS event.<br>o Atorvastatin or rosuvastatin dose may be decreased from the initial dose in patients who develop adverse events (e.g, myalgia), in accordance with usual guidance.<br>o Any change in the dose of atorvastatin or rosuvastatin must have occurred at least 2 weeks prior to the qualifying Visit (V2, Week-2).<br>o Patients who do not tolerate the minimum approved dose of atorvastatin (10 mg) or rosuvastatin (5 mg) may nonetheless qualify for the trial without any ongoing atorvastatin/rosuvastatin therapy, provided that they are compliant with all other inclusion and exclusion criteria and they are on stable treatment with other non statin LMTs for at least 4 weeks prior to the qualifying Visit (V2, Week-2).

- LMTs other than statin can be maintained during the run-in period, as long as qualifying labs are obtained (V2, Week-2) a minimum of 4 weeks after any change in dose of non-statin LMT; for fibrates, only fenofibrate is allowed.
- Ezetimibe (but not other LMTs) can be initiated during the run-in period as long as qualifying labs are obtained (V2, Week-2) a minimum of 4 weeks after any change in dose of ezetimibe.

In summary, *the run-in period ends with a randomization visit (V3) that occurs 4 weeks to 16 weeks (+3 days)after the index ACS event and no later than 2 weeks (± 3 days) after qualifying Visit (V2):*

- Qualifying (central) lipid parameters are obtained at the qualifying Visit (V2) when all of the following conditions have been met: at least 2 weeks have elapsed after index ACS event, at least 2 weeks have elapsed after any change in atorvastatin or rosuvastatin dose, and at least 4 weeks have elapsed after any change in non-statin LMT.
- Randomization (at Visit V3) may take place as soon as it is verified that qualifying V2 labs fulfill laboratory inclusion criteria, (and no exclusion criteria are met), and should occur no later than 2 weeks (± 3 days) after visit V2 and no later than16 weeks (+ 3 days) after index ACS event.

During this run-in period, patients (or their caregivers) will administer at least 2 subcutaneous injections of the placebo SAR236553 in order to train them to administer the self-injection and to ensure patient's acceptance of an injectable study treatment. These training injections should be performed on site at screening Visit (V1, Week-16 to Week-4) and qualifying Visit (V2, Week-2), respectively. A third training injection can be performed at an additional visit such as the optional visit V2b

2/ The double-blind treatment period will continue until each surviving randomized patient has been followed for a minimum of 24 months or the target number of events (1613) is reached whichever comes last. The corresponding estimated study duration is 64 months (as described in the sample size considerations – Section 11.1).

During this double-blind treatment period, the dosing of SAR236553 is intended to achieve LDL-C levels of 30-50 mg/dL (0.78-1.29 mmol/L) which is considered as the physiologic ideal level and to avoid levels of LDL-C that are clearly below the physiologic range (i.e.,<15 mg/dL or 0.39 mmol/L)). To achieve this goal the following up-titration or down-titration scheme (including discontinuation if needed) will be applied:

- At randomization Visit (V3), the starting dose of SAR236553 will be 75 mg every 2 weeks (Q2W). At Month 2, patients randomized to SAR236553 will, in a blinded manner, either:
    - Continue SAR236553 75 mg Q2W, if the Month 1 LDL-C is <50 mg/dL (1.29 mmol/L) **OR**
    - Be up-titrated to SAR236553 150 mg Q2W, if the Month 1 LDL-C is ≥ 50 mg/dL (1.29 mmol/L).
- At subsequent visits, for patients on SAR236553, the following adjustments may be applied depending on the dose received:

	- For patients receiving 150 mg Q2W: if LDL-C < 25 mg/dL (0.65 mmol/L) (including LDL-C < 15 mg/dL[0.39 mmol/L]) on 2 consecutive measurements, down-titration to 75 mg Q2W will be done in a blinded manner at the next visit. Additional monitoring will be implemented until the down-titration is done.<br>- For patients receiving 75 mg Q2W:<br>o If LDL-C < 25 mg/dL (0.65 mmol/L) but ≥ 15 mg/dL (0.39 mmol/L) on 2 consecutive measurements: SAR236553 will be continued but additional monitoring will be implemented, to further confirm the safety of low LDL-C levels.<br>o If LDL-C < 15 mg/dL (0.39 mmol/L) on 2 consecutive measurements: SAR236553 treatment will be discontinued at the next visit. In order to maintain the blind there will be blinded substitution to placebo injections for the remaining duration of the study. Additional monitoring will be implemented until the study treatment discontinuation is done.<br>As lipid values will not be communicated to investigators after randomization to maintain the blind, the continuation of the 75 mg dose, the up-titration to the 150 mg dose, the down-titration from 150 to 75 mg, or discontinuation will occur automatically, without site or patient awareness. Moreover, blinded dose adjustment will avoid the need for dummy titrations in the placebo group.<br>Atorvastatin or rosuvastatin daily dose, as well as dose of other non statin LMT (if applicable), is required to be stable from randomization up to the end of the study, unless safety reasons prompt dose reduction or discontinuation.<br>**3/ A follow-up contact** will only be applicable for patients still on study treatment at the time of the common study end date; this follow-up contact should be scheduled 8 weeks (i.e., 2 months) after the common study end date and will be a phone call.<br>Patients should be on a stable diet (NCEP-ATPIII TLC diet or equivalent) throughout the entire study duration from screening to the end of study.<br>*Note: all patients, even those who have achieved an endpoint or prematurely discontinued the study treatment, will be followed, as scheduled by the protocol, from randomization until the common study end date.*
**STUDY POPULATION**<br>**Main selection criteria**	**Inclusion criteria**<br>• Hospitalization for ACS defined by:<br>- Ischemic symptoms with unstable pattern, occurring at rest or minimal exertion within 24 hrs of an unscheduled hospital admission, due to presumed or proven obstructive coronary disease **AND at least one of the following:**<br>o elevated cardiac biomarkers **OR**<br>o Resting ECG changes consistent with ischemia or infarction **AND** additional evidence of obstructive coronary disease.<br>• Patients not adequately controlled (as defined by at least one of the following: LDL-C ≥ 70 mg/dL [≥ 1.81 mmol/L], ApoB ≥ 80 mg/dL [≥ 0.8 mmol/L], or non-HDL-C ≥ 100 mg/dL [≥ 2.59 mmol/L]) at the qualifying Visit (V2), despite evidence-based lipid lowering therapy (including

	intensive atorvastatin/rosuvastatin therapy or maximally tolerated dose of either of these 2 statins, or other non statin LMTs).<br>**Exclusion criteria**<br>***For the entry into the run-in period and the double-blind treatment period (randomization)***<br>• All of the following: LDL-C < 70 mg/dL (< 1.81 mmol/L), ApoB < 80 mg/dL (< 0.8 mmol/L), and non-HDL-C < 100 mg/dL (< 2.59 mmol/L) at the qualifying Visit (V2).<br>• Age < 40 years.<br>• Patients who have experienced an ACS event more than 16 weeks (+ 3 days) prior to randomization visit (V3).<br>• Not on a stable dose of atorvastatin or rosuvastatin for at least 2 weeks prior to qualifying visit (V2 or V2b) and at least 4 weeks prior to randomization visit (V3), or not on a stable dose of any other authorized LMT for at least 4 weeks prior to qualifying visit (V2 or V2b) and at least 6 weeks prior to randomization visit (V3, Month 0).<br>• Uncontrolled hypertension (SBP > 180 mmHg or DBP > 110 mmHg) at V2 or V3.<br>• New York Heart Association class III or IV congestive heart failure persisting despite treatment or if measured LVEF < 25%.<br>• Known history of hemorrhagic stroke.<br>• Fasting serum triglycerides (TG) > 400 mg/dL (> 4.52 mmol/L) prior to randomization.
**Total expected number of patients**	Approximately 18000 patients should be randomized (9000 patients / group).<br>Approximately 1400 sites
**STUDY TREATMENT(s)**<br>**Investigational medicinal product(s)**	SAR236553
**Formulation**	Sterile SAR236553drug product supplied at a concentration of 75 mg/mL or 150 mg/mL in 10 mM histidine, pH 6.0, 0.2% (w/v) polysorbate 20, and 10% (w/v) sucrose
**Route(s) of administration**	Subcutaneous (SC) injection in the abdomen, thigh or outer area of upper arm
**Dose regimen**	75 mg every 2 weeks **OR**<br>75 mg every 2 weeks up to Month 2 followed by 150 mg every 2 weeks at Month 2 onwards (with criteria for up-titration and down-titration as indicated above).
**INJECTION FOR TRAINING AND PLACEBO COMPARATOR**<br>**Investigational medicinal product(s) (if applicable)**	Placebo
**Formulation**	Same formulation as SAR236553 without the addition of protein.

**Route(s) of administration**	Subcutaneous (SC) injection in the abdomen, thigh or outer area of upper arm.
**ENDPOINT(S)**	**Primary endpoint**<br>• Time from randomization to first occurrence of one of the following Clinical Events, as determined by the CEC:<br>- CHD death.<br>- Any non-fatal MI.<br>- Fatal and non-fatal ischemic stroke.<br>- Unstable angina requiring hospitalization.<br>**Main Secondary Efficacy Endpoint(s):**<br>• Time from randomization to first occurrence of any CHD event (major CHD event, unstable angina requiring hospitalization, hospitalization for unanticipated coronary revascularization procedure).<br>• Time from randomization to first occurrence of any major CHD event (CHD death, non-fatal MI).<br>• Time from randomization to first occurrence of any CV event defined as follows: any non-fatal CHD event, any CV death, and non-fatal ischemic stroke.<br>• Time from randomization to first occurrence of all cause mortality, non-fatal MI, non-fatal ischemic stroke.<br>• Time from randomization to death (all cause mortality).<br>**Other Secondary Efficacy Endpoint(s):**<br>• Components of the primary end point considered individually: CHD death, or non-fatal MI, or fatal and non-fatal ischemic stroke, unstable angina requiring hospitalization.<br>• Hospitalization for unanticipated coronary revascularization procedure.<br>• Congestive heart failure requiring hospitalization.<br>**Safety Endpoint(s):**<br>• Safety endpoints: all adverse events, heart rate and blood pressure, hematology and biochemistry assessments.<br>**Other Endpoint(s):**<br>• Anti-SAR236553 antibodies assessed throughout the study.<br>• The percent change in calculated LDL-C, in ApoB and non HDL-C.
**ASSESSMENT SCHEDULE**	**Visits scheduled during the run-in period:**<br>The run-in period should have a minimum duration of 4 weeks. The run-in period cannot begin later than 12 weeks after the index ACS event, to allow

	randomization no later than 16 weeks (+ 3 days) after the index ACS event.<br><br>Screening Visit can be performed at the maximum 16 weeks (+ 3 days) and at the minimum 4 weeks prior to randomization. After this screening Visit (V1) patients will enter the run-in period of at least 4 weeks.<br><br>During the run-in period, patients will attend at least the qualifying Visit (V2) to be performed within 2 weeks (± 3 days) prior to randomization to obtain the laboratory parameters required for eligibility. If necessary, one additional visit (V2b) and central laboratory assessments may occur during the run-in period if modifications to background statin or other LMTs are made after an initial visit V2, to ensure that lipid criteria are nonetheless fulfilled prior to randomization.<br>*Note: An optional visit (V2b) and central laboratory assessments may occur during the run-in period if modifications to background statin or other LMTs are made after the initial qualifying visit (V2), to ascertain that lipid criteria are fulfilled after these modifications and within 2 weeks prior to randomization.*<br><br>Randomization visit (V3) should occur no later than 2 weeks (± 3 days) after qualifying Visit (V2), no later than 16 weeks (+ 3 days) after the index ACS event, and also no sooner than 4 weeks after the screening visit (V1).<br><br>At the end of this run-in period, the randomization Visit (V3, Month 0) will be performed, and eligible patients will be randomized to the double-blind study treatment period.<br><br>**Visits schedule during the double-blind treatment period:**<br><br>• On-site visits:<br>- For the first year: visit at Month1, Month 2, Month 4 and then every 4 months (Month 8, Month 12).<br>- For the second year: visit every 4 months.<br>- For the subsequent years: visit every 6 months up to the end of the study.<br><br>**Contacts schedule during the double-blind treatment period:**<br><br>• Once on a 4-month visit schedule and then continuing on a 6-month visit schedule, at least one phone call or contact via internet (as permitted by institutional privacy policies) should occur in between each on-site visit.<br><br>**Follow-up contact (only for patients still on study treatment at the common study end date):**<br><br>• Phone call to be scheduled 8 weeks (i.e., 2 months) after the common study end date.
**STATISTICAL CONSIDERATIONS**	**Sample size determination:**<br>Based on the targeted population for the study, a Kaplan-Meier event rate in placebo group of 11.4% at 48 months (3.8% at 12 months, 6.4% at 24 months, 9.0% at 36 months) is assumed. With a 40-month recruitment period, a 64-month total study duration, 9000 patients per group (total 18,000), and 1,613 patients experiencing at least one primary endpoint event, the study has 90% power (one-sided Logrank test at the overall 0.025 alpha level) assuming a 15% risk reduction associated with SAR236553 treatment. The

	sample size calculation takes into account two interim analyses.<br>**Analysis Populations:**<br>Randomized population includes any patients who have been allocated to a randomized treatment regardless of whether the treatment kit was used or not.<br>Efficacy analyses will be performed on the intention-to-treat (ITT) population, consisting of all randomized patients. Patients in the ITT population will be analyzed according to the treatment group allocated by randomization.<br>The safety population consists of the randomized population who actually received at least one dose or partial dose of IMP analyzed according to the treatment actually received.<br>**Primary Analysis:**<br>The primary endpoint will be compared between treatment groups by a log-rank test, stratified by region (North America, South America, Western Europe, Eastern Europe, Asia, other region). The distribution will be estimated by treatment group with Kaplan-Meier methodology. Treatment hazard ratios (HRs) for the primary endpoint will be estimated from Cox regression models stratified by region.<br>**Analysis of the main secondary endpoints**<br>A hierarchical procedure will be used to control the type I error and handle multiple endpoints. If the primary endpoint analysis is significant (at the 0.0001 one-sided alpha level at the second interim analysis or at the 0.0249 one-sided alpha level at the final analysis), main secondary efficacy endpoints will be tested sequentially, using the order defined in section "Primary and secondary endpoints".<br>Secondary endpoints will be analyzed in the ITT population using the same statistical methodology as for the primary endpoint (time-to-event analysis).<br>**Safety analysis**<br>Safety analysis (adverse events, laboratory, vital signs) will be descriptive, based on the safety population. The safety analysis will focus on the Treatment Emergent Adverse Event (TEAE) period. This period is defined as the time from the first to the last dose of double-blind IMP + 70 days (10 weeks).<br>**Interim analyses:**<br>Patients will be followed until 1,613 patients experience at least one primary endpoint event or for approximately 24 months after the date of the last randomized patient, whichever comes last.<br>Interim analyses for futility will be performed, under the supervision of the CV DMC, when 50% and 75% of events have occurred. An interim analysis for efficacy will be performed when 75% of events have occurred. Control of the type I and type II error will be ensured using gamma (-5) spending function for type II error (futility) and Gamma (-22) for type I error (efficacy). Stopping rules details are further described in Section 11.5.
**DURATION OF STUDY PERIOD (per patient)**	The duration of the run-in period should be at least 4 weeks, and randomization should occur no later than16 weeks (+ 3 days) after the index ACS event.<br>The double-blind treatment period will continue until each surviving

	randomized patient has been followed for a minimum of 24 months or the target number of events (1613) is reached, whichever comes last. The corresponding estimated study duration is 64 months (as described in the sample size considerations).<br><br>All patients, even those who have achieved an endpoint or prematurely discontinued study treatment, will be followed from randomization until the common study end date.

1 FLOW CHARTS

1.1 GRAPHICAL STUDY DESIGN

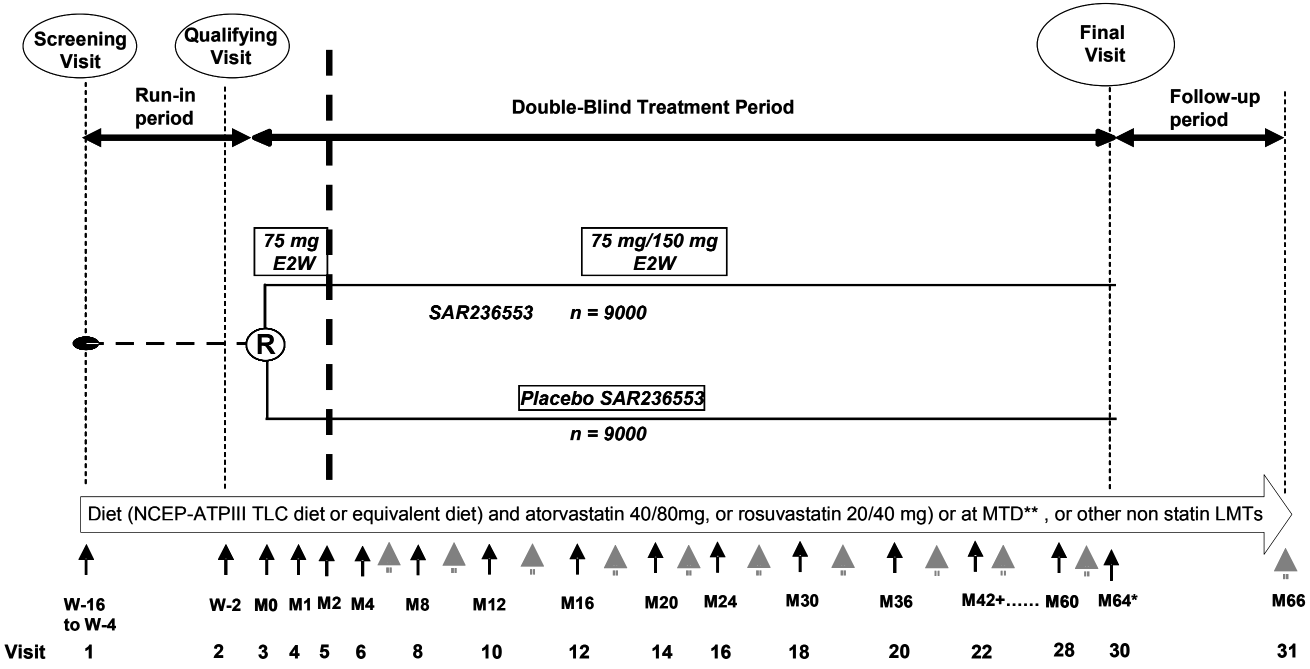

Contact phone calls/Internet | On site visit | Randomization

+ Visit every 6 months

* Final visit can occur between 24 months and 64 months of follow-up, when the last randomized patient reaches a 24-month follow-up or the target number of events (1613) is reached, whichever comes last, and will determine the common study end date. Final V30 (Month 64) will be completed for all patients regardless of the compliance with study treatment.

Note: an early end of treatment visit (V70) should be performed within 2 months for patients who prematurely discontinue the study treatment; assessments performed at this visit are similar to those to be performed for study completers at Final visit (V30). for study completers at Final visit (V30). Prematurely discontinued patients will be maintained in the study until the common study end date or Final visit (V30).

**MTD: maximally tolerated dose.

1.2 STUDY FLOW CHART

1.2.1 On-site visits during the study

	Screening including run-in period		Double blind period											
**On-site Visit**	V1	V2[b]	V3	V4	V5	V6	V8	V10	V12/V14/V18/V22/V26	V16/V20/V24/V28	V30 [m]		V70[n]	V31
**Week (W)/ Month (M)** [a]	W-16 to W-4	W-2	M0 (D1)	M1	M2	M4	M8	M12	M16/M20/M30/M42/M54	M24/M36/M48/M60	M64 Final visit (common study end date)		Early end of treatment visit	M66 Follow-up visit (phone call)
											Study completers	Prematurely discontinued patients		
**Design:**														
Informed Consent/Patient Demography	X													
Inclusion/Exclusion Criteria	X	X	X											
Medical/surgical history, alcohol habits, smoking habits	X													
Prior Medication History[c]	X													
Physical Examination	X		X					X		X	X		X	
Body weight	X		X			X	X	X	X	X	X		X	
Height	X													
Randomization			X											
Patient diary dispensation/ review / collection[d]			X	X	X	X	X	X	X	X	X		X	
IVRS/IWRS contact	X	X	X		X	X	X	X	X	X	X	X	X	X

	Screening including run-in period		Double blind period											
**On-site Visit**	V1	V2[b]	V3	V4	V5	V6	V8	V10	V12/V14/V18/V22/V26	V16/V20/V24/V28	V30 [m]		V70[n]	V31
**Week (W)/ Month (M)** [a]	W-16 to W-4	W-2	M0 (D1)	M1	M2	M4	M8	M12	M16/M20/M30/M42/M54	M24/M36/M48/M60	M64 Final visit (common study end date)		Early end of treatment visit	M66 Follow-up visit (phone call)
											Study completers	Prematurely discontinued patients		
**<u>Treatment:</u>**														
Injection training	X[e]	X[e]	X[f]											
Double Blind Investigational Medicinal Product (IMP) kit dispensation			X		X	X	X	X	X	X				
Compliance check of IMP (review patient diary and treatment kit) and data collection on IMP administration				X	X	X	X	X	X	X	X		X	
Compliance check for atorvastatin, rosuvastatin, ± other LMTs		X	X	X	X	X	X	X	X	X	X	X	X	
Concomitant Medication[c]	X	X	X	X	X	X	X	X	X	X	X	X	X	X
**<u>Vital signs:</u>**														
Heart rate, blood pressure	X	X	X	X	X	X	X	X	X	X	X	X	X	
**<u>Safety:</u>**														
AE /SAE recording	X	X	X	X	X	X	X	X	X	X	X	X	X	X
12-lead ECG			X								X		X	

	Screening including run-in period		Double blind period											
**On-site Visit**	V1	V2[b]	V3	V4	V5	V6	V8	V10	V12/V14/V18/V22/V26	V16/V20/V24/V28	V30 [m]		V70[n]	V31
**Week (W)/ Month (M)** [a]	W-16 to W-4	W-2	M0 (D1)	M1	M2	M4	M8	M12	M16/M20/M30/M42/M54	M24/M36/M48/M60	M64 Final visit (common study end date)		Early end of treatment visit	M66 Follow-up visit (phone call)
											Study completers	Prematurely discontinued patients		
**Laboratory Testing/Efficacy:**														
TC, LDL-C, HDL-C, TG, non-HDL-C, ApoB	X	X	X	X	X	X		X	X	X	X		X	
ApoA-1, ratio ApoB/ApoA-1, and Lp (a)			X			X		X			X		X	
**Laboratory Testing/Safety:**														
Hematology and chemistry[g]	X		X					X		X	X		X	
Creatine phosphokinase (CPK)	X		X	X		X		X	X	X	X		X	
Liver panel[h]	X		X	X		X		X	X	X	X		X	
Hepatitis B surface antigen	X													
Hepatitis C antibody	X		X								X		X	
Serum pregnancy test[i]	X													
Urine pregnancy test[i]			X			X	X	X	X	X	X		X	
Urinalysis (dipstick and if abnormal, microscopy)	X		X					X		X	X		X	

	**Screening including run-in period**		**Double blind period**											
**On-site Visit**	V1	V2[b]	V3	V4	V5	V6	V8	V10	V12/V14/V18/V22/V26	V16/V20/V24/V28	V30 [m]		V70[n]	V31
**Week (W)/ Month (M)** [a]	W-16 to W-4	W-2	M0 (D1)	M1	M2	M4	M8	M12	M16/M20/M30/M42/M54	M24/M36/M48/M60	M64 Final visit (common study end date)		Early end of treatment visit	M66 Follow-up visit (phone call)
											Study completers	Prematurely discontinued patients		
**Laboratory Testing/Other:**														
HbA_{1c}	X		X					X		X	X		X	
High sensitivity C-reactive protein (hs-CRP)			X			X		X			X		X	
Anti-SAR236553 antibodies			X		X	X		X		X	X	X	X	
Library samples[j]			X			X		X			X		X	
**Genomics**														
Genomics consent (optional)			X											
Collect specimen (if genomics consent)[k]			X											
**Quality of Life Variables**														
EQ-5D patient questionnaire[L]			X		X	X	X	X	X	X	X		X	

[a] Window period for visits: at Week -2 is ± 3 days, at Months 1 and 2 is ± 7 days, and for all other visits it is ± 14 days.

[b] An optional visit (V2b) and central laboratory assessments may occur during the run-in period if modifications to background statin or other LMTs are made after an initial visit V2, to ensure that lipid criteria are nonetheless fulfilled prior to randomization.

[c] Prior medication: within 3 months prior to screening visit V1 and randomization (IMP administration), can be discontinued at any time prior to randomization or continued during the double-blind treatment period. Concomitant medication: received concomitantly to the IMP, from first IMP to the end of treatment + 70 days.

[d] Along with kit dispensation, the treatment administration package should be given as well as the IMP diary and injection instruction manual, as needed.

[e] Injection training during the run-in period is performed with placebo. Injection trainings should be done on site at V1 (Week-16 to Week-2) and V2 (Week-2) to make sure that patient is doing correctly self-injection, a third injection training can be performed at an additional visit such as the optional visit V2b.

[f] Injection is performed at randomization Visit Month 0/Day 1 on site with double-blind study treatment kit allocated by IVRS.

[g]. Hematology includes: complete blood cell count (CBC) including hematocrit, hemoglobin, red blood cell count, white blood cell count with differential count, and platelets. Chemistry includes: glucose, sodium, potassium, chloride, bicarbonate, calcium, phosphorous, urea nitrogen, creatinine, uric acid, LDH, total protein, albumin, and γGT.

[h] Liver panel: ALT, AST, alkaline phosphatase (ALP), and total bilirubin.

[i] Women of Child Bearing Potential (WOCBP) only.

[J] Library samples may be stored for up to 10 years for exploratory research that may include the study of biomarkers of PCSK9 action, lipoprotein metabolism and mechanisms of hyperlipidemia and cardiovascular disease (e.g. lipoprotein–associated phospholipase A2).

[k] If blood sample not collected at randomization, could be collected at any time during the study.

[L] EQ-5D patient questionnaire will only be administered in patients still on treatment, for prematurely discontinued patients the last administration will be done at the early end of treatment visit.

[m] Final visit can occur between 24 months and 64 months of follow-up when the last randomized patient reaches a 24-month follow-up or the target number of events (1613) is reached whichever comes last and will determine the common study end date. Final V30 (Month 64) will be completed for all patients regardless of the compliance with study treatment.

[n] Early end of treatment visit (V70) should be performed within 2 months for patients who prematurely discontinue the study treatment; assessments performed at this visit are similar to those to be performed for study completers at Final visit (V30). Prematurely discontinued patients will be maintained in the study until the common study end date or Final visit (V30).

1.2.2 Contacts (phone calls or contacts via internet) during the study

Month (M)[a]	M6/ M10/ M14/ M18/ M22/ M27/ M33/ M39/ M45/ M51/ M57/ M62
**Visit**	V7/ V9/ V11/ V13/ V15/ V17/ V19/ V21/ V23/ V25/ V27/ V29
**Phone Call**	☎ or Internet contact[b]
**Concomitant Medication**	X
**Collect information on IMP administration**	X
**Collect information on possible occurrence of efficacy endpoints**	X
**Reminders[c]**	X

[a] The month number is only indicative, flexibility is allowed in the timing of phone call in between the on-site visits.

[b] As permitted by privacy regulations governing individual sites.

[c] Reminders as applicable for IMP administration schedule, timing of next appointments, fasting conditions for next lab assessments, bring the diary, and used and unused kits at the next study site visit.

2 TABLE OF CONTENTS

CLINICAL TRIAL PROTOCOL **1**

1 FLOW CHARTS **12**

1.1 GRAPHICAL STUDY DESIGN 12

1.2 STUDY FLOW CHART 13

1.2.1 On-site visits during the study 13

1.2.2 Contacts (phone calls or contacts via internet) during the study 18

2 TABLE OF CONTENTS **19**

3 LIST OF ABBREVIATIONS **25**

4 INTRODUCTION AND RATIONALE **28**

5 STUDY OBJECTIVES **35**

5.1 PRIMARY 35

5.2 SECONDARY 35

6 STUDY DESIGN **36**

6.1 DESCRIPTION OF THE PROTOCOL 36

6.1.1 Run-in period 37

6.1.2 Double-blind treatment period 38

6.1.3 Follow-up contact 40

6.2 DURATION OF STUDY PARTICIPATION. 40

6.2.1 Duration of study participation for each patient 40

6.2.2 Determination of end of clinical trial (all patients) 40

6.3 INTERIM ANALYSIS 40

6.4 STUDY COMMITTEES 41

7 SELECTION OF PATIENTS **43**

7.1 INCLUSION CRITERIA 43

7.2 EXCLUSION CRITERIA 44

7.2.1 Exclusion criteria related to study methodology 44

7.2.2 Exclusion criteria related to the background therapy 46
7.2.3 Exclusion criteria related to the current knowledge of Sanofi compound 46

8 STUDY TREATMENTS 47

8.1 INVESTIGATIONAL MEDICINAL PRODUCT(S) 47
8.1.1 Route and method of administration 47
8.1.2 Timing of administration 47

8.2 NON-INVESTIGATIONAL MEDICINAL PRODUCT(S) 48

8.3 BLINDING PROCEDURES 48
8.3.1 Methods of blinding 48
8.3.2 Lipid parameters 49
8.3.3 Anti-SAR236553 antibodies 50
8.3.4 Committees 50
8.3.5 Data Analysis 51
8.3.6 Randomization code breaking during the study 51

8.4 METHOD OF ASSIGNING PATIENTS TO TREATMENT GROUP 51

8.5 PACKAGING AND LABELING 52

8.6 STORAGE CONDITIONS AND SHELF LIFE 53

8.7 RESPONSIBILITIES 53
8.7.1 Treatment accountability and compliance 53
8.7.2 Return and/or destruction of treatments 54

8.8 CONCOMITANT MEDICATION 54

9 ASSESSMENT OF INVESTIGATIONAL MEDICINAL PRODUCT 55

9.1 EFFICACY ENDPOINTS 55
9.1.1 Primary efficacy endpoint 55
9.1.2 Secondary efficacy endpoints 55
9.1.3 Main Secondary Efficacy Endpoint(s): 55
9.1.4 Other Secondary Efficacy Endpoint(s): 56
9.1.5 Efficacy assessment methods 56
9.1.6 Definitions of components of the composite primary efficacy endpoint 56
9.1.6.1 Coronary Heart Disease (CHD) Death 56
9.1.6.2 Non-fatal myocardial infarction (MI) 58
9.1.6.3 Fatal and Non-fatal stroke 58
9.1.6.4 Unstable angina requiring hospitalization 59
9.1.7 Definitions of the secondary efficacy endpoints 60

9.1.7.1 Death 60
9.1.7.2 Hospitalization for unanticipated coronary revascularization procedure. 63
9.1.7.3 Congestive heart failure requiring hospitalization. 63

9.2 SAFETY ENDPOINT(S): 65
9.2.1 Adverse event 65
9.2.2 Safety laboratory 65
9.2.3 Vital signs measurement 66
9.2.4 Electrocardiogram measurement 66

9.3 OTHER ENDPOINT(S): 66
9.3.1 Anti-SAR236553 antibody assessments 66
9.3.1.1 Sampling time 66
9.3.1.2 Sampling procedure 66
9.3.1.3 Bioanalytical method 66
9.3.2 Lipid parameters 67
9.3.2.1 Endpoints 67
9.3.2.2 Assessment method 67
9.3.3 hs-CRP 67
9.3.4 HbA_{1C} 67
9.3.5 EQ-5D Patient Questionnaire 68
9.3.6 Pharmacogenomic Samples 68

10 STUDY PROCEDURES 70

10.1 VISIT SCHEDULE 73
10.1.1 Screening visit - Visit 1 (Week-16 to Week-4) and entry in the run-in period 73
10.1.2 Qualifying visit - Visit 2 (Week-2, ± 3 days) 75
10.1.3 Optional qualifying visit - Visit 2b (Week-2, ± 3 days) 75
10.1.4 Double-blind treatment period 76
10.1.4.1 Study site visits from Visit 3 (Month 0, D1) to Visit 30 (Month 64) 76
10.1.4.2 Contacts (phone calls or contacts via internet) from randomization 83
10.1.4.3 Early end of treatment visit – Visit 70 84
10.1.4.4 Common study end date – Final Visit – Visit 30 (Month 64, ± 14 days) 84
10.1.4.5 Post-treatment Follow-up phone call 86

10.2 DEFINITION OF SOURCE DATA 86

10.3 HANDLING OF PATIENT TEMPORARY OR PERMANENT TREATMENT DISCONTINUATION AND OF PATIENT STUDY DISCONTINUATION 87
10.3.1 Temporary treatment discontinuation with investigational medicinal product(s) 87
10.3.2 Permanent treatment discontinuation with investigational medicinal product(s) 88
10.3.3 List of criteria for permanent treatment discontinuation 88
10.3.4 Handling of patients after permanent treatment discontinuation 89

10.3.5 Procedure and consequence for patient withdrawal from study 89

10.4 OBLIGATION OF THE INVESTIGATOR REGARDING SAFETY REPORTING 90

10.4.1 Definitions of adverse events 90
10.4.1.1 Adverse event 90
10.4.1.2 Serious adverse event 90

10.4.2 Adverse event of special interest 91

10.4.3 Serious adverse events waived from expedited regulatory reporting to health authorities 91

10.4.4 General guidelines for reporting adverse events 93

10.4.5 Instructions for reporting serious adverse events 94

10.4.6 Guidelines for reporting adverse events of special interest 94
10.4.6.1 Reporting of adverse events of special interest with immediate notification 94
10.4.6.2 Reporting of adverse events of special interest without immediate notification 95

10.4.7 Guidelines for management of specific laboratory abnormalities 96
10.4.7.1 Hemoglobin decrease 96

10.5 OBLIGATIONS OF THE SPONSOR 97

10.6 SAFETY INSTRUCTIONS 97

10.6.1 Local tolerability (Local Injection Site Reactions) 97

10.6.2 Allergic adverse events (See Sections 10.4.6.1 and 10.4.6.2) 97
10.6.2.1 Allergic adverse event with cutaneous involvement 98
10.6.2.2 Acute allergic injection reactions (Section 10.4.6.2) 98

10.6.3 Monitoring related to two consecutive LDL-C <25 mg/dL (0.65 mmol/L) 99

10.7 ADVERSE EVENTS MONITORING 100

11 STATISTICAL CONSIDERATIONS 101

11.1 DETERMINATION OF SAMPLE SIZE 101

11.2 DISPOSITION OF PATIENTS 102

11.3 ANALYSIS POPULATIONS 102

11.3.1 Efficacy populations 102
11.3.1.1 Intent–to-treat population 102

11.3.2 Safety population 102

11.3.3 Other analysis populations 103

11.4 STATISTICAL METHODS 103

11.4.1 Extent of study treatment exposure and compliance 103
11.4.1.1 Extent of investigational medicinal product exposure 103
11.4.1.2 Compliance 103

11.4.2 Analyses of efficacy endpoints 104
11.4.2.1 Analysis of primary efficacy endpoint(s) 104

11.4.2.2 Analyses of secondary efficacy endpoints 106
11.4.2.3 Multiplicity consideration 106
11.4.3 Analyses of safety data 106
11.4.3.1 Adverse events 107
11.4.3.2 Laboratory data and vital signs 108
11.4.4 Other endpoints: 109
11.5 INTERIM ANALYSIS 109
12 ETHICAL AND REGULATORY STANDARDS 111
12.1 ETHICAL PRINCIPLES 111
12.2 LAWS AND REGULATIONS 111
12.3 INFORMED CONSENT 111
12.4 INSTITUTIONAL REVIEW BOARD/INDEPENDENT ETHICS COMMITTEE (IRB/IEC) 112
13 STUDY MONITORING 113
13.1 RESPONSIBILITIES OF THE INVESTIGATOR(S) 113
13.2 RESPONSIBILITIES OF THE SPONSOR 113
13.3 SOURCE DOCUMENT REQUIREMENTS 114
13.4 USE AND COMPLETION OF CASE REPORT FORMS (E-CRFS) AND ADDITIONAL REQUEST 114
13.5 USE OF COMPUTERIZED SYSTEMS 114
14 ADMINISTRATIVE EXPECTATIONS 115
14.1 CURRICULUM VITAE 115
14.2 RECORD RETENTION IN STUDY SITES 115
14.3 CONFIDENTIALITY 115
14.4 PROPERTY RIGHTS 116
14.5 DATA PROTECTION 116
14.6 INSURANCE COMPENSATION 116
14.7 SPONSOR AUDITS AND INSPECTIONS BY REGULATORY AGENCIES 117
14.8 PREMATURE DISCONTINUATION OF THE STUDY OR PREMATURE CLOSE-OUT OF A SITE 117
14.8.1 Decided by the Sponsor 117
14.8.2 Decided by the Investigator 118

14.9 CLINICAL TRIAL RESULTS 118

14.10 PUBLICATIONS AND COMMUNICATIONS 118

15 CLINICAL TRIAL PROTOCOL AMENDMENTS 120

16 BIBLIOGRAPHIC REFERENCES 121

17 APPENDICES 125

APPENDIX A MONITORING PLAN FOR LDL-C < 25 MG/DL (0.65 MMOL/L) 126

APPENDIX B DNA STORAGE SAMPLES 128

APPENDIX C GENERAL GUIDANCE FOR THE FOLLOW-UP OF LABORATORY ABNORMALITIES BY SANOFI 131

APPENDIX D GUIDELINES FOR HEMOGLOBIN (HB) DECREASE ≥ 1.5 G/DL 137

APPENDIX E SUMMARY OF ADVERSE EVENT REPORTING INSTRUCTIONS 138

APPENDIX F ASSESSMENT OF LOCAL INJECTION SITE REACTIONS 140

APPENDIX G EQ-5D PATIENT QUESTIONNAIRE 141

3 LIST OF ABBREVIATIONS

ACS	Acute coronary syndrome
ADA	Anti drug antibody
AE	Adverse event
AESI	Adverse event of special interest
ALP	Alkaline phosphatase
ALT	Alanine aminotransferase
ANCOVA	Analysis of covariance
Apo	Apolipoprotein
ARF	Acute renal failure
AST	Aspartate aminotransferase
BMI	Body mass index
BP	Blood pressure
CABG	Coronary artery bypass graft surgery
CBC	Complete blood count
CEC	Clinical Events Committee
CHD	Coronary Heart Disease
CI	Confidence interval
CIB	Clinical Investigator's brochure
CPK	Creatine Phosphokinase
CRO	Contract Research Organization
CSR	Clinical Study Report
CT	Computed tomography
CV	Cardiovascular
DBTP	Double Blind Treatment Period
DM	Diabetes mellitus
DMC	Data Monitoring Committee
DNA	Deoxyribonucleic acid
DRF	Discrepancy resolution form
ECG	Electrocardiogram
e.g.	Exempli gratia = for example
e-SMS	Emergency Scientific & Medical Services
e-CRF	Electronic case report form
EDTA	Ethylene diamine tetra-acetic acid
ELISA	Enzyme linked immuno-sorbent assay
eGFR	Estimated Glomerular Filtration Rate
FH	Familial hypercholesterolemia
FPI	First patient in
FU	Follow-up
GCP	Good Clinical Practice
γGT	Gamma-glutamyl Transferase

HbA_{1c}	Glycated haemoglobin A_{1c}
HDL-C	High-density lipoprotein cholesterol
HeFH	Heterozygous familial hypercholesterolemia
HLGT	High Level Group Term
HLT	High level term
HR	Heart rate
hs-CRP	High-sensitivity C-reactive protein
IA	Interim Analysis
ICF	Informed consent form
ICH	International Conference on Harmonization
i.e.	Id est = that is
IEC	Independent ethics committee
INN	International Nonproprietary Name
IMP	Investigational Medicinal Product
IRB	Institutional review board
ITT	Intent-to-treat
IV	Intravenous
IVRS	Interactive Voice Response System
IWRS	Interactive Web Response System
Kg	Kilogram
LDH	Lactate dehydrogenase
LDL-C	Low-density lipoprotein cholesterol
LDL-R	Low-density lipoprotein receptor
LLN	Lower limit of normal range
LMT	Lipid modifying therapy
LPI	Last patient in
LOCF	Last-observation-carried-forward
Lp(a)	Lipoprotein a
MDRD	Modification of Diet in Renal Disease
MedDRA	Medical Dictionary for Regulatory Activities
µg	Microgram
MI	Myocardial infarction
mITT	Modified intent-to-treat
mmHg	Millimeter of mercury
NCEPATPIII	National Cholesterol Education Program Adult Treatment Panel III
NYHA	New York heart association
NIMP	Non Investigational Medicinal Product
PAD	Peripheral Arterial Disease
PCI	Percutaneous coronary intervention
PCSA	Potentially Clinically Significant Abnormality
PCSK9	Proprotein convertase subtilisin/kexin type 9
PD	Pharmacodynamics
PDM	Project Demand Manager
PK	Pharmacokinetics
PT	Preferred term

PTC	Product technical complaint
Q2W	every 2 weeks
SAE	Serious Adverse Event
SAP	Statistical analysis plan
SC	Subcutaneous
SD	Standard deviation
SNP	Single nucleotide polymorphisms
SOC	System-organ-class
SUSAR	Suspected Unexpected Serious Adverse Reaction
TOTAL-C	Total cholesterol
TEAE	Treatment Emergent Adverse Event
TG	Triglycerides
TIA	Transient ischemic attack
TLC	Therapeutic lifestyle changes
ULN	Upper limit of normal range
WBC	White blood cell
WHO-DD	World Health Organization-Drug Dictionary
WOCBP	Women of childbearing potential

4 INTRODUCTION AND RATIONALE

SAR236553 is a fully human monoclonal antibody that binds PCSK9. All relevant information concerning the compound is available in the latest version of the Clinical Investigator's Brochure (CIB) (1).

SAR236553 is also referred to as REGN727. However, in the context of the EFC11570 study protocol, it will be referred to as SAR236553.

Patients with recent acute coronary syndrome (ACS) are at very high risk for suffering recurrent coronary events in the near term. In approximately 10% of patients with ACS, cardiovascular death, recurrent myocardial infarction, or stroke occur within 1 year (2). Based on the results of large clinical trials, early intensive statin therapy has become formally endorsed as a treatment recommendation (3) (4) for patients with ACS (5). The use of high-dose statins has been largely demonstrated to be safe and well tolerated (6).

Both epidemiological and pharmacological intervention trials have demonstrated a strong and linear relationship between the levels of low-density lipoprotein cholesterol (LDL-C) and cardiovascular (CV) events. Three of the most recent statin trials, the TNT trial (7), the PROVE-IT trial (8), and the JUPITER trial (9), have provided new information on the relationship between low levels of LDL-C and CV event rates, with demonstration that treatment of LDL-C to a mean level of 77 mg/dL , 62 mg/dL, or 55 mg/dL was associated with a greater reduction in CV events.

The overall results of these trials have helped to demonstrate the continued linear relationship between LDL-C and CV events to these levels and to support the establishment of 70 mg/dL as a treatment goal for high-risk patients (10) (11). The lack of a demonstrated existing threshold or plateau between LDL-C and CV risk from these studies begs the question that even further reductions beyond the 55-77 mg/dL observed in these trials could provide additional benefits in CV event reduction. In the above three trials post-hoc analyses were conducted analyzing both the efficacy (in regards to CV event rates) and safety of achieving LDL-C levels at the lower end of the treatment spectrum.

- The PROVE-IT/TIMI-22 trial examined patients with LDL-C <100 mg/dL in the atorvastatin arm and found a lower rate of CV events in the patients with achieved LDL-C levels in the <40 mg/dL and the 40-60 mg/dL groups than those patients with higher achieved LDL-C levels (12).
- In the TNT trial, patients in the lowest quartile of achieved LDL-C had value < 64 mg/dL and a mean LDL-C level of 54 mg/dL as compared to means of 70, 83, 97 and 122 mg/dL in the remaining quintiles. Within these quintiles, there was a strong and significant relationship between the lower achieved LDL-C levels and lower rates of major CV events (p<0.0001) (13).
- In the JUPITER trial, the investigators split the patients in the rosuvastatin group into two cohorts of achieved LDL-C <50 mg/dL (n=4154) and >50 mg/dL (n=4000) and compared

them to the placebo group. Those that had achieved LDL-C < 50 mg/dL demonstrated significantly lower rates of CV events than either of the other two groups (14).

In none of these three analyses there was evidence of an adverse safety signal observed with patients that achieved these lower levels of LDL-C. A recent communication compared patients who achieved a LDL-C ≤ 30 mg/dL (n=621) versus those with LDL-C >30 mg/dL (n=7533) (15). No differences in overall TEAEs and many other specific AEs were observed, with the exception of a greater incidence in insomnia (1.6/100 PY vs. 1.2/100 PY; p=0.031) and hematuria (1.8 /100 PY vs. 1.1/100 PY; p=0.0007) among patients with LDL-C ≤ 30 mg/dL. The rate of clinically relevant declines in eGFR (≥ 30%) tended to be lower among patients with lower reduction in LDL-C.

Similar findings have been observed in a patient-level meta-analysis conducted by the Choleterol Trialists Treatment Collaboration. This examination of 26 large statin CV event trials encompassing approximately 170,000 patients has demonstrated that 20% reduction in major CV events can be derived for every 1 mmol/L (38.6 mg/dl) reduction in LDL-C, even in patients whose starting LDL-C levels are less than 2 mmol/L (77 mg/dl) (16). This meta-analysis has also demonstrated no significant safety risks with long-term cholesterol reduction treatment, including cancer (17).

These data provide strong support for the concept that high risk patients may derive further benefits of reductions in LDL-C to levels <50 mg/dl (1.29 mmol/L). A number of lipid and cardiovascular experts have begun to consider lower LDL-C values as the ideal level for humans with the concept that LDL-C levels of 30-50 mg/dL (0.78-1.29 mmol/L) represent the physiological ideal. This is based upon the findings from both the observed LDL-C levels in human newborns as well as in humans with more primitive/paleolithic lifestyles (18) (19) (20).

However, many high CV risk patients cannot achieve such levels with currently available lipid-lowering drugs. Furthermore, a significant number of high-risk patients even fail to achieve their recommended LDL-C target levels (21) (22) and most CV events are actually not prevented, leaving a substantial “residual risk” for patients and thus additional pharmacologic therapies for the prevention of coronary heart disease (CHD) remains essential, particularly for high-risk patients with ACS.

Introduction to proprotein convertase subtilisin kexin type 9 (PCSK9):

Proprotein convertase subtilisin kexin type 9 (PCSK9) belongs to the subtilisin family of serine proteases and is highly expressed in the liver. PCSK9 is involved in regulating the levels of the low-density lipoprotein receptor (LDL-R) protein (23) (24). Once PCSK9 is secreted into plasma it directly binds to the LDL-R and promotes its degradation. The increased degradation of LDLRs leads to a reduced LDL-C removal, and therefore higher LDL-C circulating levels. Experiments with mice have shown that increasing PCSK9 protein levels decreases levels of LDL-R protein in the liver while PCSK9 knockout mice have increased levels of LDL-R in the liver (25) (26). In humans, PCSK9 mutations have been identified: the gain-of-function mutations are rare and cause an autosomal dominant form of severe hypercholesterolemia and premature CHD, whereas loss-of-function mutations are more common and are associated with reduced plasma levels of LDL-C and protection from CHD (27) (28).

Therefore blocking PCSK9 binding to the LDL-R can potentially benefit patients with hypercholesterolemia by decreasing their plasma LDL-C levels. In addition, PCSK9 messenger ribonucleic acid (mRNA) and protein levels are increased in response to statins, potentially attenuating their cholesterol-lowering effect (29).

Summary of selected clinical studies with SAR236553:

Phase 1 studies

Three Phase 1 studies have been conducted with SAR236553 and evaluated the safety, tolerability and pharmacokinetics/pharmacodynamics (PK/PD) profile. Two studies were single dose administration (R727-CL-0902 study with intravenous (IV) administration of doses from 0.3 to 12 mg/kg and R727-CL-0904 study with SC administration of doses from 50 to 250 mg) conducted in healthy volunteers with LDL-C >100 mg/dL for whom statin therapy was not indicated. The third study (R727-CL-1001 study) was conducted in hypercholesterolemic patients (familial or non-familial) with single to multiple subcutaneous (SC) administration of 50 mg, 100 mg, 150 mg and 200 mg either as add-on to stable doses of atorvastatin from 10 to 40 mg/day or as monotherapy.

Results of these Phase 1 studies showed that SAR236553 administered to healthy volunteers and patients either by IV or SC administration was generally well tolerated at all doses; treatment emergent adverse events (TEAEs) did not display a dose relationship. No pattern of adverse events related to the drug was identified. In all these Phase 1 studies, administration of SAR236553 induced rapid, substantial, and sustained reductions from baseline in LDL-C, up to 60%. The magnitude and duration of these reductions were positively related to the dose administered. It should also be noted that in the R727-CL-1001 study, results were similar in the familial and non familial hypercholesterolemic patients.

Overall, a total of 109 subjects were exposed to at least one dose of SAR236553 in these three Phases 1 studies.

Phase 2 studies:

Three Phase 2 studies have been conducted:

- Two dose / dose regimen finding studies (DFI11565 and R727-CL-1003) with the main objective to assess, over a 12 week-treatment duration, the effects on LDL-C level reduction of several doses of SAR236553 and 2 dose-regimens (50 mg, 100 mg and 150 mg every 2 weeks (Q2W), and 200 mg and 300 mg every 4 weeks (Q4W) for the DFI11565 study and 150 mg Q2W, 150 mg, 200 mg, and 300 mg Q4W for the R727-CL-1003 study). DFI11565 was conducted in hypercholesterolemic patients with elevated LDL-C (≥100 mg/dL or 2.59 mmol/L) despite stable atorvastatin therapy. R727-CL-1003 study was conducted in patients with heterozygous familial hypercholesterolemia (heFH) and with elevated LDLC (≥100 mg/dL or 2.59 mmol/L) despite their current lipid lowering therapy (statin ± ezetimibe).
- The third Phase 2 study (DFI11566) aimed to evaluate in patients with hypercholesterolemia the efficacy and safety of the co-administration of SAR236553 150 mg every 2 weeks and a high daily dose of atorvastatin (80 mg) in comparison to the co-administration of placebo and this high daily dose of atorvastatin (80 mg), in patients previously receiving a stable dose of

atorvastatin 10 mg. This treatment scheme is anticipated to be used when a rapid decrease in LDLC level is needed, e.g. after an acute coronary syndrome.

Overall, a total of 274 patients were exposed to at least one dose of SAR236553 in these three Phases 2 studies.

Efficacy results:

In both dose finding studies, statistically significant decreases in percent change from baseline in LDL-C at 12 weeks were observed in all SAR236553 groups compared to the placebo group. In the DFI11565 study for the Q2W dose regimen, the greatest decrease was seen in the 150 mg Q2W group (-72.4%) compared with a small decrease in the placebo group (-5.1%) (LS mean difference versus placebo of -67.3%; p<0.0001). The decreases observed with the doses administered Q2W were maintained from the first injection throughout the study and more particularly throughout the interval period between the injections. A similar pattern with the dose of 150 mg Q2W was seen in the R727-CL-1003 study with a significant decrease from baseline of -67.9% versus -10.7% in the placebo group (LS mean difference versus. placebo of -57.3%; p<0.0001). Large decreases in LDL-C from baseline to 12 weeks were also observed with doses administered Q4W; however, the treatment effect was not fully maintained over a 4-week period (i.e., the time interval between the two injections).

The same magnitude of effect was shown for the dose of 150 mg Q2W in the DFI11566 study, with a statistically significant decrease in LDL-C at 8 weeks in the SAR236553 150 mg + atorvastatin 80 mg group (median reduction of – 70.6 %) compared with the placebo + atorvastatin 80 mg group (median reduction of – 26.9 %).

In all three studies, consistent results were seen for total-cholesterol (TC), ApoB, non-HDL-C and ApoB/ApoA-1 ratio. A favorable trend was also observed for high-density lipoprotein cholesterol (HDL-C), apolipoprotein A-1 (ApoA-1), triglycerides (TG) and lipoprotein a (Lp(a)).

Safety results

SAR236553 was well tolerated in all completed Phase 2 studies throughout the treatment period and for all treatment groups. Injection sites reactions were reported in patients including placebo-treated patients; the reporting of these events was greatest in the R727-CL-1003 study (40.3% in SAR236553-treated patients vs 12.6% and 3.3% in DFI11565 and DFI 11566, respectively); however these events were generally transient with no dose relationship. Rare cases of hypersensitivity reactions were reported. Among all SAEs reported for all SAR236553 studies, only one case, leucocytoclastic vasculitis (angiitis), was reported as being related to SAR236553 (DFI11565 study) . The patient developed one episode of diarrhea followed on the same evening by rash in arms, legs and abdomen 9 days after the first administration of SAR236553 300 mg Q4W. The diagnosis was confirmed by skin biopsy. The patient was discontinued from study drug but completed the study. The patient fully recovered from the rash after a course of tapering steroid administration.. A positive ADA status was reported with a low titer of 30 (corresponding to the minimum titer detected by the assay), only observed at the Week 20 assessment (ie, between 2.5 and 3 months after the event) with a negative retest after 6 months. Additional tests also obtained 6 months after the event were unremarkable and included normal

immunoglobulin levels, a negative antinuclear antibody test, and only a mild elevation of CRP. No particular signal was noted for TEAEs related to musculoskeletal or connective tissue disorders as well as there were no LFT elevations. Given the limited published data on the safety of LDL-C level < 25 mg/dL, a prespecified statistical analysis was conducted in patients reaching LDL-C value < 25mg/dL in all the phase 2 studies with no specific safety signal identified over the study duration. In the two dose finding studies (DFI11565 and R727-CL-1003), the proportion of patients reaching an LDL-C < 25 mg/dL in the 150 mg Q2W group was from 31.3% to 44.8%. In DFI11566, with atorvastatin 80mg the proportion in the 150 mg Q2W group was approximately 50%.

For detailed information, please refer to the CIB (1).

Dose and regimen selection

Based on the results of the above studies, the Q2W dosing regimen was selected as the most appropriate to maintain constant LDL-C lowering throughout the interdosing interval. Since the magnitude of effect observed with 150 mg Q2W may not be needed to achieve the LDL-C goal in all patients, a lower dose of 75 mg was selected as a starting dose. This selection is also based on the LDL-C reduction needed to provide the best benefit in terms of CVD reduction, and potential safety considerations regarding low LDL-C values.

The current and most relevant evidence around the effects of achieved low LDL-C levels comes from examinations of large statin trials (12) (13) (14) as presented above, and patients with PCSK9 loss-of-function mutations (30). The patients achieving the lower levels of LDL-C had the lower CV event rates. To date, there is no evidence that very low LDL-C levels result in significant adverse health effects based on these sources of information, though this conclusion is based on a relatively low number of patients with very low LDL-C who have been studied.

Rationale for protocol design:

The objective of the present study is to evaluate the ability of SAR236533 to reduce CV events in patients who recently experienced an ACS event and despite intensive statin therapy or at maximally tolerated dose don't reach the goal as defined in the guidelines for these very high risk patients.

For this randomized, double-blind, placebo-controlled study it is estimated that approximately 18,000 patients will be enrolled with a minimum follow-up of approximately 24 months and a total duration of approximately 5 years. Randomization will occur within 16 weeks of the index ACS event and patients will enter a run-in period during which they will have to be stabilized on intensive statin therapy defined as atorvastatin 40 or 80 mg or rosuvastatin 20 or 40 mg or maximally tolerated dose of either of these 2 agents in case of tolerability issues. Only patients not reaching goal, i.e. LDL-C ≥ 70 mg/d L (≥ 1.81 mmol/L) or ApoB ≥ 80 mg/dL (≥ 0.8 mmol/L) or non HDL-C ≥ 100 mg/dL (≥ 2.59 mmol/L), will be randomized to either background therapy + SAR2365553 or background therapy + placebo. The choice to select eligible patients on two other lipid parameters, in addition to the traditional LDL-C target lipid parameter, is based on the acceptance that ApoB and non HDL-C can be considered to be equal to LDL-C in risk prediction

(10) (11). Some adjustment in the lipid-modifying background therapy can occur during the run-in period, e.g. in case of poor tolerance to intensive statin therapy.

The proposed primary efficacy endpoint is the effect of SAR236553 compared to placebo on top of best evidence background therapy on the occurrence of the following composite endpoint: coronary heart disease (CHD) death, non-fatal myocardial infarction, non-fatal and fatal ischaemic stroke, and unstable angina requiring hospitalization with stringent criteria for the definition of this latter endpoint. Based on other contemporary studies (31) (32), the primary endpoint event rate in the placebo group is assumed to be 11.4% at 48 months (3.8% at 12 months, 6.4% at 24 months, 9.0% at 36 months).

In this trial, all patients will be initially treated with 75 mg Q2W, and only those patients whose LDL-C levels remain equal or higher than 50 mg/dL (1.29 mmol/L) after 1 month of treatment will be up-titrated to 150 mg Q2W (at Month 2).

With this treatment scheme, most patients can be expected to achieve their target LDL-C, as recommended by international guidelines committees (10) (11). Furthermore, the up-titration threshold set at 50 mg/dL (1.29 mmol/L), supported by findings from post-hoc analyses of the PROVE-IT, TNT and JUPITER trials, will promote the achievement of an LDL-C level within the ‘physiologic ideal’ zone.

Available data do not point to a lower limit of safe and effective cholesterol lowering (12) (13) (14). However, this conclusion is based on a relatively low number of patients with very low LDL-C who have been studied. In this trial, with the use of 75 mg as starting dose and a target-based up-titration scheme, it is anticipated that few patients will reach a LDL-C level below 25 mg/dL (0.65 mmol/L). For patients uptitrated to 150 mg Q2W who reach a LDL-C level below 25 mg/dL (0.65 mmol/L), a down-titration from 150 mg to 75 mg Q2W will be performed. For patients on 75 mg Q2W, two different rules will be applied depending on the level of LDL-C. In case of an LDL-C level below 25 mg/dL (0.65 mmol/L) but greater than or equal to 15 mg/dL (0.39 mmol/L), additional monitoring will be implemented to further confirm the safety of low LDL-C levels. Due to lack of available information at a LDL-C level below 15 mg/dL (0.39 mmol/L) (except in subjects with rare genetic mutations), patients reaching such low levels on 2 consecutive occasions will have their treatment discontinued.

Addition to SAR236553 in patients not yet at goal should derive to further benefits in terms of reductions in LDL-C. However a greater treatment effect in patients with higher LDL-C levels at baseline cannot be ruled out. For this reason, the distribution of LDL-C levels at baseline will be monitored to ensure that the initial assumption of a mean baseline LDL-C of 90-100 mg:dL (2.33-2.59 mmol/L) is fulfilled. In case it is observed that the distribution is shifted to lower baseline levels of LDL-C, a capping of the number of patients with a baseline LDL-C between 70-80 mg/dL may be considered.

Preliminary PK data from Phase 2 studies DFI11565, DFI11566 and R727-CL-1003, showed that exposure to SAR235663 declined during the 8-week follow-up period that followed the double-blind treatment period, with serum total concentrations of SAR236553 still detectable, but at very low levels. Therefore to ensure sufficient low, non-effective SAR236553 serum concentrations,

patients, still on study treatment at the time of the common study end date, will continue to be followed during a follow-up period of 8 weeks (i.e., 2 months; 10 weeks after last dosing).

Conclusion on the benefit risk assessment with SAR236553

Based on the clinical data available to date, treatment with SAR236553 has demonstrated a significant LDL-C lowering effect and was generally well tolerated in a population of patients with non familial hypercholesterolemia or with heterozygous familial hypercholesterolemia. The efficacy on LDL-C was associated with consistent results in total cholesterol, ApoB, non-HDL-C and ApoB/ApoA-1 ratio and a positive trend for HDL-C, TG and Lp (a). There was no evidence that SAR236553 adversely affects other cardiovascular risk factors, e.g., body weight, blood pressure, glucose, or CRP.

In terms of identified or potential risks with SAR236553, local injection site reactions were reported as well as rare cases of hypersensitivity reactions. Local injection site reactions were reported in both SAR236553 and placebo treatment groups with no evidence of dose relatioship. These AEs will be monitored in the Phase 3 program including this study. A substantial proportion of patients reached low LDL-C levels (< 25 mg/dl [0.65 mmol/L]) with no safety signal identified to date. However, further monitoring for potential AEs associated with low LDL-C levels will be implemented. Although no particular signal related to CPK elevation and associated AEs (e.g., myalgia, rhabdomyolysis) was detected with the co-administration of SAR236553 and statins over a maximum duration of 12 weeks, monitoring for such adverse events will continue for all the Phase 3 studies, including this study. An independent Data Monitoring Committee, dedicated to the EFC11570 study and identified as CV DMC, will meet periodically to review unblinded safety data. This CV DMC will have a close connexion with the other independent DMC implemented for all Phase 3 studies evaluating the efficacy and safety of SAR236553 on LDL-C (identified as Phase 3a DMC).

This CV outcome study is undertaken to demonstrate in patients who recently experienced an ACS event and who are not at their LDL-C goal despite an intensive lipid-lowering therapy that SAR236553 75mg Q2W or 75 mg Q2W / 150 mg Q2W provides an additional benefit with the reduction of CV events.

5 STUDY OBJECTIVES

5.1 PRIMARY

The primary objective of this study is to compare the effect of SAR236553 with placebo on the occurrence of cardiovascular events (composite endpoint of coronary heart disease (CHD) death, non-fatal myocardial infarction (MI), fatal and non-fatal ischemic stroke, unstable angina requiring hospitalization) in patients who have experienced an acute coronary syndrome (ACS) event 4 to 16 weeks prior to randomization and are treated with intensive statin therapy (defined as atorvastatin 40 or 80 mg, or rosuvastatin 20 or 40 mg) or at maximally tolerated dose of these given statins, or other non statin LMT(s).

5.2 SECONDARY

The secondary objectives are:

To compare the efficacy of SAR236553 versus placebo on secondary endpoints (major CHD event, any CHD event, any CV event, composite of all cause mortality/non-fatal MI/non-fatal ischemic stroke, all cause mortality).

A Clinical Events Committee (CEC) will be established to adjudicate, in a blinded fashion, events corresponding to the primary and secondary cardiovascular endpoints as well as all causes of death.

- To evaluate the safety and tolerability of SAR236553 throughout the study.
- To evaluate the development of anti-SAR236553 antibodies.
- To evaluate the effect of SAR236553 on low-density lipoprotein cholesterol (LDL-C), apolipoprotein B (ApoB), and non-high-density lipoprotein cholesterol (non-HDL-C).

6 STUDY DESIGN

This is a double-blind, randomized, placebo-controlled, balanced (1:1, SAR236553: placebo), parallel-group, multi-national, multicenter study. Randomization will take place 4 weeks to 16 weeks after the index event and will be stratified according to country. Prior to this randomization, eligible patients will enter a run-in period of at least 4 weeks, during which they will receive intensive statin therapy defined as atorvastatin 40 or 80 mg or rosuvastatin 20 or 40 mg; in case where patients are unable to tolerate atorvastatin 40/80 mg or rosuvastatin 20/40 mg, they will be allowed to receive the highest tolerated dose of atorvastatin or rosuvastatin, or under some documented circumstances other lipid lowering treatment than a statin. Following this run-in period, only patients not reaching goal on the current LMT, i.e. LDL-C ≥ 70 mg/dL (≥ 1.81 mmol/L) or ApoB ≥ 80 mg/dL (≥ 0.8 mmol/L) or non-HDL-C ≥ 100 mg/dL (≥ 2.59 mmol/L), will be randomized to either background therapy + SAR2365553 or background therapy + placebo. All patients randomized to SAR236553 will initially receive SAR236553 75 mg Q2W. Patients on SAR236553 not reaching the target LDL-C level at Month 1 will have their dose up-titrated to 150 mg Q2W at Month 2 in a blinded fashion. The double-blind treatment period will continue until each surviving randomized patient has been followed for a minimum of 24 months or the target number of events (1613) is reached, whichever comes last. The corresponding estimated study duration is 64 months. All patients, even if they have achieved an endpoint or those who have prematurely discontinued the study treatment, will be asked to remain in the study until common study end date. A follow-up will only be applicable for patients still on study treatment at the time of the common study end date, and should be scheduled 8 weeks (i.e., 2 months) after the common study end date.

6.1 DESCRIPTION OF THE PROTOCOL

The study will comprise 3 periods:

- A run-in period during which the background lipid-modifying therapy (LMT) will be adjusted as needed, to ascertain the patient is receiving the required intensive statin treatment (defined as atorvastatin 40 or 80 mg, or rosuvastatin 20 or 40 mg) or the maximally tolerated doses of these given statins, or other non statin LMTs prior to randomization, and to stabilize this treatment.
- A double-blind treatment period during which the background LMT will not be modified (except for safety reasons).
- A follow-up contact 8 weeks (i.e., 2 months) after the final visit (Visit 30, Month 64) that corresponds to the common study end date. This follow-up contact only applies for patients still on study treatment at the time of the common study end date.

Patients should be on a stable diet (NCEP-ATPIII TLC diet or equivalent) throughout the entire study duration from screening to the end of study.

6.1.1 Run-in period

The run-in period will start by a screening visit during which patients will be evaluated for eligibility to enter this run-in period. This screening visit can occur as soon as the day of the index ACS event, but no later than 12 weeks (+ 3 days) after the index event. Then a qualifying Visit (V2, Week-2) will be performed within 2 weeks (± 3 days) prior to randomization to obtain the results of the qualifying lipid parameters.

Note: An optional visit (V2b) and central laboratory assessments may occur during the run-in period if modifications to background statin or other LMTs are made after the initial qualifying visit (V2), to ascertain that lipid criteria are fulfilled after these modifications and within 2 weeks prior to randomization.

During this run-in period the following rules will be applied:

- All patients will receive atorvastatin or rosuvastatin as follows:
  - Following the index ACS event, intensive statin therapy as defined by atorvastatin 40 or 80 mg, or rosuvastatin 20 or 40 mg daily will be given to statin naïve patients as well as to patients previously treated with other statins or lower doses of atorvastatin or rosuvastatin.
  - However:

  a) An initial dose of atorvastatin lower than 40/ 80 mg or rosuvastatin 20/40mg may be chosen for patients who are known to be intolerant to these doses. The maximum tolerated dose of atorvastatin or rosuvastatin will be determined by the investigator.

  b) An initial dose of atorvastatin lower than 40/ 80 mg or rosuvastatin 20/40mg may be chosen for patients in whom the investigator considers such dose prudent due to advanced age, low body mass index or other concerns. If the initial dose is tolerated, the dose may afterwards be increased up to atorvastatin 40 or 80 mg or rosuvastatin 20 or 40 mg, at the investigator's discretion but no later than 12 weeks after the index ACS event.

  c) Atorvastatin or rosuvastatin dose may be decreased from the initial dose in patients who develop adverse events (e.g, myalgia), in accordance with usual guidance.

  d) Any change in the dose of atorvastatin or rosuvastatin must have occurred at least 2 weeks prior to the qualifying Visit (V2, Week-2).

  e) Patients who do not tolerate the minimum approved dose of atorvastatin (10 mg) or rosuvastatin (5 mg) may nonetheless qualify for the trial without any ongoing atorvastatin/rosuvastatin therapy, provided that they are compliant with all other inclusion and exclusion criteria and they are on stable treatment with other non statin LMTs for at least 4 weeks prior to the qualifying Visit (V2, Week-2).

- LMTs other than statin can be maintained during the run-in period, as long as qualifying labs are obtained (V2, Week-2) a minimum of 4 weeks after any change in dose of non-statin LMT; for fibrates, only fenofibrate is allowed.

- Ezetimibe (but not other LMTs) can be initiated during the run-in period as long as qualifying labs are obtained (V2, Week-2) a minimum of 4 weeks after any change in dose of ezetimibe.

In summary, the run-in period ends with a randomization visit (V3) that occurs 4 weeks to 16 weeks (+ 3 days) after the index ACS event and no later than 2 weeks (± 3 days) after qualifying Visit V2:

- Qualifying (central) lipid parameters are obtained at the qualifying Visit (V2) when all of the following conditions have been met: at least 2 weeks have elapsed after index ACS event, at least 2 weeks have elapsed after any change in atorvastatin or rosuvastatin dose, and at least 4 weeks have elapsed after any change in non-statin LMT.
- Randomization (at Visit V3) may take place as soon as it is verified that qualifying V2 labs fulfill laboratory inclusion criteria, (and no exclusion criteria are met), and should occur no later than 2 weeks (± 3 days) after visit V2 and no later than16 weeks (+ 3 days) after index ACS event.

During the run-in period, patients (or their caregivers) will administer at least 2 subcutaneous injections of the placebo SAR236553 in order to train them to administer the self-injection and to ensure patient's acceptance of an injectable study treatment. These training injections should be performed on site at screening Visit (V1, Week-16 to Week-4) and qualifying Visit (V2, Week-2), respectively. A third training injection can be performed at an additional visit such as the optional visit V2b.

6.1.2 Double-blind treatment period

The double-blind treatment period will continue until each surviving randomized patient has been followed for a minimum of 24 months or the target number of events (1613) is reached, whichever comes last. The corresponding estimated study duration is 64 months (as described in the sample size considerations – Section 11.1).

During this double-blind treatment period, the dosing of SAR236553 is intended to achieve LDL-C levels of 30-50 mg/dL (0.78-1.29 mmol/L) which is considered as the physiologic ideal level and to avoid levels of LDL-C that are clearly below the physiologic range (i.e.,<15 mg/dL). To achieve this goal the following up-titration or down-titration scheme (including discontinuation if needed) will be applied:

- At randomization Visit (V3), the starting dose of SAR236553 will be 75 mg every 2 weeks (Q2W). At Month 2, patients randomized to SAR236553 will, in a blinded manner, either:
  - Continue SAR236553 75 mg Q2W, if the Month 1 LDL-C is <50 mg/dL (1.29 mmol/L) **OR**
  - Be up-titrated to SAR236553 150 mg Q2W, if the Month 1 LDL-C is ≥ 50 mg/dL (1.29 mmol/L).
- At subsequent visits, for patients on SAR236553, the following adjustments may be applied:

- For patients receiving 150 mg Q2W:

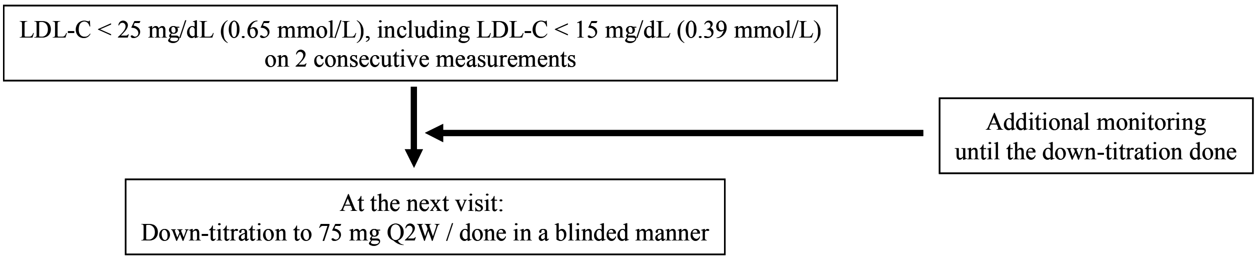

- For patients receiving 75 mg Q2W:

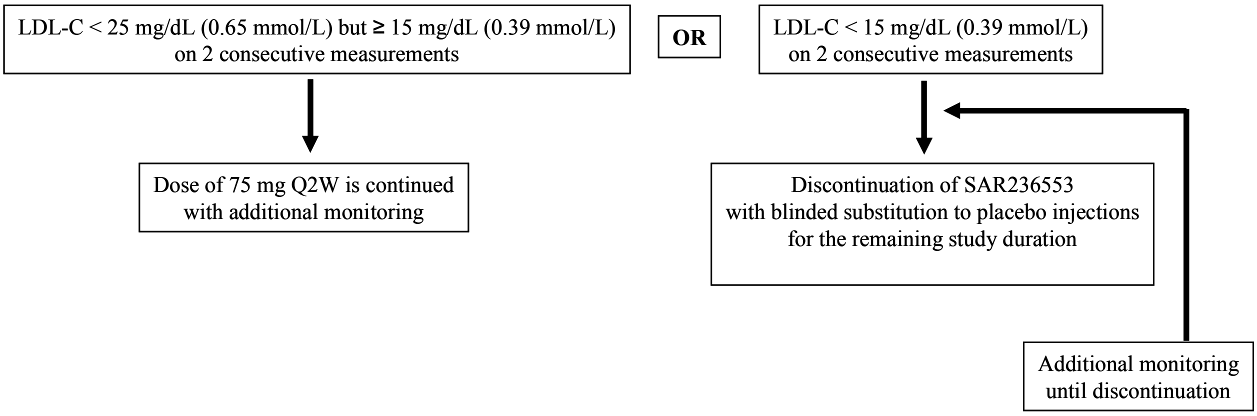

As lipid values will not be communicated to investigators after randomization to maintain the blind, the continuation of the 75 mg dose, the up-titration to the 150 mg dose, the down-titration from 150 to 75 mg, or discontinuation will occur automatically, without site or patient awareness. Moreover, blinded dose adjustment will avoid the need for dummy titrations in the placebo group.

Patient's who achieve 2 consecutive LDL-C levels < 25 mg/dL (0.65 mmol/L) during the study will be monitored and managed as per Appendix A . An independent external physician will be notified by the central laboratory of 2 consecutive LDL-C <25 mg/dL (0.65 mmol/L). The independent physician will review the unmasked LDL-C values and patient safety data, in close collaboration with the dedicated member of the Phase 3a DMC implemented for all Phase 3 studies evaluating the efficacy and safety of SAR236553 on LDL-C (called Phase 3a studies). This dedicated Phase 3a DMC member will then transmit any information as necessary to the common member involved in both DMCs (Phase 3a DMC and CV DMC).

Atorvastatin or rosuvastatin daily dose as well as dose of other non statin LMT (if applicable) should be stable from randomization up to the common study end date, unless safety reasons prompt dose reduction or discontinuation.

6.1.3 Follow-up contact

This follow-up contact will only be applicable for patients still on study treatment at the time of the common study end date; this follow-up contact should be scheduled 8 weeks (i.e., 2 months) after the common study end date and will be a phone call.

Note: all patients, even those who have achieved an endpoint or prematurely discontinued the study treatment will be followed, as scheduled by the protocol, from randomization until the common study end date.

6.2 DURATION OF STUDY PARTICIPATION.

6.2.1 Duration of study participation for each patient

The duration of the run-in period must be at least 4 weeks, and randomization must occur no later than16 weeks (+ 3 days) after the index ACS event.

The double-blind treatment period will continue until each surviving randomized patient has been followed for a minimum of 24 months or the target number of events (1613) is reached, whichever comes last. The corresponding estimated study duration is 64 months (as described in the sample size considerations).

Patients who experience an ongoing SAE or an Adverse Event of Special Interest (AESI) (see Section 10.4.2), at the common study end date, should be followed until resolution, stabilization, or death and related data will be collected.

The end of the study per patient (regardless of the patient status, prematurely discontinued or completer) is the common study end date or the resolution/stabilization of all SAEs, and AESI, whichever comes last.

6.2.2 Determination of end of clinical trial (all patients)

All randomized patients will be followed up to a common study end date, defined as the date when the last randomized patient reaches a 24-month follow-up or the target number of events (1613) is reached whichever comes last. This is planned to occur when the last surviving randomized patient reaches 24 months of follow-up (i.e., approximately 64 months after first patient randomized).

6.3 INTERIM ANALYSIS

Patients will be followed until 1,613 patients experience at least one primary endpoint event or for approximately 24 months after the date of the last randomized patient, whichever comes last.

Interim analyses for futility will be performed, under the supervision of the CV DMC, when 50% and 75% of events have occurred.

An interim analysis for efficacy will be performed when 75% of events have occurred. Control of the type I and type II error will be ensured using gamma (-5) spending function for type II error (futility) and Gamma (-22) for type I error (efficacy). Stopping rules details are further described in Section 11.5.

6.4 STUDY COMMITTEES

Executive Steering Committee:

The Executive Steering Committee is composed of university-based scientists (experts in cardiology field, and lipids) with clinical and methodological expertise, working in collaboration with Sponsor based scientists. The Steering Committee provides scientific and strategic direction for the trial and will have overall responsibility for its execution. The committee provides guidance on producing and conducting a scientifically sound design and ensuring accurate reporting of the study. In that capacity, the Steering Committee must address and resolve scientific issues encountered during the study.

Among its responsibilities, the Steering Committee will receive blinded (aggregate) study status reports from the Sponsor. The Steering Committee will also review the recommendations from the Data Monitoring Committee throughout the study. The Steering Committee members and Sponsor based scientists will participate in face-to-face meetings at regular intervals throughout the study and in regularly scheduled teleconferences.

Detailed activities and responsibilities of the Steering Committee are described in the Steering Committee charter.

Data Monitoring Committee:

An independent Data Monitoring Committee (DMC), composed of members independent from the Sponsor and the study Investigators, is implemented in order to monitor patient safety by conducting formal reviews of accumulated safety data that will be unblinded. This DMC exclusively dedicated to this study will be identified as CV DMC. The chairman of the DMC dedicated to the Phase 3a studies supporting the LDL-C reduction indication (Phase 3a DMC) will be also a member of this CV DMC and will serve as a liaison between the two DMCs. Safety data review will include the cardiovascular outcomes adjudicated or not at the time of this review. The CV DMC will also supervise the two interim analyses for futility and efficacy conducted when 50% and 75% of events occur (see Section 6.3). The CV DMC will provide the Sponsor and the Steering Committee with appropriate recommendations on the conduct of the clinical trial to ensure the protection and safety of the patients enrolled in the study. In addition, the CV DMC will also institute any measures that may be required for ensuring the integrity of the study results during the study execution.

Additionally, the CV DMC will thoroughly analyze the aggregate data for patients who achieve LDL-C < 25 mg/dL during their periodic reviews throughout the study and more particularly, will review adverse events potentially associated with LDL-C <25 mg/dL (0.65 mmol/L) (see Section 10.6.3 and Appendix A).

All activities and responsibilities of this CV DMC are described in the DMC charter.

Clinical Events Committee:

The Clinical Events Committee (CEC), managed by the Duke Clinical Research Institute (DCRI), is composed of experts in the field of cardiovascular diseases, independent from the Sponsor and the Investigators. This committee will be responsible for defining, validating and classifying, in a blinded fashion, all components of the primary and secondary endpoints related to cardiovascular outcomes as well as validating the classification of the cause of all deaths.

A charter and an adjudication operational manual specify the procedures, criteria, and classification used for adjudication of these events.

7 SELECTION OF PATIENTS

7.1 INCLUSION CRITERIA

I 01. Patients hospitalized for ACS defined by:

- Ischemic symptoms with unstable pattern, occurring at rest or minimal exertion within 24 hrs of an unscheduled hospital admission, due to presumed or proven obstructive coronary disease **AND at least one of the following (A and/or B)**:

A) Elevated cardiac biomarkers (troponin I or T or CK-MB mass with at least one determination >99th percentile or upper limit of normal for the laboratory).

OR

B) Resting ECG changes consistent with ischemia or infarction (B1) **AND** additional evidence of obstructive coronary disease, based upon the following criteria (B2):

B1. Resting ECG changes consistent with ischemia or infarction requires at least one of the following:

a) new or presumed new ST depression ≥ 0.5 mm in 2 contiguous leads;

b) new or presumed new ST elevation at the J-point in 2 contiguous leads with cut-offs ≥0.2 mV in men or ≥ 0.15 mV in women in leads V2-V3 and/or ≥ 0.1 mV in other leads or new or presumed new LBBB;

c) new or presumed new T wave inversion ≥ 1 mm in leads with predominant R wave in 2 contiguous leads;

d) new or presumed new pathologic Q waves ≥ 30 ms wide and ≥ 1 mm deep in any 2 leads of a contiguous lead grouping or ≥ 20 ms or QS complex in leads V2 and V3;

e) new tall R-wave ≥ 40 ms in V1 and V2 and R/S ≥ 1 in V1 with concordant positive T-wave in the absence of a conduction defect.

B2. Additional evidence of obstructive coronary disease requires at least one of the following:

a) new or presumed new evidence of myocardial ischemia or infarction by perfusion imaging.

b) new or presumed new regional wall motion abnormality.

c) epicardial coronary artery stenosis >70% by coronary angiography.

I 02. Patients not adequately controlled (as defined by at least one of the following: LDL-C ≥ 70 mg/dL [≥ 1.81 mmol/L], ApoB ≥ 80 mg/dL [≥ 0.8 mmol/L], or non-HDL-C ≥ 100 mg/dL [≥ 2.59 mmol/L]) at the the qualifying Visit (V2), despite evidence-based lipid lowering

therapy (including intensive atorvastatin/rosuvastatin therapy or maximally tolerated dose of either of these 2 statins, or other non statin LMTs).

I 03. Signed written informed consent.

7.2 EXCLUSION CRITERIA

Patients who have met all the above inclusion criteria listed in Section 7.1 will be screened for the following exclusion criteria which are sorted and numbered in the following 3 subsections:

7.2.1 Exclusion criteria related to study methodology

***Exclusion criteria for the entry into the run-in period and the double-blind treatment per**iod*

E 01. Age < 40 years.

E 02. Uncontrolled hypertension (SBP > 180 mmHg or DBP > 110 mmHg) at V2 or V3

E 03. History of New York Heart Association (NYHA) class III or IV congestive heart failure persisting despite treatment or, if measured, LVEF < 25% at the most recent measurement.

E 04. Known history of hemorrhagic stroke.

E 05. History of cancer within the past 5 years, except for adequately treated basal cell skin cancer, squamous cell skin cancer, or in situ cervical cancer.

E 06. Known history of untreated hypothyroidism.

E 07. Patient who has previously participated in any clinical trial of SAR236553 or any other anti-PCSK9 monoclonal antibody.

E 08. Patient who has taken other investigational drugs within 1 month or 5 half lives, whichever is longer.

E 09. Laboratory findings measured during screening and before randomization visit:

- Positive test for hepatitis B surface antigen and/or hepatitis C antibody.
- Triglycerides (TG) > 400mg/dL (>4.52 mmol/L) (1 repeat lab allowed).
- Positive serum or urine pregnancy test in females of childbearing potential.
- eGFR <30 mL/min/1.73 m^2 according to 4-variable MDRD Study equation (calculated by central lab).
- ALT or AST >3 x ULN on most recent determination prior to randomization (1 repeat lab is allowed).
- CPK >3 x ULN on most recent determination prior to randomization (1 repeat lab is allowed).

E 10. Conditions/situations such as:

- Any clinically significant abnormality identified at the time of screening that in the judgment of the Investigator or any sub-Investigator would preclude safe completion of the study or constrain endpoints assessment such as major systemic diseases, patients with short life expectancy.
- Patients considered by the Investigator or any sub-Investigator as inappropriate for this study for any reason, e.g.:
  - Those deemed unable to meet specific protocol requirements, such as scheduled visits.
  - Investigator or any sub-Investigator, pharmacist, study coordinator, other study staff or relative thereof directly involved in the conduct of the protocol, etc.
  - Presence of any other conditions (e.g., geographic, social….) actual or anticipated, that the Investigator feels would restrict or limit the patient's participation for the duration of the study.

Only for the entry into the double-blind treatment period (randomization):

E 11. All of the following: LDL-C < 70 mg/dL (< 1.81 mmol/L), ApoB < 80 mg/dL (<0.8 mmol/L), and non-HDL-C < 100 mg/dL (< 2.59 mmol/L) at the qualifying visit (V2).

E 12. Patients who have experienced an ACS event more than 16 weeks (+ 3 days) prior to randomization visit (V3).

E 13. Not on a stable dose of atorvastatin or rosuvastatin for at least 2 weeks prior to qualifying visit (V2 or V2b) and at least 4 weeks prior to randomization visit (V3), or not on a stable dose of any other authorized LMT for at least 4 weeks prior to qualifying visit (V2 or V2b) and at least 6 weeks prior to randomization visit (V3, Month 0).

E 14. Use of fibrates, other than fenofibrate within 6 weeks prior to randomization visit (Month 0) (i.e., within 4 weeks prior to qualifying visit (V2).

E 15. Any change in dose of atorvastatin, rosuvastatin or non-statin LMT after the qualifying visit (V2) without subsequent requalification for laboratory parameters.

E 16. Use of nutraceutical products or over-the-counter therapies that may affect lipids which have not been at a stable dose/amount for at least 6 weeks prior to randomization Visit (V3, Month 0).

E 17. Use of red yeast rice products during the run-in period up to randomization Visit (V3, Month 0).

E 18. Coronary revascularization (PCI or CABG) planned after randomization and/or performed within 2 weeks prior to the randomization Visit (V3, Month 0).

E 19. Patient who withdraws consent during the screening period (patient who is not willing to continue or fails to return).

7.2.2 Exclusion criteria related to the background therapy

E 20. All contraindications to atorvastatin, rosuvastatin or other LMTs or warning/precaution of use (when appropriate) as displayed in the respective National Product Labeling for these treatments.

7.2.3 Exclusion criteria related to the current knowledge of Sanofi compound

E 21. Known sensitivity to monoclonal antibody therapeutics.

E 22. Pregnant or breast-feeding women.

E 23. Women of childbearing potential with no effective contraceptive method of birth control and/or who are unwilling or unable to be tested for pregnancy.

Note: Women of childbearing potential must have a confirmed negative pregnancy test at screening and randomization visits. They must use an effective contraceptive method throughout the study, and agree to repeat urine pregnancy test at designated visits. The applied methods of contraception have to meet the criteria for a highly effective method of birth control according to the “Note for guidance on non-clinical safety studies for the conduct of human clinical trials for pharmaceuticals (CPMP/ICH/286/95)” (Note for guidance on non-clinical safety studies for the conduct of human clinical trials and marketing authorization for pharmaceuticals (CPMP/ICH/286/95). http://www.ema.europa.eu/pdfs/human/ich/028695en.pdf.) (33). Postmenopausal women must be amenorrheic for at least 12 months.

8 STUDY TREATMENTS

8.1 INVESTIGATIONAL MEDICINAL PRODUCT(S)

Sterile SAR236553 drug product will be supplied at a concentration of 75 mg/mL and 150 mg/mL both as 1 mL volume in an autoinjector.

Sterile placebo for SAR236553 will be prepared in the same formulation as SAR236553 without the addition of protein as 1 mL volume in an autoinjector.

8.1.1 Route and method of administration

A manual for IMP administration (injection instruction manual) will be provided to patients containing detailed instructions on use. Also, an administration package containing gauze, alcohol swabs, band aids, etc will be provided to the patients.

The IMP could be administered by self-injection or by another designated person (such as a spouse, relative, etc…). The used autoinjector will be discarded in a sharps container which will be provided to patients.

Patients will be asked to store the IMP in a refrigerator. Prior to administration, the IMP should be set outside in a safe location at room temperature for about 30 to 40 minutes. Thereafter, the IMP should be administered as soon as possible.

Instructions as outlined above should be provided to the patient (or another designated person [such as spouse, relative, etc...] who will administer the injections) at training and as needed during the course of the study. Close supervision and feedback should be given at the training visit, randomization visit, and other visits as needed.

8.1.2 Timing of administration

During the run-in period, patients (or their caregivers) will administer at least 2 subcutaneous injections of the placebo SAR236553 in order to train them to administer the self-injection and to ensure patient's acceptance of an injectable study treatment. These training injections should be performed on site at screening Visit (V1, Week-16 to Week-4) and qualifying Visit (V2, Week-2), respectively. A third training injection can be performed at an additional visit such as the optional visit V2b.

During the double-blind treatment period, SAR236553 or placebo will be administered subcutaneously every 2 weeks, starting at randomization Visit (Month 0) continuing up to the common study end date.

Further training with the scheduled double-blind IMP can be done at any time during the study as necessary.

Double-blind IMP will start as soon as possible after the call for randomization using the treatment kit number provided by the IVRS. The first injection after randomization will be done at the investigational site by the patient or another designated person (such as spouse, relative, etc…) under direct site staff supervision. Patients will be monitored at the investigational site for at least 30 minutes after this first double-blind injection.

IMP should ideally be administered every 2 weeks subcutaneously at approximately the same time of the day; however it is acceptable to have a window period of ± 3 days. The time of the day is based on patient's preference.

If by mistake or due to other circumstances an injection is delayed by more than 7 days or completely missed, then the patient should return to the original schedule of study treatment administration without administering delayed injections. On the other hand, if the delay is less than or equal to 7 days from the missed date, then the patient should administer the delayed injection and then resume the original schedule of study treatment administration.

8.2 NON-INVESTIGATIONAL MEDICINAL PRODUCT(S)

The following classes of drugs are identified as non-investigational medicinal products (NIMP) because the medication is a background therapy:

- Statins (atorvastatin, rosuvastatin).
- Cholesterol absorption inhibitors (ezetimibe).
- Bile acid-binding sequestrants (such as cholestyramine, colestipol, colesevelam).
- Nicotinic acid.
- Fenofibrate.
- Omega-3 fatty acids (≥ 1000 mg daily).

Please see Section 8.8 for further information.

8.3 BLINDING PROCEDURES

8.3.1 Methods of blinding

SAR236553 and placebo for SAR236553 will be provided in identically matched auto injector and packaged identically which includes labeling to protect the blind.

Each double-blind treatment kit will be labeled with a number, which will be generated by a computer program from Sanofi. The treatment kit numbers will be obtained by the Investigator at the time of patient randomization and subsequent patient visits scheduled via a centralized treatment allocation system that will be available 24 hours-a-day, 7 days-a-week.

In accordance with the double-blind design, study patients, Investigators and study site personnel will remain blinded to study treatment and will not have access to the randomization (treatment codes) except under circumstances described in Section 8.3.6.

8.3.2 Lipid parameters

Lipid parameter values from blood samples obtained after the randomization visit, run by the central lab, will not be communicated to the sites so that they cannot deduce the treatment group of their patients based on LDL-C level attained. The sponsor's operational team will not have access to lipid parameters after randomization and until after the final database lock has occurred.

For patients who achieve 2 consecutive LDL-C <25 mg/dL (0.65mmol/L) on SAR236553 and depending on the dose received, the following will be applied:

- If the dose is 150 mg Q2W:

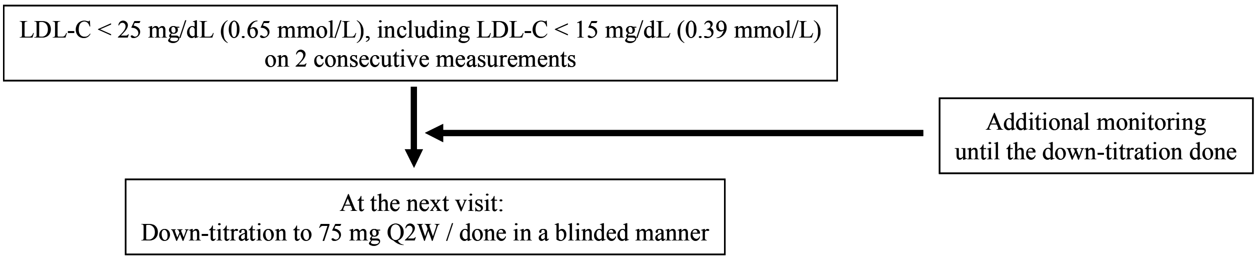

- If the dose is 75 mg Q2W:

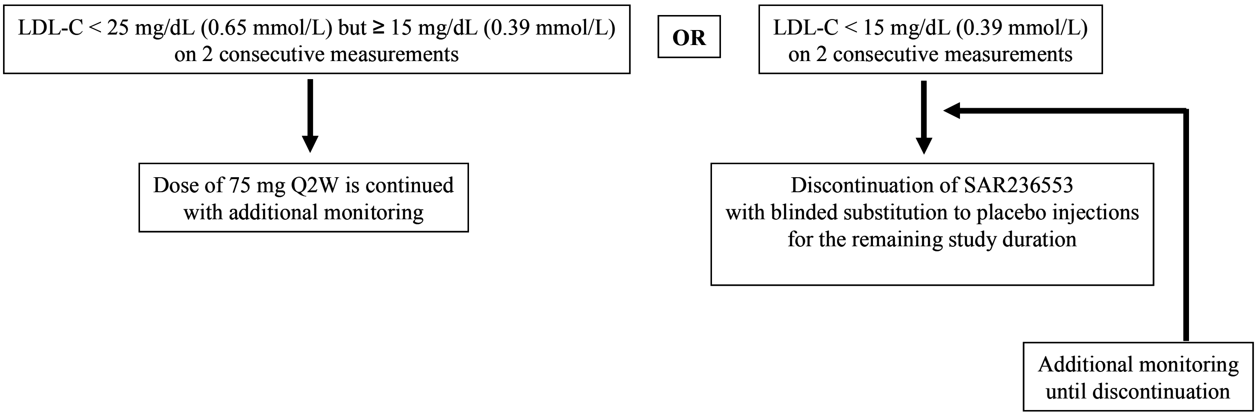

As described above, in case of LDL-C < 25 mg/dL on 2 consecutive measurements and until specific actions are undertaken, patients will be monitored according to process outlined in Section 10.6.3 and Appendix A. In order to maintain the integrity of the blind as much as possible with this monitoring process, the following points will be undertaken:

- Specific steps will be in place to ensure that the work which will be carried out by the central lab group and the communication with the independent external physician(s) (also known as independent physician), who is responsible for closely monitoring patients with these 2 consecutive LDL-C levels, will be in strict confidence.
- The independent physician(s) and the dedicated member of the Phase 3a DMC (implemented for all Phase 3 studies evaluating the efficacy and safety of SAR236553 on LDL-C) will work in close collaboration and independently from the clinical team and the sites. This dedicated Phase 3a DMC member will then transmit any information as necessary to the common member involved in both DMCs (CV DMC and Phase 3a DMC).
- The actual LDL-C levels will not be reported to the sites.
- Monitoring will be discontinued when specific actions are undertaken as described above, unless patients still display a LDL-C < 25 mg/dL.

8.3.3 Anti-SAR236553 antibodies

Patients' anti-SAR236553 antibody results will not be communicated to the sites during the study.

The sponsor's operational team will not have access to anti-SAR236553 antibodies associated with patient identification until after the final database lock has occurred.

The lab technicians involved in the determination of patients' anti-SAR236553 antibodies are excluded from the operational team and a process will be set up to prevent any potential unblinding.

Patients who have titers at or above 240 for anti-SAR236553 antibodies at the common study end date will have an additional antibody sample within 12 months after this date.

In order to maintain the blind of the study, the requests for sample collection of post-study anti-SAR236553 antibodies will also be made on patients with titers below 240 at the common study end date.

8.3.4 Committees

The independent Clinical Events Committee will review and adjudicate events in a blinded manner.

The Data Monitoring Committee will receive blinded by treatment group or unblinded (if necessary) confidential reports from an independent statistician for review (section 6.4)

8.3.5 Data Analysis

Regular DMC safety analyses and both Interim Analyses will be performed by the external independent CV DMC Statistician and will be reviewed under the supervision of the CV DMC.

8.3.6 Randomization code breaking during the study

In case of an Adverse Event (AE), the code must be broken by the site only in exceptional circumstances when knowledge of the IMP is essential for treating the patient. If possible, a contact should be initiated with the Monitoring Team/Medical Monitor before breaking the code. All calls will be documented by the Monitoring Team as appropriate to include date and time of the call, name of the person contacted within the Monitoring Team, patient ID, documentation of the request, and decision for unblinding or not.

Code breaking can be performed at any time by using the proper module of the centralized treatment allocation system and/or by calling any other phone number provided by the Sponsor for that purpose. However, it is preferable to contact the Medical Monitor to discuss the case before unblinding the case. If the blind is broken, the Investigator should document the date, time of day, and reason for code breaking, and report this information (or "relevant information as required by") on the appropriate page of the e-CRF.

Note that when documenting the reason for unblinding, the Investigator must not provide any detail regarding the nature of the IMP. The Investigator should not divulge IMP detail to the Sponsor's representative or to any staff members until database closure. Furthermore, when completing forms (e.g., AE, SAE), the study treatment should not be disclosed on the forms.

The code-breaking can also be performed by contacting the "24 hour alert system"; but this system should be used in very exceptional cases only (i.e., unavailability of a centralized treatment allocation system or inability to contact Investigator and/or site staff). However, the preferred option is to unblind using a centralized treatment allocation system. The Investigators will be informed by the clinical monitoring team about the availability of the local code-breaking details (through an emergency centralized 24 hour telephone system for use with e-SMS). A patient card, including the relevant "24 hour alert system" telephone number will be provided to every patient who will participate in the study.

Unblinding may also be performed by the Sponsor for some Serious Adverse Events that are both related and unexpected in order to conform to regulatory reporting requirements.

8.4 METHOD OF ASSIGNING PATIENTS TO TREATMENT GROUP

The randomized list of treatment kit numbers will be generated centrally by Sanofi. The IMP (SAR236553 75 mg or 150 mg kit, or placebo kit) will be packaged in accordance with this list.

The Project Demand manager will provide the randomized list of treatment kit numbers and the Study Biostatistician will provide the randomization scheme to the centralized treatment allocation system provider. Then, this centralized treatment allocation system provider will

generate the patient randomization list according to which it will allocate the treatment kits to the patients.

Patients will be randomized to receive either placebo or SAR236553 during the double-blind study treatment period using a ratio 1:1, with permuted-block randomization. Randomization will be stratified according to country.

The treatment kit numbers will be allocated using the centralized treatment allocation system on randomization visit (Day 1, Month 0), Month 2, Month 4, every 4 months up to Month 24, and then every 6 months up to Month 64.

For patients in the SAR236553 treatment arm, the treatment kit allocated at Month 2 will be based on their Month 1 LDL-C level following the up-titration rules (see Section 6.1). Regular transfer of data will be planned between the central laboratory and the centralized treatment allocation system provider in order to proceed in a blinded manner for study sites and sponsor.

Before randomizing a patient, the Investigator or designee will have to contact the centralized treatment allocation system.

A randomized patient is defined as a patient who is registered and assigned with a treatment kit number from the centralized treatment allocation system, as documented from its log file. A patient cannot be randomized more than once in the study. If a treatment is used without contacting the centralized treatment allocation system “patient will be considered as not randomized and withdrawn from the study.

Two types of centralized treatment allocation system will be used, the Interactive Voice Response System (IVRS) and the Interactive Web Response System (IWRS) depending on the choice of the site.

8.5 PACKAGING AND LABELING

For the double-blind treatment period, each double-blind treatment kit, either SAR236553 or placebo, will be prepared to contain 6 autoinjectors in a child-resistant package.

In order to protect the blind, all double-blind treatment kit boxes will have the same look and feel and therefore will be labeled with a double-blind label.

In addition to the double-blind treatment kits, a training kit containing 1 placebo autoinjector each will be prepared for the purpose of instructing patients on injection administration which is to be performed prior to randomization. These training injections should be performed on site at screening Visit (V1, Week-16 to Week-4) and qualifying Visit (V2, Week-2), respectively. A third training injection can be performed at an additional visit such as the optional visit V2b.

The content of the labeling is in accordance with the local regulatory specifications and requirements.

8.6 STORAGE CONDITIONS AND SHELF LIFE

The IMP (SAR236553 or placebo for SAR236553) will be stored in a refrigerator between +2°C and +8°C (36°- 46° F) by the site. The temperature of the site refrigerator should be checked daily and recorded on a log sheet.

The IMP that will be stored at the investigational site should be kept in an appropriate locked room, under the responsibility of the Investigator or designee or other authorized person in accordance with the storage conditions indicated on the label.

After the supply of IMP kits to patients at the study site visits, appropriate provisions as necessary will be in place for transportation of the IMP kits from the study site to the patient's refrigerator.

8.7 RESPONSIBILITIES

The Investigator, the Pharmacist, or other personnel allowed to store and dispense IMP will be responsible for ensuring that the IMP used in the clinical trial is securely maintained as specified by the Sponsor and in accordance with the applicable regulatory requirements.

All IMP shall be dispensed after IVRS contact in accordance with the Clinical Trial Protocol and it is the Investigator's responsibility to ensure that an accurate record of IMP issued and returned is maintained.

Any quality issue noticed with the receipt or use of an IMP (deficiency in condition, appearance, pertaining documentation, labeling, expiration date, etc.) should be promptly notified to the Sponsor. Some deficiencies may be recorded through a complaint procedure (i.e. Product Technical Complaint (PTC) form).

A potential defect in the quality of IMP may be subject to initiation of a recall procedure by the Sponsor. In this case, the Investigator will be responsible for promptly addressing any request made by the Sponsor, in order to recall IMP and eliminate potential hazards.

Under no circumstances will the Investigator supply IMP to a third party, allows the IMP to be used other than as directed by this Clinical Trial Protocol, or dispose of IMP in any other manner.

8.7.1 Treatment accountability and compliance

IMP administration data will be recorded by the patients onto a patient's diary.

Measures taken to ensure and document IMP compliance and accountability are described below:

- The Investigator or designee will obtain via IVRS/IWRS the treatment kit number(s) and he/she will dispense the treatment kit(s) to the patient.
- The accountability is to be performed at IMP kit re-supply visits only (see Section 10.1.4). The used and unused kit(s) should be brought back to such visits for accountability purposes.

- The Investigator or designee will complete the corresponding treatment log form from patient's diary.
- The Investigator/study coordinator will enter data in the appropriate e-CRF pages, according to data recorded in the treatment log form.
- The monitor will check the data consistency between e-CRF pages, treatment log forms using patient's diary, and returned unused autoinjectors of a corresponding kit.

8.7.2 Return and/or destruction of treatments

All treatments kits will be retrieved by the Sponsor. A detailed treatment log of the returned IMP will be established with the Investigator or designee and countersigned by the Investigator and the Monitoring Team.

8.8 CONCOMITANT MEDICATION

A concomitant medication is any treatment received by the patient concomitantly to the study (until the common study end date).

All patients should receive contemporary evidence-based treatment for ACS and chronic CHD as described in regional professional guidelines, including, but not limited to anti-platelet agents, beta blockers, angiotensin-converting-enzyme (ACE) inhibitors or angiotensin-receptor blockers (ARBs), and treatments for diabetes, hypertension, and smoking. Besides the specific information related to concomitant medications provided in this section, any other concomitant medication(s) will be allowed and will have to be recorded in the e-CRF and source data.

For background LMT, including statins, sites must follow the national product label for the safety monitoring and management of patients.

Nutraceutical products or over-the-counter therapies that may affect lipids are allowed only if they have been used at a stable dose for at least 6 weeks prior to randomization visit (Month 0), and throughout the study. Examples of such nutraceutical products or over-the-counter therapies include omega-3 fatty acids at doses <1000 mg, plant stanols such as found in Benecol, flax seed oil, and regular, ongoing use of psyllium.

9 ASSESSMENT OF INVESTIGATIONAL MEDICINAL PRODUCT

9.1 EFFICACY ENDPOINTS

Any suspected cardiovascular event suggestive of an endpoint as well as all deaths will be submitted to the Clinical Events Committee (CEC). The CEC will review the data of the reported cases in a blinded manner for adjudication purpose and will validate if the event should be considered as an endpoint. The cardiovascular events adjudicated and validated by the CEC will be used for the analyses.

Events that the CEC couldn't classify as well as suspected event according to investigator but not confirmed by the CEC will not be part of the outcomes.

9.1.1 Primary efficacy endpoint

The primary efficacy endpoint is the time from randomization to first occurrence of one of the following clinical events, as determined by the CEC:

- CHD death (including "undetermined causes of death" as per the CEC)
- Any non-fatal MI;
- Fatal and non-fatal ischemic stroke (including "undetermined causes of stroke" as per the CEC);
- Unstable angina requiring hospitalization.

If none of these events is observed at the time of the analysis cut-off date (final or interim, depending of the analysis, see Section 11.4.2.1 for details), the patient will be censored at the date of last contact, at the date of death, or at the date of cut-off, whichever comes first.

Of note, suspected event according to the investigator but not confirmed by the CEC will not be part of the primary efficacy outcome; their description will be provided separately.

9.1.2 Secondary efficacy endpoints

Time-to-event secondary endpoints will be censored using the same methodology as for the primary efficacy endpoint.

9.1.3 Main Secondary Efficacy Endpoint(s):

- Time from randomization to first occurrence of any CHD event (major CHD event, unstable angina requiring hospitalization, hospitalization for unanticipated coronary revascularization procedure).
- Time from randomization to first occurrence of any major CHD event (CHD death, non-fatal MI).

- Time from randomization to first occurrence of any CV event defined as follows: any non-fatal CHD event, any CV death, and non-fatal ischemic stroke.
- Time from randomization to first occurrence of all cause mortality, non-fatal MI, non-fatal ischemic stroke.
- Time from randomization to death (all cause mortality).

9.1.4 Other Secondary Efficacy Endpoint(s):

- Component of the primary endpoint considered individually:
  - Time from randomization to CHD death;
  - Time from randomization to first occurrence of any non-fatal MI;
  - Time from randomization to first occurrence of fatal or any non-fatal ischemic stroke;
  - Time from randomization to first occurrence of any unstable angina requiring hospitalization.
- Time from randomization to first occurrence of any hospitalization for unanticipated coronary revascularization procedure.
- Time from randomization to first occurrence of any congestive heart failure requiring hospitalization.

9.1.5 Efficacy assessment methods

Definitions of the primary and secondary efficacy endpoints related to CV events and death are based on FDA/CDISC *Standardized Definitions for End Point Events in Cardiovascular Trials,* and on the Thygesen Universal Definition for the definition of myocardial infarction (34) (35) (36).

9.1.6 Definitions of components of the composite primary efficacy endpoint

9.1.6.1 Coronary Heart Disease (CHD) Death

Coronary Heart Disease Death is defined as the subset of Cardiovascular deaths for which there is a clear relationship to underlying coronary heart disease, including death secondary to acute MI, sudden death, heart failure, complication of a coronary revascularization procedure performed for symptoms, coronary disease progression, or new myocardial ischemia where the cause of death is clearly related to the procedure, unobserved and unexpected death, and other death that cannot definitely be attributed to a nonvascular cause (see Section 9.1.7.1.1 for the different definitions).

9.1.6.1.1 Death due to acute myocardial infarction

Death by any mechanism (arrhythmia, heart failure, low output) within 30 days after a myocardial infarction (MI) related to the immediate consequences of the myocardial infarction, such as progressive congestive heart failure (CHF), inadequate cardiac output, or refractory arrhythmia. If these events occur after a “break” (e.g., a CHF and arrhythmia free period of at least a week), they

should be designated by the immediate cause, even though the MI may have increased the risk of that event (e.g., late arrhythmic death becomes more likely after an acute myocardial infarction (AMI)). The acute myocardial infarction should be verified to the extent possible by the diagnostic criteria outlined for acute myocardial infarction or by autopsy findings showing recent myocardial infarction or recent coronary thrombus.

Sudden cardiac death, if accompanied by symptoms suggestive of myocardial ischemia, new ST elevation, new LBBB, or evidence of fresh thrombus by coronary angiography and/or at autopsy should be considered death resulting from an acute myocardial infarction, even if death occurs before blood samples or 12-lead electrocardiogram (ECG) could be obtained, or at a time before the appearance of cardiac biomarkers in the blood.

Death resulting from a procedure to treat a myocardial infarction percutaneous coronary intervention (PCI), coronary artery bypass graft surgery (CABG), or to treat a complication resulting from myocardial infarction, should also be considered death due to acute MI.

Death resulting from a procedure to treat myocardial ischemia (excluding direct treatment of acute myocardial infarction) are considered as death due to other cardiovascular causes, even if an acute myocardial infarction occurs as direct consequence of a cardiovascular investigation/procedure/operation.

9.1.6.1.2 Sudden cardiac death

Death that occurs unexpectedly, not following an acute MI, and includes the following deaths:

a) Death witnessed and instantaneous without new or worsening symptoms.
b) Death witnessed within 60 minutes of the onset of new or worsening cardiac symptoms, unless documented (i.e. by ECG or other objective) to be due to acute myocardial infarction.
c) Death witnessed and attributed to an identified arrhythmia (e.g., captured on an electrocardiographic (ECG) recording, witnessed on a monitor, or unwitnessed but found on implantable cardioverter-defibrillator review).
d) Death after unsuccessful resuscitation from cardiac arrest.
e) Death after successful resuscitation from cardiac arrest and without identification of a non-cardiac etiology (Post-Cardiac Arrest Syndrome).
f) Unwitnessed death without other cause of death (information regarding the patient's clinical status preceding death should be provided, if available).

General considerations

A subject seen alive and clinically stable 12-24 hours prior to being found dead without any evidence or information of a specific cause of death should be classified as "sudden cardiac death."

Typical scenarios include:

- Subject well the previous day but found dead in bed the next day
- Subject found dead at home on the couch with the television on

9.1.6.2 Non-fatal myocardial infarction (MI)

It is expected that the definitions for myocardial will be updated by the Universal working group in a near future, for this reason the different definitions are more detailed in the Clinical Event Committee charter in which they will be updated once the Universal working group provides new MI definition standard. MI events are classified / defined into 3 general categories as follows:

9.1.6.2.1 Spontaneous MI (No Recent Procedures)

Detection of rise and/or fall of cardiac biomarkers (CK-MB or troponin) with at least one value above the 99th percentile of the upper reference limit (URL)* together with evidence of myocardial ischemia **with at least one of the following:**

- Symptoms of ischemia (Angina or equivalent symptoms that need to be treated medically or lasting ≥ 20 min. Ischemic symptoms as determined by the treating physician include but are not limited to defined as weakness, shortness of breath, wheezing, tiredness, fainting, sweating, nausea/vomiting, abdominal pain, back pain, jaw pain, palpitations, fast heartbeat, drug use for chest pain (nitroglycerin, morphine, beta blocker etc.)) **OR.**
- ECG changes indicative of new ischemia [new ST-T changes or new left bundle branch block (LBBB)] **OR**
- Development of pathological Q waves **OR**
- Imaging evidence of new loss of viable myocardium or new regional wall motion abnormality.

9.1.6.2.2 Peri-PCI MI

The definition is detailed in the CEC charter.

9.1.6.2.3 Peri-CABG MI

The definition is detailed in the CEC charter.

9.1.6.3 Fatal and Non-fatal stroke

Stroke is defined as an acute episode of focal or global neurological dysfunction caused by brain, spinal cord, or retinal vascular injury as a result of hemorrhage or infarction.

Classification:

a) Ischemic Stroke

Ischemic stroke is defined as an acute episode of focal cerebral, spinal, or retinal dysfunction caused by infarction of central nervous system tissue.

Hemorrhage may be a consequence of ischemic stroke. In this situation, the stroke is an ischemic stroke with hemorrhagic transformation and not a hemorrhagic stroke.

b) Hemorrhagic Stroke

Hemorrhagic stroke is defined as an acute episode of focal or global cerebral or spinal dysfunction caused by intraparenchymal, intraventricular, or subarachnoid hemorrhage

c) Undetermined Stroke

Undetermined stroke is defined as an acute episode of focal or global neurological dysfunction caused by presumed brain, spinal cord, or retinal vascular injury as a result of hemorrhage or infarction but with insufficient information to allow categorization as a) or b).

Fatal stroke

Death due to Stroke refers to death occurring up to 30 days after a stroke that is either due to the stroke (fatal stroke) or caused by a complication of the stroke (stroke contributing to death).

9.1.6.4 Unstable angina requiring hospitalization

A diagnosis of a qualifying ACS event without elevations in cardiac biomarkers that meets the primary endpoint requires the following:

- Admission to hospital or emergency room (exceeding 23 hrs) with symptoms presumed to be caused by myocardial ischemia with an accelerating tempo in the prior 48 hrs and/or prolonged (at least 20 min) rest chest discomfort

AND

- New ECG findings consistent with ischemia or infarction (or presumed new if no prior ECG available). ECG findings are subcategorized as highest risk or abnormal but not highest risk as defined below:
  - Highest Risk ECG Criteria:
  a) New or presumed new STD > 0.5mm in 2 contiguous leads or T wave inversion > 1mm in leads with prominent R wave or R/S >1 in 2 contiguous leads.
  b) New or presumed new STE at the J point in > 2 contiguous leads > 0.2mV in V2 or V3 in men or > 0.15 mV in women in V2 or V3 or > 0.1mV in other leads. LBBB (new or presumed new).
  - Abnormal but not highest risk ECG Criteria:
  a) New ECG abnormalities that are less severe and do not meet the high risk criteria as described above.

AND

- Definite evidence of inducible myocardial ischemia as demonstrated by:
  - Angiographic progression of epicardial coronary stenosis compared with a prior study (excluding restenosis at site of prior coronary intervention) **OR**
  - Need for coronary revascularization procedure **OR**
  - Angiographic evidence of at least 70% epicardial coronary stenosis.

9.1.7 Definitions of the secondary efficacy endpoints

9.1.7.1 Death

All deaths will be categorized as Cardiovascular, non-Cardiovascular or Unknown based on the definitions below. In addition, all deaths will also be categorized as Coronary Heart Disease Death and further sub-typed based on the specific Cardiovascular and non-Cardiovascular categories defined below.

9.1.7.1.1 Definition of Cardiovascular Death

Cardiovascular Death is defined as death resulting from an acute myocardial infarction, sudden cardiac death, death due to heart failure, death due to stroke, and death due to other cardiovascular causes.

9.1.7.1.1.1 Coronary Heart Disease (CHD) Death

Definition of CHD death is described in Section 9.1.6.1.

9.1.7.1.1.2 Other Cardiovascular Deaths

<u>Death due to Heart Failure or Cardiogenic Shock:</u>

Death occurring in the context of clinically worsening symptoms and/or signs of heart failure without evidence of another cause of death and not following an acute MI (AMI). Deaths due to heart failure can have various etiologies, including prior AMIs (late effect), ischemic or non-ischemic cardiomyopathy, or valve disease.

a) New or increasing symptoms and/or signs of heart failure requiring the initiation of, or an increase in, treatment directed at heart failure or occurring in a patient already receiving maximal therapy for heart failure.

b) Heart failure symptoms or signs requiring continuous intravenous therapy or chronic oxygen administration for hypoxia due to pulmonary edema.

c) Confinement to bed predominantly due to heart failure symptoms.

d) Pulmonary edema sufficient to cause tachypnea and distress **not** occurring in the context of an acute myocardial infarction, worsening renal function, or as the consequence of an arrhythmia occurring in the absence of worsening heart failure.

e) Cardiogenic shock **not** occurring in the context of an acute myocardial infarction or as the consequence of an arrhythmia occurring in the absence of worsening heart failure. Cardiogenic shock is defined as systolic blood pressure (SBP) < 90 mm Hg for greater than 1 hour, not responsive to fluid resuscitation and/or heart rate correction, and felt to be secondary to cardiac dysfunction and associated with at least one of the following signs of hypoperfusion:

- Cool, clammy skin or
- Oliguria (urine output < 30 mL/hour) or
- Altered sensorium or
- Cardiac index < 2.2 L/min/m²

Cardiogenic shock can also be defined if SBP < 90 mm Hg and increases to ≥ 90 mm Hg in less than 1 hour with positive inotropic or vasopressor agents alone and/or with mechanical support.

Death due to Stroke (see Section 9.1.6.3).

Death due to Stroke refers to death occurring up to 30 days after a stroke that is either due to the stroke (fatal stroke) or caused by a complication of the stroke (stroke contributing to death).

Death due to Other Ischemic Cardiovascular Causes:

Death consequence of complication of arterial atherosclerotic disease, not included in any of the above categories.

Death due to Cardiovascular Cause other than ischemia

Example: Pulmonary embolism, aortic aneurysm rupture, complications of cardiac surgery or non-surgical revascularization.

Non-cardiovascular death is defined as any death that is not thought to be due to a cause. The following categories may be collected

9.1.7.1.2 Definition of Non-Cardiovascular Death

Non-cardiovascular death is defined as any death that is not thought to be due to a cardiovascular (CV) cause. The following categories may be collected.

9.1.7.1.2.1 Non-Malignant Causes

- Pulmonary.
- Renal.

- Gastrointestinal.
- Hepatobiliary.
- Pancreatic.
- Infection (includes sepsis).
- Non-infectious (e.g., systemic inflammatory response syndrome (SIRS)).
- Hemorrhage* excluding hemorrhagic strokes and bleeding in the setting of coronary revascularization.
- Non-CV system organ failure (e.g., hepatic failure).
- Non-CV surgery.
- Accidental/Trauma.
- Suicide.
- Drug overdose.
- Other non-CV, specify: _________________

*Examples:
Death due to GI bleeding is not considered a CV death. Death due to retroperitoneal hematoma following PCI is considered CV death. Death due to intracerebral hemorrhage is considered CV death.

9.1.7.1.2.2 Malignant Causes

- Death results directly from the cancer; **OR**
- Death results from a complication of the cancer (e.g. infection, complication of surgery / chemotherapy / radiotherapy); **OR**
- Death results from withdrawal of other therapies because of concerns relating to the poor prognosis associated with the cancer.

Cancer deaths may arise from cancers that were present prior to randomization or which developed subsequently should be further classified (worsening prior malignancy; new malignancy).

9.1.7.1.3 Definition of Unknown Cause of Death

Unknown cause of death refers to a death not attributable to one of the above categories of cardiovascular death or to a non-cardiovascular cause, due to absence of any information (e.g., the only available information is “patient died”). The use of this category of death is discouraged and should apply to a minimal number of cases when no information at all on the circumstances of death is available (i.e. found on obituary of local newspaper). In all circumstances the reviewer will use all available information to attribute to one of the categories based on best clinical judgment.

9.1.7.2 Hospitalization for unanticipated coronary revascularization procedure.

A coronary revascularization procedure is a catheter-based or open surgical procedure designed to improve myocardial blood flow. Catheter-based tools (e.g., balloon catheters, cutting balloons, atherectomy devices, lasers, bare metal stents, and drug-eluting stents) improve myocardial blood flow by increasing the luminal area at a site of an obstructive coronary lesion. Aortocoronary bypass grafts CABG, (arterial, venous, or synthetic) improve myocardial blood flow by providing a conduit for blood flow distal to an obstructive coronary lesion. Insertion of a guidewire through a coronary guide catheter into a coronary vessel or aortocoronary bypass graft for the purpose of percutaneous coronary intervention (PCI) is considered intention for PCI. However, in the assessment of the severity of intermediate lesions with the use of intravascular ultrasound, Doppler flow velocity, or fractional flow reserve, insertion of a guidewire will NOT be considered PCI.

Elective: An elective procedure is one performed on a patient with stable cardiac function in the days or weeks prior to the procedure.

Non-Elective: A non-elective procedure is one performed on a patient who has been stabilized following initial treatment of acute coronary ischemia, and there is clinical consensus that the procedure should occur within the next 24 hours.

9.1.7.3 Congestive heart failure requiring hospitalization.

Heart failure (HF) requiring hospitalization is defined as an event that meets the following

Requires hospitalization defined as an admission to an inpatient unit or a visit to an emergency department that results in an overnight stay.

AND

Clinical symptoms of heart failure, including **at least one of the following**:

- New or worsening dyspnea.
- Orthopnea.
- Paroxysmal nocturnal dyspnea.
- Increasing fatigue/worsening exercise tolerance.

AND

Physical signs of heart failure, including **at least two of the following**:

- Edema (greater than 2+ lower extremity).
- Pulmonary crackles greater than basilar (pulmonary edema must be sufficient to cause tachypnea and distress not occurring in the context of an acute myocardial infarction or as the consequence of an arrhythmia occurring in the absence of worsening heart failure).
- Jugular venous distension.

- Tachypnea (respiratory rate > 20 breaths/minute).
- Rapid weight gain.
- S3 gallop
- Increasing abdominal distension or ascites.
- Hepatojugular reflux.
- Radiological evidence of worsening heart failure.
- A right heart catheterization within 24 hours of admission showing a pulmonary capillary wedge pressure (pulmonary artery occlusion pressure) ≥ 18 mm Hg or a cardiac output < 2.2 L/min/m².

Biomarker results (e.g., brain natriuretic peptide (BNP)) consistent with congestive heart failure will be supportive of this diagnosis, but the elevation in BNP cannot be due to other conditions such as cor pulmonale, pulmonary embolus, primary pulmonary hypertension, or congenital heart disease. Increasing levels of BNP, although not exceeding the ULN, may also be supportive of the diagnosis of congestive heart failure in selected cases (e.g. morbid obesity).

AND

Need for additional/increased therapy

Initiation of, or an increase in, treatment directed at heart failure or occurring in a patient already receiving maximal therapy for heart failure and including **at least one of the following**:

- Initiation of or a significant augmentation in oral therapy for the treatment of congestive heart failure.
- Initiation of intravenous diuretic, inotrope, or vasodilator therapy.
- Uptitration of intravenous therapy, if already on therapy.
- Initiation of mechanical or surgical intervention (mechanical circulatory support, heart transplantation or ventricular pacing to improve cardiac function), or the use of ultrafiltration, hemofiltration, or dialysis that is specifically directed at treatment of heart failure.

AND

No other non-cardiac etiology (such as chronic obstructive pulmonary disease, hepatic cirrhosis, acute renal failure, or venous insufficiency) and no other cardiac etiology (such as pulmonary embolus, cor pulmonale, primary pulmonary hypertension, or congenital heart disease) for signs or symptoms is identified.

NOTE: *It is recognized that some patients may have multiple simultaneous disease processes. Nevertheless, for the end point event of heart failure requiring hospitalization, the diagnosis of congestive heart failure would need to be the primary disease process accounting for the above signs and symptoms.*

9.2 SAFETY ENDPOINT(S):

Observation period

The observation of safety data will be as follows:

- PRE-TREATMENT period: The PRE-TREATMENT observation period is defined from the signed informed consent up to the first dose of double-blind IMP injection.
- TEAE period: The TEAE observation period is defined as the time from the first dose of double-blind IMP injection to the last dose of IMP injection + 70 days (10 weeks) as residual effect of treatment is expected until 10 weeks after the stop of double-blind IMP.
- POST-TREATMENT period: The POST-TREATMENT observation period is defined as the time starting the day after the end of the TEAE period up to the end of the study.

Rationale for TEAE period definition is detailed in Section 4.

9.2.1 Adverse event

All adverse events diagnosed by the Investigator, irrespective of the result of the adjudication for cardiovascular events, will be reported and described.

All AEs will be coded to a “Lowest Level Term (LLT)”, “Preferred Term (PT)”, “High Level term (HLT)”, “High Level Group Term (HLGT)” and associated primary “System Organ Class (SOC)” using the version of MedDRA (Medical Dictionary for Regulatory Activities) currently in effect at Sanofi at the time of the considered database lock.

AEs of special interest includes the following:

- Allergic events (using special e-CRF pages, see Section 10.6.2)
- Local injection site reactions (using special e-CRF pages, see Section 10.6.1)
- Hemolytic anemia (using special e-CRF pages, see Section 10.4.7.1)

Adverse event observation period

The AE observations are per the observation periods defined above.

Death observation period

The death observations are per the observation periods defined above.

9.2.2 Safety laboratory

The clinical laboratory data consist of urinalysis, hematology (red blood cell count, hemoglobin, hematocrit, platelets, white blood cell count with differential blood count), standard chemistry (glucose, sodium, potassium, chloride, bicarbonate, calcium, phosphorous, urea nitrogen, creatinine, uric acid, LDH, total protein, albumin, γ Glutamyl Transferase [γGT]), Hepatitis C antibody, liver panel (ALT, AST, ALP, and total bilirubin), and CPK.

Some additional safety laboratory parameters may be reflexively measured, based on actual data (please refer to Section 10.4.7).

Clinical laboratory values will be analyzed after conversion into standard international units. Standard international units will be used in all listings and tables.

9.2.3 Vital signs measurement

Vital signs include: weight, heart rate, systolic and diastolic blood pressure in sitting position.

9.2.4 Electrocardiogram measurement

Electrocardiogram (ECG) assessments will be described as normal or abnormal.

9.3 OTHER ENDPOINT(S):

9.3.1 Anti-SAR236553 antibody assessments

Anti-SAR236553 antibodies include the antibody status (positive/negative) and antibody titers.

9.3.1.1 Sampling time

Serum samples for anti-SAR236553 antibody determination will be drawn periodically throughout the study as per schedule noted in the study flowchart – Section 1.2. The first scheduled sample at randomization visit will be obtained before IMP injection (pre-dose).

Patients who have titers at or above 240 for anti-SAR236553 antibodies at the common study end date will have an additional antibody sample within 12 months after this date.

In order to maintain the blind of the study, the requests for sample collection of post-study anti-SAR236553 antibodies will also be made on patients with titers below 240 at the common study end date.

9.3.1.2 Sampling procedure

Detailed procedure of sample preparation, storage and shipment will be described in the specific laboratory manual which will be provided to sites. Five (5) ml blood volume is to be collected for each anti-SAR236553 antibody sample.

9.3.1.3 Bioanalytical method

All anti-SAR236553 antibody samples will be analyzed by the Regeneron Sample Analysis group.

Anti-SAR236553 antibody samples will be analyzed using a validated, non-quantitative, titer-based bridging immunoassay. It involves an initial screen, a confirmation assay based on drug specificity, and a measurement of the titer of anti-SAR236553 antibodies in the sample. [redacted]

Samples that are positive in the ADA assay will be assessed for neutralizing antibodies using a validated, non-quantitative, competitive ligand binding assay [redacted]

9.3.2 Lipid parameters

9.3.2.1 Endpoints

The percent changes from baseline to Month 4, to Month 24, and to the final analysis cut off date for the following parameters:

- Calculated LDL-C;
- ApoB;
- Non-HDL-C.

All measurements (scheduled or unscheduled, fasting or not fasting) may be used to provide a value for the endpoint considered, even if assessed after patient's discontinuation to the study treatment (ITT approach). The analysis windows used to allocate a time point to a measurement will be defined in the SAP.

9.3.2.2 Assessment method

LDL-C will be calculated using the Friedewald formula (37) . If TG values exceed 400 mg/dL (4.52 mmol/L), then the central lab will reflexively measure (via the beta quantification method) the LDL-C rather than calculating it. Apo B will be directly measured by the Central Laboratory. Non-HDL-C will be calculated by subtracting HDL-C from the total-C. Detailed procedures of sample preparation, storage and shipment will be described in the specific laboratory manual which will be provided to sites. Lipids parameters will be assessed from screening to common study end date.

9.3.3 hs-CRP

The percent change in hs-CRP from baseline up to the common study end date.

9.3.4 HbA_{1C}

The absolute change in HbA_{1c} (%) from baseline up to the common study end date.

9.3.5 EQ-5D Patient Questionnaire

EQ-5D is a standardized measure of health status developed by the EuroQol Group in order to provide a simple, generic measure of health for clinical and economic appraisal. The EQ-5D as a measure of health-related quality of life, defines health in terms of 5 dimensions: mobility, self-care, usual activities, pain/discomfort, anxiety/depression. Each dimension can take one of three responses (3 ordinal levels of severity): 'no problem' (1). “some problems” (2). “severe problems” (3). Overall health state is defined as a 5-digit number. Health states defined by the 5-dimensional classification can be converted into corresponding index scores that quantify health status, where 0 represents 'death' and 1 represents “perfect health” (See Appendix G) If response to one or more dimension is missing, the index score will be missing.)

EQ-5D variables include response of each EQ-5D items, index score and change of index score from baseline Week (38).

This questionnaire will only be administered in patients receiving the double-blind treatment. Patients who will prematurely discontinue will be asked to fill in this questionnaire until the early end of treatment visit (V70) to be performed at the time of discontinuation.

9.3.6 Pharmacogenomic Samples

An optional pharmacogenomic sub-study will be conducted to identify genetic associations with clinical or biomarker response to PCSK9 inhibition, hyperlipidemia, or cardiovascular disease. If needed, samples may also be used to identify markers associated with toxicity.

Randomized patients who agree to participate in the genomics sub-study will be required to sign a separate genomics sub-study informed consent form (ICF) prior to collection of the DNA sample. Blood for DNA extraction should be collected before IMP injection (pre-dose) on randomization visit; however, it could be collected at any time during the study. Patients who choose not to enroll in the genomics sub-study are still eligible to enroll in the primary study.

Special procedures for storage and shipping of pharmacogenomic samples are summarized below (Table 1) and are described in detail in Appendix B.

Table 1 - Summary of handling procedures for DNA storage samples

**Sample Type(s)**	Pharmacogenetics
**Blood Sample Volume**	6 mL
**Tube Type**	6 mL Becton Dickinson K2 EDTA VACUTAINER™ Plus tubes with HEMOGARD™ closure (PN367863/4) sterile tubes
**Anticoagulant**	K2 EDTA
**Blood Handling Procedures**	Keep blood on ice or frozen at -20°C (or colder) within 30 min of sampling time. DO NOT CENTRIFUGE.
**Storage Conditions**	In collection tube at approximately -20°C (or colder)

The Sponsor has included safeguards for protecting patient confidentiality. The blood sample and DNA that is extracted from it will be assigned a second number, a Genetic ID (de-identification code) that is different from the Subject ID. This “double coding” is performed to separate a patient’s medical information and DNA data. The clinical study data (coded by Subject ID) will be stored in a distinct database at a different location from the database containing the pharmacogenomic data (coded by Genetic ID). The key linking Subject ID and Genetic ID will be maintained by a third party, under appropriate access control. The matching of clinical data and pharmacogenomic data, for the purpose of data analysis, will be possible only by using this key, which will be under strict access control. All data will be reported only in coded form in order to maintain confidentiality.

DNA may be stored and used for analyses for up to 15 years from the completion of the Clinical Study Report. Analyses may include sequence determination or single nucleotide polymorphisms (SNP) from candidate genes. Candidate genes may include (but are not limited to) PCSK9, Apo B and LDL-R. Genome-wide studies, including (but not limited to) SNP analyses and/or genomic sequencing may also be performed.

10 STUDY PROCEDURES

The study consists of a run-in period of at least 4 weeks with randomization 16 weeks after the index ACS event at the latest. The double-blind, placebo-controlled treatment period will continue until each surviving randomized patient has been followed for a minimum of 24 months, its estimated duration is approximately 64 months. For patients who will complete the entire double-blind treatment period, the final visit that will correspond to the end of treatment visit will be followed 2-months after by a follow-up contact, which will be a telephone call.

A window period of ± 3 days is allowed for the visit Week -2. Then for all visits after Day 1/Month 0 (randomization visit), a timeframe of a certain number of days will be allowed. The window period for visits at Months 1 and 2 are ± 7 days, and for all other visits it is ± 14 days during the double-blind treatment period.

For all visits after Day 1/randomization visit, if one visit date is changed, then the next visit should take place according to the original schedule as outlined Section 1.2.

Ideally all the visits should take place in the morning approximately at the same time. However after randomization in case there is no other possibility for the patient, visits can be arranged later in the day.

For the phone calls/contacts via internet to be performed in between on-site visits, the timeframe will be ± 21 days too.

Blood samplings:

The blood sampling for determination of lipid parameters (e.g., LDL-C, Apo B, and non-HDL-C) should be preferably performed in the morning, in fasting condition (i.e. overnight, at least 10-12 hours fast and refrain from smoking) for all site visits throughout the study. Alcohol consumption within 48 hours and intense physical exercise within 24 hours preceding the blood sampling are discouraged.

Laboratory tests:

The laboratory data are collected in accordance with the study schedule in Section 1.2 and forwarded to the central laboratory:

- Hematology
- Chemistry
- Liver panel: in case of total bilirubin values above the normal range, differentiation into conjugated and non-conjugated bilirubin will occur automatically.
- Creatine Phosphokinase (CPK).
- Hepatitis B surface antigen

- Hepatitis C antibody: positive tests will be confirmed with reflexive testing.
- Serum pregnancy test.

Urine samplings:

- Urinalysis - dipstick will be performed on site and will assess for pH, specific gravity, and for the presence of blood, protein, glucose, ketones, nitrates, leukocyte esterase, uro-bilinogen and bilirubin. If the dipstick is abnormal then standard microscopy will be conducted.
- Urine pregnancy test – dipstick will be performed on site.

Notes:

Any clinically relevant abnormal laboratory value should be immediately rechecked (whenever possible using the central laboratory) for confirmation before making any decision for the concerned patient. It should be documented as an AE/SAE as applicable. Please also refer to Section 10.4.4.

Decision trees for the management of certain laboratory abnormalities by Sanofi are provided in Appendix C and Appendix D.

Other endpoints assessment methods

All other blood parameters will also be measured by a Central Laboratory during the study (as per the schedule in Section 1.2, on blood samples taken preferably in the morning in fasting condition (at least 10 to 12 hours fast and refrain from smoking). Alcohol consumption within 48 hours and intense physical exercise within 24 hours preceding the blood sampling are discouraged.

- Glycemic parameters (HbA_{1c}) and serum glucose will be measured by a Central laboratory, periodically throughout the study as per the schedule in Section 1.2.
- The blood sampling for inflammatory parameter, hs-CRP will be collected periodically throughout the study as per the schedule in Section 1.2.

Note: in case of high HbA_{1c} values at screening, the Investigator is responsible for the optimization of the patient's treatment to achieve HbA_{1c} targets as defined by local guidelines or the Standards of Medical Care in Diabetes-2012 by the American Diabetes Association (39).

Library samples

Library (plasma and serum) samples will be collected periodically throughout the study as per schedule noted in the study flowchart - Section 1.2. The first scheduled sample at randomization visit will be obtained before IMP injection (pre-dose).

Library samples will be coded to maintain patient confidentiality and may be stored for up to 10 years for exploratory research that may include the study of PCSK9 levels, PCSK9 function, effect(s) of PCSK9 inhibition with a monoclonal antibody, lipoprotein sub-fractionation, and mechanisms of hyperlipidemia and cardiovascular disease (e.g. lipoprotein–associated

phospholipase A2). If needed, samples may also be used to identify markers associated with toxicity. The library samples will never be used for genomic analysis.

Detailed procedure of sample preparation, storage and shipment will be described in the specific laboratory manual which will be provided to sites. Library samples will be sent to a central laboratory (only for randomized patients) for long-term storage between –70°C to –85°C.

- Plasma samples: 8.5 mL blood volume to be collected as specified in the specific laboratory manual.
- Serum samples: 2.5 mL blood volume to be collected as specified in the specific laboratory manual.

Physical examination:

A general physical examination should be performed at the time points indicated in the study schedule flowchart in Section 1.2. If a new clinically significant abnormality or worsening from baseline is detected after randomization, then an AE should be reported and the patient should be considered for further clinical investigations and/or specialist consultation as per the Investigator's medical judgment.

Blood pressure (BP)/heart rate:

BP should be measured in sitting position under standardized conditions, approximately at the same time of the day, on the same arm, with the same apparatus (after the patient has rested comfortably in sitting position for at least five minutes). Values are to be recorded in the e-CRF; both systolic blood pressure and diastolic blood pressure should be recorded. At the first screening visit, blood pressure should be measured in both arms. The arm with the highest diastolic pressure will be determined at this visit, and blood pressure should be measured on this arm throughout the study. This highest value will be recorded in the e-CRF.

Heart rate will be measured at the time of the measurement of blood pressure.

Notes: in case of high BP values at screening the Investigator is responsible for the optimization of the patient's treatment to achieve BP targets as defined by local guidelines or the Seventh Report of the Joint National Committee on Prevention, Detection, Evaluation, and Treatment of High Blood Pressure (JNC 7) (40).

ECG:

The 12-lead ECGs should be performed after at least 10 minutes rest and in the supine position. The electrodes should be positioned at the same place as much as possible, for each ECG recording throughout the study. The ECG will be interpreted locally by the Investigator. Any clinically significant abnormality should be documented as an AE/SAE as applicable (see Section 10.4.4).All ECG traces will be kept as source data.

Body weight and height

Body weight should be obtained with the patient wearing undergarments or very light clothing and no shoes, and with an empty bladder. The same scale should be used throughout the study.

The use of calibrated balance scales is recommended, if possible. Self-reported weights are not acceptable; patients must not read the scales themselves.

Height needs to be measured as self-reported heights are not acceptable.

10.1 VISIT SCHEDULE

10.1.1 Screening visit - Visit 1 (Week-16 to Week-4) and entry in the run-in period

The patient will receive verbal information concerning the aims and methods of the study, its constraints and risks and the study duration. A written summary in the form of an information leaflet will be given to the patient. The written informed consent must be signed by the patient and the Investigator prior to any investigations. Only patients who meet the inclusion criteria as noted in Section 7 may be screened. Women of childbearing potential will be requested to use a medically approved contraceptive method during the entire study. If it is planned to have another designated person administer the injections to the patient during the study, then this person should be present for the first injection-training done at this visit V1.

This visit will include:

- Complete informed consent.
- Assessment of inclusion and exclusion criteria.
- Demographic (age, gender, race, ethnicity).
- Patient’s medical and surgical history (including menopausal status and cardiovascular history), alcohol habits, and smoking habits.
- Patient’s cardiovascular history.
- Index ACS event: type of event and date of onset.
- Record of concomitant and previous medication (within the previous 3 months), including lipid-modifying treatments, cardiovascular medications, OTC and herbal medicine.
- Body weight and height measurements.
- Physical examination including vital signs: sitting systolic and diastolic blood pressure (SBP and DBP), heart rate.
- Collection of adverse events from this point onward:

All adverse events and serious adverse events will be collected from the time of informed consent signature and throughout the study until the post study treatment follow-up visit.

- IVRS/IWRS contact for notification of screening and entry in the run-in period:
  - Patients meeting the inclusion/exclusion criteria for eligibility at screening will enter the run-in period of the study. IVRS/IWRS is to be contacted for notification of screening and for patient number allocation (please note that it is important to have the IVRS/IWRS contact before any blood sample is drawn because the patient number is given by IVRS/IWRS and it must be reported on the requisition forms). This patient number is composed of a 9-digit number containing the 3-digit country code, the 3-digit center code and the 3-digit patient chronological number (the 3-digit patient chronological number is 001 for the first patient screened in a center, 002 for the second patient screened in the same center...).
  - Allocation of a batch number for training kit
  - Information on the dose of atorvastatin or rosuvastatin, or if not applicable specify that this is other LMT(s).
- Record batch number allocated in e-CRF.
- Urinalysis.
- Fasting blood sample for:
  - Lipids: TC, LDL-C, HDL-C, TG, non-HDL-C, ApoB.
  - Hematology: blood cell count including hematocrit, hemoglobin, red blood cell count, white blood cell count with differential count and platelets.
  - Chemistry: glucose, sodium, potassium, chloride, bicarbonate, calcium, phosphorous, urea nitrogen, creatinine, uric acid, LDH, total protein, albumin, and γGT.
  - Liver panel (ALT, AST, ALP, and total bilirubin).
  - HbA_{1c}.
  - CPK.
  - Hepatitis B surface antigen and hepatitis C antibody tests.
  - Serum pregnancy test (females of childbearing potential only).
- First injection-training on site:
  - Injection-training should be provided as outlined in Section 8.1.2.
  - The placebo should be administered by the patient or another designated person (such as spouse, relative, etc...) at the study site under supervision of site staff with appropriate feedback.
  - Record batch number allocated in e-CRF.
- An appointment will be given for the next visit at least two weeks after the screening visit provided that there is no change in the dose of LMT (e.g. atorvastatin or rosuvastatin as well as in the other non statin LMT (if applicable).
  - This visit has to be scheduled within 2 weeks prior to randomization visit V3 to obtain the laboratory parameters required for eligibility. However if necessary one additional

visit (V2b) and central laboratory assessments may occur during the run-in period if modifications to background statin or other LMTs are made after an initial visit V2, to ensure that lipid criteria are nonetheless fulfilled prior to randomization.

The patient should be seen in the morning and in fasting condition (i.e., overnight, at least 10-12 hours fast and refrain from smoking).

10.1.2 Qualifying visit - Visit 2 (Week-2, ± 3 days)

This visit will include:

- Assessment of inclusion and exclusion criteria.
- Record of concomitant medication.
- Vital signs: SBP and DBP, heart rate.
- Collection of adverse events.
- Check of the compliance and stability of the dose of the LMT (atorvastatin, rosuvastatin, ± other LMT).
- Fasting blood sample for:
  - Lipids: TC, LDL-C, HDL-C, TG, non-HDL-C, ApoB.
- Second injection-training on site:
  - IVRS/IWRS contact for allocation of a batch number for training kit.
  - Injection-training should be provided as outlined in Section 8.1.2.
  - The placebo should be administered by the patient or another designated person (such as spouse, relative, etc...) at the study site under supervision of site staff with appropriate feedback.
  - Record batch number allocated in e-CRF.
- An appointment will be given for the next visit according to the LMT prescribed. This appointment could be 2 weeks after provided that the dose of atorvastatin or rosuvastatin has been stable during these 2 weeks and for the other non statin LMT (if applicable) the dose has been stable for at least 4 weeks.
  - Remind patient to be in fasting conditions (i.e. overnight, at least 10-12 hours fast and refrain from smoking) for next visit. Also, alcohol consumption within 48 hours and intense physical exercise within 24 hours preceding the next visit are discouraged.
  - The patient will be instructed to continue the LMT at the same dose.

10.1.3 Optional qualifying visit - Visit 2b (Week-2, ± 3 days)

This visit will be performed in case the dose of atorvastatin or rosuvastatin as well as of the other non statin LMT (if applicable) has not been stable for at least 4 weeks at the time of the Visit V2.

This visit is similar to the Visit V2, and a third training injection can be performed.

- Third training injection as necessary
  - IVRS/IWRS contact for allocation of a batch number for training kit.
  - Injection-training should be provided as outlined in Section 8.1.2.
  - The placebo should be administered by the patient or another designated person (such as spouse, relative, etc...) at the study site under supervision of site staff with appropriate feedback.
  - Record batch number allocated in e-CRF.
- An appointment will be given for the next visit within 2 weeks.
  - Remind patient to be in fasting conditions (i.e. overnight, at least 10-12 hours fast and refrain from smoking) for next visit. Also, alcohol consumption within 48 hours and intense physical exercise within 24 hours preceding the next visit are discouraged.

10.1.4 Double-blind treatment period

10.1.4.1 Study site visits from Visit 3 (Month 0, D1) to Visit 30 (Month 64)

10.1.4.1.1 Baseline (randomization) visit - Visit 3 (Month 0, D1)

This visit will include:

- Assessment of inclusion and exclusion Criteria.
- Record of concomitant medication.
- Body weight measurement.
- Physical examination including vital signs: SBP and DBP, heart rate.
- Collection of adverse events.
- Check of the compliance and stability of the dose of the LMT (atorvastatin, rosuvastatin, ± other LMT).
- Informed consent form proposal for the optional genomic sub-study, and if patient agrees to participate then obtain written consent.
  - If patient declines participation in the genomic sub-study then this has no consequences for participation in the study otherwise.

If the patient is confirmed eligible, the Investigator will start the next study procedures:

- IVRS/IWRS contact for randomization and allocation of a 7-digit treatment kit number according to the randomization list. Investigators should never allocate a treatment kit number to a patient without contacting IVRS/IWRS.
- 12-lead ECG.
- Urinalysis.
- Urine pregnancy test (females of childbearing potential only).

- Blood sample for:
  - Lipids: TC, LDL-C, HDL-C, TG, non-HDL-C, ApoB, ApoA-1, ratio ApoB/ApoA-1, and Lp (a).
  - Hematology: blood cell count including hematocrit, hemoglobin, red blood cell count, white blood cell count with differential count and platelets.
  - Chemistry: glucose, sodium, potassium, chloride, bicarbonate, calcium, phosphorous, urea nitrogen, creatinine, uric acid, LDH, total protein, albumin, and γGT.
  - Liver panel (ALT, AST, ALP, and total bilirubin).
  - HbA_{1c}.
  - CPK.
  - hs-CRP.
  - Hepatitis C antibody test.
  - Library samples.
  - Anti-SAR236553 antibodies.
  - Genomic specimen collection (for specifically consented patients only).
- EQ-5D patient questionnaire: to be completed by the patient on site and data will be reported onto the e-CRF by site staff.
- Double-blind IMP kit dispensation as per treatment kit number provided by IVRS along with schedule reminder. The patient injection instruction manual and treatment administration package should be provided. The patient diary should be given and instructions on its completion should be reviewed.
- The first double-blind IMP injection will take place at the study site, but only after the collection of the fasting blood samples and after the assessment of all evaluations planned at that visit. Close supervision, feedback and further training to be provided for IMP administration. The patient should be stay in observation for at least 30 minutes after the injection.
- Reminders:
  - An appointment will be given for the next study site visit.
  - Remind patient to be in fasting conditions (i.e. overnight, at least 10-12 hours fast and refrain from smoking) for next study site visit, if applicable. Also, alcohol consumption within 48 hours and intense physical exercise within 24 hours preceding the next study site visit are discouraged.
  - Remind patient to bring the diary at the next study site visit.

10.1.4.1.2 Visit 4 (Month 1, ± 7 days)

This visit will include:

- Record of concomitant medication.

- Vital signs: SBP and DBP, heart rate.
- Collection of adverse events.
- Data collection on IMP administration and IMP compliance checked by review of diary.
- Check of the compliance and stability of the dose of the LMT (atorvastatin, rosuvastatin, ± other LMT).
- Blood sample for:
  - Lipids: TC, LDL-C, HDL-C, TG, non-HDL-C, ApoB.
  - Liver panel (ALT, AST, ALP, and total bilirubin).
  - CPK.
- The patient diary should be given and instructions on its completion should be reviewed.
- Reminders:
  - An appointment will be given for the next study site visit.
  - Remind patient to be in fasting conditions (i.e. overnight, at least 10-12 hours fast and refrain from smoking) for next study site visit, if applicable. Also, alcohol consumption within 48 hours and intense physical exercise within 24 hours preceding the next study site visit are discouraged.
  - Remind patient to bring the diary, and used and unused kits at the next study site visit.

10.1.4.1.3 Visit 5 (Month 2, ± 7 days)

This visit will include:

- Record of concomitant medication.
- Vital signs: SBP and DBP, heart rate
- Collection of adverse events.
- Data collection on IMP administration and IMP compliance check by review of diary and treatment kit accountability (used and unused kits).
- Check of the compliance and stability of the dose of the LMT (atorvastatin, rosuvastatin, ± other LMT).
- Blood sample for:
  - Lipids: TC, LDL-C, HDL-C, TG, non-HDL-C, Apo B.
  - Anti-SAR236553 antibodies.
- EQ-5D patient questionnaire.
- Double blind IMP kit dispensation as per treatment kit number provided by IVRS along with schedule reminder and the treatment administration package should be provided. The patient injection instruction manual and diary may be given, as needed.
- Reminders:

- An appointment will be given for the next study site visit.
- Remind patient to be in fasting conditions (i.e. overnight, at least 10-12 hours fast and refrain from smoking) for next study site visit, if applicable. Also, alcohol consumption within 48 hours and intense physical exercise within 24 hours preceding the next study site visit are discouraged.
- Remind patient to bring the diary, and used and unused kits at the next study site visit.

10.1.4.1.4 Visit 6 (Month 4, ± 14 days)

This visit will include:

- Record of concomitant medication.
- Body weight measurement.
- Vital signs: SBP and DBP, heart rate
- Collection of adverse events.
- Data collection on IMP administration and IMP compliance check by review of diary and treatment kit accountability (used and unused kits).
- Check of the compliance and stability of the dose of the LMT (atorvastatin, rosuvastatin, ± other LMT).
- Urinalysis.
- Urine pregnancy test (females of childbearing potential only).
- Blood sample for:
  - Lipids: TC, LDL-C, HDL-C, TG, non-HDL-C, ApoB, ApoA-1, ratio ApoB/ApoA-1, and Lp (a)
  - Liver panel (ALT, AST, ALP, and total bilirubin).
  - CPK.
  - hs-CRP.
  - Library samples.
  - Anti-SAR236553 antibodies.
- EQ-5D patient questionnaire.
- Double blind IMP kit dispensation as per treatment kit number provided by IVRS along with schedule reminder and the treatment administration package should be provided. The patient injection instruction manual and diary may be given, as needed.
- Reminders:
  - An appointment will be given for the next study site visit.
  - Remind patient to bring the diary, and used and unused kits at the next study site visit.

10.1.4.1.5 Visit 8 (Month 8, ± 14 days)

This visit will include:

- Record of concomitant medication.
- Body weight measurement.
- Vital signs: SBP and DBP, heart rate
- Collection of adverse events.
- Data collection on IMP administration and IMP compliance check by review of diary and treatment kit accountability (used and unused kits).
- Check of the compliance and stability of the dose of the LMT (atorvastatin, rosuvastatin, ± other LMT).
- EQ-5D patient questionnaire.
- Double blind IMP kit dispensation as per treatment kit number provided by IVRS along with schedule reminder and the treatment administration package should be provided. The patient injection instruction manual and diary may be given, as needed.
- Reminders:
  - An appointment will be given for the next study site visit.
  - Remind patient to be in fasting conditions (i.e., overnight, at least 10-12 hours fast and refrain from smoking) for next study site visit, if applicable. Also, alcohol consumption within 48 hours and intense physical exercise within 24 hours preceding the next study site visit are discouraged.
  - Remind patient to bring the diary, and used and unused kits at the next study site visit.

10.1.4.1.6 Visit 10 (Month 12, ± 14 days)

This visit will include:

- Record of concomitant medication.
- Body weight measurement.
- Physical examination including vital signs: SBP and DBP, heart rate.
- Collection of adverse events.
- Data collection on IMP administration and IMP compliance check by review of diary and treatment kit accountability (used and unused kits).
- Check of the compliance and stability of the dose of the LMT (atorvastatin, rosuvastatin, ± other LMT).
- Urinalysis.
- Urine pregnancy test (females of childbearing potential only).
- Blood sample for:

- Lipids: TC, LDL-C, HDL-C, TG, non-HDL-C, ApoB, ApoA-1, ratio ApoB/ApoA-1, and Lp (a).
- Hematology: blood cell count including hematocrit, hemoglobin, red blood cell count, white blood cell count with differential count and platelets.
- Chemistry: glucose, sodium, potassium, chloride, bicarbonate, calcium, phosphorous, urea nitrogen, creatinine, uric acid, LDH, total protein, albumin, and γGT.
- Liver panel (ALT, AST, ALP, and total bilirubin).
- HbA_{1c}.
- CPK.
- hs-CRP.
- Library samples.
- Anti-SAR236553 antibodies.

- EQ-5D patient questionnaire.
- Double-blind IMP kit dispensation as per treatment kit number provided by IVRS along with schedule reminder. The patient injection instruction manual and treatment administration package should be provided. The patient diary should be given and instructions on its completion should be reviewed.
- Reminders:
  - An appointment will be given for the next study site visit.
  - Remind patient to be in fasting conditions (i.e. overnight, at least 10-12 hours fast and refrain from smoking) for next study site visit, if applicable. Also, alcohol consumption within 48 hours and intense physical exercise within 24 hours preceding the next study site visit are discouraged.
  - Remind patient to bring the diary, and used and unused kits at the next study site visit.

10.1.4.1.7 Visit 12 (Month 16)/ Visit 14 (Month 20)/ Visit 18 (Month 30)/ Visit 22 (Month 42)/ Visit 26 (Month 54) (± 14 days)

These visits will include:

- Record of concomitant medication.
- Body weight measurement.
- Vital signs: SBP and DBP, heart rate.
- Collection of adverse events.
- Data collection on IMP administration and IMP compliance check by review of diary and treatment kit accountability (used and unused kits).
- Check of the compliance and stability of the dose of the LMT (atorvastatin, rosuvastatin, ± other LMT).

- Urine pregnancy test (females of childbearing potential only).
- Blood sample for:
  - Lipids: TC, LDL-C, HDL-C, TG, non-HDL-C, ApoB.
  - Liver panel (ALT, AST, ALP, and total bilirubin).
  - CPK.
- EQ-5D patient questionnaire.
- Double-blind IMP kit dispensation as per treatment kit number provided by IVRS along with schedule reminder. The patient injection instruction manual and treatment administration package should be provided. The patient diary should be given and instructions on its completion should be reviewed.
- Reminders:
  - An appointment will be given for the next study site visit.
  - Remind patient to be in fasting conditions (i.e., overnight, at least 10-12 hours fast and refrain from smoking) for next study site visit, if applicable. Also, alcohol consumption within 48 hours and intense physical exercise within 24 hours preceding the next study site visit are discouraged.
  - Remind patient to bring the diary, and used and unused kits at the next study site visit.

10.1.4.1.8 Visit 16 (Month 24)/ Visit 20 (Month 36)/ Visit 24 (Month 48)/ Visit 28 (Month 60) (± 14 days)

These visits will include:

- Record of concomitant medication.
- Body weight measurement.
- Physical examination including vital signs: SBP and DBP, heart rate.
- Collection of adverse events.
- Data collection on IMP administration and IMP compliance check by review of diary and treatment kit accountability (used and unused kits).
- Check of the compliance and stability of the dose of the LMT (atorvastatin, rosuvastatin, ± other LMT).
- Urinalysis.
- Urine pregnancy test (females of childbearing potential only).
- Blood sample for:
  - Lipids: TC, LDL-C, HDL-C, TG, non-HDL-C, ApoB.
  - Hematology: blood cell count including hematocrit, hemoglobin, red blood cell count, white blood cell count with differential count and platelets.

- Chemistry: glucose, sodium, potassium, chloride, bicarbonate, calcium, phosphorous, urea nitrogen, creatinine, uric acid, LDH, total protein, albumin, and γGT.
- Liver panel (ALT, AST, ALP, and total bilirubin).
- HbA_{1c}.
- CPK.
- Anti-SAR236553 antibodies.

- EQ-5D patient questionnaire.
- Double-blind IMP kit dispensation as per treatment kit number provided by IVRS along with schedule reminder. The patient injection instruction manual and treatment administration package should be provided. The patient diary should be given and instructions on its completion should be reviewed.
- Reminders:
  - An appointment will be given for the next study site visit.
  - Remind patient to be in fasting conditions (i.e., overnight, at least 10-12 hours fast and refrain from smoking) for next study site visit, if applicable. Also, alcohol consumption within 48 hours and intense physical exercise within 24 hours preceding the next study site visit are discouraged.
  - Remind patient to bring the diary, and used and unused kits at the next study site visit.

10.1.4.2 Contacts (phone calls or contacts via internet) from randomization

Contacts should be scheduled in between on-sites visits as follows: Visit 7 (Month 6)/ Visit 9 (Month 10)/ Visit 11 (Month 14)/ Visit 13 (Month 18)/ Visit 15 (Month 22)/ Visit 17 (Month 27)/ Visit 19 (Month 33)/ Visit 21 (Month 39)/ Visit 23 (Month 45)/ Visit 25 (Month 51)/ Visit 27 (Month 57)/ Visit 29 (Month62). The timing described above in Month is only indicative, flexibility will be allowed for performing the phone calls in between on-site visits.

These contacts will conform with privacy regulations at each site.

This contact will include:

- Record of concomitant medication.
- Collection of information on IMP administration.
- Collection of information on possible occurrence of efficacy endpoints.
- Reminders: as applicable for IMP administration schedule, timing of next visit, fasting conditions for next lab assessment, to bring the diary and used and unused kits at the next study site visit.

10.1.4.3 Early end of treatment visit – Visit 70

For patients who will have prematurely discontinued IMP, an end of treatment visit called Visit 70 (early end of treatment visit) will be performed as soon as possible after discontinuation. Assessments done at this visit will be similar to those planned for the study completers at the final visit (Visit 30, Month 64) (see Section 10.1.4.4.1). Then those patients will be strongly encouraged to complete all the remaining study visits and procedures as originally scheduled, as described in Section 1.2, until the common study end date (i.e., final visit, Visit 30, Month 64) with the exception of study treatment administration and its associated procedures. Patients who will not be able to attend any particular study visit will be invited to attend a subsequent visit.

Finally, the Investigator will make every effort to contact participants who are lost to follow-up. Attempts to contact such participants must be documented in the participant's records.

10.1.4.4 Common study end date – Final Visit – Visit 30 (Month 64, ± 14 days)

This visit is scheduled to be performed when the last surviving randomized patient has been followed for a minimum of 24 months or the target number of events (1613) is reached, whichever comes last; assuming a recruitment period of 40 months and therefore a 64-month follow-up for the first randomized patient. This final visit (Visit 30, Month 64) should be performed for all patients regardless of the patient status (prematurely discontinued or completer) and determine the common study end date. Thus, for patients not attending a scheduled visit at this time, final visit (Visit 30, Month 64) may occur up to 30 days after the announcement of the end of the study.

The patient should return to the investigational site in the morning in fasting condition (i.e. overnight, at least 10-12 hours fast and refrain from smoking) with the used and unused kits and the diary.

Depending on the status of patients, either study completers or prematurely discontinued, assessments that have to be performed will be different:

- For patients who will be still on treatment, this final on-site visit (Visit 30, Month 64) will correspond to the end of treatment visit. Patients will be instructed to stop the IMP injections 2 weeks prior to this final visit. Assessments to be performed are described in Section 10.1.4.4.1.
- For who will have prematurely discontinued IMP, this final on-site visit (Visit 30, Month 64) will correspond to the end of study visit. Assessments to be performed are described in Section 10.1.4.4.2.

The patient should return to the investigational site in the morning in fasting condition (i.e. overnight, at least 10-12 hours fast and refrain from smoking) with the used and unused kits and the diary.

10.1.4.4.1 Final Visit - Visit 30 for study completers

This visit will include:

- Record of concomitant medication.
- Body weight measurement.
- Physical examination including vital signs: SBP and DBP, heart rate.
- Collection of adverse events.
- Data collection on IMP administration and IMP compliance check by review of diary and treatment kit accountability (used and unused kits).
- Check of the compliance and stability of the dose of the LMT (atorvastatin, rosuvastatin, ± other LMT).
- 12-lead ECG.
- Urinalysis.
- Urine pregnancy test (females of childbearing potential only).
- Blood sample for:
  - Lipids: TC, LDL-C, HDL-C, TG, non-HDL-C, ApoB, ApoA-1, ratio ApoB/ApoA-1, and Lp (a).
  - Hematology: blood cell count including hematocrit, hemoglobin, red blood cell count, white blood cell count with differential count and platelets.
  - Chemistry: glucose, sodium, potassium, chloride, bicarbonate, calcium, phosphorous, urea nitrogen, creatinine, uric acid, LDH, total protein, albumin, and γGT.
  - Liver panel (ALT, AST, ALP, and total bilirubin).
  - HbA_{1c}.
  - CPK.
  - hs-CRP.
  - Hepatitis C antibody test.
  - Library samples.
  - Anti-SAR236553 antibodies.
- EQ-5D patient questionnaire.
- IVRS/IWRS contact for notification of the date of this final visit; for patients still on treatment this will be the end of treatment visit.

For these patients who will be still on treatment at this visit, an appointment for a phone call visit 2 months later will be given. For those patients, instructions will be given to contact the site in case of occurrence of adverse event, and in this case to return to the site as deemed appropriate.

10.1.4.4.2 Final Visit - Visit 30 for prematurely discontinued patients

This visit will include:

- Record of concomitant medication.

- Vital signs: SBP and DBP, heart rate.
- Collection of adverse events.
- Check of the compliance and stability of the dose of the LMT (atorvastatin, rosuvastatin, ± other LMT).
- Blood sample for
  - Anti-SAR236553 antibodies.
- IVRS/IWRS contact for notification of the date of this final visit.

10.1.4.5 Post-treatment Follow-up phone call

This visit is a phone call visit to be held 8 weeks (± 14 day) after the final visit (Visit 30, Month 64) only for patients still on treatment at the time of this visit. The patient will be called by the Investigator at a certain, previously agreed time point.

This phone call will include:

- Record of concomitant medication.
- Collection of adverse events.

In case of an adverse event the patient will be asked to come to the site, if necessary.

- IVRS/IWRS contact for notification of the date of the post-treatment follow-up phone call.

10.2 DEFINITION OF SOURCE DATA

Evaluations that are reported in the e-CRF must be supported by appropriately signed identified source documentation related but not limited to the following:

- Agreement, date, and signature of informed consent mentioning the study identification.
- Patient identification, last participation in a clinical trial, medical history, associated diseases, and data related to the studied pathology.
- Contraception methods for women of childbearing potential.
- Previous and concomitant medication (including the lipid modifying therapy).
- Study identification.
- Treatment number, dates of administration.
- Dates of visits and assessments including the examination report.
- Vital signs, height, body weight.
- Faxed central lab reports (dated and signed by the Principal Investigator or Sub-Investigator).

- IVRS/IWRS confirmation fax (screening, screen failure, training kit allocation, randomization, treatment reallocation, discontinuation, end of double blind treatment period, end of study, unblinding if applicable).
- ECG records signed and dated.
- Adverse events and follow-up:
- In case of SAE, the site should file in the source document at least copies of the hospitalization reports and any relevant examination reports documenting the follow up of the SAE.
- Date of premature study discontinuation (if any) and reason.

Source documentation may be found in the following:

- Patient's identity.
- Medical history.
- Hospital records.
- Nursing notes.
- Physician's notes.

10.3 HANDLING OF PATIENT TEMPORARY OR PERMANENT TREATMENT DISCONTINUATION AND OF PATIENT STUDY DISCONTINUATION

The IMP should be continued whenever possible. In case the IMP is stopped, it should be determined if the stop can be made temporarily; permanent IMP discontinuation should be a last resort. Any IMP discontinuation should be fully documented in the e-CRF and source notes. In any case, the patient should remain in the study as long as possible.

Pregnancy will lead to definitive treatment discontinuation in all cases. Stopping rules described in Appendix C should be applied.

10.3.1 Temporary treatment discontinuation with investigational medicinal product(s)

Temporary treatment discontinuation (also referred to as treatment interruption) may be considered by the Investigator because of suspected AEs. Reinitiating of treatment with the IMP will be done under close and appropriate clinical and/or laboratory monitoring once the Investigator will have considered according to his/her best medical judgment that the responsibility of the IMP in the occurrence of the concerned event was unlikely and if the selection criteria for the study are still met (refer to Section 7.1 and Section 7.2).

All treatment interruption duration should be recorded by the Investigator in the appropriate e-CRF screens when considered as confirmed. Temporary treatment interruption is defined as an interruption of scheduled injections for no more than 2 months (i.e., no more than 4 consecutive injections missed), otherwise the patient should be considered as permanently discontinued from treatment.

Treatment interruption is defined as one or more scheduled injections that are not administered to the patient as decided by the Investigator.

10.3.2 Permanent treatment discontinuation with investigational medicinal product(s)

Permanent treatment discontinuation (also referred to as treatment discontinuation) is any treatment discontinuation associated with the definitive decision from the Investigator or the patient not to re-expose the patient to the IMP at any time.

10.3.3 List of criteria for permanent treatment discontinuation

The patients may withdraw from treatment with the IMP if they decide to do so, at any time and irrespective of the reason, or this may be the Investigator's decision. All efforts should be made to document the reasons for treatment discontinuation and this should be documented in the e-CRF.

Patients should discontinue the Investigational Medicinal Product (IMP) for the following reasons:

- Pregnancy, intention for pregnancy, or no longer with effective contraceptive method of birth control (females only).
- Acute injection reaction of clinical concern.
- Serious adverse event (or non-serious but severe in intensity) of hypersensitivity reaction considered related to IMP.
- At patient request.
- If, in the Investigator's opinion, continuation with the administration of the IMP would be detrimental to the patient's well-being.
- Intercurrent condition that requires discontinuation of the IMP (e.g. laboratory abnormalities, please refer to decision tree Appendix C).
- At the specific request of the Sponsor.
- Any code breaking requested by the Investigator.
- Patient receives double-blind treatment prior to randomization.

Patient withdrawal from the study treatment or study should be avoided as much as possible. If this occurs, every attempt should be made to restart study drug if medically appropriate, whatever the duration of discontinuation. In case of permanent study treatment discontinuation, the appropriate follow-up until the common study end date should still be continued.

All efforts should be made to document the reasons for treatment discontinuation and this should be documented in the e-CRF.

10.3.4 Handling of patients after permanent treatment discontinuation

Patients who prematurely discontinue study treatment (regardless of the reason) must be maintained in the study and will be strongly encouraged to complete all the remaining study visits and procedures as originally scheduled, as described in Section 1.2, until the common study end date with the exception of study treatment administration and its associated procedures. At the time of the common study end date, visit V30 (Month 64) should be performed. Patients who will not be able to attend any particular study visit will be invited to attend a subsequent visit

At the time of treatment discontinuation, and as soon as possible, the Visit 70 (early end of treatment visit) will be filled in and the patients will be assessed using the procedure normally planned for the last dosing day with the IP (visit similar to final visit (Visit 30, Month 64).

In case of written withdrawal of consent (WOC) for follow-up visits, and unless otherwise stated by the patient in the informed consent form, Investigators will be encouraged to get information from the general practitioner, any other physician, or other medical-care provider, in order to follow the medical status of the patients (especially when they withdraw their consent after having experienced an AE/SAE or a cardiovascular event [efficacy endpoint]). Investigators will also be expected to try as much as possible to re-contact those patients at the end of the trial, in order to obtain at least their vital status (dead or alive), as well as their CV status if possible, and thus avoid lost to follow-up for the efficacy assessment.

If the patient exercises his right of opposition to transmission of the data to the sponsor or removal of data from the database, the investigator will inform, in writing, the clinical trial sponsor, and the sponsor will decide how to handle the subject data and samples based on local regulations and data privacy requirements.

All definitive discontinuation of study treatment should be recorded by the Investigator in the appropriate screens of the e-CRF and in the patient’s medical records when considered as confirmed. IVRS/IWRS should be notified when a patient prematurely discontinues study treatment.

10.3.5 Procedure and consequence for patient withdrawal from study

The patients may withdraw from the study, before study completion if they decide to do so, at any time and irrespective of the reason or this may be the Investigator’s decision:

- All study withdrawals should be recorded by the Investigator in the appropriate screens of the e-CRF and in the patient’s medical records when considered as confirmed (at least date of withdrawal and reason for). IVRS/IWRS should be notified when a patient prematurely discontinues study.
- The patients should be assessed using the procedure normally planned for the visit V70 which corresponds to the early end of treatment visit. (see Section 10.3.4).

However all randomized patients must be followed up according to the study procedures as specified in this protocol up to the scheduled date of study completion or death, regardless of

whether they discontinued the study treatment prematurely or not. Any event occurring after early study treatment discontinuation will be recorded up through the common study end date.

In case of study treatment discontinuation (temporary or permanent) due to an adverse event, such patients will be closely monitored until the resolution or stabilization of this adverse event. This may mean that follow-up will continue after the patients have completed the study.

For patients who fail to return to the site, the Investigator should make the best effort to re-contact the patient (e.g., contacting patient's family or private physician, review available registries or health care database), and to determine his/her health status, including at least his/her vital status dead or alive at minimum, preferably also stroke or MI status). Attempts to contact such patients must be documented in the patient's records (e.g., times and dates of attempted telephone contact, receipt for sending a registered letter). This will be clearly stated in the informed consent form.

For patients considered lost to follow-up, the e-CRF must be completed up to the last visit performed. The statistical analysis plan will provide the details concerning the analysis of these patients.

Patients who have withdrawn from the study cannot be re-randomized (treated) in the study. Their inclusion and treatment number must not be reused.

10.4 OBLIGATION OF THE INVESTIGATOR REGARDING SAFETY REPORTING

10.4.1 Definitions of adverse events

Please refer to Appendix E for Adverse Event (AE) reporting requirements.

10.4.1.1 Adverse event

An **adverse event** (AE) is any untoward medical occurrence in a patient or clinical investigation patient administered a pharmaceutical product and which does not necessarily have to have a causal relationship with this treatment.

10.4.1.2 Serious adverse event

A **serious adverse event** (SAE) is any untoward medical occurrence that at any dose:

- Results in death, or
- Is life-threatening, or
  Note: The term "life-threatening" in the definition of "serious" refers to an event in which the patient was at risk of death at the time of the event; it does not refer to an event which hypothetically might have caused death if it were more severe.
- Requires inpatient hospitalization or prolongation of existing hospitalization, or
- Results in persistent or significant disability/incapacity, or
- Is a congenital anomaly/birth defect

- Is a medically important event
  Medical and scientific judgment should be exercised in deciding whether expedited reporting is appropriate in other situations, such as important medical events that may not be immediately life-threatening or result in death or hospitalization but may jeopardize the patient or may require intervention (ie, specific measures or corrective treatment) to prevent one of the other outcomes listed in the definition above.

  Note: The following list of medically important events is intended to serve as a guideline for determining which condition has to be considered as a medically important event. The list is not intended to be exhaustive:

  - Intensive treatment in an emergency room or at home for:
    - Allergic bronchospasm
    - Blood dyscrasias (ie, agranulocytosis, aplastic anemia, bone marrow aplasia, myelodysplasia, pancytopenia, etc),
    - Convulsions (seizures, epilepsy, epileptic fit, absence, etc).
  - Development of drug dependency or drug abuse
  - ALT >3 x ULN + total bilirubin >2 x ULN or asymptomatic ALT increase >10 x ULN
  - Suicide attempt or any event suggestive of suicidality
  - Syncope, loss of consciousness (except if documented as a consequence of blood sampling)
  - Bullous cutaneous eruptions
  - Cancers diagnosed during the study or aggravated during the study.
  - Chronic neurodegenerative diseases (newly diagnosed) or aggravated during the study.

10.4.2 Adverse event of special interest

Adverse Events of Special Interest (AESI) are AEs (serious or non-serious) that need to be monitored, documented, and managed in a pre-specified manner described in the protocol. Please see Section 10.4.6.2 and Appendix E for additional information.

10.4.3 Serious adverse events waived from expedited regulatory reporting to health authorities

Unlike most studies where the primary efficacy variable is the resolution or improvement of an existing condition, in this study efficacy outcomes include the occurrence of life-threatening events. Indeed, participants to this study are recruited precisely because they are at high risk for these life threatening events. They are therefore expected to have at least one cardiovascular efficacy endpoint during the course of the study.

In light of the above, it is important that the cardiovascular efficacy endpoints specified in this study (primary and secondary efficacy endpoints) are waived from expedited reporting to Health

Authorities providing an agreement has been reached with them. Those SAEs that are disease related with reference to the product IB will be considered expected.

Cardiovascular efficacy endpoints will be therefore reported (within 1 working day) in the specific outcome event screens of the e-CRF; the system will automatically send the notification to the Monitoring Team and the company responsible for the data coordination process for these events (DCRI) after approval of the Investigator within the e-CRF or after a standard delay.

However expedited reporting to Health Authorities for the following cardiovascular outcomes will be waived:

- CV death including CHD death,
- All other causes of death
- Non-fatal MI.
- Non-fatal stroke.
- Unstable angina requiring hospitalization.
- Hospitalization for unanticipated coronary revascularization procedure.
- Congestive heart failure requiring hospitalization.

10.4.4 General guidelines for reporting adverse events

- All AEs, regardless of seriousness or relationship to IMP/NIMP, spanning from the signature of the informed consent form until the end of the study as defined by the protocol for that patient, are to be recorded on the corresponding screen(s) included in the e-CRF.
- When a safety event is categorized as a primary outcome, the event will be reported as an AE but will be waived from reporting to health authorities providing an agreement has been reached with them (see Section 10.4.3).
- Whenever possible, diagnosis or single syndrome should be reported instead of symptoms. The Investigator should specify the date of onset, intensity, action taken with respect to IMP, corrective treatment/therapy given, additional investigations performed, outcome, and his/her opinion as to whether there is a reasonable possibility that the AE was caused by the IMP.
- When treatment is prematurely discontinued, the patient will be maintained in the study and the patient's observations will continue until the common study end date as defined by the protocol for that patient.
- Laboratory, vital signs or ECG abnormalities are to be recorded as AEs only if:
    - Symptomatic and/or
    - Requiring either corrective treatment or consultation, and/or
    - Leading to IMP discontinuation or modification of dosing, and/or
    - Considered as clinically relevant (such as for ECG a prolonged QTcB > 500 ms or an increase in QTcB of > 60 msec compared to baseline), and/or
    - Fulfilling a seriousness criterion, and/or
    - Defined as an AE of special interest with immediate notification.

See Appendix E for a summary of AE reporting guidelines.

10.4.5 Instructions for reporting serious adverse events

In the case of occurrence of an SAE, the Investigator must immediately:

- ENTER (within 1 working day) the information related to the SAE in the appropriate screens of the e-CRF; the system will automatically send a notification to the monitoring team after approval of the Investigator within the e-CRF or after a standard delay.
- SEND (preferably by fax or e-mail) a photocopy of all examinations carried out and the dates on which these examinations were performed, to the representative of the monitoring team whose name, fax number, and email address appear on the clinical trial protocol. Care should be taken to ensure that the patient's identity is protected and the patient's identifiers in the clinical trial are properly mentioned on any copy of a source document provided to the Sponsor. For laboratory results, include the laboratory normal ranges.
- All further data updates should be recorded in the e-CRF as appropriate, and further documentation as well as additional information (for laboratory data, concomitant medications, patient status, etc) should be sent (by fax or e-mail) to the monitoring team within 1 working day of knowledge of the SAE. In addition, every effort should be made to further document any SAE that is fatal or life threatening within a week (7 days) of the initial notification.
- A back-up plan (using a paper CRF process) is available and should be used when the e-CRF system does not work.

10.4.6 Guidelines for reporting adverse events of special interest

10.4.6.1 Reporting of adverse events of special interest with immediate notification

For these AEs, the Sponsor will be informed immediately (i.e. within 1 working day), as per SAEs notification described in Section 10.4.5, even if not fulfilling a seriousness criterion, using the corresponding screens in the e-CRF.

- ALT ≥ 3 ULN (if baseline ALT < ULN) Or ALT ≥ 2 times the baseline value (if baseline ALT ≥ ULN) (Please refer to related flowchart in Appendix C).
- Allergic events
  - Allergic drug reactions and/or local injection site reactions deemed to be allergic (or have an allergic component) that require consultation with another physician for further evaluation of hypersensitivity/allergy, as per the investigator's medical judgment or as per Section 10.6.2, should be reported as an AESI with immediate notification.
  - All allergic events, and all injection site reactions having an allergic component or deemed to be allergic, require completion of the specific e-CRF screen (see Section 10.6.2), regardless of requirements for immediate reporting.
- Hemolytic anemia (see Section 10.4.7.1 and Appendix D)

  - If there is a decrease in hemoglobin and reflexive testing as per Appendix D suggesting hemolysis, then report this as an AESI with immediate notification. Special e-CRF screen will need to be completed.

- Pregnancy
  - Pregnancy occurring in a female patient included in the clinical trial. Pregnancy will be recorded as a pre-specified AE with immediate notification in all cases. It will be qualified as an SAE only if it fulfills the SAE criteria.
  - In the event of pregnancy, IMP should be discontinued.
  - The follow-up of the pregnancy will be mandatory until the outcome has been determined.
- Symptomatic Overdose with IMP
  - An overdose (accidental or intentional) is an event suspected by the Investigator or spontaneously notified by the patient (not based on systematic injection counts) and defined as at least twice of the intended dose within the intended therapeutic interval (i.e., 2 or more injections from the double-blind treatment kit are administered in <7 calendar days); to be reported using the corresponding screens in the e-CRF using the Term “symptomatic OVERDOSE (accidental [or intentional])”. The patient should be monitored and appropriate symptomatic treatment instituted.
  - The circumstances of the overdose should be clearly specified in the verbatim.

10.4.6.2 Reporting of adverse events of special interest without immediate notification

See Appendix E.

For these AEs, the Sponsor does not have to be informed immediately, unless meeting seriousness criterion.

- Asymptomatic overdose with IMP
  - An overdose (accidental or intentional) is an event suspected by the Investigator or spontaneously notified by the patient (not based on systematic injection counts) and defined as at least twice of the intended dose within the intended therapeutic interval (ie, 2 or more injections from the double-blind treatment kit are administered in <7 calendar days); to be reported using the corresponding screens in the e-CRF using the Term “asymptomatic OVERDOSE (accidental [or intentional])” The patient should be monitored for any AEs and treated, as needed.
- Local injection site reactions (see Section 10.6.1)
  - Local injection site reactions that are considered as non-allergic events should be further characterized by evaluating the related symptoms that comprise an injection site reaction such as but not limited to redness, pain, etc (See Appendix F). Special e-CRF screens will need to be completed. If such an AE was to occur, then do not report the individual components of the reaction but rather the term “local injection site reaction”, the individual components being described in the specific e-CRF screen.

- Allergic events not referred for consultation with another physician (see Section 10.4.6.1)
  - All allergic events will need to have allergy specific e-CRF screens completed (see Section 10.6.2), regardless of requirements for immediate reporting

10.4.7 Guidelines for management of specific laboratory abnormalities

Laboratory abnormalities with pre-specified monitoring should be monitored, documented, and managed according to the related flowchart in protocol Appendix C and Appendix D:

- Neutropenia.
- Thrombocytopenia.
- Increase in ALT.
- Acute renal insufficiency.
- Decrease in hemoglobin (defined as ≥ 1.5 g/dL).
- Increase in CPK and suspicion of rhabdomyolysis.

10.4.7.1 Hemoglobin decrease

See Appendix D.

At the first post-randomization occurrence of a hemoglobin (Hb) measurement decrease by ≥ 1.5 g/dL as compared to the randomization visit hemoglobin measurement, then the Central Lab will reflexively measure reticulocyte count and haptoglobin using specimens already obtained at the same time point for which the hemoglobin decrease was detected. The Central Lab will then provide the results of the reticulocyte count and haptoglobin (including LDH and indirect bilirubin [reflexively measured only if the total bilirubin > ULN]) to the investigator.

- If the following pattern of abnormalities is noted:
  - Reticulocyte count > Central Lab's upper limit of the reference range (also referred to as ULN) **AND**
  - Haptoglobin < Central Lab's lower limit of the reference range (also referred to as LLN) **AND**
  - LDH > ULN **AND**
  - Indirect bilirubin > ULN (only if the total bilirubin > ULN).

  The patient should be referred to a hematologist. The hematologist should obtain a peripheral blood smear and anti-erythrocyte antibodies (direct and indirect) by Coombs test. Further investigations are at the discretion of the hematologist.
- If the results are normal or the pattern of abnormality is something other than that described above, then the Investigator should exercise his/her medical judgment in the interpretation of the results, necessity for workup of the decrease in hemoglobin or referral to a hematologist.

If a second hemoglobin measurement demonstrating a further decrease of ≥ 1 g/dL from the last available value is observed, even if the previous work-up was negative, the same investigations can be repeated and a hematology consultation can be requested at the discretion of the Investigator or at the Sponsor's request.

10.5 OBLIGATIONS OF THE SPONSOR

During the course of the study, the Sponsor will report in an expedited manner all SAEs that are both unexpected and at least reasonably related to the IMP (Suspected Unexpected Serious Adverse Reaction; SUSAR), to the Health Authorities, IECs/IRBs as appropriate and to the Investigators.

In addition, the Sponsor will report in an expedited manner all SAEs that are expected and at least reasonably related to the IMP to the Health Authorities, according to local regulations.

In this study, cardiovascular efficacy endpoints specified (primary and secondary efficacy endpoints) are waived from expedited reporting to Health Authorities providing an agreement has been reached with them.

Also some other AEs may be considered related to the underlying condition and thus will not be considered unexpected as given in the Investigator's Brochure.

Any other AE not listed as an expected event in the Investigator's Brochure or in this protocol will be considered as unexpected.

The Sponsor will report all safety observations made during the conduct of the trial in the clinical study report (CSR).

10.6 SAFETY INSTRUCTIONS

10.6.1 Local tolerability (Local Injection Site Reactions)

In case the Investigator or the patient recognizes any signs of local intolerability, then this should be treated and followed up as per the Investigator's medical judgment. See Section 10.4.6.2 and Appendix F for further information.

10.6.2 Allergic adverse events (See Sections 10.4.6.1 and 10.4.6.2)

Specific eCRF screens are to be filled in to assess allergic reactions or allergic-like reactions that may occur during the clinical studies conducted with SAR236553.

Sometimes transient injection site reactions, irritant in nature, may occur, requiring no intervention and being of dubious significance. These reactions would not be considered to be allergic reactions.

Adverse events that may constitute an allergic reaction (e.g., generalized itch, nasal itch, swelling at injection site, flushing, hives, swelling at lips, eyes, face, tongue, hands, feet, lump in throat, difficulty to swallow, hoarseness, change in pitch of voice, incapacity to speak, wheezing, chest tightness, stridor, etc) should be considered to be reported on the General Allergic Reaction and/or Local Injection Site Reaction Complementary Form.

Adverse events that are obviously not of allergic origin (e.g., local injection site reactions) should only be recorded on the Local Injection Site Reaction Complementary Form. However, injection site reactions which progress/expand/worsen/etc should be evaluated as recommended in Section 10.6.2.1 and General Allergic Reaction Complementary form should be completed.

The IMP should be immediately interrupted (temporarily discontinued) if there is a suspicion of an allergic event related to IMP. See Section 10.3.1 for further information on treatment interruption and Section 10.3.2 for criteria for permanent treatment discontinuation.

10.6.2.1 Allergic adverse event with cutaneous involvement

Adverse events with cutaneous involvement which are obviously of allergic origin or injection site reactions which progress/expand/worsen/etc should be evaluated by a dermatologist as soon as possible, and preferably within one week of the site first becoming aware of the event.

The investigator should evaluate the patient for possible etiologies (new medications, etc) and extra-cutaneous symptoms and signs. An unscheduled Central Laboratory assessment for hematology, chemistry, liver panel, PK, and ADA should be obtained. If it is possible, the site will take pictures of the skin lesions in order to provide the patient with them for the dermatologist's visit. If the photos are obtained, then copies should be kept as source documents which may later be collected by the sponsor. The investigator will provide a summary of the patient's case, reason for consultation, and information being requested to the consulting dermatologist.

A full consultation report should be sent by the dermatologist to the investigator. The full report should contain, at a minimum, the following information; a detailed description of the rash (such as the morphology [lesion type], shape of individual lesions, arrangement of multiple lesions [eg, scattered, grouped, linear, etc], distribution, color, consistency, presence of pruritus or pain, and other clinical signs) and in case a skin biopsy (including histopathology and immunofluorescence) was done (if it was deemed necessary as per the dermatologist's or investigator's medical judgment), the results of this investigation with, if applicable, a specific diagnosis of the AE. The investigator will fax the full report and the corrected AE form if necessary, to the Monitoring Team Representative within one working day.

10.6.2.2 Acute allergic injection reactions (Section 10.4.6.2)

Acute allergic injection reaction (which are considered under the category of general allergic reactions) is defined as any AE that occurs during or shortly after injection of the IMP (characterized by but not limited to hypotension, bronchoconstriction, urticaria, edema, angioedema, nausea, vomiting). The investigator should ascertain that patient can be rapidly managed with emergency equipment and medication for the treatment of these potential adverse

effects (e.g., antihistamines, bronchodilators, IV saline, corticosteroids, acetaminophen, and epinephrine) for the injections at the training, and randomization visits.

Patients will be observed at the investigational site for at least 30 minutes following the injection that takes place at the randomization visit. Patients should be treated symptomatically if any AEs are observed. Patients are to remain on observation until any acute injection reaction is assessed as stable, per the Investigator's or emergency team's discretion. General Allergic Reaction and/or Local Injection Site Reaction Complementary Form will have to be completed.

10.6.3 Monitoring related to two consecutive LDL-C <25 mg/dL (0.65 mmol/L)

Patient's who achieve 2 consecutive LDL-C levels < 25 mg/dL (0.65 mmol/L) during the study will be monitored and managed as per Appendix A (any time after randomization) as the lower limit of safe and effective LDL-C lowering has not yet been established. An independent external physician(s) (also known as independent physician) will be notified by the central laboratory of 2 consecutive LDL-C <25 mg/dL (0.65 mmol/L). The independent physician will review the unmasked LDL-C values and patient safety data, in close collaboration with the dedicated member of the Phase 3a (implemented for all Phase 3 studies evaluating the efficacy and safety of SAR236553 on LDL-C). This dedicated Phase 3a DMC member will then transmit any information as necessary to the common member involved in both DMCs (CV DMC and Phase 3a DMC).

Please see Appendix A for an outline of the process.

Then at subsequent visits, specific actions can be undertaken depending on the SAR236553 dose (down-titration or discontinuation) as follows:

- On dose of 150 mg Q2W : if LDL-C < 25 mg/dL (including LDL-C < 15 mg/dL) on 2 consecutive measurements, down-titration to 75 mg Q2W will be done in a blinded manner at the next visit and for the remaining duration of the study. As described above specific monitoring will be implemented until the down-titration is done and the confirmation that LDL-C level returns to ≥ 25 mg/dL.
- On dose of 75 mg Q2W:
  - If LDL-C < 25 mg/dL but ≥ 15 mg/dL on 2 consecutive measurements: study treatment will be continued but additional monitoring will be implemented, to further confirm the safety of low LDL-C levels.
  - If LDL-C < 15 mg/dL on 2 consecutive measurements: study treatment will be discontinued at the next visit. In order to maintain the blind there will be blinded substitution to placebo injections for the remaining duration of the study.

As lipid values will not be communicated to investigators after randomization to maintain the blind, the continuation of the 75 mg dose, the up-titration to the 150 mg dose, the down-titration from 150 to 75 mg, or discontinuation will occur automatically, without site or patient awareness. Moreover, blinded dose adjustment will avoid the need for dummy titrations in the placebo group.

10.7 ADVERSE EVENTS MONITORING

All events will be managed and reported in compliance with all applicable regulations, and included in the final clinical study report.

11 STATISTICAL CONSIDERATIONS

11.1 DETERMINATION OF SAMPLE SIZE

The sample size calculations are based on the primary efficacy variable, with the following assumptions:

- Primary efficacy endpoint is the time from randomization to the date of first occurrence of one of the following Clinical Events, as determined by the CEC: CHD death, any non-fatal MI, fatal and non-fatal ischemic stroke, unstable angina requiring hospitalization. Based on PROVE-IT results (8) and considering adjustment based on the study design and endpoints definition, the following Kaplan-Meier probabilities of event in the placebo group have been considered: 3.8% at 12 months, 6.4% at 24 months, 9.0% at 36 months, 11.4% at 48 months. Probability of event at different time points are estimated using a piece-wise exponential model. The primary efficacy endpoint will be analyzed on an intent-to-treat basis (all randomized patients, including those who discontinue study medication are followed for any efficacy event until the termination of the study).
- Hazard ratio of 0.85 in test group relative to placebo (corresponding to a 15% risk reduction). Constant hazard ratio assumption is used.
- A log-rank test at a 1-sided 2.5% significance level with 90% power.
- Two interim analyses, according to a group sequential design, using for efficacy Gamma (-22) α-spending function, and for futility a Gamma (-5) β-spending function. Non-binding spending functions are used. See Section 11.5 for interim analysis details.
- 1% lost-to-follow rate at 24 months in both arms
- Enrolment rate: maximum sample size of 18000 patients enrolled in 1400 sites (these sites should be active within 12 months). The table below describes the expected enrolment rate per month; these assumptions are based on internal experience to enroll this patient population.

Table 2 - Enrolment rate assumptions per month

Month	1 to 3	4	5	6	7	8	9	10	11	12 to 40
No. pts per month	32	64	80	104	160	240	320	420	520	560
Cumulative no. patients	96	160	240	344	504	744	1064	1484	2004	2564 at M12,<br>18000 at M40

Based on the above assumptions, 1613 events are needed for 90% power. In order to achieve the 1613 targeted events, 18 000 patients (9 000 per group) will need to be randomized, over a 40 month period, with a follow-up of the last patient randomized of approximately 24 months.

Calculations were made using East 5.4 software.

11.2 DISPOSITION OF PATIENTS

Screened patients are defined as any patient who met the qualifying ACS inclusion criteria and signed the informed consent.

Randomized patients consist of all screened patients, with a double-blind treatment kit number allocated and recorded in the IVRS/IWRS database, regardless of whether the treatment kit was used or not. Patients treated without being randomized or treated with a double-blind treatment kit before the randomization will not be considered as randomized and will not be included in any analysis population. The safety experience of these patients will be reported separately, and they will not be in the safety population.

For any patient randomized more than once, only the data associated with the first randomization will be used in any analysis population. The safety experience associated with any later randomization will be assessed separately.

11.3 ANALYSIS POPULATIONS

11.3.1 Efficacy populations

11.3.1.1 Intent–to-treat population

The primary efficacy analysis population will be the intent-to-treat (ITT) population, consisting of all randomized patients as defined above. Patients in the ITT population will be analyzed according to the treatment group allocated by randomization.

11.3.2 Safety population

The Safety population considered for safety analyses will be the randomized population who did actually receive at least one dose or part of a dose of the double-blind IMP. Patients will be analyzed according to the treatment actually received (placebo or SAR236553).

In addition:

- Randomized patients for whom it is unclear whether they took the study medication will be included in the safety population as randomized.
- For patients receiving double-blind IMP from more than 1 treatment group during the trial, the treatment group allocation for as-treated analysis will be the one to which the patient was treated with the longest duration.
- In case of two consecutive LDL-C values < 15 mg/dL, placebo injections will be given to patients randomized in the SAR236553 group in order to maintain the blind. Those placebo injections will not be considered as double-blind IMP.

11.3.3 Other analysis populations

The anti-SAR236553 antibody analysis will be performed on all treated patients (safety population) with a blood sample on D1 (baseline) and at least one evaluable blood sample for antibodies post double-blind IMP injection.

Analysis populations for pharmacogenomics will be defined in a specific Statistical Analysis Plan (SAP).

11.4 STATISTICAL METHODS

11.4.1 Extent of study treatment exposure and compliance

The extent of study treatment exposure and compliance will be assessed and summarized by actual treatment received within the safety population.

Double-blind IMP injections are those administered from randomization to discontinuation of the study treatment, that is:

- Containing placebo for the ones administered to patients randomized in the placebo group
- Containing 75 or 150 mg of SAR236553

Placebo injections given to patients randomized in the SAR236553 following 2 consecutive LDL-C < 15 mg/dL will not be considered as double-blind IMP in the statistical analyses.

11.4.1.1 Extent of investigational medicinal product exposure

The total exposure will be assessed by:

- Duration of IMP exposure in months defined as: (last dose of double-blind IMP injection date +14 – first dose of double-blind IMP injection date)/30.4375, regardless of unplanned intermittent discontinuations.
- The total number of double-blind IMP injections by patient.
- Duration of observation period (months), defined as: (last contact date – first dose date+1)/30.4375. Non-integer values will be rounded to one decimal place.

The number (n) and percentage (%) of patients with an up-titration in the SAR236553 group will be described.

The number (n) and percentage (%) of patients with an up-titration followed by a down-titration in the SAR236553 group will be also described.

11.4.1.2 Compliance

Compliance will be assessed using the following parameters:

- The injection frequency will be defined for each patient as the average number of days between 2 injections, that is: (last dose date – first dose date)/(number of injections -1).
- The overall compliance will be defined for each patient as: 1-(% days with under-planned dosing + % days with above-planned dosing).Under-planned and above-planned dosing will be defined as follows, considering that injections should be performed every 2 weeks (+/- 3 days allowed time window):
  - the % days with under-planned dosing will be defined for each patient as the number of days with the previous injection administered more than 17 days before/duration of IMP exposure in days.
  - the % days with above-planned dosing will be defined for each patient as the number of days with the previous injection administered less than 11 days before/duration of IMP exposure in days.

These parameters will be summarized descriptively (N, Mean, SD, Median, Min and Max) at 6 months, by year, and on the overall study period.

Cases of overdose (i.e., 2 or more injections from the double-blind treatment kit are administered in < 7 calendar days) will be summarized by treatment group.

11.4.2 Analyses of efficacy endpoints

All efficacy analyses will be performed based on intent-to-treat (ITT) approach that included events occurring or assessments performed from randomization to the analysis cut-off date, even after the patient has discontinued the study treatment.

11.4.2.1 Analysis of primary efficacy endpoint(s)

The analysis of primary efficacy endpoint will be the comparison between the two treatments using the log-rank test procedure stratified by region (North America, South America, Western Europe, Eastern Europe, Asia, Other). As the number of events per individual country is expected to be low (about 50 countries), the analysis will be stratified according to a grouping of countries into regions.

This primary comparison is the test of the following hypotheses on the hazard ratio (HR), applying a one-sided nominal type I error of 0.0249 at the final analysis:

$$H_0: HR \geq 1 \text{ versus } H_1: HR < 1$$

The estimates of the HR and corresponding confidence interval at (1-2α)% level (α being the one-sided nominal significance level: α=0.249 at final analysis, α=0.0001 at second interim analysis) will be provided using a Cox Proportional Hazard Model stratified by region as for the log-rank test described above.

Consistency of the treatment effect across regions will be assessed.

Underlying assumptions of the Cox Proportional Hazard Model will be checked using graphical methods. If proportionality is not observed, some ad-hoc sensitivity analyses will be performed depending on the data (data-driven).

The survival curves will be estimated using Kaplan-Meier estimates: probabilities of surviving (without event) at 6 months and by year, and appropriate confidence interval will be presented by treatment arm using Kaplan-Meier estimates. Kaplan-Meier curves will be displayed by treatment arm.

Two interim analyses will be performed See Section 11.5 for description of these analyses. The cut-off dates of final and interim analyses are expected to be:

- First interim analysis (futility): when 807 patients have been experienced at least one primary efficacy event (50% fraction information)
- Second interim analysis (futility and efficacy): when 1210 patients have been experienced at least one primary efficacy event (75% fraction information)
- Final analysis: when 1613 patients have been experienced at least one primary efficacy event or for 24 months after the date of the last randomized patient, whichever comes last.

Subgroup analyses

For the primary endpoint, the treatment effects across the following subgroup factors will be examined:

- Gender;
- Age group (<65, ≥65);
- Race (Caucasian, Black, Asian/Oriental, and Other, as appropriate);
- Country (IVRS stratum, depending on the size of subgroups);
- Region (USA/Non-USA, and North America/South America/Eastern Europe/Western Europe/Asia/Rest of world);

For each parameter, a Cox proportional hazard model will be used for the overall population, including the parameter and the treatment by parameter interaction. In addition, Kaplan-Meier curves and summary statistics showing number of patients, number (%) of primary efficacy outcome events, probabilities of surviving (without event) at 6 month and by year, and appropriate confidence interval may be provided for each treatment arm in previously selected subgroups defined by the baseline characteristic/prognostic factors.

In addition, the effect of the time from ACS event to randomization (weeks) will be assessed using a Cox proportional hazard model including the time from ACS event to randomization (continuous) as a covariate, the treatment group and the interaction.

11.4.2.2 Analyses of secondary efficacy endpoints

Method for controlling the overall type-I error rate when testing the key secondary efficacy endpoints is described in Section 11.4.2.3.

Time to events secondary endpoints will be analyzed using the same statistical methodology as for the primary endpoint.

The percent change from baseline in calculated LDL-C at Month 4, at Month 24 and at the end of the study will be analyzed in the ITT population using an analysis of covariance (ANCOVA) model with treatment group and region as fixed effects, and the baseline calculated LDL-C as covariate.

Similar analyses will be performed for ApoB and non-HDL.

Based on previous experiences and published data on these endpoints, the assumptions of normality of the residuals, homogeneity of slopes and homoscedasticity underlying these models are usually valid.

Throughout the ANCOVA models, the SAR236553 group will be compared to placebo using an appropriate contrast tested at the two-sided 0.05 level, and providing the 95% confidence interval (CI) of the difference.

For patients without calculated LDL-C, ApoB, or non-HDL-C value in the time window analyzed, the percent change from baseline will not be calculated.

11.4.2.3 Multiplicity consideration

In order to handle multiple main secondary endpoints, the overall type-I error will be controlled by the use of a sequential inferential approach. Statistical significance of the primary parameter is required before drawing inferential conclusions about first key secondary parameter (at the 0.0001 one-sided alpha level at the second interim analysis or at the 0.0249 one-sided alpha level at the final analysis). The order of tests is detailed in Section 9.1.2.1. Inferential conclusions about successive main secondary parameters require statistical significance of the prior one.

This fixed hierarchical approach will ensure a strong control of the overall type-I error rate at the required one-sided level (0.0001 for the second interim analysis and 0.0249 at the final analysis).

No further adjustments will be made for other secondary endpoints or subgroup analyses for which p-values will be provided for descriptive purpose only.

11.4.3 Analyses of safety data

The summary of safety results will be presented by treatment group. No formal inferential testing will be performed. Summaries will be descriptive in nature.

All safety analyses will be performed on the Safety population, using the last available value before first double-blind IMP injection as baseline definition.

The following definitions will be applied to laboratory parameters, and vital signs.

- The potentially clinically significant abnormality (PCSA) values are defined as abnormal values considered medically important by the Sponsor according to predefined criteria/thresholds based on literature review and defined by the Sponsor for clinical laboratory tests, and vital signs.
- PCSA criteria will determine which patients had at least 1 PCSA during the TEAE period, taking into account all evaluations performed during the TEAE period, including unscheduled or repeated evaluations. The number of all such patients will be the numerator for the PCSA percentage.

AE definition:

- Pre-treatment AEs are AEs that developed or worsened or became serious during the PRE-TREATMENT period;
- Treatment-emergent AEs (TEAEs) are AEs that developed or worsened or became serious during the TEAE period;
- Post-treatment AEs are AEs that developed or worsened or became serious during the POST-TREATMENT period.

Possible drug-induced liver injury

The liver function tests, namely ALT, AST, alkaline phosphatase and total bilirubin, are used to assess possible drug induced liver toxicity. The proportion of patients with PCSA values at any post-baseline visit by baseline status will be displayed by treatment group for each parameter.

A graph of distribution of peak values of ALT versus peak values of total bilirubin will also be presented. Note that the ALT and total bilirubin values are presented on a logarithmic scale. The graph will be divided into 4 quadrants with a vertical line corresponding to 3 x ULN for ALT and a horizontal line corresponding to 2 x ULN for total bilirubin.

The incidence of liver-related AEs will be summarized by treatment group. The selection of preferred terms will be based on standardized MedDRA query (SMQ) Hepatic disorder. Time to liver-related treatment discontinuation and time to liver death may also be provided based on hepatic disorder SMQ.

11.4.3.1 Adverse events

Adverse event incidence tables will present by system organ class (SOC) (sorted by internationally agreed order), high-level group term (HLGT), high level term (HLT) and preferred term (PT) sorted in alphabetical order for each treatment group, the number (n) and percentage (%) of patients experiencing an AE. Multiple occurrences of the same event in the same patient will be counted only once in the tables within a treatment phase. The denominator for computation of percentages is the safety population within each treatment group.

Adverse event incidence table will be provided by treatment group for all types of TEAEs: all TEAEs, all treatment emergent AESI (defined with a PT or a prespecified grouping), all treatment emergent SAEs and all TEAEs leading to permanent treatment discontinuation. If any clinically significant signal is detected and need further characterization or for adverse event of clinical interest (e.g. injection site reaction, allergic reaction), exploration of time to onset will be performed for these selected TEAEs as described below to account for the differential exposure time in all patients.

Local injection site reaction could be further described in terms of time pattern (time to first occurrence, duration, recurrence) and intensity.

Selected TEAEs will be also analyzed using time-to-event approach (Kaplan-Meier methodology). Time from the first dose of double-blind IMP injection to the first occurrence of the event will be calculated (only the first event will be counted). Patients without any event will be censored at the end of the TEAE period. Incidence rates at 6 months and by year of exposure will be presented and Kaplan-Meier curves will be provided.

<u>LDL-C < 25 mg/dL:</u>

Summaries of adverse events will be also provided on the safety subgroup population of patients with two consecutive LDL-C < 25mg/dL in the SAR236553 group. Only adverse events, for which it will be confirmed or unclear that they occurred, worsened or became serious after the first level of LDL-C < 25mg/dL will be considered.

<u>Death:</u>

The following deaths summaries will be generated:

- Number (%) of patients who died by study period (TEAE, on-study) and adjudicated reasons, summarized on the safety population by treatment received
- Death in non randomized patients or randomized and not treated patients
- TEAE leading to death (death as an outcome on the AE e-CRF page as reported by the Investigator) by primary SOC , HLGT, HLT and PT showing number (%) of patients sorted by internationally agreed order of SOC and alphabetic order of HLGT, HLT, and PT.

11.4.3.2 Laboratory data and vital signs

The summary statistics (including mean, median, Q1, Q3, standard error, minimum and maximum) of all laboratory variables, all vital signs parameters (raw data and changes from baseline) will be calculated for each visit, last and worst value assessed during the treatment period and presented by treatment group. For selected parameters, mean changes from baseline with the corresponding standard error will be plotted over time (at same time points) in each treatment group.

The incidence of PCSAs at any time during the TEAE period will be summarized by treatment group whatever the baseline level and/or according to the following baseline categories:

- Normal/missing
- Abnormal according to PCSA criterion or criteria

For laboratory parameters for which PCSA criterion is not defined, similar table(s) using the normal range could be provided.

Hepatitis C antibody:

The number and percentage of patients with an observed seroconversion for Hepatitis C Test will be provided by treatment group.

11.4.4 Other endpoints:

All analyses for other endpoints will be performed on the Safety population. The baseline value is defined as the last available value before first double-blind IMP injection.

The number and percentage of patients with calculated LDL-C <25 mg/dL (respectively, calculated LDL-C <15 mg/dL) will be provided by treatment group and visit.

Exploratory variables defined in Section 9.3 will be summarized by time points using number of available data, mean, SD, median, minimum, and maximum for each treatment group (for hs-CRP, Q1 and Q3 will be also provided). The time profile of each parameter will be also plotted by treatment group with the corresponding standard errors. For hs-CRP, the incidence of PCSA at any time during the TEAE period will be also summarized by treatment group using descriptive statistics.

The anti-SAR236553 antibody status (positive/negative) and antibody titers will be summarized by treatment group and visit using descriptive statistics. Anti-SAR236553 antibody will be further described in terms of time-to-onset, persistence (transient/persistent anti-SAR236553 antibodies). Correlations between antibody titers, safety and/or efficacy endpoints could be also provided by graphical methods. Further details will be provided in SAP.

11.5 INTERIM ANALYSIS

Two interim analyses (IA) are planned, when 50% and 75% of the total number of expected events have occurred:

- Interim analysis for futility will be conducted, when approximately 807 events (50% of the targeted number of primary endpoint events) have occurred.
- Interim analysis for futility and overwhelming efficacy will be conducted, when approximately 1210 events (75% of the targeted number of primary endpoint events) have occurred.

Both IA will be performed by the external independent CV DMC Statistician and will be reviewed under the supervision of the CV DMC. The CV DMC will also review secondary

efficacy endpoints and safety data (AEs, laboratory data, vital signs) available at the time of the IA.

Control of the type I and type II error will be ensured using gamma (-5) spending function for type II error (futility) and Gamma (-22) for type I error (efficacy) at each IA (the type I error spending function is also applied at the first IA, even if the objective of this first IA is only futility). It has to be noted that, in order to protect the global type one error in case the decision is taken to overrule the futility rule, non binding boundaries were used.

The following table shows the stopping rules at each interim analysis (using the sample size assumptions described in Section 11.1):

Table 3 - Interim Analyses stopping boundaries corresponding to Gamma (-22) type I error and Gamma (-5) type II error spending functions

**Timing of analyses**	**Stopping boundaries (one-sided p-value and Hazard ratio)**	
	**Futility**	**Overwhelming efficacy**
First IA : 50% of targeted events	p > 0.548<br>(⇔ HR > 1.008)	NA
Second IA: 75% of targeted events	p > 0.19<br>(⇔ HR > 0.951)	p < 0.0001*<br>(⇔ HR < 0.802)

Calculations done using EAST 5.4

*Should the second interim analysis be triggered just before or after 1210 events have been reached, the exact nominal significance level to be used at the second IA would be re-computed based on a Gamma(-22) spending function.

The CV DMC could consider early stopping of the study for overwhelming efficacy at the second IA, if the following conditions are met:

- Stopping boundaries for overwhelming efficacy are crossed.
- Positive trend observed for secondary efficacy endpoints, including all cause mortality, and no excess of non-CV mortality
- Consistency of the treatment effect on the primary efficacy endpoint across subgroups and regions

12 ETHICAL AND REGULATORY STANDARDS

12.1 ETHICAL PRINCIPLES

This clinical trial will be conducted in accordance with the principles laid down by the 18th World Medical Assembly (Helsinki, 1964) and all applicable amendments laid down by the World Medical Assemblies, and the ICH guidelines for good clinical practice (GCP).

In compliance with Sanofi public disclosure commitments, this clinical trial will be recorded in the public registry website clinicaltrials.gov before the enrollment of the first patient. The registry will contain basic information about the trial sufficient to inform interested patients (and their healthcare practitioners) how to enroll in the trial.

12.2 LAWS AND REGULATIONS

This clinical trial will be conducted in compliance with all international guidelines, and national laws and regulations of the country(ies) in which the clinical trial is performed, as well as any applicable guidelines. Please see Section 13.1

12.3 INFORMED CONSENT

The Investigator (according to applicable regulatory requirements), or a person designated by the Investigator, and under the Investigator's responsibility, should fully inform the patient of all pertinent aspects of the clinical trial including the written information giving approval/favorable opinion by the Ethics Committee (IRB/IEC). All participants should be informed to the fullest extent possible about the study, in language and terms they are able to understand.

Prior to a patient's participation in the clinical trial, the written informed consent form should be signed, name filled in and personally dated by the patient or by the patient's legally acceptable representative, and by the person who conducted the informed consent discussion. A copy of the signed and dated written informed consent form will be provided to the patient.

The Informed Consent Form and the optional written Informed Consent Form for pharmacogenetics used by the Investigator for obtaining the patient's informed consent must be reviewed and approved by the Sponsor prior to submission to the appropriate Ethics Committee (IRB/IEC) for approval/favorable opinion.

Prior to collection of blood for pharmacogenetics, the optional pharmacogenetic informed consent form (written) should be signed, name filled in, and personally dated by the patient or by the subject's legally acceptable representative, and by the person who conducted the consent discussion. A copy of the signed and dated written optional informed consent form will be provided to the subject.

12.4 INSTITUTIONAL REVIEW BOARD/INDEPENDENT ETHICS COMMITTEE (IRB/IEC)

As required by local regulation, the Investigator or the Sponsor must submit this clinical trial protocol to the appropriate Ethics Committee (IRB/IEC), and is required to forward to the respective other party a copy of the written and dated approval/favorable opinion signed by the Chairman with Ethics Committee (IRB/IEC) composition.

The clinical trial (study number, clinical trial protocol title and version number), the documents reviewed (clinical trial protocol, informed consent form, Investigator's Brochure, Investigator's curriculum vitae [CV], etc) and the date of the review should be clearly stated on the written (IRB/IEC) approval/favorable opinion.

IMP will not be released at the study site and the Investigator will not start the study before the written and dated approval/favorable opinion is received by the Investigator and the Sponsor.

During the clinical trial, any amendment or modification to the clinical trial protocol should be submitted to the Ethics Committee (IRB/IEC) before implementation, unless the change is necessary to eliminate an immediate hazard to the patients, in which case the IRB/IEC should be informed as soon as possible. It should also be informed of any event likely to affect the safety of patients or the continued conduct of the clinical trial, in particular any change in safety. All updates to the Investigator's Brochure will be sent to the Ethics Committee (IRB/IEC).

A progress report is sent to the Ethics Committee (IRB/IEC) at least annually and a summary of the clinical trial's outcome at the end of the clinical trial.

13 STUDY MONITORING

13.1 RESPONSIBILITIES OF THE INVESTIGATOR(S)

The Investigator(s) and delegated Investigator staff undertake(s) to perform the clinical trial in accordance with this clinical trial protocol, ICH guidelines for Good Clinical Practice and the applicable regulatory requirements.

The Investigator is required to ensure compliance with all procedures required by the clinical trial protocol and with all study procedures provided by the Sponsor (including security rules). The Investigator agrees to provide reliable data and all information requested by the clinical trial protocol (with the help of the e-CRF, Discrepancy Resolution Form [DRF] or other appropriate instrument) in an accurate and legible manner according to the instructions provided and to ensure direct access to source documents by Sponsor representatives.

If any circuit includes transfer of data particular attention should be paid to the confidentiality of the patient's data to be transferred.

The Investigator may appoint such other individuals as he/she may deem appropriate as Subinvestigators to assist in the conduct of the clinical trial in accordance with the clinical trial protocol. All Subinvestigators shall be appointed and listed in a timely manner. The Subinvestigators will be supervised by and work under the responsibility of the Investigator. The Investigator will provide them with a copy of the clinical trial protocol and all necessary information.

13.2 RESPONSIBILITIES OF THE SPONSOR

The Sponsor of this clinical trial is responsible to health authorities for taking all reasonable steps to ensure the proper conduct of the clinical trial protocol as regards ethics, clinical trial protocol compliance, and integrity and validity of the data recorded on the e-s. Thus, the main duty of the monitoring team is to help the Investigator and the Sponsor maintain a high level of ethical, scientific, technical and regulatory quality in all aspects of the clinical trial.

At regular intervals during the clinical trial, the site will be contacted, through monitoring visits, letters or telephone calls, by a representative of the monitoring team to review study progress, Investigator and patient compliance with clinical trial protocol requirements and any emergent problems. These monitoring visits will include but not be limited to review of the following aspects: patient informed consent, patient recruitment and follow-up, SAE documentation and reporting, AESI documentation and reporting, AE documentation, IMP allocation, patient compliance with the IMP regimen, IMP accountability, concomitant therapy use and quality of data.

13.3 SOURCE DOCUMENT REQUIREMENTS

According to the ICH guidelines for Good Clinical Practice, the monitoring team must check the e-CRF entries against the source documents, except for the pre-identified source data directly recorded in the e-CRF. The informed consent form will include a statement by which the patient allows the Sponsor's duly authorized personnel, the Ethics Committee (IRB/IEC), and the regulatory authorities to have direct access to original medical records which support the data on the e-CRFs (eg, patient's medical file, appointment books, original laboratory records, etc). These personnel, bound by professional secrecy, must maintain the confidentiality of all personal identity or personal medical information (according to confidentiality and personal data protection rules).

13.4 USE AND COMPLETION OF CASE REPORT FORMS (e-CRFS) AND ADDITIONAL REQUEST

It is the responsibility of the Investigator to maintain adequate and accurate e-CRFs (according to the technology used) designed by the Sponsor to record (according to Sponsor instructions) all observations and other data pertinent to the clinical investigation in a timely manner. All e-CRFs should be completed in their entirety in a neat, legible manner to ensure accurate interpretation of data.

Should a correction be made, the corrected information will be entered in the e-CRF overwriting the initial information. An audit trail allows identifying the modification.

Data are available within the system to the sponsor as soon as they are entered in the e-CRF.

The computerized handling of the data by the Sponsor when available in the e-CRF may generate additional requests (DRF) to which the Investigator is obliged to respond by confirming or modifying the data questioned. The requests with their responses will be managed through the e-CRF.

13.5 USE OF COMPUTERIZED SYSTEMS

Computerized systems used during the different steps of the study are:

- For data management activities, Medidata RAVE version 5.6.4 (Covance).
- For statistical activities, nQuery Advisor 6.01, SAS, EAST 5.4.
- For pharmacovigilance activities, AWARE , Business Objects XI
- For monitoring activities, CTMS (DCRI), Trial Tracker then CTMS (Covance).
- For medical writing activities, DOMASYS.

External data loading is planned for this clinical trial.

14 ADMINISTRATIVE EXPECTATIONS

14.1 CURRICULUM VITAE

A current copy of the curriculum vitae describing the experience, qualification and training of each Investigator and Subinvestigator will be signed, dated and provided to the Sponsor prior to the beginning of the clinical trial.

14.2 RECORD RETENTION IN STUDY SITES

The Investigator must maintain confidential all study documentation, and take measures to prevent accidental or premature destruction of these documents.

The Investigator should retain the study documents at least 15 years after the completion or discontinuation of the clinical trial.

However, applicable regulatory requirements should be taken into account in the event that a longer period is required.

The Investigator must notify the Sponsor prior to destroying any study essential documents following the clinical trial completion or discontinuation.

If the Investigator's personal situation is such that archiving can no longer be ensured by him/her, the Investigator shall inform the Sponsor and the relevant records shall be transferred to a mutually agreed upon designee.

14.3 CONFIDENTIALITY

All information disclosed or provided by the Sponsor (or any company/institution acting on their behalf), or produced during the clinical trial, including, but not limited to, the clinical trial protocol, the CRFs, the Investigator's Brochure and the results obtained during the course of the clinical trial, is confidential, prior to the publication of results. The Investigator and any person under his/her authority agree to undertake to keep confidential and not to disclose the information to any third party without the prior written approval of the Sponsor.

However, the submission of this clinical trial protocol and other necessary documentation to the Ethics committee (IRB/IEC) is expressly permitted, the IRB/IEC members having the same obligation of confidentiality.

The Subinvestigators shall be bound by the same obligation as the Investigator. The Investigator shall inform the Subinvestigators of the confidential nature of the clinical trial.

The Investigator and the Subinvestigators shall use the information solely for the purposes of the clinical trial, to the exclusion of any use for their own or for a third party's account.

Furthermore, the Investigator and the Sponsor agree to adhere to the principles of personal data confidentiality in relation to the patients, Investigator and its collaborators involved in the study.

14.4 PROPERTY RIGHTS

All information, documents and IMP provided by the Sponsor or its designee are and remain the sole property of the Sponsor.

The Investigator shall not mention any information or the Product in any application for a patent or for any other intellectual property rights.

All the results, data, documents and inventions, which arise directly or indirectly from the clinical trial in any form, shall be the immediate and exclusive property of the Sponsor.

The Sponsor may use or exploit all the results at its own discretion, without any limitation to its property right (territory, field, continuance). The Sponsor shall be under no obligation to patent, develop, market or otherwise use the results of the clinical trial.

As the case may be, the Investigator and/or the Subinvestigators shall provide all assistance required by the Sponsor, at the Sponsor's expense, for obtaining and defending any patent, including signature of legal documents.

14.5 DATA PROTECTION

The patient's personal data, which are included in the Sponsor database shall be treated in compliance with all applicable laws and regulations;

When archiving or processing personal data pertaining to the Investigator and/or to the patients, the Sponsor shall take all appropriate measures to safeguard and prevent access to this data by any unauthorized third party.

The Sponsor also collects specific data regarding Investigator as well as personal data from any person involved in the study which may be included in the Sponsor’s databases, shall be treated by both the Sponsor and the Investigator in compliance with all applicable laws and regulations.

14.6 INSURANCE COMPENSATION

The Sponsor certifies that it has taken out a liability insurance policy covering all clinical trials under its sponsorship. This insurance policy is in accordance with local laws and requirements. The insurance of the Sponsor does not relieve the Investigator and the collaborators from maintaining their own liability insurance policy. An insurance certificate will be provided to the Ethics Committees (IECs/IRBs) or health authorities in countries requiring this document.

14.7 SPONSOR AUDITS AND INSPECTIONS BY REGULATORY AGENCIES

For the purpose of ensuring compliance with the clinical trial protocol, Good Clinical Practice and applicable regulatory requirements, the Investigator should permit auditing by or on the behalf of the Sponsor and inspection by regulatory authorities.

The Investigator agrees to allow the auditors/inspectors to have direct access to his/her study records for review, being understood that these personnel is bound by professional secrecy, and as such will not disclose any personal identity or personal medical information.

The Investigator will make every effort to help with the performance of the audits and inspections, giving access to all necessary facilities, data, and documents.

As soon as the Investigator is notified of a planned inspection by the authorities, he will inform the Sponsor and authorize the Sponsor to participate in this inspection.

The confidentiality of the data verified and the protection of the patients should be respected during these inspections.

Any result and information arising from the inspections by the regulatory authorities will be immediately communicated by the Investigator to the Sponsor.

The Investigator shall take appropriate measures required by the Sponsor to take corrective actions for all problems found during the audit or inspections.

14.8 PREMATURE DISCONTINUATION OF THE STUDY OR PREMATURE CLOSE-OUT OF A SITE

14.8.1 Decided by the Sponsor

Decided by the Sponsor in the following cases

- If the information on the product leads to doubt as to the benefit/risk ratio;
- If the Investigator has received from the Sponsor all IMP, means and information necessary to perform the clinical trial and has not included any patient after a reasonable period of time mutually agreed upon;
- In the event of breach by the Investigator of a fundamental obligation under this agreement, including but not limited to breach of the clinical trial protocol, breach of the applicable laws and regulations or breach of the ICH guidelines on Good Clinical Practice;
- If the total number of patients are included earlier than expected;

In any case the Sponsor will notify the Investigator of its decision by written notice.

14.8.2 Decided by the Investigator

The Investigator must notify (30 days' prior notice) the Sponsor of his/her decision and give the reason in writing.

In all cases (decided by the Sponsor or by the Investigator), the appropriate Ethics Committee(s) (IRB/IEC) and Health Authorities should be informed according to applicable regulatory requirements.

14.9 CLINICAL TRIAL RESULTS

The Sponsor will be responsible for preparing a clinical study report and to provide a summary of study results to the Investigator.

14.10 PUBLICATIONS AND COMMUNICATIONS

During the course of the study, the Executive Steering Committee co-Chairs will form a Publications Subcommittee, which will include all ESC members, and Sponsor representatives and to which selected National Leaders may be invited. The role of the Publication Sub-Committee will be to oversee the publications from the Study, assign authorship, assure that authorship requirements are met, and that the publications which are created are of the highest scientific quality.

The Publications Subcommittee will review and approve all manuscripts of Study results prior to publication.

All study participants (Investigators and Committee members) give full authority to the ESC for primary presentation and/or primary publication of results. No other publication is allowed before the primary publication. Any subsequent presentation or publication by a study participant must be approved by this Publications Committee and/or ESC and make reference to the study and the primary publication.

As the study is being conducted at multiple sites, the Sponsor and the Publications Subcommittee agree that, consistent with scientific standards, first presentation or publication of the results of the study shall be made only as part of a publication of the results obtained by all sites performing the protocol. However, if no multicenter publication has occurred within twelve (12) months of the completion of this study at all sites, the Investigator shall have the right to publish or present independently the results of this study to the review procedure set forth herein. The Investigator shall provide the Sponsor and the Publications Subcommittee with a copy of any such presentation or publication derived from the study for review and comment at least 30 days in advance of any presentation or submission for publication. In addition, if requested by the Sponsor, any presentation or submission for publication shall be delayed for a limited time, not to exceed 90 days, to allow for filing of a patent application or such other measures as the Sponsor deems appropriate to establish and preserve its proprietary rights.

The Investigator shall not use the name(s) of the Sponsor and/or its employees in advertising or promotional material or publication without the prior written consent of the Sponsor. The Sponsor shall not use the name(s) of the Investigator and/or the collaborators in advertising or promotional material or publication without having received his/her and/or their prior written consent(s).

The Sponsor has the right at any time to publish the results of the study.

15 CLINICAL TRIAL PROTOCOL AMENDMENTS

All appendices attached hereto and referred to herein are made part of this clinical trial protocol.

The Investigator should not implement any deviation from, or changes of the clinical trial protocol without agreement by the Sponsor and prior review and documented approval/favorable opinion from the IRB/IEC of an amendment, except where necessary to eliminate an immediate hazard(s) to clinical trial Patients, or when the change(s) involves only logistical or administrative aspects of the trial. Any change agreed upon will be recorded in writing, the written amendment will be signed by the Investigator and by the Sponsor and the signed amendment will be filed with this clinical trial protocol.

Any amendment to the clinical trial protocol requires written approval/favorable opinion by the Ethics Committee (IRB/IEC) prior to its implementation, unless there are overriding safety reasons.

In some instances, an amendment may require a change to the informed consent form. The Investigator must receive an IRB/IEC approval/favorable opinion concerning the revised informed consent form prior to implementation of the change and patient signature should be re-collected if necessary.

16 BIBLIOGRAPHIC REFERENCES

1. SAR236553 CIB Ed. 4 (August 2011).

2. Yusuf S, Zhao F, Mehta SR, et al. Effects of clopidogrel in addition to aspirin in patients with acute coronary syndromes without ST-segment elevation. N Engl J Med 2001; 345: 494-502.

3. Antman EM, Hand M, Armstrong PW, Bates ER, Green LA, Halasyamani LK et al. 2007 focused update of the ACC/AHA 2004 guidelines for the management of patients with ST-elevation myocardial infarction: a report of the American College of Cardiology/American Heart Association Task Force on Practice Guidelines (Writing Group to Review New Evidence and Update the ACC/AHA 2004 Guidelines for the Management of Patients With ST-Elevation Myocardial Infarction). J Am Coll Cardiol 2008; 51: 210–47.

4. Anderson JL, Adams CD, Antman EM, Bridges CR, Califf RM, Casey DE et al., ACC/AHA 2007 guidelines for the management of patients with unstable angina/non–ST-elevation myocardial infarction: a report of the American College of Cardiology/American Heart Association Task Force on Practice Guidelines (Writing Committee to Revise the 2002 Guidelines for the Management of Patients With Unstable Angina/Non–ST-Elevation Myocardial Infarction). J Am Coll Cardiol 2007; 50: 652–726.

5. Krumholz HM, Anderson JL, Bachelder BL, Fesmire FM, Fihn SD, Foody JM et al., ACC/AHA 2008 performance measures for adults with ST-elevation and non–STelevation myocardial infarction: a report of the American College of Cardiology/American Heart Association Task Force on Performance Measures (Writing Committee to Develop Performance Measures for ST-Elevation and Non–ST-Elevation Myocardial Infarction). J Am Coll Cardiol 2008; 52: 2046 –99.

6. Newman C, Tsai J, Szarek M, Luo D, Gibson E. Comparative safety of atorvastatin 80 mg versus 10 mg derived from analysis of 49 completed trials in 14,236 patients. Am J Cardiol 2006; 97: 61-7.

7. LaRosa JC, Grundy SM, Waters DD, Shear C, Barter P, Fruchart JC; Treating to New Targets (TNT) Investigators. Intensive lipid lowering with atorvastatin in patients with stable coronary disease. N Engl J Med 2005; 352 (14): 1425-35.

8. Cannon CP, Braunwald E, McCabe CH, et al, for the Pravastatin or Atorvastatin Evaluation and Infection Therapy-Thrombolysis in Myocardial Infarction 22 Investigators. Intensive versus moderate lipid lowering with statins after acute coronary syndromes. N Engl J Med. 2004;350:1495-1504.

9. Ridker PM, Danielson E, Fonseca FA, Genest J, Gotto AM, Kastelein JJ; JUPITER Study Group. Rosuvastatin to prevent vascular events in men and women with elevated C-reactive protein. N Engl J Med 2008; 359 (21): 2195-207.

10. Grundy SM, Cleeman JI, Merz CNB, et al. Implications of recent clinical trials for National Cholesterol Education Program Adult Treatment Panel III Guidelines. Circulation. 2004; 227-39.

11. The Task Force for the management of dyslipidaemias of the European Society of Cardiology (ESC) and the European Atherosclerosis Society (EAS). ESC/EAS Guidelines for the management of dyslipidaemias. Eur Heart J 2011; 32: 1769-818.

12. Wiviott SD, Cannon CP, Morrow DA, Ray KK, Pfeffer MA, Braunwald E, PROVE IT-TIMI 22 Investigators. Can low-density lipoprotein be too low? The safety and efficacy of achieving very low low-density lipoprotein with intensive statin therapy: a PROVE IT-TIMI 22 substudy. J Am Coll Cardiol 2005; 46: 1411-6.

13. LaRosa JC, Grundy SM, Kastelein JJ, Kostis JB, Greten H. Treating to New Targets (TNT) Steering Committee and Investigators. Safety and efficacy of Atorvastatin-induced very low-density lipoprotein cholesterol levels in Patients with coronary heart disease (a post hoc analysis of the treating to new targets [TNT] study). Am J Cardiol 2007; 100: 747-52.

14. Hsia J, MacFadyen JG, Monyak J, Ridker PM. Cardiovascular event reduction and adverse events among subjects attaining low-density lipoprotein cholesterol <50 mg/dl with rosuvastatin. The JUPITER trial (Justification for the Use of Statins in Prevention: an Intervention Trial Evaluating Rosuvastatin). J Am Coll Cardiol 2011; 57: 1666-75.

15. Everett BM, Mora S, Grossbard J, MacFadyen J, Ridker PM. Safety Profile of Statin-Treated Patients with LDL-C ≤ 30 mg/dL or LDL-C Reduction of ≥ 70%. ACC meeting, 2012.

16. Cholesterol Treatment Trialists' (CTT) Collaboration. Efficacy and safety of more intensive lowering of LDL cholesterol: a meta-analysis of data from 170 000 participants in 26 randomised trials. Lancet 2010; 376: 1670–81.

17. Emberson JR, Kearney PM, Blackwell L, Newman C, Reith C, Bhala Net al., Cholesterol Treatment Trialists' (CTT) Collaboration. Lack of effect of lowering LDL cholesterol on cancer: meta-analysis of individual data from 175,000 people in 27 randomised trials of statin therapy. PLoS One 2012; 7(1): e29849.

18. Brown MS, Goldstein JL. A receptor-mediated pathway for cholesterol homeostasis. Science 1986; 232: 34-47.

19. Hochholzer W and Giugliano RP. Lipid lowering goals: back to nature? Ther Adv Cardiovasc Dis 2010; 4(3):185-91.

20. Cordain L, Eaton SB, Mann N, Hill K. The paradoxical nature of hunter-gatherer diets: meat-based, yet non-atherogenic. Eur J Clin Nutr 2002; 56, Suppl 1: S42-S52.

21. Yan AT, Yan RT, Tan M, et al. Contemporary management of dyslipidemia in high-risk patients: Targets still not met. Am J Med 2006; 119: 676-83.

22. Virani SS, Wooddard LD, Landrum CR, Pietz K, Wang D, Ballantyne CM, Petersen LA. Institutional, provider, and patient correlates of low-density lipoprotein and non-high-density lipoprotein cholesterol goal attainment according to the Adult Treatment Panel III guidelines. Am Heart J 2011; 161:1140-6.

23. Horton JD, Cohen JC, Hobbs HH. Molecular biology of PCSK9: its role in LDL metabolism. Trends Biochem Sci. 2007; 32(2): 71-7.

24. Benjannet S, Rhainds D, Essalmani R, Mayne J, Wickham L, Jin W, et al. NARC-1/PCSK9 and its natural mutants: zymogen cleavage and effects on the low density lipoprotein (LDL) receptor and LDL cholesterol. J Biol Chem 2004; 279: 48865–75.

25. Park SW, Moon YA, Horton JD. Post-transcriptional regulation of low density lipoprotein receptor protein by proprotein convertase subtilisin/kexin type 9a in mouse liver. J Biol Chem 2004; 279: 50630-8.

26. Rashid S, Curtis DE, Garuti R, Anderson NN, Bashmakov Y, Ho YK, et al. Decreased plasma cholesterol and hypersensitivity to statins in mice lacking PCSK9. Proc Natl Acad Sci USA 2005; 102: 5374-9.

27. Abifadel M, Varret M, Rabes JP, Allard D, Ouguerram K, Devillers M, et al. Mutations in PCSK9 cause autosomal dominant hypercholesterolemia. Nat Genet 2003; 34: 154-6.

28. Cohen JC, Boerwinkle E, Mosley TH, Hobbs HH. Sequence variations in PCSK9, low LDL, and protection against coronary heart disease. N Engl J Med 2006; 354: 34-42.

29. Steinberg D, Witztum JL. Inhibition of PCSK9: a powerful weapon for achieving ideal LDL cholesterol levels. Proc Natl Acad Sci U S A. 2009; 106(24): 9546-7.

30. Zhao Z, Tuakli-Wosornu Y, Lagace TA, et al. Molecular characterization of loss-of-function mutations in PCSK9 and identification of a compound heterozygote. Am J Hum Genet 2006; 79: 514-23.

31. Schwartz GG, Olsson AG, Ballantyne C, PJ Barter, Holme IM, Kallend D et al. Rationale and design of the dal-OUTCOMES trial: Efficacy and safety of dalcetrapib in patients with recent acute coronary syndrome dal-OUTCOMES Committees. Am Heart J 2009; 158: 896-901.

32. Cannon CP, Giugliano RP, Blazing MA, Harrington RA, Peterson JL, McCrary Sisk C et al. Rationale and design of IMPROVE-IT (IMProved Reduction of Outcomes: Vytorin Efficacy International Trial): Comparison of ezetimbe/simvastatin versus simvastatin monotherapy on cardiovascular outcomes in patients with acute coronary sindrome. Am Heart J 2008; 156: 826-32. http://www.theheart.org/article/852455.do

33. Note for guidance on non-clinical safety studies for the conduct of human clinical trials and marketing authorization for pharmaceuticals (CPMP/ICH/286/95). http://www.ema.europa.eu/pdfs/human/ich/028695en.pdf.

34. Hicks KA, Hung HM.J, Mahaffey KW, Mehran R, Nissen SE, Stockbridge NL, Targum SL, Temple R; on behalf of the Standardized Data Collection for Cardiovascular Trials Initiative. Standardized Definitions for End Point Events in Cardiovascular Trials. May 31, 2011.

35. Thygesen K, Alpert JS, White HD on behalf of the Joint ESC/ACCF/AHA/WHF Task Force for the Redefinition of Myocardial Infarction. Universal definition of myocardial infarction. Circulation 2007; 116: 2634-53.

36. Mendis S, Thygesen K, Kuulasmaa K, Giampaoli S, Mähönen M, Blackett KN, Lisheng L and Writing group on behalf of the participating experts of the WHO consultation for revision of WHO definition of myocardial infarction. World Health Organization definition of myocardial infarction: 2008–09 revision. Int J Epidemiol 2011; 40 (1): 139-46.

37. Friedewald W. T., Levy R. I., Fredrickson D.S. Estimation of the concentration of Low-Density Lipoprotein Cholesterol in Plasma, without use of the preparative ultracentrifuge. Clinical chemistry 1972; 18(6):499-502.

38. Dolan P. Modeling valuations for EuroQol health states. Medical Care Vol 35, No. 11, pp. 1095-1108. 1997.

39. American Diabetes Association Standards of Medical Care in Diabetes—2012. Diabetes Care. 2012 Jan;35 Suppl 1:S11-63.

40. Chobanian AV, Bakris GL, Black HR, et al, and the National High Blood Pressure Education Program Coordinating Committee. The Seventh Report of the Joint National Committee on Prevention, Detection, Evaluation, and Treatment of High Blood Pressure: the JNC 7 report. JAMA. 2003; 289: 2560-72.

17 APPENDICES

Appendix A Monitoring Plan for LDL-C < 25 mg/dL (0.65 mmol/L)

An independent external academic physician(s) (also known as independent physician) and the dedicated member of the Phase 3a DMC (implemented for all Phase 3 studies evaluating the efficacy and safety of SAR236553 on LDL-C; called Phase 3a studies) will be the coordinators of this specific monitoring. The process will follow that applied for the Phase 3 studies evaluating the effect on LDL-C.

An independent physician will be notified by the central laboratory of all patients who achieve two consecutive LDL-C < 25 mg/dL (0.65 mmol/L). The independent physician will carefully review all available data on the patient, as soon as possible, after such notification. Such data will also include any AEs potentially associated with low LDL-C. AEs potentially associated with low LDL-C will be included in the independent physician related documents, which include, but are not limited to, disorders related to fat-soluble vitamin deficiency, adrenal insufficiency, hypogonadism, neuropathies and hemorrhagic stroke. The independent physician will communicate with the dedicated Phase 3a DMC member on an expedited basis, as needed, or routinely. This dedicated Phase 3a DMC member will then transmit any information as necessary to the common member involved in both DMCs (Phase 3a DMC and CV DMC). The independent physician will continue to regularly monitor and track such patients throughout the study.

For a given patient the follow-up by the independent physician will be maintained until specific actions are undertaken at the next visit:

- On dose of 150 mg Q2W : if LDL-C < 25 mg/dL (including LDL-C < 15 mg/dL) on 2 consecutive measurements, a down-titration to 75 mg Q2W will be done in a blinded manner and for the remaining duration of the study. Unless the LDL-C level remains below 25 mg/dL, the independent physician will stop the review of the given patient.
- On dose of 75 mg Q2W:
  - If LDL-C < 25 mg/dL but ≥ 15 mg/dL on 2 consecutive measurements: study treatment will be continued but the independent physician will continue to monitor the patient.
  - If LDL-C < 15 mg/dL on 2 consecutive measurements: study treatment will be discontinued at the next visit. In order to maintain the blind there will be blinded substitution to placebo injections for the remaining duration of the study. Unless the LDL-C level remains below 25 mg/dL, the independent physician will stop the review of the given patient.

As lipid values will not be communicated to investigators after randomization to maintain the blind, the continuation of the 75 mg dose, the up-titration to the 150 mg dose, the down-titration from 150 to 75 mg, or discontinuation will occur automatically, without site or patient awareness. Moreover, blinded dose adjustment will avoid the need for dummy titrations in the placebo group.

Thus for patients who will still display a LDL-C <25 mg/dL, the independent physician will continue:

- To periodically monitor the patient's AE profile (including AEs potentially associated with low LDL-C)
- To inform the dedicated Phase 3a DMC member on any new and relevant information related to this patient, with expedited discussion, as needed.

Under exceptional circumstances the decision can be made by the dedicated Phase 3a DMC member to notify the site, then:

- The central lab will notify the Investigator.
- The Investigator should then follow the recommended steps outlined below for an alert related to 2 consecutive calculated LDL-C <25 mg/dL (0.65 mmol/L). These steps may include:
  - Call the patient as soon as possible to inquire about interval occurrence of AEs.
  - Decide whether the patient should be requested to rapidly have an unscheduled site visit, or assessment could be done at the next scheduled visit.
  - At the site visit, plan for the following, based on his/her medical judgment:
  a) Assess the need for conducting clinical investigations, arranging specialist consultation(s) as needed, and any relevant additional work-up.
  b) Assess the need for study treatment temporary or permanent discontinuation, or continuation. Regardless of the action taken regarding study treatment, the patient should continue the study as per Section 10.3

In addition to the Investigator oversight, the independent physician continues monitoring the patient and communicating with the designated Phase 3a DMC member as described above.

As mentioned above the dedicated Phase 3a DMC member will then transmit any information as necessary to the common member involved in both DMCs (Phase 3a DMC and CV DMC). The CV DMC will thoroughly analyze the aggregate data for patients who achieve LDL-C < 25 mg/dL during their periodic reviews throughout the study. The CV DMC may adjust the above monitoring plan, if needed.

Appendix B DNA storage samples

1. PROCEDURE FOR COLLECTION, HANDLING, AND SHIPMENT OF OPTIONAL DNA STORAGE SPECIMENS

- Collection schedule: per protocol.
- Labeling of samples
  - Each sample tube should have attached to it the label provided by Covance.

DNA Subject ID:XXX-001-YYY<br>Study Number/Compound (pre-printed)<br>Bar Code (preprinted)<br>Accession Number (pre-printed)

  - In the event of damage or loss of the provided labels, a new label should be immediately requested from Covance.
- Procedure
  - Using a **waterproof pen**, write Subject ID Number on label in space provided.
  - Collect 6 mL of blood, using the 6 mL Vacutainer (Becton Dickinson; K2 EDTA with HEMOGARD Closure) provided, and gently invert tube at least 8 times permitting the specimen to mix with the anticoagulant.
  - **Under no circumstances should the tube be centrifuged.**
  - Ensure the sample tube is clearly and appropriately labeled as described above and in detail in the Covance Laboratory Manual.
  - Immediately freeze and maintain the blood in an upright position at -20°C or colder for storage. Samples must be stored on dry ice if a freezer is not immediately available.
  - Complete the Laboratory Requisition Form (provided by Covance) for each sample.
- Storage
  - Samples must be kept at -20 ºC or colder, organized in a rack in numeric order according to the Subject ID, until ready for packaging and shipping.
- Packaging and shipment
  - Samples and accompanying documents should be packaged according to the detailed instructions in the Covance Laboratory Manual provided at the initiation of the study.
  - Samples must be packaged according to IATA Dangerous Goods Regulations, Packing Instructions 650, using the packing materials provided by contracted company.
  - In the event that the packaging materials or instructions are lost, please contact the study sponsor.

- Ship samples on dry ice to Covance as described in the Global Study Schedule, using the shipping materials provided.

- **Note:** Additional detailed information can be found in the Covance Laboratory Manual, provided at the beginning of the study. This includes additional details regarding:
  - Sample collection kits
  - Sample collection procedures
  - Documentation procedures
  - Packing and shipping instructions
  - Sample kit resupply
  - How to get help

2. SHIPMENT CONTACT NAMES AND ADDRESSES

For Optional DNA Banking Samples:

Use for the Americas: USA and Canada, as well as Latin America and the Islands (Dominican Republic, etc):
Covance CLS Indianapolis
8211 SciCor Drive
Indianapolis, IN
46214-2985 USA
[redacted]

Use for Europe, the Middle East, and Africa:
Covance CLS Genève
Rue Moïse-Marcinhes 7
1217 Meyrin/Genève-CH
[redacted]

Use for Asia-Pacific, including Thailand, Malaysia, and Australia:
Covance CLS Singapore
1 International Business Park
#05-12A/B The Synergy
Singapore 609917
[redacted]

Use for China only:
Covance CLS Shanghai
1st Floor, No. 6 Building
151 Li Bing Rd
Zhangjiang Hi-Tech Park
Shanghai 201203
China
[redacted]

Appendix C General Guidance for the follow-up of laboratory abnormalities by Sanofi

NEUTROPENIA

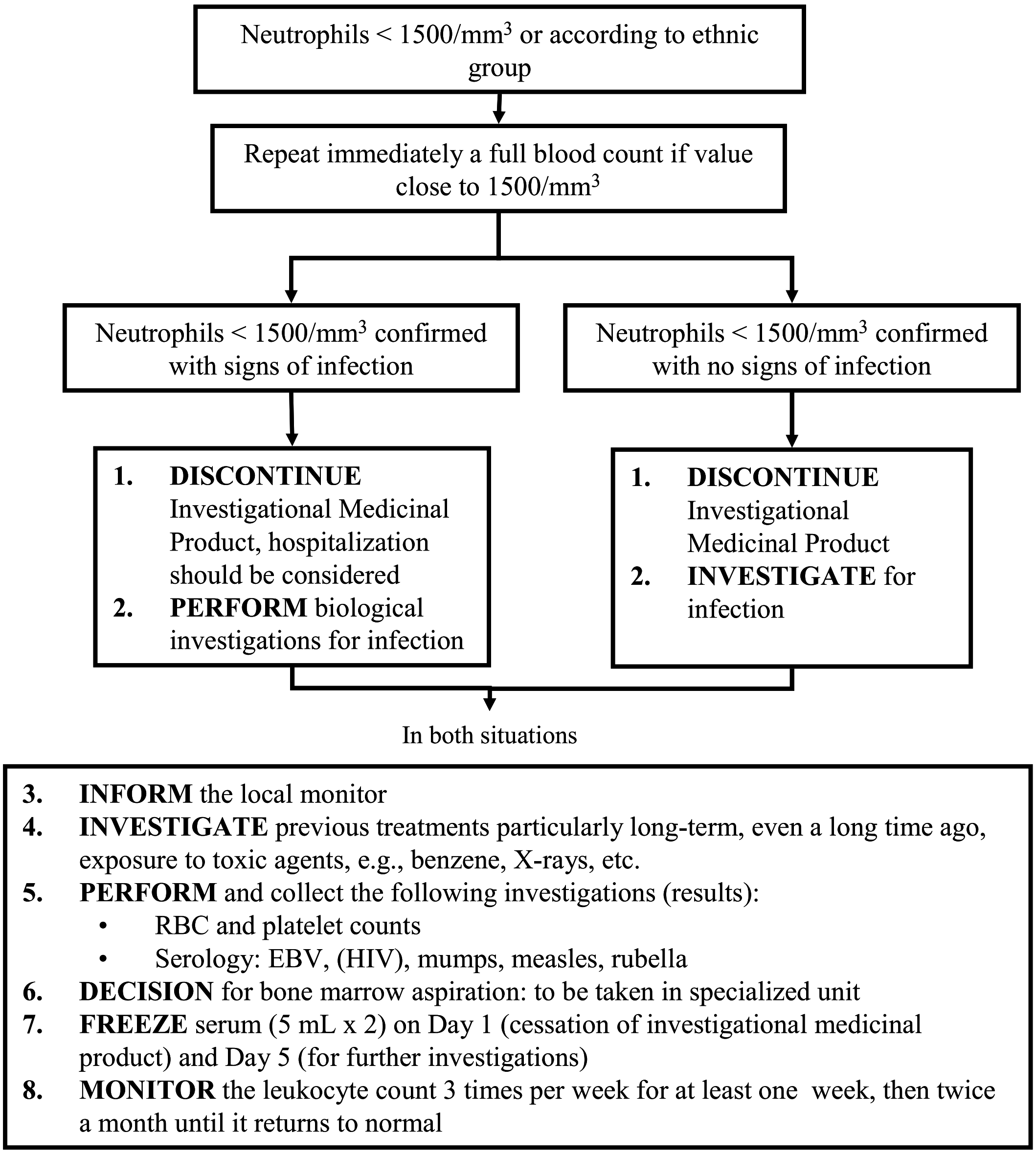

Note:

•The procedures described in the above flowchart are to be discussed with the patient only in case the event occurs. If applicable (according to local regulations), an additional consent (e.g., for HIV testing) will only be obtained in the case the event actually occurs.

•For individuals of African descent, the relevant value of concern is <1000/mm3

Neutropenia are to be recorded as AE only if they are :

- Symptomatic, and/or
- Requiring either corrective treatment or consultation, and/or
- Leading to IMP discontinuation or modification of dosing, and/or
- Fulfilling a seriousness criterion [in that case, the event (SAE) should be notified within 1 working day to the MT], and/or
- Defined as an Adverse Event of Special Interest (AESI) with immediate notification

THROMBOCYTOPENIA

Platelets < 100 000/mm^3 (rule out EDTA – induced pseudo-thrombocytopenia)

↓

Repeat immediately the count (rule out EDTA anticoagulant in the sample)

↓

Platelets < 100 000/mm^3 confirmed with bleeding

1. **DISCONTINUE** Investigational Medicinal Product
2. **HOSPITALIZATION** should be considered

Platelets < 100 000/mm^3 confirmed with no bleeding

1. **DISCONTINUE** Investigational Medicinal Product
2. **INVESTIGATE** for bleeding

↓

In both situations

3. **INFORM** the local Monitor
4. **QUESTION** about last intake of quinine (drinks), alcoholism, heparin administration
5. **PERFORM** or collect the following investigations:
   - Complete blood count, schizocytes, creatinine
   - Bleeding time and coagulation test (fibrinogen, PT, aPTT), Fibrin Degradation Product
   - Viral serology: EBV, HIV, mumps, measles, rubella
6. **FREEZE** serum (5 mL x 2) on Day 1 (end of treatment) and Day 5 to test for drug-induced antiplatelets antibodies
7. **DECISION** for bone marrow aspiration: to be taken in specialized unit
   - On Day 1 in the case of associated anemia and/or leukopenia
   - On Day 8 if the Platelets remain < 50 000/mm^3
8. **MONITOR** the platelet count every day for at least one week and then regularly until it returns to normal

Note:
the procedures described in the above flowchart are to be discussed with the patient only in case the event occurs. If applicable (according to local regulations), an additional consent (e.g., for HIV testing) will only be obtained in the case the event actually occurs.

Thrombocytopenia are to be recorded as AE only if they are:

- Symptomatic, and/or
- Requiring either corrective treatment or consultation, and/or
- Leading to IMP discontinuation or modification of dosing, and/or
- Fulfilling a seriousness criterion [in that case, the event (SAE) should be notified within 1 working day to the MT], and/or
- Defined as an Adverse Event of Special Interest (AESI) with immediate notification

INCREASE IN ALT

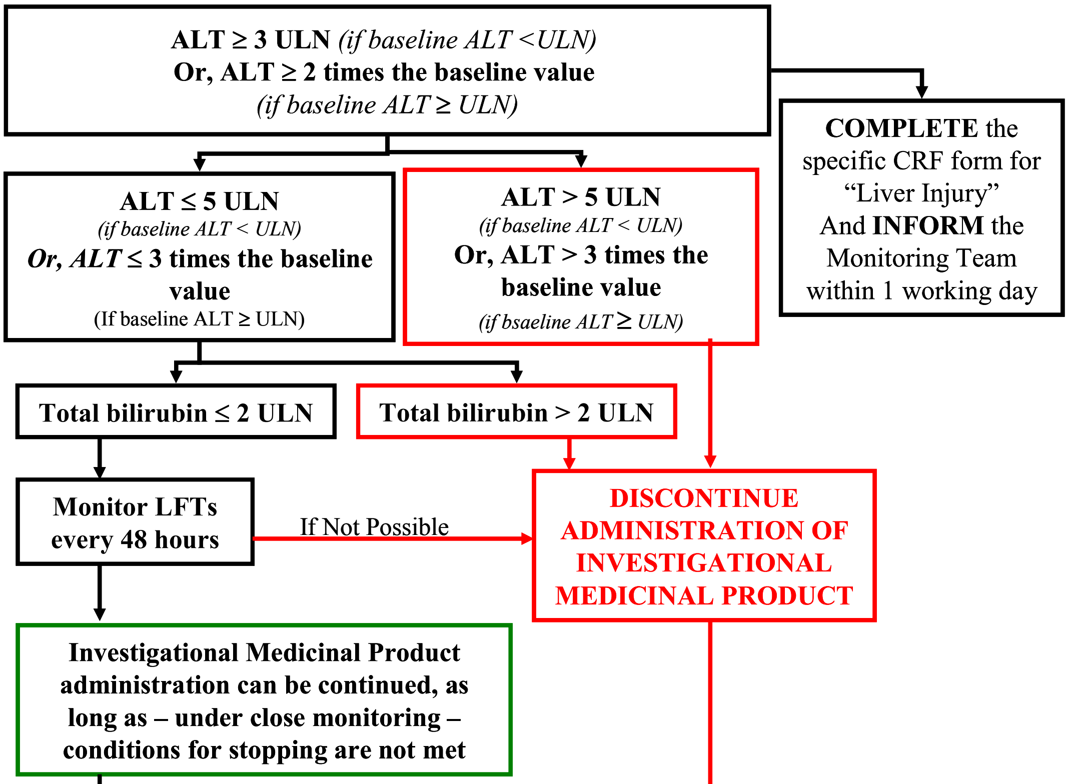

In ANY CASE, **FOLLOW** the instructions #1 to 6 listed in the box below.

1. **INVESTIGATE** specifically for malaise with or without loss of consciousness, dizziness, and/or hypotension and/or episode of arrhythmia in the previous 72 hours; rule out muscular injury
2. **PERFORM** the following tests:
   - LFTs : AST, ALT, Alkaline Phosphatase, Total and Conjugated Bilirubin and Prothrombin Time / INR
   - CPK, serum creatinine, complete blood count
   - Anti-HAV IgM, anti-HBc IgM, anti-HCV and HCV RNA , anti-CMV IgM and anti-HEV IgM antibodies,
   - Auto-antibodies : anti-nuclear, anti-DNA, anti-smooth muscle, anti-LKM
   - Depending on the clinical context, check for recent infection with EBV, Herpes viruses and toxoplasma
   - Hepatobiliary ultrasonography (can be completed by other imaging investigations if needed)
3. **CONSIDER** consultation with hepatologist
4. **CONSIDER** patient hospitalisation if INR>2 (or PT<50%) and/or central nervous system disturbances suggesting hepatic encephalopathy.
5. **MONITOR LFTs**
   - **If investigational medicinal product is continued**: **every 48 hours** until return to normal (<2ULN) or baseline. If ALT elevation persists beyond 2 weeks then perform LFTs every 2 weeks and 15 to 30 days after the last dose according to the study protocol.
   - **If investigational medicinal product is discontinued**: as closely as possible to every 48 hours until stabilization then every 2 weeks until return to normal (<2ULN) or baseline or for at least 3 months, whichever comes last.
6. **FREEZE** serum (5 ml X 2)

NOTE: IN ADDITION, AS SOON AS A SERIOUSNESS CRITERION IS MET, THE EVENT SHOULD BE NOTIFIED WITHIN 1 WORKING DAY TO THE MONITORING TEAM.

ACUTE RENAL FAILURE

Rapid increase in serum creatinine over 150 µmol/L or rapid decrease in creatinine clearance below 50ml/mn

Can be rapidly reversed:
- By volume repletion
- Or relief of urinary tract obstruction (according to etiology)

1. Investigational Medicinal Product may be continued
2. **MONITOR** serum creatinine until return to baseline level

Cannot be rapidly reversed:
- Occurrence/aggravation of life threatening symptoms of ARF: anemia, hyperkalemia, hyperuricemia, metabolic acidosis, cardiac insufficiency, pulmonary edema, arrhythmia, DIC, etc.
- And/or predominant elimination of Investigational Medicinal Product by renal route

1. **INFORM** the local monitor
2. **DISCONTINUE** Investigational Medicinal Product administration
3. **HOSPITALIZATION** should be considered and seek for nephrologic advice
4. **PERFORM** the following examinations:
   - BP, HR, hydration status, ECG
   - Blood count
   - Liver function tests + CPK
   - Biochemistry, including urea
   - Urinalysis
5. **FREEZE** serum (5mL x 2)
6. **MONITOR** renal function until return to baseline level (every day at the beginning, then every week)

Acute renal failure is to be recorded as AE only if it is :
- Symptomatic, and/or
- Requiring either corrective treatment or consultation, and/or
- Leading to IMP discontinuation or modification of dosing, and/or
- Fulfilling a seriousness criterion [in that case, the event (SAE) should be notified within 1 working day to the MT], and/or
- Defined as an Adverse Event of Special Interest (AESI) with immediate notification

SUSPICION OF RHABDOMYOLYSIS

Muscular symptoms (myalgia, pain, weakness, dark urines)

Systematic CPK assessment as per protocol

↓

Perform CPK

↓

If Increase in CPK (expressed in ULN)

↓

> 3 ULN

↓

Repeat immediately the count.
If confirmed, inform the local monitor and

↓

INVESTIGATE for the origin:

- **PERFORM**:
  - ECG
  - CPK-MB -MM
  - Troponin
  - Creatinine
  - Iono (k+, Ca^2+)
  - Transaminases + Total and conjugated bilirubin
  - Myoglobin (serum and urines)
- **FREEZE** SERUM (5mlx2) for PK
- **INTERVIEW** the patient about a recent intensive muscular effort, trauma, convulsions, electrical injury, injury or stress to the skeletal muscle, multiple intramuscular injections, recent surgery, concomitant medications, consumption of alcohol, morphine, cocaine.
- **SEARCH** for alternative causes to cardiac or muscular toxicity, ie: stroke, pulmonary infarction, dermatomyositis or polymyositis, convulsions, hypothyroidism, delirium tremens, muscular dystrophies.

↓

If either the cardiac origin or the rhabdomyolysis is confirmed or if CPK > 10 ULN:

1. **DISCONTINUE** Investigational Medicinal Product administration
2. **MONITOR** CPK every 3 days for the first week then once weekly until return to normal or for at least 3 months
3. **HOSPITALIZATION** should be considered

If the cardiac origin or the rhabdomyolysis is ruled out and if CPK ≤ 10 ULN:
MONITOR CPK every 3 days for the first week then once weekly until return to normal or for at least 3 months

Suspicion of rhabdomyolysis is to be recorded as AE only if it is:

- Symptomatic, and/or
- Requiring either corrective treatment or consultation, and/or
- Leading to IMP discontinuation or modification of dosing, and/or
- Fulfilling a seriousness criterion [in that case, the event (SAE) should be notified within 1 working day to the MT], and/or
- Defined as an Adverse Event of Special Interest (AESI) with immediate notification

Appendix D Guidelines for hemoglobin (Hb) decrease ≥ 1.5 g/dL

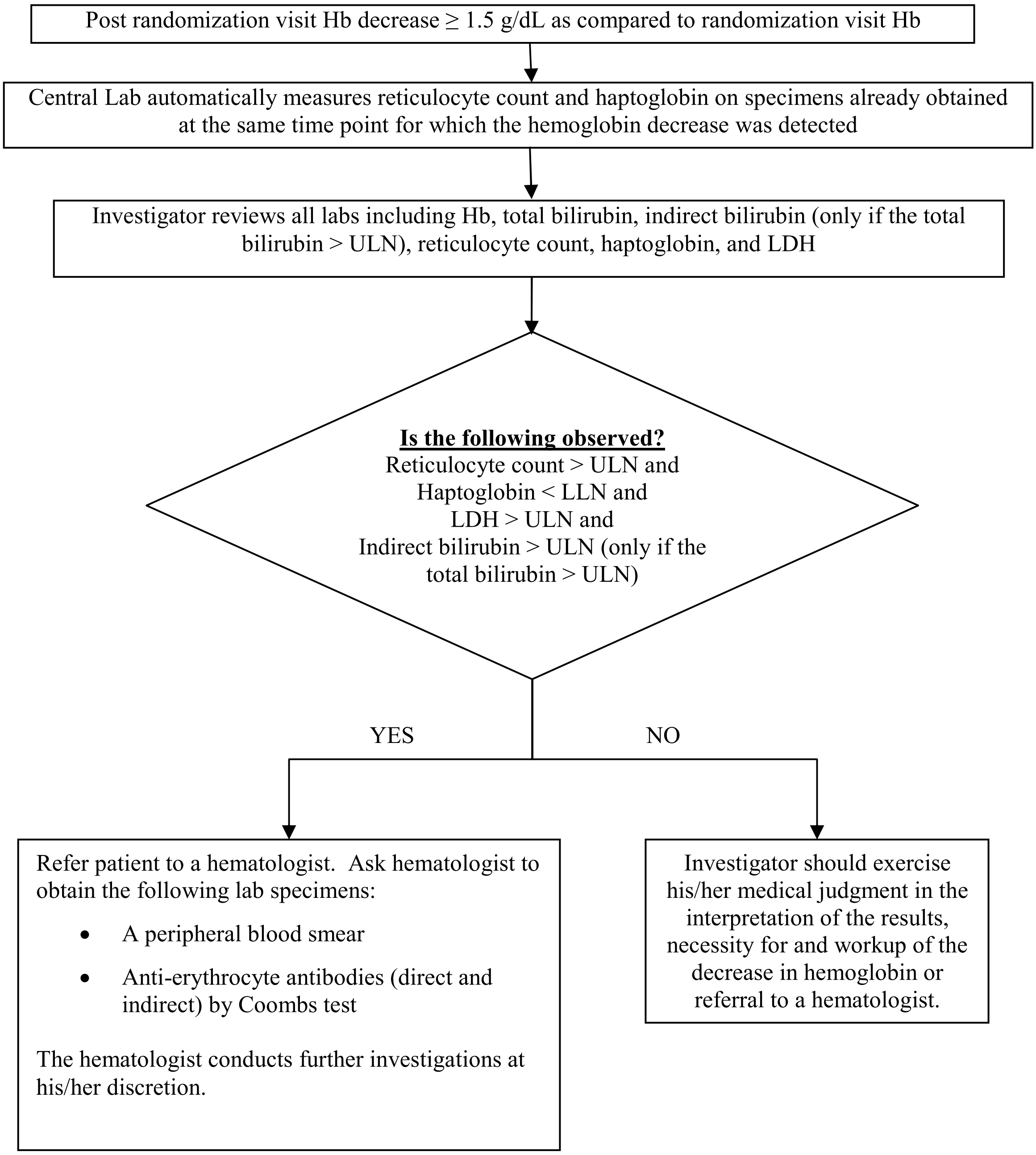

Suspicion of hemolytic anemia is recorded as AE only if:

- Symptomatic and/or
- Requiring either corrective treatment or consultation, and/or
- Leading to IMP discontinuation or modification of dosing, and/or
- Fulfilling a seriousness criterion

Appendix E Summary of Adverse Event Reporting Instructions

EVENT CATEGORY	REPORTING TIMEFRAME	SPECIFIC EVENTS IN THIS CATEGORY	CASE REPORT FORM COMPLETION		
			AE form	Safety Complementary Form	Other specific forms
Adverse Event (non-SAE, non-AESI)	Routine	Any AE that is not SAE or AESI	Yes	No	No
Serious Adverse Event (non-AESI or AESI)	Expedited (within 1 working day)	Any AE meeting seriousness criterion per Section 10.4.1.2	Yes	Yes	No, unless applicable
Adverse Event of Special Interest (AESI) WITHOUT immediate notification (non-SAE)	Routine	Asymptomatic overdose with IMP	Yes	No	No
		Allergy events (except events specified in Section 10.4.6.2)	Yes	No	Yes
		Local injections site reactions	Yes	No	Yes

EVENT CATEGORY	REPORTING TIMEFRAME	SPECIFIC EVENTS IN THIS CATEGORY	CASE REPORT FORM COMPLETION		
			AE form	Safety Complementary Form	Other specific forms
Adverse Event of Special Interest (AESI) WITH immediate notification (non-SAE)	Expedited (within 1 working day)	Pregnancy of female patient/subject	Yes	Yes	Yes
		Symptomatic overdose with IMP	Yes<br>Yes	Yes<br>Yes	No<br>Yes
		Allergic drug reactions and/or local injections site reactions deemed to be allergic as specified in Section 10.4.6.1	Yes	No	Yes
		Hemolytic anemia	Yes	No	Yes
		Increase in ALT as follows:<br>- ALT ≥3 ULN (if baseline ALT <ULN) Or<br>- ALT ≥2 times the baseline value (if baseline ALT ≥ULN)<br>Please refer to related flowchart (per *Appendix C*)	Yes	Yes	Yes
Laboratory, vital sign, or ECG abnormality (non-SAE, non-AESI) that is:<br>- Symptomatic<br>- Requiring corrective treatment or consultation<br>- Leading to IMP discontinuation or dose regimen modification	Routine	Neutropenia (per *Appendix C*)	Yes	No	No
		Thrombocytopenia (per *Appendix C*)	Yes	No	No
		Acute renal insufficiency (per *Appendix C*)	Yes	No	No
		Increase in CPK and suspicion of rhabdomyolysis (per *Appendix C*)	Yes	No	No

Appendix F Assessment of Local Injection Site Reactions

Reaction to Injectable Product	Mild (Grade 1)	Moderate (Grade 2)	Severe (Grade 3)	Very Severe (Grade 4)
Pain	Does not interfere with activity	Interferes with activity or repeated use of non-narcotic pain reliever	Prevents daily activity or repeated use of narcotic pain reliever	Emergency Room (ER) visit or hospitalization
Tenderness	Mild pain to touch	Pain with movement	Significant pain at rest	ER visit or hospitalization
Erythema / Redness *	2.5 – 5 cm	5.1 – 10 cm	>10 cm	Necrosis or exfoliative dermatitis
Swelling **	2.5 – 5 cm and does not interfere with activity	5.1 – 10 cm or interferes with activity	> 10 cm or prevents daily activity	Necrosis
Itching	Does not interfere with activity	Interferes with activity or repeated use of topical or systemic treatment	Prevents daily activity or leads to other significant dermatologic conditions (such as infection, scarring, etc.)	Emergency Room (ER) visit or hospitalization
Other (Please specify)***	No modification of daily activities and/or does not require symptomatic treatment	Hinders normal daily activities and/or requires symptomatic treatment.	Prevents daily activities and requires symptomatic treatment.	Emergency Room (ER) visit or hospitalization

* In addition to grading the measured local reaction at the greatest single diameter, the measurement should be recorded as a continuous variable
** Swelling should be evaluated and graded using the functional scale as well as the actual measurement
*** Please specify the other signs or symptoms (for example, hematoma, discoloration, re-activation, etc.)

ADAPTED from the toxicity grading scale table from the FDA Draft Guidance for Industry: Toxicity Grading Scale for Healthy Adult and Adolescent Volunteers Enrolled in Preventive Vaccine Clinical Trials April 2005

Appendix G EQ-5D Patient Questionnaire

[redacted]

AMENDED CLINICAL TRIAL PROTOCOL NO. 11

COMPOUND: alirocumab (SAR236553/REGN727)

A Randomized, Double-Blind, Placebo-Controlled, Parallel-Group Study to Evaluate the Effect of Alirocumab (SAR236553/REGN727) on the Occurrence of Cardiovascular Events in Patients Who Have Recently Experienced an Acute Coronary Syndrome

STUDY NUMBER: EFC11570

VERSION DATE/STATUS: 25-Feb-2016

STUDY NAME: ODYSSEY Outcomes

Version Number:	1	EudraCT<br>IND Number(s)	2011-005698-21<br>105574
Date:	25-Feb-2016	Total number of pages:	171

Any and all information presented in this document shall be treated as confidential and shall remain the exclusive property of Sanofi (or any of its affiliated companies). The use of such confidential information must be restricted to the recipient for the agreed purpose and must not be disclosed, published or otherwise communicated to any unauthorized persons, for any reason, in any form whatsoever without the prior written consent of Sanofi (or the concerned affiliated company); 'affiliated company' means any corporation, partnership or other entity which at the date of communication or afterwards (i) controls directly or indirectly Sanofi, (ii) is directly or indirectly controlled by Sanofi, with 'control' meaning direct or indirect ownership of more than 50% of the capital stock or the voting rights in such corporation, partnership or other entity

HISTORY

Amended Clinical Trial Protocol 11	Version number: 1	Date: 25-Feb-2016
Protocol Amendment 11	Version number: 1	Date: 25-Feb-2016
Amended Clinical Trial Protocol 10 - Colombia	Version number: 1	Date: 27-Jan-2016
Protocol Amendment 10 - Colombia	Version number: 1	Date: 27-Jan-2016
Amended Clinical Trial Protocol 09 - China	Version number: 1	Date: 10-Dec-2015
Protocol Amendment 09 - China	Version number: 1	Date: 10-Dec-2015
Amended Clinical Trial Protocol 08 - Germany	Version number: 1	Date: 08-Dec-2015
Amended Clinical Trial Protocol 08 - Latvia	Version number: 1	Date: 08-Dec-2015
Amended Clinical Trial Protocol 08 - France	Version number: 1	Date: 08-Dec-2015
Amended Clinical Trial Protocol 08	Version number: 1	Date: 16-Apr-2015
Protocol Amendment 08	Version number: 1	Date: 16-Apr-2015
Amended Clinical Trial Protocol 07 - China	Version number: 1	Date: 11-Apr-2014
Protocol Amendment 07 - China	Version number: 1	Date: 11-Apr-2014
Amended Clinical Trial Protocol 06 - Germany	Version number: 1	Date: 13-Jan-2014
Amended Clinical Trial Protocol 06 - Latvia	Version number: 1	Date: 13-Jan-2014
Amended Clinical Trial Protocol 06 - France	Version number: 1	Date: 13-Jan-2014
Amended Clinical Trial Protocol 06	Version number: 2	Date: 05-Dec-2013
Protocol Amendment 06	Version number: 2	Date: 05-Dec-2013
Amended Clinical Trial Protocol 05 - China	Version number: 1	Date: 29-Jul-2013
Protocol Amendment 05 - China	Version number: 1	Date: 29-Jul-2013
Amended Clinical Trial Protocol 04 - Germany	Version number: 1	Date: 25-Mar-2013
Protocol Amendment 04 - Germany	Version number: 1	Date: 25-Mar-2013
Amended Clinical Trial Protocol 03 - Latvia	Version number: 1	Date: 22-Mar-2013
Protocol Amendment 03 - Latvia	Version number: 1	Date: 22-Mar-2013
Amended Clinical Trial Protocol 02 - France	Version number: 1	Date: 01-Mar-2013
Protocol Amendment 02 - France	Version number: 1	Date: 01-Mar-2013
Amended Clinical Trial Protocol 01	Version number: 1	Date: 17-Sep-2012
Protocol Amendment 01	Version number: 1	Date: 17-Sep-2012
Clinical Trial Protocol	Version number: 1	Date: 19-Jul-2012

(electronic 1.0)

NAMES AND ADDRESSES

**COORDINATING INVESTIGATOR**	Name:<br>Address:<br><br>Tel:<br>Fax:<br>E-mail:	
**MONITORING TEAM'S REPRESENTATIVE**	Name:<br>Address:<br><br>Tel:<br>Fax:<br>E-mail:	
**SPONSOR**	Company:<br>Address:	
**OTHER EMERGENCY TELEPHONE NUMBERS**		

(electronic 1.0)

CLINICAL TRIAL SUMMARY

COMPOUND: ALIROCUMAB (SAR236553/REGN727)<br>STUDY No: EFC11570<br>STUDY NAME: ODYSSEY Outcomes	
**TITLE**	A Randomized, Double-Blind, Placebo-Controlled, Parallel-Group Study to Evaluate the Effect of Alirocumab (SAR236553/REGN727) on the Occurrence of Cardiovascular Events in Patients Who Have Recently Experienced an Acute Coronary Syndrome
**INVESTIGATOR/TRIAL LOCATION**	Worldwide – multicenter study
**PHASE OF DEVELOPMENT**	IIIb
**STUDY OBJECTIVE(S)**	**Primary objective**<br>The primary objective of this study is to compare the effect of alirocumab with placebo on the occurrence of cardiovascular events (composite endpoint of coronary heart disease (CHD) death, non-fatal myocardial infarction (MI), fatal and non-fatal ischemic stroke, unstable angina requiring hospitalization) in patients who have experienced an acute coronary syndrome (ACS) event 4 to 52 weeks prior to randomization and are treated with an LMT regimen that is statin-intensive (defined as atorvastatin 40 or 80 mg, or rosuvastatin 20 or 40 mg) or at maximally tolerated dose of these given statins and optimized for long-term chronic use with other non-statin LMT(s) at investigator's discretion.<br><br>**Secondary objective(s)**<br>To compare the efficacy of alirocumab versus placebo on secondary endpoints (any CHD event, major CHD event, any CV event, composite of all-cause mortality/non-fatal MI/non-fatal ischemic stroke, all-cause mortality).<br>*A Clinical Events Committee (CEC) will be established to adjudicate, in a blinded fashion, events corresponding to the primary and secondary cardiovascular endpoints as well as all causes of death.*<br>• To evaluate the safety and tolerability of alirocumab throughout the study.<br>• To evaluate the development of anti- alirocumab antibodies.<br>• To evaluate the effect of alirocumab on low-density lipoprotein cholesterol (LDL-C), apolipoprotein B (ApoB), and non-high-density lipoprotein cholesterol (non-HDL-C).

**STUDY DESIGN**	This is a double-blind, randomized, placebo-controlled, parallel-group study, multi-national, multicenter study. Randomization will be stratified according to country.<br>The study will comprise 2 periods:<br>• A run-in period (~2 to 16 weeks) during which the background lipid-modifying therapy (LMT) will be adjusted as needed, to ascertain the patient is receiving the required intensive statin treatment (defined as atorvastatin 40 or 80 mg, or rosuvastatin 20 or 40 mg) or the maximally tolerated doses of these given statins, or other non-statin LMTs prior to randomization, and to stabilize this treatment.<br>• A double-blind treatment period (~2 to 5 years) during which the following should be emphasized and reinforced:<br>- compliance with study visits and assessments (all randomized patients, including those who permanently discontinued treatment early, must remain in the study and be followed until the end of the study)<br>- compliance with blinded study treatment (IMP)<br>- compliance with required background LMT regimen (which should be continued for as long as it remains well tolerated)<br>- compliance with requirement to not check cholesterol levels<br><br>**1/ The Run-in period** starts with a screening visit (V1), continues with a qualifying visit (V2), and ends with a randomization visit (V3). V1 and V2 visits can be separate or combined.<br><u>The main goals of the run-in period are to ensure that</u>:<br>• patient has received prior to V2 a required LMT regimen that is<br>- statin-intensive (defined as atorvastatin 40 or 80 mg or rosuvastatin 20 or 40 mg) or maximal tolerated dose of one these 2 statins<br>- optimized for long-term chronic use with addition of non-statin LMTs (at Investigator's discretion)<br>- well tolerated after at least 2 weeks of stable dose<br>• lipid criteria outlined in the inclusion criteria are met at V2<br>• patient (and family) has been adequately informed and agrees to participate in a long-term study with an injection every 2 weeks<br>• patient has been trained to self-administer study drug injections<br>• there are no exclusion criteria<br><br><u>The run-in period should be planned carefully</u>, keeping in mind 3 elements:<br>• The visit and time interval requirements with respect to:<br>- Index ACS event<br>- V1 (start of run-in period)<br>- V2/V2b (collection of lipid qualifying labs)<br>- V3 (randomization)<br>• The required background lipid-modifying therapy (LMT)<br>• The coronary revascularization strategy

	Visit and Time Intervals during Run-In Period<br>• Interval between index ACS event and V1 is flexible:<br>- V1 can be performed as early as on the day of the index ACS event and no later than 50 weeks after the index ACS event if V1 and V2 are separate visits (or 50 weeks + 5 days after ACS event, if combined V1/V2)<br>• Run-In Period (from V1 to V3) includes 2 time interval requirements (and both should be met):<br>- Time from V1 to V3 (i.e. from start of run-in period to randomization): this is the duration of the overall run-in period. It should be between 2 weeks (14 days) and 16 weeks (+5 days).<br>- Time from index ACS event to randomization (V3): this includes the time between index ACS event and V1 (start of run-in period) plus time from V1 to V3 (randomization). V3 (randomization) should occur no earlier than 4 weeks (28 days) and no later than 52 weeks (+ 5 days) post index ACS event<br>The run-in period can be conducted in one of 2 ways, depending on whether the required LMT regimen prior to V2 was already administered for at least 2 weeks (and found well tolerated) prior to V1 or not.<br>- If required LMT regimen was not already administered for at least 2 weeks prior to V1, then V1 and V2 should be separate visits with an interval of at least 2 weeks, during which required LMT regimen is administered. V3 (randomization) can occur as soon as the qualifying lipid lab results from V2 are obtained from the central lab (typically within 2-5 days of collection), pending patients meets all eligibility criteria<br>- If it is documented that required LMT regimen was already administered for at least 2 weeks (and found well tolerated) prior to V1, then V1 and V2 can be combined. V3 (randomization) should then occur at least 2 weeks (14 days) after the combined V1/V2 visit<br>An optional additional V2 visit (V2b) with repeat central laboratory lipid assessments may be scheduled if needed.<br><br>Required background LMT during the run-in period<br>Investigator must have identified prior to V2 (or combined V1/V2), a background LMT regimen that is:<br>• Statin-intensive (defined as atorvastatin 40 or 80 mg, or rosuvastatin 20 or 40 mg), or maximally tolerated dose of these given statins; statins other than atorvastatin, rosuvastatin are not allowed<br>• Optimized for long-term chronic use, with addition of non-statin LMT(s), as per Investigator's discretion; no fibrate allowed (other than fenofibrate or fenofibric acid)<br>- In statin-intolerant patients (defined as intolerance to 2 or more statins), LMT can be optimized with non-statins LMT only (e.g. ezetimibe, or other non-statin LMT)<br>• Well tolerated for at least 2 weeks (at stable dose) prior to V2

	Coronary revascularization strategy<br>When indicated for the treatment of the qualifying index ACS event, a coronary revascularization (PCI, CABG) may have taken place before V1, but may also occur during the run-in period.<br>If a PCI and/or CABG occur during the run-in period, randomization should be scheduled to allow for a minimum 2 week interval between the last coronary procedure and the randomization visit V3.<br><br>Rescreening<br><br>A patient who fails screening and left the study may undergo rescreening and re-enter the study (only once) - see Section 6.1.3.<br><br>**2/ The double-blind treatment period** will continue for 24 months after the closing of randomization ex-China (ie, last date of randomization in all countries except China) or until the target number of events (1613) is reached whichever comes last.<br>• *Closing of randomization ex-China* will occur shortly after a total of 18,000 patients have been randomized (these may or may not include some patients randomized in China).<br>• *Closing of randomization in China* will occur shortly after a total of 600 patients have been randomized in China or at the common study end date (see Section 6.2.2), whichever comes first.<br>Therefore, at the end of the double-blind treatment period, the overall population will include about 18,000 randomized patients who have either died or been followed for a minimum of 24 months, supplemented with an additional subset of patients from China (~600) who may be followed for less than 24 months. The corresponding estimated study duration is 64 months (as described in the sample size considerations – Section 11.1).<br>During this double-blind treatment period, the dosing of alirocumab is intended to achieve LDL-C levels of 30-50 mg/dL (0.78-1.29 mmol/L) which is considered as the physiologic ideal level and to avoid levels of LDL-C that are clearly below the physiologic range (ie,<15 mg/dL or 0.39 mmol/L)). To achieve this goal the following up-titration or down-titration scheme (including discontinuation if needed) will be applied:<br>• At randomization Visit (V3), the starting dose of alirocumab will be 75 mg every 2 weeks (Q2W). At Month 2, patients randomized to alirocumab will, in a blinded manner, either:<br>- Continue alirocumab 75 mg Q2W, if the Month 1 LDL-C is <50 mg/dL (1.29 mmol/L) **OR**<br>- Be up-titrated to alirocumab 150 mg Q2W, if the Month 1 LDL-C is ≥50 mg/dL (1.29 mmol/L).<br><br>• At subsequent visits, for patients on alirocumab, the following adjustments may be applied depending on the dose received:<br>- For patients receiving 150 mg Q2W: if LDL-C <25 mg/dL (0.65 mmol/L) (including LDL-C < 15 mg/dL [0.39 mmol/L]) on 2 consecutive measurements, down-titration to 75 mg Q2W will be done in a blinded manner at the next visit. Additional monitoring will be implemented until the down-titration is done.

<table>
<tr><td></td><td>
- For patients receiving 75 mg Q2W:
  o If LDL-C <25 mg/dL (0.65 mmol/L) but ≥ 15 mg/dL (0.39 mmol/L) on 2 consecutive measurements: alirocumab will be continued but additional monitoring will be implemented, to further confirm the safety of low LDL-C levels.
  o If LDL-C <15 mg/dL (0.39 mmol/L) on 2 consecutive measurements: alirocumab treatment will be discontinued at the next visit. In order to maintain the blind there will be blinded substitution to placebo injections for the remaining duration of the study. Additional monitoring will be implemented until the study treatment discontinuation is done.
As lipid values will not be communicated to investigators after randomization to maintain the blind, the continuation of the 75 mg dose, the up-titration to the 150 mg dose, the down-titration from 150 to 75 mg, or discontinuation will occur automatically, without site or patient awareness. Moreover, blinded dose adjustment will avoid the need for dummy titrations in the placebo group.
Atorvastatin or rosuvastatin daily dose, as well as dose of other non-statin LMT (if applicable), is required to be stable from randomization up to the end of the study, unless safety reasons prompt dose reduction or discontinuation.
Patients should be on stable diet (NCEP-ATPIII TLC diet or equivalent) throughout the entire study duration from screening to the end of the study.
</td></tr>
<tr><td>STUDY POPULATION
Main selection criteria</td><td>
Inclusion criteria
• Hospitalization for ACS (ST-elevation MI, non-ST elevation MI or high-risk unstable angina) defined by:
  - Ischemic symptoms with unstable pattern, occurring at rest or minimal exertion within 72 hrs of an unscheduled hospital admission, due to presumed or proven obstructive coronary disease AND at least one of the following:
    o Elevated cardiac biomarkers, OR
    o Resting ECG changes consistent with ischemia or infarction AND additional evidence of obstructive coronary disease
• Patient lipid levels not adequately controlled at V2 (qualifying visit), despite evidence-based lipid lowering therapy (including intensive atorvastatin/rosuvastatin therapy or maximally tolerated dose of either of these 2 statins), or other non-statin LMTs. Inadequate lipid control means that patient must meet <u>at least one</u> of the following criteria at V2 to qualify:
  - LDL-C ≥ 70 mg/dL (≥1.81 mmol/L), or
  - ApoB ≥ 80 mg/dL (≥ 0.8 g/L), or
  - non-HDL-C ≥ 100 mg/dL (≥2.59 mmol/L)
Exclusion criteria
• All of the 3 following criteria are concomitantly present at the qualifying visit (V2):
  - LDL-C <70 mg/dL (<1.81 mmol/L), and
  - ApoB < 80 mg/dL (< 0.8 g/L), and
  - non-HDL-C <100 mg/dL (<2.59 mmol/L)
</td></tr>
</table>

	NOTE: *If not all 3 but only 1 or 2 criteria are present then the patient may qualify.*<br>• Age < 40 years<br>• Patients in whom the qualifying index ACS event occurred less than 4 weeks (28 days) or more than 52 weeks (+ 5 days) prior to randomization visit (V3)<br>• Not on stable LMT doses (statin and/or non-statin LMT) for at least 2 weeks prior to qualifying visit (V2)<br>• Uncontrolled hypertension (multiple readings with SBP > 180 mmHg or DBP > 110 mmHg) at V3<br>• New York Heart Association Class III or IV congestive heart failure persisting despite treatment or if measured LVEF <25%<br>• Known history of hemorrhagic stroke<br>• Fasting serum triglycerides (TG) >400 mg/dL (>4.52 mmol/L) prior to randomization<br>• New ACS event occurring within 2 weeks prior to the randomization Visit (V3)<br>• Coronary revascularization (PCI or CABG) planned after randomization and/or performed within 2 weeks prior to the randomization Visit (V3)
**Total expected number of patients**	Approximately 18,600 patients should be randomized (9,300 patients / group).<br>Approximately 1400 sites
**STUDY TREATMENT(s)**<br>**Investigational medicinal product(s)**<br><br>**Formulation**	Alirocumab<br><br>Sterile alirocumab drug product supplied at a concentration of 75 mg/mL or 150 mg/mL in histidine, pH 6.0, polysorbate 20, and sucrose
**Route(s) of administration**	Subcutaneous (SC) injection in the abdomen, thigh or outer area of upper arm
**Dose regimen**	75 mg every 2 weeks **OR**<br>75 mg every 2 weeks up to Month 2 followed by 150 mg every 2 weeks at Month 2 onwards (with criteria for up-titration and down-titration as indicated above)
**INJECTION FOR TRAINING AND PLACEBO COMPARATOR**<br>**Investigational medicinal product(s) (if applicable)**<br><br>**Formulation**	Placebo<br><br>Same formulation as alirocumab without the addition of protein.
**Route(s) of administration**	Subcutaneous (SC) injection in the abdomen, thigh or outer area of upper arm.

**ENDPOINT(S)**	**Primary endpoint**<br>• Time from randomization to first occurrence of one of the following Clinical Events, as determined by the CEC:<br>- CHD death<br>- Any non-fatal MI<br>- Fatal and non-fatal ischemic stroke<br>- Unstable angina requiring hospitalization<br>**Main Secondary Efficacy Endpoint(s):**<br>• Time from randomization to first occurrence of any CHD event (major CHD event, unstable angina requiring hospitalization, ischemia-driven coronary revascularization procedure)<br>• Time from randomization to first occurrence of any major CHD event (CHD death, non-fatal MI)<br>• Time from randomization to first occurrence of any CV event defined as follows: any non-fatal CHD event, any CV death, and non-fatal ischemic stroke<br>• Time from randomization to first occurrence of all-cause mortality, non-fatal MI, non-fatal ischemic stroke<br>• Time from randomization to death (all-cause mortality)<br>**Other Secondary Efficacy Endpoint(s):**<br>• Components of the primary end point considered individually: CHD death, or non-fatal MI, or fatal and non-fatal ischemic stroke, unstable angina requiring hospitalization<br>• Ischemia-driven coronary revascularization procedure<br>• Congestive heart failure requiring hospitalization<br>**Safety Endpoint(s):**<br>• Safety endpoints: all adverse events, heart rate and blood pressure, hematology and biochemistry assessments<br>**Other Endpoint(s):**<br>• Anti- alirocumab antibodies assessed throughout the study<br>• The percent change in calculated LDL-C, in ApoB and non HDL-C

**ASSESSMENT SCHEDULE**	**Visits scheduled during the run-in period:**<br>Please refer to Run-In Period section under 'Study Design'<br><br>**Visits schedule during the double-blind treatment period:**<br>• Clinic/On-site visits:<br>- For the first year: visit at Month1, Month 2, Month 4 and then every 4 months (Month 8, Month 12)<br>- For the second year: visit every 4 months<br>- For the subsequent years: visit every 6 months up to the end of the study<br>**Contacts schedule during the double-blind treatment period:**<br>• A phone call or contact via internet (as permitted by institutional privacy policies) should occur in between clinic visits throughout the study with the goal of maintaining a contact with the patient about every 2 months during first 24 months, and about every 3 months thereafter.
**STATISTICAL CONSIDERATIONS**	**Sample size determination:**<br>Based on the targeted population for the study, a Kaplan-Meier event rate in placebo group of 11.4% at 48 months (3.8% at 12 months, 6.4% at 24 months, 9.0% at 36 months) is assumed. With a 40-month recruitment period, a 64-month total study duration, 9300 patients per group (total 18,600), and 1,613 patients experiencing at least one primary endpoint event, the study has 90% power (one-sided Logrank test at the overall 0.025 alpha level) assuming a 15% risk reduction associated with alirocumab treatment. The sample size calculation takes into account two interim analyses.<br>**Analysis Populations:**<br>Randomized population includes any patients who have been allocated to a randomized treatment regardless of whether the treatment kit was used or not.<br>Efficacy analyses will be performed on the intention-to-treat (ITT) population, consisting of all randomized patients. Patients in the ITT population will be analyzed according to the treatment group allocated by randomization.<br>The safety population consists of the randomized population who actually received at least one dose or partial dose of IMP analyzed according to the treatment actually received.<br>**Primary Analysis:**<br>The primary endpoint will be compared between treatment groups by a log-rank test, stratified by region (North America, South America, Western Europe, Eastern Europe, Asia, other region). The distribution will be estimated by treatment group with Kaplan-Meier methodology. Treatment hazard ratios (HRs) for the primary endpoint will be estimated from Cox regression models stratified by region.<br><br>**Analysis of the main secondary endpoints**<br>A hierarchical procedure will be used to control the type I error and handle multiple endpoints. If the primary endpoint analysis is significant (at the 0.0001 one-sided alpha level at the second interim analysis or at the 0.0249 one-sided alpha level at the final analysis), main secondary efficacy endpoints will be tested sequentially, using the order defined in section "Primary and secondary

	endpoints".<br>Secondary endpoints will be analyzed in the ITT population using the same statistical methodology as for the primary endpoint (time-to-event analysis).<br>**Safety analysis**<br>Safety analysis (adverse events, laboratory, and vital signs) will be descriptive, based on the safety population. The safety analysis will focus on the Treatment Emergent Adverse Event (TEAE) period. This period is defined as the time from the first to the last dose of double-blind IMP + 70 days (10 weeks).<br>**Interim analyses:**<br>Patients will be followed until 1,613 patients experience at least one primary endpoint event or for approximately 24 months after the closing of randomization ex-China, whichever comes last.<br><br>Interim analyses for futility will be performed, under the supervision of the CV DMC, when 50% and 75% of events have occurred. An interim analysis for efficacy will be performed when 75% of events have occurred. Control of the type I and type II error will be ensured using gamma (-5) spending function for type II error (futility) and Gamma (-22) for type I error (efficacy). Stopping rules details are further described in Section 11.5.
**DURATION OF STUDY PERIOD (per patient)**	The duration of the run-in period (V1 to V3) should be between 2 weeks (14 days) and 16 weeks (+ 5 days). Randomization must occur no earlier than 4 weeks (28 days) and no later than 52 weeks (+ 5 days) after the index ACS event.<br>The double-blind treatment period will continue for 24 months after the closing of randomization ex-China (last date for randomization in all countries except China) or until the target number of events (1613) is reached whichever comes last. The corresponding estimated study duration is 64 months (as described in the sample size considerations).<br>All patients, even those who have achieved an endpoint or prematurely discontinued study treatment, will be followed from randomization until the common study end date.

1 FLOW CHARTS

1.1 GRAPHICAL STUDY DESIGN

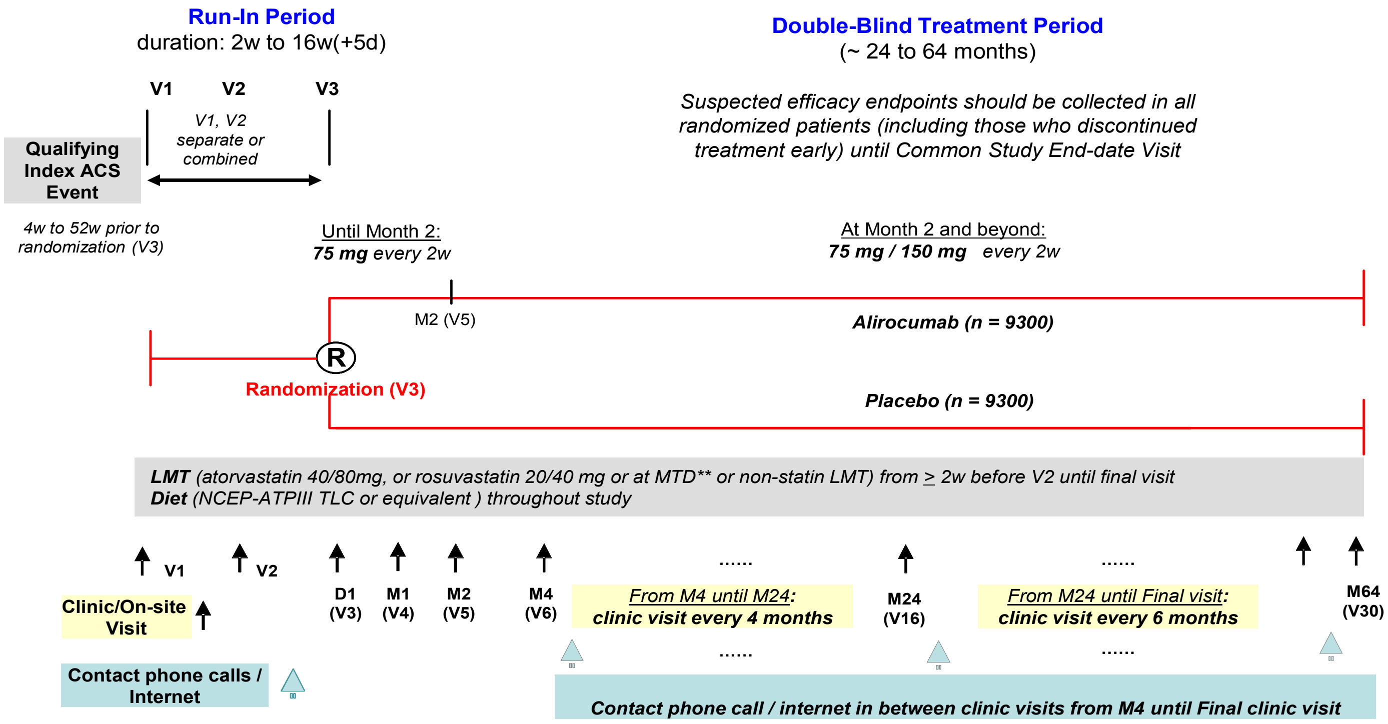

Run-In Period (duration between 2w and 16w+5d)
- **V1:** earliest is on day of ACS; latest is 50w post ACS (if V1, V2 separate) or 50w (+5d) post ACS (if combined V1/V2)
- **V2:** collection qualifying lipid labs after receiving lipid-modifying therapy (LMT) that is statin-intensive (atorvastatin 40/80mg, or rosuvastatin 20/40 mg or at max. tolerated dose), optimized for long-term chronic use (with addition of non-statin LMT at Inv. Discretion) and well tolerated after ≥ 2w of stable LMT dose
- **V3**: earliest is 4w and latest is 52w(+5d) post-index ACS

After randomization
- Final clinic visit (Common Study End Date Visit, V30 M64) should be completed in all randomized patients (regardless of whether they are still on treatment or had discontinued treatment early) and should occur between Common Study End Date (CSED) and CSED+30days.

ACS: acute coronary syndrome, LMT: lipid-modifying therapy, MTD: maximal tolerated dose

1.2 STUDY FLOW CHART

1.2.1 On-site/Clinic visits during the study

	Screening (run-in period)		Double-blind period										
**On-site / Clinic Visit**	V1 (or combined V1/V2)	V2[b]	V3	V4	V5	V6	V8	V10	V12/V14/V18/V22/V26	V16/V20/V24/V28	V30 [m]		V70 [n]
**Week(W) / Month(M)** [a]	≥ 2w prior to V3 AND 0 to 50w post index ACS	W-2 After ≥2w required LMT	M0 (D1) 4 to 52w post index ACS	M1	M2	M4	M8	M12	M16/M20/M30/M42/M54	M24/M36/M48/M60	M64 Final visit (common study end date visit)		Early end of treatment visit
											Study completers	Prematurely discontinued patients [m]	
**Design:**													
Informed Consent / Patient Demography	X												
Inclusion / Exclusion Criteria	X	X	X										
Medical / surgical history (incl. relevant family history e.g. allergy), alcohol / smoking habits	X												
Prior Medication History[c]	X												
Physical Examination	X		X					X		X	X		X
Body weight	X		X			X	X	X	X	X	X		X
Height	X												
Randomization			X										

	**Screening (run-in period)**		**Double-blind period**										
**On-site / Clinic Visit**	V1 (or combined V1/V2)	V2[b]	V3	V4	V5	V6	V8	V10	V12/V14/V18/V22/V26	V16/V20/V24/V28	V30[m]		V70[n]
**Week(W) / Month(M)**[a]	≥ 2w prior to V3 AND 0 to 50w post index ACS	W-2 After ≥2w required LMT	M0 (D1) 4 to 52w post index ACS	M1	M2	M4	M8	M12	M16/M20/M30/M42/M54	M24/M36/M48/M60	M64 Final visit (common study end date visit)		Early end of treatment visit
											Study completers	Prematurely discontinued patients[m]	
Patient diary dispensation/ review / collection[d]			X	X	X	X	X	X	X	X	X		X
IVRS/IWRS contact	X	X	X		X	X	X	X	X	X	X	X	X
**Treatment:**													
Injection training	X[e]	X[e]	X[f]										
Double Blind Investigational Medicinal Product (IMP) kit dispensation			X		X	X	X	X	X	X			
Compliance check of IMP (review patient diary and treatment kit) and data collection on IMP administration				X	X	X	X	X	X	X	X		X
Compliance check for atorvastatin, rosuvastatin, ± other LMTs		X	X	X	X	X	X	X	X	X	X	X (statin, ezetimibe only)	X
Concomitant Medication[c]	X	X	X	X	X	X	X	X	X	X	X		X

	**Screening (run-in period)**		**Double-blind period**										
**On-site / Clinic Visit**	V1 (or combined V1/V2)	V2[b]	V3	V4	V5	V6	V8	V10	V12/V14/V18/V22/V26	V16/V20/V24/V28	V30[m]		V70[n]
**Week(W) / Month(M)**[a]	≥ 2w prior to V3 <u>AND</u> 0 to 50w post index ACS	W-2<br>After ≥2w required LMT	M0 (D1) 4 to 52w post index ACS	M1	M2	M4	M8	M12	M16/M20/M30/M42/M54	M24/M36/M48/M60	M64 Final visit (common study end date visit)		Early end of treatment visit
											Study completers	Prematurely discontinued patients[m]	
**<u>Vital signs:</u>**													
Heart rate, blood pressure	X	X	X	X	X	X	X	X	X	X	X		X
**<u>Efficacy Endpoints</u>** (primary and secondary) and **<u>Cardiovascular Events of Interest (other than efficacy endpoints)</u>**													
Update patient contact information (incl. patient's family, patient's GP/cardiologist)	X	X	X	X	X	X	X	X	X	X			X
Check patient card (with mention of study participation and site contact information)			X	X	X	X	X	X	X	X			X
Collect information on suspected efficacy endpoints				X	X	X	X	X	X	X	X	X	X

	**Screening (run-in period)**		**Double-blind period**										
**On-site / Clinic Visit**	V1 (or combined V1/V2)	V2[b]	V3	V4	V5	V6	V8	V10	V12/V14/V18/V22/V26	V16/V20/V24/V28	V30[m]		V70[n]
**Week(W) / Month(M)**[a]	≥ 2w prior to V3 AND 0 to 50w post index ACS	W-2 After ≥2w required LMT	M0 (D1) 4 to 52w post index ACS	M1	M2	M4	M8	M12	M16/M20/M30/M42/M54	M24/M36/M48/M60	M64 Final visit (common study end date visit)		Early end of treatment visit
											Study completers	Prematurely discontinued patients[m]	
Collect cardiovascular events of interest other than efficacy endpoints (related to peripheral arterial disease, venous thromboembolic events)				X	X	X	X	X	X	X	X	X	X
**Safety:**													
AE /SAE recording	X	X	X	X	X	X	X	X	X	X	X	X (related and/or serious AE)	X
12-lead ECG			X								X		X
**Laboratory Testing / Efficacy:**													
TC, LDL-C, HDL-C, TG, non-HDL-C	X	X	X	X	X	X	X	X	X	X	X	X	X
ApoB	X	X	X			X		X		X (M24 only)	X	X	X
ApoA-1, ratio ApoB/ApoA-1, Lp (a)			X			X		X			X		X

	**Screening (run-in period)**		**Double-blind period**										
**On-site / Clinic Visit**	V1 (or combined V1/V2)	V2[b]	V3	V4	V5	V6	V8	V10	V12/V14/V18/V22/V26	V16/V20/V24/V28	V30[m]		V70[n]
**Week(W) / Month(M)**[a]	≥ 2w prior to V3 AND 0 to 50w post index ACS	W-2 After ≥2w required LMT	M0 (D1) 4 to 52w post index ACS	M1	M2	M4	M8	M12	M16/M20/M30/M42/M54	M24/M36/M48/M60	M64 Final visit (common study end date visit)		Early end of treatment visit
											Study completers	Prematurely discontinued patients[m]	
**Laboratory Testing / Safety:**													
Hematology and chemistry[g]	X		X					X		X	X		X
Creatine phosphokinase (CPK)	X		X	X		X		X	X	X	X		X
Liver panel[h]	X		X	X		X		X	X	X	X		X
Hepatitis B surface antigen	X												
Hepatitis C antibody[h]	X		X								X		X
Serum pregnancy test[h]	X												
Urine pregnancy test[h]			X			X	X	X	X	X	X		X
Urinalysis (at selected sites only) [h]			X					X		X	X		X

	**Screening (run-in period)**		**Double-blind period**										
**On-site / Clinic Visit**	V1 (or combined V1/V2)	V2[b]	V3	V4	V5	V6	V8	V10	V12/V14/V18/V22/V26	V16/V20/V24/V28	V30[m]		V70[n]
**Week(W) / Month(M)**[a]	≥ 2w prior to V3 AND 0 to 50w post index ACS	W-2 After ≥2w required LMT	M0 (D1) 4 to 52w post index ACS	M1	M2	M4	M8	M12	M16/M20/M30/M42/M54	M24/M36/M48/M60	M64 Final visit (common study end date visit)		Early end of treatment visit
											Study completers	Prematurely discontinued patients[m]	
**Laboratory Testing / Other:**													
HbA_{1c}	X		X					X		X	X	X	X
High sensitivity C-reactive protein (hs-CRP)			X			X		X			X		X
Anti- alirocumab antibodies			X		X	X		X		X	X	X	X
Library samples[j]			X			X		X			X		X
**Genomics**													
Genomics consent (optional)			X										
Collect specimen (if genomics consent)[k]			X										
**Quality of Life Variables**													
EQ-5D patient questionnaire[l]			X		X	X	X	X	X	X	X		X

a Window period for visits: randomization visit (V3) should be performed no earlier than 4w(28d) post-index ACS, and no later than 52w(+5d) post index ACS, window at Months 1 and 2 is ± 7 days, and for all other subsequent visits it is ± 14 days (in case of major IMP issue, please refer to Section 8.9).

b V2 is main visit for collection of qualifying lipid labs (sent to Central Labs) following ≥ 2w of well tolerated, stable required LMT (i.e. statin-intensive, and optimized for long-term chronic use with addition of non-statin LMT, at Investigator's discretion). If required LMT treatment was already administered prior to V1 for ≥ 2w, V1 and V2 visits can be combined as one visit. Otherwise, interval between V1 and V2 should be ≥2w. After 2 to 5 days, V2 lab results are obtained, and patient may be randomized if all eligibility criteria are met. Overall, duration of run-in period (between V1 and V3, or between combined V1/V2 and V3) should be between 2w (14 days) and 16w(+5 days). V3 (randomization) should occur no earlier than 4w (28 days) and no later than 52w (+5days) post ACS index event. An optional visit (V2b) with repeat Central Laboratory assessments may be scheduled during run-in after V2 in the following scenario: V2 labs are not met, however patient was kept in run-in period after 'failed' V2 and subsequently develops intolerance to statin leading to lower statin dose; patient may then be retested with lower statin dose. A V2b visit should be performed if V2 labs are met but statin dose (or non-statin LMT regimen) was subsequently increased, after V2.

c Prior medication: prior to screening visit V1 and randomization (IMP administration); only restrictions pertain to fibrates (other than fenofibrate, fenofibric acid) and statins (other than atorvastatin, rosuvastatin) which should be discontinued at V1. Concomitant medication: received concomitantly to the IMP, from first IMP to the end of treatment + 70 days.

d Along with kit dispensation, the treatment administration package should be given as well as the IMP diary and injection instruction manual, as needed.

e At least 2 training injections are required on 2 different occasions. These can take place at V1, V2, Combined V1/V2, or V3 at the discretion of the study site. First training injection (with placebo) must be done before V3. The initial IMP double-blind injection (active or placebo) performed at V3 may serve as second training injection. If needed, additional training injections (with placebo) are available

f Injection is performed at randomization Visit Month 0/Day 1 on site with double-blind study treatment kit allocated by IVRS (can serve as training injection).

g Hematology includes: complete blood cell count (CBC) including hematocrit, hemoglobin, red blood cell count, reticulocyte count, white blood cell count with differential count, and platelets (Note: reticulocyte not done at V1). Chemistry includes: glucose, sodium, potassium, chloride, bicarbonate, calcium, phosphorous, urea nitrogen, creatinine, uric acid, LDH, total protein, albumin, and γGT.

h Liver panel: ALT, AST, alkaline phosphatase (ALP), and total bilirubin. Hepatitis C antibody: In case of positive hepatitis C antibody test, a blood sample will be collected at a subsequent visit and sent to Covance Central Lab for further confirmatory testing

i Pregnancy tests (serum, urine) in Women of Child Bearing Potential (WOCBP) only. Urinalysis to be performed only at selected sites (see Section 10).

j Library samples should be collected, as permitted by local regulatory policies. They may be stored for up to 10 years or as permitted by local regulatory policies, whichever is shorter, for exploratory research of PCSK9 levels, PCSK9 function, effect(s) of PCSK9 inhibition with a monoclonal antibody, lipoprotein sub-fraction, inflammation, and cardiovascular risk markers (eg, lipoprotein–associated phospholipase A2).

k If blood sample not collected at randomization, could be collected at any time during the study.

l EQ-5D patient questionnaire will only be administered in patients still on treatment, for prematurely (permanently) discontinued patients the last administration will be done at the early end of treatment visit.

m Common study end date (CSED) will occur 24 months after the closing of randomization ex-China or after the target number of events (1613) is reached whichever comes last. A final clinic visit V30 (CSED visit, Month 64) should be completed within 30 days of CSED for all randomized patients regardless of the compliance with study treatment (see Section 10.1.5.1.4). Reduced assessments in 'Prematurely discontinued patients' column apply only if V30 occurs more than 6 months after V70 (see Section 10.3.4).

n For patients who prematurely discontinue blinded study treatment (IMP): as a general rule, any IMP treatment discontinuation should be initially considered temporary, and Investigator should make best effort to resume IMP treatment as early as practically possible, after several weeks or months (pending there are no safety concerns), and perform all study visits and assessments as usual. Investigator is strongly encouraged to discuss with monitoring team before considering any treatment discontinuation as permanent. A discussion with National Coordinator may occur regarding other possible options If and when a treatment discontinuation is considered 'permanent', an extra visit (early end of treatment visit V70) should be performed as soon as practically possible (within 1 month) after IMP discontinuation is considered permanent by Investigator. Such patients (with premature permanent treatment discontinuation) must continue to remain in the study and should return for all study visits until the common study end date (final visit V30). Complete usual study assessments should be performed for 6 months following V70. Thereafter, assessments are reduced to collection of suspected efficacy endpoints, cardiovascular events of interest other than efficacy endpoints (peripheral arterial disease, venous thromboembolic events – see Section 9.3.7), related and/or serious AEs , selected concomitant medications (statins, ezetimibe) and selected labs (anti- alirocumab antibodies, lipid panel, ApoB, HbA1c) (See Section 10.3.4).

1.2.2 Contacts (phone calls or contacts via internet) during the study

**Month (M)**[a]	**M6/ M10/ M14/ M18/ M22/ M27/ M33/ M39/ M45/ M51/ M57/ M62**
**Visit**	V7/ V9/ V11/ V13/ V15/ V17/ V19/ V21/ V23/ V25/ V27/ V29
**Phone Call**	☎ or Internet contact [b]
Update patient contact information (incl.patient's family, patient's GP/cardiologist) [d]	X
Review compliance with statins, other LMTs [d]	X
Collect information on IMP administration (and remind patient to complete diary)	X
Collect information on possible occurrence of efficacy endpoints (and remind patient to call site in case of hospitalization and not wait until next visit) [d]	X
Reminders [c]	X

a Although flexibility is allowed in the timing of phone call in between the on-site/clinic visits, it should be scheduled with the goal of maintaining a contact with the patient about every 2 months during first 24 months, and about every 3 months thereafter. Having no phone or e mail contact with a patient for a prolonged period (>4 months) should be avoided as much as possible. Some additional instructions for scheduling phone call may be provided at time of interim analysis.

b In addition, an optional service will be made available for sites to send via SMS/text messages appointment and injection reminders to any patient who wishes to and is able to receive them. These contacts will conform with privacy regulations at each site.

c Reminders as applicable for IMP administration schedule, timing of next appointments, fasting conditions for next lab assessments, bring the diary, and used and unused kits at the next study site visit.

d In patients who have permanently discontinued treatment, contacts will include: update of patient contact information (incl. family and patient's GP/cardiologist), collection of suspected efficacy endpoints during entire study until common study end-date, and review of compliance with statin and other LMT for 6 months following V70, and with statin and ezetimibe thereafter.

2 TABLE OF CONTENTS

AMENDED CLINICAL TRIAL PROTOCOL NO. 11 ..... **1**

1 FLOW CHARTS **13**

1.1 GRAPHICAL STUDY DESIGN 13

1.2 STUDY FLOW CHART 14

1.2.1 On-site/Clinic visits during the study 14

1.2.2 Contacts (phone calls or contacts via internet) during the study 21

2 TABLE OF CONTENTS **22**

3 LIST OF ABBREVIATIONS **28**

4 INTRODUCTION AND RATIONALE **31**

5 STUDY OBJECTIVES **38**

5.1 PRIMARY 38

5.2 SECONDARY 38

6 STUDY DESIGN **39**

6.1 DESCRIPTION OF THE PROTOCOL 40

6.1.1 Run-in period 41

6.1.1.1 Overview of run-in period 41

6.1.1.2 Visit and Time Intervals during Run-In Period 42

6.1.1.3 Required background LMT during the run-in period 44

6.1.2 Coronary revascularization strategy 47

6.1.3 Screen failures and Rescreening 47

6.1.3.1 Screen failure 47

6.1.3.2 Rescreening 47

6.1.4 Double-blind treatment period 48

6.2 DURATION OF STUDY PARTICIPATION. 50

6.2.1 Duration of study participation for each patient 50

6.2.2 Determination of end of clinical trial (all patients) - Common Study End Date 50

6.3 INTERIM ANALYSIS 50

6.4 STUDY COMMITTEES 51

7 SELECTION OF PATIENTS **53**

7.1 INCLUSION CRITERIA 53
7.2 EXCLUSION CRITERIA 54
7.2.1 Exclusion criteria related to study methodology 54
7.2.2 Exclusion criteria related to the background therapy 56
7.2.3 Exclusion criteria related to the current knowledge of alirocumab 56
8 STUDY TREATMENTS 57
8.1 INVESTIGATIONAL MEDICINAL PRODUCT(S) 57
8.1.1 Route and method of administration 57
8.1.2 Timing of administration 58
8.2 NON-INVESTIGATIONAL MEDICINAL PRODUCT(S) 59
8.3 BLINDING PROCEDURES 59
8.3.1 Methods of blinding 59
8.3.2 Lipid parameters 59
8.3.3 Anti-alirocumab antibodies 61
8.3.4 Committees 61
8.3.5 Data Analysis 61
8.3.6 Randomization code breaking during the study 62
8.4 METHOD OF ASSIGNING PATIENTS TO TREATMENT GROUP 62
8.5 PACKAGING AND LABELING 63
8.6 STORAGE CONDITIONS AND SHELF LIFE 64
8.7 RESPONSIBILITIES 64
8.7.1 Treatment accountability and compliance 65
8.7.2 Return and/or destruction of treatments 65
8.8 CONCOMITANT MEDICATION 66
8.8.1 Background Lipid-Modifying Therapy 66
8.8.2 Other Concomitant Medications 67
8.9 MITIGATION PLAN IN CASE OF IMP ISSUE 68
9 ASSESSMENT OF INVESTIGATIONAL MEDICINAL PRODUCT 69
9.1 EFFICACY ENDPOINTS 69
9.1.1 Primary efficacy endpoint 69
9.1.2 Secondary efficacy endpoints 69
9.1.3 Main Secondary Efficacy Endpoint(s): 69

9.1.4 Other Secondary Efficacy Endpoint(s): 70
9.1.5 Efficacy assessment methods 70
9.1.6 Definitions of components of the composite primary efficacy endpoint 70
9.1.6.1 Coronary Heart Disease (CHD) Death 70
9.1.6.2 Non-fatal myocardial infarction (MI) 72
9.1.6.3 Fatal and Non-fatal stroke 75
9.1.6.4 Unstable angina requiring hospitalization 76
9.1.7 Definitions of the secondary efficacy endpoints 77
9.1.7.1 Death 77
9.1.7.2 Ischemia-driven coronary revascularization procedure 79
9.1.7.3 Congestive Heart Failure requiring hospitalization. 80
9.2 SAFETY ENDPOINT(S): 81
9.2.1 Adverse event 82
9.2.2 Safety laboratory 82
9.2.3 Vital signs measurement 82
9.2.4 Electrocardiogram measurement 82
9.3 OTHER ENDPOINT(S): 83
9.3.1 Anti-alirocumab antibody assessments 83
9.3.1.1 Sampling time 83
9.3.1.2 Sampling procedure 83
9.3.1.3 Bioanalytical method 83
9.3.2 Lipid parameters 84
9.3.2.1 Endpoints 84
9.3.2.2 Assessment method 84
9.3.3 hs-CRP 84
9.3.4 HbA_{1C} 84
9.3.5 EQ-5D Patient Questionnaire 84
9.3.6 Pharmacogenomic Samples 85
9.3.7 Cardiovascular events of interest (other than efficacy endpoints) 86
10 STUDY PROCEDURES 87
10.1 VISIT SCHEDULE 91
10.1.1 Screening visit - Visit 1 (at least 2 weeks prior to randomization visit) and entry in the run-in period 91
10.1.2 Qualifying visit - Visit 2 93
10.1.3 Combined V1/V2 Visit 94
10.1.4 Optional qualifying visit - Visit 2b 94
10.1.5 Double-blind treatment period 95
10.1.5.1 Study site visits from Visit 3 (Month 0, D1) to Visit 30 (Month 64) 95
10.1.5.2 Contacts (phone calls, text messages or contacts via internet) from randomization 107

10.1.5.3 Early end of treatment visit – Visit 70 107
10.1.5.4 Common study end date visit (Final Visit) – Visit 30 (~Month 64, between CSED and CSED + 30 days) 108

10.2 DEFINITION OF SOURCE DATA 111

10.3 HANDLING OF PATIENT TEMPORARY OR PERMANENT TREATMENT DISCONTINUATION AND OF PATIENT STUDY DISCONTINUATION 112
10.3.1 Temporary treatment discontinuation with investigational medicinal product(s) 112
10.3.2 Permanent treatment discontinuation with investigational medicinal product(s) 113
10.3.3 List of criteria for permanent treatment discontinuation 113
10.3.4 Handling of patients after permanent treatment discontinuation 114
10.3.5 Procedure and consequence for patient withdrawal from study 115

10.4 OBLIGATION OF THE INVESTIGATOR REGARDING SAFETY REPORTING 116
10.4.1 Definitions of adverse events 116
10.4.1.1 Adverse event 116
10.4.1.2 Serious adverse event 116
10.4.2 Adverse event of special interest 117
10.4.3 Serious adverse events waived from expedited regulatory reporting to health authorities 117
10.4.4 General guidelines for reporting adverse events 118
10.4.5 Instructions for reporting serious adverse events 119
10.4.6 Guidelines for reporting adverse events of special interest 119
10.4.6.1 Reporting of adverse events of special interest with immediate notification 119
10.4.6.2 Reporting of adverse events of special interest without immediate notification 120
10.4.7 Guidelines for management of specific laboratory abnormalities 121
10.4.7.1 Hemoglobin decrease 122

10.5 OBLIGATIONS OF THE SPONSOR 123

10.6 SAFETY INSTRUCTIONS 123
10.6.1 Local tolerability (Local Injection Site Reactions) 123
10.6.2 Allergic adverse events (See Section 10.4.6.1 and Section 10.4.6.2) 123
10.6.2.1 Allergic adverse event with cutaneous involvement 124
10.6.2.2 Acute allergic injection reactions (Section 10.4.6.2) 124
10.6.3 Monitoring related to two consecutive LDL-C <25 mg/dL (0.65 mmol/L) 125

10.7 ADVERSE EVENTS MONITORING 125

11 STATISTICAL CONSIDERATIONS 126

11.1 DETERMINATION OF SAMPLE SIZE 126

11.2 DISPOSITION OF PATIENTS 127

11.3 ANALYSIS POPULATIONS 127

11.3.1 Efficacy populations 127
11.3.1.1 Intent–to-treat population 127
11.3.2 Safety population 128
11.3.3 Other analysis populations 128
11.4 STATISTICAL METHODS 128
11.4.1 Extent of study treatment exposure and compliance 128
11.4.1.1 Extent of investigational medicinal product exposure 129
11.4.1.2 Compliance 129
11.4.2 Analyses of efficacy endpoints 129
11.4.2.1 Analysis of primary efficacy endpoint(s) 130
11.4.2.2 Analyses of secondary efficacy endpoints 131
11.4.2.3 Multiplicity consideration 131
11.4.3 Analyses of safety data 132
11.4.3.1 Adverse events 133
11.4.3.2 Laboratory data and vital signs 134
11.4.4 Other endpoints: 134
11.4.4.1 Cardiovascular events of interest (other than efficacy endpoints) 134
11.4.4.2 Other endpoints 135
11.5 INTERIM ANALYSIS 135
12 ETHICAL AND REGULATORY STANDARDS 137
12.1 ETHICAL PRINCIPLES 137
12.2 LAWS AND REGULATIONS 137
12.3 INFORMED CONSENT 137
12.4 INSTITUTIONAL REVIEW BOARD/INDEPENDENT ETHICS COMMITTEE (IRB/IEC) 138
13 STUDY MONITORING 139
13.1 RESPONSIBILITIES OF THE INVESTIGATOR(S) 139
13.2 RESPONSIBILITIES OF THE SPONSOR 139
13.3 SOURCE DOCUMENT REQUIREMENTS 140
13.4 USE AND COMPLETION OF CASE REPORT FORMS (eCRFS) AND ADDITIONAL REQUEST 140
13.5 USE OF COMPUTERIZED SYSTEMS 140
14 ADMINISTRATIVE EXPECTATIONS 141
14.1 CURRICULUM VITAE 141
14.2 RECORD RETENTION IN STUDY SITES 141

14.3 CONFIDENTIALITY 141

14.4 PROPERTY RIGHTS 142

14.5 DATA PROTECTION 142

14.6 INSURANCE COMPENSATION 142

14.7 SPONSOR AUDITS AND INSPECTIONS BY REGULATORY AGENCIES 143

14.8 PREMATURE DISCONTINUATION OF THE STUDY OR PREMATURE CLOSE-OUT OF A SITE 143

14.8.1 Decided by the Sponsor 143

14.8.2 Decided by the Investigator 144

14.9 CLINICAL TRIAL RESULTS 144

14.10 PUBLICATIONS AND COMMUNICATIONS 144

15 CLINICAL TRIAL PROTOCOL AMENDMENTS 146

16 BIBLIOGRAPHIC REFERENCES 147

17 APPENDICES 151

APPENDIX A MONITORING AND MANAGEMENT PLAN FOR LDL-C < 25 MG/DL (0.65 MMOL/L) 152

APPENDIX B DNA STORAGE SAMPLES 154

APPENDIX C GENERAL GUIDANCE FOR THE FOLLOW-UP OF LABORATORY ABNORMALITIES BY SANOFI 156

APPENDIX D GUIDELINES FOR HEMOGLOBIN (HB) DECREASE ≥ 1.5 G/DL 162

APPENDIX E SUMMARY OF ADVERSE EVENT AND SUSPECTED EFFICACY ENDPOINT REPORTING INSTRUCTIONS 163

APPENDIX F ASSESSMENT OF LOCAL INJECTION SITE REACTIONS 168

APPENDIX G EQ-5D PATIENT QUESTIONNAIRE 169

APPENDIX H NEW YORK HEART ASSOCIATION FUNCTIONAL CLASSIFICATION 170

APPENDIX I STROKE FUNCTIONAL DISABILITY SCORE (ADAPTED FROM MODIFIED RANKIN SCALE) 171

3 LIST OF ABBREVIATIONS

ACS	Acute coronary syndrome
ADA	Anti-drug antibody
AE	Adverse event
AESI	Adverse event of special interest
ALP	Alkaline phosphatase
ALT	Alanine aminotransferase
ANCOVA	Analysis of covariance
Apo	Apolipoprotein
ARF	Acute renal failure
AST	Aspartate aminotransferase
BMI	Body mass index
BP	Blood pressure
CABG	Coronary artery bypass graft surgery
CEC	Clinical Events Committee
CHD	Coronary Heart Disease
CI	Confidence interval
CIB	Clinical Investigator's brochure
CPK	Creatine Phosphokinase
CSED	Common Study End Date
CSR	Clinical Study Report
CV	Cardiovascular
DBTP	Double Blind Treatment Period
DMC	Data Monitoring Committee
DNA	Deoxyribonucleic acid
DRF	Discrepancy resolution form
ECG	Electrocardiogram
eg	Exempli gratia = for example
e-SMS	Emergency Scientific & Medical Services
eCRF	Electronic case report form
EDTA	Ethylene diamine tetra-acetic acid
eGFR	Estimated Glomerular Filtration Rate
FH	Familial hypercholesterolemia
FPI	First patient in
FU	Follow-up
GCP	Good Clinical Practice
γGT	Gamma-glutamyl Transferase
HbA_{1c}	Glycated hemoglobin A_{1c}
HDL-C	High-density lipoprotein cholesterol
HeFH	Heterozygous familial hypercholesterolemia
HLGT	High Level Group Term
HLT	High level term

HR	Hazard ratio
hs-CRP	High-sensitivity C-reactive protein
IA	Interim Analysis
ICF	Informed consent form
ICH	International Conference on Harmonization
ie	Id est = that is
IEC	Independent ethics committee
IMP	Investigational Medicinal Product
IRB	Institutional review board
ITT	Intent-to-treat
IV	Intravenous
IVRS	Interactive Voice Response System
IWRS	Interactive Web Response System
LDH	Lactate dehydrogenase
LDL-C	Low-density lipoprotein cholesterol
LDL-R	Low-density lipoprotein receptor
LLN	Lower limit of normal range
LMT	Lipid modifying therapy
Lp(a)	Lipoprotein a
MDRD	Modification of Diet in Renal Disease
MedDRA	Medical Dictionary for Regulatory Activities
MI	Myocardial infarction
mmHg	Millimeter of mercury
NCEPATPIII	National Cholesterol Education Program Adult Treatment Panel III
NYHA	New York heart association
NIMP	Non-Investigational Medicinal Product
PAD	Peripheral Arterial Disease
PCI	Percutaneous coronary intervention
PCSA	Potentially Clinically Significant Abnormality
PCSK9	Proprotein convertase subtilisin/kexin type 9
PD	Pharmacodynamics
PK	Pharmacokinetics
PT	Preferred term
PTC	Product technical complaint
Q2W	every 2 weeks
SAE	Serious Adverse Event
SAP	Statistical analysis plan
SC	Subcutaneous
SD	Standard deviation
SNP	Single nucleotide polymorphisms
SOC	System organ class
SUSAR	Suspected Unexpected Serious Adverse Reaction
TOTAL-C	Total cholesterol
TEAE	Treatment Emergent Adverse Event
TG	Triglycerides

TLC	Therapeutic lifestyle changes
ULN	Upper limit of normal range
WBC	White blood cell
WOCBP	Women of childbearing potential

(electronic 1.0)

4 INTRODUCTION AND RATIONALE

Alirocumab is a fully human monoclonal antibody that binds PCSK9. All relevant information concerning the compound is available in the latest version of the Clinical Investigator's Brochure (CIB) (1).

Alirocumab is also referred to as SAR236557/REGN727. However, for this study protocol ODYSSEY Outcomes (EFC11570), it will be referred to as alirocumab.

Patients with recent acute coronary syndrome (ACS) are at very high risk for suffering recurrent coronary events in the near term. In approximately 10% of patients with ACS, cardiovascular death, recurrent myocardial infarction (MI), or stroke, occur within 1 year (2). Based on the results of large clinical trials, early intensive statin therapy has become formally endorsed as a treatment recommendation (3) (4) for patients with ACS (5). The use of high-dose statins has been largely demonstrated to be safe and well tolerated (6).

Both epidemiological and pharmacological intervention trials have demonstrated a strong and linear relationship between the levels of low-density lipoprotein cholesterol (LDL-C) and cardiovascular (CV) events. Three of the most recent statin trials, the TNT trial (7), the PROVE-IT trial (8), and the JUPITER trial (9), have provided new information on the relationship between low levels of LDL-C and CV event rates, with demonstration that treatment of LDL-C to a mean level of 77 mg/dL, 62 mg/dL, or 55 mg/dL was associated with a greater reduction in CV events.

The overall results of these trials have helped to demonstrate the continued linear relationship between LDL-C and CV events to these levels and to support the establishment of 70 mg/dL as a treatment goal for high-risk patients (10) (11). The lack of a demonstrated existing threshold or plateau between LDL-C and CV risk from these studies begs the question that even further reductions beyond the 55-77 mg/dL observed in these trials could provide additional benefits in CV event reduction. In the above three trials post-hoc analyses were conducted analyzing both the efficacy (in regards to CV event rates) and safety of achieving LDL-C levels at the lower end of the treatment spectrum.

- The PROVE-IT/TIMI-22 trial examined patients with LDL-C <100 mg/dL in the atorvastatin arm and found a lower rate of CV events in the patients with achieved LDL-C levels in the <40 mg/dL and the 40-60 mg/dL groups than those patients with higher achieved LDL-C levels (12).
- In the TNT trial, patients in the lowest quartile of achieved LDL-C had value <64 mg/dL and a mean LDL-C level of 54 mg/dL as compared to means of 70, 83, 97 and 122 mg/dL in the remaining quintiles. Within these quintiles, there was a strong and significant relationship between the lower achieved LDL-C levels and lower rates of major CV events (p<0.0001) (13).

- In the JUPITER trial, the investigators split the patients in the rosuvastatin group into two cohorts of achieved LDL-C <50 mg/dL (n=4154) and >50 mg/dL (n=4000) and compared them to the placebo group. Those that had achieved LDL-C <50 mg/dL demonstrated significantly lower rates of CV events than either of the other two groups (14).

In none of these three analyses there was evidence of an adverse safety signal observed with patients that achieved these lower levels of LDL-C. A recent communication compared patients who achieved a LDL-C ≤30 mg/dL (n=621) versus those with LDL-C >30 mg/dL (n=7533) (15). No differences in overall TEAEs and many other specific AEs were observed, with the exception of a greater incidence in insomnia (1.6/100 PY versus. 1.2/100 PY; p=0.031) and hematuria (1.8 /100 PY versus. 1.1/100 PY; p=0.0007) among patients with LDL-C ≤30 mg/dL. The rate of clinically relevant declines in eGFR (≥30%) tended to be lower among patients with lower reduction in LDL-C.

Similar findings have been observed in a patient-level meta-analysis conducted by the Cholesterol Trialists Treatment Collaboration. This examination of 26 large statin CV event trials encompassing approximately 170,000 patients has demonstrated that 20% reduction in major CV events can be derived for every 1 mmol/L (38.6 mg/dl) reduction in LDL-C, even in patients whose starting LDL-C levels are less than 2 mmol/L (77 mg/dl) (16). This meta-analysis has also demonstrated no significant safety risks with long-term cholesterol reduction treatment, including cancer (17).

These data provide strong support for the concept that high risk patients may derive further benefits of reductions in LDL-C to levels <50 mg/dl (1.29 mmol/L). A number of lipid and cardiovascular experts have begun to consider lower LDL-C values as the ideal level for humans with the concept that LDL-C levels of 30-50 mg/dL (0.78-1.29 mmol/L) represent the physiological ideal. This is based upon the findings from both the observed LDL-C levels in human newborns as well as in humans with more primitive/paleolithic lifestyles (18) (19) (20).

However, many high CV risk patients cannot achieve such levels with currently available lipid-lowering drugs. Furthermore, a significant number of high-risk patients even fail to achieve their recommended LDL-C target levels (21) (22) and most CV events are actually not prevented, leaving a substantial “residual risk” for patients and thus additional pharmacologic therapies for the prevention of coronary heart disease (CHD) remains essential, particularly for high-risk patients with ACS.

Introduction to proprotein convertase subtilisin kexin type 9 (PCSK9):

Proprotein convertase subtilisin kexin type 9 (PCSK9) belongs to the subtilisin family of serine proteases and is highly expressed in the liver. PCSK9 is involved in regulating the levels of the low-density lipoprotein receptor (LDL-R) protein (23) (24). Once PCSK9 is secreted into plasma it directly binds to the LDL-R and promotes its degradation. The increased degradation of LDLRs leads to a reduced LDL-C removal, and therefore higher LDL-C circulating levels. Experiments with mice have shown that increasing PCSK9 protein levels decreases levels of LDL-R protein in the liver while PCSK9 knockout mice have increased levels of LDL-R in the liver (25) (26). In humans, PCSK9 mutations have been identified: the gain-of-function mutations are rare and cause an autosomal dominant form of severe hypercholesterolemia and premature CHD, whereas

loss-of-function mutations are more common and are associated with reduced plasma levels of LDL-C and protection from CHD (27) (28).

Therefore blocking PCSK9 binding to the LDL-R can potentially benefit patients with hypercholesterolemia by decreasing their plasma LDL-C levels. In addition, PCSK9 messenger ribonucleic acid (mRNA) and protein levels are increased in response to statins, potentially attenuating their cholesterol-lowering effect (29).

Summary of selected clinical studies with alirocumab:

<u>Phase 1 studies</u>

Three Phase 1 studies have been conducted with alirocumab and evaluated the safety, tolerability and pharmacokinetics/pharmacodynamics (PK/PD) profile. Two studies were single dose administration (R727-CL-0902 study with intravenous (IV) administration of doses from 0.3 to 12 mg/kg and R727-CL-0904 study with SC administration of doses from 50 to 250 mg) conducted in healthy volunteers with LDL-C >100 mg/dL for whom statin therapy was not indicated. The third study (R727-CL-1001 study) was conducted in hypercholesterolemic patients (familial or non-familial) with single to multiple subcutaneous (SC) administration of 50 mg, 100 mg, 150 mg and 200 mg either as add-on to stable doses of atorvastatin from 10 to 40 mg/day or as monotherapy.

Results of these Phase 1 studies showed that alirocumab administered to healthy volunteers and patients either by IV or SC administration was generally well tolerated at all doses; treatment emergent adverse events (TEAEs) did not display a dose relationship. No pattern of adverse events related to the drug was identified. In all these Phase 1 studies, administration of alirocumab induced rapid, substantial, and sustained reductions from baseline in LDL-C, up to 60%. The magnitude and duration of these reductions were positively related to the dose administered. It should also be noted that in the R727-CL-1001 study, results were similar in the familial and non-familial hypercholesterolemic patients.

Overall, a total of 109 subjects were exposed to at least one dose of alirocumab in these three Phases 1 studies.

<u>Phase 2 studies:</u>

Three Phase 2 studies have been conducted:

- Two dose / dose regimen finding studies (DFI11565 and R727-CL-1003) with the main objective to assess, over a 12 week-treatment duration, the effects on LDL-C level reduction of several doses of alirocumab and 2 dose-regimens (50 mg, 100 mg and 150 mg every 2 weeks (Q2W), and 200 mg and 300 mg every 4 weeks (Q4W) for the DFI11565 study and 150 mg Q2W, 150 mg, 200 mg, and 300 mg Q4W for the R727-CL-1003 study). DFI11565 was conducted in hypercholesterolemic patients with elevated LDL-C (≥100 mg/dL or 2.59 mmol/L) despite stable atorvastatin therapy. R727-CL-1003 study was conducted in patients with heterozygous familial hypercholesterolemia (heFH) and with elevated LDLC (≥100 mg/dL or 2.59 mmol/L) despite their current lipid lowering therapy (statin ± ezetimibe).

- The third Phase 2 study (DFI11566) aimed to evaluate in patients with hypercholesterolemia the efficacy and safety of the co-administration of alirocumab 150 mg every 2 weeks and a high daily dose of atorvastatin (80 mg) in comparison to the co-administration of placebo and this high daily dose of atorvastatin (80 mg), in patients previously receiving a stable dose of atorvastatin 10 mg. This treatment scheme is anticipated to be used when a rapid decrease in LDLC level is needed, eg after an acute coronary syndrome.

Overall, a total of 274 patients were exposed to at least one dose of alirocumab in these three Phases 2 studies.

Efficacy results:

In both dose finding studies, statistically significant decreases in percent change from baseline in LDL-C at 12 weeks were observed in all alirocumab groups compared to the placebo group. In the DFI11565 study for the Q2W dose regimen, the greatest decrease was seen in the 150 mg Q2W group (-72.4%) compared with a small decrease in the placebo group (-5.1%) (LS mean difference versus placebo of -67.3%; p<0.0001). The decreases observed with the doses administered Q2W were maintained from the first injection throughout the study and more particularly throughout the interval period between the injections. A similar pattern with the dose of 150 mg Q2W was seen in the R727-CL-1003 study with a significant decrease from baseline of -67.9% versus -10.7% in the placebo group (LS mean difference versus. placebo of -57.3%; p<0.0001). Large decreases in LDL-C from baseline to 12 weeks were also observed with doses administered Q4W; however, the treatment effect was not fully maintained over a 4-week period (ie, the time interval between the two injections).

The same magnitude of effect was shown for the dose of 150 mg Q2W in the DFI11566 study, with a statistically significant decrease in LDL-C at 8 weeks in the alirocumab 150 mg + atorvastatin 80 mg group (median reduction of – 70.6 %) compared with the placebo + atorvastatin 80 mg group (median reduction of – 26.9 %).

In all three studies, consistent results were seen for total-cholesterol (TC), ApoB, non-HDL-C and ApoB/ApoA-1 ratio. A favorable trend was also observed for high-density lipoprotein cholesterol (HDL-C), apolipoprotein A-1 (ApoA-1), triglycerides (TG) and lipoprotein a (Lp (a)).

Safety results

Alirocumab was well tolerated in all completed Phase 2 studies throughout the treatment period and for all treatment groups. Injection sites reactions were reported in patients including placebo-treated patients; the reporting of these events was greatest in the R727-CL-1003 study (40.3% in alirocumab-treated patients versus 12.6% and 3.3% in DFI11565 and DFI 11566, respectively); however these events were generally transient with no dose relationship. Rare cases of hypersensitivity reactions were reported. Among all SAEs reported for all alirocumab studies, only one case, leucocytoclastic vasculitis (angiitis), was reported as being related to alirocumab (DFI11565 study). The patient developed one episode of diarrhea followed on the same evening by rash on arms, legs and abdomen, 9-days after the first administration of alirocumab 300 mg Q4W. The diagnosis was confirmed by skin biopsy. The patient was discontinued from study drug

but completed the study. The patient fully recovered from the rash after a course of tapering steroid administration. A positive antidrug antibody (ADA) status was reported with a low titer of 30 (corresponding to the minimum titer detected by the assay), only observed at the Week 20 assessment (ie, between 2.5 and 3 months after the event) with a negative retest after 6 months. Additional tests also obtained 6 months after the event were unremarkable and included normal immunoglobulin levels, a negative antinuclear antibody test, and only a mild elevation of CRP. No particular signal was noted for TEAEs related to musculoskeletal or connective tissue disorders as well as there were no LFT elevations. Given the limited published data on the safety of LDL-C level <25 mg/dL, a prespecified statistical analysis was conducted in patients reaching LDL-C value <25mg/dL in all the Phase 2 studies with no specific safety signal identified over the study duration. In the two dose finding studies (DFI11565 and R727-CL-1003), the proportion of patients reaching an LDL-C <25 mg/dL in the 150 mg Q2W group was from 31.3% to 44.8%. In DFI11566, with atorvastatin 80mg the proportion in the 150 mg Q2W group was approximately 50%.

For detailed information, please refer to the CIB (1).

Dose and regimen selection

Based on the results of the above studies, the Q2W dosing regimen was selected as the most appropriate to maintain constant LDL-C lowering throughout the interdosing interval. Since the magnitude of effect observed with 150 mg Q2W may not be needed to achieve the LDL-C goal in all patients, a lower dose of 75 mg was selected as a starting dose. This selection is also based on the LDL-C reduction needed to provide the best benefit in terms of CVD reduction, and potential safety considerations regarding low LDL-C values.

The current and most relevant evidence around the effects of achieved low LDL-C levels comes from examinations of large statin trials (12) (13) (14) as presented above, and patients with PCSK9 loss-of-function mutations (30). The patients achieving the lower levels of LDL-C had the lower CV event rates. To date, there is no evidence that very low LDL-C levels result in significant adverse health effects based on these sources of information, though this conclusion is based on a relatively low number of patients with very low LDL-C who have been studied.

Rationale for protocol design:

The objective of the present study is to evaluate the ability of alirocumab to reduce CV events in patients who recently experienced an ACS event and despite intensive statin therapy or at maximally tolerated dose don't reach the goal as defined in the guidelines for these very high risk patients.

For this randomized, double-blind, placebo-controlled study it is estimated that approximately 18,600 patients will be enrolled, of which about 18,000 will either have died or have had a minimum follow-up of approximately 24 months, supplemented by an additional subset of patients from China (~600) possibly followed for less than 24 months. Total study duration will be approximately 5 years. Randomization will occur within 52 weeks of the index ACS event and patients will enter a run-in period during which (if not already receiving such treatment before V1) they will have to be stabilized on an LMT that includes intensive statin therapy (defined as

atorvastatin 40 or 80 mg or rosuvastatin 20 or 40 mg) or maximally tolerated dose of either of these 2 agents in case of tolerability issues, and is optimized for long-term chronic use with non-statin LMT (at Investigator's discretion). Only patients not reaching goal, ie, LDL-C ≥70 mg/d L (≥1.81 mmol/L) or ApoB ≥80 mg/dL (≥ 0.8 g/L) or non HDL-C ≥100 mg/dL (≥2.59 mmol/L) under such treatment, will be randomized to either background LMT therapy + alirocumab or background LMT therapy + placebo. The choice to select eligible patients on two other lipid parameters, in addition to the traditional LDL-C target lipid parameter, is based on the acceptance that ApoB and non HDL-C can be considered to be equal to LDL-C in risk prediction (10) (11). Some adjustment in the lipid-modifying background therapy can occur during the run-in period, e.g., in case of poor tolerance to intensive statin therapy.

The proposed primary efficacy endpoint is the effect of alirocumab compared to placebo on top of best evidence background therapy on the occurrence of the following composite endpoint: coronary heart disease (CHD) death, non-fatal myocardial infarction, non-fatal and fatal ischaemic stroke, and unstable angina requiring hospitalization with stringent criteria for the definition of this later endpoint. Based on other contemporary studies (31) (32), the primary endpoint event rate in the placebo group is assumed to be 11.4% at 48 months (3.8% at 12 months, 6.4% at 24 months, 9.0% at 36 months).

In this trial, all patients will be initially treated with 75 mg Q2W, and only those patients whose LDL-C levels remain equal or higher than 50 mg/dL (1.29 mmol/L) after 1 month of treatment will be up-titrated to 150 mg Q2W (at Month 2).

With this treatment scheme, most patients can be expected to achieve their target LDL-C, as recommended by international guidelines committees (10) (11). Furthermore, the up-titration threshold set at 50 mg/dL (1.29 mmol/L), supported by findings from post-hoc analyses of the PROVE-IT, TNT and JUPITER trials, will promote the achievement of an LDL-C level within the 'physiologic ideal' zone.

Available data do not point to a lower limit of safe and effective cholesterol lowering (12) (13) (14). However, this conclusion is based on a relatively low number of patients with very low LDL-C who have been studied. In this trial, with the use of 75 mg as starting dose and a target-based up-titration scheme, it is anticipated that few patients will reach a LDL-C level below 25 mg/dL (0.65 mmol/L). For patients titrated-up to 150 mg Q2W who reach a LDL-C level below 25 mg/dL (0.65 mmol/L), a down-titration from 150 mg to 75 mg Q2W will be performed. For patients on 75 mg Q2W, two different rules will be applied depending on the level of LDL-C. In case of an LDL-C level below 25 mg/dL (0.65 mmol/L) but greater than or equal to 15 mg/dL (0.39 mmol/L), additional monitoring will be implemented to further confirm the safety of low LDL-C levels. Due to lack of available information at a LDL-C level below 15 mg/dL (0.39 mmol/L) (except in subjects with rare genetic mutations), patients reaching such low levels on 2 consecutive occasions will have their treatment discontinued.

Addition to alirocumab in patients not yet at goal should derive to further benefits in terms of reductions in LDL-C. However a greater treatment effect in patients with higher LDL-C levels at baseline cannot be ruled out. For this reason, the distribution of LDL-C levels at baseline will be monitored to ensure that the initial assumption of a mean baseline LDL-C of 90-100 mg: dL (2.33-2.59 mmol/L) is fulfilled. In case it is observed that the distribution is shifted to lower

baseline levels of LDL-C, a capping of the number of patients with a baseline LDL-C between 70-80 mg/dL may be considered.

Conclusion on the benefit risk assessment with alirocumab

Based on the clinical data available to date, treatment with alirocumab has demonstrated a significant LDL-C lowering effect and was generally well tolerated in a population of patients with non-familial hypercholesterolemia or with heterozygous familial hypercholesterolemia. The efficacy on LDL-C was associated with consistent results in total cholesterol, ApoB, non-HDL-C and ApoB/ApoA-1 ratio and a positive trend for HDL-C, TG and Lp (a). There was no evidence that alirocumab adversely affects other cardiovascular risk factors, eg, body weight, blood pressure, glucose, or CRP.

In terms of identified or potential risks with alirocumab, local injection site reactions were reported as well as rare cases of hypersensitivity reactions. Local injection site reactions were reported in both alirocumab and placebo treatment groups with no evidence of dose relationship. These AEs will be monitored in the Phase 3 program including this study. A substantial proportion of patients reached low LDL-C levels (<25 mg/dl [0.65 mmol/L]) with no safety signal identified to date. However, further monitoring for potential AEs associated with low LDL-C levels will be implemented. Although no particular signal related to CPK elevation and associated AEs (e.g., myalgia, rhabdomyolysis) was detected with the co-administration of alirocumab and statins over a maximum duration of 12 weeks, monitoring for such adverse events will continue for all the Phase 3 studies, including this study. An independent Data Monitoring Committee (DMC), dedicated to the EFC11570 study and identified as CV DMC, will meet periodically to review unblinded safety data. This CV DMC will have a close connection with the other independent DMC implemented for all Phase 3 studies evaluating the efficacy and safety of alirocumab on LDL-C (identified as Phase 3a DMC).

This CV outcome study is undertaken to demonstrate in patients who recently experienced an ACS event and who are not at their LDL-C goal despite an intensive lipid-lowering therapy that alirocumab 75mg Q2W or 75 mg Q2W / 150 mg Q2W provides an additional benefit with the reduction of CV events.

5 STUDY OBJECTIVES

5.1 PRIMARY

The primary objective of this study is to compare the effect of alirocumab with placebo on the occurrence of cardiovascular events (composite endpoint of coronary heart disease (CHD) death, non-fatal myocardial infarction (MI), fatal and non-fatal ischemic stroke, unstable angina requiring hospitalization) in patients who have experienced an acute coronary syndrome (ACS) event 4 to 52 weeks prior to randomization and are treated with an LMT regimen that is statin-intensive (defined as atorvastatin 40 or 80 mg, or rosuvastatin 20 or 40 mg) or at maximally tolerated dose of these given statins, and optimized for long-term chronic use with other non-statin LMT(s) at Investigator's discretion.

5.2 SECONDARY

The secondary objectives are:

- To compare the efficacy of alirocumab versus placebo on secondary endpoints (any CHD event, major CHD event, any CV event, composite of all-cause mortality/non-fatal MI/non-fatal ischemic stroke, all-cause mortality)

A Clinical Events Committee (CEC) will be established to adjudicate, in a blinded fashion, events corresponding to the primary and secondary cardiovascular endpoints as well as all causes of death.

- To evaluate the safety and tolerability of alirocumab throughout the study
- To evaluate the development of anti-alirocumab antibodies
- To evaluate the effect of alirocumab on low-density lipoprotein cholesterol (LDL-C) apolipoprotein B (ApoB), and non-high-density lipoprotein cholesterol (non-HDL-C)

6 STUDY DESIGN

This is a double-blind, randomized, placebo-controlled, balanced (1:1, alirocumab: placebo), parallel-group, multi-national, multicenter study. Randomization will take place between 4 weeks (28 days) and 52 weeks (+5 days) after the qualifying index ACS event and will be stratified according to country.

Prior to this randomization, eligible patients will enter a run-in period of at least 2 weeks (14 days) but no more than 16 weeks (+ 5 days), during which Investigator ensures that patient has received an LMT regimen that is:

- statin-intensive (defined as atorvastatin 40 or 80 mg or rosuvastatin 20 or 40 mg) or maximal tolerated dose of one of these 2 statins, and
- optimized for long-term chronic use (with other non-statin LMT(s) at Investigator's discretion), and
- well tolerated after at least 2 weeks of stable dose prior to V2.

Following this run-in period, only patients not reaching goal, ie, LDL-C ≥70 mg/dL (≥1.81 mmol/L) or ApoB ≥ 80 mg/dL (≥ 0.8 g/L) or non-HDL-C ≥ 100 mg/dL (≥ 2.59 mmol/L) after ≥ 2 weeks of such treatment, will be randomized to either background LMT + alirocumab, or background LMT + placebo. All patients randomized to alirocumab will initially receive alirocumab 75 mg Q2W. Patients on alirocumab not reaching the target LDL-C level (< 50 mg/dL) at Month 1 will have their dose up-titrated to 150 mg Q2W at Month 2 in a blinded fashion.

The double-blind treatment period will continue for 24 months after the closing of randomization ex-China (last date of randomization in all countries except China) or until the target number of events (1613) is reached whichever comes last.

- *Closing of randomization ex-China* will occur shortly after a total of 18,000 patients have been randomized (these may or may not include some patients randomized in China).
- *Closing of randomization in China* will occur shortly after a total of 600 patients have been randomized in China or at the common study end date (see Section 6.2.2), whichever comes first.

<u>NOTE 1</u>: *Throughout this document, 'China' refers to mainland China, excluding Hong Kong.*

<u>NOTE 2</u>: *Continued enrolment of patients in China after the initial target of 18,000 randomized globally has been reached, and leading to an increase in sample size to about 18,600 patients, will be implemented only after appropriate local authorizations in China have been obtained.*

The corresponding estimated study duration is 64 months. All patients, even if they have achieved an endpoint or those who have prematurely discontinued the study treatment, will be asked to remain in the study until the common study end date (so that all suspected efficacy endpoints are collected until the end of study).

6.1 DESCRIPTION OF THE PROTOCOL

The study will comprise 2 periods:

- A run-in period (~ 2 to 16 weeks) during which the background lipid-modifying therapy (LMT) will be adjusted as needed, to ascertain the patient is receiving the required intensive statin treatment (defined as atorvastatin 40 or 80 mg, or rosuvastatin 20 or 40 mg) or the maximally tolerated doses of these given statins, or other non-statin LMTs prior to randomization, and to stabilize this treatment.
- A double-blind treatment period (~ 2 to 5 years) during which the following should be emphasized and reinforced:
  - *compliance with study visits and assessments*: all randomized patients (including those who permanently discontinued treatment early) must remain in the study and be followed until the end of the study; sites should maintain a contact with the patient every 2 to 3 months (as per study assessments) throughout the study and contact information (for patient, family, GP/cardiologist) should be periodically updated as necessary; in addition, it is important to continuously remind patients (and their family) throughout the study that, in case of hospitalization, patient (or patient's family member) should inform site (Investigator, Coordinator) as soon as possible and not wait until the next visit.
  - *compliance with blinded study treatment (IMP):* patients should be treated with blinded study treatment for as long as possible, unless safety concerns arise (in case of temporary interruption, blinded study treatment should be restarted, as best as possible, unless there are safety concerns; to be assessed by Investigator)
  - *compliance with required background LMT regimen* (as determined from run-in period see Section 6.1.1): intensive statin therapy (atorvastatin 40 or 80 mg or rosuvastatin 20 or 40 mg) or maximal tolerated dose of one these 2 statins, optimized with addition of non-statin LMTs (at Investigator's discretion) should be continued for as long as this regimen remains well tolerated. If tolerability concerns arise, LMT can be modified, based on clinical tolerability only (see Figure 2, Section 8.8.1)
  - *compliance with requirement to not check cholesterol levels:* every effort should be made to refrain from checking cholesterol levels during the entire duration of the study (to preserve double-blind nature of the study); Investigators are encouraged to communicate and explain this aspect to patient, patient's family and patient's family doctor/cardiologist at the time of informed consent and run-in period, and throughout the study (along with rationale that patient receives best possible LMT with intensive or maximal tolerated statin therapy with addition of non-statin LMTs, at Investigator' s discretion).

Patients should be on stable diet (NCEP-ATPIII TLC diet or equivalent) throughout the entire study duration from screening to the end of the study.

6.1.1 Run-in period

6.1.1.1 Overview of run-in period

The Run-in period starts with a screening visit (V1), continues with a qualifying visit (V2), and ends with a randomization visit (V3). V1 and V2 visits can be separate visits or, in some circumstances (described below), can be combined as one visit (V1/V2)

The main goals of the run-in period are to ensure that:

- the patient has received prior to the qualifying visit (V2), a required LMT regimen that is:
  - statin-intensive (defined as atorvastatin 40 or 80 mg or rosuvastatin 20 or 40 mg) or maximal tolerated dose of one these 2 statins, and
  - optimized for long-term chronic use with addition of non-statin LMTs (at Investigator's discretion), and
  - well tolerated after at least 2 weeks of stable dose
- lipid criteria outlined in the inclusion criteria are met at V2
- patient (and his/her family) has been adequately informed and agrees to participate in a long-term study (up to ~ 5 years) with an injection every 2 weeks
- patient has been trained on at least 2 occasions to self-administer study drug injections
  - at least 2 training injections are required on 2 different occasions. These can take place at V1, V2, Combined V1/V2, or V3 at the discretion of the study site. If the second training injection is done at V3, the randomization study drug injection may be used as the second training injection
- there are no exclusion criteria

The run-in period should be planned carefully, keeping in mind 3 elements:

- The visit and time interval requirements with respect to:
  - Index ACS event
  - V1 (start of run-in period)
  - V2/V2b (collection of lipid qualifying labs)
  - V3 (randomization)
- The required background lipid-modifying therapy (LMT)
- The coronary revascularization strategy

6.1.1.2 Visit and Time Intervals during Run-In Period

6.1.1.2.1 From qualifying index ACS event to Start of Run-In Period (V1)

Interval between index ACS event and V1 is flexible. V1 can be performed:

- as early as on the day of the index ACS event
- but no later than 50 weeks after the index ACS event if V1 and V2 are separate visits (or 50 weeks + 5 days after ACS event, if combined V1/V2)

NOTE: *In case of several ACS before V1, the last ACS event fulfilling the inclusion criteria before V1 should be used as the index ACS event. In case of rescreening, see* Section 6.1.3.2.

Prior to V1, relevant information should be provided to the patient and his/her family about the study, before obtaining informed consent. Investigators are encouraged to assess and explain the following aspects to the patient and his/her family:

- Willingness to participate in a long-term study (up to ~ 5 years) in which patient will receive an injection every 2 weeks
- Willingness to come back periodically for study visits for several years (even if study treatment was discontinued early), and to remain in contact with Investigator/Coordinator during entire duration of the study, including in case of hospitalizations outside of enrolling site

It is also advisable that the Investigator communicates with the patient's general practitioner/family doctor/cardiologist about main aspects of the study requirements (e.g. background LMT during run-in, and requirement for blinding of lipid levels during the entire study).

6.1.1.2.2 Run-In Period (from V1 to V3)

The run-in period includes 2 time interval requirements (and both should be met):

- Time from V1 to V3: this is the duration of overall run-in period. It should be between 2 weeks (14 days) and 16 weeks (+5days)
- Time from index ACS event to randomization (V3): this includes the run-in period plus time between index ACS event and V1. Randomization (V3) should occur no earlier than 4 weeks (28days) and no later than 52 weeks (+ 5 days) after the index ACS event

The run-in period can be conducted in one of 2 ways, depending on whether the required LMT regimen prior to V2 lipid qualifying labs (see below Section 6.1.1.3) was already administered for at least 2 weeks (and found well tolerated) prior to V1 or not.

If required LMT regimen was not already administered for at least 2 weeks prior to V1, then V1 and V2 visits should be separate visits with an interval of at least 2 weeks:

- Investigator will administer required LMT regimen at or after V1
- V2 should occur after at least 2 weeks of treatment with the required LMT regimen (at stable doses), and no further LMT increase is planned (i.e. no addition of LMT treatment and/or no increase in doses of statin or non-statin is planned)
- V3 (randomization) can occur as soon as the qualifying lipid lab results from V2 are obtained from the central lab (typically results are available within 2-5 days of collection) and it is determined that the patient meets the lipid eligibility criteria and all other eligibility criteria have been confirmed

If it is documented that the required LMT regimen was already administered for at least 2 weeks prior to V1 (and found well tolerated), then V1 and V2 can be combined as one visit (combined V1/V2); this scenario can occur under the following circumstances:

- patient has been on stable, well tolerated dose of atorvastatin 40/80 mg or rosuvastatin 20/40 mg for at least 2 weeks prior to V1
- patient has documented intolerance to the required high doses of atorvastatin or rosuvastatin, but has been on a stable well tolerated low/moderate dose of atorvastatin or rosuvastatin for at least 2 weeks prior to V1
- patient has documented intolerance to statin therapy (i.e. intolerance to 2 or more statins) and has been on at least 2 weeks of non-statin LMT prior to V1
- patient has documented intolerance to statin therapy (i.e. intolerance to 2 or more statins), and Investigator has determined (after discussion with patient, his/her family and patient's GP/cardiologist) that best option for the long-term is to not administer any chronic LMT (statin or non-statin) to the patient

NOTE: *Prior to conducting a combined V1/V2, prior LMT treatments (and, if applicable, intolerance to statin) must be documented in a source document such as a discharge summary, history and physical, clinic note or consultation report to confirm treatment.*

In case of combined V1/V2 visit, after qualifying lipid labs are collected and if patient is eligible, V3 (randomization) should occur at least 2 weeks (14 days) after the combined V1/V2 visit.

6.1.1.2.3 Optional V2b Visit

An optional additional V2 visit with repeat central laboratory lipid assessments (V2b) may be scheduled in the following circumstance:

- V2 labs indicate that lipid inclusion criteria are not met: Investigator has the option to keep patient in the study and continue the run-in period while monitoring tolerability of statin-intensive LMT regimen. If patient subsequently develops intolerance to statin-intensive LMT leading to lowering the statin dose (or to using the other statin or, if documented intolerance to 2 or more statins, to using no statin), Investigator can repeat V2 labs after the new lower (and well tolerated) statin dose has been administered for at least

2 weeks (and ideally 4 weeks, in order for the V2b lipid labs to fully reflect the effect of the new lower dose rather than the previous higher statin dose)

Below are 2 other scenarios, one in which V2b must be performed (i.e. not optional) and one in which V2b cannot be performed:

- If V2 lipid labs inclusion criteria are met however statin dose (or non-statin LMT regimen) was subsequently increased after V2, then a V2b visit (i.e. with repeat central laboratory lipid qualifying assessments) must be performed to check if patient still qualifies with this higher dose.
- If V2 lipid labs inclusion criteria were not met with a given statin regimen, a V2b visit (ie, with repeat central laboratory lipid qualifying assessments) cannot be performed when using the same statin regimen (ie, no ‘second chance’ regarding lipid inclusion critera is allowed, using same statin regimen).

6.1.1.3 Required background LMT during the run-in period

Since the study aims to evaluate whether alirocumab is beneficial in patients who do not meet desired lipid levels with high-dose statins, one of the main objectives of the run-in period is that the Investigator has identified prior to V2 (or combined V1/V2), a background LMT regimen that meets all following criteria:

- statin-intensive (defined as atorvastatin 40 or 80 mg, or rosuvastatin 20 or 40 mg), or with maximally tolerated dose of these given statins
- optimized for long-term chronic use (i.e. with the intent of administering such treatment, on a chronic basis, for several years)
- well tolerated (after stable dose for at least 2 weeks)

Figure 1 - Overview Adjustment Required Background LMT during Run-In Period

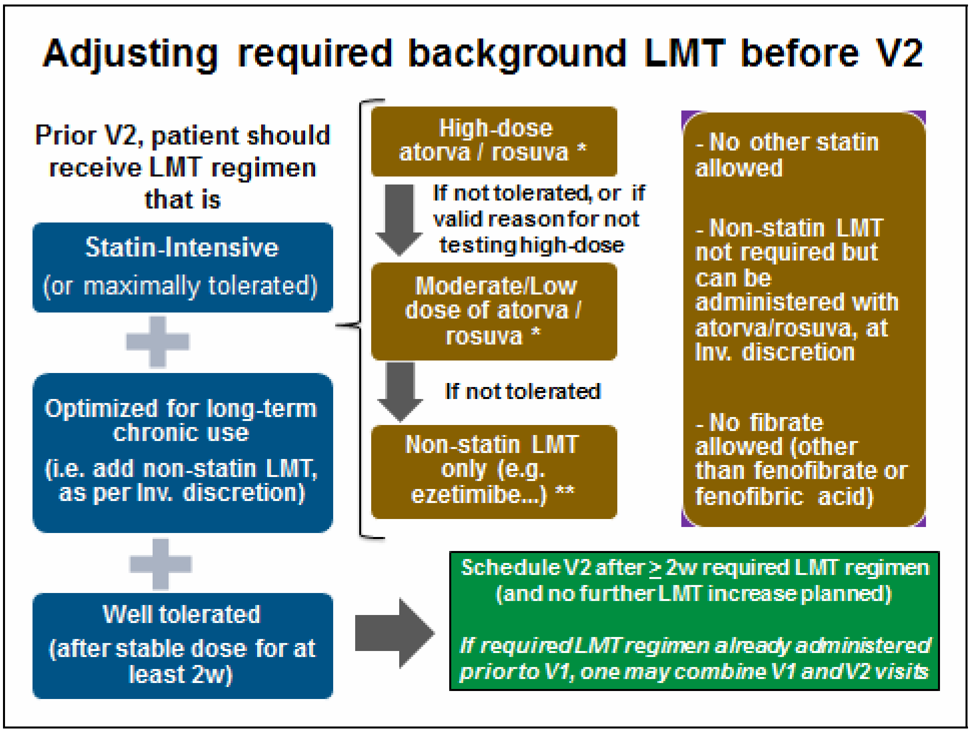

* Atorvastatin high-dose (80, 40 mg daily), moderate/low dose (20, 10 mg daily)
Rosuvastatin high-dose (40, 20 mg daily), moderate/low dose (10, 5 mg daily)

** In statin-intolerant patients (2 or more statins), after careful assessment (incl. after review of latest evidence and after discussion with the patient, his/her family and patient's primary GP/cardiologist), Investigator may determine that no background chronic LMT is appropriate for the patient.

6.1.1.3.1 Statin-intensive treatment

Statin-intensive treatment is defined as high-dose atorvastatin (40 or 80 mg daily) or high-dose rosuvastatin (20 or 40 mg daily); these should be administered to all patients, including:

- statin-naive patients (i.e. patients not receiving any LMT, or patients on non-statin LMT)
- patients previously treated with other statins (statins other than atorvastatin or rosuvastatin are not allowed during the run-in period, and should be discontinued at V1)
- patients treated with and tolerating moderate or low doses of atorvastatin or rosuvastatin

High-dose atorvastatin/rosuvastatin:

- may have been administered prior to V1, and should then be continued at V1 and beyond, or
- can be initiated at V1, or
- can be reached progressively during the interval between V1 and V2, starting with lower doses at V1 (such as in patients with known prior statin tolerability issues, or with advanced age, or low body mass index or other concerns)

If high doses of atorvastatin/rosuvastatin are administered but not tolerated, then Investigator should administer the maximal tolerated dose of atorvastatin 10 or 20 mg, or rosuvastatin 5 or 10 mg.

In infrequent instances, when there is a valid reason as per Investigator's best judgment (including but not limited to prior statin tolerability issues, advanced age, low body mass index or other concerns), it is acceptable to use during the run-in period, only a low or moderate dose of atorvastatin (10 or 20 mg) or rosuvastatin (5 or 10 mg), and not use high doses, as described above. In such instances however, the reasons for not treating the patient with high dose statin should be documented in source notes.

Statin-intolerant patients:

In statin-intolerant patients (defined as intolerance to at least 2 statins), LMT can be optimized with non-statins LMT only (e.g. ezetimibe, or other non-statin LMT). After careful assessment (and ideally after discussion with patient's primary GP/cardiologist), Investigator may alternatively elect to not to administer any LMT at all (i.e. use of non-statin LMT is not mandatory in statin-intolerant patients – see Section 6.1.1.3.2 below).

6.1.1.3.2 Optimized for long-term chronic use

Prior to V2, LMT should be also optimized for long-term chronic use, as per Investigator's discretion.

This means that, in addition to being statin-intensive, Investigator may choose to administer (before V2) non-statin LMT that he/she deems beneficial for the patient for long-term chronic administration. Addition of non-statin LMT to chronic administration of atorvastatin or rosuvastatin however is not required, and should be determined by the Investigator, and after discussion with patient's GP/cardiologist, and on the basis of available clinical evidence and clinical judgment to support such a strategy.

During the run-in period, fibrates (other than fenofibrate or fenofibric acid) are prohibited, and should be discontinued at V1, if patient was receiving such treatments.

6.1.1.3.3 Well tolerated LMT

Well tolerated LMT is defined as an LMT that is well tolerated after at least 2 weeks of stable LMT doses (statin and/or non-statin LMT) prior to V2(or V2b).

6.1.2 Coronary revascularization strategy

When indicated for the treatment of the qualifying index ACS event, a coronary revascularization (PCI, CABG) may have taken place before V1, but may also occur during the run-in period.

If a PCI and/or CABG occurs during the run-in period, randomization should be scheduled to allow for a minimum 2 week interval between the last coronary procedure and the randomization visit V3 (see exclusion criterion E18).

6.1.3 Screen failures and Rescreening

6.1.3.1 Screen failure

Screen failure may occur in (but is not limited to) the following circumstances:

- Lipid inclusion criteria are not met on the V2/V2b qualifying labs
- Presence of exclusionary non-lipid laboratory abnormalities (including on repeat labs) during run-in
- New ACS event or coronary intervention occurring within 2 weeks prior to last possible randomization day (52 weeks + 5 days) after original index ACS event, in a patient who had not yet been randomized (See exclusion criteria)
- Duration of run-in period has exceeded 16 weeks (+5 days)

6.1.3.2 Rescreening

A patient who failed screening and left the study may undergo rescreening and re-enter the study (only once) under the following scenarios:

- If lipid inclusion criteria at V2/V2b (LDL-C, non-HDL-C, Apo B) were met during a (first initial) screening with a given statin dose and patient failed screening for a reason not related to these lipid inclusion criteria, then patient can be rescreened using the same statin at same dose or, if documented intolerance, with same statin at lower dose or other authorized statin or no statin (later option only if documented intolerance to 2 or more statins). Reasons include but are not limited to: correction of exclusionary labs other than lipid inclusion criteria (ie, other than LDL-C, non-HDL-C, Apo B) , patient and/or family changed their mind regarding trial participation
- However, if lipid inclusion criteria at V2/V2b (LDL-C, non-HDL-C, Apo B) were not met during a (first) screening with a given statin dose, patient cannot be rescreened using the same statin at same dose (i.e. no 'second chance' regarding lipid inclusion critera is allowed, using same statin regimen). Such patient can only be rescreened if statin intolerance has developed subsequently (to be documented in source notes) leading to using same statin at lower dose, or using other authorized statin or no statin (later option only if documented intolerance to 2 or more statins).

In such cases, the first screening will be referred to as 'Screening 1', and the rescreening as 'Screening 2'.

For the index ACS event relative to the rescreening, one should:

- Use the original index ACS event (ie, from Screening 1) as the index ACS event for Screening 2, if the run-in period can be restarted and completed within 52 weeks (+5 days) of the original index ACS event
- Otherwise, use a new (subsequent) qualifying ACS event as the index event

6.1.4 Double-blind treatment period

The double-blind treatment period will continue until 24 months after the closing of randomization ex-China (ie, last date of randomization in all countries except China) or until the target number of events (1613) is reached, whichever comes last (see beginning of Section 6). The corresponding estimated study duration is 64 months (as described in the sample size considerations – Section 11.1).

During this double-blind treatment period, the dosing of alirocumab is intended to achieve LDL-C levels of 30-50 mg/dL (0.78-1.29 mmol/L) which is considered as the physiologic ideal level and to avoid levels of LDL-C that are clearly below the physiologic range (ie, <15 mg/dL). To achieve this goal the following up-titration or down-titration scheme (including discontinuation if needed) will be applied:

- At randomization Visit (V3), the starting dose of alirocumab will be 75 mg every 2 weeks (Q2W). At Month 2 (V5), patients randomized to alirocumab will, in a blinded manner, either:
  - Continue alirocumab 75 mg Q2W, if the Month 1 (V4) LDL-C is <50 mg/dL (1.29 mmol/L) **OR**
  - Be titrated-up to alirocumab 150 mg Q2W, if the Month 1 (V4) LDL-C is ≥50 mg/dL (1.29 mmol/L)

NOTE: *In case Month 1 (V4) LDL-C is not available or not valid (e.g. issue with blood sample) for potential up-titration at Month 2 (V5), the next available LDL-C sample at Month 2 (V5) will be used for potential up-titration at Month 4 (V6).*

- At subsequent visits, for patients on alirocumab, the following adjustments may be applied:
  - For patients receiving 150 mg Q2W:

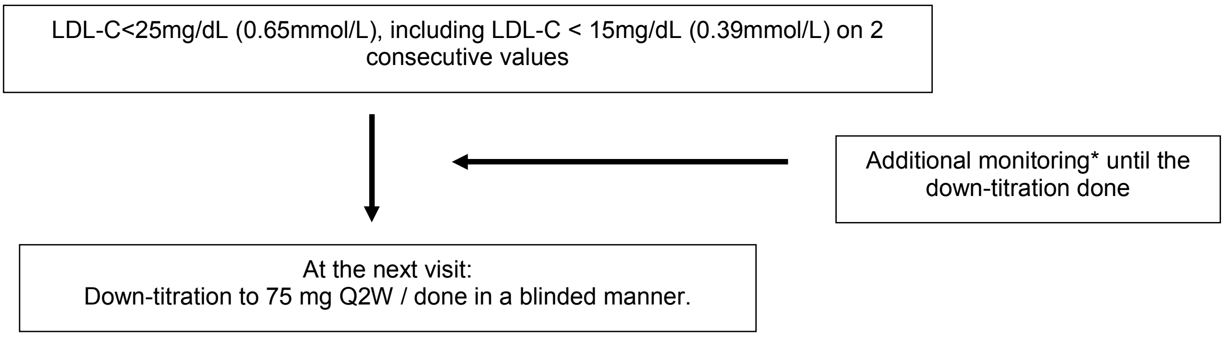

- For patients receiving 75 mg Q2W:

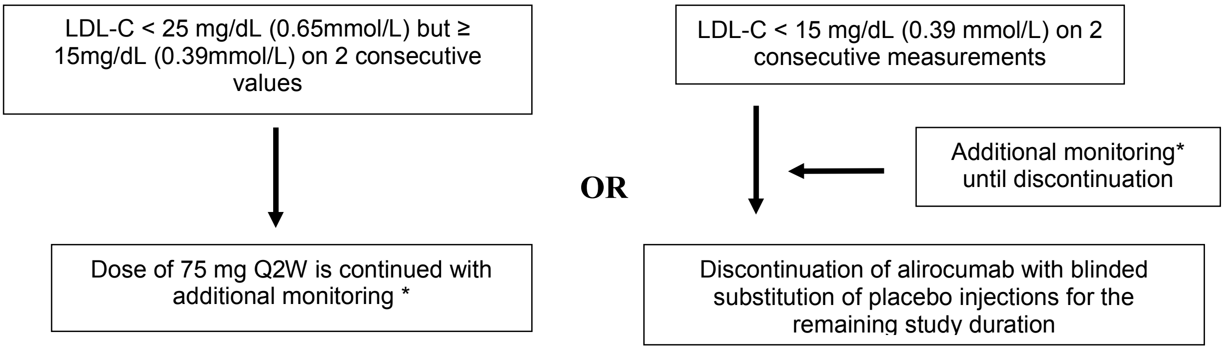

* <u>NOTE</u>: *Additional monitoring includes*:

- *Patient level listing for surveillance and review for patients who are managed by IVRS/IWRS (i.e with planned automatic down-titration from 150mg to 75mg, or switch from 75mg to placebo)*
- *Individual patient profile monitoring for potential site alerts for patients on alirocumab 75 mg and not managed by IVRS/IWRS (i.e. with no planned automatic down-titration or switch to placebo by IVRS/IWRS)*

Further details are provided in Appendix A.

As lipid values will not be communicated to investigators after randomization to maintain the blind, the continuation of the 75 mg dose, the up-titration to the 150 mg dose, the down-titration from 150 to 75 mg, or discontinuation will occur automatically, without site or patient awareness. Moreover, blinded dose adjustment will avoid the need for dummy titrations in the placebo group.

Patients who achieve 2 consecutive LDL-C levels <25 mg/dL (0.65 mmol/L) during the study will be monitored and managed as per Appendix A . An independent external physician will be notified by the central laboratory of 2 consecutive LDL-C <25 mg/dL (0.65 mmol/L). The independent physician will review the unmasked LDL-C values and patient safety data, in close collaboration with the dedicated member of the Phase 3a DMC implemented for all Phase 3 studies evaluating the efficacy and safety of alirocumab on LDL-C (called Phase 3a studies). This dedicated Phase 3a DMC member will then transmit any information as necessary to the common member involved in both DMCs (Phase 3a DMC and CV DMC).

Atorvastatin or rosuvastatin daily dose as well as dose of other non-statin LMT (if applicable) should be stable from randomization up to the common study end date, unless safety reasons prompt dose reduction or discontinuation (see also Section 8.8.1 and Section 10.3).

<u>NOTE</u>*: all randomized patients, even those who have achieved an endpoint or prematurely discontinued the study treatment should be followed during the entire duration of the study (ie, from randomization until the common study end date visit V30) for collection of suspected efficacy endpoints (please refer to Section 10.3 for further details on handling of patients who discontinued treatment early).*

6.2 DURATION OF STUDY PARTICIPATION.

6.2.1 Duration of study participation for each patient

The duration of the run-in period (V1 to V3) must be between 2 weeks (14 days) and 16 weeks (+5 days). Randomization must occur no earlier than 4 weeks (28 days) and no later than 52 weeks (+ 5 days) after the index ACS event.

The double-blind treatment period will continue (for about 2 to 5 years) until 24 months after the closing of randomization ex-China (ie, after last date of randomization in all countries except China) or until the target number of events (1613) is reached, whichever comes last (see beginning of Section 6).

The corresponding estimated study duration is 64 months (as described in the sample size considerations).

6.2.2 Determination of end of clinical trial (all patients) - Common Study End Date

All randomized patients will be followed up until the Common Study End Date (CSED) visit, which is the final study visit (and a clinic visit) to be scheduled for each patient within 30 days of the CSED (see Section 10.1.5.4).

The CSED is defined as the date corresponding to 24 months after the closing of randomization ex-China (last date of randomization in all countries except China) or date when the target number of events (1613) is reached, whichever comes last.

- *Closing of randomization ex-China* will occur shortly after a total of 18,000 patients have been randomized (these may or may not include some patients randomized in China).

Closing of randomization in China will occur shortly after a total of 600 patients have been randomized in China or at the common study end date (see Section 6.2.2), whichever comes first.

Therefore, at the end of the double-blind treatment period, the overall randomized population will include about 18,000 patients who have either died or been followed for a minimum of 24 months, supplemented with an additional subset of patients from China (~600) who may be followed for less than 24 months.

NOTE: Common study end date is not dependent on the situation in China, and therefore will apply, regardless of Clinical Trial Authorization status and randomization status in China.

6.3 INTERIM ANALYSIS

Patients will be followed until 1,613 patients experience at least one primary endpoint event or for approximately 24 months after the last date of randomization ex-China, whichever comes last.

Interim analyses for futility will be performed, under the supervision of the CV DMC, when 50% and 75% of events have occurred.

An interim analysis for efficacy will be performed when 75% of events have occurred. Control of the type I and type II error will be ensured using gamma (-5) spending function for Type II error (futility) and Gamma (-22) for Type I error (efficacy). Stopping rules details are further described in Section 11.5.

6.4 STUDY COMMITTEES

Executive Steering Committee:

The Executive Steering Committee is composed of university-based scientists (experts in cardiology field, and lipids) with clinical and methodological expertise, working in collaboration with Sponsor based scientists. The Steering Committee provides scientific and strategic direction for the trial and will have overall responsibility for its execution. The committee provides guidance on producing and conducting a scientifically sound design and ensuring accurate reporting of the study. In that capacity, the Steering Committee must address and resolve scientific issues encountered during the study.

Among its responsibilities, the Steering Committee will receive blinded (aggregate) study status reports from the Sponsor. The Steering Committee will also review the recommendations from the Data Monitoring Committee throughout the study. The Steering Committee members and Sponsor based scientists will participate in face-to-face meetings at regular intervals throughout the study and in regularly scheduled teleconferences.

Detailed activities and responsibilities of the Steering Committee are described in the Steering Committee charter.

Data Monitoring Committee:

An independent Data Monitoring Committee (DMC), composed of members independent from the Sponsor and the study Investigators, is implemented in order to monitor patient safety by conducting formal reviews of accumulated safety data that will be unblinded. This DMC exclusively dedicated to this study will be identified as CV DMC. The chairman of the DMC dedicated to the Phase 3a studies supporting the LDL-C reduction indication (Phase 3a DMC) will be also a member of this CV DMC and will serve as a liaison between the two DMCs. Safety data review will include the cardiovascular outcomes adjudicated or not at the time of this review. The CV DMC will also supervise the two interim analyses for futility and efficacy conducted when 50% and 75% of events occur (see Section 6.3). The CV DMC will provide the Sponsor and the Steering Committee with appropriate recommendations on the conduct of the clinical trial to ensure the protection and safety of the patients enrolled in the study. In addition, the CV DMC will also institute any measures that may be required for ensuring the integrity of the study results during the study execution.

Additionally, the CV DMC will thoroughly analyze the aggregate data for patients who achieve LDL-C <25 mg/dL during their periodic reviews throughout the study and more particularly, will review adverse events potentially associated with LDL-C <25 mg/dL (0.65 mmol/L) (see Section 10.6.3 and Appendix A).

All activities and responsibilities of this CV DMC are described in the DMC charter.

Clinical Events Committee:

The Clinical Events Committee (CEC), managed by the Duke Clinical Research Institute (DCRI), is composed of experts in the field of cardiovascular diseases, independent from the Sponsor and the Investigators. This committee will be responsible for defining, validating and classifying, in a blinded fashion, all components of the primary and secondary endpoints related to cardiovascular outcomes as well as validating the classification of the cause of all deaths.

A CEC charter and an adjudication operational manual specify the procedures, criteria, and classification used for adjudication of these events.

7 SELECTION OF PATIENTS

<u>NOTE</u>: *Qualifying visit (V2) as mentioned below in some inclusion / exclusion criteria refers to the latest visit where lipid labs are collected (before V3), and may correspond to a combined V1/V2, V2 or V2b visit.*

7.1 INCLUSION CRITERIA

I 01. (A6) Patients hospitalized for ACS (ST-elevation MI, non-ST-elevation MI or high-risk unstable angina) defined by:

- Ischemic symptoms with unstable pattern, occurring at rest or minimal exertion within 72 hrs of an unscheduled hospital admission, due to presumed or proven obstructive coronary disease **<u>AND</u> at least one of the following (A and/or B):**

A) Elevated cardiac biomarkers (troponin I or T or CK-MB with at least one determination >99th percentile or upper limit of normal for the laboratory).

<u>OR</u>

B) Resting ECG changes consistent with ischemia or infarction (B1) **AND** additional evidence of obstructive coronary disease, based upon the following criteria (B2):

B1. Resting ECG changes consistent with ischemia or infarction requires at least one of the following:

a) new or presumed new ST depression

b) new or presumed new ST elevation

c) new or presumed new T wave inversion

B2. Additional evidence of obstructive coronary disease requires at least one of the following:

a) new or presumed new evidence of myocardial ischemia or infarction by perfusion imaging

b) new or presumed new regional wall motion abnormality

c) current evidence of at least one epicardial coronary artery stenosis $\geq$ 70% by coronary angiography

a) need for revascularization (PCI or CABG) related to index ACS event

<u>NOTE</u>*: Latest ACS event occurring prior to V1 and meeting the ACS criteria as defined above will be the qualifying index ACS event. In case of rescreening, see* Section 6.1.3.2

I 02. (A6) Patient lipid levels not adequately controlled at V2 (qualifying visit) despite evidence-based lipid lowering therapy (including intensive atorvastatin/rosuvastatin therapy or maximally tolerated dose of either of these 2 statins) or other non-statin LMTs. Inadequate lipid control means that patient must meet at least one of the following criteria at V2 to qualify:

- LDL-C ≥ 70 mg/dL [≥1.81 mmol/L], or
- ApoB ≥ 80 mg/dL [≥ 0.8 g/L], or
- non-HDL-C ≥100 mg/dL [≥2.59 mmol/L]

I 03. Signed written informed consent.

7.2 EXCLUSION CRITERIA

Patients who have met all the above inclusion criteria listed in Section 7.1 will be screened for the following exclusion criteria which are sorted and numbered in the following 3 subsections:

7.2.1 Exclusion criteria related to study methodology

E 01. Age < 40 years.

E 02. (A6) Uncontrolled hypertension (multiple readings with SBP > 180 mmHg or DBP > 110 mmHg) at V3.

E 03. History of New York Heart Association (NYHA) class III or IV congestive heart failure persisting despite treatment or, if measured, LVEF < 25% at the most recent measurement.

E 04. Known history of hemorrhagic stroke.

E 05. History of cancer within the past 5 years, except for adequately treated basal cell skin cancer, squamous cell skin cancer, or in situ cervical cancer.

E 06. (A6) Recent diagnosis of hypothyroidism for which treatment was initiated within 1 month prior to qualifying visit (V2).

E 07. Patient who has been previously treated with at least one dose of alirocumab or any other anti-PCSK9 monoclonal antibody in other clinical trials.

E 08. Patient who has taken other investigational drugs within 1 month or 5 half-lives, whichever is longer.

E 09. (A6) Laboratory findings measured during screening and before randomization visit:

- Positive test for hepatitis B surface antigen
- Positive hepatitis C antibody confirmed with positive RNA testing (indicative of active hepatitis C infection)

- Triglycerides (TG) > 400mg/dL (>4.52 mmol/L) (1 repeat lab allowed)
- Positive serum or urine pregnancy test in females of childbearing potential
- eGFR <30 mL/min/1.73 m² according to 4-variable MDRD Study equation (calculated by central lab)
- ALT or AST >3 x ULN on most recent determination prior to randomization (1 repeat lab is allowed)
- CPK >3 x ULN on most recent determination prior to randomization (1 repeat lab is allowed)

E 10. Conditions/situations such as:

- Any clinically significant abnormality identified at the time of screening that in the judgment of the Investigator or any sub-Investigator would preclude safe completion of the study or constrain endpoints assessment such as major systemic diseases, patients with short life expectancy.
- Patients considered by the Investigator or any sub-Investigator as inappropriate for this study for any reason, eg:
  - Those deemed unable to meet specific protocol requirements, such as scheduled visits
  - Investigator or any sub-Investigator, pharmacist, study coordinator, other study staff or relative thereof directly involved in the conduct of the protocol, etc
  - Presence of any other conditions (eg, geographic, social….) actual or anticipated, that the Investigator feels would restrict or limit the patient's participation for the duration of the study

For the entry into the double-blind treatment period (randomization):

E 11. (A6) All of the 3 following criteria are concomitantly present at the qualifying visit (V2):

- LDL-C <70 mg/dL (<1.81 mmol/L), <u>and</u>
- ApoB <80 mg/dL (<0.8 g/L), <u>and</u>
- non-HDL-C <100 mg/dL (<2.59 mmol/L)

<u>NOTE</u>: *If not all 3 but only 1 or 2 criteria are present then the patient may qualify*

E 12. (A6) Patients in whom the qualifying index ACS event occurred less than 4 weeks (28 days) or more than 52 weeks (+ 5 days) prior to randomization visit (V3).

E 13. (A6) Not on stable LMT doses (statin and/or non-statin LMT) for at least 2 weeks prior to qualifying visit (V2).

E 14. (A6) Use of fibrates, other than fenofibrate or fenofibric acid, during the run-in period.

E 15. (A6) In patients who meet lipid eligibility criteria at qualifying visit (V2), any increase in dose of atorvastatin, rosuvastatin or non-statin LMT after V2 without subsequent requalification for lipid laboratory parameters.

E 16. (A6) Use of red yeast rice products during the run-in period up to randomization Visit (V3).

E 17. (A6) New ACS within 2 weeks prior to the randomization Visit (V3).

E 18. (A6) Coronary revascularization (PCI or CABG) planned after randomization and/or performed within 2 weeks prior to the randomization Visit (V3).

E 19. Patient who withdraws consent during the screening period (patient who is not willing to continue or fails to return).

7.2.2 Exclusion criteria related to the background therapy

E 20. All contraindications to atorvastatin, rosuvastatin or other LMTs or warning/precaution of use (when appropriate) as displayed in the respective National Product Labeling for these treatments.

7.2.3 Exclusion criteria related to the current knowledge of alirocumab

E 21. (A6) Known hypersensitivity to monoclonal antibody or any component of the drug product.

E 22. Pregnant or breast-feeding women.

E 23. Women of childbearing potential not protected by highly effective method(s) of birth control (as defined in the informed consent form and/or in a local protocol addendum) and/or who are unwilling or unable to be tested for pregnancy.

NOTE: Women of childbearing potential must have a confirmed negative pregnancy test at screening and randomization visits. They must use a highly effective contraceptive method throughout the entire duration of the study while receiving blinded study treatment (IMP), and for 10 weeks following the last injection of blinded study treatment (IMP) and agree to repeat urine pregnancy test at designated visits. The applied methods of contraception have to meet the criteria for a highly effective method of birth control according to the ‘Note for guidance on non-clinical safety studies for the conduct of human clinical trials for pharmaceuticals (CPMP/ICH/286/95)’ (33). Postmenopausal women must be amenorrheic for at least 12 months.

8 STUDY TREATMENTS

8.1 INVESTIGATIONAL MEDICINAL PRODUCT(S)

Sterile alirocumab drug product will be supplied at a concentration of 75 mg/mL and 150 mg/mL both as 1 mL volume in an autoinjector.

Sterile placebo for alirocumab will be prepared in the same formulation as alirocumab without the addition of protein as 1 mL volume in an autoinjector.

NOTE: in order to ensure the continuity of the study treatment without interruption (only in the event the manufacturer faces any performance or supply issues of the auto-injector), contingency alternatives are:

- in case of disruption of the 150 mg auto-injector only, if the use of 75 mg auto-injectors is maintained, patients will need to administer 2 injections as follows:
  - 2 injections of 75 mg as 1 mL each in an auto-injector for patients receiving the 150 mg dose
  - 1 injection of 75 mg as 1 mL in an auto-injector plus 1 injection of placebo as 1mL in an auto-injector for patients receiving the 75mg dose
  - 2 placebo injections as 1 mL each in an auto-injector for patients receiving placebo

OR

- in case of disruption of either 75 mg or 150 mg or both auto-injectors, patients will be switched to the use of prefilled syringes of placebo, 75 mg and 150 mg, with one injection of 1 mL for each of these doses

Should this occur, the alternative investigational medicinal product (IMP) will be maintained until the end of the study.

8.1.1 Route and method of administration

A manual for IMP administration (injection instruction manual) will be provided to patients containing detailed instructions on use. Also, an administration package containing gauze, alcohol swabs, band aids, etc will be provided to the patients.

The IMP could be administered by self-injection or by another designated person (such as a spouse, relative, nurse etc…). The used autoinjector will be discarded in a sharps container which will be provided to patients. It is recommended that the IMP injections be rotated within an anatomical area (eg, right thigh then left thigh or right abdomen then left abdomen). Patients also have the option to inject in a different anatomical area (eg, thigh then abdomen) during the study. It should be noted that if patients have problems activating the auto-injector by pressing the

needle cover against their belly (e.g. soft tissue), it is recommended to inject into the thigh, where the skin is firmer than the belly. If another concomitant drug is being injected at the same site planned for the IMP injection, then the patient should be advised to use an alternate location for administration of the IMP.

Patients will be asked to store the IMP in a refrigerator. Prior to administration, the IMP should be set outside in a safe location at room temperature for about 30 to 40 minutes. Thereafter, the IMP should be administered as soon as possible.

Instructions as outlined above should be provided to the patient (or to another designated person [such as spouse, relative, nurse etc...] who will administer the injections) during the run-in period (training injections) and as needed during the course of the study. Close supervision and feedback should be given at the training visit, randomization visit, and other visits as needed.

8.1.2 Timing of administration

During the run-in period, at least 2 training injections are required on 2 different occasions. These can take place at V1, V2, Combined V1/V2, or V3 at the discretion of the study site. First training injection (with placebo) must be done before V3. The initial IMP double-blind injection (active or placebo) performed at V3 may serve as second training injection. If needed, additional training injections (with placebo) are available.

During the double-blind treatment period, alirocumab or placebo will be administered subcutaneously every 2 weeks, starting at randomization Visit (V3, Day 1, Month 0) continuing up to the common study end date.

Further training with the scheduled double-blind IMP can be done at any time during the study as necessary.

Double-blind IMP will start as soon as possible after the call for randomization using the treatment kit number provided by the IVRS. The first injection after randomization will be done at the investigational site by the patient or another designated person (such as spouse, relative, etc…) under direct site staff supervision. Patients will be monitored at the investigational site for at least 30 minutes after this first double-blind injection.

IMP should ideally be administered every 2 weeks subcutaneously at approximately the same time of the day; however it is acceptable to have a window period of ± 3 days. The time of the day is based on patient's preference.

If by mistake or due to other circumstances an injection is delayed by more than 7 days or completely missed, then the patient should return to the original schedule of study treatment administration without administering delayed injections. On the other hand, if the delay is less than or equal to 7 days from the missed date, then the patient should administer the delayed injection and then resume the original schedule of study treatment administration.

8.2 NON-INVESTIGATIONAL MEDICINAL PRODUCT(S)

The following classes of drugs are identified as non-investigational medicinal products (NIMP) because the medication is a background therapy:

- Statins (during run-in period: only atorvastatin or rosuvastatin are allowed; after randomization: every effort should be made to continue atorvastatin / rosuvastatin until end of study; alternative, including other statin, allowed in case intolerance to lowest dose of atorvastatin/rosuvastatin has developed post-randomization – see Figure 2, Section 8.8.2)
- Cholesterol absorption inhibitors (ezetimibe)
- Bile acid-binding sequestrants (such as cholestyramine, colestipol, colesevelam)
- Nicotinic acid (niacin)
- Fenofibrate, fenofibric acid
- Omega-3 fatty acids

Please see Section 8.8 for further information.

8.3 BLINDING PROCEDURES

8.3.1 Methods of blinding

Alirocumab and placebo will be provided in identically matched auto injector and packaged identically which includes labeling to protect the blind.

Each double-blind treatment kit will be labeled with a number, which will be generated by a computer program from Sanofi. The treatment kit numbers will be obtained by the Investigator at the time of patient randomization and subsequent patient visits scheduled via a centralized treatment allocation system that will be available 24 hours-a-day, 7 days-a-week.

In accordance with the double-blind design, study patients, Investigators and study site personnel will remain blinded to study treatment and will not have access to the randomization (treatment codes) except under circumstances described in Section 8.3.6.

8.3.2 Lipid parameters

Lipid parameter values from blood samples obtained after the randomization visit, run by the central lab, will not be communicated to the sites so that they cannot deduce the treatment group of their patients based on LDL-C level attained. The sponsor's operational team will not have access to lipid parameters after randomization and until after the final database lock has occurred.

(electronic 1.0)

For patients who achieve 2 consecutive LDL-C <25 mg/dL (0.65mmol/L) on alirocumab and depending on the dose received, the following will be applied:

- If the dose is 150 mg Q2W:

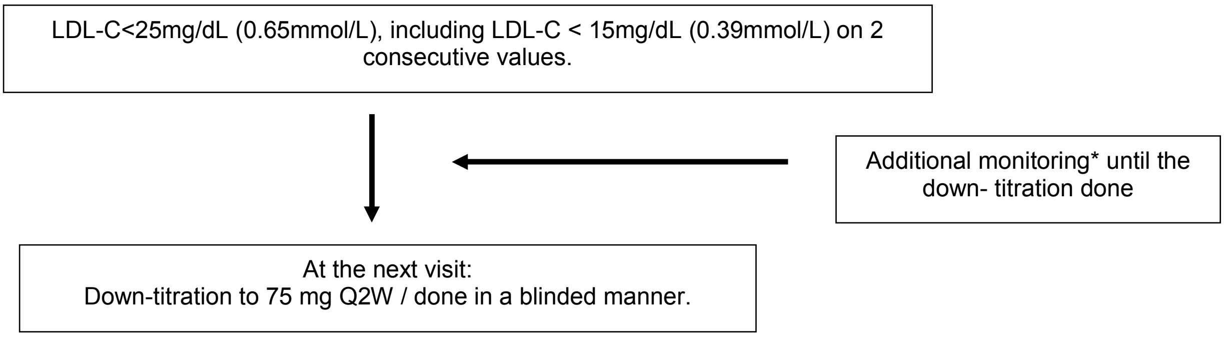

- If the dose is 75 mg Q2W:

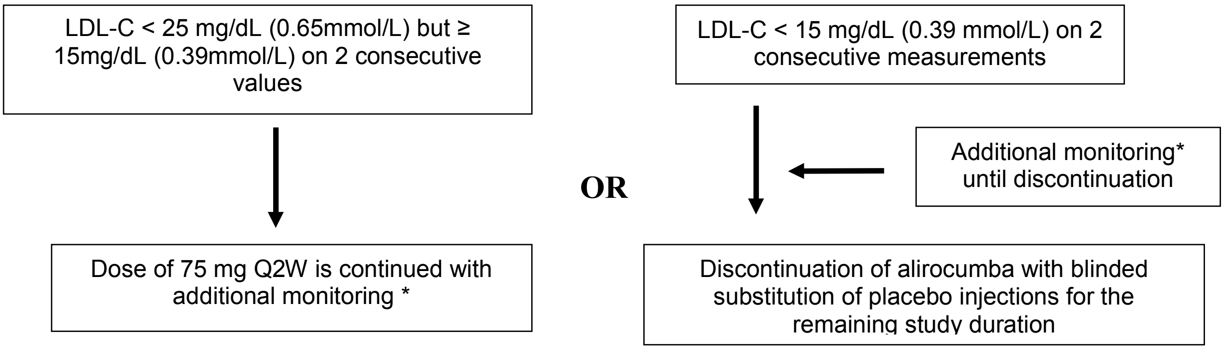

* <u>NOTE</u>: *Additional monitoring includes:*

- *Patient level listing for surveillance and review for patients who are managed by IVRS/IWRS (i.e with planned automatic down-titration from 150 mg to 75 mg, or switch from 75mg to placebo)*
- *Individual patient profile monitoring for potential site alerts for patients on alirocumab 75 mg and not managed by IVRS/IWRS (i.e. with no planned automatic down-titration or switch to placebo by IVRS/IWRS)*

Further details are provided in Appendix A.

As described above, in case of LDL-C <25 mg/dL (0.65 mmol/L) on 2 consecutive values and until specific actions are undertaken, patients will be monitored according to process outlined in Section 10.6.3 and Appendix A. In order to maintain the integrity of the blind as much as possible with this monitoring process, the following points will be undertaken:

- Specific steps will be in place to ensure that the work which will be carried out by the central lab group and the communication with the independent external physician(s) (also known as independent physician), who is responsible for closely monitoring patients with these 2 consecutive LDL-C levels, will be in strict confidence.

- The independent physician(s) and the dedicated member of the Phase 3a DMC (implemented for all Phase 3 studies evaluating the efficacy and safety of alirocumab on LDL-C) will work in close collaboration and independently from the clinical team and the sites. This dedicated Phase 3a DMC member will then transmit any information as necessary to the common member involved in both DMCs (CV DMC and Phase 3a DMC)
- The actual LDL-C levels will not be reported to the sites
- Monitoring will be discontinued when specific actions are undertaken as described above, unless patients still display a LDL-C <25 mg/dL

8.3.3 Anti-alirocumab antibodies

Patients' anti-alirocumab antibody results will not be communicated to the sites during the study.

The sponsor's operational team will not have access to anti- alirocumab antibodies associated with patient identification until after the final database lock has occurred.

The lab technicians involved in the determination of patients' anti- alirocumab antibodies are excluded from the operational team and a process will be set up to prevent any potential unblinding.

Patients who have titers at or above 240 for anti- alirocumab antibodies at the common study end date will have an additional antibody sample between 6 to 12 months after this date.

In patients who permanently discontinued treatment early, blood sample for anti- alirocumab antibodies should be drawn at the early end-of-treatment visit (V70) and when the patient returns for an on-site clinic visit where this assessment was planned, i.e. annually (see also Section 10.3.4).

8.3.4 Committees

The independent Clinical Events Committee (CEC) will review and adjudicate events in a blinded manner.

The Data Monitoring Committee will receive blinded by treatment group or unblinded (if necessary) confidential reports from an independent statistician for review (Section 6.4).

8.3.5 Data Analysis

Regular DMC safety analyses and both Interim Analyses will be performed by the external independent CV DMC Statistician and will be reviewed under the supervision of the CV DMC.

8.3.6 Randomization code breaking during the study

In case of an Adverse Event (AE), the code must be broken by the site only in exceptional circumstances when knowledge of the IMP is essential for treating the patient. If possible, a contact should be initiated with the Monitoring Team/Medical Monitor before breaking the code. All calls will be documented by the Monitoring Team as appropriate to include date and time of the call, name of the person contacted within the Monitoring Team, patient ID, documentation of the request, and decision for unblinding or not.

Code breaking can be performed at any time by using the proper module of the centralized treatment allocation system and/or by calling any other phone number provided by the Sponsor for that purpose. However, it is preferable to contact the Medical Monitor to discuss the case before unblinding the case. If the blind is broken, the Investigator should document the date, time of day, and reason for code breaking, and report this information (or “relevant information as required by”) on the appropriate page of the electronic case report from (eCRF).

Note that when documenting the reason for unblinding, the Investigator must not provide any detail regarding the nature of the IMP. The Investigator should not divulge IMP detail to the Sponsor’s representative or to any staff members until database closure. Furthermore, when completing forms (eg, AE, SAE), the study treatment should not be disclosed on the forms.

The code-breaking can also be performed by contacting the "24 hour alert system"; but this system should be used in very exceptional cases only (ie, unavailability of a centralized treatment allocation system or inability to contact Investigator and/or site staff). However, the preferred option is to unblind using a centralized treatment allocation system. The Investigators will be informed by the clinical monitoring team about the availability of the local code-breaking details (through an emergency centralized 24 hour telephone system for use with e-SMS). A patient card, including the relevant “24 hour alert system” telephone number will be provided to every patient who will participate in the study.

Unblinding may also be performed by the Sponsor for some Serious Adverse Events that are both related and unexpected in order to conform to regulatory reporting requirements.

8.4 METHOD OF ASSIGNING PATIENTS TO TREATMENT GROUP

The randomized list of treatment kit numbers will be generated centrally by Sanofi. The IMP [alirocumab kit (75 mg or 150 mg), or placebo kit] will be packaged in accordance with this list.

The Project Demand manager will provide the randomized list of treatment kit numbers and the Study Biostatistician will provide the randomization scheme to the centralized treatment allocation system provider. Then, this centralized treatment allocation system provider will generate the patient randomization list according to which it will allocate the treatment kits to the patients.

Patients will be randomized to receive either placebo or alirocumab during the double-blind study treatment period using a ratio 1:1, with permuted-block randomization. Randomization will be stratified according to country.

The treatment kit numbers will be allocated using the centralized treatment allocation system on randomization visit (Day 1, Month 0), Month 2, Month 4, every 4 months up to Month 24, and then every 6 months up to Month 64.

For patients in the alirocumab treatment arm, the treatment kit allocated at Month 2 (V5) will be based on their Month 1 (V4) LDL-C level following the up-titration rules (see Section 6.1.4). Regular transfer of data will be planned between the central laboratory and the centralized treatment allocation system provider in order to proceed in a blinded manner for study sites and sponsor.

Before randomizing a patient, the Investigator or designee will have to contact the centralized treatment allocation system.

A randomized patient is defined as a patient who is registered and assigned with a treatment kit number from the centralized treatment allocation system, as documented from its log file. A patient cannot be randomized more than once in the study. If a treatment is used without contacting the centralized treatment allocation system “patient will be considered as not randomized and withdrawn from the study.

Two types of centralized treatment allocation system will be used, the Interactive Voice Response System (IVRS) and the Interactive Web Response System (IWRS) depending on the choice of the site.

8.5 PACKAGING AND LABELING

For the double-blind treatment period, each double-blind treatment kit, either alirocumab or placebo, will be prepared to contain 6 autoinjectors in a child-resistant package.

In order to protect the blind, all double-blind treatment kit boxes will have the same look and feel and therefore will be labeled with a double-blind label.

In addition to the double-blind treatment kits, a training kit containing 1 placebo autoinjector each will be prepared for the purpose of instructing patients on injection administration which is to be performed prior to randomization. These training injections should be performed on site at screening Visit (V1, Week-16 to Week-4) and qualifying Visit (V2, Week-2), respectively. A third training injection can be performed at an additional visit such as the optional visit V2b.

The content of the labeling is in accordance with the local regulatory specifications and requirements.

8.6 STORAGE CONDITIONS AND SHELF LIFE

The IMP (alirocumab or placebo) will be stored in a refrigerator between +2°C and +8°C (36°- 46° F) by the site. The temperature of the site refrigerator should be checked every working day and recorded on a log sheet.

The IMP that will be stored at the investigational site should be kept in an appropriate locked room, under the responsibility of the Investigator or designee or other authorized person in accordance with the storage conditions indicated on the label.

After the supply of IMP kits to patients at the study site visits, appropriate provisions as necessary will be in place for transportation of the IMP kits from the study site to the patient's refrigerator.

NOTE: *Exceptionally, after discussion between site and sponsor (eg, patient unable to attend a clinic visit due to special circumstances) some IMP kits could be supplied, when feasible, directly from site to patient via a sponsor-approved courier company. This process (which requires maintenance of the cold chain) would be implemented only at selected sites/countries (where certain conditions would be fulfilled, and where permitted locally) and for selected patients (who could handle and would consent to such a process). This direct-to-patient process will be described in detail in a separate document and would be implemented after appropriate training of monitoring teams and investigational sites.*

8.7 RESPONSIBILITIES

The Investigator, the Pharmacist, or other personnel allowed to store and dispense IMP will be responsible for ensuring that the IMP used in the clinical trial is securely maintained as specified by the Sponsor and in accordance with the applicable regulatory requirements.

All IMP shall be dispensed after IVRS contact in accordance with the Clinical Trial Protocol and it is the Investigator's responsibility to ensure that an accurate record of IMP issued and returned is maintained.

Any quality issue noticed with the receipt or use of an IMP (deficiency in condition, appearance, pertaining documentation, labeling, expiration date, etc.) should be promptly notified to the Sponsor. Some deficiencies may be recorded through a complaint procedure (ie, Product Technical Complaint [PTC] form).

A potential defect in the quality of IMP may be subject to initiation of a recall procedure by the Sponsor. In this case, the Investigator will be responsible for promptly addressing any request made by the Sponsor, in order to recall IMP and eliminate potential hazards.

Under no circumstances will the Investigator supply IMP to a third party, allows the IMP to be used other than as directed by this Clinical Trial Protocol, or dispose of IMP in any other manner.

(electronic 1.0)

8.7.1 Treatment accountability and compliance

IMP administration data will be recorded by the patients onto a patient's diary.

Measures taken to ensure and document IMP compliance and accountability are described below:

- The Investigator or designee will obtain via IVRS/IWRS the treatment kit number(s) and he/she will dispense the treatment kit(s) to the patient.
- The accountability is to be performed at IMP kit re-supply visits only (see Section 10.1.5). The used and unused kit(s) should be brought back to such visits for accountability purposes.
- The Investigator or designee will complete the corresponding treatment log form from patient's diary.

  NOTES:

  At every opportunity (clinic visit or phone call), Investigator should remind patient to complete the diary and to bring the diary at the next clinic visit.

  If patient forgets to bring the diary at a clinic visit, site should come up with an alternative solution to obtain as soon as possible information contained in the diary in order to be able to complete IMP information in eCRF in a timely manner.

  If patient dies, site should make every possible effort to obtain patient diary from the family (in addition to collecting detailed information about the circumstances of patient death).
- The Investigator/study coordinator will enter data in the appropriate eCRF pages, according to data recorded in the treatment log form.
- The monitor will check the data consistency between eCRF pages, treatment log forms using patient's diary, and returned unused IMPs of a corresponding kit.

8.7.2 Return and/or destruction of treatments

Destruction of IMP kits (i.e., used, unused or expired) can be performed during the course of the study in addition to the end of the study.

Destruction at site is strongly encouraged and can be performed provided that the following requirements are met:

- Site has the appropriate facilities to destroy IMP and
- Site has procedures to allow traceability of the batches and quantities destroyed and delivers the corresponding destruction documentation/certificate,
- Sanofi provides the appropriate authorization.

In case the above requirements are not satisfied and the site cannot safely destroy IMP, the treatments will be returned to the local depot for destruction. A detailed treatment log of the returned IMP will be established with the Investigator or designee and countersigned by the Investigator and the Monitoring Team.

8.8 CONCOMITANT MEDICATION

A concomitant medication is any treatment received by the patient concomitantly to the study (until the common study end date).

8.8.1 Background Lipid-Modifying Therapy

During the run-in period, all LMTs are authorized with the exception of fibrates (other than fenofibrate and fenofibric acid), and statins other than atorvastatin, and rosuvastatin.

These include but are not limited to:

- Statins (atorvastatin, rosuvastatin only; other statins are not allowed during run-in period)
- Cholesterol absorption inhibitors (ezetimibe)
- Bile acid-binding sequestrants (such as cholestyramine, colestipol, colesevelam)
- Nicotinic acid (niacin)
- Fenofibrate, fenofibric acid
- Omega-3 fatty acids

After randomization, the required background LMT regimen, as determined from the run-in period, with intensive statin therapy (atorvastatin 40 or 80 mg or rosuvastatin 20 or 40 mg) or maximal tolerated dose of one these 2 statins, and optimized with addition of non-statin LMTs (at the Investigator's discretion) should be continued, for as long as this regimen remains well tolerated.

If, during the course of the study, significant tolerability concerns arise and patient is found intolerant to any dose of atorvastatin or rosuvastatin, other statins (i.e. other than atorvastatin or rosuvastatin) as well as non-statin LMTs are authorized. A proposed algorithm for modification of LMT post-randomization is provided in Figure 2.

Figure 2 - Proposed algorithm for adjusting LMT post-randomization, in case tolerability concerns arise (e.g. myalgia)

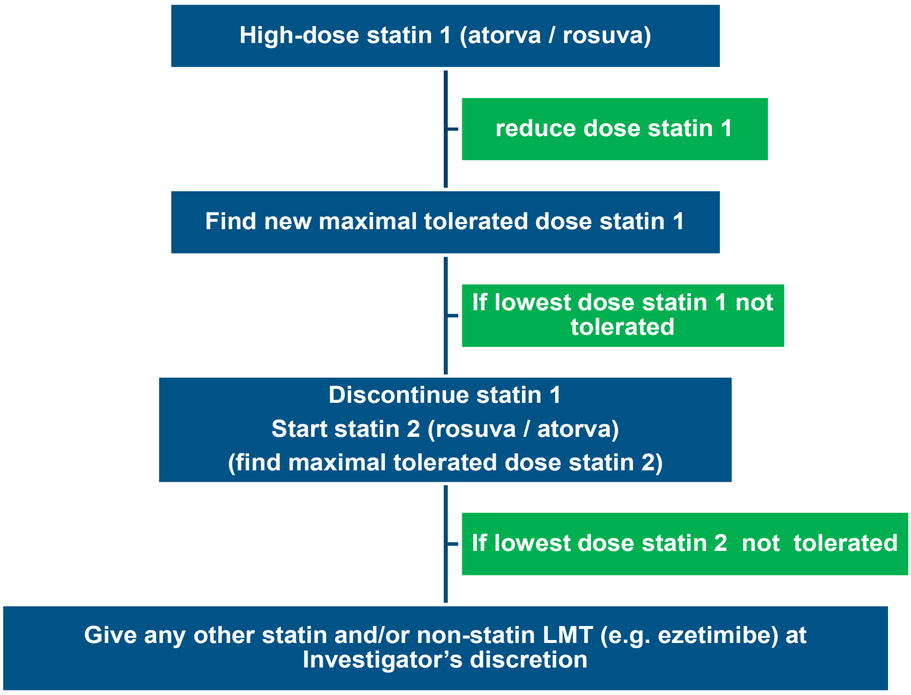

NOTE: *If statin 1 is atorvastatin, statin 2 is rosuvastatin; if statin 1 is rosuvastatin, statin 2 is atorvastatin.*

For background LMT, including statins, sites must follow the national product label for the safety monitoring and management of patients.

LMT (whether administered as prescription drugs or over the counter) will be recorded in the CRF and source data. However, detailed use of nutraceutical products (such as plant stanols found in Benecol, flax seed oil, psyllium or LMT found in multivitamins) will not be collected in the CRF but should be maintained in source documents.

8.8.2 Other Concomitant Medications

All other concomitant medication(s) are allowed.

All patients should receive contemporary evidence-based treatment for ACS and chronic CHD as described in regional professional guidelines, including, but not limited to anti-platelet agents, beta blockers, angiotensin-converting-enzyme (ACE) inhibitors or angiotensin-receptor blockers (ARBs), and treatments for diabetes, hypertension, and smoking.

Concomitant medications (from a prescription or over-the-counter) that are administered chronically, as well as those that are administered during hospitalizations and considered relevant (i.e. pertaining to the patient's background or current medical history, or related to an AE/AESI/SAE, and as determined by Investigator's best judgment) are allowed and will be

recorded in the e–CRF and source data. Use of some medications related to efficacy may be collected only in (cardiovascular) efficacy endpoint eCRF pages (e.g. use of diuretics for CHF).

Other medications administered during hospitalizations and considered not relevant [including but not limited to: PRN (i.e. as needed) medications, anesthetic agents, medications given related to the performance of a procedure, IV fluids] as well use of multivitamins or nutraceuticals will not be recorded in the eCRF, however should be maintained in the source documents.

8.9 MITIGATION PLAN IN CASE OF IMP ISSUE

In the exceptional case of a major IMP issue, the following mitigation plan may be implemented in order to achieve the dual goal of optimizing IMP inventory at all sites, while maintaining patient treatment continuity with IMP:

From M24 (V16) and at all subsequent clinic visits (M30, M36, M42, M48, M54, and M60), a reduced number of IMP autoinjectors would be dispensed (12 instead of 18) covering 24 weeks of treatment. As a consequence, subsequent clinic visits would need to be scheduled in a more tightly manner and at a slightly shorter interval, ie, every 22 to 24 weeks (161d ±7d), instead of every 26w ±14d.

9 ASSESSMENT OF INVESTIGATIONAL MEDICINAL PRODUCT

9.1 EFFICACY ENDPOINTS

Any suspected cardiovascular event suggestive of an endpoint as well as all deaths will be submitted to the Clinical Events Committee (CEC). The CEC will review the data of the reported cases in a blinded manner for adjudication purpose and will validate if the event should be considered as an endpoint. The cardiovascular events adjudicated and validated by the CEC will be used for the analyses.

Events that the CEC could not classify as well as suspected event according to investigator but not confirmed by the CEC will not be part of the outcomes.

9.1.1 Primary efficacy endpoint

The primary efficacy endpoint is the time from randomization to first occurrence of one of the following clinical events, as determined by the CEC:

- CHD death (including "undetermined causes of death" as per the CEC)
- Any non-fatal MI
- Fatal and non-fatal ischemic stroke (including "stroke not otherwise specified" as per the CEC)
- Unstable angina requiring hospitalization

If none of these events is observed at the time of the analysis cut-off date (final or interim, depending of the analysis, see Section 11.4.2.1 for details), the patient will be censored at the date of last contact, at the date of death, or at the date of cut-off, whichever comes first.

Of note, suspected event according to the investigator but not confirmed by the CEC will not be part of the primary efficacy outcome; their description will be provided separately.

9.1.2 Secondary efficacy endpoints

Time-to-event secondary endpoints will be censored using the same methodology as for the primary efficacy endpoint.

9.1.3 Main Secondary Efficacy Endpoint(s):

- Time from randomization to first occurrence of any CHD event (major CHD event, unstable angina requiring hospitalization, ischemia-driven coronary revascularization procedure)
- Time from randomization to first occurrence of any major CHD event (CHD death, non-fatal MI)

- Time from randomization to first occurrence of any CV event defined as follows: any non-fatal CHD event, any CV death, and non-fatal ischemic stroke
- Time from randomization to first occurrence of all-cause mortality, non-fatal MI, non-fatal ischemic stroke
- Time from randomization to death (all-cause mortality)

9.1.4 Other Secondary Efficacy Endpoint(s):

- Component of the primary endpoint considered individually:
  - Time from randomization to CHD death
  - Time from randomization to first occurrence of any non-fatal MI
  - Time from randomization to first occurrence of fatal or any non-fatal ischemic stroke
  - Time from randomization to first occurrence of any unstable angina requiring hospitalization
- Time from randomization to first occurrence of any ischemia-driven coronary revascularization procedure
- Time from randomization to first occurrence of any congestive heart failure requiring hospitalization

9.1.5 Efficacy assessment methods

Definitions of the primary and secondary efficacy endpoints related to CV events and death are based on FDA/CDISC *Standardized Definitions for End Point Events in Cardiovascular Trials,* and on the Thygesen Universal Definition for the definition of myocardial infarction (34) (35) (36).

9.1.6 Definitions of components of the composite primary efficacy endpoint

9.1.6.1 Coronary Heart Disease (CHD) Death

Coronary Heart Disease Death is defined as the subset of Cardiovascular deaths for which there is a clear relationship to underlying coronary heart disease, including death secondary to acute MI, sudden death, heart failure, complication of a coronary revascularization procedure performed for symptoms, coronary disease progression, or new myocardial ischemia where the cause of death is clearly related to the procedure, unobserved and unexpected death, and other death that cannot definitely be attributed to a nonvascular cause (see Section 9.1.7.1 for the different definitions).

9.1.6.1.1 Death due to acute myocardial infarction

Death by any mechanism (arrhythmia, heart failure, low output) within 30 days after a myocardial infarction (MI) related to the immediate consequences of the myocardial infarction, such as progressive congestive heart failure (CHF), inadequate cardiac output, or refractory arrhythmia. If these events occur after a “break” (eg, a CHF and arrhythmia free period of at least a week), they

should be designated by the immediate cause, even though the MI may have increased the risk of that event (eg, late arrhythmic death becomes more likely after an acute myocardial infarction (AMI)). The acute myocardial infarction should be verified to the extent possible by the diagnostic criteria outlined for acute myocardial infarction or by autopsy findings showing recent myocardial infarction or recent coronary thrombus.

Sudden cardiac death, if accompanied by symptoms suggestive of myocardial ischemia, new ST elevation, new LBBB, or evidence of fresh thrombus by coronary angiography and/or at autopsy should be considered death resulting from an acute myocardial infarction, even if death occurs before blood samples or 12-lead electrocardiogram (ECG) could be obtained, or at a time before the appearance of cardiac biomarkers in the blood.

Death resulting from a procedure to treat a myocardial infarction such as percutaneous coronary intervention (PCI), coronary artery bypass graft surgery (CABG), or to treat a complication resulting from myocardial infarction, should also be considered death due to acute MI.

Death resulting from an elective coronary procedure to treat myocardial ischemia (ie, chronic stable angina) or death due to a MI that occurs as a direct consequence of a CV investigation/procedure/operation should be considered as a death due to a CV procedure.

9.1.6.1.2 Sudden cardiac death

Death that occurs unexpectedly, not following an acute MI, and includes the following deaths:

a) Death witnessed and occurring without new or worsening symptoms
b) Death witnessed within 60 minutes of the onset of new or worsening cardiac symptoms, unless documented (ie by ECG or other objective) to be due to acute myocardial infarction
c) Death witnessed and attributed to an identified arrhythmia (eg, captured on an electrocardiographic (ECG) recording, witnessed on a monitor, or unwitnessed but found on implantable cardioverter-defibrillator review)
d) Death after unsuccessful resuscitation from cardiac arrest
e) Death after successful resuscitation from cardiac arrest and without identification of a non-cardiac etiology
f) Unwitnessed death without other cause of death (information regarding the patient's clinical status preceding death should be provided, if available)

General considerations

A subject seen alive and clinically stable 24 hours prior to being found dead without any evidence or information of a specific cause of death should be classified as "sudden cardiac death."

Typical scenarios include:

- Subject well the previous day but found dead in bed the next day
- Subject found dead at home on the couch with the television on

Deaths for which there is no information beyond 'Patient found dead at home' may be classified as 'Death due to other cardiovascular causes'.

9.1.6.2 Non-fatal myocardial infarction (MI)

Definition for myocardial infarction is based on the most recent Thygesen Universal Definition (37):

9.1.6.2.1 Acute myocardial infarction

The term acute myocardial infarction (MI) refers to evidence of myocardial necrosis in a clinical setting consistent with acute myocardial ischemia. Under these conditions any one of the following criteria meets the diagnosis for MI:

a) Detection of a rise and/or fall of cardiac biomarker values [preferably cardiac troponin (cTn)] with at least one value above the upper reference limit (URL) and with at least one of the following:
    - Symptoms of ischemia
    - New or presumed new significant ST-segment–T wave (ST–T) changes or new left bundle branch block (LBBB).
    - Development of pathological Q waves in the ECG
    - Imaging evidence of new loss of viable myocardium or new regional wall motion abnormality
    - Identification of an intracoronary thrombus by angiography or autopsy
b) Cardiac death with symptoms suggestive of myocardial ischaemia and presumed new ischemic ECG changes or new LBBB, but death occurred before cardiac biomarkers were obtained, or before cardiac biomarker values would be increased.
c) Percutaneous coronary intervention (PCI) related MI is defined by elevation of cTn values (>5 x URL) occurring within 48h of the procedure in patients with normal baseline values (≤URL) or a rise of cTn values >20% if the baseline values are elevated and are stable or falling. In addition, at least one of the following is required:
    - Symptoms suggestive of myocardial ischemia
    - New ischemic ECG changes
    - Angiographic findings consistent with a procedural complication
    - Imaging demonstration of new loss of viable myocardium or new regional wall motion abnormality

d) Stent thrombosis associated with MI when detected by coronary angiography or autopsy in the setting of evidence of myocardial ischemia and with a rise and/or fall of cardiac biomarker values with at least one value above the URL.

e) Coronary artery bypass grafting (CABG) related MI is defined by elevation of troponin values (>10 x URL) occurring within 48h of the procedure in patients with normal baseline cTn values (≤URL). In addition at least one of the following is required:

- New pathological Q waves or new LBBB
- Angiographic documented new graft or new native coronary artery occlusion
- Imaging evidence of new loss of viable myocardium or new regional wall motion abnormality

Cardiac troponin is the preferred biomarker for diagnosis of MI. In absence of troponin, CK-MB will be used.

9.1.6.2.2 Silent myocardial infarction

Silent MI is not considered part of the primary endpoint.

Asymptomatic patients who develop new pathologic Q wave criteria for MI detected during routine ECG follow-up, or reveal evidence of MI by cardiac imaging, that cannot be directly attributed to a coronary revascularization procedure, are termed ‘silent MI”. Any one of the following criteria meets the diagnosis:

- Pathological Q waves with or without symptoms in the absence of non-ischemic causes
- Imaging evidence of a region of loss of viable myocardium that is thinned and fails to contract, in the absence of a non-ischemic cause
- Pathological findings of a prior MI

9.1.6.2.3 Classification according to Universal MI definition subtypes

All MI events will be classified by Universal MI definition subtypes as follows:

- Type 1

Spontaneous myocardial infarction related to atherosclerotic plaque rupture, ulceration, fissuring, erosion, or dissection with resulting intraluminal thrombus in one or more of the coronary arteries leading to decreased myocardial blood flow or distal platelet emboli with ensuing myocyte necrosis. The patient may have underlying severe coronary artery disease (CAD) but on occasion non-obstructive or no CAD.

- Type 2

In instances of myocardial injury with necrosis where a condition other than CAD contributes to an imbalance between myocardial oxygen supply and/or demand, eg, coronary endothelial dysfunction, coronary artery spasm, coronary embolism, tachy/brady-arrhythmias, anemia,

respiratory failure, hypotension, and hypertension with or without left ventricular hypertrophy (LVH).

- Type 3

Cardiac death with symptoms suggestive of myocardial ischemia and presumed new ischemic ECG changes or new LBBB, but death occurring before blood samples could be obtained, before cardiac biomarker could rise, or in rare cases cardiac biomarkers were not collected.

- Type 4a

Myocardial infarction associated with PCI.

- Type 4b

Myocardial infarction associated with stent thrombosis.

- Type 4c

Myocardial infarction associated with restenosis (restenosis is the only angiographic explanation)

- Type 5

Myocardial infarction associated with CABG.

9.1.6.2.4 Sub-classifications

a) Sub-classifications into STEMI versus NSTEMI

All MI events will be sub-classified into STEMI versus NSTEMI as follows:

- STEMI: new ST elevation at the J point in two contiguous leads with the cut-points: ≥0.1 mV in all leads other than leads V2–V3 where the following cut points apply: ≥0.2 mV in men ≥40 years; ≥0.25 mV in men <40 years, or ≥0.15 mV in women.
- NSTEMI: if ECG does not meet STEMI criteria will be classified as NSTEMI
- If ECGs are unavailable or uninterpretable the MI will be classified as unknown

b) Sub-classifications into Q wave versus Non Q wave MI

MI events will be sub-classified into Q wave vs. Non Q wave MI as follows:

Criteria for abnormal Q-waves are any one of:

- Any Q wave in leadsV2–V3 ≥0.02 sec or QS complex in leads V2 and V3
- Q wave ≥0.03 sec and ≥0.1 mV deep or QS complex in leads I, II, aVL, aVF or V4–V6 in any two leads of a contiguous lead grouping (I, aVL; V1–V6; II, III, aVF)[a]

  [a] *The same criteria are used for supplemental leads V7-V9.*

- R wave ≥0.04 sec in V1–V2 and R/S ≥1 with a concordant positive T wave in absence of conduction defect

- If Q-waves criteria are not met, MI is classified as non-Q-wave MI
- If ECGs are unavailable or uninterpretable the MI will be classified as unknown

9.1.6.3 Fatal and Non-fatal stroke

Stroke is defined as an acute episode of focal or global neurological dysfunction caused by central nervous system (CNS) vascular injury as a result of hemorrhage or infarction. CNS includes brain, spinal cord and retina.

Classification:

a) Ischemic Stroke

Ischemic stroke is defined as an acute episode of focal cerebral, spinal, or retinal dysfunction caused by CNS infarction. Evidence of infarction is defined as:

- Pathological, imaging, or other objective evidence of acute cerebral, spinal cord, or retinal focal ischemic injury in a defined vascular distribution; or
- In the absence of the above (i.e. imaging or autopsy unavailable), clinical evidence of cerebral, spinal cord, or retinal focal ischemic injury is based on symptoms persisting ≥24 hours or until death, and other etiologies excluded

NOTE: *Hemorrhagic infarction, defined as parenchymal hemorrhage after CNS infarction, is considered an ischemic stroke (i.e. hemorrhagic conversion of infarction) and is part of the primary endpoint.*

Ischemic strokes may be further classified according to most likely etiology (example large artery atherosclerosis, cardio-embolic, etc.)

b) Cerebral Hemorrhage

Hemorrhages in the CNS are classified as stroke if they are nontraumatic, caused by a vascular event, and result in injury to the CNS. In contrast, traumatic hemorrhages will not be characterized as stroke. Subdural hematoma will not be classified as a stroke.

The diagnoses included in this section are intracerebral hemorrhage (intraparenchymal and intraventricular) and subarachnoid hemorrhage (both aneurysmal and nonaneurysmal).

Diagnoses included in this section are not part of the primary endpoint.

- Stroke caused by intracerebral hemorrhage

  Rapidly developing clinical signs of neurological dysfunction (focal or global) attributable to a focal collection of blood within the brain parenchyma or ventricular system that is not caused by trauma.

- Stroke caused by subarachnoid hemorrhage

  Rapidly developing signs of neurological dysfunction (focal or global) and/or headache because of bleeding into the subarachnoid space (the space between the arachnoid membrane and the pia mater of the brain or spinal cord), which is not caused by trauma.

Hemorrhages may be further classified according to location (example, supratentorial, subtentorial, intraparenchymal etc.)

c) Stroke not otherwise specified

An episode of acute neurological dysfunction presumed to be caused by ischemia or hemorrhage, persisting ≥24 hours or until death, but without sufficient evidence to be classified as one of the above.

Strokes not otherwise specified are part of the primary endpoint.

A functional disability assessment will be performed for strokes after 3 to 6 months following the start date of the event (see Appendix I).

Fatal stroke

Death due to Stroke refers to death after a stroke that is either a direct consequence of the stroke or a complication of the stroke. Acute stroke should be verified to the extent possible by the diagnostic criteria outlined for stroke.

9.1.6.4 Unstable angina requiring hospitalization

A diagnosis of unstable angina (new ACS event without elevations in cardiac biomarkers) that meets the primary endpoint requires the following:

- Admission to hospital or emergency room (until at least next calendar day) with symptoms presumed to be caused by myocardial ischemia with an accelerating tempo in the prior 48 hrs and/or prolonged (at least 20 min) rest chest discomfort

AND

- New high-risk ECG findings consistent with ischemia or infarction (or presumed new if no prior ECG available) as defined below:
  - New or presumed new ST depression >0.5mm in 2 contiguous leads or T wave inversion >1mm in leads with prominent R wave or R/S >1 in 2 contiguous leads, OR
  - New or presumed new ST elevation at the J point in > 2 contiguous leads >0.2mV in V2 or V3 in men or >0.15 mV in women in V2 or V3 or >0.1mV in other leads.
  - LBBB (new or presumed new)

AND

- Definite contemporary evidence (defined below) of angiographically significant coronary disease as demonstrated by:
  - Need for coronary revascularization procedure (PCI or CABG) excluding those performed to treat only restenosis lesion(s) at previous PCI site(s) **OR**
  - Angiographic evidence of at least one significant (≥ 70%) epicardial coronary stenosis not due to restenosis at previous PCI site

The coronary revascularization procedure or the diagnostic angiography must have been performed during the hospitalization for that event.

9.1.7 Definitions of the secondary efficacy endpoints

9.1.7.1 Death

All deaths will be categorized as Cardiovascular, non-Cardiovascular or Undetermined based on the definitions below. In addition, all deaths will also be categorized as Coronary Heart Disease Death and further sub-typed based on the specific Cardiovascular and non-Cardiovascular categories defined below.

9.1.7.1.1 Definition of Cardiovascular Death

Cardiovascular Death is defined as death resulting from an acute myocardial infarction, sudden cardiac death, death due to heart failure, death due to stroke, death due to cardiovascular (CV) procedures, death due to CV hemorrhage, and death due to other cardiovascular causes.

9.1.7.1.1.1 Coronary Heart Disease (CHD) Death

Definition of CHD death is described in Section 9.1.6.1.

9.1.7.1.1.2 Other Cardiovascular Deaths

Death due to Heart Failure or Cardiogenic Shock:

Death due to Congestive Heart Failure refers to a death in association with clinically worsening symptoms and/or signs of heart failure not following an acute MI. Deaths due to heart failure can have various etiologies, including single or recurrent myocardial infarctions, ischemic or non-ischemic cardiomyopathy, hypertension, or valvular disease.

Cardiogenic shock **not** occurring in the context of an acute myocardial infarction or as the consequence of an arrhythmia occurring in the absence of worsening heart failure.
Cardiogenic shock is defined as systolic blood pressure (SBP) <90 mm Hg for greater than 1 hour, not responsive to fluid resuscitation and/or heart rate correction, and felt to be secondary to cardiac dysfunction and associated with at least one of the following signs of hypoperfusion:

- Cool, clammy skin or
- Oliguria (urine output <30 mL/hour) or
- Altered sensorium or
- Cardiac index <2.2 L/min/m²

Cardiogenic shock can also be defined if SBP <90 mm Hg and increases to ≥90 mm Hg in less than 1 hour with positive inotropic or vasopressor agents alone and/or with mechanical support.

Death due to Stroke (see Section 9.1.6.3 for the definition of fatal stroke).

Death due to Cardiovascular Procedures

Death due to Cardiovascular Procedures refers to death caused by the immediate complications of a cardiac procedure and excludes death resulting from procedures to treat an acute MI or the complications resulting from an acute MI.

Death due to Cardiovascular Hemorrhage

Death due to Cardiovascular Hemorrhage refers to death related to hemorrhage such as a non-stroke intracranial hemorrhage, non-procedural or non-traumatic vascular rupture (eg, aortic aneurysm), or hemorrhage causing cardiac tamponade.

Death due to Other Cardiovascular Causes:

Death due to Other Cardiovascular Causes refers to a cardiovascular death not included in the above categories (e.g., pulmonary embolism or peripheral arterial disease).

9.1.7.1.2 Definition of Non-Cardiovascular Death

Non-cardiovascular death is defined as any death that is not thought to be due to a cardiovascular (CV) cause. The following categories may be collected.

9.1.7.1.2.1 Non-Malignant Causes

- Pulmonary
- Renal
- Gastrointestinal
- Hepatobiliary
- Pancreatic
- Infection (includes sepsis)
- Non-infectious (eg, systemic inflammatory response syndrome [SIRS])
- Hemorrhage* excluding hemorrhagic strokes and bleeding in the setting of coronary revascularization

- Non-cardiovascular procedure or surgery
- Accidental (e.g. physical accidents or drug overdose) or Trauma
- Suicide
- Prescription drug error (e.g. prescribed drug overdose, use of inappropriate drug, or drug-drug interaction)
- Neurological process that is not a stroke or hemorrhage
- Other non-cardiovascular, specify: _________________

*Examples:
Death due to GI bleeding is not considered a CV death. Death due to retroperitoneal hematoma following PCI is considered CV death. Death due to intracerebral hemorrhage is considered CV death.

9.1.7.1.2.2 Malignant Causes

- Death results directly from the cancer; **OR**
- Death results from a complication of the cancer (e.g., infection, complication of surgery / chemotherapy / radiotherapy); **OR**
- Death results from withdrawal of other therapies because of concerns relating to the poor prognosis associated with the cancer

Cancer deaths may arise from cancers that were present prior to randomization or which developed subsequently. Those cancer deaths should be further classified (worsening prior malignancy; new malignancy).

9.1.7.1.3 Definition of Undetermined Cause of Death

Undetermined cause of death refers to a death not attributable to one of the above categories of cardiovascular death or to a non-cardiovascular cause, due to absence of any information (eg, the only available information is "patient died"). The use of this category of death is discouraged and should apply to a minimal number of cases when no information at all on the circumstances of death is available (ie, found on obituary of local newspaper). In all circumstances the reviewer will use all available information to attribute to one of the categories based on best clinical judgment.

9.1.7.2 Ischemia-driven coronary revascularization procedure

'Ischemia-driven coronary revascularization' includes all coronary revascularization procedures (PCI/CABG) performed during the study, and driven by new or presumed new myocardial ischemia since randomization (categories 1 and 2 described below), and excluding procedures performed only to treat restenosis lesion(s) at prior PCI site(s).

Reasons for the PCI/CABG will be collected in the CRF as follows:

1. driven by acute ischemia (ACS).
2. driven by new/progressive (i.e. not present at randomization or with indication of progression since randomization), chronic (ie not in context of ACS) ischemia, evidenced by new/progressive symptoms (angina or equivalent) or new/progressive functional testing abnormalities (e.g. stress test, imaging).
3. other (i.e. not driven by ACS event, or by new/progressive chronic ischemia).

PCI involves a catheter-based tool (eg, balloon catheters, cutting balloons, atherectomy devices, lasers, bare metal stents, and drug-eluting stents) that improves myocardial blood flow by increasing the luminal area at a site of an obstructive coronary lesion. Coronary artery bypass grafting (CABG) is an open surgical procedure designed to improve myocardial blood flow by providing a conduit (arterial, venous, or synthetic) for blood flow distal to an obstructive coronary lesion. Insertion of a guidewire through a coronary guide catheter into a coronary vessel or aortocoronary bypass graft for the purpose of percutaneous coronary intervention (PCI) is considered as a PCI (since there is an intention to perform a PCI). However, insertion of a guidewire in order to assess the severity of intermediate lesions with the use of intravascular ultrasound, Doppler flow velocity, or fractional flow reserve, will NOT be considered PCI.

9.1.7.3 Congestive Heart Failure requiring hospitalization.

Congestive Heart Failure (CHF) requiring hospitalization is defined as an event that meets ALL of the following criteria:

1) The patient is admitted to the hospital or emergency room (until at least next calendar day) with a primary diagnosis of CHF
2) The patient exhibits documented new or worsening symptoms due to CHF on presentation, including at least ONE of the following:
   a) Dyspnea (dyspnea with exertion, dyspnea at rest, orthopnea, paroxysmal nocturnal dyspnea)
   b) Decreased exercise tolerance
   c) Fatigue
   d) Other symptoms of worsened end-organ perfusion or volume overload
3) The patient has objective evidence of new or worsening CHF, consisting of at least TWO physical examination findings OR one physical examination finding and at least ONE laboratory/imaging/hemodynamic criterion), including:
   a) Physical examination findings considered to be due to heart failure, including new or worsened:
      - Peripheral edema.
      - Increasing abdominal distension or ascites (in the absence of primary hepatic disease)

- Pulmonary rales/crackles/crepitations
- Increased jugular venous pressure and/or hepatojugular reflux
- S3 gallop
- Clinically significant or rapid weight gain thought to be related to fluid retention

b) Laboratory, imaging or hemodynamic evidence of new or worsening CHF, including:

- Increased B-type natriuretic peptide (BNP)/ N-terminal pro-BNP (NT-proBNP) concentrations consistent with decompensation of heart failure. In patients with chronically elevated natriuretic peptides, a significant increase should be noted above baseline
- Radiological evidence of pulmonary congestion
- Hemodynamic evidence from right-heart catheterization (e.g. elevated pulmonary capillary wedge pressure, elevated central venous pressure, or low cardiac index) or from left heart catheterization (elevated left ventricular end-diastolic pressure)

4) The patient receives initiation or intensification of treatment specifically for HF, including at least ONE of the following:

a) Significant augmentation in oral diuretic therapy

b) Intravenous diuretic, inotrope, or vasodilator therapy

c) Mechanical or surgical intervention, including:

- Mechanical circulatory support (eg, intra-aortic balloon pump, ventricular assist device)
- Mechanical fluid removal (eg, ultrafiltration, hemofiltration, dialysis)

9.2 SAFETY ENDPOINT(S):

Observation period

The observation of safety data will be as follows:

- PRE-TREATMENT period: The PRE-TREATMENT observation period is defined from the signed informed consent up to the first dose of double-blind IMP injection
- Treatment Emergent Adverse Event (TEAE) period: The TEAE observation period is defined as the time from the first dose of double-blind IMP injection to the last dose of IMP injection + 70 days (10 weeks) as residual effect of treatment is expected until 10 weeks after the stop of double-blind IMP
- POST-TREATMENT period: The POST-TREATMENT observation period is defined as the time starting the day after the end of the TEAE period up to the end of the study

Rationale for TEAE period definition is detailed in Section 4.

9.2.1 Adverse event

All adverse events diagnosed by the Investigator, irrespective of the result of the adjudication for cardiovascular events, will be reported and described.

All AEs will be coded to a "Lowest Level Term (LLT)", "Preferred Term (PT)", "High Level term (HLT)", "High Level Group Term (HLGT)" and associated primary "System Organ Class (SOC)" using the version of MedDRA (Medical Dictionary for Regulatory Activities) currently in effect at Sanofi at the time of the considered database lock.

AEs of special interest include, but are not limited to, the following:

- Allergic events (using special eCRF pages, see Section 10.6.2)
- Local injection site reactions (using special eCRF pages, see Section 10.6.1)
- Hemolytic anemia (using special eCRF pages, see Section 10.4.7.1)

Adverse event observation period

The AE observations are per the observation periods defined above.

Death observation period

The death observations are per the observation periods defined above.

9.2.2 Safety laboratory

The clinical laboratory data consist of hematology (red blood cell count, reticulocyte count, hemoglobin, hematocrit, platelets, white blood cell count [WBC] with differential blood count), standard chemistry (glucose, sodium, potassium, chloride, bicarbonate, calcium, phosphorous, urea nitrogen, creatinine, uric acid, LDH, total protein, albumin, γ Glutamyl Transferase [γGT]), Hepatitis C antibody, liver panel (ALT, AST, alkaline phosphatase [ALP], and total bilirubin), and CPK.

Some additional safety laboratory parameters may be reflexively measured, based on actual data (please refer to Section 10.4.7).

Clinical laboratory values will be analyzed after conversion into standard international units. Standard international units will be used in all listings and tables.

9.2.3 Vital signs measurement

Vital signs include: weight, heart rate, systolic and diastolic blood pressure in sitting position.

9.2.4 Electrocardiogram measurement

Electrocardiogram (ECG) assessments will be described as normal or abnormal.

(electronic 1.0)

9.3 OTHER ENDPOINT(S):

9.3.1 Anti-alirocumab antibody assessments

Anti-alirocumab antibodies include the antibody status (positive/negative) and antibody titers.

9.3.1.1 Sampling time

Serum samples for anti-alirocumab antibody determination will be drawn periodically throughout the study as per schedule noted in the study flowchart – Section 1.2. The first scheduled sample at randomization visit will be obtained before IMP injection (pre-dose).

Patients who have titers at or above 240 for anti-alirocumab antibodies at the common study end date will have an additional antibody sample between 6 to 12 months after this date.

In patients who permanently discontinued blinded study treatment early, anti-alirocumab antibody assessments will be performed at the early end-of-treatment visit (V70) and at every subsequent clinic visit, where this assessment was planned.

9.3.1.2 Sampling procedure

Detailed procedure of sample preparation, storage and shipment will be described in the specific laboratory manual which will be provided to sites. Five (5) ml blood volume is to be collected for each anti-alirocumab antibody sample.

9.3.1.3 Bioanalytical method

All anti-alirocumab antibody samples will be analyzed by the Regeneron Sample Analysis group.

Anti- alirocumab antibody samples will be analyzed using a validated, non-quantitative, titer-based bridging immunoassay. It involves an initial screen, a confirmation assay based on drug specificity, and a measurement of the titer of anti-alirocumab antibodies in the sample. [redacted]

Samples that are positive in the ADA assay will be assessed for neutralizing antibodies using a validated, non-quantitative, competitive ligand binding assay [redacted]

9.3.2 Lipid parameters

9.3.2.1 Endpoints

The percent changes from baseline to Month 4, to Month 24, and to the final analysis cutoff date for the following parameters:

- Calculated LDL-C
- ApoB
- Non-HDL-C

All measurements (scheduled or unscheduled, fasting or not fasting) may be used to provide a value for the endpoint considered, even if assessed after patient's discontinuation to the study treatment (Intent-To-Treat [ITT] approach). The analysis windows used to allocate a time point to a measurement will be defined in the Statistical Analysis Plan (SAP).

9.3.2.2 Assessment method

LDL-C will be calculated using the Friedewald formula (38). In case of calculated LDL-C <15 mg/dL (0.39 mmol/L), LDL-C value will be confirmed with direct measurement. If the TG values exceed 400 mg/dL (4.52 mmol/L), the central lab will reflexively measure the LDL-C rather than calculating it. Direct LDL-C measurement will be done via the beta quantification method. Apo B will be directly measured by the Central Laboratory. Non-HDL-C will be calculated by subtracting HDL-C from the total-C. Detailed procedures of sample preparation, storage and shipment will be described in the specific laboratory manual which will be provided to sites. Lipids parameters will be assessed from screening to common study end date.

9.3.3 hs-CRP

The percent change in hs-CRP from baseline up to the common study end date.

9.3.4 HbA$_{1C}$

The absolute change in HbA$_{1c}$ (%) from baseline up to the common study end date.

9.3.5 EQ-5D Patient Questionnaire

EQ-5D is a standardized measure of health status developed by the EuroQol Group in order to provide a simple, generic measure of health for clinical and economic appraisal. The EQ-5D as a measure of health-related quality of life, defines health in terms of 5 dimensions: mobility, self-care, usual activities, pain/discomfort, anxiety/depression. Each dimension can take one of three responses (3 ordinal levels of severity): 'no problem' (1). "some problems" (2). "severe problems" (3). Overall health state is defined as a 5-digit number. Health states defined by the 5-dimensional classification can be converted into corresponding index scores that quantify health status, where

0 represents 'death' and 1 represents “perfect health” (See Appendix G). If response to one or more dimension is missing, the index score will be missing.)

EQ-5D variables include response of each EQ-5D items, index score and change of index score from baseline Week (39).

This questionnaire will only be administered in patients receiving the double-blind treatment. Patients who will prematurely discontinue will be asked to fill in this questionnaire until the early end of treatment visit (V70) to be performed at the time of discontinuation.

9.3.6 Pharmacogenomic Samples

An optional pharmacogenomic sub-study will be conducted to identify genetic associations with clinical or biomarker response to PCSK9 inhibition, hyperlipidemia, or cardiovascular disease. If needed, samples may also be used to identify markers associated with toxicity.

Randomized patients who agree to participate in the genomics sub-study will be required to sign a separate genomics sub-study informed consent form (ICF) prior to collection of the DNA sample. Blood for DNA extraction should be collected before IMP injection (pre-dose) on randomization visit; however, it could be collected at any time during the study. Patients who choose not to enroll in the genomics sub-study are still eligible to enroll in the primary study.

Special procedures for storage and shipping of pharmacogenomic samples are summarized below (Table 1) and are described in detail in Appendix B.

Table 1 - Summary of handling procedures for DNA storage samples

**Sample Type(s)**	Pharmacogenetics
**Blood Sample Volume**	6 mL
**Tube Type**	6 mL Becton Dickinson K2 EDTA VACUTAINER™ Plus tubes with HEMOGARD™ closure (PN367863/4) sterile tubes
**Anticoagulant**	K2 EDTA
**Blood Handling Procedures**	Keep blood on ice or frozen at -20°C (or colder) within 30 min of sampling time. DO NOT CENTRIFUGE.
**Storage Conditions**	In collection tube at approximately -20°C (or colder)

The Sponsor has included safeguards for protecting patient confidentiality. The blood sample and DNA that is extracted from it will be assigned a second number, a Genetic ID (de-identification code) that is different from the Subject ID. This “double coding” is performed to separate a patient’s medical information and DNA data. The clinical study data (coded by Subject ID) will be stored in a distinct database at a different location from the database containing the pharmacogenomic data (coded by Genetic ID). The key linking Subject ID and Genetic ID will be maintained by a third party, under appropriate access control. The matching of clinical data and pharmacogenomic data, for the purpose of data analysis, will be possible only by using this key,

which will be under strict access control. All data will be reported only in coded form in order to maintain confidentiality.

DNA may be stored and used for analyses for up to 15 years from the completion of the Clinical Study Report (CSR). Analyses may include sequence determination or single nucleotide polymorphisms (SNP) from candidate genes. Candidate genes may include (but are not limited to) PCSK9, Apo B and LDL-R. Genome-wide studies, including (but not limited to) SNP analyses and/or genomic sequencing may also be performed.

9.3.7 Cardiovascular events of interest (other than efficacy endpoints)

Clinically significant complications or procedures (not planned at the time of randomization) related to peripheral arterial disease (such as critical limb ischemia, amputation, peripheral revascularization) as well as venous thromboembolic events (deep vein thrombosis, pulmonary embolism) will be collected on the case report form.

10 STUDY PROCEDURES

The study consists of a run-in period of at least 2 weeks with randomization no earlier than 4 weeks (28 days) and no later than 52 weeks (+ 5 days) after the index ACS event. The double-blind, placebo-controlled treatment period will continue for 24 months after the closing of randomization ex-China or until the target number of events (1613) is reached whichever comes last (see beginning of Section 6). The corresponding estimated study duration is approximately 64 months. All randomized patients (whether they are still receving IMP at the end of the study, or had earlier permanent treatment discontinuation) should have a final visit (CSED visit) between the CSED and CSED + 30 days.

For all visits after Day 1/Month 0 (randomization visit), a timeframe of a certain number of days will be allowed. The window period for visits at Months 1 and 2 are ± 7 days, and for all other subsequent visits it is ± 14 days during the double-blind treatment period (NOTE: in case of major IMP issue, please refer to Section 8.9).

For all visits after Day 1/randomization visit, if one visit date is changed, then the next visit should take place according to the original schedule as outlined Section 1.2.

Ideally all the visits should take place in the morning approximately at the same time. However after randomization in case there is no other possibility for the patient, visits can be arranged later in the day.

For the phone calls/contacts via internet to be performed in between on-site/clinic visits, they should be scheduled with the goal of maintaining a contact with the patient about every 2 months during first 24 months, and about every 3 months thereafter. Having no phone or email contact with a patient for a prolonged period (>4 months) should be avoided as much as possible.

Blood samplings:

The blood sampling for determination of lipid parameters (eg, LDL-C, Apo B, and non-HDL-C) should be preferably performed in the morning, in fasting condition (ie overnight, at least 8 hours fast) for all site visits throughout the study.

Laboratory tests:

The laboratory data are collected in accordance with the study schedule in Section 1.2 and forwarded to the central laboratory:

- Hematology

  NOTE: *At selected sites with a longer transit time to Central Lab, a duplicate hematology sample will be collected for local assessment of reticulocyte count,*

- Chemistry

- Liver panel: in case of total bilirubin values above the normal range, differentiation into conjugated and non-conjugated bilirubin will occur automatically
- Lipids
- Creatine Phosphokinase (CPK).
- Hepatitis B surface antigen
- Hepatitis C antibody

  NOTE: *In case of positive hepatitis C antibody test, a blood sample will be collected at a subsequent visit and sent to Covance Central Lab for further confirmatory testing*
- Serum pregnancy test

Central Laboratory results will be provided to sites (with the exception of lipid levels collected post-randomization in order to maintain the double-blind nature of the study). An alert will be provided by the Central Laboratory to sites in case of 2 consecutive significant elevations of triglyceride levels. Table 2 summarizes the management of selected blood samples during the study.

Table 2 - Overview of selected blood samples during study

Blood samples	Collection	Results	Comment
Blood samples described above (other than lipids)	Collected periodically at study visits during run-in and post-randomization and sent to Central Laboratory	Results provided to sites	During run-in period, results used for qualification
Blood sample for lipids	Collected at study visits during run-in period and sent to Central Laboratory	Results provided to sites	Results at V2/V2b used for qualification (run-in period)
	Collected post-randomization periodically at study visits throughout the study and sent to Central Laboratory	Results not provided to sites (in order to maintain the blind)	- Results will be analyzed at the end of the trial<br>- Alert provided to sites by Central Laboratory for significant elevation of triglycerides (2 consecutive values)<br>- Low cholesterol values (and associated clinical safety) reviewed in unblinded manner by Independent Physician with oversight by DMC members (who may determine upon individual circumstances that a site should be alerted)<br>- No alert provided for high or elevation of cholesterol levels (see below)
	Additional lipid level assessments (at a local laboratory) should not be performed during entire study		- Rationale is that patient is already treated with maximal LMT regimen (statin-intensive and optimized for long-term chronic use) since study start<br>- Compliance to required background LMT regimen should be emphasized throughout study; LMT may be modified based on clinical tolerability considerations only – see Section

Blood samples	Collection	Results	Comment
			8.8.1<br>- Investigator should periodically remind and educate GP/cardiologist of study requirements (i.e. during entire study, GP/cardiologist should refrain from checking lipid levels at a local laboratory, and should not modify LMT regimen without discussing with Investigator)
Cardiac biomarkers	To be collected and analyzed locally, as needed(for assessment of suspected cardiac events), with results provided to sites		Results used for efficacy endpoints

Urine samplings:

- Urinalysis will be performed periodically (yearly) throughout the study in about 750 randomized patients, at selected sites. A urine sample will be sent to Covance Central Labs for macroscopic analysis (presence of blood, protein, glucose, ketones, nitrates, leukocyte esterase, uro-bilinogen and bilirubin). In case of abnormalities, Covance Central Lab will perform a microscopic analysis.
- Urine pregnancy test – dipstick will be performed on site.

NOTE*: Any clinically relevant abnormal laboratory value should be immediately rechecked (whenever possible using the central laboratory) for confirmation before making any decision for the concerned patient. It should be documented as an AE/SAE as applicable. Please also refer to Section 10.4.4 and Section 10.4.7.*

Decision trees for the management of certain laboratory abnormalities by Sanofi are provided in Appendix C and Appendix D

Other endpoints assessment methods

All other blood parameters will also be measured by a Central Laboratory during the study (as per the schedule in Section 1.2, on blood samples taken preferably in the morning in fasting condition (at least 8 hours fast).

- Glycemic parameters (HbA_{1c}) and serum glucose will be measured by a Central laboratory, periodically throughout the study as per the schedule in Section 1.2.
- The blood sampling for inflammatory parameter, hs-CRP will be collected periodically throughout the study as per the schedule in Section 1.2.

NOTE: *In case of high HbA_{1c} values at screening, the Investigator is responsible for the optimization of the patient's treatment to achieve HbA_{1c} targets as defined by local guidelines or the Standards of Medical Care in Diabetes-2012 by the American Diabetes Association (40).*

Library samples

Library (plasma and serum) samples should be collected, as permitted by local regulatory policies. They will be collected periodically throughout the study as per schedule noted in the study flowchart - Section 1.2. The first scheduled sample at randomization visit will be obtained before IMP injection (pre-dose).

Library samples will be coded to maintain patient confidentiality and may be stored for up to 10 years or as permitted by local regulatory policies, whichever is shorter, for exploratory research of PCSK9 levels, PCSK9 function, effect(s) of PCSK9 inhibition with a monoclonal antibody, lipoprotein sub-fraction, inflammation, and cardiovascular risk markers (eg, lipoprotein–associated phospholipase A2). If needed, samples may also be used to identify markers associated with toxicity. The library samples will never be used for genomic analysis.

Detailed procedure of sample preparation, storage and shipment will be described in the specific laboratory manual which will be provided to sites. Library samples will be sent to a central laboratory (only for randomized patients) for long-term storage between –70°C to –85°C.

- Plasma samples: 8.5 mL blood volume to be collected as specified in the specific laboratory manual
- Serum samples: 2.5 mL blood volume to be collected as specified in the specific laboratory manual

Physical examination:

A general physical examination should be performed at the time points indicated in the study schedule flowchart in Section 1.2. If a new clinically significant abnormality or worsening from baseline is detected after randomization, then an AE should be reported and the patient should be considered for further clinical investigations and/or specialist consultation as per the Investigator's medical judgment.

Blood pressure (BP)/heart rate:

BP should be measured in sitting position under standardized conditions.

Heart rate will be measured at the time of the measurement of blood pressure.

NOTE: *In case of high BP values at screening the Investigator is responsible for the optimization of the patient's treatment to achieve BP targets as defined by local guidelines or the Seventh Report of the Joint National Committee on Prevention, Detection, Evaluation, and Treatment of High Blood Pressure (JNC 7) (41).*

ECG:

The ECGs will be interpreted locally by the Investigator. Any clinically significant abnormality should be documented as an AE/SAE as applicable (see Section 10.4.4). All ECG traces will be kept as source data.

Body weight and height

Body weight should be obtained with the patient wearing undergarments or very light clothing and no shoes, and with an empty bladder.

Height needs to be measured as self-reported heights are not acceptable.

10.1 VISIT SCHEDULE

10.1.1 Screening visit - Visit 1 (at least 2 weeks prior to randomization visit) and entry in the run-in period

Visit 1 (V1) and Visit 2 (V2) can be separate visits or can be combined (see Section 6.1.1. - Run-in period.

- If V1 and V2 are separate visits, V1 should be scheduled between 0 and 50 weeks post index ACS event
- If V1 and V2 are combined, the combined V1/V2 visit should be scheduled between 0 and 50 weeks + 5 days post index ACS event

The patient will receive verbal information concerning the aims and methods of the study, its constraints and risks and the study duration. A written summary in the form of an information leaflet will be given to the patient. The written informed consent must be signed by the patient and the Investigator prior to any investigations. Only patients who meet the inclusion criteria as noted in Section 7 may be screened. Women of childbearing potential will be requested to use a medically approved contraceptive method during the entire study. If it is planned to have another designated person administer the injections to the patient during the study, then this person should be present for the first injection-training done at this visit V1.

This visit will include:

- Complete informed consent
- Assessment of inclusion and exclusion criteria
- Demographic (age, gender, race, ethnicity)
- Collect contact information (address, e mail, home and cell phone number) for patient, patient's family and patient's GP/cardiologist
- Patient's medical and surgical history (including menopausal status and cardiovascular history, and relevant family history e.g. allergy), alcohol habits, and smoking habits
- Patient's cardiovascular history
- Index ACS event: type of event and date of onset
- Record of previous LMT medications (related to statins, and non-statin LMTs) from prescription or over-the-counter within 1 month prior to screening; use of chronic statin treatment or not prior to index ACS event

- Record of concomitant medications, especially lipid-modifying treatments (statins and non-statin LMTs from prescription or over-the counter therapies as above), and cardiovascular medications
- Body weight and height measurements
- Physical examination including vital signs: sitting systolic and diastolic blood pressure (SBP and DBP), heart rate
- Collection of adverse events from this point onward:

All adverse events and serious adverse events will be collected from the time of informed consent signature and throughout the study until the common study end-date visit (V30).

- IVRS/IWRS contact for notification of screening and entry in the run-in period:
  - Patients meeting the inclusion/exclusion criteria for eligibility at screening will enter the run-in period of the study. IVRS/IWRS is to be contacted for notification of screening and for patient number allocation (please note that it is important to have the IVRS/IWRS contact before any blood sample is drawn because the patient number is given by IVRS/IWRS and it must be reported on the requisition forms). This patient number is composed of a 9-digit number containing the 3-digit country code, the 3-digit center code and the 3-digit patient chronological number (the 3-digit patient chronological number is 001 for the first patient screened in a center, 002 for the second patient screened in the same center...).
  - Allocation of a batch number for training kit
- Fasting blood sample for:
  - Lipids: TC, LDL-C, HDL-C, TG, non-HDL-C, ApoB
  - Hematology: blood cell count including hematocrit, hemoglobin, red blood cell count, white blood cell count with differential count and platelets
  - Chemistry: glucose, sodium, potassium, chloride, bicarbonate, calcium, phosphorous, urea nitrogen, creatinine, uric acid, LDH, total protein, albumin, and γGT
  - Liver panel (ALT, AST, ALP, and total bilirubin).
  - HbA_{1c}
  - CPK
  - Hepatitis B surface antigen and hepatitis C antibody tests

    NOTE: *In case of positive hepatitis C antibody test, a blood sample will be collected at a subsequent visit and sent to Covance Central Lab for further confirmatory testing*
  - Serum pregnancy test (females of childbearing potential only)

- First potential injection-training on site (see also Section 8.1.2):
  - Injection-training should be provided as outlined in Section 8.1.2
  - The placebo should be administered by the patient or another designated person (such as spouse, relative, etc...) at the study site under supervision of site staff with appropriate feedback
  - Record batch number allocated in eCRF
- An appointment will be given for the next visit V2 after required LMT regimen (statin-intensive, and optimized for long-term chronic use with addition of non-statin LMT, at Investigator's discretion) has been administered for at least two weeks, was found well tolerated and no further increase in LMT regimen is planned.

If necessary one additional visit (V2b) and central laboratory assessments may occur during the run-in period (see Section 6.1.1.2.3)

The patient should be seen for the next visit in the morning and preferably in fasting condition (ie, overnight, at least 8 hours fast).

10.1.2 Qualifying visit - Visit 2

V2 should be scheduled after required LMT regimen was administered at stable dose for at least 2 weeks and found well tolerated (see Section 6.1.1.3).

- If V1 and V2 are separate visits, the interval between V1 and V2 should be at least 2 weeks (14 days)
- Alternatively, V1 and V2 may be combined in one visit (see Section 10.1.3)

This visit will include:

- Update contact information (address, e mail, home and cell phone number) for patient, patient's family and patient's GP/cardiologist
- Assessment of inclusion and exclusion criteria
- Record of concomitant medication (including but not limited to LMT regimen used during the 2 weeks prior to V2)

  NOTE: In case of rescreening, reason for potential different statin dose/regimen used during the 2 weeks prior to V2/Screening 2 (as compared to the 2 weeks prior to V2/Screening 1) will be recorded
- Vital signs: SBP and DBP, heart rate
- Collection of adverse events
- Check of the compliance and stability of the dose of the LMT (atorvastatin, rosuvastatin, ± other LMT)
- Fasting blood sample for qualifying lipid labs:
  - Lipids: TC, LDL-C, HDL-C, TG, non-HDL-C, ApoB

- Potential injection-training on site:
  - IVRS/IWRS contact for allocation of a batch number for training kit
  - Injection-training should be provided as outlined in Section 8.1.2
  - The placebo should be administered by the patient or another designated person (such as spouse, relative, etc...) at the study site under supervision of site staff with appropriate feedback
  - Record batch number allocated in eCRF
- An appointment will be given for the next visit (V3 randomization) in 2 to 5 days, after results of V2 labs are obtained
  - Remind patient to be in fasting conditions (ie, overnight, at least 8 hours fast) for next visit
  - The patient will be instructed to continue the LMT at the same dose

10.1.3 Combined V1/V2 Visit

If required LMT regimen (see Section 6.1.1.3) was already administered at stable dose prior to V1 for ≥ 2 weeks and found well tolerated, V1 and V2 visits can be combined as one visit (combined V1/V2 visit), with all assessments of V1 and V2 visits to be performed at this combined visit.

NOTE: *Prior to conducting a combined V1/V2 visit, prior LMT (and, if applicable, intolerance to statin) must be adequately documented in a source document such as a discharge summary, history and physical, clinic note or consultation report to confirm treatment.*

10.1.4 Optional qualifying visit - Visit 2b

See Section 6.1.1.2.3 for circumstances in which this visit should be performed.

This visit is similar to the Visit V2, and a training injection can be performed.

- Third (potential) training injection as necessary
  - IVRS/IWRS contact for allocation of a batch number for training kit
  - Injection-training should be provided as outlined in Section 8.1.2
  - The placebo should be administered by the patient or another designated person (such as spouse, relative, etc...) at the study site under supervision of site staff with appropriate feedback
  - Record batch number allocated in eCRF
- An appointment will be given for the next visit V3 (randomization) – See Section 10.1.5.1.1
  - Remind patient to be in fasting conditions (ie overnight, at least 8 hours fast) for next visit

10.1.5 Double-blind treatment period

10.1.5.1 Study site visits from Visit 3 (Month 0, D1) to Visit 30 (Month 64)

10.1.5.1.1 Baseline (randomization) visit - Visit 3 (Month 0, D1)

V3 should be scheduled as follows:

- If V1 and V2 are separate visits (≥ 2 weeks between V1 and V2): V3 (randomization) can occur as soon as the qualifying lipid lab results from V2 are obtained from the central lab (typically final results are available within 2-5 days of collection)
- In case of combined V1/V2 visit, after qualifying lipid labs are collected and if patient is eligible, V3 (randomization) should occur at least 2 weeks (14 days) after the combined V1/V2 visit
- In all cases, V3 (randomization) should occur no earlier than 4 weeks (28 days) and no later than 52 weeks (+ 5 days) post index ACS event
- Also, there should be no new ACS event or coronary intervention within 2 weeks prior to V3.

This visit will include:

- Update contact information (address, e mail, home and cell phone number) for patient, patient's family and patient's GP/cardiologist
- Assessment of inclusion and exclusion Criteria
- Record of concomitant medication
- Body weight measurement
- Physical examination including vital signs: SBP and DBP, heart rate
- Collection of adverse events
- Check of the compliance and stability of the dose of the LMT (atorvastatin, rosuvastatin, ± other LMT)
- Informed consent form proposal for the optional genomic sub-study, and if patient agrees to participate then obtain written consent
  - If patient declines participation in the genomic sub-study then this has no consequences for participation in the study otherwise

If the patient is confirmed eligible, the Investigator will start the next study procedures:

- IVRS/IWRS contact for randomization and allocation of a 7-digit treatment kit number according to the randomization list. Investigators should never allocate a treatment kit number to a patient without contacting IVRS/IWRS
- 12-lead ECG
- Urinalysis (at selected sites), with urine sample sent to Central Lab

- Urine pregnancy test (females of childbearing potential only)
- Blood sample for:
  - Lipids: TC, LDL-C, HDL-C, TG, non-HDL-C, ApoB, ApoA-1, ratio ApoB/ApoA-1, and Lp (a)
  - Hematology: blood cell count including hematocrit, hemoglobin, red blood cell count, reticulocyte count, white blood cell count with differential count and platelets

    NOTE: *At selected sites with a longer transit time to Central Lab, a second hematology sample will be collected for local assessment of reticulocyte count*
  - Chemistry: glucose, sodium, potassium, chloride, bicarbonate, calcium, phosphorous, urea nitrogen, creatinine, uric acid, LDH, total protein, albumin, and γGT
  - Liver panel (ALT, AST, ALP, and total bilirubin)
  - HbA_{1c}
  - CPK
  - hs-CRP
  - Hepatitis C antibody test

    NOTE: *In case of positive hepatitis C antibody test, a blood sample will be collected at a subsequent visit and sent to Covance Central Lab for further confirmatory testing*
  - Library samples
  - Anti- alirocumab antibodies
  - Genomic specimen collection (for specifically consented patients only)

NOTE: *Collection of all blood and urine samples at V3 should be performed before first double-blind IMP injection.*

- EQ-5D patient questionnaire: to be completed by the patient on site and data will be reported onto the eCRF by site staff.
- Double-blind IMP kit dispensation as per treatment kit number provided by IVRS along with schedule reminder. The patient injection instruction manual and treatment administration package should be provided. The patient diary should be given and instructions on its completion should be reviewed.
- The first double-blind IMP injection will take place at the study site, but only after the collection of the fasting blood samples and after the assessment of all evaluations planned at that visit. Close supervision, feedback and further training to be provided for IMP administration. The patient should be stay in observation for at least 30 minutes after the injection.

  NOTE*: This first injection of double-blind IMP injection can serve as second training injection.*
- Provide ODYSSEY Outcomes card (or equivalent) to patient (and family) mentioning patient participation in the study and site contact information.

- Reminders:
  - An appointment will be given for the next study site visit
  - Remind patient to be in fasting conditions (ie overnight, at least 8 hours fast) for next study site visit, if applicable
  - Remind patient to bring 3 items at the next study visit:
    - the diary,
    - used and unused IMP kits,
    - the ODYSSEY Outcomes card (or equivalent) containing site contact information.
  - Remind patient (and family) that, in case patient is hospitalized, they should contact the site (Investigator, Research Coordinator) as soon as possible, and not wait until the next visit.

10.1.5.1.2 Visit 4 (Month 1, ± 7 days)

This visit will include:

- Update contact information (address, e mail, home and cell phone number) for patient, patient's family and patient's GP/cardiologist
- ODYSSEY Outcomes card (or equivalent) mentioning patient participation in the study and site contact information:
  - Verify that patient has the card; if unsure, provide new cards to patient (and family).
  - Verify that site contact information (Investigator, Study Coordinator) provided to the patient is correct (update as necessary).
- Record of concomitant medication
- Vital signs: SBP and DBP, heart rate
- Collection of information on suspected efficacy endpoints
- Collection of cardiovascular events of interest other than efficacy endpoints (peripheral arterial disease, venous thromboembolic events)
- Collection of adverse events
- Data collection on IMP administration and IMP compliance checked by review of diary

  NOTE: *In case patient forgets to bring back diary at the visit, site should come up with an alternative solution to obtain as soon as possible information contained in patient diary in order to be able to complete IMP information in eCRF in a timely manner.*
- Check of the compliance and stability of the dose of the LMT (atorvastatin, rosuvastatin, ± other LMT)

- Blood sample for:
  - Lipids: TC, LDL-C, HDL-C, TG, non-HDL-C
  - Liver panel (ALT, AST, ALP, and total bilirubin)
  - CPK
- The patient diary should be given and instructions on its completion should be reviewed.
- Reminders:
  - An appointment will be given for the next study site visit.
  - Remind patient to be in fasting conditions (ie overnight, at least 8 hours fast) for next study site visit, if applicable.
  - Remind patient to bring 3 items at the next study visit:
    - the diary,
    - used and unused IMP kits,
    - the ODYSSEY Outcomes card (or equivalent) containing site contact information.
  - Remind patient (and family) that, in case patient is hospitalized, they should contact the site (Investigator, Research Coordinator) as soon as possible, and not wait until the next visit.

10.1.5.1.3 Visit 5 (Month 2, ± 7 days)

This visit will include:

- Update contact information (address, e mail, home and cell phone number) for patient, patient's family and patient's GP/cardiologist
- ODYSSEY Outcomes card (or equivalent) mentioning patient participation in the study and site contact information:
  - Verify that patient has the card; if unsure, provide new cards to patient (and family).
  - Verify that site contact information (Investigator, Study Coordinator) provided to the patient is correct (update as necessary).
- Record of concomitant medication
- Vital signs: SBP and DBP, heart rate
- Collection of information on suspected efficacy endpoints
- Collection of cardiovascular events of interest other than efficacy endpoints (peripheral arterial disease, venous thromboembolic events)
- Collection of adverse events

- Data collection on IMP administration and IMP compliance check by review of diary and treatment kit accountability (used and unused kits)

  NOTE: *In case patient forgets to bring back diary at the visit, site should come up with an alternative solution to obtain as soon as possible information contained in patient diary in order to be able to complete IMP information in eCRF in a timely manner.*
- Check of the compliance and stability of the dose of the LMT (atorvastatin, rosuvastatin, ± other LMT)
- Blood sample for:
  - Lipids: TC, LDL-C, HDL-C, TG, non-HDL-C
  - Anti-alirocumab antibodies.
- EQ-5D patient questionnaire
- Double blind IMP kit dispensation as per treatment kit number provided by IVRS along with schedule reminder and the treatment administration package should be provided. The patient injection instruction manual and diary may be given, as needed
- Reminders:
  - An appointment will be given for the next study site visit
  - Remind patient to be in fasting conditions (ie overnight, at least 8 hours fast) for next study site visit, if applicable
  - Remind patient to bring 3 items at the next study visit:
    - the diary,
    - used and unused IMP kits,
    - the ODYSSEY Outcomes card (or equivalent) containing site contact information.
  - Remind patient (and family) that, in case patient is hospitalized, they should contact the site (Investigator, Research Coordinator) as soon as possible, and not wait until the next visit.

10.1.5.1.4 Visit 6 (Month 4, ± 14 days)

This visit will include:

- Update contact information (address, e mail, home and cell phone number) for patient, patient's family and patient's GP/cardiologist
- ODYSSEY Outcomes card (or equivalent) mentioning patient participation in the study and site contact information:
  - Verify that patient has the card; if unsure, provide new cards to patient (and family).
  - Verify that site contact information (Investigator, Study Coordinator) provided to the patient is correct (update as necessary).
- Record of concomitant medication
- Body weight measurement

- Vital signs: SBP and DBP, heart rate
- Collection of information on suspected efficacy endpoints
- Collection of cardiovascular events of interest other than efficacy endpoints (peripheral arterial disease, venous thromboembolic events)
- Collection of adverse events
- Data collection on IMP administration and IMP compliance check by review of diary and treatment kit accountability (used and unused kits)

  NOTE: *In case patient forgets to bring back diary at the visit, site should come up with an alternative solution to obtain as soon as possible information contained in patient diary in order to be able to complete IMP information in eCRF in a timely manner.*
- Check of the compliance and stability of the dose of the LMT (atorvastatin, rosuvastatin, ± other LMT)
- Urine pregnancy test (females of childbearing potential only)
- Blood sample for:
  - Lipids: TC, LDL-C, HDL-C, TG, non-HDL-C, ApoB, ApoA-1, ratio ApoB/ApoA-1, and Lp (a)
  - Liver panel (ALT, AST, ALP, and total bilirubin)
  - CPK
  - hs-CRP
  - Library samples
  - Anti- alirocumab antibodies
- EQ-5D patient questionnaire
- Double blind IMP kit dispensation as per treatment kit number provided by IVRS along with schedule reminder and the treatment administration package should be provided. The patient injection instruction manual and diary may be given, as needed
- Reminders:
  - An appointment will be given for the next study site visit
  - Remind patient to be in fasting conditions (ie, overnight, at least 8 hours fast) for next study site visit, if applicable
  - Remind patient to bring 3 items at the next study visit:
    - the diary,
    - used and unused IMP kits,
    - the ODYSSEY Outcomes card (or equivalent) containing site contact information.
  - Remind patient (and family) that, in case patient is hospitalized, they should contact the site (Investigator, Research Coordinator) as soon as possible, and not wait until the next visit.

10.1.5.1.5 Visit 8 (Month 8, ± 14 days)

This visit will include:

- Update contact information (address, e mail, home and cell phone number) for patient, patient's family and patient's GP/cardiologist
- ODYSSEY Outcomes card (or equivalent) mentioning patient participation in the study and site contact information:
  - Verify that patient has the card; if unsure, provide new cards to patient (and family).
  - Verify that site contact information (Investigator, Study Coordinator) provided to the patient is correct (update as necessary).
- Record of concomitant medication
- Body weight measurement
- Vital signs: SBP and DBP, heart rate
- Collection of information on suspected efficacy endpoints
- Collection of cardiovascular events of interest other than efficacy endpoints (peripheral arterial disease, venous thromboembolic events)
- Collection of adverse events
- Data collection on IMP administration and IMP compliance check by review of diary and treatment kit accountability (used and unused kits)

  NOTE: *In case patient forgets to bring back diary at the visit, site should come up with an alternative solution to obtain as soon as possible information contained in patient diary in order to be able to complete IMP information in eCRF in a timely manner.*
- Check of the compliance and stability of the dose of the LMT (atorvastatin, rosuvastatin, ± other LMT)
- Urine pregnancy test (females of child-bearing potential only)
- Blood sample for:
  - Lipids: TC, LDL-C, HDL-C, TG, non-HDL-C
- EQ-5D patient questionnaire
- Double blind IMP kit dispensation as per treatment kit number provided by IVRS along with schedule reminder and the treatment administration package should be provided. The patient injection instruction manual and diary may be given, as needed
- Reminders:
  - An appointment will be given for the next study site visit
  - Remind patient to be in fasting conditions (ie, overnight, at least 8 hours fast) for next study site visit, if applicable

- Remind patient to bring 3 items at the next study visit:
  - the diary,
  - used and unused IMP kits,
  - the ODYSSEY Outcomes card (or equivalent) containing site contact information.
- Remind patient (and family) that, in case patient is hospitalized, they should contact the site (Investigator, Research Coordinator) as soon as possible, and not wait until the next visit.

10.1.5.1.6 Visit 10 (Month 12, ± 14 days)

This visit will include:

- Update contact information (address, e mail, home and cell phone number) for patient, patient's family and patient's GP/cardiologist
- ODYSSEY Outcomes card (or equivalent) mentioning patient participation in the study and site contact information:
  - Verify that patient has the card; if unsure, provide new cards to patient (and family).
  - Verify that site contact information (Investigator, Study Coordinator) provided to the patient is correct (update as necessary).
- Record of concomitant medication
- Body weight measurement
- Physical examination including vital signs: SBP and DBP, heart rate
- Collection of information on suspected efficacy endpoints
- Collection of cardiovascular events of interest other than efficacy endpoints (peripheral arterial disease, venous thromboembolic events)
- Collection of adverse events
- Data collection on IMP administration and IMP compliance check by review of diary and treatment kit accountability (used and unused kits)

  NOTE: *In case patient forgets to bring back diary at the visit, site should come up with an alternative solution to obtain as soon as possible information contained in patient diary in order to be able to complete IMP information in eCRF in a timely manner.*
- Check of the compliance and stability of the dose of the LMT (atorvastatin, rosuvastatin, ± other LMT)
- Urinalysis (at selected sites), with urine sample sent to Central Lab
- Urine pregnancy test (females of childbearing potential only)

- Blood sample for:
  - Lipids: TC, LDL-C, HDL-C, TG, non-HDL-C, ApoB, ApoA-1, ratio ApoB/ApoA-1, and Lp (a)
  - Hematology: blood cell count including hematocrit, hemoglobin, red blood cell count, reticulocyte count, white blood cell count with differential count and platelets

    NOTE: *At selected sites with a longer transit time to Central Lab, a duplicate hematology sample will be collected for local assessment of reticulocyte count*
  - Chemistry: glucose, sodium, potassium, chloride, bicarbonate, calcium, phosphorous, urea nitrogen, creatinine, uric acid, LDH, total protein, albumin, and γGT
  - Liver panel (ALT, AST, ALP, and total bilirubin)
  - HbA_{1c}
  - CPK
  - hs-CRP
  - Library samples
  - Anti- alirocumab antibodies
- EQ-5D patient questionnaire
- Double-blind IMP kit dispensation as per treatment kit number provided by IVRS along with schedule reminder. The patient injection instruction manual and treatment administration package should be provided. The patient diary should be given and instructions on its completion should be reviewed
- Reminders:
  - An appointment will be given for the next study site visit
  - Remind patient to be in fasting conditions (ie, overnight, at least 8 hours fast) for next study site visit, if applicable
  - Remind patient to bring 3 items at the next study visit:
    - the diary,
    - used and unused IMP kits,
    - the ODYSSEY Outcomes card (or equivalent) containing site contact information.
  - Remind patient (and family) that, in case patient is hospitalized, they should contact the site (Investigator, Research Coordinator) as soon as possible, and not wait until the next visit.

10.1.5.1.7 Visit 12 (Month 16)/ Visit 14 (Month 20)/ Visit 18 (Month 30)/ Visit 22 (Month 42)/ Visit 26 (Month 54) (± 14 days)

These visits will include:

- Update contact information (address, e mail, home and cell phone number) for patient, patient's family and patient's GP/cardiologist
- ODYSSEY Outcomes card (or equivalent) mentioning patient participation in the study and site contact information:
  - Verify that patient has the card; if unsure, provide new cards to patient (and family).
  - Verify that site contact information (Investigator, Study Coordinator) provided to the patient is correct (update as necessary).
- Record of concomitant medication
- Body weight measurement
- Vital signs: SBP and DBP, heart rate
- Collection of information on suspected efficacy endpoints
- Collection of cardiovascular events of interest other than efficacy endpoints (peripheral arterial disease, venous thromboembolic events)
- Collection of adverse events
- Data collection on IMP administration and IMP compliance check by review of diary and treatment kit accountability (used and unused kits)

  NOTE: *In case patient forgets to bring back diary at the visit, site should come up with an alternative solution to obtain as soon as possible information contained in patient diary in order to be able to complete IMP information in eCRF in a timely manner.*
- Check of the compliance and stability of the dose of the LMT (atorvastatin, rosuvastatin, ± other LMT)
- Urine pregnancy test (females of childbearing potential only)
- Blood sample for:
  - Lipids: TC, LDL-C, HDL-C, TG, non-HDL-C
  - Liver panel (ALT, AST, ALP, and total bilirubin)
  - CPK
- EQ-5D patient questionnaire
- Double-blind IMP kit dispensation as per treatment kit number provided by IVRS along with schedule reminder. In case an extra kit for the patient is needed, it should be done by contacting the IVRS/IWRS. The patient injection instruction manual and treatment administration package should be provided. The patient diary should be given and instructions on its completion should be reviewed

- Reminders:
  - An appointment will be given for the next study site visit
  - Remind patient to be in fasting conditions (ie, overnight, at least 8 hours fast) for next study site visit, if applicable
  - Remind patient to bring 3 items at the next study visit:
    - the diary,
    - used and unused IMP kits,
    - the ODYSSEY Outcomes card (or equivalent) containing site contact information.
  - Remind patient (and family) that, in case patient is hospitalized, they should contact the site (Investigator, Research Coordinator) as soon as possible, and not wait until the next visit.

10.1.5.1.8 Visit 16 (Month 24)/ Visit 20 (Month 36)/ Visit 24 (Month 48)/ Visit 28 (Month 60) (± 14 days)

These visits will include:

- Update contact information (address, e mail, home and cell phone number) for patient, patient's family and patient's GP/cardiologist
- ODYSSEY Outcomes card (or equivalent) mentioning patient participation in the study and site contact information:
  - Verify that patient has the card; if unsure, provide new cards to patient (and family).
  - Verify that site contact information (Investigator, Study Coordinator) provided to the patient is correct (update as necessary).
- Record of concomitant medication
- Body weight measurement
- Physical examination including vital signs: SBP and DBP, heart rate
- Collection of information on suspected efficacy endpoints
- Collection of cardiovascular events of interest other than efficacy endpoints (peripheral arterial disease, venous thromboembolic events)
- Collection of adverse events
- Data collection on IMP administration and IMP compliance check by review of diary and treatment kit accountability (used and unused kits)

  NOTE: *In case patient forgets to bring back diary at the visit, site should come up with an alternative solution to obtain as soon as possible information contained in patient diary in order to be able to complete IMP information in eCRF in a timely manner.*

- Check of the compliance and stability of the dose of the LMT (atorvastatin, rosuvastatin, ± other LMT)
- Urinalysis (at selected sites), with urine sample sent to Central Lab
- Urine pregnancy test (females of childbearing potential only)
- Blood sample for:
  - Lipids: TC, LDL-C, HDL-C, TG, non-HDL-C (in addition, at Month 24 only, ApoB will be collected)
  - Hematology: blood cell count including hematocrit, hemoglobin, red blood cell count, reticulocyte count, white blood cell count with differential count and platelets

    NOTE: at selected sites with a longer transit time to Central Lab, a second hematology sample will be collected for local assessment of reticulocyte count
  - Chemistry: glucose, sodium, potassium, chloride, bicarbonate, calcium, phosphorous, urea nitrogen, creatinine, uric acid, LDH, total protein, albumin, and γGT
  - Liver panel (ALT, AST, ALP, and total bilirubin)
  - HbA_{1c}
  - CPK
  - Anti- alirocumab antibodies
- EQ-5D patient questionnaire
- Double-blind IMP kit dispensation as per treatment kit number provided by IVRS along with schedule reminder. In case an extra kit for the patient is needed, it should be done by contacting the IVRS/IWRS. The patient injection instruction manual and treatment administration package should be provided. The patient diary should be given and instructions on its completion should be reviewed
- Reminders:
  - An appointment will be given for the next study site visit
  - Remind patient to be in fasting conditions (ie, overnight, at least 8 hours fast) for next study site visit, if applicable
  - Remind patient to bring 3 items at the next study visit:
    - the diary,
    - used and unused IMP kits,
    - the ODYSSEY Outcomes card (or equivalent) containing site contact information.
  - Remind patient (and family) that, in case patient is hospitalized, they should contact the site (Investigator, Research Coordinator) as soon as possible, and not wait until the next visit.

10.1.5.2 Contacts (phone calls, text messages or contacts via internet) from randomization

Contacts should be scheduled in between on-sites visits as follows: Visit 7 (Month 6)/ Visit 9 (Month 10)/ Visit 11 (Month 14)/ Visit 13 (Month 18)/ Visit 15 (Month 22)/ Visit 17 (Month 27)/ Visit 19 (Month 33)/ Visit 21 (Month 39)/ Visit 23 (Month 45)/ Visit 25 (Month 51)/ Visit 27 (Month 57)/ Visit 29 (Month 62).

Although flexibility is allowed in the timing of phone call in between the on-site/clinic visits, it should be scheduled with the goal of maintaining a contact with the patient about every 2 months during first 24 months, and about every 3 months thereafter. Having no phone or email contact with a patient for a prolonged period (>4 months) should be avoided as much as possible. Some additional instructions for scheduling phone call may be provided at time of interim analysis.

This contact will include:

- Update contact information (address, e mail, home and cell phone number) for patient, patient's family and patient's GP/cardiologist
- Review compliance with background LMT (including statin, ezetimibe)
- Collection of information on IMP administration (and remind patient to complete diary)
- Collection of information on suspected efficacy endpoints (and remind patient to call site in case of hospitalization and not wait until next visit)
- Reminders: as applicable for IMP administration schedule, timing of next visit, fasting conditions for next lab assessment, to bring the diary and used and unused kits at the next study site visit

In addition to the contacts detailed above, a SMS/text messaging vendor (EXCO InTouch) has been engaged to send appointment and injection reminders to any patient who wishes to and is able to receive them. This service is optional and each study site will have the opportunity to participate or refuse as per their policies.

These contacts will conform to privacy regulations at each site.

10.1.5.3 Early end of treatment visit – Visit 70

For patients who will have prematurely permanently discontinued IMP (see Section 10.3.2 for more details on when to consider that a patient has permanently discontinued treatment), an end of treatment visit called Visit 70 (early end of treatment visit) will be performed as soon as practically possible (within 1 month) after IMP discontinuation is considered permanent by the Investigator. Assessments done at this visit will be similar to those planned for the study completers at the final visit (Visit 30, Month 64) (see Section 1.2).

In addition:

- Update contact information (address, email, home and cell phone number) for patient, patient's family, and patient's GP/cardiologist
- ODYSSEY Outcomes card (or equivalent) mentioning patient participation in the study and site contact information:
  - Verify that patient has the card; if unsure, provide new cards to patient (and family).
  - Verify that site contact information (Investigator, Study Coordinator) provided to the patient is correct (update as necessary).

Then those patients must continue to remain in the study and will be strongly encouraged to complete all the remaining study visits as originally scheduled (as described in Section 1.2), until the common study end date (ie, final visit, Visit 30, Month 64). Complete usual study assessments (with the exception of IMP administration and its associated procedures) will be performed for 6 months following V70. Thereafter, study assessments are reduced (see Section 10.3.4 for more details). Patients who will not be able to attend any particular study visit will be invited to attend a subsequent visit.

Finally, the Investigator will make every effort to contact participants who are lost to follow-up. Attempts to contact such participants must be documented in the participant's records.

10.1.5.4 Common study end date visit (Final Visit) – Visit 30 (~Month 64, between CSED and CSED + 30 days)

This final CSED visit should be scheduled and be performed on or shortly after the actual occurrence of the CSED (see also Section 6.2.2). CSED is scheduled to occur at around Month 64, and will be announced by the sponsor in advance in order for sites to be able to plan this final visit.

This final CSED visit (Visit 30, Month 64):

- should be a clinic visit,
- should be performed for all randomized patients regardless of the patient status (still receiving IMP or having prematurely discontinued IMP permanently),
- however there are some differences on how to plan the visit (and last IMP injection, when applicable) and in assessments to be performed at that final visit, depending on patient status.

<u>For patients still receiving IMP (study completers)</u>, both CSED visit and last IMP injection should be determined taking into account the following:

- CSED visit should take place between CSED and CSED + 30 days
- CSED visit should occur 14 days (±3 days) after last IMP injection
- Site has clearly communicated and agreed in advance with patient (and family) on date of last IMP injection and date of CSED visit

- In order to ensure compliance with these critical final study procedures, sites should:
    - contact patients a few days prior to the agreed-upon date of last IMP injection,
    - remind patient about the final IMP injection and final CSED visit, and
    - ask patient to return to the investigational site in the morning preferably in fasting condition (ie, overnight, at least 8 hours fast) and to bring the used and unused kits and the diary.

Assessments to be performed at the CSED visit for study completers are described in Section 10.1.5.4.1.

<u>For patients who had previously permanently discontinued IMP:</u>

- CSED visit should take place between CSED and CSED + 30 days.
- Site has communicated and agreed in advance with patient (and family) on date of CSED visit.
- In order to ensure compliance with these critical final study procedures, sites should:
    - contact patients a few days prior to the final CSED visit, and
    - ask patient to return to the investigational site in the morning preferably in fasting condition (ie, overnight, at least 8 hours fast).
    - If IMP permanent discontinuation occurred after the prior most recent clinic visit and/or if patient still has IMP kits at home, patient should bring the used and unused kits and the diary.

Assessments to be performed at the CSED visit for patients who had permanently discontinued IMP are described in Section 10.1.5.4.2.

10.1.5.4.1 Final Visit - Visit 30 for study completers

This visit will include:

- Record of concomitant medication
- Body weight measurement
- Physical examination including vital signs: SBP and DBP, heart rate
- Collect information on suspected efficacy endpoints
- Collection of cardiovascular events of interest other than efficacy endpoints (peripheral arterial disease, venous thromboembolic events)
- Collection of adverse events

- Data collection on IMP administration and IMP compliance check by review of diary and treatment kit accountability (used and unused kits)

  NOTE: *In case patient forgets to bring back diary at the visit, site should come up with an alternative solution to obtain as soon as possible information contained in patient diary in order to be able to complete final IMP information in eCRF.*
- Check of the compliance and stability of the dose of the LMT (atorvastatin, rosuvastatin, ± other LMT)
- 12-lead ECG
- Urinalysis (at selected sites), with urine sample sent to Central Lab
- Urine pregnancy test (females of childbearing potential only)
- Blood sample for:
  - Lipids: TC, LDL-C, HDL-C, TG, non-HDL-C, ApoB, ApoA-1, ratio ApoB/ApoA-1, and Lp (a)
  - Hematology: blood cell count including hematocrit, hemoglobin, red blood cell count, reticulocyte count, white blood cell count with differential count and platelets

    NOTE: *At selected sites with a longer transit time to Central Lab, a second hematology sample will be collected for local assessment of reticulocyte count*
  - Chemistry: glucose, sodium, potassium, chloride, bicarbonate, calcium, phosphorous, urea nitrogen, creatinine, uric acid, LDH, total protein, albumin, and γGT
  - Liver panel (ALT, AST, ALP, and total bilirubin)
  - HbA_{1c}
  - CPK
  - hs-CRP
  - Hepatitis C antibody test

    NOTE: *In case of positive hepatitis C antibody test at V30, patient should be recontacted as soon as possible after the site has been informed by Central Lab of this positive test, and a subsequent visit should be scheduled also as soon as possible, for collection of a confirmatory blood sample to be sent to Covance Central Lab.*
  - Library samples
  - Anti-alirocumab antibodies
- EQ-5D patient questionnaire
- IVRS/IWRS contact for notification of the date of this final visit; for patients still on treatment this will also be the end of treatment visit

This will be the final study visit for the patient. New or ongoing related or serious AE (as well as new or ongoing AESI) at this CSED visit should continue to be followed until resolution, stabilization, or death (whichever comes first) and related data will be collected (see Section 6.2.1).

10.1.5.4.2 Final Visit - Visit 30 for prematurely discontinued patients

This visit will include:

- Collection of information on suspected efficacy endpoints
- Collection of cardiovascular events of interest other than efficacy endpoints (peripheral arterial disease, venous thromboembolic events)
- Collection of related and/or serious AEs
- Selected concomitant medications (statin, ezetimibe)
- Blood sample for
  - Lipids: TC, LDL-C, HDL-C, TG, non-HDL-C, ApoB
  - HbA1c
  - Anti-alirocumab antibodies
- IVRS/IWRS contact for notification of the date of this final visit

10.2 DEFINITION OF SOURCE DATA

Evaluations that are reported in the eCRF must be supported by appropriately signed identified source documentation related but not limited to the following:

- Agreement, date, and signature of informed consent mentioning the study identification
- Patient identification, last participation in a clinical trial, medical history, associated diseases, and data related to the studied pathology
- Contraception methods for women of childbearing potential
- Previous and concomitant medication (including the lipid modifying therapy)
- Study identification
- Treatment number, dates of administration
- Dates of visits and assessments including the examination report
- Vital signs, height, body weigh.
- Faxed central lab reports (dated and signed by the Principal Investigator or Sub-Investigator)
- IVRS/IWRS confirmation fax (screening, screen failure, training kit allocation, randomization, treatment reallocation, discontinuation, end of double blind treatment period, end of study, unblinding if applicable)
- ECG records signed and dated

- Adverse events and follow-up
  - In case of SAE, the site should file in the source document at least copies of the hospitalization reports and any relevant examination reports documenting the follow up of the SAE
- Date of premature study discontinuation (if any) and reason

Source documentation may be found in the following:

- Patient's identity
- Medical history
- Hospital records
- Nursing notes
- Physician's notes

10.3 HANDLING OF PATIENT TEMPORARY OR PERMANENT TREATMENT DISCONTINUATION AND OF PATIENT STUDY DISCONTINUATION

All randomized patients should be treated with blinded study treatment (IMP) for as long as possible.

As a general rule, any IMP treatment discontinuation should be initially considered temporary, and Investigator should make best effort to resume IMP treatment as early as practically possible, after several weeks or months (pending there are no safety concerns), and perform all study visits and assessments as usual.

Permanent IMP discontinuation should be a last resort. Any IMP discontinuation should be fully documented in the eCRF and source notes.

Pregnancy will lead to permanent treatment discontinuation in all cases.

All randomized patients should be followed-up (and suspected efficacy endpoints collected) until the end of the study (common study end date visit), even if treatment was permanently discontinued early.

10.3.1 Temporary treatment discontinuation with investigational medicinal product(s)

Temporary treatment discontinuation (also referred to as treatment interruption) may be considered by the Investigator in case of AEs/SAEs or for other reasons. In general, every effort should be made to resume treatment with blinded study drug (IMP) following a temporary interruption. In case of AEs/SAEs, resuming treatment with the IMP will be done under close and appropriate clinical and/or laboratory monitoring once the Investigator has considered according to his/her best medical judgment that the responsibility of the IMP in the occurrence of the concerned event was unlikely and patient does not meet any permanent discontinuation criteria.

(electronic 1.0)

All treatment interruption duration should be recorded by the Investigator in the appropriate eCRF screens when considered as confirmed.

Treatment interruption is defined as one or more scheduled injections that are not administered to the patient, and typically as decided by the Investigator.

10.3.2 Permanent treatment discontinuation with investigational medicinal product(s)

Permanent treatment discontinuation (also referred to as treatment discontinuation) is any treatment discontinuation associated with the definitive decision from the Investigator or the patient not to re-expose the patient to the IMP at any time.

Investigator is strongly encouraged to discuss with monitoring team before considering any treatment discontinuation as permanent. A discussion with National Coordinator may occur regarding other possible options.

10.3.3 List of criteria for permanent treatment discontinuation

The patients may withdraw from treatment with the IMP if they decide to do so, at any time and irrespective of the reason, or this may be the Investigator's decision. All efforts should be made to document the reasons for treatment discontinuation and this should be documented in the eCRF.

Patients should discontinue the Investigational Medicinal Product (IMP) for the following reasons:

- Pregnancy, intention for pregnancy, or no longer with effective contraceptive method of birth control (females only)
- Acute injection reaction of clinical concern
- Serious adverse event (or non-serious but severe in intensity) of hypersensitivity reaction considered related to IMP
- At patient request
- If, in the Investigator's opinion, continuation with the administration of the IMP would be detrimental to the patient's safety or well-being (NOTE: please refer to Section 10.4.7, Appendix C and Appendix D, for management and follow-up of selected laboratory abnormalities, including guidance for treatment discontinuation)
- At the specific request of the Sponsor
- Any code breaking requested by the Investigator
- Patient receives double-blind treatment prior to randomization

Patient withdrawal from the study treatment or study should be avoided as much as possible. If this occurs, every attempt should be made to restart study drug if medically appropriate, whatever the duration of discontinuation. In case of permanent study treatment discontinuation, the appropriate follow-up until the common study end date visit should still be continued.

All efforts should be made to document the reasons for treatment discontinuation and this should be documented in the eCRF.

10.3.4 Handling of patients after permanent treatment discontinuation

Patients in whom IMP treatment discontinuation is considered permanent by Investigator should come for an extra visit (early end-of-treatment visit V70) as soon as practically possible (within 1 month) after IMP discontinuation is considered permanent by Investigator. V70 is similar to the common end of study visit (Visit 30, Month 64) for study treatment completers, and should include similar procedures.

Such patients must continue to remain in the study and will be strongly encouraged to complete all the remaining study visits as originally scheduled in Section 1.2, until the common study end-date visit. Therefore, a contact should be maintained with these patients every 2 to 3 months (as per study assessments), and a clinic visit performed every 4 to 6 months until the common study end-date visit (V30).

- Over the 6 months following V70, all assessments (other than IMP administration and its associated procedures) should be performed as originally planned. These include but are not limited to collection of suspected efficacy endpoints, AE/AESI/SAE, concomitant medications, hematology and chemistry labs, lipid labs and anti- alirocumab antibodies.
- From 6 months post V70 until common study end date visit (V30), assessment at visits are simplified and will include updating patient contact information (as well as patients' family and patient's GP/cardiologist) and the collection of:
  - suspected efficacy endpoints
  - cardiovascular events of interest other than efficacy endpoints (peripheral arterial disease and venous thromboembolic events)
  - related and/or serious AEs
  - selected concomitant medications (statins, ezetimibe)
  - selected labs:
    - anti alirocumab antibodies, lipid panel and HbA1c: annually (at M12, M24, M36, M48, M60) and at common study end-date
    - ApoB at month 24 and at common study end-date.

  Previously observed AEs/AESIs/SAEs that had not resolved should be followed-up until an outcome (e.g. resolution, stabilization) has been determined.
- In case of difficulty to comply with the 4 to 6-month clinic visit periodicity in patients who discontinued treatment early, every effort should be made to have an actual clinic visit at least annually (with periodic phone/internet contacts every 3 months in between) and a final visit V30. During that annual visit and at V30, selected blood samples as described above should be collected.

In rare cases of written withdrawal of consent (WOC) for follow-up visits (i.e. patient does not wish to come back even for an annual visit), and unless otherwise stated by the patient in the informed consent form, Investigators will be encouraged to get information from the general practitioner, any other physician, or other medical-care provider, in order to follow the medical status of the patients (especially when they withdraw their consent after having experienced an AE/SAE or a cardiovascular event [efficacy endpoint]). Investigators will also be expected to try as much as possible to re-contact those patients at the end of the trial, in order to obtain at least

their vital status (dead or alive), as well as their cardiovascular status if possible, and thus avoid lost to follow-up for the efficacy assessment.

If the patient exercises his right of opposition to transmission of the data to the sponsor or removal of data from the database, the investigator will inform, in writing, the clinical trial sponsor, and the sponsor will decide how to handle the subject data and samples based on local regulations and data privacy requirements.

All definitive discontinuation of study treatment should be recorded by the Investigator in the appropriate screens of the eCRF and in the patient's medical records when considered as confirmed. IVRS/IWRS should be notified when a patient prematurely discontinues study treatment.

10.3.5 Procedure and consequence for patient withdrawal from study

The patients may withdraw from the study, before study completion if they decide to do so, at any time and irrespective of the reason or this may be the Investigator's decision:

- All study withdrawals should be recorded by the Investigator in the appropriate screens of the eCRF and in the patient's medical records when considered as confirmed (at least date of withdrawal and reason for). IVRS/IWRS should be notified when a patient prematurely discontinues study
- The patients should be assessed using the procedure normally planned for the visit V70 which corresponds to the early end of treatment visit. (see Section 10.3.4)

However, all randomized patients should be followed-up (and suspected efficacy endpoints as well as related and/or serious AEs collected) until the end of the study (common study end date visit), even if treatment was permanently discontinued early.

In case of study treatment discontinuation (temporary or permanent) due to an adverse event, such patients will be closely monitored until the resolution or stabilization of this adverse event. This may mean that follow-up will continue after the patients have completed the study.

For patients who fail to return to the site, the Investigator should make the best effort to re-contact the patient (eg, contacting patient's family or private physician, review available registries or health care database), and to determine his/her health status, including at least his/her vital status dead or alive at minimum, preferably also stroke or MI status). Attempts to contact such patients must be documented in the patient's records (e.g., times and dates of attempted telephone contact, receipt for sending a registered letter). This will be clearly stated in the informed consent form.

For patients considered lost to follow-up, the eCRF must be completed up to the last visit performed. The statistical analysis plan will provide the details concerning the analysis of these patients.

Patients who have withdrawn from the study cannot be re-randomized (treated) in the study. Their inclusion and treatment number must not be reused.

10.4 OBLIGATION OF THE INVESTIGATOR REGARDING SAFETY REPORTING

10.4.1 Definitions of adverse events

Please refer to Appendix E for Adverse Event (AE) reporting requirements.

10.4.1.1 Adverse event

An **adverse event** (AE) is any untoward medical occurrence in a patient or clinical investigation patient administered a pharmaceutical product and which does not necessarily have to have a causal relationship with this treatment.

10.4.1.2 Serious adverse event

A **serious adverse event** (SAE) is any untoward medical occurrence that at any dose:

- Results in death, or
- Is life-threatening, or
  Note: The term "life-threatening" in the definition of "serious" refers to an event in which the patient was at risk of death at the time of the event; it does not refer to an event which hypothetically might have caused death if it were more severe
- Requires inpatient hospitalization or prolongation of existing hospitalization, or
- Results in persistent or significant disability/incapacity, or
- Is a congenital anomaly/birth defect
- Is a medically important event
  Medical and scientific judgment should be exercised in deciding whether expedited reporting is appropriate in other situations, such as important medical events that may not be immediately life-threatening or result in death or hospitalization but may jeopardize the patient or may require intervention (ie, specific measures or corrective treatment) to prevent one of the other outcomes listed in the definition above

  NOTE: The following list of medically important events is intended to serve as a guideline for determining which condition has to be considered as a medically important event. The list is not intended to be exhaustive:
  - Intensive treatment in an emergency room or at home for:
    - Allergic bronchospasm
    - Blood dyscrasias (ie, agranulocytosis, aplastic anemia, bone marrow aplasia, myelodysplasia, pancytopenia, etc)
    - Convulsions (seizures, epilepsy, epileptic fit, absence, etc)
  - Development of drug dependency or drug abuse
  - ALT >3 x ULN + total bilirubin >2 x ULN or asymptomatic ALT increase >10 x ULN
  - Suicide attempt or any event suggestive of suicidality

- Syncope, loss of consciousness (except if documented as a consequence of blood sampling)
- Bullous cutaneous eruptions
- Cancers diagnosed during the study or aggravated during the study
- Chronic neurodegenerative diseases (newly diagnosed) or aggravated during the study

10.4.2 Adverse event of special interest

Adverse Events of Special Interest (AESI) are AEs (serious or non-serious) that need to be monitored, documented, and managed in a pre-specified manner described in the protocol. Please see Section 10.4.6.2 and Appendix E for additional information.

10.4.3 Serious adverse events waived from expedited regulatory reporting to health authorities

Unlike most studies where the primary efficacy variable is the resolution or improvement of an existing condition, in this study efficacy outcomes include the occurrence of life-threatening events. Indeed, participants to this study are recruited precisely because they are at high risk for these life threatening events. They are therefore expected to have at least one cardiovascular efficacy endpoint during the course of the study.

In light of the above, (primary and secondary) suspected cardiovascular efficacy endpoints as specified in this protocol will not be considered as AEs and are waived from regulatory reporting to Health Authorities except if the Investigator according to his/her best medical judgment considers these events as unexpected in the context of the underlying disease condition. In that case, the Investigator will complete an AE/SAE form (in addition to eCRF efficacy endpoint page – see below) including causality assessment within 24 hours.

For this study, all suspected cardiovascular efficacy endpoints will be reported (within 24 hours) in the specific eCRF efficacy endpoint pages; the system will automatically send the notification to the Clinical Event Committee Coordination Center at DCRI. This automatic notification will occur after the Investigator has approved the eCRF screens or after a standard time interval has elapsed, whichever comes first.

Expedited reporting to Health Authorities for the following cardiovascular outcomes will be waived:

- CV death including CHD death
- All other causes of death
- Non-fatal MI
- Non-fatal stroke
- Unstable angina requiring hospitalization
- Ischemia-driven coronary revascularization procedure
- Congestive heart failure requiring hospitalization

10.4.4 General guidelines for reporting adverse events

- AEs are to be recorded in corresponding screen(s) included in the eCRF.
- The reporting requirement of AEs depends on patient status (study completer or patient who had premature permanent discontinuation of IMP)
  - In patients who receive IMP until the end of the study (study completer), all AEs, regardless of seriousness or relationship to IMP/NIMP, spanning from the signature of the informed consent form until the CSED visit are to be recorded in the eCRF. Patients who experience a new or ongoing related or serious AE or a new or ongoing AESI (see Section 10.4.2) at the CSED visit should be followed beyond the CSED visit until resolution, stabilization, or death (whichever comes first) and related data will be collected (see Section 10.1.5.4.1).
  - In patients who have permanent premature IMP discontinuation:
    - All AEs, regardless of seriousness or relationship to IMP/NIMP, spanning from the signature of the informed consent form until 6 months following V70 (end of treatment visit) are to be recorded in the eCRF.
    - From 6 months following V70 until the CSED visit, related and/or serious AEs should be recorded in the eCRF.

  NOTE: *Reporting requirement for suspected efficacy endpoints is the same regardless of patient status (study completers or patients who had premature permanent discontinuation of IMP); suspected efficacy endpoinsts should be reported in all randomized patients until the end of the study (CSED).*
- Whenever possible, diagnosis or single syndrome should be reported instead of symptoms. The Investigator should specify the date of onset, intensity, action taken with respect to IMP, corrective treatment/therapy given, additional investigations performed, outcome, and his/her opinion as to whether there is a reasonable possibility that the AE was caused by the IMP.
- When treatment is prematurely discontinued, the patient will be maintained in the study and the patient's observations will continue until the common study end date visit (see Section 10.3.4).
- Laboratory, vital signs or ECG abnormalities are to be recorded as AEs only if:
  - Symptomatic and/or
  - Requiring either corrective treatment or consultation, and/or
  - Leading to IMP discontinuation or modification of dosing (with exception of LDL-C levels which may lead to IMP dosing modification, but in a blinded manner), and/or
  - Considered as clinically relevant (such as for ECG a prolonged QTcB > 500 ms or an increase in QTcB of > 60 msec compared to baseline), and/or
  - Fulfilling a seriousness criterion, and/or
  - Defined as an AE of special interest with immediate notification

See Appendix E for a summary of AE and efficacy endpoint reporting guidelines.

10.4.5 Instructions for reporting serious adverse events

In the case of occurrence of an SAE, the Investigator must immediately:

- ENTER (within 24 hours) the information related to the SAE in the appropriate screens of the eCRF; the system will automatically send a notification to a representative of the pharmacovigilance team after approval of the Investigator within the eCRF or after a standard time has elapsed, whichever comes first. Transmission to the pharmacovigilance team of information related to the SAE will be done either by fax or with an electronic solution.
- SEND (preferably by fax or e-mail) a photocopy of all examinations carried out and the dates on which these examinations were performed, to a representative of the pharmacovigilance team whose name, fax number, and email address will be provided for each region in a separate document. Care should be taken to ensure that the patient's identity is protected and the patient's identifiers in the clinical trial are properly mentioned on any copy of a source document provided to the Sponsor. For laboratory results, include the laboratory normal ranges.
- All further data updates should be recorded in the eCRF as appropriate, and further documentation as well as additional information (for laboratory data, concomitant medications, patient status, etc) should be sent (by fax or e-mail) to a representative of the pharmacovigilance team within 24 hours of knowledge of the SAE. In addition, every effort should be made to further document any SAE that is fatal or life threatening within a week (7 days) of the initial notification.
- A back-up plan (using a paper CRF process) is available and should be used when the eCRF system does not work.

10.4.6 Guidelines for reporting adverse events of special interest

10.4.6.1 Reporting of adverse events of special interest with immediate notification

For these AEs, the Sponsor will be informed immediately (ie within 24 hours), as per SAEs notification described in Section 10.4.5, even if not fulfilling a seriousness criterion, using the corresponding screens in the eCRF.

- ALT ≥3 ULN (if baseline ALT < ULN) Or ALT ≥2 times the baseline value (if baseline ALT ≥ ULN) (Please refer to related flowchart in Appendix C).
- Allergic events
  - Allergic drug reactions and/or local injection site reactions deemed to be allergic (or have an allergic component) that require consultation with another physician for further evaluation of hypersensitivity/allergy, as per the investigator's medical judgment or as per Section 10.6.2, should be reported as an AESI with immediate notification.
  - All allergic events, and all injection site reactions having an allergic component or deemed to be allergic, require completion of the specific eCRF screen (see Section 10.6.2), regardless of requirements for immediate reporting.

- Hemolytic anemia (see Section 10.4.7.1 and Appendix D)
  - If there is a decrease in hemoglobin and reflexive testing as per Appendix D suggesting hemolysis, then report this as an AESI with immediate notification. Special eCRF screen will need to be completed
- Pregnancy
  - *Pregnancy occurring in a female patient enrolled in the study* will be recorded as a pre-specified AE with immediate notification in all cases, and IMP should be discontinued in all cases. It will be qualified as an SAE only if it fulfills the SAE criteria. The follow-up of the pregnancy will be mandatory until the outcome of the pregnancy has been determined.
  - *Pregnancy occurring in the female partner of a male patient included in the clinical trial*: if permitted by the female partner and by local regulatory policies, it will be recorded as a pre-specified AE with immediate notification (SAE if it fulfills the SAE criteria), and pregnancy should be followed-up until the outcome has been determined.
- Symptomatic Overdose with IMP
  - An overdose (accidental or intentional) is an event suspected by the Investigator or spontaneously notified by the patient (not based on systematic injection counts) and defined as at least twice of the intended dose within the intended therapeutic interval (ie, 2 or more injections from the double-blind treatment kit are administered in <7 calendar days); to be reported using the corresponding screens in the eCRF using the Term "symptomatic OVERDOSE (accidental [or intentional])". The patient should be monitored and appropriate symptomatic treatment instituted
  - The circumstances of the overdose should be clearly specified in the verbatim
- Neurologic and Neurocognitive Events
  - Neurologic and Neurocognitive Events that require additional examinations/procedures and/or referral to a specialist should be reported as an AESI with immediate notification (see also Appendix E for reporting requirements).

10.4.6.2 Reporting of adverse events of special interest without immediate notification

See Appendix E.

For these AEs, the Sponsor does not have to be informed immediately, unless meeting seriousness criterion.

- Asymptomatic overdose with IMP
  - An overdose (accidental or intentional) is an event suspected by the Investigator or spontaneously notified by the patient (not based on systematic injection counts) and defined as at least twice of the intended dose within the intended therapeutic interval (ie, 2 or more injections from the double-blind treatment kit are administered in <7 calendar days); to be reported using the corresponding screens in the eCRF using

the Term "asymptomatic OVERDOSE (accidental [or intentional])" The patient should be monitored for any AEs and treated, as needed

- Local injection site reactions (see Section 10.6.1)
  - Local injection site reactions related to IMP that are considered as non-allergic events should be further characterized by evaluating the related symptoms that comprise an injection site reaction such as but not limited to redness, pain, etc (See Appendix F). Special eCRF screens will need to be completed. If such an AE was to occur, then do not report the individual components of the reaction but rather the term "local injection site reaction", the individual components being described in the specific eCRF screen
- Allergic events not referred for consultation with another physician (see Section 10.4.6.1)
  - All allergic events will need to have allergy specific eCRF screens completed (see Section 10.6.2), regardless of requirements for immediate reporting.
- Neurologic and Neurocognitive events
  - AEs related to neurologic or neurocognitive abnormalities with the exception of those requiring additional examinations/procedures and/or referral to a specialist (as mentioned in Section 10.4.6.1) should be reported in accordance with Appendix E.

10.4.7 Guidelines for management of specific laboratory abnormalities

Laboratory abnormalities with pre-specified monitoring should be monitored, documented, and managed according to the related flowchart in protocol Appendix C and Appendix D

- Neutropenia
- Thrombocytopenia
- Increase in ALT
- Acute renal insufficiency
- Decrease in hemoglobin (defined as ≥1.5 g/dL decrease from pre-randomization baseline value)
- Increase in CPK (other than cardiac-related) and suspicion of rhabdomyolysis.

Investigators are strongly encouraged to follow these algorithms in Appendix C and Appendix D, especially in situations where the abnormality persists or when there is no clear explanation for the observed abnormality.

However there may be situations where these algorithms are not entirely applicable, therefore Investigator may use his/her best judgment. Also, in some situations, the Sponsor may wish to discuss with the Investigator. Examples where these algorithms may not be applicable include (but are not limited to) the following situations:

- patients with known stable low or borderline neutrophil or platelet count or impaired renal function at baseline; Investigator should attempt to have a diagnosis for the observed finding and should use his/her best judgment whether or not to enroll these patients, and if

patient is enrolled, on how to best monitor these baseline abormalities throughout the study

- patients with ALT increase or elevated CK for which the abnormality resolves following statin dose reduction or statin discontinuation
- patients with elevated CK caused by a myocardial infarction

In addition, discontinuation caused by a laboratory abnormality can be either permanent or temporary, depending on the particular case. There is no requirement for permanent treatment discontinuation in every case of the general guidance for the follow up of selected laboratory abnormalities mentioned in Appendix C·

10.4.7.1 Hemoglobin decrease

See Appendix D.

At the first post-randomization study visit with occurrence of a hemoglobin (Hb) measurement decrease by ≥1.5 g/dL as compared to the randomization visit hemoglobin measurement, then the Central Lab will reflexively measure haptoglobin using specimens already obtained at the same time point for which the hemoglobin decrease was detected. The Central Lab will then provide the results of the reticulocyte count, haptoglobin, LDH and indirect bilirubin (reflexively measured only if the total bilirubin > ULN) to the Investigator.

<u>NOTE</u>: *At selected sites with a longer transit time to Central Lab where reticulocyte count cannot be measured by Central Lab, a second hematology sample will be collected for local assessment of reticulocyte count,*

- If the following pattern of abnormalities is noted:
  - Reticulocyte count > Central Lab (or if applicable, local lab) upper limit of the reference range (also referred to as ULN) **AND**
  - Haptoglobin < Central Lab's lower limit of the reference range (also referred to as LLN) **AND**
  - LDH > ULN **AND**
  - Indirect bilirubin > ULN (only if the total bilirubin > ULN)

  The patient should be referred to a hematologist. The hematologist should obtain a peripheral blood smear and anti-erythrocyte antibodies (direct and indirect) by Coombs test. Further investigations are at the discretion of the hematologist.
- If the results are normal or the pattern of abnormality is something other than that described above, then the Investigator should exercise his/her medical judgment in the interpretation of the results, necessity for workup of the decrease in hemoglobin or referral to a hematologist

If a second hemoglobin measurement demonstrating a further decrease of ≥1 g/dL from the last available value is observed, even if the previous work-up was negative, the same investigations can be repeated and a hematology consultation can be requested at the discretion of the Investigator or at the Sponsor's request.

10.5 OBLIGATIONS OF THE SPONSOR

During the course of the study, the Sponsor will report in an expedited manner all SAEs that are both unexpected and at least reasonably related to the IMP (Suspected Unexpected Serious Adverse Reaction[SUSAR]), to the Health Authorities, IECs/IRBs as appropriate and to the Investigators.

In addition, the Sponsor will report in an expedited manner all SAEs that are expected and at least reasonably related to the IMP to the Health Authorities, according to local regulations.

In this study, cardiovascular efficacy endpoints specified (primary and secondary efficacy endpoints) are waived from expedited reporting to Health Authorities providing an agreement has been reached with them.

Also some other AEs may be considered related to the underlying condition and thus will not be considered unexpected as given in the Investigator's Brochure.

Any other AE not listed as an expected event in the Investigator's Brochure or in this protocol will be considered as unexpected.

The Sponsor will report all safety observations made during the conduct of the trial in the clinical study report (CSR).

10.6 SAFETY INSTRUCTIONS

10.6.1 Local tolerability (Local Injection Site Reactions)

In case the Investigator or the patient recognizes any signs of local intolerability, then this should be treated and followed up as per the Investigator's medical judgment. See Section 10.4.6.2 and Appendix F for further information.

10.6.2 Allergic adverse events (See Section 10.4.6.1 and Section 10.4.6.2)

Specific eCRF screens are to be filled in to assess allergic reactions or allergic-like reactions that may occur during the clinical studies conducted with alirocumab.

Sometimes transient injection site reactions, irritant in nature, may occur, requiring no intervention and being of dubious significance. These reactions would not be considered to be allergic reactions.

Adverse events that may constitute an allergic reaction (eg, generalized itch, nasal itch, swelling at injection site, flushing, hives, swelling at lips, eyes, face, tongue, hands, feet, lump in throat, difficulty to swallow, hoarseness, change in pitch of voice, incapacity to speak, wheezing, chest tightness, stridor, etc) should be considered to be reported on the General Allergic Reaction and/or Local Injection Site Reaction Complementary Form.

Adverse events that are obviously not of allergic origin (eg, local injection site reactions related to IMP and with no allergic component) should only be recorded on the Local Injection Site

Reaction Complementary Form. However, injection site reactions which progress/expand/worsen/etc should be evaluated as recommended in Section 10.6.2.1 and General Allergic Reaction Complementary form should be completed.

The IMP should be immediately interrupted (temporarily discontinued) if there is a suspicion of an allergic event related to IMP. See Section 10.3.1 for further information on treatment interruption and Section 10.3.2 for criteria for permanent treatment discontinuation.

10.6.2.1 Allergic adverse event with cutaneous involvement

Adverse events with cutaneous involvement which are obviously of allergic origin or injection site reactions which progress/expand/worsen/etc should be evaluated by a dermatologist as soon as possible, and preferably within one week of the site first becoming aware of the event.

The investigator should evaluate the patient for possible etiologies (new medications, etc) and extra-cutaneous symptoms and signs. An unscheduled Central Laboratory assessment for hematology, chemistry, liver panel, and ADA should be obtained. If it is possible, the site will take pictures of the skin lesions in order to provide the patient with them for the dermatologist's visit. If the photos are obtained, then copies should be kept as source documents which may later be collected by the sponsor. The investigator will provide a summary of the patient's case, reason for consultation, and information being requested to the consulting dermatologist.

A full consultation report should be sent by the dermatologist to the investigator. The full report should contain, at a minimum, the following information; a detailed description of the rash (such as the morphology [lesion type], shape of individual lesions, arrangement of multiple lesions [eg, scattered, grouped, linear, etc], distribution, color, consistency, presence of pruritus or pain, and other clinical signs) and in case a skin biopsy (including histopathology and immunofluorescence) was done (if it was deemed necessary as per the dermatologist's or investigator's medical judgment), the results of this investigation with, if applicable, a specific diagnosis of the AE. The investigator will fax the full report and the corrected AE form if necessary, to the Monitoring Team Representative within 24 hours.

10.6.2.2 Acute allergic injection reactions (Section 10.4.6.2)

Acute allergic injection reaction (which are considered under the category of general allergic reactions) is defined as any AE that occurs during or shortly after injection of the IMP (characterized by but not limited to hypotension, bronchoconstriction, urticaria, edema, angioedema, nausea, vomiting). The investigator should ascertain that patient can be rapidly managed with emergency equipment and medication for the treatment of these potential adverse effects (eg, antihistamines, bronchodilators, IV saline, corticosteroids, acetaminophen, and epinephrine) for the injections at the training, and randomization visits.

Patients will be observed at the investigational site for at least 30 minutes following the injection that takes place at the randomization visit. Patients should be treated symptomatically if any AEs are observed. Patients are to remain on observation until any acute injection reaction is assessed as stable, per the Investigator's or emergency team's discretion. General Allergic Reaction and/or Local Injection Site Reaction Complementary Form will have to be completed.

10.6.3 Monitoring related to two consecutive LDL-C <25 mg/dL (0.65 mmol/L)

Patients who achieve 2 consecutive LDL-C levels <25 mg/dL (0.65 mmol/L) during the study will be monitored and managed as per Appendix A (any time after randomization) as the lower limit of safe and effective LDL-C lowering has not yet been established. An independent external physician(s) (also known as independent physician) will be notified by the central laboratory of 2consecutive LDL-C <25 mg/dL (0.65 mmol/L). The independent physician will review the unmasked LDL-C values and patient safety data, in close collaboration with the dedicated member of the Phase 3a DMC (implemented for all Phase 3 studies evaluating the efficacy and safety of alirocumab on LDL-C). This dedicated Phase 3a DMC member will then transmit any information as necessary to the common member involved in both DMCs (CV DMC and Phase 3a DMC).

Please see Appendix A for an outline of the process.

Then at subsequent visits, specific actions can be undertaken depending on the alirocumab dose (down-titration or discontinuation) as follows:

- On dose of 150 mg Q2W: if LDL-C <25 mg/dL (including LDL-C <15 mg/dL) on 2 consecutive values, down-titration to 75 mg Q2W will be done in a blinded manner at the next visit. The patient will remain at the down titrated dose of 75 mg Q2W for the remaining duration of the study, unless patient meets criteria below. As described above, specific monitoring consisting of review of patient level listing will be implemented.
- On dose of 75 mg Q2W:
  - If LDL-C <25 mg/dL on 2 consecutive values but there are no 2 consecutive measurements < 15 mg/dL (i.e. no measurement < 15 mg/dL or only one occasional measurement < 15 mg/dL): study treatment with 75 mg Q2W will be continued but additional monitoring with review of individual patient profiles will be implemented, to further confirm the safety of low LDL-C levels. A site alert related to 2 consecutive LDL-C < 25 mg/dL (0.65 mmol/L) may be requested by the independent physician (after consultation with dedicated phase 3a DMC member)
  - If measured LDL-C <15 mg/dL on 2 consecutive measurements: study treatment with 75 mg Q2W will be discontinued at the next visit. In order to maintain the blind there will be blinded substitution to placebo injections for the remaining duration of the study

As lipid values will not be communicated to investigators after randomization to maintain the blind, the continuation of the 75 mg dose, the up-titration to the 150 mg dose, the down-titration from 150 to 75 mg, or discontinuation will all occur automatically, without site or patient awareness. Moreover, blinded dose adjustment will avoid the need for dummy titrations in the placebo group.

10.7 ADVERSE EVENTS MONITORING

All events will be managed and reported in compliance with all applicable regulations, and included in the final clinical study report.

11 STATISTICAL CONSIDERATIONS

11.1 DETERMINATION OF SAMPLE SIZE

The sample size calculations are based on the primary efficacy variable, with the following assumptions:

- Primary efficacy endpoint is the time from randomization to the date of first occurrence of one of the following Clinical Events, as determined by the CEC: CHD death, any non-fatal MI, fatal and non-fatal ischemic stroke, unstable angina requiring hospitalization. Based on PROVE-IT results (8) and considering adjustment based on the study design and endpoints definition, the following Kaplan-Meier probabilities of event in the placebo group have been considered: 3.8% at 12 months, 6.4% at 24 months, 9.0% at 36 months, 11.4% at 48 months. Probability of event at different time points are estimated using a piece-wise exponential model. The primary efficacy endpoint will be analyzed on an intent-to-treat basis (all randomized patients, including those who discontinue study medication are followed for any efficacy event until the termination of the study)
- Hazard ratio of 0.85 in test group relative to placebo (corresponding to a 15% risk reduction). Constant hazard ratio assumption is used
- A log-rank test at a 1-sided 2.5% significance level with 90% power
- Two interim analyses, according to a group sequential design, using for efficacy Gamma (-22) α-spending function, and for futility a Gamma (-5) β-spending function. Non-binding spending functions are used. See Section 11.5 for interim analysis details
- 1% lost-to-follow rate at 24 months in both arms
- Enrolment rate: sample size of about 18,600 patients enrolled in 1400 sites (these sites should be active within 12 months). The table below describes the expected enrolment rate per month; these assumptions are based on internal experience to enroll this patient population

Table 3 - Enrolment rate assumptions per month

Month	1 to 3	4	5	6	7	8	9	10	11	12 to 40
**No. pts per month**	32	64	80	104	160	240	320	420	520	560
**Cumulative no. patients**	96	160	240	344	504	744	1064	1484	2004	2564 at M12, 18000 at M40

Based on the above assumptions, 1613 events are needed for 90% power. In order to achieve the 1613 targeted events, about 18000 patients (~9000 per group) will need to be randomized over a period of about 40 months. However, taking into account the situation in China (target of about 600 randomized patients in China based on local regulatory considerations, and local delayed

study start), the total number of patients randomized will be increased to approximately 18,600 patients (~9,300 per group). At the end of the study, the overall population will include about 18,000 randomized patients who have either died or been followed for a minimum of 24 months, supplemented with an additional subset of patients from China (~600) who may be followed for less than 24 months.

NOTE: *Continued enrolment of patients in China after the initial target of 18,000 randomized globally has been reached, and leading to an increase in sample size to about 18,600 patients, will be implemented only after appropriate local authorizations in China have been obtained.*

Calculations were made using East 5.4 software.

11.2 DISPOSITION OF PATIENTS

Screened patients are defined as any patient who met the qualifying ACS inclusion criteria and signed the informed consent.

Randomized patients consist of all screened patients, with a double-blind treatment kit number allocated and recorded in the IVRS/IWRS database, regardless of whether the treatment kit was used or not. Patients treated without being randomized or treated with a double-blind treatment kit before the randomization will not be considered as randomized and will not be included in any analysis population. The safety experience of these patients will be reported separately, and they will not be in the safety population.

For any patient randomized more than once, only the data associated with the first randomization will be used in any analysis population. The safety experience associated with any later randomization will be assessed separately.

11.3 ANALYSIS POPULATIONS

11.3.1 Efficacy populations

11.3.1.1 Intent–to-treat population

The primary efficacy analysis population will be the intent-to-treat (ITT) population, consisting of all randomized patients as defined above. Patients in the ITT population will be analyzed according to the treatment group allocated by randomization.

11.3.2 Safety population

The Safety population considered for safety analyses will be the randomized population who did actually receive at least one dose or part of a dose of the double-blind IMP. Patients will be analyzed according to the treatment actually received (placebo or alirocumab).

In addition:

- Randomized patients for whom it is unclear whether they took the study medication will be included in the safety population as randomized
- For patients receiving double-blind IMP from more than 1 treatment group during the trial, the treatment group allocation for as-treated analysis will be the one to which the patient was treated with the longest duration
- In case of two consecutive LDL-C values <15 mg/dL, placebo injections will be given to patients randomized in the alirocumab group in order to maintain the blind. Those placebo injections will not be considered as double-blind IMP

11.3.3 Other analysis populations

The anti- alirocumab antibody analysis will be performed on all treated patients (safety population) with a blood sample on D1 (baseline) and at least one evaluable blood sample for antibodies post double-blind IMP injection.

Analysis populations for pharmacogenomics will be defined in a specific Statistical Analysis Plan (SAP).

11.4 STATISTICAL METHODS

11.4.1 Extent of study treatment exposure and compliance

The extent of study treatment exposure and compliance will be assessed and summarized by actual treatment received within the safety population.

In order to ensure the continuity of the investigational treatment for the patients without interruption (only in case a disruption occurs in the availability of device components or during production of the auto injectors), back-up plans may be implemented as described in Section 8.1.

In that case, exposure to initial device and back-up device, if applicable, will be summarized and impact on study results will be assessed. More details will be provided in the SAP, if applicable.

Double-blind IMP injections are those administered from randomization to discontinuation of the study treatment, that is:

- Containing placebo for the ones administered to patients randomized in the placebo group
- Containing 75 or 150 mg of alirocumab

Placebo injections given to patients randomized to alirocumab following 2 consecutive LDL-C <15 mg/dL will not be considered as double-blind IMP in the statistical analyses.

11.4.1.1 Extent of investigational medicinal product exposure

The total exposure will be assessed by:

- Duration of IMP exposure in months defined as: (last dose of double-blind IMP injection date +14 – first dose of double-blind IMP injection date)/30.4375, regardless of unplanned intermittent discontinuations
- The total number of double-blind IMP injections by patient
- Duration of observation period (months), defined as: (last contact date – first dose date+1)/30.4375. Non-integer values will be rounded to one decimal place

The number (n) and percentage (%) of patients with an up-titration in the alirocumab group will be described.

The number (n) and percentage (%) of patients with an up-titration followed by a down-titration in the alirocumab group will be also described.

11.4.1.2 Compliance

Compliance will be assessed using the following parameters:

- The injection frequency will be defined for each patient as the average number of days between 2 injections, that is: (last dose date – first dose date)/(number of injections -1)
- The overall compliance will be defined for each patient as: 100-(% days with under-planned dosing + % days with above-planned dosing).Under-planned and above-planned dosing will be defined as follows, considering that injections should be performed every 2 weeks (+/- 3 days allowed time window):
  - The % days with under-planned dosing will be defined for each patient as the number of days with no injection administered within the previous 17 days divided by the duration of IMP injection exposure in days.
  - The % days with above-planned dosing will be defined for each patient as the number of days with more than one injection administered within the 11 days before/duration of IMP exposure in days.

These parameters will be summarized descriptively (N, Mean, standard deviation [SD], Median, Min and Max) at 6 months, by year, and on the overall study period.

Cases of overdose (ie, 2 or more injections from the double-blind treatment kit are administered in <7 calendar days) will be summarized by treatment group.

11.4.2 Analyses of efficacy endpoints

All efficacy analyses will be performed based on intent-to-treat (ITT) approach that included events occurring or assessments performed from randomization to the analysis cut-off date, even after the patient has discontinued the study treatment.

11.4.2.1 Analysis of primary efficacy endpoint(s)

The analysis of primary efficacy endpoint will be the comparison between the two treatments using the log-rank test procedure stratified by region (North America, South America, Western Europe, Eastern Europe, Asia, Other). As the number of events per individual country is expected to be low (about 50 countries), the analysis will be stratified according to a grouping of countries into regions.

This primary comparison is the test of the following hypotheses on the hazard ratio (HR), applying a one-sided nominal type I error of 0.0249 at the final analysis:

$$H_0\text{: HR} \geq 1 \text{ versus } H_1\text{: HR} < 1$$

The estimates of the HR and corresponding confidence interval at (1-2α) % level (α being the one-sided nominal significance level: α=0.249 at final analysis, α=0.0001 at second interim analysis) will be provided using a Cox Proportional Hazard Model stratified by region as for the log-rank test described above.

Consistency of the treatment effect across regions will be assessed.

Underlying assumptions of the Cox Proportional Hazard Model will be checked using graphical methods. If proportionality is not observed, some ad-hoc sensitivity analyses will be performed depending on the data (data-driven).

The survival curves will be estimated using Kaplan-Meier estimates: cumulative incidence of events at 6 months and by year, and appropriate confidence interval will be presented by treatment arm using Kaplan-Meier estimates. Kaplan-Meier curves will be displayed by treatment arm.

Two interim analyses will be performed See Section 11.5 for description of these analyses. The cut-off dates of final and interim analyses are expected to be:

- First interim analysis (futility): when 807 patients have been experienced at least one primary efficacy event (50% fraction information)
- Second interim analysis (futility and efficacy): when 1210 patients have been experienced at least one primary efficacy event (75% fraction information)
- Final analysis: when 1613 patients have experienced at least one primary efficacy event or 24 months after the last date of randomization ex-China, whichever comes last

Subgroup analyses

For the primary endpoint, the treatment effects across the following subgroup factors will be examined:

- Gender
- Age group (<65, ≥65)
- Race (Caucasian, Black, Asian/Oriental, and Other, as appropriate)

- Country (IVRS stratum, depending on the size of subgroups)
- Region (USA/Non-USA, and North America/South America/Eastern Europe/Western Europe/Asia/Rest of world)
- Time from ACS event to randomization (eg, 4-24 weeks, > 24 weeks)

For each parameter, a Cox proportional hazard model will be used for the overall population, including the parameter and the treatment by parameter interaction. In addition, Kaplan-Meier curves and summary statistics showing number of patients, number (%) of primary efficacy outcome events, cumulative incidence of events at 6 month and by year, and appropriate confidence interval may be provided for each treatment arm in previously selected subgroups defined by the baseline characteristic/prognostic factors.

In addition, the effect of the time from ACS event to randomization (weeks) will be assessed using a Cox proportional hazard model including the time from ACS event to randomization (continuous) as a covariate, the treatment group and the interaction.

11.4.2.2 Analyses of secondary efficacy endpoints

Method for controlling the overall Type-I error rate when testing the key secondary efficacy endpoints is described in Section 11.4.2.3.

Time to events secondary endpoints will be analyzed using the same statistical methodology as for the primary endpoint.

The percent change from baseline in calculated LDL-C at Month 4, at Month 24 and at the end of the study will be analyzed in the ITT population using an analysis of covariance (ANCOVA) model with treatment group and region as fixed effects, and the baseline calculated LDL-C as covariate.

Similar analyses will be performed for ApoB and non-HDL.

Based on previous experiences and published data on these endpoints, the assumptions of normality of the residuals, homogeneity of slopes and homoscedasticity underlying these models are usually valid.

Throughout the ANCOVA models, the alirocumab group will be compared to placebo using an appropriate contrast tested at the two-sided 0.05 level, and providing the 95% confidence interval (CI) of the difference.

For patients without calculated LDL-C, ApoB, or non-HDL-C value in the time window analyzed, the percent change from baseline will not be calculated.

11.4.2.3 Multiplicity consideration

In order to handle multiple main secondary endpoints, the overall Type-I error will be controlled by the use of a sequential inferential approach. Statistical significance of the primary parameter is

required before drawing inferential conclusions about first key secondary parameter (at the 0.0001 one-sided alpha level at the second interim analysis or at the 0.0249 one-sided alpha level at the final analysis). The order of tests is detailed in Section 9.1.3. Inferential conclusions about successive main secondary parameters require statistical significance of the prior one.

This fixed hierarchical approach will ensure a strong control of the overall type-I error rate at the required one-sided level (0.0001 for the second interim analysis and 0.0249 at the final analysis).

No further adjustments will be made for other secondary endpoints or subgroup analyses for which p-values will be provided for descriptive purpose only.

11.4.3 Analyses of safety data

The summary of safety results will be presented by treatment group. No formal inferential testing will be performed. Summaries will be descriptive in nature.

All safety analyses will be performed on the Safety population, using the last available value before first double-blind IMP injection as baseline definition.

The following definitions will be applied to laboratory parameters, and vital signs.

- The potentially clinically significant abnormality (PCSA) values are defined as abnormal values considered medically important by the Sponsor according to predefined criteria/thresholds based on literature review and defined by the Sponsor for clinical laboratory tests, and vital signs.
- PCSA criteria will determine which patients had at least 1 PCSA during the TEAE period, taking into account all evaluations performed during the TEAE period, including unscheduled or repeated evaluations. The number of all such patients will be the numerator for the PCSA percentage.

<u>AE definition:</u>

- Pre-treatment AEs are AEs that developed or worsened or became serious during the PRE-TREATMENT period;
- Treatment-emergent AEs (TEAEs) are AEs that developed or worsened or became serious during the TEAE period;
- Post-treatment AEs are AEs that developed or worsened or became serious during the POST-TREATMENT period.

Possible drug-induced liver injury

The liver function tests, namely ALT, AST, alkaline phosphatase and total bilirubin, are used to assess possible drug induced liver toxicity. The proportion of patients with PCSA values at any post-baseline visit by baseline status will be displayed by treatment group for each parameter.

A graph of distribution of peak values of ALT versus peak values of total bilirubin will also be presented. Note that the ALT and total bilirubin values are presented on a logarithmic scale. The

graph will be divided into 4 quadrants with a vertical line corresponding to 3 x ULN for ALT and a horizontal line corresponding to 2 x ULN for total bilirubin.

The incidence of liver-related AEs will be summarized by treatment group. The selection of preferred terms will be based on standardized MedDRA query (SMQ) Hepatic disorder. Time to liver-related treatment discontinuation and time to liver death may also be provided based on hepatic disorder SMQ.

11.4.3.1 Adverse events

Adverse event incidence tables will present by system organ class (SOC) (sorted by internationally agreed order), high-level group term (HLGT), high level term (HLT) and preferred term (PT) sorted in alphabetical order for each treatment group, the number (n) and percentage (%) of patients experiencing an AE. Multiple occurrences of the same event in the same patient will be counted only once in the tables within a treatment phase. The denominator for computation of percentages is the safety population within each treatment group.

Adverse event incidence table will be provided by treatment group for all types of TEAEs: all TEAEs, all treatment emergent AESI (defined with a PT or a prespecified grouping), all treatment emergent SAEs and all TEAEs leading to permanent treatment discontinuation. If any clinically significant signal is detected and need further characterization or for adverse event of clinical interest (eg, injection site reaction, allergic reaction), exploration of time to onset will be performed for these selected TEAEs as described below to account for the differential exposure time in all patients.

Local injection site reaction could be further described in terms of time pattern (time to first occurrence, duration, recurrence) and intensity.

Selected TEAEs will be also analyzed using time-to-event approach (Kaplan-Meier methodology). Time from the first dose of double-blind IMP injection to the first occurrence of the event will be calculated (only the first event will be counted). Patients without any event will be censored at the end of the TEAE period. Incidence rates at 6 months and by year of exposure will be presented and Kaplan-Meier curves will be provided.

LDL-C <25 mg/dL:

Summaries of adverse events will be also provided on the safety subgroup population of patients with two consecutive LDL-C <25mg/dL in the alirocumab group. Only adverse events, for which it will be confirmed or unclear that they occurred, worsened or became serious after the first level of LDL-C <25mg/dL will be considered.

Death:

The following deaths summaries will be generated:

- Number (%) of patients who died by study period (TEAE, on-study) and adjudicated reasons, summarized on the safety population by treatment received;
- Death in non randomized patients or randomized and not treated patients;
- TEAE leading to death (death as an outcome on the AE eCRF page as reported by the Investigator) by primary SOC , HLGT, HLT and PT showing number (%) of patients sorted by internationally agreed order of SOC and alphabetic order of HLGT, HLT, and PT.

11.4.3.2 Laboratory data and vital signs

The summary statistics (including mean, median, Q1, Q3, standard error, minimum and maximum) of all laboratory variables, all vital signs parameters (raw data and changes from baseline) will be calculated for each visit, last and worst value assessed during the treatment period and presented by treatment group. For selected parameters, mean changes from baseline with the corresponding standard error will be plotted over time (at same time points) in each treatment group.

The incidence of PCSAs at any time during the TEAE period will be summarized by treatment group whatever the baseline level and/or according to the following baseline categories:

- Normal/missing;
- Abnormal according to PCSA criterion or criteria.

For laboratory parameters for which PCSA criterion is not defined, similar table(s) using the normal range could be provided.

Hepatitis C antibody:

The number and percentage of patients with an observed seroconversion for Hepatitis C Test will be provided by treatment group.

11.4.4 Other endpoints:

11.4.4.1 Cardiovascular events of interest (other than efficacy endpoints)

Clinically significant complications or procedures (not planned at the time of randomization) related to peripheral arterial disease, and venous thromboembolic events (see Section 9.3.7) will be analyzed using a time-to-event approach (Kaplan-Meier methodology) in the ITT population. Time from randomization to the first occurrence of the event will be calculated. Patients without any event will be censored using the same methodology as for the primary efficacy endpoint.

11.4.4.2 Other endpoints

All analyses for other endpoints (not already described above) will be performed on the Safety population. The baseline value is defined as the last available value before first double-blind IMP injection.

The number and percentage of patients with calculated LDL-C <25 mg/dL (respectively, LDL-C <15 mg/dL) will be provided by treatment group and visit.

Exploratory variables defined in Section 9.3 will be summarized by time points using number of available data, mean, SD, median, minimum, and maximum for each treatment group (for hs-CRP, Q1 and Q3 will be also provided). The time profile of each parameter will be also plotted by treatment group with the corresponding standard errors. For hs-CRP, the incidence of PCSA at any time during the TEAE period will be also summarized by treatment group using descriptive statistics.

The anti-alirocumab antibody status (positive/negative) and antibody titers will be summarized by treatment group and visit using descriptive statistics. Anti- alirocumab antibody will be further described in terms of time-to-onset, persistence (transient/persistent anti- alirocumab antibodies). Correlations between antibody titers, safety and/or efficacy endpoints could be also provided by graphical methods. Further details will be provided in SAP.

11.5 INTERIM ANALYSIS

Two interim analyses (IA) are planned, when 50% and 75% of the total number of expected events have occurred:

- Interim analysis for futility will be conducted, when approximately 807 events (50% of the targeted number of primary endpoint events) have occurred.
- Interim analysis for futility and overwhelming efficacy will be conducted, when approximately 1210 events (75% of the targeted number of primary endpoint events) have occurred.

Both IA will be performed by the external independent CV DMC Statistician and will be reviewed under the supervision of the CV DMC. The CV DMC will also review secondary efficacy endpoints and safety data (AEs, laboratory data, vital signs) available at the time of the IA.

Control of the type I and type II error will be ensured using gamma (-5) spending function for Type II error (futility) and Gamma (-22) for Type I error (efficacy) at each IA (the Type I error spending function is also applied at the first IA, even if the objective of this first IA is only futility). It has to be noted that, in order to protect the global type one error in case the decision is taken to overrule the futility rule, non binding boundaries were used.

The following table shows the stopping rules at each interim analysis (using the sample size assumptions described in Section 11.1):

Table 4 - Interim Analyses stopping boundaries corresponding to Gamma (-22) type I error and Gamma (-5) type II error spending functions

Timing of analyses	Stopping boundaries (one-sided p-value and Hazard ratio)	
	**Futility**	**Overwhelming efficacy**
First IA : 50% of targeted events	p > 0.548<br>(⇔ HR > 1.008)	NA
Second IA: 75% of targeted events	p > 0.19<br>(⇔ HR > 0.951)	p < 0.0001*<br>(⇔ HR < 0.802)

Calculations done using EAST 5.4

*Should the second interim analysis be triggered just before or after 1210 events have been reached, the exact nominal significance level to be used at the second IA would be re-computed based on a Gamma(-22) spending function.

The CV DMC could consider early stopping of the study for overwhelming efficacy at the second IA, if the following conditions are met:

- Stopping boundaries for overwhelming efficacy are crossed
- Positive trend observed for secondary efficacy endpoints, including all cause mortality, and no excess of non-CV mortality
- Consistency of the treatment effect on the primary efficacy endpoint across subgroups and regions

12 ETHICAL AND REGULATORY STANDARDS

12.1 ETHICAL PRINCIPLES

This clinical trial will be conducted in accordance with the principles laid down by the 18th World Medical Assembly (Helsinki, 1964) and all applicable amendments laid down by the World Medical Assemblies, and the International Conference on Harmonization [ICH] guidelines for good clinical practice (GCP).

In compliance with Sanofi public disclosure commitments, this clinical trial will be recorded in the public registry website clinicaltrials.gov before the enrollment of the first patient. The registry will contain basic information about the trial sufficient to inform interested patients (and their healthcare practitioners) how to enroll in the trial.

12.2 LAWS AND REGULATIONS

This clinical trial will be conducted in compliance with all international guidelines, and national laws and regulations of the country(ies) in which the clinical trial is performed, as well as any applicable guidelines. Please see Section 13.1

12.3 INFORMED CONSENT

The Investigator (according to applicable regulatory requirements), or a person designated by the Investigator, and under the Investigator's responsibility, should fully inform the patient of all pertinent aspects of the clinical trial including the written information giving approval/favorable opinion by the Ethics Committee (IRB/IEC). All participants should be informed to the fullest extent possible about the study, in language and terms they are able to understand.

Prior to a patient's participation in the clinical trial, the written informed consent form should be signed, name filled in and personally dated by the patient or by the patient's legally acceptable representative, and by the person who conducted the informed consent discussion. A copy of the signed and dated written informed consent form will be provided to the patient.

The Informed Consent Form and the optional written Informed Consent Form for pharmacogenetics used by the Investigator for obtaining the patient's informed consent must be reviewed and approved by the Sponsor prior to submission to the appropriate Ethics Committee (IRB/IEC) for approval/favorable opinion.

Prior to collection of blood for pharmacogenetics, the optional pharmacogenetic informed consent form (written) should be signed, name filled in, and personally dated by the patient or by the subject's legally acceptable representative, and by the person who conducted the consent discussion. A copy of the signed and dated written optional informed consent form will be provided to the subject.

12.4 INSTITUTIONAL REVIEW BOARD/INDEPENDENT ETHICS COMMITTEE (IRB/IEC)

As required by local regulation, the Investigator or the Sponsor must submit this clinical trial protocol to the appropriate Ethics Committee (IRB/IEC), and is required to forward to the respective other party a copy of the written and dated approval/favorable opinion signed by the Chairman with Ethics Committee (IRB/IEC) composition.

The clinical trial (study number, clinical trial protocol title and version number), the documents reviewed (clinical trial protocol, informed consent form, Investigator's Brochure, Investigator's curriculum vitae, etc) and the date of the review should be clearly stated on the written (IRB/IEC) approval/favorable opinion.

IMP will not be released at the study site and the Investigator will not start the study before the written and dated approval/favorable opinion is received by the Investigator and the Sponsor.

During the clinical trial, any amendment or modification to the clinical trial protocol should be submitted to the Ethics Committee (IRB/IEC) before implementation, unless the change is necessary to eliminate an immediate hazard to the patients, in which case the IRB/IEC should be informed as soon as possible. It should also be informed of any event likely to affect the safety of patients or the continued conduct of the clinical trial, in particular any change in safety. All updates to the Investigator's Brochure will be sent to the Ethics Committee (IRB/IEC).

A progress report is sent to the Ethics Committee (IRB/IEC) at least annually and a summary of the clinical trial's outcome at the end of the clinical trial.

(electronic 1.0)

13 STUDY MONITORING

13.1 RESPONSIBILITIES OF THE INVESTIGATOR(S)

The Investigator(s) and delegated Investigator staff undertake(s) to perform the clinical trial in accordance with this clinical trial protocol, ICH guidelines for Good Clinical Practice and the applicable regulatory requirements.

The Investigator is required to ensure compliance with all procedures required by the clinical trial protocol and with all study procedures provided by the Sponsor (including security rules). The Investigator agrees to provide reliable data and all information requested by the clinical trial protocol (with the help of the eCRF, Discrepancy Resolution Form [DRF] or other appropriate instrument) in an accurate and legible manner according to the instructions provided and to ensure direct access to source documents by Sponsor representatives.

If any circuit includes transfer of data particular attention should be paid to the confidentiality of the patient's data to be transferred.

The Investigator may appoint such other individuals as he/she may deem appropriate as Subinvestigators to assist in the conduct of the clinical trial in accordance with the clinical trial protocol. All Subinvestigators shall be appointed and listed in a timely manner. The Subinvestigators will be supervised by and work under the responsibility of the Investigator. The Investigator will provide them with a copy of the clinical trial protocol and all necessary information.

13.2 RESPONSIBILITIES OF THE SPONSOR

The Sponsor of this clinical trial is responsible to health authorities for taking all reasonable steps to ensure the proper conduct of the clinical trial protocol as regards ethics, clinical trial protocol compliance, and integrity and validity of the data recorded on the e-s. Thus, the main duty of the monitoring team is to help the Investigator and the Sponsor maintain a high level of ethical, scientific, technical and regulatory quality in all aspects of the clinical trial.

At regular intervals during the clinical trial, the site will be contacted, through monitoring visits, letters or telephone calls, by a representative of the monitoring team to review study progress, Investigator and patient compliance with clinical trial protocol requirements and any emergent problems. These monitoring visits will include but not be limited to review of the following aspects: patient informed consent, patient recruitment and follow-up, SAE documentation and reporting, AESI documentation and reporting, AE documentation, IMP allocation, patient compliance with the IMP regimen, IMP accountability, concomitant therapy use and quality of data.

13.3 SOURCE DOCUMENT REQUIREMENTS

According to the ICH guidelines for Good Clinical Practice, and in accordance with the Sponsor's guidelines and policies for source document verification, the monitoring team will check the eCRF entries against the source documents. This does not include pre-identified source data directly recorded in the eCRF. The informed consent form will include a statement by which the patient allows the Sponsor's duly authorized personnel, the Ethics Committee (IRB/IEC), and the regulatory authorities to have direct access to original medical records which support the data on the eCRFs (eg, patient's medical file, appointment books, original laboratory records, etc). These personnel, bound by professional secrecy, must maintain the confidentiality of all personal identity or personal medical information (according to confidentiality and personal data protection rules).

13.4 USE AND COMPLETION OF CASE REPORT FORMS (eCRFS) AND ADDITIONAL REQUEST

It is the responsibility of the Investigator to maintain adequate and accurate eCRFs (according to the technology used) designed by the Sponsor to record (according to Sponsor instructions) all observations and other data pertinent to the clinical investigation in a timely manner. All eCRFs should be completed in their entirety in a neat, legible manner to ensure accurate interpretation of data.

Should a correction be made, the corrected information will be entered in the eCRF overwriting the initial information. An audit trail allows identifying the modification.

Data are available within the system to the sponsor as soon as they are entered in the eCRF.

The computerized handling of the data by the Sponsor when available in the eCRF may generate additional requests (DRF) to which the Investigator is obliged to respond by confirming or modifying the data questioned. The requests with their responses will be managed through the eCRF.

13.5 USE OF COMPUTERIZED SYSTEMS

Computerized systems used during the different steps of the study are:

- For data management activities, Medidata RAVE version 5.6.4 (Covance)
- For statistical activities, nQuery Advisor 6.01, SAS, EAST 5.4
- For pharmacovigilance activities, AWARE , Business Objects XI
- For monitoring activities, CTMS of each involved institution (DCRI, Covance, and Sanofi, as applicable).
- For medical writing activities, DOMASYS

External data loading is planned for this clinical trial.

(electronic 1.0)

14 ADMINISTRATIVE EXPECTATIONS

14.1 CURRICULUM VITAE

A current copy of the curriculum vitae describing the experience, qualification and training of each Investigator and Subinvestigator will be signed, dated and provided to the Sponsor prior to the beginning of the clinical trial.

14.2 RECORD RETENTION IN STUDY SITES

The Investigator must maintain confidential all study documentation, and take measures to prevent accidental or premature destruction of these documents.

The Investigator should retain the study documents at least 15 years after the completion or discontinuation of the clinical trial.

However, applicable regulatory requirements should be taken into account in the event that a longer period is required.

The Investigator must notify the Sponsor prior to destroying any study essential documents following the clinical trial completion or discontinuation.

If the Investigator's personal situation is such that archiving can no longer be ensured by him/her, the Investigator shall inform the Sponsor and the relevant records shall be transferred to a mutually agreed upon designee.

14.3 CONFIDENTIALITY

All information disclosed or provided by the Sponsor (or any company/institution acting on their behalf), or produced during the clinical trial, including, but not limited to, the clinical trial protocol, the CRFs, the Investigator's Brochure and the results obtained during the course of the clinical trial, is confidential, prior to the publication of results. The Investigator and any person under his/her authority agree to undertake to keep confidential and not to disclose the information to any third party without the prior written approval of the Sponsor.

However, the submission of this clinical trial protocol and other necessary documentation to the Ethics committee (IRB/IEC) is expressly permitted, the IRB/IEC members having the same obligation of confidentiality.

The Subinvestigators shall be bound by the same obligation as the Investigator. The Investigator shall inform the Subinvestigators of the confidential nature of the clinical trial.

The Investigator and the Subinvestigators shall use the information solely for the purposes of the clinical trial, to the exclusion of any use for their own or for a third party's account.

Furthermore, the Investigator and the Sponsor agree to adhere to the principles of personal data confidentiality in relation to the patients, Investigator and its collaborators involved in the study.

14.4 PROPERTY RIGHTS

All information, documents and IMP provided by the Sponsor or its designee are and remain the sole property of the Sponsor.

The Investigator shall not mention any information or the Product in any application for a patent or for any other intellectual property rights.

All the results, data, documents and inventions, which arise directly or indirectly from the clinical trial in any form, shall be the immediate and exclusive property of the Sponsor.

The Sponsor may use or exploit all the results at its own discretion, without any limitation to its property right (territory, field, continuance). The Sponsor shall be under no obligation to patent, develop, market or otherwise use the results of the clinical trial.

As the case may be, the Investigator and/or the Subinvestigators shall provide all assistance required by the Sponsor, at the Sponsor's expense, for obtaining and defending any patent, including signature of legal documents.

14.5 DATA PROTECTION

The patient's personal data, which are included in the Sponsor's database, shall be treated in compliance with all applicable laws and regulations.

When archiving or processing personal data pertaining to the Investigator and/or to the patients, the Sponsor shall take all appropriate measures to safeguard and prevent access to this data by any unauthorized third party.

The Sponsor also collects specific data regarding Investigator as well as personal data from any person involved in the study which may be included in the Sponsor's databases, shall be treated by both the Sponsor and the Investigator in compliance with all applicable laws and regulations.

14.6 INSURANCE COMPENSATION

The Sponsor certifies that it has taken out a liability insurance policy covering all clinical trials under its sponsorship. This insurance policy is in accordance with local laws and requirements. The insurance of the Sponsor does not relieve the Investigator and the collaborators from maintaining their own liability insurance policy. An insurance certificate will be provided to the Ethics Committees (IECs/IRBs) or health authorities in countries requiring this document.

(electronic 1.0)

14.7 SPONSOR AUDITS AND INSPECTIONS BY REGULATORY AGENCIES

For the purpose of ensuring compliance with the clinical trial protocol, Good Clinical Practice and applicable regulatory requirements, the Investigator should permit auditing by or on the behalf of the Sponsor and inspection by regulatory authorities.

The Investigator agrees to allow the auditors/inspectors to have direct access to his/her study records for review, being understood that these personnel is bound by professional secrecy, and as such will not disclose any personal identity or personal medical information.

The Investigator will make every effort to help with the performance of the audits and inspections, giving access to all necessary facilities, data, and documents.

As soon as the Investigator is notified of a planned inspection by the authorities, he will inform the Sponsor and authorize the Sponsor to participate in this inspection.

The confidentiality of the data verified and the protection of the patients should be respected during these inspections.

Any result and information arising from the inspections by the regulatory authorities will be immediately communicated by the Investigator to the Sponsor.

The Investigator shall take appropriate measures required by the Sponsor to take corrective actions for all problems found during the audit or inspections.

14.8 PREMATURE DISCONTINUATION OF THE STUDY OR PREMATURE CLOSE-OUT OF A SITE

14.8.1 Decided by the Sponsor

Decided by the Sponsor in the following cases

- If the information on the product leads to doubt as to the benefit/risk ratio
- If the Investigator has received from the Sponsor all IMP, means and information necessary to perform the clinical trial and has not included any patient after a reasonable period of time mutually agreed upon
- In the event of breach by the Investigator of a fundamental obligation under this agreement, including but not limited to breach of the clinical trial protocol, breach of the applicable laws and regulations or breach of the ICH guidelines on Good Clinical Practice
- If the total number of patients are included earlier than expected

In any case the Sponsor will notify the Investigator of its decision by written notice.

14.8.2 Decided by the Investigator

The Investigator must notify (30 days' prior notice) the Sponsor of his/her decision and give the reason in writing.

In all cases (decided by the Sponsor or by the Investigator), the appropriate Ethics Committee(s) (IRB/IEC) and Health Authorities should be informed according to applicable regulatory requirements.

14.9 CLINICAL TRIAL RESULTS

The Sponsor will be responsible for preparing a clinical study report and to provide a summary of study results to the Investigator.

14.10 PUBLICATIONS AND COMMUNICATIONS

During the course of the study, the Executive Steering Committee co-Chairs will form a Publications Subcommittee, which will include all ESC members, and Sponsor representatives and to which selected National Leaders may be invited. The role of the Publication Sub-Committee will be to oversee the publications from the Study, assign authorship, assure that authorship requirements are met, and that the publications which are created are of the highest scientific quality.

The Publications Subcommittee will review and approve all manuscripts of Study results prior to publication.

All study participants (Investigators and Committee members) give full authority to the ESC for primary presentation and/or primary publication of results. No other publication is allowed before the primary publication. Any subsequent presentation or publication by a study participant must be approved by this Publications Committee and/or ESC and make reference to the study and the primary publication.

As the study is being conducted at multiple sites, the Sponsor and the Publications Subcommittee agree that, consistent with scientific standards, first presentation or publication of the results of the study shall be made only as part of a publication of the results obtained by all sites performing the protocol. However, if no multicenter publication has occurred within twelve (12) months of the completion of this study at all sites, the Investigator shall have the right to publish or present independently the results of this study to the review procedure set forth herein. The Investigator shall provide the Sponsor and the Publications Subcommittee with a copy of any such presentation or publication derived from the study for review and comment at least 30 days in advance of any presentation or submission for publication. In addition, if requested by the Sponsor, any presentation or submission for publication shall be delayed for a limited time, not to exceed 90 days, to allow for filing of a patent application or such other measures as the Sponsor deems appropriate to establish and preserve its proprietary rights.

The Investigator shall not use the name(s) of the Sponsor and/or its employees in advertising or promotional material or publication without the prior written consent of the Sponsor. The Sponsor shall not use the name(s) of the Investigator and/or the collaborators in advertising or promotional material or publication without having received his/her and/or their prior written consent(s).

The Sponsor has the right at any time to publish the results of the study.

(electronic 1.0)

15 CLINICAL TRIAL PROTOCOL AMENDMENTS

All appendices attached hereto and referred to herein are made part of this clinical trial protocol.

The Investigator should not implement any deviation from, or changes of the clinical trial protocol without agreement by the Sponsor and prior review and documented approval/favorable opinion from the IRB/IEC of an amendment, except where necessary to eliminate an immediate hazard(s) to clinical trial Patients, or when the change(s) involves only logistical or administrative aspects of the trial. Any change agreed upon will be recorded in writing, the written amendment will be signed by the Investigator and by the Sponsor and the signed amendment will be filed with this clinical trial protocol.

Any amendment to the clinical trial protocol requires written approval/favorable opinion by the Ethics Committee (IRB/IEC) prior to its implementation, unless there are overriding safety reasons.

In some instances, an amendment may require a change to the informed consent form. The Investigator must receive an IRB/IEC approval/favorable opinion concerning the revised informed consent form prior to implementation of the change and patient signature should be re-collected if necessary.

16 BIBLIOGRAPHIC REFERENCES

1. Alirocumab CIB - Refer to Latest Annual Update

2. Yusuf S, Zhao F, Mehta SR, Chrolavicius S, Tognoni G, Fox KK et al. Effects of clopidogrel in addition to aspirin in patients with acute coronary syndromes without ST-segment elevation. N Engl J Med 2001; 345(7):494-502.

3. Antman EM, Hand M, Armstrong PW, Bates ER, Green LA, Halasyamani LK et al. 2007 focused update of the ACC/AHA 2004 guidelines for the management of patients with ST-elevation myocardial infarction: a report of the American College of Cardiology/American Heart Association Task Force on Practice Guidelines (Writing Group to Review New Evidence and Update the ACC/AHA 2004 Guidelines for the Management of Patients With ST-Elevation Myocardial Infarction). J Am Coll Cardiol 2008; 51(2): 210–47.

4. Anderson JL, Adams CD, Antman EM, Bridges CR, Califf RM, Casey DE et al., ACC/AHA 2007 guidelines for the management of patients with unstable angina/non–ST-elevation myocardial infarction: a report of the American College of Cardiology/American Heart Association Task Force on Practice Guidelines (Writing Committee to Revise the 2002 Guidelines for the Management of Patients With Unstable Angina/Non–ST-Elevation Myocardial Infarction). J Am Coll Cardiol 2007; 50(7):e1-e157

5. Krumholz HM, Anderson JL, Bachelder BL, Fesmire FM, Fihn SD, Foody JM et al., ACC/AHA 2008 performance measures for adults with ST-elevation and non–STelevation myocardial infarction: a report of the American College of Cardiology/American Heart Association Task Force on Performance Measures (Writing Committee to Develop Performance Measures for ST-Elevation and Non–ST-Elevation Myocardial Infarction). J Am Coll Cardiol 2008; 52(24): 2046 –99.

6. Newman C, Tsai J, Szarek M, Luo D, Gibson E. Comparative safety of atorvastatin 80 mg versus 10 mg derived from analysis of 49 completed trials in 14,236 patients. Am J Cardiol 2006; 97(1): 61-7.

7. LaRosa JC, Grundy SM, Waters DD, Shear C, Barter P, Fruchart JC et al, for the Treating to New Targets (TNT) Investigators. Intensive lipid lowering with atorvastatin in patients with stable coronary disease. N Engl J Med 2005; 352 (14): 1425-35.

8. Cannon CP, Braunwald E, McCabe CH, Rader DJ, Rouleau JL, Belder R et al, for the Pravastatin or Atorvastatin Evaluation and Infection Therapy-Thrombolysis in Myocardial Infarction 22 Investigators. Intensive versus moderate lipid lowering with statins after acute coronary syndromes. N Engl J Med. 2004;350(15):1495-1504.

9. Ridker PM, Danielson E, Fonseca FA, Genest J, Gotto AM, Kastelein JJ at al, for the JUPITER Study Group. Rosuvastatin to prevent vascular events in men and women with elevated C-reactive protein. N Engl J Med 2008; 359 (21): 2195-207.

10. Grundy SM, Cleeman JI, Merz CNB, Brewer B Jr, Clark LT, Hunninghake DB et al. Implications of recent clinical trials for the National Cholesterol Education Program Adult Treatment Panel III Guidelines. Circulation. 2004;110(2):227-39.

11. Reiner Z, Catapano AL, De Backer G, Graham I, Taskinen MR, Wiklund et al, for The Task Force for the management of dyslipidaemias of the European Society of Cardiology (ESC) and the European Atherosclerosis Society (EAS). ESC/EAS Guidelines for the management of dyslipidaemias. Eur Heart J 2011; 32(14): 1769-818.

12. Wiviott SD, Cannon CP, Morrow DA, Ray KK, Pfeffer MA, Braunwald E, PROVE IT-TIMI 22 Investigators. Can low-density lipoprotein be too low? The safety and efficacy of achieving very low low-density lipoprotein with intensive statin therapy: a PROVE IT-TIMI 22 substudy. J Am Coll Cardiol 2005; 46(8): 1411-6.

13. LaRosa JC, Grundy SM, Kastelein JJ, Kostis JB, Greten H. Treating to New Targets (TNT) Steering Committee and Investigators. Safety and efficacy of Atorvastatin-induced very low-density lipoprotein cholesterol levels in Patients with coronary heart disease (a post hoc analysis of the treating to new targets [TNT] study). Am J Cardiol 2007; 100(5): 747-52.

14. Hsia J, MacFadyen JG, Monyak J, Ridker PM. Cardiovascular event reduction and adverse events among subjects attaining low-density lipoprotein cholesterol <50 mg/dl with rosuvastatin. The JUPITER trial (Justification for the Use of Statins in Prevention: an Intervention Trial Evaluating Rosuvastatin). J Am Coll Cardiol 2011; 57(16): 1666-75.

15. Everett BM, Mora S, Grossbard J, MacFadyen J, Ridker PM. Safety profile of statin-treated patients with LDL-C < 30 mg/dL or LDL-C reduction of <70%. ACC Meeting, 2012.

16. Baigent C, Blackwell L, Emberson J, Holland LE, Reith C, Bhala N et al. Cholesterol Treatment Trialists' (CTT) Collaboration. Efficacy and safety of more intensive lowering of LDL cholesterol: a meta-analysis of data from 170 000 participants in 26 randomised trials. Lancet 2010; 376(9753): 1670–81.

17. Emberson JR, Kearney PM, Blackwell L, Newman C, Reith C, Bhala Net al., Cholesterol Treatment Trialists' (CTT) Collaboration. Lack of effect of lowering LDL cholesterol on cancer: meta-analysis of individual data from 175,000 people in 27 randomised trials of statin therapy. PLoS One 2012; 7(1): e29849.

18. Brown MS, Goldstein JL. A receptor-mediated pathway for cholesterol homeostasis. Science 1986; 232(4746): 34-47.

19. Hochholzer W and Giugliano RP. Lipid lowering goals: back to nature? Ther Adv Cardiovasc Dis 2010; 4(3):185-91.

20. Cordain L, Eaton SB, Miller JB, Mann N, Hill K. The paradoxical nature of hunter-gatherer diets: meat-based, yet non-atherogenic. Eur J Clin Nutr 2002; 56, Suppl 1: S42-S52.

21. Yan AT, Yan RT, Tan M, Hackam DG, Leblanc KL, Kertland et al. Contemporary management of dyslipidemia in high-risk patients: Targets still not met. Am J Med 2006; 119(8): 676-83.

22. Virani SS, Wooddard LD, Landrum CR, Pietz K, Wang D, Ballantyne CM et al. Institutional, provider, and patient correlates of low-density lipoprotein and non-high-density lipoprotein cholesterol goal attainment according to the Adult Treatment Panel III guidelines. Am Heart J 2011; 161(6):1140-6.

23. Horton JD, Cohen JC, Hobbs HH. Molecular biology of PCSK9: its role in LDL metabolism. Trends Biochem Sci. 2007; 32(2): 71-7.

24. Benjannet S, Rhainds D, Essalmani R, Mayne J, Wickham L, Jin W, et al. NARC-1/PCSK9 and its natural mutants: zymogen cleavage and effects on the low density lipoprotein (LDL) receptor and LDL cholesterol. J Biol Chem 2004; 279(47): 48865–75.

25. Park SW, Moon YA, Horton JD. Post-transcriptional regulation of low density lipoprotein receptor protein by proprotein convertase subtilisin/kexin type 9a in mouse liver. J Biol Chem 2004; 279(48): 50630-8.

26. Rashid S, Curtis DE, Garuti R, Anderson NN, Bashmakov Y, Ho YK, et al. Decreased plasma cholesterol and hypersensitivity to statins in mice lacking PCSK9. Proc Natl Acad Sci USA 2005; 102(15): 5374-9.

27. Abifadel M, Varret M, Rabes JP, Allard D, Ouguerram K, Devillers M, et al. Mutations in PCSK9 cause autosomal dominant hypercholesterolemia. Nat Genet 2003; 34(2): 154-6.

28. Cohen JC, Boerwinkle E, Mosley TH, Hobbs HH. Sequence variations in PCSK9, low LDL, and protection against coronary heart disease. N Engl J Med 2006; 354(12): 1264-72.

29. Steinberg D, Witztum JL. Inhibition of PCSK9: a powerful weapon for achieving ideal LDL cholesterol levels. Proc Natl Acad Sci U S A. 2009; 106(24): 9546-7.

30. Zhao Z, Tuakli-Wosornu Y, Lagace TA, Kinch L, Grishin NV, Cohen JC et al. Molecular characterization of loss-of-function mutations in PCSK9 and identification of a compound heterozygote. Am J Hum Genet 2006; 79(3): 514-23.

31. Schwartz GG, Olsson AG, Ballantyne C, Barter PJ, Holme IM, Kallend D et al. Rationale and design of the dal-OUTCOMES trial: Efficacy and safety of dalcetrapib in patients with recent acute coronary syndrome, dal-OUTCOMES Committees. Am Heart J 2009; 158: 896-901.

32. Cannon CP, Giugliano RP, Blazing MA, Harrington RA, Peterson JL, Sisk CM et al. Rationale and design of IMPROVE-IT (IMProved Reduction of Outcomes: Vytorin Efficacy International Trial): Comparison of ezetimbe/simvastatin versus simvastatin monotherapy on cardiovascular outcomes in patients with acute coronary syndrome. Am Heart J 2008; 156(5): 826-32.

33. International Conference on Harmonisation of Technical Requirements for Registration of Pharmaceuticals for Human Use. M3(R2): Guidance on nonclinical safety studies for the conduct of human clinical trials and marketing authorization for pharmaceuticals. ICH 2009 Step 4:1-25

34. Hicks KA, Hung HM.J, Mahaffey KW, Mehran R, Nissen SE, Stockbridge NL, Targum SL, Temple R; on behalf of the Standardized Data Collection for Cardiovascular Trials Initiative. Standardized Definitions for End Point Events in Cardiovascular Trials. May 31, 2011.

35. Thygesen K, Alpert JS, White HD, Jaffe AS, Apple FS, Galvani M et al. on behalf of the Joint ESC/ACCF/AHA/WHF Task Force for the Redefinition of Myocardial Infarction. Universal definition of myocardial infarction. Circulation 2007; 116(22): 2634-53.

36. Mendis S, Thygesen K, Kuulasmaa K, Giampaoli S, Mähönen M, Blackett KN, Lisheng L and Writing group on behalf of the participating experts of the WHO consultation for revision of WHO definition of myocardial infarction. World Health Organization definition of myocardial infarction: 2008–09 revision. Int J Epidemiol 2011; 40 (1): 139-46.

37. Thygesen K, Alpert JS, Jaffe AS, Simoons ML, Chaitman BR, White HD et al. Third Universal Definition of Myocardial Infarction. J Am Coll Cardiol Oct 2012;60(16):1581-98

38. Friedewald WT, Levy RI, Fredrickson DS. Estimation of the concentration of Low-Density Lipoprotein Cholesterol in Plasma, without use of the preparative ultracentrifuge. Clin Chem 1972; 18(6):499-502.

39. Dolan P. Modeling valuations for EuroQol health states. Med Care 1997;35(11):1095-1108.

40. American Diabetes Association Standards of Medical Care in Diabetes—2012. Diabetes Care. Jan 2012;35 Suppl 1:S11-63.

41. Chobanian AV, Bakris GL, Black HR, Cushman WC, Green LA, Izzo JL Jr et al, and the National High Blood Pressure Education Program Coordinating Committee. The Seventh Report of the Joint National Committee on Prevention, Detection, Evaluation, and Treatment of High Blood Pressure: the JNC 7 report. JAMA. 2003; 289(19): 2560-72.

17 APPENDICES

Appendix A Monitoring and Management Plan for LDL-C < 25 mg/dL (0.65 mmol/L)

The monitoring and management plan for patients reaching an LDL-C < 25 mg/dL (0.65 mmol/L) consists of 2 separate but integrated systems (subsequently detailed below):

- An independent external academic physician and a dedicated member of the Phase 3a DMC under the auspices of the CV DMC
- A blinded automated process for dose adjustment/continuation/discontinuation.

An independent external academic physician(s) (also known as independent physician) and the dedicated member of the Phase 3a DMC (implemented for all Phase 3 studies evaluating the efficacy and safety of alirocumab on LDL-C; called Phase 3a studies) will be the coordinators of this specific monitoring related to first bullet above.

An independent physician will be notified by the central laboratory of all patients who achieve two consecutive LDL-C <25 mg/dL (0.65 mmol/L). The independent physician will perform additional monitoring which includes (1) patient level listing for surveillance and review for patients who are managed by IVRS/IWRS (i.e with planned automatic down-titration from 150mg to 75mg, or switch from 75mg to placebo) and (2) individual patient profile monitoring for potential site alerts in patients on alirocumab 75 mg and not managed by IVRS/IWRS (i.e. no planned automatic down-titration or switch to placebo by IVRS/IWRS). This process will occur, as soon as possible, after such notification from the central laboratory. Among data reviewed are any AEs potentially associated with low LDL-C. These include, but are not limited to, disorders related to fat-soluble vitamin deficiency, adrenal insufficiency, hypogonadism, neuropathies and hemorrhagic stroke. The independent physician will communicate with the dedicated Phase 3a DMC member on an expedited basis, as needed, or routinely. This dedicated Phase 3a DMC member will then transmit any information as necessary to the common member involved in both DMCs (Phase 3a DMC and CV DMC). The independent physician may elect to regularly monitor and track such patients throughout the study, as outlined below.

Specific actions can be undertaken depending on the alirocumab dose and the LDL-C levels achieved, as outlined below:

- **On dose of 150 mg Q2W**: if LDL-C <25 mg/dL (including LDL-C <15 mg/dL) on 2 consecutive values, a down-titration to 75 mg Q2W will be automatically done in a blinded manner at the next visit. The patient will remain at the down titrated dose of 75 mg Q2W for the remaining duration of the study unless patient meets criteria below. As described above, specific monitoring consisting of review of patient level listing will be implemented.
- **On dose of 75 mg Q2W:**
  - If LDL-C <25 mg/dL on 2 consecutive values but there are no 2 consecutive measurements < 15 mg/dL (i.e. no measurement < 15 mg/dL or only one occasional measurement < 15 mg/dL): study treatment with 75 mg Q2W will be continued but additional monitoring with review of individual patient profile will be implemented by

the independent physician to further confirm the safety of low LDL-C levels. An alert related to 2 consecutive LDL-C < 25 mg/dL (0.65 mmol/L) may be sent to the site at the request of the independent physician, after consultation with dedicated phase 3a DMC member (see below for further details).

- If measured LDL-C <15 mg/dL on 2 consecutive measurements: study treatment with 75 mg will be discontinued at the next visit. In order to maintain the blind there will be blinded substitution to placebo injections for the remaining duration of the study. Unless the LDL-C level remains below 25 mg/dL, the independent physician will, after some time, stop the review of the given patient.

As lipid values will not be communicated to investigators after randomization to maintain the blind, the continuation of the 75 mg dose, the up-titration to the 150 mg dose, the down-titration from 150 to 75 mg, or discontinuation will all occur automatically, without site or patient awareness. Moreover, blinded dose adjustment will avoid the need for dummy titrations in the placebo group.

Under exceptional circumstances the decision can be made by the dedicated Phase 3a DMC member to send an alert related to 2 consecutive LDL-C < 25 mg/dL (0.65 mmol/L) to the site. If this were to occur, then:

- The central lab will notify the Investigator
- The Investigator should then follow the recommended steps outlined below for an alert related to 2 consecutive LDL-C <25 mg/dL (0.65 mmol/L). These steps may include:
  - Call the patient as soon as possible to inquire about interval occurrence of AEs
  - Decide whether the patient should be requested to rapidly have an unscheduled site visit, or assessment could be done at the next scheduled visit
- At the site visit, plan for the following, based on his/her medical judgment:
  - Assess the need for conducting clinical investigations, arranging specialist consultation(s) as needed, and any relevant additional work-up
  - Assess the need for study treatment temporary or permanent discontinuation, or continuation. Regardless of the action taken regarding study treatment, the patient should continue the study as per Section 10.3.

In addition to the Investigator oversight, the independent physician continues monitoring the patient and communicating with the designated Phase 3a DMC member as described above.

As mentioned above the dedicated Phase 3a DMC member will then transmit any information as necessary to the common member involved in both DMCs (Phase 3a DMC and CV DMC). The CV DMC will thoroughly analyze the aggregate data for patients who achieve LDL-C <25 mg/dL during their periodic reviews throughout the study. The CV DMC may adjust the above monitoring plan, if needed.

Appendix B DNA storage samples

1. PROCEDURE FOR COLLECTION, HANDLING, AND SHIPMENT OF OPTIONAL DNA STORAGE SPECIMENS

- Collection schedule: per protocol.
- Labeling of samples
  - Each sample tube should have attached to it the label provided by Covance.

DNA Subject ID:XXX-001-YYY<br>Study Number/Compound (pre-printed)<br>Bar Code (preprinted)<br>Accession Number (pre-printed)

  - In the event of damage or loss of the provided labels, a new label should be immediately requested from Covance.
- Procedure
  - Using a **waterproof pen**, write Subject ID Number on label in space provided.
  - Collect 6 mL of blood, using the 6 mL Vacutainer (Becton Dickinson; K2 EDTA with HEMOGARD Closure) provided, and gently invert tube at least 8 times permitting the specimen to mix with the anticoagulant.
  - **Under no circumstances should the tube be centrifuged.**
  - Ensure the sample tube is clearly and appropriately labeled as described above and in detail in the Covance Laboratory Manual.
  - Immediately freeze and maintain the blood in an upright position at -20°C or colder for storage. Samples must be stored on dry ice if a freezer is not immediately available.
  - Complete the Laboratory Requisition Form (provided by Covance) for each sample.
- Storage
  - Samples must be kept at -20 ºC or colder, organized in a rack in numeric order according to the Subject ID, until ready for packaging and shipping
- Packaging and shipment
  - Samples and accompanying documents should be packaged according to the detailed instructions in the Covance Laboratory Manual provided at the initiation of the study
  - Samples must be packaged according to IATA Dangerous Goods Regulations, Packing Instructions 650, using the packing materials provided by contracted company
  - In the event that the packaging materials or instructions are lost, please contact the study Sponsor

- Ship samples on dry ice to Covance as described in the Global Study Schedule, using the shipping materials provided

- **Note:** Additional detailed information can be found in the Covance Laboratory Manual, provided at the beginning of the study. This includes additional details regarding:
  - Sample collection kits
  - Sample collection procedures
  - Documentation procedures
  - Packing and shipping instructions
  - Sample kit resupply
  - How to get help

2 SHIPMENT CONTACT NAMES AND ADDRESSES

Please refer to latest version of Operations Manual for Contact names and addresses for Optional DNA Banking Samples

Appendix C General Guidance for the follow-up of laboratory abnormalities by Sanofi

Please refer also to Section 10.4.7. – Guidelines for management of specific laboratory abnormalities.

(electronic 1.0)

NEUTROPENIA

Neutrophils < 1500/mm^3 or according to ethnic group

↓

Repeat immediately a full blood count if value close to 1500/mm^3

↓

Neutrophils < 1500/mm^3 confirmed with signs of infection	Neutrophils < 1500/mm^3 confirmed with no signs of infection
1. **DISCONTINUE** Investigational Medicinal Product, hospitalization should be considered<br>2. **PERFORM** biological investigations for infection	1. **DISCONTINUE** Investigational Medicinal Product<br>2. **INVESTIGATE** for infection

↓

In both situations

3. **INFORM** the local monitor
4. **INVESTIGATE** previous treatments particularly long-term, even a long time ago, exposure to toxic agents, e.g., benzene, X-rays, etc.
5. **PERFORM** and collect the following investigations (results):
   - RBC and platelet counts
   - Serology: EBV, (HIV), mumps, measles, rubella
6. **DECISION** for bone marrow aspiration: to be taken in specialized unit
7. **FREEZE** serum (5 mL x 2) on Day 1 (cessation of investigational medicinal product) and Day 5 (for further investigations)
8. **MONITOR** the leukocyte count 3 times per week for at least one week, then twice a month until it returns to normal

Note:

•The procedures described in the above flowchart are to be discussed with the patient only in case the event occurs. If applicable (according to local regulations), an additional consent (e.g., for HIV testing) will only be obtained in the case the event actually occurs.

•For individuals of African descent, the relevant value of concern is <1000/mm3

Neutropenia are to be recorded as AE only if they are :

- Symptomatic, and/or
- Requiring either corrective treatment or consultation, and/or
- Leading to IMP discontinuation or modification of dosing, and/or
- Fulfilling a seriousness criterion [in that case, the event (SAE) should be notified within 24 hours to the MT], and/or
- Defined as an Adverse Event of Special Interest (AESI) with immediate notification

THROMBOCYTOPENIA

Platelets < 100 000/mm^3 (rule out EDTA – induced pseudo-thrombocytopenia)

↓

Repeat immediately the count (rule out EDTA anticoagulant in the sample)

↓

Platelets < 100 000/mm^3 confirmed with bleeding	Platelets < 100 000/mm^3 confirmed with no bleeding
1. **DISCONTINUE** Investigational Medicinal Product<br>2. **HOSPITALIZATION** should be considered	1. **DISCONTINUE** Investigational Medicinal Product<br>2. **INVESTIGATE** for bleeding

↓

In both situations

3. **INFORM** the local Monitor
4. **QUESTION** about last intake of quinine (drinks), alcoholism, heparin administration
5. **PERFORM** or collect the following investigations:
   - Complete blood count, schizocytes, creatinine
   - Bleeding time and coagulation test (fibrinogen, PT, aPTT), Fibrin Degradation Product
   - Viral serology: EBV, HIV, mumps, measles, rubella
6. **FREEZE** serum (5 mL x 2) on Day 1 (end of treatment) and Day 5 to test for drug-induced antiplatelets antibodies
7. **DECISION** for bone marrow aspiration: to be taken in specialized unit
   - On Day 1 in the case of associated anemia and/or leukopenia
   - On Day 8 if the Platelets remain < 50 000/mm^3
8. **MONITOR** the platelet count every day for at least one week and then regularly until it returns to normal

Note:
the procedures described in the above flowchart are to be discussed with the patient only in case the event occurs. If applicable (according to local regulations), an additional consent (e.g., for HIV testing) will only be obtained in the case the event actually occurs.

Thrombocytopenia are to be recorded as AE only if they are:

- Symptomatic, and/or
- Requiring either corrective treatment or consultation, and/or
- Leading to IMP discontinuation or modification of dosing, and/or
- Fulfilling a seriousness criterion [in that case, the event (SAE) should be notified within 24 hours to the MT], and/or
- Defined as an Adverse Event of Special Interest (AESI) with immediate notification

INCREASE IN ALT

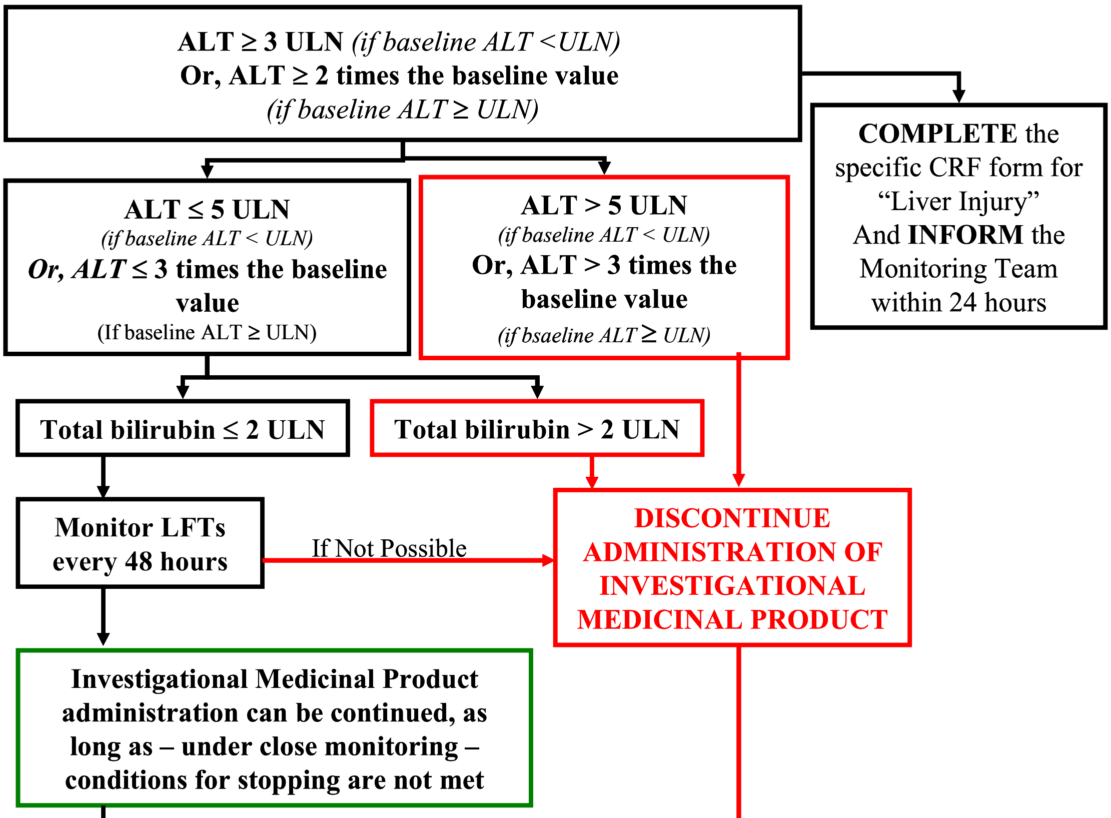

In ANY CASE, **FOLLOW** the instructions #1 to 6 listed in the box below.

1. **INVESTIGATE** specifically for malaise with or without loss of consciousness, dizziness, and/or hypotension and/or episode of arrhythmia in the previous 72 hours; rule out muscular injury
2. **PERFORM** the following tests:
   - LFTs : AST, ALT, Alkaline Phosphatase, Total and Conjugated Bilirubin and Prothrombin Time / INR
   - CPK, serum creatinine, complete blood count
   - Anti-HAV IgM, anti-HBc IgM, anti-HCV and HCV RNA , anti-CMV IgM and anti-HEV IgM antibodies,
   - Auto-antibodies : anti-nuclear, anti-DNA, anti-smooth muscle, anti-LKM
   - Depending on the clinical context, check for recent infection with EBV, Herpes viruses and toxoplasma
   - Hepatobiliary ultrasonography (can be completed by other imaging investigations if needed)
3. **CONSIDER** consultation with hepatologist
4. **CONSIDER** patient hospitalisation if INR>2 (or PT<50%) and/or central nervous system disturbances suggesting hepatic encephalopathy.
5. **MONITOR LFTs**
   - **If investigational medicinal product is continued**: **every 48 hours** until return to normal (<2ULN) or baseline. If ALT elevation persists beyond 2 weeks then perform LFTs every 2 weeks and 15 to 30 days after the last dose according to the study protocol.
   - **If investigational medicinal product is discontinued**: as closely as possible to every 48 hours until stabilization then every 2 weeks until return to normal (<2ULN) or baseline or for at least 3 months, whichever comes last.
6. **FREEZE** serum (5 ml X 2)

NOTE: IN ADDITION, AS SOON AS A SERIOUSNESS CRITERION IS MET, THE EVENT SHOULD BE NOTIFIED WITHIN 24 HOURS TO THE MONITORING TEAM.

ACUTE RENAL FAILURE

Rapid increase in serum creatinine over 150 µmol/L or rapid decrease in creatinine clearance below 50ml/mn

Can be rapidly reversed:
- By volume repletion
- Or relief of urinary tract obstruction (according to etiology)

1. Investigational Medicinal Product may be continued
2. **MONITOR** serum creatinine until return to baseline level

Cannot be rapidly reversed:
- Occurrence/aggravation of life threatening symptoms of ARF: anemia, hyperkalemia, hyperuricemia, metabolic acidosis, cardiac insufficiency, pulmonary edema, arrhythmia, DIC, etc.
- And/or predominant elimination of Investigational Medicinal Product by renal route

1. **INFORM** the local monitor
2. **DISCONTINUE** Investigational Medicinal Product administration
3. **HOSPITALIZATION** should be considered and seek for nephrologic advice
4. **PERFORM** the following examinations:
    - BP, HR, hydration status, ECG
    - Blood count
    - Liver function tests + CPK
    - Biochemistry, including urea
    - Urinalysis
5. **FREEZE** serum (5mL x 2)
6. **MONITOR** renal function until return to baseline level (every day at the beginning, then every week)

Acute renal failure is to be recorded as AE only if it is :
- Symptomatic, and/or
- Requiring either corrective treatment or consultation, and/or
- Leading to IMP discontinuation or modification of dosing, and/or
- Fulfilling a seriousness criterion [in that case, the event (SAE) should be notified within 24 hours to the MT], and/or
- Defined as an Adverse Event of Special Interest (AESI) with immediate notification

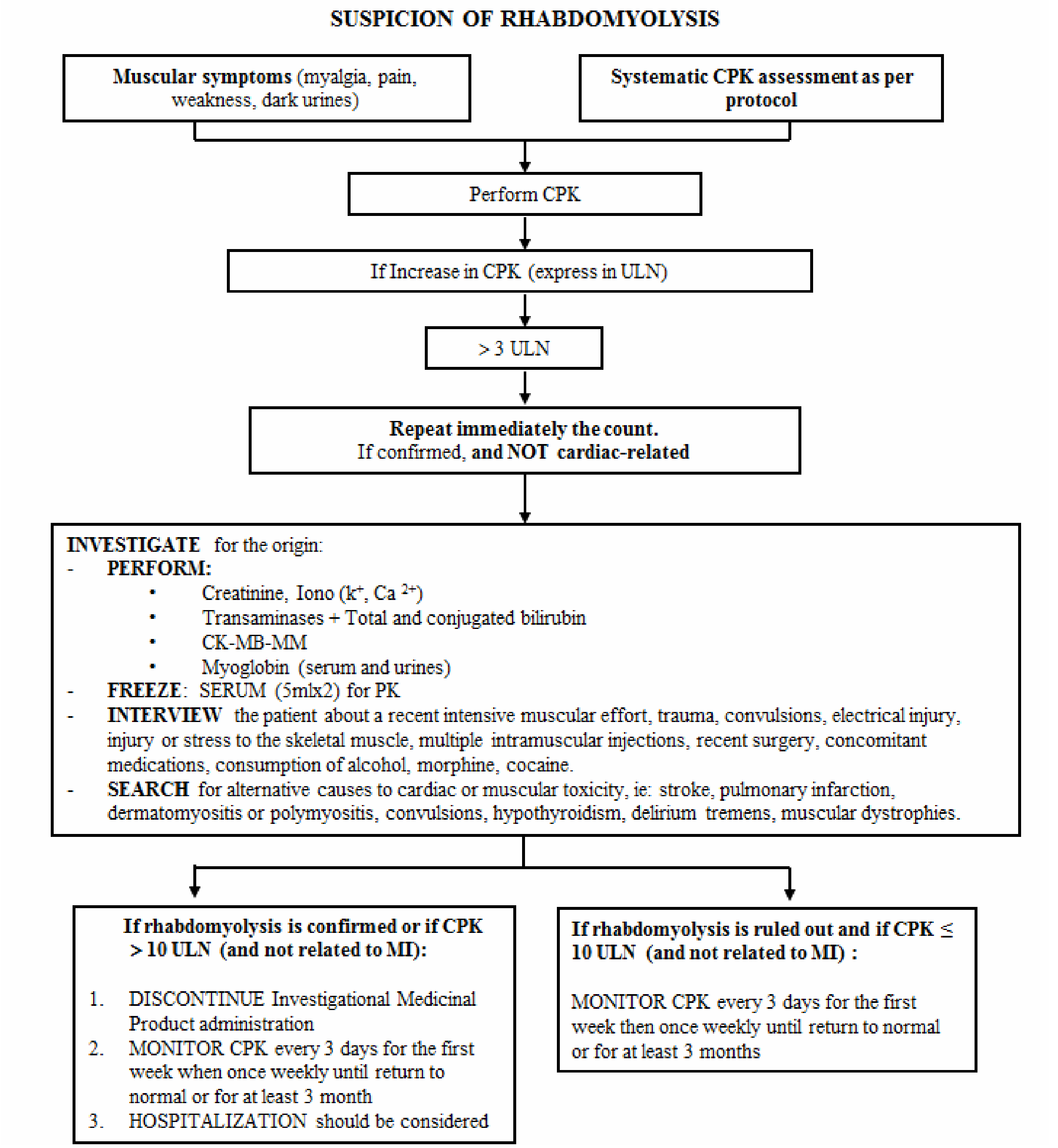

Suspicion of rhabdomyolysis is to be recorded as AE if it is:

- Symptomatic, and/or
- Requiring either corrective treatment or consultation, and/or
- Leading to IMP discontinuation or modification of dosing, and/or
- Fulfilling a seriousness criterion [in that case, the event (SAE) should be notified within ***24 hours*** to the Pharmacovigilance / Monitoring Team],
-

Appendix D Guidelines for hemoglobin (Hb) decrease ≥ 1.5 g/dL

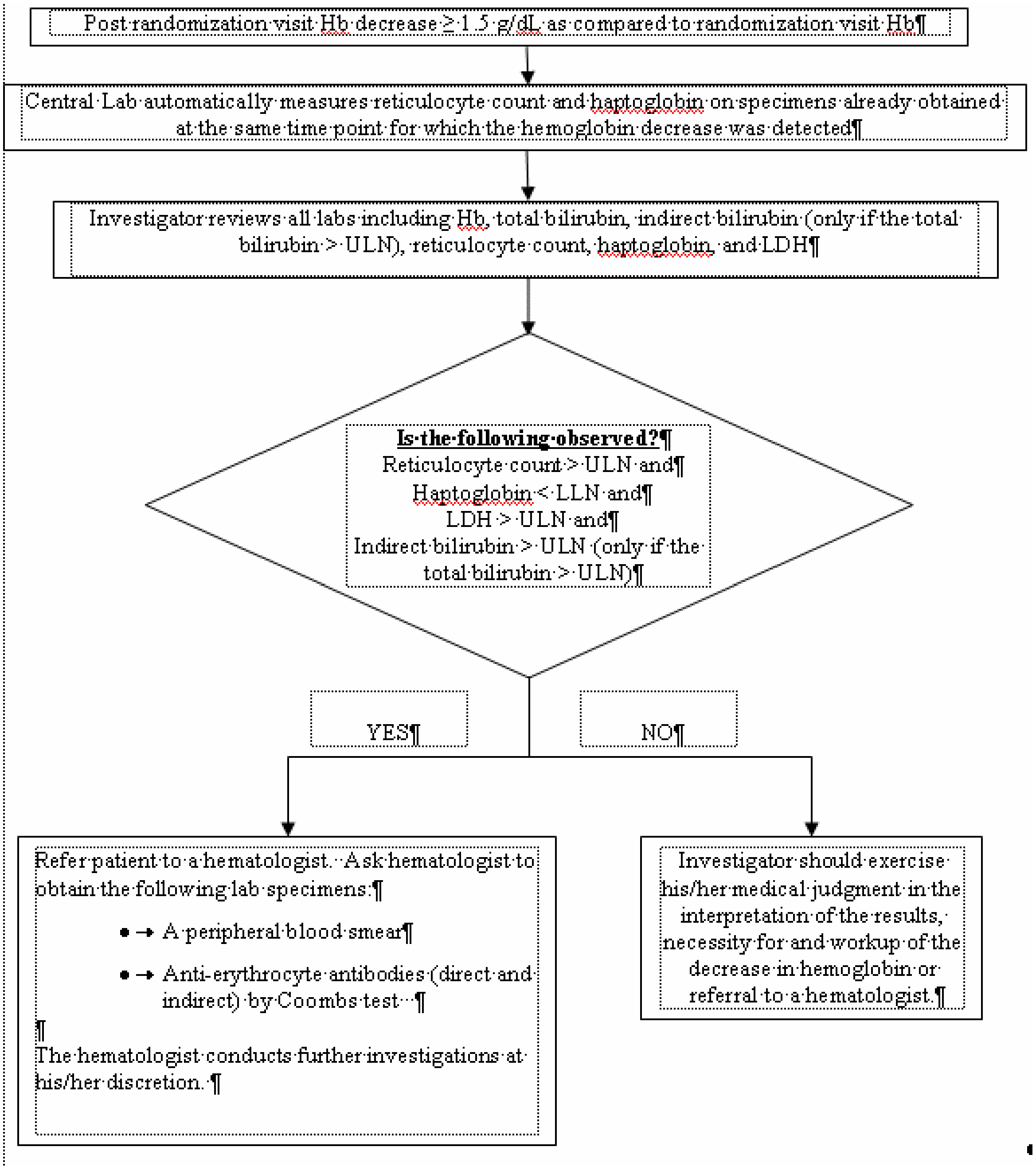

Suspicion of hemolytic anemia is recorded as AE only if:

- Symptomatic and/or
- Requiring either corrective treatment or consultation, and/or
- Leading to IMP discontinuation or modification of dosing, and/or
- Fulfilling a seriousness criterion

Appendix E Summary of Adverse Event and Suspected Efficacy Endpoint Reporting Instructions

This Appendix summarizes reporting instructions for reporting adverse events and suspected efficacy endpoint pages (please refer to CRF instruction manual for latest updated instructions).

EVENT CLASS	REPORTING TIMEFRAME (CRF, PV)	SPECIFIC EVENTS IN THIS CLASS	CRF Completion		
			Event form/ AE category in eCRF (1)	Safety Complementary Form	Other specific forms (8)
Adverse Event (non-SAE, non-AESI)	Routine	Any AE that is not SAE or AESI	AE	No	No
**Serious Adverse Event (SAE)** (non-AESI or AESI)	**Expedited (24 hours) (6)**	**Any AE meeting seriousness criterion**	AE	**Yes (SAE form)**	**<u>If fatal event</u>** Death Efficacy Endpoint Form must also be completed if death occurred during the period of interest (from randomization until Common Study End Date) (7)<br><br>**<u>If AESI</u>**, complete applicable specific forms (see below)
Adverse Event of Special Interest (**AESI**) **WITHOUT immediate notification** (non-SAE)	Routine	**Asymptomatic overdose with IMP**	Overdose (2)	No	No
		**Allergy events** (<u>Not requiring consultation</u> with another physician)	AE / 'General Allergic Reaction AE'	No	Allergic reaction <u>and/or</u> Local Injection site reaction complementary form (5)
		**Local injections site reactions** (<u>Not requiring consultation</u> with another physician)	AE/ 'Local Injection Site Reaction AE'	No	Allergic reaction <u>and/or</u> Local Injection site reaction complementary form (5)
		**Neurologic / Neurocognitive events** (<u>Not requiring</u> additional	AE / 'Neurologic-Neurocognitive AE'	No	Neurologic-Neurocognitive AE Complementary form

EVENT CLASS	REPORTING TIMEFRAME (CRF, PV)	SPECIFIC EVENTS IN THIS CLASS	CRF Completion		
			Event form/ AE category in eCRF (1)	Safety Complementary Form	Other specific forms (8)
		examinations/procedures or referral to a specialist)			
Adverse Event of Special Interest **(AESI) WITH immediate notification**<br><br>(non-SAE)	**Expedited (24 hours)**<br><br>**(6)**	**Pregnancy of female patient/subject OR partner pregnancy**	Pregnancy (2)	**Yes**	DEVP (3)
		**Symptomatic overdose with IMP**	Overdose (2) + AE forms for symptoms (4)	**Yes**	No
		**Increase in ALT** ALT ≥ 3 ULN (if baseline ALT < ULN) Or ALT ≥ 2 times the baseline value (if baseline ALT ≥ ULN)	AE / 'ALT increase AE'	**Yes**	ALT increase complementary form
		**Allergy events** (REQUIRING consultation with another physician) (8)	AE / 'General Allergic Reaction AE'	**Yes**	Allergic reaction and/or Local Injection site reaction complementary form (5)
		**Local injections site reactions** (REQUIRING consultation with another physician) (8)	AE/ 'Local Injection Site Reaction AE'	**Yes**	Allergic reaction and/or Local Injection site reaction complementary form (5)
		**Neurologic / Neurocognitive events** (REQUIRING additional examinations/procedures and/or referral to a specialist) (8)	AE / 'Neurologic – Neurocognitive AE'	**Yes**	Neurologic-Neurocognitive AE Complementary form
		**Hemolytic anemia** (Appendix D)	AE/ 'Hemolytic anemia'	**Yes**	Hemolytic anemia complementary form
**Laboratory, vital sign, or ECG abnormality**<br>(non-SAE, non-AESI) that is:<br>• symptomatic<br>• requiring	Routine	**Neutropenia**<br>(per protocol Appendix C, Section 10.4.7)	AE/ 'Other'	No	No
		**Thrombocytopenia**<br>(per protocol Appendix C, Section 10.4.7)	AE/ 'Other'	No	No
		**Acute renal insufficiency**<br>(per protocol Appendix C, Section 10.4.7)	AE/ 'Other'	No	No

EVENT CLASS	REPORTING TIMEFRAME (CRF, PV)	SPECIFIC EVENTS IN THIS CLASS	CRF Completion		
			Event form/ AE category in eCRF (1)	Safety Complementary Form	Other specific forms (8)
corrective treatment or consultation (8)<br>• leading to IMP discontinuation or dose modification		**Increase in CPK and suspicion of rhabdomyolysis**<br>(per protocol Appendix C,Section 10.4.7)	AE/ 'Other'	No	No
**Suspected Efficacy Endpoints** occurring during the period of interest (from randomization until Common Study End Date / V30)	**Expedited (24 hours)**	- Any event with fatal outcome (7)<br>- Non-fatal MI,<br>- Non-fatal cerebrovascular event<br>- Unstable angina requiring admission to hospital or emergency room or occurring while hospitalized,<br>- Coronary revascularization (PCI, CABG)<br>- Congestive heart failure requiring admission to hospital or emergency room or occurring while hospitalized,	**Specific Efficacy Endpoint Form**<br><br>Fill out also AE page in 2 situations (1) if suspected efficacy endpoint presents with unusual features that make it unexpected in the context of the patient's underlying medical condition or (2) in case of fatal event, if initial event is not a suspected efficacy endpoint	**No**<br><br>If applicable (i.e. if event is serious), fill out also SAE page in 2 situations<br>(1) if suspected efficacy endpoint presents with unusual features that make it unexpected in the context of the patient's underlying medical condition<br>(2) in case of fatal event, if initial event is not a suspected efficacy endpoint	**If fatal event**, complete also 'Death' Efficacy Endpoint Form (7)<br><br>If cardiac cath only (i.e. without PCI) was performed, complete also cardiac cath page

EVENT CLASS	REPORTING TIMEFRAME (CRF, PV)	SPECIFIC EVENTS IN THIS CLASS	CRF Completion		
			Event form/ AE category in eCRF (1)	Safety Complementary Form	Other specific forms (8)
**Suspected efficacy endpoints** occurring outside the period of interest (i.e. outside interval from randomization until Common Study End Date / V30)	**Expedited (24 hours)**<br>**(if Serious)**	- Any event with fatal outcome (7)<br>- Non-fatal MI,<br>- Non-fatal cerebro-vascular event<br>- Unstable angina requiring admission to hospital or emergency room or occurring while hospitalized<br>- Coronary revascularization (PCI, CABG)<br>- Congestive heart failure requiring admission to hospital or emergency room or occurring while hospitalized	AE/ 'Other'	**Yes (if serious)**	No<br>(Death efficacy endpoint form must not be completed)
**Other cardiovascular events that are not suspected efficacy endpoints** (including unstable angina and congestive heart failure not requiring admission to hospitalization or emergency room, and not occurring while hospitalized)	**Expedited (24 hours)**<br>**(if Serious)**		AE/ 'Other'	**Yes (if serious)**	**If fatal event (7)**<br>Death Efficacy Endpoint Form for any event with fatal outcome which occurred during the period of interest (from randomization to Common Study End Date)

CRF = Case Report Form, PV = Pharmacovigilance

IMPORTANT NOTES RELATED TO Appendix E:

(1) in AE form, for each event reported 1 category needs to be ticked among the 6

Adverse Event Category	General Allergic Reaction AE<br>Local Injection Site Reaction AE<br>ALT Increase AE<br>Hemolytic Anemia AE<br>Neurologic/Neurocognitive AE<br>Other	☐<br>☐<br>☐<br>☐<br>☐<br>☐

(2) Separate forms exist for Overdose and Pregnancy

(3) DEVP form (Drug Exposure Via Parent) is not in eCRF but should be completed on paper format

(4) Symptoms due to IMP overdose are to be reported on separate AE forms
NOTE: if any overdose is suspected due to device malfunction, complete Product Technical Complaint form

(5) General Allergic Reaction or Local Injection Site Reaction sections of the Complementary Form should be completed as applicable according to the type of reaction (General or Local). However, for Local Injection Site Reactions which have progressed/expanded/worsened/etc, complete both sections (Local and General) of the Complementary form.

(6) In case of expedited reporting (within 24 hours) to Pharmacovigilance the following forms need also to be completed in eCRF (within 24h) in order to be submitted to Pharmacovigilance at the time of initial notification:

- Demography
- Medical History (all categories)
- Prior and concomitant Medications (all 3 categories)
- Investigational Medicinal Product
- End of Treatment (if applicable)

(7) In case of Death the specific Death Efficacy Endpoint form located within eCRF needs to be appropriately completed if date of death is equal to or greater than date of randomization and before or equal to Common study end date (V30); also, event which led to fatal outcome should be captured in efficacy endpoint page (if suspected efficacy endpoint led to fatal outcome) or otherwise in AE page, and in some circumstances in both pages (if suspected efficacy endpoint presents with unusual features that make it unexpected in the context of the patient's underlying medical condition)

(8) In case of additional examinations/procedures, or referral to another physician/specialist: please complete also a ‘Procedure’ Form in CRF

Appendix F Assessment of Local Injection Site Reactions

Reaction to Injectable Product	Mild (Grade 1)	Moderate (Grade 2)	Severe (Grade 3)	Very Severe (Grade 4)
Pain	Does not interfere with activity	Interferes with activity or repeated use of non-narcotic pain reliever	Prevents daily activity or repeated use of narcotic pain reliever	Emergency Room (ER) visit or hospitalization
Tenderness	Mild pain to touch	Pain with movement	Significant pain at rest	ER visit or hospitalization
Erythema / Redness *	2.5 – 5 cm	5.1 – 10 cm	>10 cm	Necrosis or exfoliative dermatitis
Swelling **	2.5 – 5 cm and does not interfere with activity	5.1 – 10 cm or interferes with activity	> 10 cm or prevents daily activity	Necrosis
Itching	Does not interfere with activity	Interferes with activity or repeated use of topical or systemic treatment	Prevents daily activity or leads to other significant dermatologic conditions (such as infection, scarring, etc.)	Emergency Room (ER) visit or hospitalization
Other (Please specify)***	No modification of daily activities and/or does not require symptomatic treatment	Hinders normal daily activities and/or requires symptomatic treatment.	Prevents daily activities and requires symptomatic treatment.	Emergency Room (ER) visit or hospitalization

* In addition to grading the measured local reaction at the greatest single diameter, the measurement should be recorded as a continuous variable

** Swelling should be evaluated and graded using the functional scale as well as the actual measurement

*** Please specify the other signs or symptoms (for example, hematoma, discoloration, re-activation, etc.)

ADAPTED from the toxicity grading scale table from the FDA Draft Guidance for Industry: Toxicity Grading Scale for Healthy Adult and Adolescent Volunteers Enrolled in Preventive Vaccine Clinical Trials April 2005

Appendix G EQ-5D Patient Questionnaire

[redacted]

Appendix H New York Heart Association Functional Classification (for Congestive Heart Failure)

For assessment of Exclusion Criteria E03

NYHA Class	Symptoms
I	Cardiac disease, but no symptoms and no limitation in ordinary physical activity, e.g. shortness of breath when walking, climbing stairs etc.
II	Mild symptoms (mild shortness of breath and/or angina) and slight limitation during ordinary activity.
III	Marked limitation in activity due to symptoms, even during less-than-ordinary activity, e.g. walking short distances (20–100 m).<br>Comfortable only at rest.
IV	Severe limitations. Experiences symptoms even while at rest. Mostly bedbound patients.

The Criteria Committee of the New York Heart Association. Nomenclature and Criteria for Diagnosis of Diseases of the Heart and Great Vessels. 9th ed. Boston, Mass: Little, Brown & Co; 1994:253-256.

Appendix I Stroke Functional Disability Score (adapted from Modified Rankin Scale)

In case of stroke, functional disability score will be assessed 3 to 6 months after date of onset of the event.

The score can range from 0 (perfect health without symptoms) to 5 (severe disability)

0	No symptoms
1	No significant disability. Able to carry out all usual activities, despite some symptoms.
2	Slight disability. Able to look after own affairs without assistance, but unable to carry out all previous activities.
3	Moderate disability. Requires some help, but able to walk unassisted.
4	Moderately severe disability. Unable to attend to own bodily needs without assistance, and unable to walk unassisted.
5	Severe disability. Requires constant nursing care and attention, bedridden, incontinent.

Adapted from Rankin J. Cerebral vascular accidents in patients over the age of 60. II. Prognosis". Scott Med J 1957;**2**(5): 200–15.

EFC11570 Amended protocol 11

ELECTRONIC SIGNATURES

Signed by	Meaning of Signature	Server Date (dd-MMM-yyyy HH:mm)
[redacted]		

ODYSSEY OUTCOMES Amendment Summary

Amendment 1 dated: 9/17/2012 GLOBAL AMENDMENT

The main reasons for this protocol amendment are described below:

- Change in the wording of the exclusion criteria E07 and E23:
  - For the exclusion criterion E07 related to a previous participation in any clinical trial of SAR236553 or any other anti-PCSK9 monoclonal antibody, clarification is made on the meaning of participation in order to allow the enrolment of patients who were not randomized and consequently not exposed to the treatment.
  - For the exclusion criterion E23 related to women of childbearing potential, the revision of the wording is made to allow fulfilling all applicable local regulatory requirements through the informed consent form or a local protocol addendum.
- The waived cardiovascular efficacy endpoints specified in this study (primary and secondary efficacy endpoints) will now be reported only in the specific outcome event screens/pages of the eCRF, and are no longer required to be also reported in the AE screen/page of the eCRF. This will avoid duplication of data reporting by the investigator, and possible inconsistencies. Sanofi pharmacovigilance database will be updated directly from the relevant screens/pages of the eCRF.
- Change in the timelines, from “within 1 working day” to “within 24 hours”, for reporting Serious Adverse Events and Adverse Events of Special Interest with immediate notification (including corresponding modifications in Appendix C and E) to comply with the European detailed guidance on the collection, verification and presentation of adverse event/reaction reports arising from clinical trials on medicinal products for human use (‘CT-3’), section 4.3.1 (June 2011).

Amendment 2 dated: 3/1/2013 LOCAL AMENDMENT FRANCE

Following the “Agence nationale de sécurité du médicament et des produits de santé” (ANSM) request, the Sponsor agrees to modify the protocol and to add a new exclusion criteria “Known history of active optic nerve disease”.

Amendment 3 dated: 3/22/2013 LOCAL AMENDMENT LATVIA

Following the request from Republic of Latvia State Agency of Medicine, the following changes will be done for Latvian patients:

• Addition of specific measures related to latent/ active tuberculosis as follows:

- For the exclusion criteria E09 related to laboratory findings during the screening period, an in vitro tuberculosis test is added with exclusion of patients who are tested positive.

- An in vitro tuberculosis test will be performed at screening Visit (Visit 1) and then every year.

• An ECG will be performed at all visits instead of initially planned in the protocol (i.e., at randomization visit (M0), and at common study end date (M64) or at early treatment discontinuation visit).

Amendment 4 dated: 3/25/2013 LOCAL AMENDMENT GERMANY

Following the request from German Health Authority, the following changes will be done for German patients:

• Changes related to the Exclusion criteria:
- Addition of an exclusion criterion related to known HIV infection.
- Revision of the exclusion criterion E 09 related to laboratory findings during the screening period with addition of a tuberculosis test to exclude patients who test positive for HIV.
- Exclusion criterion E 21 related to known hypersensitivity to monoclonal antibody therapeutics is revised to specify that it relates to any component of the drug product, including excipients.

Amendment 5 dated: 3/25/2013 LOCAL AMENDMENT CHINA

The main changes in the amendment are described below:

- update/clarifications in the definition of efficacy endpoints (primary and secondary) in order to better identify the events and ease the adjudication process
- run-in period: clarifications and simplifications provided, removal of previous restrictions on visits interval, clarifications and simplification regarding non-statin LMT (including possibility to introduce non-statin LMT other than ezetimibe during run-in period)
- some inclusion/exclusion criteria were adjusted (including simplified definition of ACS, clarification of exclusion criteria related to run-in period, hypertension)
- lipid monitoring and LMT post-randomization: adjustments in some lipid study assessments (LDL-C, ApoB), strengthening the message to Investigators to reinforce education/communication throughout the study to other people involved with patient care (patient’s family, GP/cardiologist) regarding study requirements including need to avoid checking lipid levels, possibility to modify background LMT post-randomization based on clinical tolerability, clarifications on LDL-C monitoring including role of independent physician in monitoring low LDL-C)
- streamline the study assessments after 6 months once patients are considered as having permanently discontinued treatment

Amendment 6 dated: 9/17/2012 GLOBAL AMENDMENT

The main reasons for this amendment are described below:

- update/clarifications in the definition of efficacy endpoints (primary and secondary) in order to better identify the events and ease the adjudication process.
- adjustment of some inclusion/exclusion criteria, including but not limited to: simplified definition of ACS, ACS window for randomization extended from 16 weeks to 52 weeks, clarification of exclusion criteria related to run-in period, hypertension.
- run-in period: clarifications and simplifications provided (with details added on how to practically conduct run-in period and adjust required background LMT before V2), adjustment of previous

restrictions on visit intervals (including possibility to combine V1 and V2 visits), duration of run-in period now reduced to 2 weeks but capped at about 16 weeks, possibility for rescreening a patient (with certain conditions), requirement that randomization (V3) should occur between about 4 to 52 weeks post ACS, clarifications and simplification regarding non-statin LMT (including possibility to introduce non-statin LMT other than ezetimibe during run-in period).

- lipid monitoring and LMT post-randomization: adjustments in some lipid study assessments (LDL-C, ApoB), strengthening the message to Investigators to reinforce education/communication throughout the study to other people involved with patient care (patient's family, GP/cardiologist) regarding study requirements including need to avoid checking lipid levels, possibility to modify background LMT post-randomization based on clinical tolerability, clarifications on LDL-C monitoring including role of independent physician in monitoring low LDL-C, possibility to use LDL cholesterol (LDL-C) value at Month 2 (V5) for up-titration at Month 4 (V6) in case of missing or invalid LDL-C at Month 1 (V4).
- clarification in safety (SAE) section and addition of an analysis of cardiovascular events of interest (other than efficacy endpoints) related to peripheral arterial disease and venous thromboembolic events.

Amendment 7 dated: 4/11/2014 LOCAL AMENDMENT CHINA

The main changes in this amendment (compared to the latest amended protocol in China – dated July 29-2013) are described below:

- extension of window from ACS index event to randomization from 16 weeks to 52 weeks.
- run-in period: clarifications provided on intervals during run-in period, duration of run-in period reduced to a minimum of 2 weeks but capped at about 16 weeks, possibility for rescreening a patient (with certain conditions), requirement that randomization (V3) should occur between about 4 to 52 weeks post ACS.
- lipid monitoring and LMT post-randomization: adjustments in some lipid study assessments at the common study end date for non-completers (LDL-C, ApoB), possibility to use LDL cholesterol (LDL-C) value at Month 2 (V5) for up-titration at Month 4 (V6) in case of missing or invalid LDL-C at Month 1 (V4).
- addition of an analysis of cardiovascular events of interest (other than efficacy endpoints) related to peripheral arterial disease and venous thromboembolic events.

Amendment 8 dated: 4/11/2014 GLOBAL AMENDMENT

The main reasons for this amendment are described below:

- It includes a plan to randomize at least 600 patients in China for local regulatory requirements. However, as the overall target of 18,000 patients is likely to be reached before the 600 patients in China, the amendment proposes to close randomization in all countries – except China – shortly after 18,000 patients have been randomized globally, and then close randomization in China, shortly after 600 patients have been randomized in China. All randomized patients (~18,600) will be included in the analysis. The amendment does not propose to postpone the timing for the end of the trial; therefore, the

randomized population will include about 18,000 patients who have either died or been followed for a minimum of 24 months, supplemented by an additional subset of patients from China (~600), possibly followed for less than 24 months. The study end date definition has been modified as follows: the date corresponding to 24 months after the closing of randomization except for China or the date when the target number of events (1613) is reached, whichever comes last.

- Neurologic and neurocognitive events are now considered as adverse events of special interest (AESI) and will require completion of additional CRF page(s). In addition, neurologic and neurocognitive events leading to additional examinations/procedures and/or referral to a specialist will be considered as 'AESI with immediate notification' and be subject to expedited reporting (within 24 hours) to the Sponsor
- If lipid inclusion criteria at V2/V2b (LDL-C, non-HDL-C, Apo B) were met during a (first initial) screening with a given statin dose and the patient failed screening for a reason not related to these lipid inclusion criteria, then the patient can be rescreened using the same statin at same dose or, if documented intolerance, with same statin at lower dose or other authorized statin or no statin (later option only if documented intolerance to 2 or more statins). Reasons include but are not limited to: correction of exclusionary labs other than lipid inclusion criteria (i.e. other than LDL-C, non-HDL-C, Apo B), patient and/or family changed their mind regarding trial participation.

Amendment 9 dated: 12/10/2015 LOCAL CHINA

The main reason for this local protocol amendment 9 in China (which describes changes from Amended Protocol 8, dated 16-Apr-2015) is to follow the recommendation from China Food and Drug Agency (CFDA) and investigate the impact of the treatment on steroid hormones: "adrenocorticotropic hormone [ACTH], follicle stimulating hormone [FSH], luteinizing hormone [LH], cortisol, testosterone and estradiol".

Amendment 10 dated: 1/27/2016 LOCAL COLUMBIA

The objective of this local protocol amendment 10 in Colombia is to add an annual neurologic examination (to be performed by a neurologist), as requested by Colombia Health Authorities, in order to enhance the detection of potential neurologic or neurocognitive adverse events.

Since all patients have already been randomized (i.e. with V3 visit already performed), the neurologic examination is added as a post-randomization assessment, to be performed by a neurologist at the same timepoints as the (general) physical examination.

Amendment 11 dated: 2/25/2016 GLOBAL

The main reason for this global protocol amendment 11 is to streamline the requirements for the end of the study.

Only one final clinic visit, called 'Common Study End Date visit' (CSED visit) is now required, for all randomized patients, to be performed at the end of the study, and within 30 days of the CSED. Prior to this amendment, an additional contact (via phone or email) was required to take place 8 weeks after that final clinic visit, for patients who were receiving blinded study treatment at the end of the trial.

STATISTICAL ANALYSIS PLAN

A Randomized, Double-Blind, Placebo-Controlled, Parallel-Group Study to Evaluate the Effect of Alirocumab (SAR236553/REGN727) on the Occurrence of Cardiovascular Events in Patients who have Recently Experienced an Acute Coronary Syndrome.

ODYSSEY Outcomes

SAR236553/REGN727-EFC11570

STATISTICIAN: [redacted]

BIOSTATISTICS PROJECT LEADER: [redacted]

DATE OF ISSUE: 25-Jan-2016

Total number of pages: 83

Any and all information presented in this document shall be treated as confidential and shall remain the exclusive property of Sanofi (or any of its affiliated companies). The use of such confidential information must be restricted to the recipient for the agreed purpose and must not be disclosed, published or otherwise communicated to any unauthorized persons, for any reason, in any form whatsoever without the prior written consent of Sanofi (or the concerned affiliated company); 'affiliated company' means any corporation, partnership or other entity which at the date of communication or afterwards (i) controls directly or indirectly Sanofi, (ii) is directly or indirectly controlled by Sanofi, with 'control' meaning direct or indirect ownership of more than 50% of the capital stock or the voting rights in such corporation, partnership or other entity

TABLE OF CONTENTS

STATISTICAL ANALYSIS PLAN 1

TABLE OF CONTENTS 2

LIST OF ABBREVIATIONS AND DEFINITION OF TERMS 5

1 OVERVIEW AND INVESTIGATIONAL PLAN 7

1.1 STUDY DESIGN AND RANDOMIZATION 7

1.2 OBJECTIVES 7
1.2.1 Primary objective 7
1.2.2 Secondary objectives 8

1.3 DETERMINATION OF SAMPLE SIZE 8

2 STUDY PLAN 10

2.1 MODIFICATIONS TO THE STATISTICAL SECTION OF THE PROTOCOL 11

2.2 STATISTICAL MODIFICATIONS MADE IN THE STATISTICAL ANALYSIS PLAN 12

3 STATISTICAL AND ANALYTICAL PROCEDURES 13

3.1 ANALYSIS ENDPOINTS 13
3.1.1 Demographic and baseline characteristics 13
3.1.2 Prior or concomitant medications 16
3.1.3 Efficacy endpoints 17
3.1.3.1 Primary efficacy endpoint(s) 17
3.1.3.2 Secondary efficacy endpoint(s) 18
3.1.4 Safety endpoints 20
3.1.4.1 Adverse events variables 21
3.1.4.2 Deaths 22
3.1.4.3 Laboratory safety variables 22
3.1.4.4 Vital signs variables 23
3.1.4.5 Electrocardiogram variables 23
3.1.5 Other endpoints 23
3.1.5.1 Lipid parameters 23
3.1.5.2 hs-CRP 24
3.1.5.3 HbA_{1C} 24
3.1.5.4 Patients with LDL-C <25 mg/dL (0.65 mmol/L) 25
3.1.5.5 Anti-alirocumab antibodies assessed throughout the study. 25
3.1.5.6 Quality-of-life parameters 26

3.1.5.7 Cardiovascular events of interest (other than efficacy endpoints) 26
3.1.6 Pharmacokinetic variables 26
3.1.7 Pharmacogenomics endpoints....27
3.2 DISPOSITION OF PATIENTS 27
3.2.1 Randomization and drug dispensing irregularities 28
3.3 ANALYSIS POPULATIONS 29
3.3.1 Efficacy populations 29
3.3.1.1 Intent–to-treat population 29
3.3.2 Safety population....29
3.3.3 Anti-alirocumab antibody population 30
3.4 STATISTICAL METHODS 30
3.4.1 Demographics and baseline characteristics 30
3.4.2 Prior, concomitant or post-treatment medications 31
3.4.3 Extent of investigational medicinal product exposure and compliance....33
3.4.3.1 Extent of investigational medicinal product exposure 33
3.4.3.2 Compliance 34
3.4.4 Analyses of efficacy endpoints....34
3.4.4.1 Analysis of primary efficacy endpoint(s)....34
3.4.4.2 Analyses of secondary efficacy endpoints 37
3.4.4.3 Multiplicity issues....38
3.4.4.4 Additional efficacy analyses 38
3.4.5 Analyses of safety data 38
3.4.5.1 Analyses of adverse events 39
3.4.5.2 Deaths....46
3.4.5.3 Analyses of laboratory variables 46
3.4.5.4 Analyses of vital sign variables 48
3.4.6 Analyses of other safety parameters....49
3.4.7 Analyses of other endpoints....49
3.4.7.1 Analyses of hs-CRP 49
3.4.7.2 Analyses of HbA_{1c} 50
3.4.7.3 Analyses of patients with LDL-C <25 mg/dL(<0.65 mmol/L)....50
3.4.7.4 Analyses of lipid parameters 50
3.4.7.5 Analyses of anti-alirocumab antibody variables 52
3.4.7.6 Analyses of quality of life/health economics variables 52
3.4.7.7 Analysis of cardiovascular events of interest (other than efficacy endpoint)....53
3.4.8 Analyses of pharmacokinetic and pharmacodynamic variables 53
3.5 DATA HANDLING CONVENTIONS....53
3.5.1 General conventions 53
3.5.2 Data handling conventions for secondary efficacy variables....54
3.5.3 Missing data 55

3.5.4 Windows for time points 57

3.5.5 Unscheduled visits 57

3.5.6 Pooling of centers for statistical analyses 58

3.5.7 Statistical technical issues 58

4 INTERIM ANALYSIS 59

5 DATABASE LOCK 61

6 SOFTWARE DOCUMENTATION 62

7 REFERENCES 63

8 LIST OF APPENDICES 64

APPENDIX A POTENTIALLY CLINICALLY SIGNIFICANT ABNORMALITIES (PCSA) CRITERIA 65

APPENDIX B LIST OF MEDDRA TERMS FOR CMQS 70

APPENDIX C DERIVATION OF THE EFFICACY ENDPOINTS/COMPONENTS 73

APPENDIX D EQ-5D PATIENT QUESTIONNAIRE 78

APPENDIX E EQ-5D UTILITY SCORE ALGORITHM 79

APPENDIX F DETAILED STATISTICAL METHODOLOGY FOR PATTERN-MIXTURE MODEL 80

LIST OF ABBREVIATIONS AND DEFINITION OF TERMS

Ab:	antibody
ACE:	angiotensin converting enzyme
ACS:	acute coronary syndrome
ADA:	anti-alirocumab antibodies
ADP:	adenosine diphosphate
AESI:	adverse event of special interest
ALT:	alanine aminotransferase
ANCOVA:	analysis of covariance
Apo:	apolipoprotein
AST:	aspartate aminotransferase
ATC:	Anatomical Therapeutic Chemical
BMI:	body mass index
CABG:	coronary artery bypass graft surgery
CEC:	Clinical Events Committee
CHD:	coronary heart disease
CI:	confidence interval
CMQ:	company MedDRA query
CPK:	creatine phosphokinase
CSED:	common study end date
CV:	cardiovascular
DMC:	data monitoring committee
DVT:	deep vein thrombosis
ECG:	electrocardiogram
e-CRF:	electronic case report form
eGFR:	estimated glomerular filtration rate
HbA_{1c}:	glycated hemoglobin A1c
HCV:	hepatitis C virus
HDL-C:	high-density lipoprotein cholesterol
HLGT:	high level group term
HLT:	high level term
HR:	hazard ratio
hs-CRP:	high-sensitivity C-reactive protein
IA:	interim analysis
IMP:	investigational medicinal product
ITT:	intent-to-treat
IVRS:	interactive voice reponse system
IWRS:	interactive web response system
LDH:	lactate dehydrogenase
LDL-C:	low-density lipoprotein cholesterol
LLN:	lower limit of normal

LLOQ:	lower limit of quantification
LMT:	lipid modifying therapy
LMWH:	low molecular weight heparin
Lp (a):	lipoprotein (a)
MAR:	missing-at-random
MDRD:	Modification of the Diet in Renal Disease
MedDRA:	Medical Dictionary for Regulatory Activities
MI:	myocardial infarction
MMRM:	mixed-effect model with repeated measures
NSAID:	nonsteroidal anit-inflammatory drug
NSTEMI:	non ST segment elevation myocardial infarction
PCI:	percutaneous coronary intervention
PCSA:	potentially clinically significant abnormality(ies)
PE:	pulmonary embolism
PT:	preferred term
Q1:	first quartile
Q2W:	every 2 weeks
Q3:	third quartile
RNA:	ribonucleic acid
SAE:	serious adverse event
SAP:	statistical analysis plan
SD:	standard deviation
SE:	standard error
SMQ:	standardized MedDRA query
SOC:	system organ class
SPERT:	safety planning, evalutation, and reporting team
STEMI:	ST segment elevation myocardial infarction
TC:	total cholesterol
TEAE:	treatment-emergent adverse event
TG:	triglycerides
TIA:	transient ischemic attack
UA:	unstable angina
UFH:	unfractionated heparin
ULN:	upper limit of normal range
ULOQ:	upper limit of quantification
WHO-DD:	World Health Organization-Drug Dictionary
γGT:	gamma glutamyl transferase

1 OVERVIEW AND INVESTIGATIONAL PLAN

1.1 STUDY DESIGN AND RANDOMIZATION

This is a double-blind, randomized, placebo-controlled, balanced (1:1, alirocumab:placebo), parallel-group, multi-national, multicenter study.

Randomization takes place 4 weeks to 52 weeks after the index event and is stratified according to country.

Prior to randomization, eligible patients enter a run-in period of at least 2 weeks but no more than 16 weeks, during which they receive statin-intensive therapy defined as daily atorvastatin 40 or 80 mg, or rosuvastatin 20 or 40 mg. In case patients are unable to tolerate atorvastatin 40/80 mg or rosuvastatin 20/40 mg, they are allowed to receive the maximal tolerated dose of atorvastatin or rosuvastatin; or under some documented circumstances for statin-intolerant patients, receive other lipid lowering treatment other than a statin (eg, ezetimibe, or other non-statin lipid modifying therapy [LMT]), or no LMT at all.

Following this run-in period, only patients not reaching goal on their current LMT at the qualifying visit, ie, LDL-C ≥70 mg/dL (≥1.81 mmol/L) or apolipoprotein B (Apo B) ≥80 mg/dL (≥0.8 g/L) or non-HDL-C ≥100 mg/dL (≥2.59 mmol/L), are randomized to either background therapy + alirocumab or background therapy + placebo. All patients randomized to alirocumab initially receive alirocumab 75 mg every 2 weeks (Q2W). After randomization, patients on alirocumab not reaching the target LDL-C level at Month 1 have their dose up-titrated to 150 mg Q2W at Month 2 in a blinded fashion (in case Month 1 LDL-C is not available or not valid for potential up-titration at Month 2, the next available LDL-C sample at Month 2 is used for potential up-titration at Month 4).

The double-blind treatment period will continue until 24 months after the closing of randomization for all countries except for China or until the target number of events (1613) is reached, whichever comes last (this date will be the Common Study End Date [CSED]). The corresponding estimated study duration is 64 months. All patients, even if they have achieved an endpoint, or have prematurely discontinued study treatment, will be asked to remain in the study until the CSED and to come back to the site as close as possible to the CSED (ie, CSED visit).

1.2 OBJECTIVES

1.2.1 Primary objective

The primary objective of this study is to compare the effect of alirocumab with placebo on the occurrence of cardiovascular events (composite endpoint of coronary heart disease [CHD] death, non-fatal myocardial infarction [MI], fatal and non-fatal ischemic stroke, unstable angina requiring hospitalization) in patients who have experienced an acute coronary syndrome (ACS)

event 4 to 52 weeks prior to randomization and are treated with an LMT regimen that is statin-intensive (defined as atorvastatin 40 or 80 mg, or rosuvastatin 20 or 40 mg) or at maximally tolerated dose of these given statins, and optimized for long-term chronic use with other non-statin LMT(s) at Investigator's discretion.

1.2.2 Secondary objectives

The secondary objectives are:

To compare the efficacy of alirocumab versus placebo on secondary endpoints (any CHD event, major CHD event, any cardiovascular [CV] event, composite of all-cause mortality/non-fatal MI/ non-fatal ischemic stroke, all-cause mortality);

- To evaluate the safety and tolerability of alirocumab throughout the study;
- To evaluate the development of anti-alirocumab antibodies;
- To evaluate the effect of alirocumab on low-density lipoprotein cholesterol (LDL-C), apolipoprotein B (Apo B), and non-high-density lipoprotein cholesterol (non-HDL-C).

A Clinical Events Committee (CEC) is established to adjudicate, in a blinded fashion, events corresponding to the primary and secondary cardiovascular endpoints as well as all causes of death.

1.3 DETERMINATION OF SAMPLE SIZE

The sample size calculations are based on the primary efficacy variable, with the following assumptions:

- Primary efficacy endpoint is the time from randomization to the date of first occurrence of one of the following clinical events, as determined by the CEC: CHD death, any non-fatal MI, fatal or non-fatal ischemic stroke, or unstable angina requiring hospitalization (with new high-risk electrocardiogram (ECG) findings and contemporary evidence of angiographically significant coronary disease). Based on PROVE-IT results (1) and considering adjustment based on the study design and endpoints definition, the following Kaplan-Meier probabilities of event in the placebo group have been assumed: 3.8% at 12 months, 6.4% at 24 months, 9.0% at 36 months, and 11.4% at 48 months. Probabilities of an event at other time points are estimated using a piece-wise exponential model. The primary efficacy endpoint will be analyzed on an intent-to-treat basis (all randomized patients, including those who discontinue study medication are followed for any efficacy event until the CSED).
- Treatment hazard ratio of 0.85 (corresponding to a 15% hazard risk reduction for the test group relative to placebo), which is assumed to be constant over time;
- A log-rank test at an overall 1-sided 2.5% significance level with 90% power;

- Two interim analyses, according to a group sequential design, using for efficacy Gamma (-22) α-spending function, and for futility a Gamma (-5) β-spending function. Non-binding spending functions are used. See Section 4 for interim analysis details;
- One percent lost-to-follow-up prior to a primary efficacy endpoint at 24 months in both arms;
- Enrolment rate: sample size of 18 000 patients enrolled in 1400 sites (all sites were expected to be activated over a 12 months period). Table 1 below describes the expected enrolment rate per month; these assumptions were based on internal experience to enroll this patient population.

Table 1 - Enrolment rate assumptions per month

Month	1 to 3	4	5	6	7	8	9	10	11	12 to 40
No. patients per month	32	64	80	104	160	240	320	420	520	560
Cumulative no. patients	96	160	240	344	504	744	1064	1484	2004	2564 at M12, 18 000 at M40

Based on the above assumptions, 1613 events are needed for 90% power. In order to achieve the 1613 targeted events, 18 000 patients (9000 per group) were initially planned to be randomized, over a period of about 40 months. However taking into account the local situation in China (regulatory requirement to randomize at least 600 patients, and anticipated delay in study start in China), the total number of patients randomized may be increased to approximately 18 600 patients (~9300 per group). At the end of the study, the overall population will include approximately 18 000 randomized patients who have either died or have been followed for a minimum of 24 months, possibly supplemented with an additional subset of Chinese patients (~600) who may be followed for less than 24 months.

(electronic 1.0)

2 STUDY PLAN

The following figure presents graphically the study design:

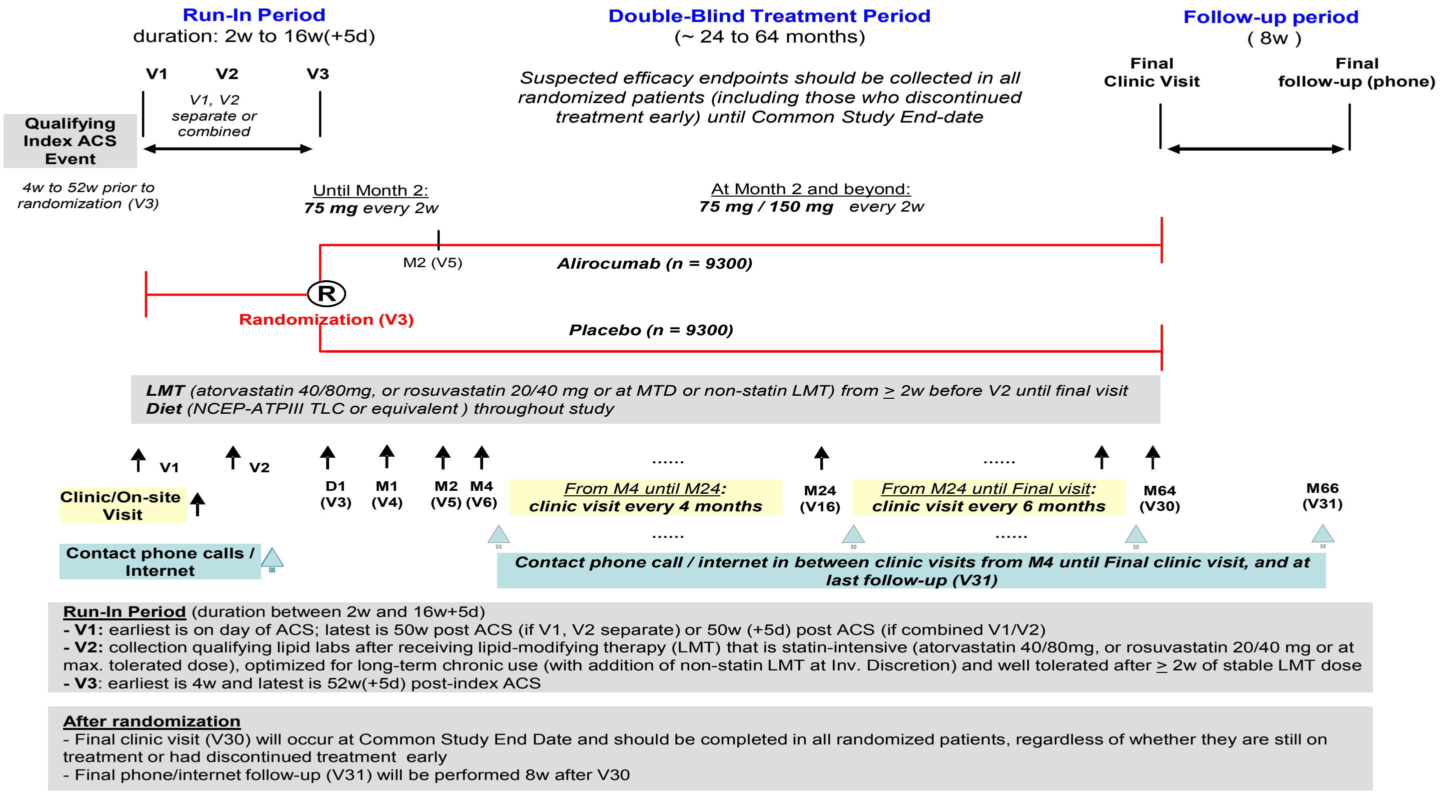

ACS: acute coronary syndrome, LMT: lipid-modifying therapy, MTD: maximal tolerated dose

The study flow chart is detailed in the protocol.

2.1 MODIFICATIONS TO THE STATISTICAL SECTION OF THE PROTOCOL

This section summarizes major changes to the protocol statistical section with emphasis on changes made after study start (after the first patient was enrolled).

The protocol history table below gives the timing, rationale, and key details of major changes to the protocol statistical section.

The first patient was randomized on 02 November 2012.

Table 2 - Protocol amendment statistical changes

Amendment Number	Date approved	Rationale	Description of statistical changes
6	05-Dec-2013	Clarification of definition of secondary endpoints in the protocol	The secondary endpoint of “Any hospitalization for unanticipated coronary revascularization procedure” was replaced with “Any ischemia-driven coronary revascularization procedure”
6	05-Dec-2013	Exclusion criteria in the protocol modified to allow inclusion of patients with a qualifying index ACS event occurring more than 16 weeks and less than 52 weeks prior to randomization	“Time from ACS event to randomization” in categorical variable (eg, 4-24 weeks, >24 weeks) is added as a subgroup factor in the Subgroups analyses of the primary efficacy endpoint
6	05-Dec-2013	The endpoint “Cardiovascular events of interest (other than efficacy endpoints)” has been added in the protocol.	The analysis of cardiovascular events of interest (other than efficacy endpoints) is added.
8	16-Apr-2015	Sample size may be increased up to approximately 18 600 patients to allow the inclusion of 600 Chinese patients because of the local delayed study start in China, and definition of the CSED updated	CSED definition modified to “when 1613 patients have experienced at least one primary efficacy event or 24 months after the closing of randomization for all countries except China, whichever comes last”
8	16-Apr-2015	Neurologic events (including neurocognitive events) are added as AESI	Neurologic events (including neurocognitive events) added in the safety analysis

ACS = acute coronary syndrome; AESI = adverse event of special interest; CSED = common study end date

2.2 STATISTICAL MODIFICATIONS MADE IN THE STATISTICAL ANALYSIS PLAN

The statistical analysis plan history table below gives the timing, rationale, and key details for major changes to the statistical analysis features in the statistical analysis plan compared to the protocol amendment 8 (version currently in effect at the time of this plan).

Changes already incorporated in a protocol amendment are listed only in Table 2.

The first patient was enrolled on 02 November 2012. The first interim analysis is planned for March/April 2016.

Table 3 - Statistical analysis plan statistical changes

SAP version number	Date approved	Rationale	Description of statistical changes
1	This version	Imputation method not planned for missing lipid values	For patients without lipid values in the time window analyzed, multiple imputation will be used with different imputation strategies depending on the time of those missing values (during the treatment period or after treatment discontinuation) (see Section 3.4.7.4.1)
1	This version	End of analysis period for the analysis of TEAEs for patients with 2 consecutives low LDL-C did not take into account down-titrattions from 150 mg to 75 mg	In the specific analysis of TEAEs for the subgroup of patients with two consecutive LDL-C <25 mg/dL (respectively 15 mg/dL) within the alirocumab treatment group, the upper limit of the analysis period for patients down-titrated from 150 mg to 75 mg will end at the date of last injection of 150 mg +70 days (see Section 3.4.5.1).
1	This version	Selection of patients with 2 consecutive LDL-C <25 mg/dL (respectively 15 mg/dL) modified consistently with the other studies of the program.	The patients will be considered as having 2 consecutive LDL-C <25 mg/dL (respectively 15 mg/dL) if these values are spaced out by at least 21 days (see Section 3.1.5.4).

SAP = statistical analysis plan; TEAE = treatment-emergent adverse event;

3 STATISTICAL AND ANALYTICAL PROCEDURES

3.1 ANALYSIS ENDPOINTS

3.1.1 Demographic and baseline characteristics

Unless otherwise specified, the baseline value is defined as the last available value obtained up to the first double-blind IMP injection.

For patients randomized and not treated, the baseline value will be the last available value on or before the day of randomization.

All baseline safety and efficacy parameters (apart from those listed below) will be presented along with the summary statistics in the safety and efficacy sections (Section 3.4.5 and Section 3.4.4).

Demographic characteristics

Demographic variables are:

- Age in years (quantitative and qualitative variable: <65, ≥65 to <75, and ≥75 years; and <65, ≥65 years);
- Gender (Male, Female);
- Race (White, Black or African American, Asian, American Indian or Alaska Native, Native Hawaiian or other Pacific Island, other);
- Ethnicity (Hispanic or Latino, Not Hispanic or Latino);
- Region (North America, South America, Western Europe, Eastern Europe, Asia, Rest of the world as defined in Section 3.5.6).

Medical or surgical history

This information will be coded using the version of Medical Dictionary for Regulatory Activities (MedDRA) currently in effect at Sanofi at the time of database lock.

Medical or surgical history includes:

- Medical history of allergies;
- Patient's family medical allergic history;
- Other relevant medical or surgical history;
- Smoking habits;
- Alcohol habits.

Medical history of specific interest includes:

- Coronary artery disease history prior to the index ACS event;
- Cardiovascular risk factors including:
  - Dyslipidemia;
  - Hypertension;
  - Family history of coronary artery disease;
  - Type 1 or Type 2 diabetes mellitus.
- Other cardiovascular disease, including:
  - Congestive heart failure;
  - Peripheral arterial disease;
  - Cerebrovascular disease (carotid endarterectomy/carotid stenting, prior stroke, transient ischemic attack).

In addition, the status of diabetes mellitus at baseline will be derived using the following definition:

- Type 1 or Type 2 diabetes reported in medical history or as an adverse event before baseline (ie, before the first IMP intake or randomization for non-treated patients) (using company MedDRA query [CMQ] "Type 1 or Type 2 diabetes" as detailed in Appendix B, Table 10); or
- HbA_{1c} ≥6.5% at baseline (V3) (or at V1 if V3 is not available); or
- 2 values of fasting blood glucose ≥126 mg/dL (7.0 mmol/L) (at V1 and V3); or
- Use of anti-diabetic medication before baseline (in case a partial start date for a given medication precludes determining whether it started prior or after baseline, the diabetes status will not consider anti-diabetic medications for the concerned patients).

Disease characteristics at baseline

Specific disease history includes:

- Information on index qualifying ACS event: number (%) of patients with elevated cardiac biomarkers (troponin I or T, or CKMB); with resting ECG changes consistent with ischemia or infarction (ST depression, ST elevation, T wave inversion, pathological Q waves, new tall R wave) and additional evidence of obstructive coronary disease (evidence of myocardial or infarction by perfusion imaging, regional wall motion abnormality, epicardial coronary artery stenosis ≥70%, need for revascularization [percutaneous coronary intervention (PCI) or coronary artery bypass graft surgery (CABG)] related to index ACS event);

- The index ACS subtype:
  - STEMI: elevated cardiac biomarkers (Troponin I or T, and/or CKMB) and new or presumed new ST elevation
  - NSTEMI: elevated cardiac biomarkers (Troponin I or T, and/or CKMB) and No new or presumed new ST elevation
  - UA: No Troponin I or T elevated, and No CKMB elevated, and "Resting ECG changed consistent with ischemia or infarction AND additional evidence of obstructive coronary disease" (ie, any combination of responses to questions "B" on the electronic case report form [e-CRF])
- Time from index ACS event to randomization (in weeks and in months), quantitatively and in category <2, ≥2 to <4, ≥4 to <6, ≥ 6 months
- Revascularization procedure associated with the index ACS event (PCI or CABG)
- Time from the revascularization procedure to randomization (in weeks and in months)
- New cardiovascular events occurring during the run-in period, selected using a list of preferred terms (PTs) from CMQ or standardized MedDRA query (SMQ).

Other baseline characteristics

Other baseline characteristics include weight in kilograms (quantitative variable), and body mass index (BMI) in kg/m^2 (quantitative and qualitative variable: <30, ≥30).

Lipid parameters, HbA_{1c} (quantitative and qualitative variable: <5.7 %, ≥5.7 to <6.5%, ≥6.5%) and hs-CRP (quantitative and qualitative variable: <2, ≥2 mg/L) at baseline will be also summarized by treatment group. Lipid parameters are total cholesterol (TC), LDL-C, non HDL-C, HDL-C, fasting triglycerides (TG), Apo A-1, Apo B, and lipoprotein (a) (Lp (a)).

For lipid parameters, both quantitative and qualitative variables will be considered, with the following categories:

- LDL-C: <70, ≥70 to <80, ≥80 to <100, ≥100 to <130, ≥130 to <160, ≥160 to <190, ≥190 mg/dL (ie, <1.81, ≥1.81 to <2.07, ≥2.07 to <2.59, ≥2.59 to <3.37, ≥3.37 to <4.14, ≥4.14 to <4.91, ≥4.91 mmol/L)
- HDL-C: <40, ≥40 mg/dL (ie, <1.04, ≥1.04 mmol/L),
- Non-HDL-C: <100, ≥100 to <110, ≥110 to <130, ≥130 to <160, ≥160 to <190, ≥190 to <220, ≥220 mg/dL (ie, <2.59, ≥2.59 to 2.84, ≥2.84 to <3.37, ≥3.37 to <4.14, ≥4.14 to <4.91, ≥4.91 to 5.69, ≥5.69 mmol/L)
- Fasting TG: <150, ≥150 to <200, ≥200 mg/dL (ie, <1.7, ≥1.7 to <2.3, ≥2.3 mmol/L), category ≥150 mg/dL (ie, ≥1.7 mmol/L [mixed dyslipidemia]) will be also displayed,
- Lp (a): <30, ≥30 to <50, ≥50 mg/dL (ie, <0.3, ≥0.3 to <0.5, ≥0.5g/L), category ≥30 mg/dL (ie, ≥0.3 g/L) will be also displayed
- Apo B: <75, ≥75 to <90, ≥90 mg/dL (ie, <0.75, ≥0.75 to <0.9, ≥0.9 g/L)

In addition the number (%) of patients not adequately controlled at baseline as per protocol definition will be described:

- LDL-C ≥70 mg/dL (1.81 mmol/L) or Apo B ≥80 mg/dL (0.8 g/L) or non-HDL-C ≥100 mg/dL (2.59 mmol/L)
- LDL-C ≥70 mg/dL (1.81 mmol/L)
- Apo B ≥80 mg/dL (0.8 g/L)
- Non-HDL-C ≥100 mg/dL (2.59 mmol/L)

Any technical details related to computation, dates, and imputation for missing dates are described in Section 3.5.

3.1.2 Prior or concomitant medications

All LMTs taken within 1 month before screening visit V1 and until the end of the study, are to be reported in 1 of the following specific case report form pages:

- Previous and concomitant statin drugs;
- Previous and concomitant medications lipid lowering drugs (other than statins);

Patients on chronic use of statin (ie, on any statin for at least 3 months prior to the index ACS event) and the reasons for any modification in the statin regimen post-randomization are to be reported on the following specific page:

- Additional statin information

Other concomitant medications taken since informed consent, including cardiovascular medications are to be reported on the following specific page:

- Concomitant medications (all other than statin and other than lipid lowering drugs).

All medications will be coded using the World Health Organization-Drug Dictionary (WHO-DD) using the version currently in effect at Sanofi at the time of database lock.

- Prior medications are those the patient used within 1 month before screening visit V1 and prior to first investigational medicinal product (IMP) injection. Prior medications can be discontinued before first administration or can be ongoing during treatment phase;
- Concomitant medications are any treatments received by the patient concomitantly to the IMP, from first IMP injection to the last double-blind injection +70 days. A given medication can be classified both as a prior medication and as a concomitant medication. Concomitant medications do not include medications started during the post-treatment period (as defined in the observation period in Section 3.1.4);
- Post-treatment medications are those the patient took in the period starting the day after the concomitant medication period up to the end of the study.

For patients randomized but not treated, medications will be categorized as prior medications or post-treatment medications according to the intake dates in relation to the date of randomization.

The following medications of specific interest will be also selected using specific coding's list:

- Aspirin or oral ADP receptor antagonists
- Injectable anticoagulants (UFH or LMWH or Bivalirudin or Selective Factor Xa inhibitor)
- Thrombolytic
- Specific oral anticoagulant
- Anti-diabetic drugs (insulin, oral anti-diabetic, other non-oral anti-diabetic)
- ACE-inhibitor or angiotensin receptor blocker
- Beta blocker
- Calcium channel blocker
- Diuretics
- Nitrates
- NSAIDs (excluding aspirin)

Any technical details related to computation, dates, imputation for missing dates are described in Section 3.5.

3.1.3 Efficacy endpoints

3.1.3.1 Primary efficacy endpoint(s)

The primary efficacy endpoint is the time from randomization to first occurrence of 1 of the following clinical events, as determined by the CEC:

- CHD death (including "undetermined causes of death" as per the CEC);
- Any non-fatal MI;
- Fatal and non-fatal ischemic stroke (including "stroke not otherwise specified" as per the CEC);
- Unstable angina requiring hospitalization.

The rules to determine the components and the date of the event that will be considered in the analyses of the primary efficacy endpoint are detailed in Appendix C.

If none of these events is observed at the time of the analysis cut-off date (final or interim, depending on the timing of the analysis, see Section 3.4.4.1 and Section 4 for details), the patient will be right-censored at the date of last contact when information on efficacy endpoints (presence or absence) has been retrieved, or at the date of death, or at the cut-off date/CSED, whichever comes first.

The last information on efficacy endpoint (presence or absence)" will be the latest date among:

- The date of last visit performed with "endpoints events" e-CRF page completed.
- The "Date of last information on efficacy endpoint (presence or absence)" reported in the visit e-CRF pages during the course of the study.
- The "Date of last information on efficacy endpoint (presence or absence)" reported at the end of the study for all patients (this information can also be completed during the course of the study for "lost-to-follow-up" patients, patients who discontinued the follow-up, and patients who died).

In case of no information on the presence or absence of efficacy endpoint, nor on death at time of database extraction for the interim analyses, the censoring date will be the randomization date (eg, for patients randomized but having not reached the Month 1 visit yet).

Handling of missing or incomplete dates

In the exceptional circumstances when the exact date of occurrence of the outcome event has not been established (day and/or month missing), the date will be imputed as follows:

Table 4 - Imputation of incomplete date of events composing the primary efficacy outcome

Type of event	Imputed date
	**Only the day is missing**
Death	The latter of the last contact date + 1 day and the 1st day of the month.
Other primary endpoint component (CHD event or ischemic stroke)	The latter of the randomization date and the 1st day of the month.
	**Day and month are missing**
Death	The latter of the last contact date + 1 day and January 1st of the year.
Other primary endpoint component (CHD event or ischemic stroke)	The latter of the randomization date and January 1st of the year.

CHD = coronary heart disease

3.1.3.2 Secondary efficacy endpoint(s)

Clinical events assessed in the analyses are those determined by the CEC. Events suspected by the investigator but not confirmed by the CEC will not be part of the outcomes. CHD death outcome will include deaths for causes that could not be determined by the CEC (adjudicated as "Undetermined cause of death"). Similarly, fatal and non-fatal ischemic stroke outcomes will include fatal and non-fatal strokes for causes that couldn't be determined by the CEC (adjudicated as "Stroke not otherwise specified").

Rules for censoring date and for imputation of incomplete dates of events will be the same as for the primary efficacy endpoint, for all secondary efficacy endpoints with the exception of the analyses of the time to CHD death and of the time to all-cause mortality for which the censoring date will be defined as the earliest date among the "date of last known alive or Date of death" and the cut-off date/CSED.

The "date of last known alive or Date of death" will be the latest date among:

- The date of last visit performed with "endpoints events" e-CRF page completed;
- The "Date of last known alive or Date of death" reported in the visit e-CRF pages during the course of the study;
- The "Date of last known alive or Date of death" reported at the end of the study for all patients (this information can also be completed during the course of the study for "lost-to-follow-up" patients, patients who discontinued the follow-up and patients who died);
- For interim analyses, the latest date among these dates, the last IMP injection date, the date of adverse events and the date of laboratory samples will be considered.

In case at time of database extraction for the interim analyses, only the randomization date is available, then the censoring date will be the randomization date.

3.1.3.2.1 Main secondary endpoint(s)

- Time from randomization to first occurrence of any CHD event (major CHD event, unstable angina requiring hospitalization, ischemia-driven coronary revascularization procedure);
- Time from randomization to first occurrence of any major CHD event (CHD death, nonfatal MI);
- Time from randomization to first occurrence of any CV event (any non-fatal CHD event, any CV death, and non-fatal ischemic stroke);
- Time from randomization to first occurrence of all-cause mortality, non-fatal MI, non-fatal ischemic stroke;
- Time from randomization to death (all-cause mortality).

3.1.3.2.2 Other secondary efficacy endpoint(s)

- Component of the primary endpoint considered individually:
  - Time from randomization to CHD death;
  - Time from randomization to first occurrence of any non-fatal MI;
  - Time from randomization to first occurrence of fatal or any non-fatal ischemic stroke;
  - Time from randomization to first occurrence of any unstable angina requiring hospitalization.

- Time from randomization to first occurrence of any ischemia-driven coronary revascularization procedure;
- Time from randomization to first occurrence of any congestive heart failure requiring hospitalization.

3.1.4 Safety endpoints

Following injections are considered as double-blind IMP injections:

- For patients randomized in the placebo group: any injection from double-blind kits;
- For patients randomized in the alirocumab group: any injection from double-blind kits excepted placebo injections given to maintain the blind in case of 2 consecutive LDL-C values <15 mg/dL.

Of note potential additional training injections after randomization using training injection kits will not be taken into account.

The period of safety observation starts from the time when the patient gives informed consent and is divided into three periods:

- PRE-TREATMENT period: defined from the signed informed consent up to the first dose of double-blind IMP injection;
- Treatment-emergent adverse event (TEAE) period: defined as the time from the first dose of double-blind IMP injection to the last dose of double-blind IMP injection +70 days (10 weeks), (as residual effect of treatment is expected until 10 weeks after the stop of double-blind IMP).

  The TEAE period will include:

  - The TREATMENT period defined as the time from the first dose of double-blind IMP up to the day of last dose of double-blind IMP injection +21 days, as serum concentration of alirocumab >10 µg/mL is expected for approximately 21 days following administration of 150 mg, and because throughout the previous studies it was observed that when alirocumab concentrations declined below this concentration, decrease in effect on LDL-C is observed.
  - The RESIDUAL TREATMENT defined as the time from the day of last dose of double-blind IMP injection +22 days up to the day of last dose of double-blind IMP injection +70 days (10 weeks).
- POST-TREATMENT period: defined as the time starting the day after the end of the TEAE period.

3.1.4.1 Adverse events variables

Occurrence of adverse events (including serious adverse events [SAEs], and adverse events of special interest [AESIs]) are recorded from the time of signed informed consent until the end of study.

All adverse events will be coded to a “Lowest Level Term (LLT)”, “Preferred Term (PT)”, “High Level Term (HLT)”, “High Level Group Term (HLGT)”, and associated primary “System Organ Class (SOC)” using the version of MedDRA currently in effect at Sanofi at the time of the database lock.

Adverse event observation periods

- Pre-treatment adverse events are adverse events that developed or worsened or became serious during the pre-treatment period;
- Treatment-emergent adverse events are adverse events that developed or worsened or became serious during the treatment-emergent adverse event period;
- Post-treatment adverse events are adverse events that developed or worsened or became serious during the post-treatment period.

Adverse events of special interest

Adverse events of special interest (AESIs) are adverse events (serious or non-serious) that need to be monitored, documented, and managed in a pre-specified manner described in the protocol. In this study, AESI are the following (their complete descriptions are provided in the protocol):

- General allergic events, selected using SMQ “hypersensitivity” (broad and narrow) excluding the following preferred terms linked to local injection site reactions (“infusion site dermatitis”, “infusion site hypersensitivity”, “infusion site rash”, “infusion site urticaria”, “injection site dermatitis”, “injection site hypersensitivity”, “injection site rash”, “injection site urticaria”, and “injection site vasculitis”)
- Local injection site reactions, selected using e-CRF specific tick box on the adverse event page
- ALT ≥3 ULN (if baseline ALT <ULN) or ALT ≥2 times the baseline value (if baseline ALT ≥ULN), selected using laboratory data
- Hemolytic anemia, selected using e-CRF specific tick box on the adverse event page and confirmed final diagnosis provided in the adverse event complementary form
- Neurologic events selected using a CMQ, based on SMQs “demyelination” (broad and narrow), “peripheral neuropathy” (broad and narrow), and “Guillain-Barre syndrome” (broad and narrow) excluding the following preferred terms “acute respiratory distress syndrome”, “asthenia”, “respiratory arrest” and “respiratory failure” and including selected PTs from SMQ "optic nerve disorders" (see Appendix B Table 11 for the list of terms)

- Neurocognitive events:
  - Selected using a CMQ, based on the following 5 HLGTs: "deliria (including confusion)", "cognitive and attention disorders and disturbances", "dementia and amnestic conditions", "disturbances in thinking and perception", and "mental impairment disorders"
  - A second grouping of terms for neurocognitive events was defined based on Regulatory Agency request (see Appendix B Table 12 for the list of terms)
- Overdose of IMP (symptomatic or asymptomatic), selected using appropriate MedDRA codes and the tick box "Overdose with IMP" in the adverse event complementary e-CRF form
- Pregnancy (including partner of a randomized male subject) selected using appropriate MedDRA codes

In addition the additional grouping of events will be provided:

- Hepatic disorder events using SMQ "Hepatic disorder"
- Diabetes mellitus or diabetic complications using HLGT "diabetes complications" (including PTs pertaining to the secondary SOC included in the HLGT), HLT "diabetes mellitus", and HLT "carbohydrate tolerance analyses (incl diabetes)" excluding PTs "blood glucose decreased" and "Glycosylated haemoglobin decreased" and including the PTs "hyperglycaemia", "Hyperglycaemic unconsciousness" and "Hyperglycaemic seizure" from the HLT "Hyperglyceamic conditions NEC"
- Cataract using HLT "Cataract conditions"

3.1.4.2 Deaths

The deaths observation period are per the observation periods defined below.

- Deaths from first IMP injection until CSED:
  - Treatment-emergent deaths: deaths occurring during the TEAE period
  - Post-treatment emergent deaths
- Deaths post-CSED

3.1.4.3 Laboratory safety variables

Clinical laboratory data consists of blood analysis, including hematology, clinical chemistry, and urinalysis. Clinical laboratory values after conversion will be analyzed into standard international units. International units will be used in all listings and tables. Clinical laboratory values converted into conventional (US) units will be also available in the database. Analyses can be provided upon request.

Blood samples for clinical laboratories will be taken as described in the study flow chart in the protocol.

The laboratory parameters will be classified as follows:

- Hematology
  - **Red blood cells and platelets**: hemoglobin, hematocrit, red blood cell count, platelet count;
  - **White blood cells:** white blood cell count, neutrophils, lymphocytes, monocytes, basophils, eosinophils.
- Clinical chemistry
  - **Metabolism:** fasting plasma glucose, total protein, albumin, creatine phosphokinase (CPK);
  - **Electrolytes**: sodium, potassium, chloride, calcium, phosphorus, bicarbonate;
  - **Renal function**: creatinine, eGFR, blood urea nitrogen, uric acid;
  - **Liver function**: alanine aminotransferase (ALT), aspartate aminotransferase (AST), alkaline phosphatase (ALP), gamma glutamyl transferase (γGT), lactate hydrogenase (LDH), total bilirubin, and in case of total bilirubin values above the normal range, must include direct and indirect bilirubin (used for describing individual cases only);
  - **Hepatitis screen:** anti-hepatitis-C antibody.

Technical formulas are described in Section 3.5.1.

3.1.4.4 Vital signs variables

Vital signs include: weight, heart rate, systolic and diastolic blood pressure in sitting position.

3.1.4.5 Electrocardiogram variables

Electrocardiograms were recorded automatically by the device at the Investigator site.

Electrocardiogram assessments will be described as normal or abnormal.

3.1.5 Other endpoints

Other assessment endpoints defined below are exploratory.

3.1.5.1 Lipid parameters

The lipid parameters include values (in conventional [US] and international units), percent change from baseline, and/or when appropriate absolute change from baseline (in conventional and international units) over time for the following parameters: LDL-C, TC, HDL-C, fasting TGs, non-HDL-C, Apo A-1, Apo B, ratio Apo B/Apo A-1, Lp (a), ratio TC/HDL.

All these parameters are measured or calculated by a central laboratory, for both scheduled and unscheduled time points. For LDL-C analysis, both calculated and measured LDL-C values will be taken into account. In case both calculated and measured LDL-C are provided for the same sampling, the measured LDL-C will be considered. Calculated LDL-C is obtained using the Friedewald formula. Non-HDL-C is calculated by subtracting HDL-C from the TC.

Unless otherwise specified, all central measurements (scheduled or unscheduled, fasting or not fasting) may be used to provide a value for each timepoint, even if assessed after treatment discontinuation (intent-to-treat [ITT] approach). The analysis windows used to allocate a measurement to a time point are defined in Section 3.5.4. For TG, only fasting measurements will be used. Measurements with missing fasting status will be excluded from the analyses.

For all time points post-baseline, the value used for the analyses at a given time point (eg, at Month 24) is the value obtained within the corresponding analysis window. The baseline value is the last available measurement obtained up to the date and time of the first double-blind IMP injection. For patients randomized and not treated, the baseline value is defined as the last available value obtained up to the date and time of randomization.

Percent changes from baseline to a timepoint are defined as: 100 x (parameter value at the time point – parameter value at baseline) / parameter value at baseline.

Data handling conventions for other endpoints are described in Section 3.5.

3.1.5.2 hs-CRP

The percent change in hs-CRP from baseline over time is defined using same definitions and rules as for LDL-C, when applicable (see section above). hs-CRP values greater or equal to 10 mg/L will be excluded from analyses in a second approach, since these are suggestive of concurrent infections, MI, or other events provoking an acute phase response (2).

PCSA criteria for hs-CRP are defined in Appendix A.

3.1.5.3 HbA_{1C}

The absolute change in HbA_{1c} (%) from baseline over time: same definitions and rules as for LDL-C (see Section 3.1.5.1).

3.1.5.4 Patients with LDL-C <25 mg/dL (0.65 mmol/L)

The assessment will include:

- The proportion of patients with two consecutive results, spaced out by at least 21 days, of LDL-C <25 mg/dL (<0.65 mmol/L) (respectively LDL-C <15 mg/dL, ie, <0.39 mmol/L) during the double-blind treatment period
- The time to the first LDL-C <25 mg/dL (respectively LDL-C <15 mg/dL) for these patients.

In case both calculated and measured LDL-C are provided for the same sampling, the measured LDL-C will be considered.

3.1.5.5 Anti-alirocumab antibodies assessed throughout the study.

Anti-alirocumab antibodies (ADAs) are assessed at Visit 3 (Month 0), Visit 5 (Month 2), Visit 6 (Month 4), and Visit 10 (Month 12) for the first year, then every year and at the final on-treatment visit (CSED visit for completers, or early end of treatment visit for patients who discontinued the treatment).

ADA measurements will be assigned to analysis windows as defined in Section 3.5.4.

The following variables will be described:

- ADA response (Positive or Negative). For ADA positive:
  - Titer levels
  - Neutralizing status (Positive or Negative)
- Pre-existing positive ADA defined as patients with positive ADA response at baseline with less than 4-fold increase in titer in the post-baseline period
- Treatment-emergent positive ADA response defined as 1) Patients with no ADA positive response at baseline but with any positive response in the post-baseline period (up to follow-up visit) or 2) Patients with a positive ADA response at baseline and at least a 4-fold increase in titer in the post-baseline period (up to follow-up visit). For treatment-emergent positive ADA, the following categories for ADA duration will be applied:
  - A persistent positive response is a treatment-emergent ADA positive response detected in at least 2 consecutive post-baseline samples separated by at least a 16-week period
  - An indeterminate duration positive response is defined as ADA present only at the last sampling time point
  - A transient positive response is defined as any treatment-emergent positive ADA response that is neither considered persistent nor indeterminate

In addition, potential ADA samples that are to collected after follow-up visit for patients with titer >240 at early end of treatment or CSED visit will be listed.

3.1.5.6 Quality-of-life parameters

EQ-5D™ is a standardized and generic instrument for measuring the health status and health related quality of life for clinical and economic assessment (3). EQ-5D instrument includes 5 items corresponding to the following dimensions: mobility, self-care, usual activities, pain/discomfort, anxiety/depression (Appendix D). Each item can take 1 of 3 responses: (1.) "no problem", (2.) "some problems", and (3.) "severe problems". Overall health status is defined as a 5-digit number and will be converted into a standard utility score ranging between -0.594 (representing severe problems) and 1 (representing no problem): the single index utility score, using a regression model (4) (Appendix E). If response to one or more dimension is missing, the utility score will be missing.

Quality of life parameters include response to each EQ-5D items and change in utility score over time from baseline.

3.1.5.7 Cardiovascular events of interest (other than efficacy endpoints)

Cardiovascular events of interest (other than efficacy endpoints) include clinically significant complications or procedures (not planned at the time of randomization), related to peripheral arterial disease and venous thromboembolic events as listed below:

- Venous thromboembolic events of interest:
  - Deep vein thrombosis (DVT)
  - Pulmonary embolism (PE)
- Lower extremity peripheral arterial disease events of interest:
  - Peripheral lower limb revascularization (endovascular revascularization, surgical revascularization)
  - Critical limb ischemia (including ischemic imputation of lower limb for the event)

The following variables will be analyzed:

- Time from randomization to first occurrence of any other CV events of interest (venous thromboembolic events or lower extremity peripheral arterial disease events)
- Time from randomization to first occurrence of any venous thromboembolic events (DVT or PE)
- Time from randomization to first occurrence of any lower extremity peripheral arterial disease events (peripheral lower limb revascularization or critical limb ischemia)

3.1.6 Pharmacokinetic variables

Not applicable.

3.1.7 Pharmacogenomics endpoints

Pharmacogenetics endpoints and analyses will be detailed in a separate SAP.

3.2 DISPOSITION OF PATIENTS

This section describes patient disposition for both patient study status and the patient analysis populations.

Screened patients are defined as any patient who met the ACS inclusion criteria and signed the informed consent (ie, patients entering the run-in phase).

Randomized patients consist of all screened patients, with a double-blind treatment kit number allocated and recorded in the interactive voice response system (IVRS)/ interactive web response system (IWRS) database, regardless of whether the treatment kit was used or not. Patients treated without being randomized or treated with a double-blind treatment kit before the randomization will not be considered as randomized and will not be included in any analysis population.

For patient study status, the total number of patients in each of the following categories will be presented in the clinical study report using a flowchart diagram or summary table:

- Screened patients;
- Screened failure patients and reasons for screen failure;
- Non-randomized but treated patients, if any;
- Randomized patients;
- Randomized but not treated patients and reason for not being treated;
- Randomized and treated patients;
- Patients who completed the double-blind study treatment period as per protocol (as per e-CRF end-of-treatment form);
- Patients who did not complete the double-blind study treatment period as per protocol (as per e-CRF end-of-treatment form);
- Patients who discontinued the double-blind study treatment by main reason for permanent treatment discontinuation (as per e-CRF end-of-treatment form);
- Status at last study contact.

For all categories of patients (except for the screened and non-randomized categories) percentages will be calculated using the number of randomized patients as the denominator.

Reasons for treatment discontinuation will be supplied in tables giving numbers and percentages by treatment group. For blinded discontinuation due to low LDL-C level (patients who switched to placebo in blinded manner), the reason reported by the investigator will be reclassified as “2 consecutive LDL-C <15mg/dL (<0.39 mmol/L)”.

Number (%) of patients who discontinued the follow-up for CV events will be summarized over time. The main reason for study discontinuation will be summarized overall and according to whether or not the patients had a primary efficacy endpoint confirmed by CEC prior study discontinuation.

A patient will be considered as having discontinued the follow-up for CV events if the date of the last information on efficacy endpoints (presence or absence) is before the common study end date.

Kaplan-Meier plots/estimates of the cumulative incidence of premature IMP treatment discontinuation due to any reason, or due to adverse event will be provided on randomized population. Not treated patient will be considered with event at Day 1 (day of randomization). All completers will be considered as right-censored observations. Time to premature IMP treatment discontinuation and censoring time will be defined as: Date of last IMP injection – Date of randomization + 14 days.

All major deviations potentially impacting efficacy analyses, randomization, and drug-dispensing irregularities, and other major deviations will be summarized in tables giving numbers and percentages of deviations by treatment group. These deviations are listed in the data review and surveillance plan.

Additionally, the following populations will be summarized by treatment group.

- Randomized population;
- Efficacy population: intent-to-treat (ITT) population;
- Safety population;
- Anti-alirocumab antibody population.

Definition of the study populations are provided in Section 3.3.

3.2.1 Randomization and drug dispensing irregularities

Randomization and drug-dispensing irregularities occur whenever:

1. A randomization is not in accordance with the protocol-defined randomization method, such as a) a patient is randomized based on an incorrect stratum, or b) a patient is randomized twice.

OR

2. A patient is dispensed an IMP kit not allocated by the protocol-defined randomization, such as a) a patient at any time in the study is dispensed a different treatment kit than as randomized (which may or may not contain the correct-as-randomized IMP), or b) a non-randomized patient is treated with IMP reserved for randomized patients.

Randomization and drug-dispensing irregularities will be monitored throughout the study and reviewed on an ongoing basis.

All randomization and drug-dispensing irregularities will be documented in the clinical study report. These irregularities will be summarized by treatment group on the randomized population. Non-randomized, treated patients will be described separately.

Randomization and drug-dispensing irregularities to be prospectively identified include but are not limited to:

Randomization and drug allocation irregularities
Kit dispensation without IVRS/IWRS transaction
Erroneous kit dispensation
Patient randomized twice
Stratification error
A kit allocated at Day 1 or any unscheduled replacement before the up-titration visit (it may be the Month 2 or the Month 4 visit) is administered to the patient after the up-titration visit[a]
A kit allocated at any visit or unscheduled replacement from the up-titration visit, is administered to the patient after the next scheduled reallocation visit[a]

a Only if dose received is different from the one expected as per IVRS/IWRS allocation

3.3 ANALYSIS POPULATIONS

Patients treated without or before being randomized will not be considered randomized and will not be included in any analysis populations. The safety experience and CV events of patients treated and not randomized will be reported separately.

Randomized population: includes all randomized patients as defined in Section 3.2.

For any patient randomized more than once, only the data associated with the first randomization will be used in any analysis population. The safety experience associated with any later randomization will be assessed separately.

3.3.1 Efficacy populations

3.3.1.1 Intent–to-treat population

The primary efficacy analysis population will be the intent-to-treat (ITT) population, consisting of all randomized patients. Patients in the ITT population will be analyzed according to the treatment group allocated by randomization.

3.3.2 Safety population

The Safety population considered for safety analyses will be the randomized patients who actually received at least 1 dose or part of a dose of the double-blind IMP injection. Patients will be

analyzed according to the treatment actually received (ie, as-treated treatment group, placebo or alirocumab).

In addition:

- Randomized patients for whom it is unclear whether they took the study medication will be included in the safety population in the treatment group as randomized;
- For patients receiving double-blind IMP injection from more than one treatment group during the trial (cases reported as protocol deviation), the treatment group used for as-treated analysis will be the one to which the patient was treated with the highest number of injections; in case of the same number of injections of each treatment is received, the as-treated treatment group will be the as-randomized group.

3.3.3 Anti-alirocumab antibody population

The ADA analysis will be performed on all randomized and treated patients (safety population) with an available ADA sample at Day 1 (baseline) and at least 1 available ADA sample post first double-blind IMP injection.

3.4 STATISTICAL METHODS

3.4.1 Demographics and baseline characteristics

Parameters described in Section 3.1.1 will be summarized by treatment group and overall using descriptive statistics.

Continuous data will be summarized using the number of available data, mean, standard deviation (SD), median, minimum, and maximum for each treatment group. First quartile (Q1) and third quartile (Q3) will be also provided for baseline lipid parameters, HbA_{1c}, and hs-CRP. Categorical and ordinal data will be summarized using the number and percentage of patients in each treatment group.

Unless otherwise specified, parameters will be summarized on the randomized population analyzed in the treatment group to which they were randomized. Similar analyses will be done on the safety population and will be included in the appendices if the size of the safety population is different (>10%) from the size of the randomized population for any treatment group. In the randomized population, parameters will also be summarized within each region.

All reported patient's medical and surgical history will be presented by primary SOC and HLT. The tables will be sorted by SOC internationally agreed order and decreasing frequency of HLT based on the overall incidence across treatment groups. In addition all medical history of specific interest will be presented by treatment group.

The diagnosis of diabetes mellitus at baseline (see also Section 3.1.1 for definition), as well as the source of diagnosis will be summarized in the randomized and safety populations by treatment group and overall, using the following mutually exclusive categories:

- From medical history or pre-treatment adverse events
- From anti-diabetic medications regardless of laboratory data (if no medical history of diabetes)
- From laboratory data only (if no medical history of diabetes and no anti-diabetic medications)

No specific description of the safety parameters will be provided at baseline. If relevant, the baseline values will be described along with each safety analysis.

3.4.2 Prior, concomitant or post-treatment medications

The prior, concomitant, and post-treatment medications will be presented for the randomized population.

Medications will be summarized by treatment group according to the WHO-DD dictionary, considering the first digit of the anatomical therapeutic chemical (ATC) class (anatomical category) and the first 3 digits of the ATC class (therapeutic category). All ATC codes corresponding to a medication will be summarized, and patients will be counted once in each ATC category (anatomical or therapeutic) linked to the medication. Therefore patients may be counted in several categories for the same medication.

The table for prior medications will be sorted by decreasing frequency of ATC followed by all other therapeutic classes based on the overall incidence across treatment groups. In case of equal frequency regarding ATCs (anatomical or therapeutic categories), alphabetical order will be used.

The tables for concomitant and post-treatment medications will be sorted by decreasing frequency of ATC followed by all other therapeutic classes based on the incidence in the alirocumab group. In case of equal frequency regarding ATCs (anatomical or therapeutic categories), alphabetical order will be used. In addition all medications of specific interest will be presented by treatment group.

The number and percentage of patients that took any statin for at least 3 months prior to the index ACS event (as reported on the e-CRF page “Additional statin information”) will be displayed by treatment group.

The background lipid modifying therapy regimen at randomization will be summarized using the following categories:

- High dose atorvastatin/rosuvastatin (defined as daily atorvastatin 40 to 80 mg, or rosuvastatin 20 to 40 mg)
- Low/moderate dose atorvastatin/rosuvastatin (defined as daily atorvastatin <40 mg, or rosuvastatin <20 mg)

- Statin other than atorvastatin or rosuvastatin, at any dose
- Only LMT other than statin
- No LMT

The reason for not being on high dose at randomization will be supplied in tables giving numbers and percentages by treatment group.

Details (ie, statin names, doses) for patients who had received at least 2 statins the day of randomization (if any) will be listed.

For atorvastatin and rosuvastatin, the dose (in mg) will be also displayed by treatment group.

- Atorvastatin daily dose in mg (10, 20, 40, 80, Other);
- Rosuvastatin daily dose in mg (5, 10, 20, 40, Other);

The LMT other than statins will be summarized by pre-specified categories, chemical class or therapeutic class, and standardized medication name.

In addition the number (%) of patients in the following background LMT categories at randomization will be displayed:

- Ezetimibe and high dose atorvastatin/rosuvastatin
- Ezetimibe and low/moderate dose atorvastatin/rosuvastatin
- Ezetimibe and statin other than atorvastatin or rosuvastatin
- Ezetimibe and other LMT other than statin
- Only ezetimibe

LMT (statins and other LMTs) used after randomization during the study will be summarized over time graphically by treatment group and LMTs intensity at randomization using the following mutually exclusive categories:

- High dose atorvastatin/rosuvastatin
- Low/moderate dose atorvastatin/rosuvastastin
- Statin other than atorvastatin or rosuvastatin
- Only LMT other than statin
- No LMT

The reason for first modification in statin regimen after randomization will be also described.

The use of ezetimibe after randomization during the study will be also summarized over time graphically by treatment group.

The number (%) of patients initiating ezetimibe after randomization will be also displayed according to background LMT status at randomization.

3.4.3 Extent of investigational medicinal product exposure and compliance

The extent of IMP exposure and compliance will be assessed and summarized by actual treatment received within the safety population.

Double-blind IMP kits contain the following:

- Placebo for the ones administered to patients randomized in the placebo group
- 75 or 150 mg of alirocumab

Placebo injections administered to patients randomized in the alirocumab following 2 consecutive LDL-C <15 mg/dL will not be considered as double-blind IMP injections in the statistical analyses.

3.4.3.1 Extent of investigational medicinal product exposure

The total exposure will be assessed by:

- Duration of IMP exposure in months defined as: (last dose of double-blind IMP injection date + 14 – first dose of double-blind IMP injection date) / 30.4375, regardless of intermittent discontinuations (see Section 3.5.3 for calculation in case of missing or incomplete data). Non-integer values will be rounded to one decimal place;
- The total number of double-blind IMP injections by patient;

In addition the duration of the observation period in months will be analyzed. The duration of observation period is defined as: (last contact date – randomization date+1)/30.4375. Non-integer values will be rounded to one decimal place.

These parameters will be summarized descriptively as a quantitative variable (number, mean, SD, median, minimum, and maximum). In addition, the durations of treatment exposure and observation period will be presented according to the following categories: <2, ≥2 to <6, ≥6 to <12, ≥12 to <24, ≥24 to <36, ≥36 to <48, ≥48 to <60, ≥60 months. The total number of double-blind IMP injections by patient will be presented similarly using the following categories: <4, ≥4 to <13, ≥13 to <26, ≥26 to <39, ≥39 to <52, ≥52 to <65, ≥65 to <78, ≥78 to <104, ≥104 to <120, ≥120.

Additionally, the cumulative exposure will be provided in patient years.

Titration

The following summaries will be provided in the alirocumab group:

- The number (%) of patients with an up-titration to 150 mg, overall and according to the time of up-titration (ie, Month 2 and Month 4)
- The number (%) of patients with an up-titration followed by a down-titration to 75 mg
- The number (%) of patients with a switch to placebo
- The number (%) of patients on 75 mg, 150 mg, placebo over time (intermittent discontinuations won't be taken into account)

Cumulative exposure in patient-year by dose level (75 mg, 150 mg, placebo) will be provided, not taking into account intermittent discontinuations.

3.4.3.2 Compliance

Compliance will be assessed using the injection frequency that will be defined for each patient as the average number of days between 2 injections, that is: (last dose date – first dose date) / (number of injections – 1).

Cases of overdose (ie, 2 or more injections from the double-blind treatment kit are administered in <7 calendar days) will be summarized by treatment group. More generally, dosing irregularities are defined in Section 3.2.1.

3.4.4 Analyses of efficacy endpoints

All efficacy analyses will be performed based on ITT approach that will include events occurring from randomization to the analysis cut-off date for interim analysis or CSED for the final analysis, even after the patient has discontinued the study treatment. Any CV endpoint events occurring after the cut-off date/CSED will not be included in the analyses, regardless of the adjudication status. These events, if any, will be reported in a listing separately.

3.4.4.1 Analysis of primary efficacy endpoint(s)

The analysis of the primary efficacy endpoint will be the comparison between the two treatments using a log-rank test stratified by region (North America, South America, Western Europe, Eastern Europe, Asian, Other). The randomization is stratified by country but since the number of events per individual country is expected to be low (about 50 countries), the analysis will be stratified according to a grouping of countries into regions.

This primary comparison will be the 1-sided test (at 0.0249 type 1 error for the final analysis) of the following hypotheses at the final analysis:

$$H_0\text{: HR} \geq 1 \text{ versus } H_1\text{: HR} < 1$$

The estimates of the HR and corresponding confidence interval (CI) at (1-2α)% level (α being the 1-sided significance level: α=0.0249 at final analysis, α=0.0001 at second interim analysis) will be provided using a Cox Proportional Hazard model stratified by region. The underlying assumption of proportional hazards for Cox model will be checked by visual inspection of Kaplan-Meier plots. If proportionality is not observed, sensitivity analyses will be performed. In particular, the results will be presented by yearly intervals: the number of events per 100 patient-years for each yearly interval will be provided for each treatment group as well as the ratio of the two event rates. In addition, between-treatment cumulative rate ratios based on the Kaplan-Meier estimates and the corresponding 95% CIs) will be provided at yearly interval.

The cumulative incidence rate over time (at 6 months and by year) together with appropriate interval will be estimated by treatment group using Kaplan-Meier estimates.

Reasons for censoring (including patient who died before the cut-off date/CSED for other reason than CHD, lost to follow-up) will be summarized. For patients censored before the cut-off date/CSED, time from last contact when information on efficacy endpoints has been retrieved to cut-off date will be summarized.

Interim analyses

Two interim analyses will be performed. See Section 4 for description of these analyses. The cut-off dates of final (ie, CSED) and interim analyses are expected to be:

- First interim analysis date (futility): when 807 patients have experienced at least 1 primary efficacy event (50% fraction information);
- Second interim analysis date (futility and overwhelming efficacy): when 1210 patients have experienced at least 1 primary efficacy event (75% fraction information);
- Final analysis date: when 1613 patients have experienced at least 1 primary efficacy event or 24 months after the last date of randomization ex-China, whichever comes last.

Sensitivity analyses

The following sensitivity analyses will be performed:

<u>Primary efficacy endpoint as per investigator</u>

A sensitivity analysis of the primary efficacy endpoint will be performed including any events up to the CSED with final diagnosis by the investigator confirming the event, whether or not confirmed by the CEC. The statistical methodology used will be the same as defined for the primary efficacy analysis.

Since Investigators are requested to report any UA regardless of whether the event fulfils the stricter protocol definition or not, a subset of the reported UA will be selected using information reported on the e-CRF on hospitalization forms, additional information reported on the UA form related to ECG findings, need for revascularization procedure, and/or the concomitance of elevation of cardiac biomarkers (see Appendix C for full details). Since the categorization of

deaths as CHD death was not requested from the Investigator, CHD deaths as per Investigator will include all deaths with primary cause of death reported as "Acute myocardial infarction", "Sudden cardiac death", "Heart failure or cardiogenic shock" as per investigator. The category "Undetermined cause of death" as per Investigator will be also included in this endpoint.

The concordance rate between Investigator opinion and adjudication by the CEC will be provided for all CV events adjudicated, by CV event's type.

Primary endpoint analysis excluding undetermined causes of death and undetermined causes of stroke

The primary efficacy analysis will be also performed excluding deaths adjudicated as "undetermined causes of death" and strokes adjudicated as "undetermined causes of stroke" by the CEC.

Supportive analyses

The primary efficacy outcome will be analyzed on randomized and treated patients considering only events that occurred during the treatment period (ie, from the first double-blind IMP injections to the last double-blind IMP injections +21 days, or up to the date of the CSED, whichever comes first), using the same statistical methodology as for the primary efficacy analysis. If a patient does not have a primary endpoint during the treatment period, the patient will be right-censored at the date of last contact when information on efficacy endpoints (presence or absence) has been retrieved, or at the date of death, or at the CSED or at the date of last double-blind IMP injection +21 days, whichever comes first.

Analysis on all events (ie, including recurrent events after the primary efficacy endpoints) will be also performed. Risk ratios between treatments groups will be estimated by Andersen-Gill (4) mean intensity model and the robust sandwich estimate of Lin and Wei (5) for the covariance matrix. Cumulative mean function and 95% CI in each treatment group will be calculated using Nelson-Aalen estimate.

Subgroup analyses

The consistency of the treatment effect on primary efficacy outcome will be evaluated with respect to the following demographic/baseline characteristic and prognostic factors:

- Gender;
- Age group (<65, ≥65);
- Race (Caucasian, Black, Asian/Oriental, and Other, as appropriate);
- Country (IVRS stratum, depending of the size of subgroups);
- Region (USA/Non-USA, and North America/South America/Eastern Europe/Western Europe/Asia/Rest of world);
- Time from ACS event to randomization (eg, ≤24 weeks, >24 weeks).

For each factor except the region, a Cox proportional hazard model will be used, including the treatment, the region, the factor, and the treatment-by-factor interaction terms as covariates. Within each selected factor, the treatment effect hazard ratio and its CI will be estimated from this Cox model. P-values of interaction will be also provided. Results will be plotted using forests plot. The treatment effect by region will be estimated using the similar, Cox proportional model with treatment, region, and treatment-by-region interaction as the covariates.

In addition, Kaplan-Meier curves and summary statistics showing number of patients, number (%) of primary efficacy outcome events, cumulative incidence of events at 6 months and by year, and appropriate CI may be provided for each treatment arm in previously selected subgroups defined above.

In addition, the effect of the time from ACS to randomization (in weeks) will be assessed using a Cox Proportional Hazard model including the time from ACS event (continuous) as a covariate, the treatment group and the interaction.

In addition, homogeneity of treatment effect in the following subgroups will be explored (providing sufficient number of events per subgroups):

- BMI (<30, ≥ 30 kg/m²)
- Age (<65, ≥65 to <75, ≥75)
- Ethnicity (hispanic or latino/not hispanic nor latino)
- Statin treatment at randomization in three categories (high dose atorvastatin/rosuvastatin; any other statin [ie, low/moderate doses of atorvastatin/rosuvastatin, any dose of other statins]; no statin)
- Diabetes mellitus status at baseline
- Baseline LDL-C (<80, ≥80 to <100, ≥100 mg/dL)
- Index ACS event (STEMI, NSTEMI, UA)
- Prior stroke
- Baseline non-HDL-C (<110, ≥110 to <130, ≥130 mg/dL)
- Baseline Apo B (<75, ≥75 to <90, ≥90 mg/dL)
- Baseline Lp (a) (<50, ≥50 mg/dL)
- Baseline hs-CRP (<2, ≥2 mg/L)

3.4.4.2 Analyses of secondary efficacy endpoints

Method for controlling the overall type-I error rate when testing the main secondary efficacy endpoints is described in Section 3.4.4.3.

Secondary endpoints will be analyzed using the same statistical methodology as for the primary endpoint.

3.4.4.3 Multiplicity issues

In order to handle multiple main secondary endpoints, the overall type-I error will be controlled by the use of a sequential inferential approach. Statistical significance of the primary endpoint is required before drawing inferential conclusions about first main secondary endpoint (at the 0.0001 1-sided alpha level at the second interim analysis or at the 0.0249 1-sided alpha level at the final analysis). Inferential conclusions about successive main secondary parameters require statistical significance of the prior one. The order of tests is detailed in Section 3.1.3.2.1.

This fixed hierarchical approach will ensure a strong control of the overall type-I error rate at the required 1-sided level (0.0001 for the second interim analysis and 0.0249 at the final analysis).

No further adjustments will be made for other secondary endpoints for which p-values will be provided for descriptive purpose only.

3.4.4.4 Additional efficacy analyses

Additional efficacy analyses, endorsed by the steering committee, may be defined in an exploratory SAP, before the unblinding of the treatment code. In particular the relationship between lipid lowering effects and the outcome of cardiovascular efficacy endpoints will be assessed.

3.4.5 Analyses of safety data

The summary of safety results will be presented by treatment group.

General common rules

All safety analyses will be performed on the safety population as defined in Section 3.3.2, unless otherwise specified, using the following common rules:

- Safety data in patients who do not belong to the safety population (ie, exposed but not randomized) will be listed separately;
- The baseline value is defined as the last available value before first double-blind IMP injection, except otherwise specified;

The potentially clinically significant abnormality (PCSA) values are defined as abnormal values considered medically important by the Sponsor according to predefined criteria/thresholds based on literature review and defined by the Sponsor for clinical laboratory tests, vital signs, and ECG (see Appendix A). In case the PCSA threshold is within the normal laboratory ranges, the analysis will be done using “<LLN” or “>ULN” threshold instead of “<PCSA threshold” or “>PCSA threshold” respectively. Of note, for HbA_{1c}, usual PCSA criteria will not be applied as specific analysis for the incidence of diabetes during the TEAE period will be provided, combining information from adverse event, laboratory parameters, and antidiabetic medications (see detail in Section 3.4.5.3).

- PCSA criteria will determine which patients had at least 1 PCSA during the TEAE period, taking into account all evaluations performed during this period, including nonscheduled, local or repeated evaluations.
- The treatment-emergent PCSA denominator by group for a given parameter will be based on the number of patients assessed for that given parameter in the TEAE period by treatment group on the safety population.
- All measurements, scheduled or unscheduled, fasting or not fasting, will be assigned to analysis windows defined in Section 3.5.4 Table 7.
- For quantitative safety parameters based on central laboratory/reading measurements, descriptive statistics will be used to summarize results and change from baseline values by visit (using analysis windows defined in Section 3.5.4 Table 7) and treatment group. Summaries will include the last on-treatment value and the worst on-treatment value.
- The worst value is defined as the nadir and/or the peak value during the treatment period according to the direction (minimum or maximum) of the abnormality as defined in the PCSA list.
- For exploratory purpose, safety analyses could also be provided according to up-titration status, ie, according to whether the patients remained on the 75 mg dose or whether they were up-titrated to 150 mg. These analyses will be exploratory and descriptive (no formal comparison per dose) as it is expected that there could be inherent differences in the baseline characteristics between those patients titrating to 150 mg and those remaining on 75 mg. In order to reduce the bias of this analysis, the period before the up-titration for patients up-titrated and the period before the first up-titration time point (ie, Month 2) for patients not up-titrated will not be included in the analysis since only the dose 75 mg is proposed for this time period and consequently the early events can only be attributed to this dose. Therefore the descriptive analysis per dose will include any safety events occurring from the first injection post up-titration time point IVRS/IWRS transaction to the end of the TEAE period or to 70 days after down-titration to 75 mg (if any), whichever comes first. Event-rate per patient-year will also be provided after up-titration time point to take into account variable duration of exposure.
- Analyses performed according to diabetes mellitus status at baseline will be done using the definition provided in Section 3.1.1.

3.4.5.1 Analyses of adverse events

Generalities

The primary focus of adverse event reporting will be on TEAEs. Pre- and post-treatment adverse events will be described separately.

If an adverse event date/time of onset (occurrence, worsening, or becoming serious) is incomplete, an imputation algorithm will be used to classify the adverse event as pre-treatment, treatment-emergent, or post-treatment. The algorithm for imputing date/time of onset will be conservative and will classify an adverse event as treatment emergent unless there is definitive

information to determine it is pre-treatment or post-treatment. Details on classification of adverse events with missing or partial onset dates are provided in Section 3.5.3.

Adverse event incidence tables will present by SOC, HLGT, HLT, and PT, sorted in alphabetical order for each treatment group, the number (n) and percentage (%) of patients experiencing an adverse event. Multiple occurrences of the same event in the same patient will be counted only once in the tables within a treatment phase. The denominator for computation of percentages is the safety population within each treatment group.

Sorting within tables should ensure the same presentation for the set of all adverse events within the observation period (pre-treatment, treatment-emergent, and post-treatment). For that purpose, the table of all TEAEs presented by SOC and PT sorted by the internationally agreed SOC order and decreasing frequency of PTs within SOCs will define the presentation order for all other tables unless otherwise specified. Sorting will be based on results for the alirocumab group.

As recommended by the safety planning, evaluation, and reporting team (SPERT, [6]) the analysis of all TEAEs will be split into 3 tiers for signal detection and analysis of adverse events.

- Tier 1: TEAEs with pre-specified detailed analysis: TEAEs for which hypothesis and comprehensive analytical approach are prospectively defined.
- Tier 2: signal detection among common TEAE: (not prespecified).
- Tier 3: descriptive analysis of infrequent TEAEs: TEAEs which are infrequent. This could include some AESI predefined in the protocol (eg, pregnancy, hemolytic anemia) to ensure close monitoring but which are expected to be so rare that statistical analysis is not meaningful. For those events, medical judgment should prevail.

Prospective analysis for Tier 1 events

Tier 1 events will include the AESIs and grouping of adverse events as defined in Section 3.1.4.1. Some of these events may be analyzed as Tier 3 in case their occurrence is infrequent. For each selected Tier 1 event, comprehensive analytic approach will be conducted, as described below, in order to evaluate whether the incidence is higher in the alirocumab group versus the placebo group.

Descriptive summaries of Tier 1 events

The number (%) of patients with an event in the TEAE period will be summarized in each treatment group: 95% CIs of the incidence rate (%) will be provided (CI calculated using the mid-p method) for each Tier 1 grouping of terms. The event rate per 100 patient-years (the number of patients with an event in question divided by total 100 patient-years), as well as 95% CI will be also provided. For a patient with an event, patient year is censored at time of first event; for patient without event, it corresponds to the length of the TEAE period. If the event is defined as a grouping of terms, the table will be presented by SMQ/CMQ and PT (when selection is based on SMQ/CMQ) and by PT (when selection is based on the e-CRF tick box or HLGT/HLT), showing the number (%) of each PT included in the grouping of terms. The summaries will be sorted by decreasing incidence of PT within each SMQ/CMQ (in the alirocumab group). In addition, above

description will be provided according to diabetes mellitus status at baseline for the diabetes mellitus or diabetic complications grouping.

An overview of each Tier 1 TEAE will be also provided in each treatment group: number (%) of TEAE, of treatment-emergent SAE, of TEAE leading to death and of TEAE leading to permanent treatment discontinuation. In addition, a summary of the following characteristics at grouping level will be provided: the severity grade (mild, moderate, severe), the outcome (Recovered/Resolved, Recovering/Resolving, Unknown, Recovered/Resolved with sequelae, Stabilized, Not recovered/Not Resolved, Fatal), the seriousness, the outcome status of Tier 1 TEAE leading to premature treatment discontinuation. In addition, summary by SMQ/CMQ and PT will be provided for serious TEAEs and TEAEs leading to permanent treatment discontinuation.

Time to liver-related treatment discontinuation and time to liver death may also be provided using hepatic disorder SMQ.

Additional statistical analyses for Tier 1

In order to compare treatment groups, the hazard ratio (HR) will be provided together with the corresponding 95% CI. Hazard ratio will be calculated using a Cox model. Patient without any event will be censored at the end of the TEAE period.

Kaplan-Meier curves for time from first dose of double-blind IMP to the first occurrence of Tier 1 TEAE will be provided for each Tier 1 (grouping of terms). Patient without any event will be censored at the end of the TEAE period.

In addition, HR and Kaplan-Meier curves and estimates will be provided according to diabetes mellitus status at baseline for the diabetes mellitus or diabetic complications grouping.

To assess the homogeneity of the treatment effect across age groups (<65 years versus ≥65 years, and <75 years versus ≥75 years), the treatment-by-age interaction will be tested in a Cox model including the age factor term and the treatment-by-age interaction term. Hazard ratio and the corresponding 95% CI within each age subgroup (calculated using a Cox model), as well as the significance level of the treatment-by-age interaction term will be also provided for descriptive purpose.

Additional summaries for local injection site reaction

The following description of local injection site reaction will be tabulated:

- Number of local injection site reaction per patient: 1, >1;
- Mean duration;
- Number of events divided by the number of double-blind IMP injections received;
- Time from first double-blind IMP injection to first local injection site reaction;
- Number of double-blind IMP injections received up to the first event;

- Intensity of the event (mild, moderate, severe);
- Description of the highest intensity of each symptom recorded in the specific e-CRF page.

Analysis of all "common" TEAE(s) - Tier 2 events

"Common" events are defined as those for which there are more (>) than n patients with an event overall in the safety population. This threshold will be defined as the number of patients with events (n) observed overall (whatever the treatment group) for which the extreme case scenario (n for alirocumab versus 0 for the placebo) doesn't allow the p-value to be less than 0.05.

All common TEAEs (by HLT and PT), showing the number (%) of TEAE, the event rate per 100 patient-years, HR (estimated using a Cox model) with the corresponding 95% CI, sorted by decreasing incidence rate in alirocumab group in one table, and by decreasing HR in the another table, will be analyzed.

If any clinically significant signal is detected and need further characterization, additional analyses similar to Tier 1 analyses, will be provided.

Descriptive analysis of infrequent adverse events – Tier 3 events

All infrequent TEAEs (Tier 3) will be reported with descriptive statistics (n, %) and event rate per 100 patient year, without comparative statistics since with so rare event statistical comparisons are not meaningful and medical judgment should prevail.

Analysis of all treatment-emergent adverse events

The following TEAE summaries, including all TEAEs (common or not, Tier 1 or not) will be generated for the safety population.

- Overview of TEAEs, summarizing number (%) of patients with any:
  - TEAE;
  - Serious TEAE;
  - TEAE leading to death;
  - TEAE leading to permanent treatment discontinuation.
- All TEAEs by primary SOC, HLGT, HLT, and PT, sorted by the SOC internationally agreed order. The other levels (HLGT, HLT, PT) will be presented in alphabetical order;
- All TEAEs regardless of relationship and related to IMP by primary SOC, HLGT, HLT, and PT, sorted by the internationally agreed SOC order. The other levels (HLGT, HLT, PT) will be presented in alphabetical order;
- All TEAEs regardless of relationship and related to statin/other lipid lowering drug by primary SOC, HLGT, HLT, and PT, sorted by the internationally agreed SOC order. The other levels (HLGT, HLT, PT) will be presented in alphabetical order;

- All TEAEs by primary SOC and PT, sorted by the internationally agreed SOC order and by decreasing incidence of PTs within each SOC (in the alirocumab group). This sorting order will be applied to all other tables by SOC and PT of TEAEs, unless otherwise specified. The event rate per 100 patient-years (the number of patients with an event in question divided by total patient-years) will be provided for all TEAEs by SOC and PT. For a patient with event, patient-year is censored at time of first event; for patient without event, it corresponds to length of TEAE period;
- All TEAEs that occurred with HLT and PT incidence ≥2% in alirocumab group and at incidence at least 0.5% higher in alirocumab than placebo, by primary SOC, HLT, and PT;
- All TEAEs that occurred with incidence ≥5% in any treatment group, by primary SOC and PT, with event rate per 100 patient-years;
- All TEAEs by maximal severity (ie, mild, moderate, or severe), presented by primary SOC and PT.

Subgroup of patients with 2 consecutive LDL-C <25 mg/dL (<0.65 mmol/L)

A 2-step approach will be used to analyze the safety in relation to low LDL-C.

The first step will screen events for potential signal using 2 approaches. The first approach will be a direct comparison of patients with 2 consecutive LDL-C <25 mg/dL versus ≥25 mg/dL (ie, patients without 2 consecutive LDL-C <25 mg/dL) within the alirocumab treatment group. Since these 2 groups are based on post-randomization data with a potential for bias, a second approach will compare the alirocumab effect versus placebo according to categories based on baseline LDL-C. The frequency of patients with 2 consecutive LDL-C <25 mg/dL is expected to be the largest in the category with the lowest baseline LDL-C and be lower and lower in the categories with higher baseline LDL-C. Therefore, an event induced by low LDL-C should be associated with a higher alirocumab effect versus placebo (ie, higher HR) in the first baseline LDL-C category(ies) than in the subsequent categories. Details of these 2 approaches are provided below.

The second step will consist in the comparison of patients with 2 consecutive LDL-C <25 mg/dL versus ≥25 mg/dL within the alirocumab group for the events detected in the first step, as well as for pre-specified events.

Similar analyses will be provided considering 15 mg/dL (0.39 mmol/L) as threshold instead of 25 mg/dL.

First step

First screening approach

TEAE summary by primary SOC, HLGT, HLT, and PT as well as groupings of events (ie, cataract, neurological events, neurocognitive disorders and "new onset of diabetes" [see Section 3.4.5.3 for the definition]) will be provided on the safety population in the groups below:

- Placebo group
- Alirocumab group
- Alirocumab LDL-C ≥25 mg/dL (ie, alirocumab patients without 2 consecutive LDL-C <25 mg/dL)
- Alirocumab patients with 2 consecutive LDL-C <25 mg/dL

For patients with 2 consecutive LDL-C <25 mg/dL, analyses will be done on the period starting from the first of the 2 consecutive LDL-C lower than 25 mg/dL to the upper limit of the TEAE period excepted for patients down-titrated from 150 mg to 75 mg for whom the analysis period will end at the date of last injection of 150 mg +70 days (as patients down-titrated from 150 mg to 75 mg are likely to come back above 25 mg/dL).

The time to the first TEAE/event will be compared for patients with 2 consecutive LDL-C <25 mg/dL versus ≥25 mg/dL, within the alirocumab group, using a Cox model, including the covariate for 2 consecutive LDL C <25 mg/dL (Yes/No). Hazard ratio (and 95% CI) from this model will be provided. The event rate per 100 patient-years (the number of patients with an event in question divided by total 100 patient-years) will also be provided.

Second screening approach

TEAE summary by primary SOC, HLGT, HLT, and PT as well as groupings of events (ie, cataract, neurological events, neurocognitive disorders and "new onset of diabetes" will be provided on the safety population according to baseline LDL-C categories (eg, <70, ≥70 to <90, ≥90 to <110, ≥110 mg/dL).

Hazard ratio (and 95% CI) for alirocumab effect versus placebo within each baseline LDL-C subgroup will be provided using a Cox model with baseline LDL-C (in categories), treatment group and the treatment-by-baseline LDL-C interaction term.

To assess the impact of baseline LDL-C on hazard ratio, a Cox model including the baseline LDL-C (as continuous factor), treatment group and treatment-by-baseline LDL-C interaction term will be used. The p-value from the interaction term will be provided for descriptive purpose to evaluate if there is a potential relationship between baseline LDL-C and hazard ratio.

Second step

For each pre-specified event (cataract, neurological events, neurocognitive disorders, new onset of diabetes) as well as for each event with a potential signal detected in the first step, the time to the

first TEAE/event will be compared for patients with 2 consecutive LDL-C <25 mg/dL versus ≥25 mg/dL, within the alirocumab group, using a Cox model, including the covariate for 2 consecutive LDL-C <25 mg/dL (Yes/No) and prognostic factors of the event analyzed. Adjusted HR (and 95% CI) from this model will be provided.

The list of prognostic factors will be established based on the literature and on study data (as applicable).

Subgroups of patients with treatment-emergent ADA positive response

All TEAEs by primary SOC, HLGT, HLT, and PT as well as local injection site reactions will be described in the alirocumab group according to the following ADA parameters:

- Treatment-emergent ADA positive response (yes/no)
- Persistent/transient/indeterminate treatment-emergent ADA positive response

Analysis of all treatment-emergent serious adverse event(s)

- All treatment-emergent SAEs by primary SOC, HLGT, HLT, and PT, sorted by the internationally agreed SOC order. The other levels (HLGT, HLT, PT) will be presented in alphabetical order;
- All treatment-emergent SAEs regardless of relationship and related to IMP, by primary SOC, HLGT, HLT, and PT, sorted by the internationally agreed SOC order. The other levels (HLGT, HLT, PT) will be presented in alphabetical order.
- All treatment-emergent SAEs by primary SOC and PT,

Analysis of all treatment-emergent adverse event(s) leading to permanent treatment discontinuation

- All treatment-emergent adverse events leading to treatment discontinuation, by primary SOC, HLGT, HLT, and PT, sorted by the internationally agreed SOC order. The other levels (HLGT, HLT, PT) will be presented in alphabetical order.
- All TEAEs leading to treatment discontinuation by primary SOC and PT;

Analysis of pre-treatment and post-treatment adverse events

- All pre-treatment adverse events by primary SOC and PT sorted by the internationally agreed SOC order and decreasing incidence of PTs within each SOC;
- All pre-treatment adverse events leading to treatment discontinuation (if any) by primary SOC and PT, sorted by the internationally agreed SOC order and decreasing incidence of PTs within each SOC;
- All post-treatment adverse events by primary SOC and PT, sorted by the internationally agreed SOC order and decreasing incidence of PTs within each SOC;
- All post-treatment SAEs by primary SOC and PT, sorted by the internationally agreed SOC order and decreasing incidence of PTs within each SOC.

(electronic 1.0)

3.4.5.2 Deaths

The following summaries of deaths will be generated for the safety population.

- Number (%) of patients who died from the first IMP injection until CSED and reasons for death as adjudicated by the CEC;
- Deaths occurring after CSED (adjudicated or not);
- Treatment-emergent adverse events leading to death (death as an outcome on the adverse event case report form page as reported by the Investigator) by primary SOC, HLGT, HLT, and PT sorted by internationally agreed SOC order, with HLGT, HLT, and PT presented in alphabetical order within each SOC;
- All post-treatment adverse events leading to death by primary SOC and PT, sorted by the internationally agreed SOC order and decreasing incidence of PTs within each SOC.
- In addition, deaths in non-randomized patients or randomized but not treated patients will be displayed.

3.4.5.3 Analyses of laboratory variables

Descriptive statistics over time

The summary statistics (including number, mean, median, Q1, Q3 standard deviation, minimum, and maximum) of all laboratory variables (central laboratory values and changes from baseline) will be calculated for each visit or study assessment (baseline, each post-baseline time point, last on-treatment and worst on-treatment value) by treatment group during the treatment period.

For glucose, this summary will be also provided according to the diabetes status at baseline. Only fasting samples will be summarized.

In addition, for some parameters of interest, mean changes from baseline with the corresponding standard error could be plotted over time in each treatment group.

Potentially clinically significant abnormalities

The incidence of PCSAs (list provided in Appendix A) as well as ALT increase as defined as AESI and hemoglobin decrease from baseline ≥15 g/L at any time during the TEAE period will be summarized by biological function and treatment group whatever the baseline level and/or according to the following baseline status categories:

- Normal/missing;
- Abnormal according to PCSA criterion or criteria.

For glucose, this summary will also be provided according to the diabetes status at baseline. Only fasting samples will be summarized.

For parameters for which no PCSA criteria are defined, similar table(s) using the normal range will be provided.

PCSA summaries will also be provided in patients from alirocumab group with 2 consecutive LDL-C <25 mg/dL in case a signal detected in the adverse events analyses (see Section 3.4.5.1) warrants further investigations. Only PCSA occurring after the first occurrence of LDL-C <25 mg/dL will be considered (see Section 3.4.5.1, sub-section "Subgroup of patients with two consecutive LDL-C <25 mg/dL (<0.65 mmol/L)" for the definition of the analysis period).

Analysis of new onset of diabetes

The incidence of new onset diabetes during the TEAE period will be analyzed in the subgroup of patients not having diabetes at baseline (see Section 3.1.1). New onset diabetes will be defined as follows, combining information from adverse events, medication, and laboratory parameters:

- Type 1 or 2 diabetes TEAE (CMQ "Type 1 or type 2 diabetes", Table 10), and/or
- Anti-diabetic medication initiated during the TEAE period, and/or
- At least 2 HbA_{1c} ≥6.5% during the TEAE period (for patients with a single measurement available during the TEAE period, a single value ≥6.5% will be considered).

Hepatitis C antibody

The number and percentage of patients with a post-baseline seroconversion for hepatitis C test will be provided by treatment group in post-baseline (including the TEAE and post TEAE periods). Post-baseline seroconversion is defined for patients with a negative baseline status who had either a "positive ribonucleic acid" (RNA) or a "confirmed positive antibody with negative RNA" post-baseline status as defined in the table below. Other situations require case by case evaluation and will be described individually if relevant.

The status as regards to hepatitis C virus (HCV) for a patient will be defined as follows for all evaluations (baseline and post-baseline).

Table 5 - Definition of the patient status regarding hepatitis C virus

	**Hepatitis C Antibody (Ab) test result**				
	Negative		Positive		
Reflexive test[a] – hepatitis C RNA test	Not available or HCV RNA not detected	HCV RNA detected	HCV RNA not detected[b]	HCV RNA detected	Not available
Hepatitis C status - label	Negative	Positive RNA	Negative[b]	Positive RNA	Positive Ab – no RNA available

a Test performed at the same time or after the antibody test in the pre-treatment period (for baseline evaluation), or post-baseline, respectively

b For post-baseline evaluation, a second antibody test with a different type of assay is to be done at the same date or after the first antibody test. The result of this test will modify the final hepatitis C status of the patient in some cases (see details in the text below the table)

The baseline evaluation will be based on tests performed during the pre-treatment period.

In case of multiple hepatitis C tests available for the post-baseline evaluation, the positive status of the patient will be defined as follows:

- "Positive RNA" status if at least 1 post-baseline positive RNA is detected, regardless of status of the patient at the end of treatment.
- Else "Positive Ab – no RNA available" status if no post-baseline reflexive RNA test is available for at least 1 post-baseline positive antibody test.

If no antibody test is available or with "indeterminate" as result pre-treatment or post-baseline, respectively, the RNA test (if available) will be used alone to determine the status of the patient. If no RNA is available then the hepatitis C status of the patient will be missing.

The post-baseline status "confirmed positive antibody with negative RNA" will replace "Negative" status as defined above in the case where no RNA was detected post-baseline and the 2 antibody tests surrounding the same visit (from 2 different types of assay) are positive.

For a conservative approach, the post-baseline status "Positive Ab – no RNA available" will not be modified by the availability of a second antibody test from a different assay.

Possible drug-induced liver injury

The liver function tests, namely AST, ALT, alkaline phosphatase, and total bilirubin, are used to assess possible drug-induced liver toxicity. The proportion of patients with PCSA values at any post-baseline visit by baseline status will be displayed by treatment group for each parameter.

A graph of distribution of peak values of ALT versus peak values of total bilirubin will also be presented. Note that the ALT and total bilirubin values are presented on a logarithmic scale. The graph will be divided into 4 quadrants with a vertical line corresponding to 3 x ULN for ALT and a horizontal line corresponding to 2 x ULN for total bilirubin.

Graph and listing of possible Hy's law cases identified by treatment group (eg, patients with any elevated ALT>3 x ULN, and associated with an increase in bilirubin ≥2 x ULN) with ALT, AST, alkaline phosphatase, total bilirubin, and, if available, direct and indirect bilirubin, will be provided.

3.4.5.4 Analyses of vital sign variables

The summary statistics (including number, mean, median, Q1, Q3, standard deviation, minimum, and maximum) of all vital signs variables (values and changes from baseline) will be calculated for each visit or study assessment (baseline, each post-baseline time point, last on-treatment and worst on-treatment value) by treatment group during the treatment period. In addition, for some parameters of interest, mean changes from baseline with the corresponding standard error could be plotted over time in each treatment group.

The incidence of PCSAs at any time during the TEAE period will be summarized by treatment group irrespective of the baseline level and/or according to the following baseline status categories:

- Normal/missing;
- Abnormal according to PCSA criterion or criteria.

3.4.6 Analyses of other safety parameters

Events initially suspected by investigators, and reported as such in endpoints forms, may finally not be confirmed by investigators and possibly classified into another category that is not a component of the primary efficacy endpoint. In addition, per protocol all suspected UAs have to be sent for adjudication regardless of whether the event fulfils the stricter protocol definition or not, therefore a high proportion of the reported UAs are expected to be finally not retained as an endpoint.

Since those events are not reported as adverse events either, they will be described separately as follows:

The number (%) of patients with events below during the TEAE period will be displayed by treatment group on the safety population:

- With final diagnosis as per investigator of stable coronary disease,
- With final diagnosis as per investigator of unstable angina regardless of whether the event fulfils the stricter protocol definition or not;
- With final diagnoses as per investigator of hemorrhagic stroke, transient ischemic attack (TIA), and subdural hematoma.

The number (%) of patients with hemorrhagic strokes or silent MI as per CEC during the TEAE period will also be summarized on the safety population

3.4.7 Analyses of other endpoints

All measurements, scheduled or unscheduled, fasting or not fasting, will be assigned to analysis windows defined in Section 3.5.4, in order to provide an assessment for Month 1 to Month 64 time points.

3.4.7.1 Analyses of hs-CRP

hs-CRP parameter will be summarized on the safety population by analysis visit using number of available data, mean, SD, median, Q1, Q3, minimum, and maximum for each treatment group during the treatment period. The time profile will be plotted by treatment group with the medians, Q1 and Q3. The incidence of PCSA at any time during the TEAE period will be summarized by treatment group using descriptive statistics.

hs-CRP values greater or equal to 10 mg/L will be excluded from analyses in a second approach, since these are suggestive of concurrent infections.

3.4.7.2 Analyses of HbA_{1c}

HbA_{1c} parameter will be summarized on the safety population by analysis visit using number of available data, mean, SD, median, minimum, and maximum for each treatment during the treatment period. Summary will be also provided according to the diabetes mellitus status at baseline (see Section 3.1.1). The time profile will be plotted by treatment group with the means and the corresponding standard errors (SEs).

In case the proportion of initiation of anti-diabetic medications is different between the 2 treatment groups, further analysis of HbA_{1c} over time would be performed.

3.4.7.3 Analyses of patients with LDL-C <25 mg/dL(<0.65 mmol/L)

The number and percentage of patients with 2 consecutive LDL-C <25 mg/dL (respectively, LDL-C <15 mg/dL, ie, 0.39 mmol/L) will be provided by treatment group on the safety population. Kaplan-Meir curves will be provided for the time to the first LDL-C <25 mg/dL (respectively 15 mg/dL) for these patients. For this analysis, patients without post-baseline LDL-C result or with only 1 post-baseline LDL-C result will not be included.

3.4.7.4 Analyses of lipid parameters

The lipids variables (see Section 3.1.5.1) will be analyzed using an ITT approach (based on the ITT population) including all lipid values, regardless of whether the patient was continuing therapy or not. In addition, analyses will also be conducted using an on-treatment approach (based on the randomized and treated population) only including lipid data collected during the treatment period.

3.4.7.4.1 ITT analyses

A pattern-mixture model approach (see Appendix F) will be used with a different imputation strategy applied for missing lipid values during the treatment period (ie, within the time period from the first double-blind IMP injection up to the day of the last double-blind injection +21 days) and missing lipid values after treatment discontinuation (ie, after the day of last injection +21 days) based on the following assumptions:

- Patients within 21 days of their last double-blind IMP injection would continue to show benefit from treatment similar to that observed at the scheduled time point. Therefore, lipid values missing during the treatment period (samples obtained outside the specified window, no blood sample available although visit was performed, etc) should be considered "Missing At Random" and imputed based on other on-treatment measurements;
- Patients who stopped taking their study treatment no longer benefited from it after discontinuation and thus tended to have lipid values returning to baseline. Thus lipid

values missing after treatment discontinuation will be imputed based on patient's own baseline value.

Missing lipid values will be imputed 10 times using the MI SAS® procedure, to generate 10 complete data sets. The percent change from baseline and/or the absolute change from baseline at a pre-specified time point will be derived from observed and imputed lipid value at this time point. Imputed values for time points after CSED will be discarded.

TGs and Lp (a) data will be log-transformed before imputation process and then back-transformed to create the imputed data sets.

The completed data sets will be analyzed using an analysis of covariance (ANCOVA) model for continuous lipid variables other than Lp (a) and TGs or a robust regression (6) model for Lp (a) and TGs continuous variables with treatment group as fixed effect, and the baseline lipid value as continuous covariate. The MIANALYZE procedure will be used to generate valid statistical inferences by combining results from the 10 analyses using Rubin's formula.

The number of imputations (10) will be informally verified by replicating sets of 10 imputations and checking whether the combined results are stable. If not stable, the number of imputations will be increased and informally checked as above, and thus until stable estimates are obtained.

The value at the CSED will be the value obtained at the CSED visit. For patients without CSED visit, the last lipid value observed or imputed up to CSED will be taken into account.

Throughout the ANCOVA and robust regression models, the alirocumab group will be compared to placebo using appropriate contrasts tested at the two-sided 0.05 level, and providing the 95% CI of the difference, for the different time points as well as at the CSED.

No adjustment will be made for lipid variables for which p-values will be provided for descriptive purpose.

3.4.7.4.2 On-treatment analyses

Analysis of lipid variables will be conducted during the treatment period. Post-treatment data will not be considered.

The lipid variables other than Lp (a) and TGs will be analyzed in the randomized and treated population using a mixed-effect model with repeated measures (MMRM) approach. All post-baseline on-treatment data available within Month 1 to Month 64 analysis windows will be used and missing data will be accounted for by the MMRM model.

The model will include the fixed categorical effects of treatment group (placebo, alirocumab), planned time point (Month 1 to Month 64), treatment-by-time point interaction, as well as, the continuous fixed covariate of baseline lipid value and baseline lipid value-by-time point interaction. This model will be run using SAS Mixed procedure with an unstructured correlation matrix to model the within-patient errors. Parameters will be estimated using restricted maximum

likelihood method with the Newton-Raphson algorithm. Denominator degrees of freedom will be estimated using Satterthwaite's approximation. This model will provide baseline adjusted least-squares means estimates over time for both treatment groups with their corresponding SEs and 95% CI. To compare the alirocumab group to the placebo group, appropriate contrasts statement will be used to test the differences of these estimates, at the 2-sided 0.05 level, for the different time points.

Note: in case of computation issue for this approach, multiple imputation under missing-at-random (MAR) assumption will be conducted, followed by an ANCOVA model.

The Lp (a) and TGs will be analyzed in the randomized and treated population using multiple imputation (same imputations as in Section 3.4.7.4.1 without discarding imputations of missing values during the post-treatment period (see Appendix F).

3.4.7.5 Analyses of anti-alirocumab antibody variables

The following summaries will be performed on the ADA population, taking into account all samples regardless of timing in relation to injections. ADA results will be summarized by treatment group and up-titration status (see Section 3.4.5).

- ADA results (negative or positive) by time point;
- Neutralizing status (negative or positive) by time point for positive ADA;
- ADA titers using descriptive statistics (median, minimum, and maximum) for positive ADA by time point;
- Number (%) of patients with pre-existing ADA and number (%) of patients with treatment-emergent ADA positive response;
- Number (%) of patients with persistent/transient/indeterminate treatment-emergent ADA positive response;
- Time to onset of treatment-emergent ADA positive response using descriptive statistics;
- Number (%) of patients with at least 1 neutralizing ADA.

Correlations between ADA parameters (eg, titers, treatment-emergent ADA positive status, neutralizing status), safety and/or efficacy endpoints will be also explored (eg, scatter plot).

3.4.7.6 Analyses of quality of life/health economics variables

The analysis of data from EQ-5D instrument will be performed on the ITT population.

Baseline is defined as the Visit 3 (Day 1) evaluation. Analysis window will be used to assign the measurements to time points (see Section 3.5.4).

Individual EQ-5D items

Response for each one of the 5 EQ-5D items will be summarized by time point for each treatment group with number (%) of patients reporting level 1 (no problems), level 2 (some problems), and level 3 (extreme problems) by item.

EQ-5D utility score

The raw value and the change from baseline of the utility score will be summarized using mean, median, Q1, Q3, SD, minimum, and maximum for each post-baseline visit. Cumulative distribution functions for the change in utility score from baseline will be displayed by treatment groups over time.

The change from baseline in utility score over time will be analyzed using a MMRM model with fixed categorical effects of treatment group, planned time points up to the CSED, treatment-by-time point interaction, as well as, the continuous fixed covariates of baseline value and baseline value-by-time point interaction.

Note: In case of computation issue for this approach, multiple imputation under MAR assumption will be conducted, followed by an ANCOVA model.

3.4.7.7 Analysis of cardiovascular events of interest (other than efficacy endpoint)

Other cardiovascular events of interest (see Section 3.1.5.7) will be analyzed using a time-to-event approach (Kaplan-Meier methodology) in the ITT population. Patients without any event will be censored using the same methodology as for the primary efficacy endpoint.

3.4.8 Analyses of pharmacokinetic and pharmacodynamic variables

Not applicable.

3.5 DATA HANDLING CONVENTIONS

3.5.1 General conventions

The following definitions/formulas will be used for computation of parameters.

Common study end date

The common study end date is defined as the date when 1613 patients have experienced at least 1 primary efficacy event or 24 months after the last date of randomization ex-China, whichever comes last.

Date of last dose of investigational medicinal product

The date of the last dose of IMP is equal to the last date of administration reported on the IMP administration case report form page, or missing if the last administration date is unknown. For patients on the alirocumab arm who will switch to placebo injection due to blinded treatment discontinuation, the date of last administration reported associated to an active injection will be considered.

Renal function formulas

eGFR value will be derived using the Modification of the Diet in Renal Disease (MDRD) equation:

175 x (creatinine in µmol/L / 88.4)$^{-1.154}$ x (age in years$^{)-0.203}$ (x 0.742 if female, x 1.212 if race is "black or african american").

Lipids variables, laboratory safety variables, hs-CRP

For data below the lower limit of quantification (LLOQ)/limit of linearity, half of the lower limit value (i.e; LLOQ/2) will be used for quantitative analyses. For data above the upper limit of quantification (ULOQ) / limit of linearity, the upper limit value (ie, ULOQ) will be used for quantitative analyses.

The above rules won't be applied for the calculated LDL-C and non-HDL-C when HDL-C value is below the LLOQ. The value of LLOQ/2 for HDL-C will be used to obtain the non-HDL-C and calculated LDL-C used for quantitative analyses.

Below is an example of data for a dummy patient reported in the database, with the values that will be used in quantitative analyses for each parameters.

Table 6 - Example of lipid data for a dummy patient

Parameter	Value reported in the database	Value used in the analysis
TC	255 mg/dL	255 mg/dL
HDL-C	<10 mg/dL	5 mg/dL
Calculated LDL-C	<221 mg/dL	216 mg/dL
NON-HDL-C	<255 mg/dL	250 mg/dL
TRIG	172 mg/dL	172 mg/dL

* Friedewald formula for calculated LDL-C (when lipid expressed in mg/dL: LDL-C=NON-HDL-C-0.2*TG

3.5.2 Data handling conventions for secondary efficacy variables

Rules defined for the primary efficacy variable will apply to time-to-event secondary efficacy variables.

3.5.3 Missing data

For categorical variables, patients with missing data are not included in calculations of percentages unless otherwise specified. When relevant, the number of patients with missing data is presented.

Handling of missing or incomplete dates of primary endpoint events

Rules for imputations are detailed in Table 4.

Handling of baseline definition if time of first double-blind injection or time of assessment at visit 3 is missing

If the time of the first double-blind injection or the time of assessment at Visit 3 is missing then the baseline value is defined as the last available value obtained before or on the day of the first double-blind IMP injection.

Handling of computation of treatment duration and compliance if IMP first or end of treatment date is missing

If the IMP first or end of treatment date is missing, the exposure duration and compliance will be left as missing.

Handling of treatment/TEAE analysis periods and survival analysis if IMP end of treatment date is unknown

If the last injection (last active injection for patients randomized in the alirocumab group, last injection from a double-blind kit for patients randomized in the placebo group) date is missing or incomplete, this date will be imputed to the earliest of the dates below to define the upper bound of the treatment/TEAE analysis periods and to define the censoring date for survival analyses performed on these periods:

- The last day of the month and year, when only the day is missing, or the 31st of December of the year, when only the year is known;
- The date of the end of treatment visit (CSED visit for completer, early end of treatment visit for patients who prematurely discontinued the IMP);
- The date of the last contact;
- The date of death (if any);
- The date of first injection of placebo following the switch to placebo in IVRS (if any).

Exception: In case the last active injection for a patient allocated to the alirocumab group was inadvertently received after the first injection of placebo following the switch to placebo in IVRS, the last active injection date (missing or incomplete) will be imputed to the earliest of the dates below:

- The last day of the month and year, when only the day is missing, or the 31st of December of the year, when only the year is known;
- The date of the end of treatment visit (CSED visit for completer, early end of treatment visit for patients who prematurely discontinued the IMP);
- The date of the last contact;
- The date of death (if any);
- The date of the placebo injection (if any) following the last active injection.

Handling of medication missing/partial dates

No imputation of medication start/end dates or times will be performed. If a medication date or time is missing or partially missing and it cannot be determined whether it was taken prior or concomitantly, it will be considered a prior, concomitant, and post-treatment medication, unless otherwise specified.

Handling of adverse events with missing or partial date/time of onset

Missing or partial adverse event onset dates and times will be imputed so that if the partial adverse event onset date/time information does not indicate that the adverse event started prior to treatment or after the TEAE period, the adverse event will be classified as treatment-emergent. No imputation of adverse event end dates/times will be performed. These data imputations are for categorization purpose only and will not be used in listings. No imputation is planned for date/time of adverse event resolution.

Handling of adverse events when date and time of first IMP administration is missing

When the date and time of the first IMP administration is missing, all adverse events that occurred on or after the day of randomization will be considered as TEAEs.

Handling of missing assessment of relationship of adverse events to IMP

If the assessment of the relationship to IMP is missing for an adverse event, the adverse event will be considered as related to the IMP in the tables of possibly related adverse events, but no imputation will be done at the data level

Handling of potentially clinically significant abnormalities

If a patient has a missing baseline value he/she will be grouped in the category “normal/missing at baseline.”

For PCSAs with 2 conditions, one based on a change from baseline value or a normal range and the other on a threshold value, with the first condition being missing, the PCSA will be based only on the second condition.

For a PCSA defined on a threshold and/or a normal range, this PCSA will be derived using this threshold if the normal range is missing (eg, “>0.5 GIGA/L” criterion will be used for eosinophils for the PCSA “>0.5 GIGA/L or >ULN if ULN ≥0.5 GIGA/L” when ULN is missing.

Measurements flagged as invalid by the laboratory will not be summarized or taken into account in the computation of PCSA values.

3.5.4 Windows for time points

Data analyzed by time point (including lipid data, laboratory safety data, vital signs, ECG, ADA, EQ-5D) will be summarized using the time windows given in Table 7 below. These time windows will be applicable for all analyses, and they are defined to provide more homogeneous data for time point-specific analyses.

Table 7 - Time windows definitions

Time point	Targeted study days	Time windows
Month 1	30	16 to 44
Month 2	60	45 to 75
Month 4	122	107 to 137
Month 8	244	229 to 259
Month 12 to Month 24	Number of months of the planned visit x 30.4375 and rounded to the nearest entire number of days	Targeted study day ±21 days
Beyond Month 24	Number of months of the planned visit x 30.4375 and rounded to the nearest entire number of days	Targeted study day ±28 days

If multiple valid values of a variable exist within a time window, the nearest from the targeted study day will be selected for the statistical analysis by time point. If the difference is a tie, the value after the targeted study day will be used. If multiple valid values of a variable exist within a same day, then the first value of the day will be selected.

3.5.5 Unscheduled visits

For lipid data, safety laboratory data, ECG, or vital signs, unscheduled visit measurements may be used to provide a measurement for a time window, a baseline, a time point, or a worst value, if appropriate according to their definition. The measurements may also be used to determine abnormal/PCSA values.

3.5.6 Pooling of centers for statistical analyses

The randomization scheme was not stratified by center to avoid risk of unbalance between treatment groups within a country induced by the large number of centers that will participate to the study. Nevertheless, as the primary efficacy and main secondary endpoints are centrally adjudicated by the CEC, these outcomes are not expected to be influenced by the center. Therefore, the center will not be added as factor in the primary analysis model.

Centers will be pooled into region (see Table 8) to describe the study population and to perform the primary efficacy analysis and subgroup analyses.

Table 8 - Definition of geographic regions

North America	South America	Western Europe	Eastern Europe	Asia	Rest of the World
Canada	Argentina	Austria	Bosnia Herzegovia	Hong Kong	Australia
United States	Brazil	Belgium	Bulgaria	India	Israel
	Chile	Denmark	Croatia	Japan	New Zealand
	Colombia	Finland	Czech Republic	Korea	Republic of South Africa
	Mexico	France	Estonia	Malaysia	
	Peru	Germany	Georgia	Philippines	
	Guatemala	Italy	Hungary	Singapore	
		Netherlands	Latvia	Sri Lanka	
		Norway	Lithuania	Thailand	
		Portugal	Macedonia	Taiwan	
		Spain	Poland	China	
		Sweden	Romania		
		Switzerland	Russian Federation		
		United Kingdom	Serbia		
		Greece	Slovakia		
			Slovenia		
			Turkey		
			Ukraine		

3.5.7 Statistical technical issues

Not applicable.

4 INTERIM ANALYSIS

Two interim analyses (IA) are planned, when 50% and 75% of the total number of expected events have occurred:

- Interim analysis for futility will be conducted, when approximately 807 events (50% of the targeted number of primary endpoint events) have occurred;
- Interim analysis for futility and overwhelming efficacy will be conducted, when approximately 1210 events (75% of the targeted number of primary endpoint events) have occurred.

Both IAs will be performed by the external independent CV DMC Statistician and will be reviewed under the supervision of the CV DMC. The CV DMC will also review secondary efficacy endpoints and safety data (adverse events, laboratory data, vital signs) available at the time of the IA.

Control of the type I and type II error will be ensured using gamma (-5) spending function for type II error (futility) and Gamma (-22) for type I error (efficacy) at each IA (the type I error spending function is also applied at the first IA, even if the objective of this first IA is only futility). It has to be noted that, in order to protect the global type I error in case the decision is taken to overrule the futility rule, non-binding boundaries were used.

The following table shows the stopping rules at each interim analysis (using the sample size assumptions described in Section 1.3):

Table 9 - Interim analyses stopping boundaries corresponding to Gamma (-22) type I error and Gamma (-5) type II error spending functions

Timing of analyses	Stopping boundaries (1-sided p-value and hazard ratio)	
	Futility	Overwhelming efficacy
First IA: 50% of targeted events	p >0.548<br>(⇔ HR >1.008)	NA
Second IA: 75% of targeted events	p >0.19<br>(⇔ HR >0.951)	p <0.0001[a]<br>(⇔ HR <0.802)

Calculations done using EAST® 5.4

a Should the second interim analysis be triggered just before or after 1210 events have been reached, the exact nominal significance level to be used at the second IA would be re-computed based on a Gamma(-22) spending function.

HR = hazard ratio; IA = interim analysis; NA = not applicable

The CV data monitoring committee (DMC) could consider early stopping of the study for overwhelming efficacy at the second IA, if the following conditions are met:

- Stopping boundaries for overwhelming efficacy are crossed;
- Positive trend observed for secondary efficacy endpoints, including all cause mortality, and no excess of non-CV mortality;
- Consistency of the treatment effect on the primary efficacy endpoint across the following subgroups: gender, age, race, country (depending on the size of subgroups), time from index ACS event to randomization, and regions (see Section 3.4.4.1).

(electronic 1.0)

5 DATABASE LOCK

The final database is planned to be locked approximately 3 months after the last patient last visit.

6 SOFTWARE DOCUMENTATION

All summaries and statistical analyses will be generated using SAS version 9.1 or higher.

Sample size calculations were done using EAST® 5.4 version.

7 REFERENCES

1. Cannon CP, Braunwald E, McCabe CH, Rader DJ, Rouleau JL, Berlder R et al, for the Pravastatin or Atorvastatin Evaluation and Infection Therapy-Thrombolysis in Myocardial Infarction 22 Investigators. Intensive versus moderate lipid lower¬ing with statins after acute coronary syndromes. N Engl J Med. 2004;350:1495-504.

2. Pearson TA, Mensah GA, Alexander RW, Anderson JL, Cannon RO 3rd, Criqui M, et al. Markers of inflammation and cardiovascular disease: application to clinical and public health practice: A statement for healthcare professionals from the Centers for Disease Control and Prevention and the American Heart Association. Circulation. 2003 Jan 28;107(3):499-511.

3. Dolan P. Modeling valuations for EuroQol health states. Med Care. 1997 Nov;35(11):1095-108.

4. Andersen PK, Gill RD. Cox's Regression Model for Counting Processes: a large sample study. The Annals of Statistics 1982 Dec;10(4):1100-20.

5. Lin DY, Wei LJ. The Robust Inference for the Cox Proportional Hazards Model. Journal of the American Statistical Association. 1989 Dec;84(408):1074-8.

6. Crowe BJ, Xia HA, Berlin JA, Watson DJ, Shi H, Lin SL, et al. Recommendations for safety planning, data collection, evaluation and reporting during drug, biologic and vaccine development: a report of the safety planning, evaluation, and reporting team. Clin Trials. 2009 Oct;6(5):430-40.

8 LIST OF APPENDICES

Appendix A: Potentially clinically significant abnormalities (PCSA) criteria

Appendix B: List of MedDRA terms for CMQs

Appendix C: Derivation of the efficacy endpoints/components

Appendix D: EQ-5D Patient Questionnaire

Appendix E: EQ-5D utility score algorithm

Appendix F: Detailed statistical methodology for pattern-mixture model

Appendix A Potentially clinically significant abnormalities (PCSA) criteria

CRITERIA for POTENTIALLY CLINICALLY SIGNIFICANT ABNORMALITIES
for phase 2/3 studies (oncology excepted)

Parameter	PCSA	Comments
**Clinical Chemistry**		
ALT	By distribution analysis :<br>>3 ULN<br>>5 ULN<br>>10 ULN<br>>20 ULN	Enzymes activities must be expressed in ULN, not in IU/L.<br>Concept paper on DILI – FDA draft Guidance Oct 2007.<br>Internal DILI WG Oct 2008.<br>Categories are cumulative.<br>First row is mandatory. Rows following one mentioning zero can be deleted.
AST	By distribution analysis :<br>>3 ULN<br>>5 ULN<br>>10 ULN<br>>20 ULN	Enzymes activities must be expressed in ULN, not in IU/L.<br>Concept paper on DILI – FDA draft Guidance Oct 2007.<br>Internal DILI WG Oct 2008.<br>Categories are cumulative.<br>First row is mandatory. Rows following one mentioning zero can be deleted.
Alkaline Phosphatase	>1.5 ULN	Enzymes activities must be expressed in ULN, not in IU/L.<br>Concept paper on DILI – FDA draft Guidance Oct 2007.<br>Internal DILI WG Oct 2008.
Total Bilirubin	>1.5 ULN<br>>2 ULN	Must be expressed in ULN, not in µmol/L or mg/L. Categories are cumulative.<br>Concept paper on DILI – FDA draft Guidance Oct 2007.<br>Internal DILI WG Oct 2008.
Conjugated Bilirubin	>35% Total Bilirubin and TBILI>1.5 ULN	Conjugated bilirubin dosed on a case-by-case basis.
ALT and Total Bilirubin	ALT>3 ULN and TBILI>2 ULN	Concept paper on DILI – FDA draft Guidance Oct 2007.<br>Internal DILI WG Oct 2008.<br>To be counted within a same treatment phase, whatever the interval between measurement.
CPK	>3 ULN<br>>10 ULN	FDA Feb 2005.<br>Am J Cardiol April 2006.<br>Categories are cumulative.<br>First row is mandatory. Rows following one mentioning zero can be deleted.

CRITERIA for POTENTIALLY CLINICALLY SIGNIFICANT ABNORMALITIES
for phase 2/3 studies (oncology excepted)

Parameter	PCSA	Comments
CLcr (mL/min)<br>(Estimated creatinine clearance based on the Cokcroft-Gault equation)	<15 (end stage renal disease)<br>≥15 - <30 (severe decrease in GFR)<br>≥30 - < 60 (moderate decrease in GFR)<br>≥60 - <90 (mild decrease in GFR)<br>≥ 90 (normal GFR)	FDA draft Guidance 2010<br>Pharmacokinetics in patients with impaired renal function-study design, data analysis, and impact on dosing and labeling
eGFR (mL/min/1.73m2)<br>(Estimate of GFR based on an MDRD equation)	<15 (end stage renal disease)<br>≥15 - <30 (severe decrease in GFR)<br>≥30 - < 60 (moderate decrease in GFR)<br>≥60 - <90 (mild decrease in GFR)<br>≥ 90 (normal GFR)	FDA draft Guidance 2010<br>Pharmacokinetics in patients with impaired renal function-study design, data analysis, and impact on dosing and labeling
Creatinine	≥150 µmol/L (Adults)<br>≥30% change from baseline<br>≥100% change from baseline	Benichou C., 1994.
Uric Acid<br>Hyperuricemia<br>Hypouricemia	 >408 µmol/L<br><120 µmol/L	Harrison- Principles of internal Medicine 17th Ed., 2008.
Blood Urea Nitrogen	≥17 mmol/L	
Chloride	<80 mmol/L<br>>115 mmol/L	
Sodium	≤129 mmol/L<br>≥160 mmol/L	
Potassium	<3 mmol/L<br>≥5.5 mmol/L	FDA Feb 2005.
Total Cholesterol	≥7.74 mmol/L	Threshold for therapeutic intervention.
Triglycerides	≥4.6 mmol/L	Threshold for therapeutic intervention.
Lipasemia	≥3 ULN	
Amylasemia	≥3 ULN	
Glucose<br>Hypoglycaemia<br>Hyperglycaemia	 ≤3.9 mmol/L and <LLN<br>≥11.1 mmol/L (unfasted); ≥7 mmol/L (fasted)	 ADA May 2005.<br>ADA Jan 2008.
HbA1c	>8%	
Albumin	≤25 g/L	

CRITERIA for POTENTIALLY CLINICALLY SIGNIFICANT ABNORMALITIES
for phase 2/3 studies (oncology excepted)

Parameter	PCSA	Comments
CRP	>2 ULN or >10 mg/L (if ULN not provided)	FDA Sept 2005.
**Hematology**		
WBC	<3.0 Giga/L (Non-Black); <2.0 Giga/L (Black)<br>≥16.0 Giga/L	Increase in WBC: not relevant.<br>To be interpreted only if no differential count available.
Lymphocytes	>4.0 Giga/L	
Neutrophils	<1.5 Giga/L (Non-Black);<1.0 Giga/L (Black)	International Consensus meeting on drug-induced blood cytopenias, 1991.<br>FDA criteria.
Monocytes	>0.7 Giga/L	
Basophils	>0.1 Giga/L	
Eosinophils	>0.5 Giga/L or >ULN (if ULN≥0.5 Giga/L)	Harrison- Principles of internal Medicine 17th Ed., 2008.
Hemoglobin	≤115 g/L (Male); ≤95 g/L (Female)<br>≥185 g/L (Male); ≥165 g/L (Female)<br>Decrease from Baseline ≥20 g/L	Criteria based upon decrease from baseline are more relevant than based on absolute value. Other categories for decrease from baseline can be used (≥30 g/L, ≥40 g/L, ≥50 g/L).
Hematocrit	≤0.37 v/v (Male) ; ≤0.32 v/v (Female)<br>≥0.55 v/v (Male) ; ≥0.5 v/v (Female)	
RBC	≥6 Tera/L	Unless specifically required for particular drug development, the analysis is redundant with that of Hb.<br>Otherwise, consider FDA criteria.
Platelets	<100 Giga/L<br>≥700 Giga/L	International Consensus meeting on drug-induced blood cytopenias, 1991.
**Urinalysis**		
pH	≤4.6<br>≥8	
**Vital signs**		
HR	≤50 bpm and decrease from baseline ≥20 bpm<br>≥120 bpm and increase from baseline≥20 bpm	To be applied for all positions (including missing) except STANDING.
SBP	≤95 mmHg and decrease from baseline ≥20mmHg<br>≥160 mmHg and increase from baseline ≥20 mmHg	To be applied for all positions (including missing) except STANDING.
DBP	≤45 mmHg and decrease from baseline ≥10 mmHg<br>≥110 mmHg and increase from baseline ≥10 mmHg	To be applied for all positions (including missing) except STANDING.

CRITERIA for POTENTIALLY CLINICALLY SIGNIFICANT ABNORMALITIES
for phase 2/3 studies (oncology excepted)

Parameter	PCSA	Comments
Orthostatic Hypotension		
Orthostatic SDB	≤-20 mmHg	
Orthostatic DBP	≤-10 mmHg	
Weight	≥5% increase from baseline<br>≥5% decrease from baseline	FDA Feb 2007.
**ECG**		Ref.: ICH E14 guidance (2005) and E14 Q&A (2012), and Cardiac Safety Research Consortium White Paper on PR and QRS (Nada et al. Am Heart J. 2013; 165(4) : 489-500)
HR	<50 bpm<br><50 bpm and decrease from baseline ≥20 bpm<br><40 bpm<br><40 bpm and decrease from baseline ≥20 bpm<br><30 bpm<br><30 bpm and decrease from baseline ≥20 bpm	Categories are cumulative
	>90 bpm<br>>90 bpm and increase from baseline ≥20bpm<br>>100 bpm<br>>100 bpm and increase from baseline ≥20bpm<br>>120 bpm<br>>120 bpm and increase from baseline ≥20 bpm	Categories are cumulative
PR	>200 ms<br>>200 ms and increase from baseline ≥25%<br>> 220 ms<br>>220 ms and increase from baseline ≥25%<br>> 240 ms<br>> 240 ms and increase from baseline ≥25%	Categories are cumulative
QRS	>110 ms<br>>110 msec and increase from baseline ≥25%<br>>120 ms<br>>120 ms and increase from baseline ≥25%	Categories are cumulative
QT	>500 ms	

CRITERIA for POTENTIALLY CLINICALLY SIGNIFICANT ABNORMALITIES
for phase 2/3 studies (oncology excepted)

Parameter	PCSA	Comments
QTc	Absolute values (ms)<br><br>>450 ms<br>>480 ms<br>>500 ms<br><br>Increase from baseline<br>Increase from baseline]30-60] ms<br>Increase from baseline >60 ms	To be applied to any kind of QT correction formula.<br>Absolute values categories are cumulative<br><br>QTc >480 ms and ΔQTc>60 ms are the 2 PCSA categories to be identified in individual subjects/patients listings.

Appendix B List of MedDRA terms for CMQs

Table 10 – CMQ "Type 1 or Type 2 diabetes"

MedDRA Term Label	Preferred Term Code
Diabetes mellitus	10012601
Diabetes mellitus inadequate control	10012607
Insulin resistant diabetes	10022491
Diabetes mellitus malnutrition-related	10050197
Diabetes mellitus management	10051599
Insulin-requiring type 2 diabetes mellitus	10053247
Type 1 diabetes mellitus	10067584
Type 2 diabetes mellitus	10067585
Fulminant type 1 diabetes mellitus	10072628

Table 11 – Selected PTs from SMQ "Optic nerve disorders" including in the CMQ for neurologic events

MedDRA Term Label	Preferred Term Code
Benign neoplasm of optic nerve	10057424
Optic atrophy	10030910
Optic discs blurred	10030923
Optic nerve disorder	10061322
Optic nerve injury	10030938
Optic nerve neoplasm	10053645
Optic nerve operation	10053272
Optic neuropathy	10061323
Papillitis	10033708
Pseudopapilloedema	10037141
Subacute myelo-opticoneuropathy	10058009
Toxic optic neuropathy	10044245
Visual evoked potentials abnormal	10047549
Amaurosis fugax	10001903
Blindness	10005169
Blindness unilateral	10005186
Colour blindness acquired	10010051

MedDRA Term Label	Preferred Term Code
Colour vision tests abnormal	10010056
Cranial nerve injury	10061094
Delayed myelination	10076456
Fundoscopy abnormal	10017520
Hemianopia	10019452
Hemianopia heteronymous	10019455
Hemianopia homonymous	10019456
Loss of visual contrast sensitivity	10064133
Neuro-ophthalmological test abnormal	10029256
Night blindness	10029404
Ophthalmological examination abnormal	10056836
Optic pathway injury	10030949
Optical coherence tomography abnormal	10073561
Quadranopia	10075427
Visual acuity reduced	10047531
Visual acuity reduced transiently	10047532
Visual acuity tests abnormal	10047534
Visual field defect	10047555
Visual field tests abnormal	10047567
Visual impairment	10047571
Visual pathway disorder	10061411

Table 12 - CMQ “Neurocognitive disorders – FDA’s recommendation”

MedDRA level	MedDRA Code	MedDRA Term Label
PTCD	10001949	Amnesia
PTCD	10061423	Amnestic disorder
PTCD	10002711	Anterograde Amnesia
PTCD	10066842	Behavioural and Psychiatric Symptoms of Dementia
PTCD	10008398	Change in sustained attention
LLTCD	10009843	Cognitive Deterioration
PTCD	10057668	Cognitive Disorder
LLTCD	10010300	Confusion
LLTCD	10048321	Confusion Aggravated

MedDRA level	MedDRA Code	MedDRA Term Label
PTCD	10010305	Confusional State
PTCD	10012218	Delirium
PTCD	10012267	Dementia
PTCD	10012271	Dementia Alzheimer's type
LLTCD	10012290	Dementia Nos
LLTCD	10012291	Dementia Nos Aggravated
LLTCD	10012292	Dementia of the Alzheimer's type NOS
PTCD	10067889	Dementia with Lewy Bodies
PTCD	10013395	Disorientation
PTCD	10013496	Disturbance in attention
PTCD	10070246	Executive dysfunction
PTCD	10068968	Frontotemporal Dementia
LLTCD	10058669	Global Amnesia
PTCD	10021402	Illogical Thinking
PTCD	10071176	Impaired reasoning
PTCD	10021630	Incoherent
PTCD	10023236	Judgement impaired
PTCD	10027175	Memory Impairment
PTCD	10027374	Mental Impairment
LLTCD	10027376	Mental Impairment Nos
LLTCD	10048345	Mental State Abnormal Aggravated
PTCD	10048294	Mental Status Changes
PTCD	10065424	Mini Mental Status Examination Abnormal
PTCD	10036631	Presenile Dementia
PTCD	10038965	Retrograde Amnesia
PTCD	10039966	Senile Dementia
LLTCD	10039967	Senile Dementia Nos
LLTCD	10040602	Short-term Memory Loss
PTCD	10043431	Thinking Abnormal
LLTCD	10043438	Thinking Slowed
PTCD	10044380	Transient Global Amnesia
PTCD	10057678	Vascular Dementia

Appendix C Derivation of the efficacy endpoints/components

The objective of this appendix is to detail the rules to derive the efficacy endpoints/components as per CEC and as per investigator. Rules describe the derivation of the type of the events, but also the date of the events that will be used for analyses using time to event approach.

A) DERIVATION OF CARDIOVASCULAR EVENTS AS PER CEC

1. Event “Non-fatal MI”

“Non-fatal MI” includes non-fatal MI or unstable angina adjudicated as MI (excluding events adjudicated as silent MI).

For patients who died with acute MI as primary cause of death as per adjudication, the last MI (excluding silent MIs) among those confirmed by adjudication and that occurred within 30 days before the death will not be considered as a “non-fatal MI”, but as a “fatal MI” included in the “CHD death” category.

The date of event that will be considered in all analyses is the onset date of the MI.

2. Event “Non-fatal ischemic stroke and fatal ischemic stroke”

“Non-fatal ischemic stroke and fatal ischemic stroke” includes strokes adjudicated as ischemic stroke or, as stroke “not otherwise specified”.

The date of event that will be considered in the analysis is the onset date of the stroke.

3. Event “Unstable angina requiring hospitalization”

“Unstable angina requiring hospitalization” includes events adjudicated as UA requiring hospitalization.

The date of the event that will be considered in the analyses is the onset date of the UA.

4. Event “CHD Death”

“CHD death” includes deaths adjudicated as due to Coronary Heart Disease and deaths adjudicated with an “Undetermined” primary cause of death.

The date of event that will be considered in the analysis of “Time to CHD death” is the date of death.

In the analysis of the primary efficacy endpoint, fatal MIs will be included in the category "CHD death", therefore the date of the CHD death that will be considered to determine the first event among the events included in the composite is:

- The date of onset of the "fatal MI" (see above in section A.1.) if the primary cause of death is "Acute myocardial infarction"
- The date of death otherwise

The same date for CHD death will be used for the analyses on "Time to any CHD event" and "Time to any major CHD event".

5. Event "Ischemia-driven coronary revascularization procedure"

"Ischemia-driven coronary revascularization procedure" includes PCI and CABG procedures with "Acute coronary syndrome" or "New-progressive symptoms of chronic ischemia or new progressive functional test abnormality that occurred since randomization" as reason for revascularization AND "De-novo lesion" and/or "Stent thrombosis" as type of lesions that required revascularization (ie, procedures performed only to treat restenosis lesion(s) are excluded from the endpoint).

The date of the event that will be considered in the analyses is the date of the revascularization procedure.

6. Event "CV Death"

"CV death" includes deaths with primary cause of death adjudicated as "Cardiovascular" or "undetermined".

In the analysis of "Time to any CV event" (any non-fatal CHD event, any CV death, or non-fatal ischemic stroke), fatal MIs and fatal strokes will be included in the category "CV death", therefore the date of the CV death that will be considered to determine the first event among the events included in the composite is:

- The date of onset of the "fatal MI" (see above in section A.1.) if the primary cause of death is "Acute myocardial infarction"
- The date of onset of the fatal ischemic stroke if the primary cause of death is "Stroke – ischemic" or "Stroke – Undetermined". The fatal ischemic stroke will be the last stroke among those confirmed by adjudication that occurred within 30 days before the death
- The date of death for other causes of death

7. Event "Congestive heart failure requiring hospitalization"

"CHF requiring hospitalization" includes events confirmed by adjudication.

The date of the event that will be considered in the analyses will be the onset date of the CHF event.

8. Event "All-cause mortality"

"All-cause mortality" includes all adjudicated deaths.

The date of event that will be considered in the analysis of "Time from randomization to death (all-cause mortality)" is the date of death.

In the analysis of "Time to all-cause mortality, non-fatal MI, non-fatal ischemic stroke", fatal MIs and fatal strokes will be included in the category "all-cause mortality", therefore the date of all-cause mortality that will be considered to determine the first event among the events included in the composite is:

- The date of onset of the "fatal MI" (see above in section A.1.) if the primary cause of death is "Acute myocardial infarction"
- The date of onset of the fatal ischemic stroke (see above in section A.2.) if the primary cause of death is "Stroke – ischemic" or "Stroke – Undetermined"
- The date of death for other causes of death

B) DERIVATION OF CARDIOVASCULAR EVENTS AS PER INVESTIGATOR

Endpoints as per investigator's opinion will be derived for the sensitivity analysis of the primary efficacy endpoint.

1. Event "Non-fatal MI"

"Non-fatal MI" includes all events reported, with a final diagnosis as per investigator of "Spontaneous, non-procedural MI", "peri-PCI MI", or "peri-CABG MI.

For patients who died with acute MI as primary cause of death as per investigator, the last MI reported and that occurred within 30 days before the death will not be considered as a "non-fatal MI", but is a "fatal MI" included in the "CHD death" category.

The date of event that will be considered in the analysis is the date of onset of ischemic symptoms that caused the subject to seek medical attention, reported by the investigator in the e-CRF specific form (could be slightly different from the date as per CEC).

2. Event “Non-fatal ischemic stroke and Fatal ischemic stroke”

“Non-fatal ischemic stroke and fatal ischemic stroke” includes all cerebrovascular events reported, with a final diagnosis as per investigator of “ischemic stroke”, “ischemic stroke with hemorrhagic conversion”, or “undetermined stroke”.

The date of event that will be considered in the analysis is the date of new or worsening neurological symptoms, reported by the investigator in the e-CRF specific form (could be slightly different from the date as per CEC).

3. Event “Unstable angina requiring hospitalization”

The definition of “Unstable angina requiring hospitalization” in the protocol of the Outcomes study is more restrictive than the unstable angina the investigators had to report in the e-CRF to allow all potential UA to be adjudicated. Therefore a subset of all the UA reported by the investigator will be selected for the analysis, as follows:

“Unstable angina requiring hospitalization” includes all events reported by the investigator, with a final diagnosis of “unstable angina”, and in addition with:

- The presence of an hospitalization (with discharge not before the next calendar day) that may correspond to the event, and;
- New ECG findings (Persistent ST elevation, ST depression, T wave inversion, New LBBB, Q waves, or Other findings), and;
- Contemporary evidence of angiographically significant coronary disease (PCI/CABG performed to treat at least one significant coronary lesion, or Diagnostic catheterization with at least one lesion >70% that is not a restenosis at previous PCI site)

In the absence of those criteria, since an elevation of cardiac biomarkers observed within 48H of the event may be the sign of a possible MI (STEMI or non STEMI), the event will be also considered as a possible composite from the primary endpoint.

The date of the event that will be considered in the analysis is the date of onset of ischemic symptoms that caused the subject to seek medical attention, reported by the investigator in the e-CRF specific form (could be slightly different from the date as per CEC).

4. Event “CHD Death”

Since the categorization of deaths as CHD death was not requested to the Investigator, we will apply the convention below for “CHD death” as per investigator’s opinion:

“CHD deaths” as per investigator include deaths due to “Acute myocardial infarction”, “Sudden cardiac death”, “Heart failure or cardiogenic shock”, and deaths with an “Undetermined” primary cause of death.

(electronic 1.0)

In the sensitive analysis of the primary efficacy endpoint as per investigator, fatal MIs will be included in the category “CHD death”, therefore the date of the CHD death that will be considered to determine the first event among the events included in the composite is:

- The date of onset of ischemic symptoms that caused the subject to seek medical attention of the “fatal MI” (see above in section B.1.) if the primary cause of death as per investigator is “Acute myocardial infarction”
- The date of death otherwise.

Appendix D EQ-5D Patient Questionnaire

[redacted]

2

© 1998 EuroQol GroupEQ-5D™ is a trade mark of the EuroQol Group

Appendix E EQ-5D utility score algorithm

[redacted]

Appendix F Detailed statistical methodology for pattern-mixture model

A pattern-mixture model approach will be used for change in LDL_C at each timepoint, with a different imputation strategy applied for missing LDL-C values during the treatment period (ie, within the time period from the first double-blind investigational medicinal product [IMP] injection up to the day of the last double-blind injection +21 days) and missing LDL C values after treatment discontinuation (ie, after the day of last double-blind injection +21 days) based on the following assumptions:

- Patients within 21 days of their last IMP injection would continue to show benefit from treatment similar to that observed at the scheduled time point. Therefore, LDL C values missing during the treatment period will be considered “Missing At Random” and imputed using a model estimated using all samples collected on treatment.
- Patients who stopped taking their study treatment no longer benefited from it in the future, and thus tended to have LDL-C values returning to baseline. Thus LDL-C values missing after treatment discontinuation will be imputed based on patient’s own baseline value.

The assumptions for this approach were based on the following considerations:

- Missing values during the treatment period are mostly consecutive to:
  - Visits performed outside of the pre-specified time-window;
  - No blood sample available although visit was done;
  - LDL-C not measurable due to technical reasons.

  In addition, these missing data were often intermittent, ie, followed by LDL-C values collected at subsequent visits. It was therefore considered reasonable to assume that these missing data were “At Random”.
- Phase 2 studies DFI11565 and R727-CL-1003 included a prospective assessment of calculated LDL-C during the follow-up period after a 12-week treatment period. These studies showed that after treatment discontinuation, the average calculated LDL-C returned to baseline level within 4 weeks after ceasing alirocumab treatment (see Figure 1 and Figure 2).

Figure 1 - Study DFI11565: calculated LDL-C mean (+/- SE) percent change from baseline

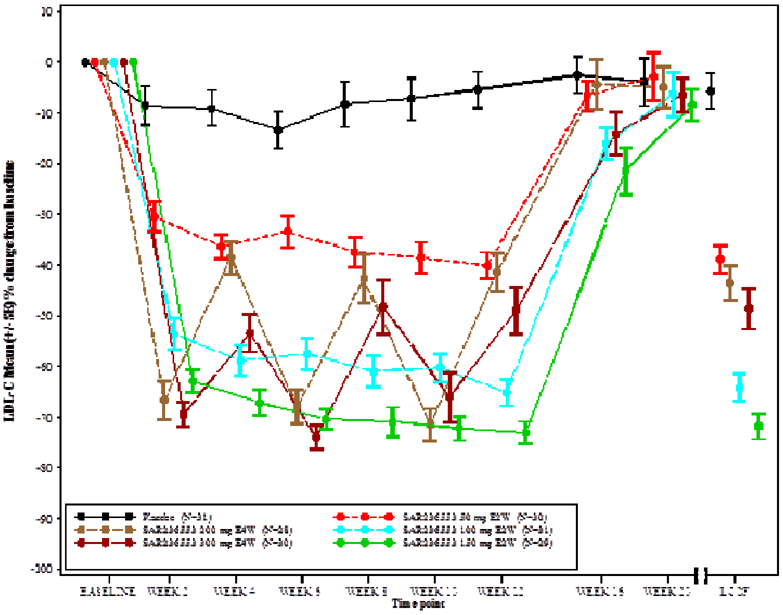

Figure 2 - Study R-727-CL-1003: calculated LDL-C mean (+/- SE) percent change from baseline

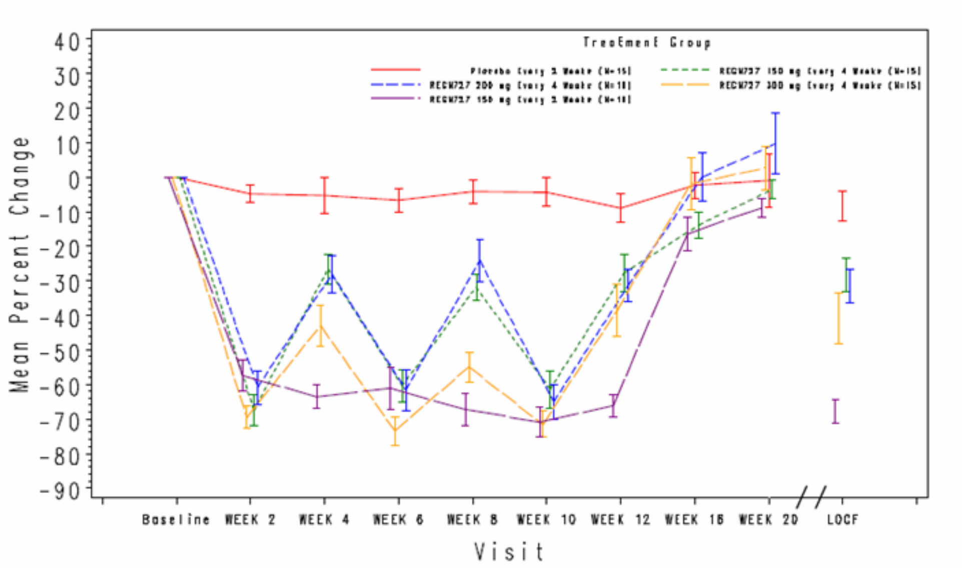

Missing LDL-C values will be imputed 10 times to generate 10 complete data sets. For each planned time point, the percent changes from baseline will be derived from observed and imputed LDL-C at this time point. The completed data sets will be analyzed using an ANCOVA model with treatment group as fixed effect, and the baseline LDL-C value as continuous covariate. The

results from the 10 analyses will be combined using Rubin's formulae. If necessary, the number of imputations (10) will be increased until stable estimates are obtained.

Imputation of missing data during the treatment period

Missing LDL-C values during the treatment period will be imputed from other on-treatment measurements assuming Missing At Random, using SAS® MI procedure.

Only LDL-C values collected during the treatment period will be included in the imputation model. This way, missing LDL-C values during the treatment period will be imputed based solely on observed on-treatment LDL-C values.

The imputation model will be estimated within each treatment arm and include baseline LDL-C value, and all LDL-C values at pre-specified visits. Since the pattern of missing data will necessarily be non-monotone, a Monte-Carlo Markov Chain (MCMC) method will be used.
A minimum value of 0 will be specified in order to avoid negative imputed LDL-C values.

A sample SAS code is provided below:

```
proc mi data=DATAIN out=DATAOUT nimpute=10 minimum=0;
  var LDL_BASE LDL_M1 LDL_M2 LDL_M4 LDL_M8 …;
by ARM;
run;
```

As stated above, the input dataset DATAIN will include only LDL-C values collected during the treatment period. Any LDL-C values collected during the post-treatment period will be excluded from the input dataset. In practice, the MI procedure will generate imputed values for all missing values (whether on-treatment or post-treatment), but only imputed values during the treatment period will be kept in the final datasets that will be analyzed. Imputed values during the post-treatment period will be discarded and replaced by imputed values described in the next paragraph.

Imputation of missing data after treatment discontinuation

Missing LDL-C values during the post-treatment period will be imputed assuming LDL C values would on average return to baseline values.

For each patient, missing post-treatment LDL-C values will be imputed 10 times, using a random draw from a normal distribution, with mean equal to patient's own baseline value and variance equal to the conditional variance at the specific time-point, given the baseline value.

Let Y_0 and Y_1 denote the LDL-C at baseline and at the specific time-point respectively. Since Y_0 and Y_1 are assumed to have a bivariate normal distribution, the conditional variance of Y_1 given Y_0 is:

$$Var(Y_1 | Y_0 = y_0) = \sigma_1^2 (1 - \rho^2)$$

Where σ_1^2 denotes the variance of Y1 and ρ the coefficient of correlation between Y_0 and Y_1.

The conditional variance will be estimated from observed data within the same treatment arm at the specific time-point.

During the random generation process, a minimum value of 0 will also be applied in order to avoid negative imputed LDL-C values.

The same methodology will be applied to all lipid parameters, using log-transformation of variables for TGs and Lp (a).

EFC11570 16.1.9 Statistical analysis plan

ELECTRONIC SIGNATURES

Signed by	Meaning of Signature	Server Date (dd-MMM-yyyy HH:mm)
[redacted]		

STATISTICAL ANALYSIS PLAN

A Randomized, Double-Blind, Placebo-Controlled, Parallel-Group Study to Evaluate the Effect of Alirocumab (SAR236553/REGN727) on the Occurrence of Cardiovascular Events in Patients who have Recently Experienced an Acute Coronary Syndrome.

ODYSSEY Outcomes

SAR236553/REGN727-EFC11570

STATISTICIAN: [redacted]

BIOSTATISTICS PROJECT LEADER: [redacted]

DATE OF ISSUE: 11-Oct-2017

Total number of pages: 89

Any and all information presented in this document shall be treated as confidential and shall remain the exclusive property of Sanofi (or any of its affiliated companies). The use of such confidential information must be restricted to the recipient for the agreed purpose and must not be disclosed, published or otherwise communicated to any unauthorized persons, for any reason, in any form whatsoever without the prior written consent of Sanofi (or the concerned affiliated company); 'affiliated company' means any corporation, partnership or other entity which at the date of communication or afterwards (i) controls directly or indirectly Sanofi, (ii) is directly or indirectly controlled by Sanofi, with 'control' meaning direct or indirect ownership of more than 50% of the capital stock or the voting rights in such corporation, partnership or other entity

TABLE OF CONTENTS

STATISTICAL ANALYSIS PLAN 1

TABLE OF CONTENTS 2

LIST OF ABBREVIATIONS AND DEFINITION OF TERMS 5

1 OVERVIEW AND INVESTIGATIONAL PLAN 7

1.1 STUDY DESIGN AND RANDOMIZATION 7

1.2 OBJECTIVES 8
1.2.1 Primary objective 8
1.2.2 Secondary objectives 8

1.3 DETERMINATION OF SAMPLE SIZE 8

2 STUDY PLAN 10

2.1 MODIFICATIONS TO THE STATISTICAL SECTION OF THE PROTOCOL 11

2.2 STATISTICAL MODIFICATIONS MADE IN THE STATISTICAL ANALYSIS PLAN 12

3 STATISTICAL AND ANALYTICAL PROCEDURES 15

3.1 ANALYSIS ENDPOINTS 15
3.1.1 Demographic and baseline characteristics 15
3.1.2 Prior or concomitant medications 19
3.1.3 Efficacy endpoints 20
3.1.3.1 Primary efficacy endpoint(s) 20
3.1.3.2 Secondary efficacy endpoint(s) 21
3.1.4 Safety endpoints 22
3.1.4.1 Adverse events variables 23
3.1.4.2 Deaths 25
3.1.4.3 Laboratory safety variables 25
3.1.4.4 Vital signs variables 26
3.1.4.5 Electrocardiogram variables 26
3.1.5 Other endpoints 26
3.1.5.1 Lipid parameters 26
3.1.5.2 hs-CRP 27
3.1.5.3 HbA_{1C} 27
3.1.5.4 Patients with LDL-C <25 mg/dL (0.65 mmol/L) 27
3.1.5.5 Anti-alirocumab antibodies assessed throughout the study. 28
3.1.5.6 Quality-of-life parameters 28

3.1.5.7 Cardiovascular events of interest (other than efficacy endpoints) 29
3.1.6 Pharmacokinetic variables 29
3.1.7 Pharmacogenomics endpoints....29
3.2 DISPOSITION OF PATIENTS 30
3.2.1 Randomization and drug dispensing irregularities 31
3.3 ANALYSIS POPULATIONS 32
3.3.1 Efficacy populations 32
3.3.1.1 Intent–to-treat population 32
3.3.2 Safety population....33
3.3.3 Anti-alirocumab antibody population 33
3.4 STATISTICAL METHODS 33
3.4.1 Demographics and baseline characteristics 33
3.4.2 Prior, concomitant or post-treatment medications 34
3.4.3 Extent of investigational medicinal product exposure and compliance....36
3.4.3.1 Extent of investigational medicinal product exposure 36
3.4.3.2 Compliance 37
3.4.4 Analyses of efficacy endpoints....37
3.4.4.1 Analysis of primary efficacy endpoint(s)....37
3.4.4.2 Analyses of secondary efficacy endpoints 41
3.4.4.3 Multiplicity issues....41
3.4.4.4 Additional efficacy analyses 41
3.4.5 Analyses of safety data 41
3.4.5.1 Analyses of adverse events 43
3.4.5.2 Deaths....49
3.4.5.3 Analyses of laboratory variables 50
3.4.5.4 Analyses of vital sign variables 53
3.4.6 Analyses of other safety parameters....53
3.4.7 Analyses of other endpoints....54
3.4.7.1 Analyses of hs-CRP 54
3.4.7.2 Analyses of HbA_{1c} 54
3.4.7.3 Analyses of patients with LDL-C <25 mg/dL(<0.65 mmol/L)....54
3.4.7.4 Analyses of lipid parameters 54
3.4.7.5 Analyses of anti-alirocumab antibody variables 56
3.4.7.6 Analyses of quality of life/health economics variables 57
3.4.7.7 Analysis of cardiovascular events of interest (other than efficacy endpoint)....57
3.4.8 Analyses of pharmacokinetic and pharmacodynamic variables 57
3.5 DATA HANDLING CONVENTIONS....58
3.5.1 General conventions 58
3.5.2 Data handling conventions for secondary efficacy variables....59
3.5.3 Missing data 59

3.5.4 Windows for time points 61
3.5.5 Unscheduled visits 62
3.5.6 Pooling of centers for statistical analyses 62
3.5.7 Statistical technical issues 63

4 INTERIM ANALYSIS 64

5 DATABASE LOCK 66

6 SOFTWARE DOCUMENTATION........67

7 REFERENCES........68

8 LIST OF APPENDICES 69

APPENDIX A POTENTIALLY CLINICALLY SIGNIFICANT ABNORMALITIES (PCSA) CRITERIA........70

APPENDIX B LIST OF MEDDRA TERMS FOR CMQS........75

APPENDIX C DERIVATION OF THE EFFICACY ENDPOINTS/COMPONENTS........79

APPENDIX D EQ-5D PATIENT QUESTIONNAIRE........84

APPENDIX E EQ-5D UTILITY SCORE ALGORITHM 85

APPENDIX F DETAILED STATISTICAL METHODOLOGY FOR PATTERN-MIXTURE MODEL 86

LIST OF ABBREVIATIONS AND DEFINITION OF TERMS

Ab:	antibody
ACE:	angiotensin converting enzyme
ACS:	acute coronary syndrome
ADA:	anti-alirocumab antibodies
ADP:	adenosine diphosphate
AESI:	adverse event of special interest
ALT:	alanine aminotransferase
ANCOVA:	analysis of covariance
Apo:	apolipoprotein
AST:	aspartate aminotransferase
ATC:	Anatomical Therapeutic Chemical
BMI:	body mass index
CABG:	coronary artery bypass graft surgery
CEC:	Clinical Events Committee
CHD:	coronary heart disease
CI:	confidence interval
CMQ:	company MedDRA query
CPK:	creatine phosphokinase
CSED:	common study end date
CV:	cardiovascular
DMC:	data monitoring committee
DVT:	deep vein thrombosis
ECG:	electrocardiogram
e-CRF:	electronic case report form
eGFR:	estimated glomerular filtration rate
HbA_{1c}:	glycated hemoglobin A1c
HCV:	hepatitis C virus
HDL-C:	high-density lipoprotein cholesterol
HLGT:	high level group term
HLT:	high level term
HR:	hazard ratio
hs-CRP:	high-sensitivity C-reactive protein
IA:	interim analysis
IMP:	investigational medicinal product
ITT:	intent-to-treat
IVRS:	interactive voice reponse system
IWRS:	interactive web response system
LDH:	lactate dehydrogenase
LDL-C:	low-density lipoprotein cholesterol
LLN:	lower limit of normal

LLOQ:	lower limit of quantification
LMT:	lipid modifying therapy
LMWH:	low molecular weight heparin
Lp (a):	lipoprotein (a)
MAR:	missing-at-random
MDRD:	Modification of the Diet in Renal Disease
MedDRA:	Medical Dictionary for Regulatory Activities
MI:	myocardial infarction
MMRM:	mixed-effect model with repeated measures
NOD:	New onset of diabetes
NSAID:	nonsteroidal anit-inflammatory drug
NSTEMI:	non ST segment elevation myocardial infarction
PCI:	percutaneous coronary intervention
PCSA:	potentially clinically significant abnormality(ies)
PE:	pulmonary embolism
PT:	preferred term
Q1:	first quartile
Q2W:	every 2 weeks
Q3:	third quartile
RNA:	ribonucleic acid
SAE:	serious adverse event
SAP:	statistical analysis plan
SD:	standard deviation
SE:	standard error
SMQ:	standardized MedDRA query
SOC:	system organ class
SPERT:	safety planning, evalutation, and reporting team
STEMI:	ST segment elevation myocardial infarction
TC:	total cholesterol
TEAE:	treatment-emergent adverse event
TG:	triglycerides
TIA:	transient ischemic attack
UA:	unstable angina
UFH:	unfractionated heparin
ULN:	upper limit of normal range
ULOQ:	upper limit of quantification
WHO-DD:	World Health Organization-Drug Dictionary
γGT:	gamma glutamyl transferase

1 OVERVIEW AND INVESTIGATIONAL PLAN

1.1 STUDY DESIGN AND RANDOMIZATION

This is a double-blind, randomized, placebo-controlled, balanced (1:1, alirocumab:placebo), parallel-group, multi-national, multicenter study.

Randomization takes place 4 weeks to 52 weeks after the index event and is stratified according to country.

Prior to randomization, eligible patients enter a run-in period of at least 2 weeks but no more than 16 weeks, during which they receive statin-intensive therapy defined as daily atorvastatin 40 or 80 mg, or rosuvastatin 20 or 40 mg. In case patients are unable to tolerate atorvastatin 40/80 mg or rosuvastatin 20/40 mg, they are allowed to receive the maximal tolerated dose of atorvastatin or rosuvastatin; or under some documented circumstances for statin-intolerant patients, receive other lipid lowering treatment other than a statin (eg, ezetimibe, or other non-statin lipid modifying therapy [LMT]), or no LMT at all.

Following this run-in period, only patients not reaching goal on their current LMT at the qualifying visit, ie, LDL-C ≥70 mg/dL (≥1.81 mmol/L) or apolipoprotein B (Apo B) ≥80 mg/dL (≥0.8 g/L) or non-HDL-C ≥100 mg/dL (≥2.59 mmol/L), are randomized to either background therapy + alirocumab or background therapy + placebo. All patients randomized to alirocumab initially receive alirocumab 75 mg every 2 weeks (Q2W). After randomization, patients on alirocumab not reaching the target LDL-C level at Month 1 have their dose up-titrated to 150 mg Q2W at Month 2 in a blinded fashion (in case Month 1 LDL-C is not available or not valid for potential up-titration at Month 2, the next available LDL-C sample at Month 2 is used for potential up-titration at Month 4).

The double-blind treatment period will continue until 24 months after the closing of randomization for all countries except for China or until the target number of events (1613) is reached, whichever comes last (this date will be the Common Study End Date [CSED]). The corresponding estimated study duration is 64 months. All patients, even if they have achieved an endpoint, or have prematurely discontinued study treatment, will be asked to remain in the study until the CSED and to come back to the site as close as possible to the CSED (ie, CSED visit).

1.2 OBJECTIVES

1.2.1 Primary objective

The primary objective of this study is to compare the effect of alirocumab with placebo on the occurrence of cardiovascular events (composite endpoint of coronary heart disease [CHD] death, non-fatal myocardial infarction [MI], fatal and non-fatal ischemic stroke, unstable angina requiring hospitalization) in patients who have experienced an acute coronary syndrome (ACS) event 4 to 52 weeks prior to randomization and are treated with an LMT regimen that is statin-intensive (defined as atorvastatin 40 or 80 mg, or rosuvastatin 20 or 40 mg) or at maximally tolerated dose of these given statins, and optimized for long-term chronic use with other non-statin LMT(s) at Investigator's discretion.

1.2.2 Secondary objectives

The secondary objectives are:

To compare the efficacy of alirocumab versus placebo on secondary endpoints (any CHD event, major CHD event, any cardiovascular [CV] event, composite of all-cause mortality/non-fatal MI/ non-fatal ischemic stroke, all-cause mortality);

- To evaluate the safety and tolerability of alirocumab throughout the study;
- To evaluate the development of anti-alirocumab antibodies;
- To evaluate the effect of alirocumab on low-density lipoprotein cholesterol (LDL-C), apolipoprotein B (Apo B), and non-high-density lipoprotein cholesterol (non-HDL-C).

A Clinical Events Committee (CEC) is established to adjudicate, in a blinded fashion, events corresponding to the primary and secondary cardiovascular endpoints as well as all causes of death.

1.3 DETERMINATION OF SAMPLE SIZE

The sample size calculations are based on the primary efficacy variable, with the following assumptions:

- Primary efficacy endpoint is the time from randomization to the date of first occurrence of one of the following clinical events, as determined by the CEC: CHD death, any non-fatal MI, fatal or non-fatal ischemic stroke, or unstable angina requiring hospitalization (with new high-risk electrocardiogram (ECG) findings and contemporary evidence of angiographically significant coronary disease). Based on PROVE-IT results (1) and considering adjustment based on the study design and endpoints definition, the following Kaplan-Meier probabilities of event in the placebo group have been assumed: 3.8% at 12 months, 6.4% at 24 months, 9.0% at 36 months, and 11.4% at 48 months. Probabilities of an event at other time points are estimated using a piece-wise exponential model. The primary efficacy endpoint will be analyzed on an intent-to-treat basis (all randomized

patients, including those who discontinue study medication are followed for any efficacy event until the CSED).

- Treatment hazard ratio of 0.85 (corresponding to a 15% hazard risk reduction for the test group relative to placebo), which is assumed to be constant over time;
- A log-rank test at an overall 1-sided 2.5% significance level with 90% power;
- Two interim analyses, according to a group sequential design, using for efficacy Gamma (-22) α-spending function, and for futility a Gamma (-5) β-spending function. Non-binding spending functions are used. See Section 4 for interim analysis details;
- One percent lost-to-follow-up prior to a primary efficacy endpoint at 24 months in both arms;
- Enrolment rate: sample size of 18 000 patients enrolled in 1400 sites (all sites were expected to be activated over a 12 months period). Table 1 below describes the expected enrolment rate per month; these assumptions were based on internal experience to enroll this patient population.

Table 1 - Enrolment rate assumptions per month

Month	1 to 3	4	5	6	7	8	9	10	11	12 to 40
No. patients per month	32	64	80	104	160	240	320	420	520	560
Cumulative no. patients	96	160	240	344	504	744	1064	1484	2004	2564 at M12, 18 000 at M40

Based on the above assumptions, 1613 events are needed for 90% power. In order to achieve the 1613 targeted events, 18 000 patients (9000 per group) were initially planned to be randomized, over a period of about 40 months. However taking into account the local situation in China (regulatory requirement to randomize at least 600 patients, and anticipated delay in study start in China), the total number of patients randomized may be increased to approximately 18 600 patients (~9300 per group). At the end of the study, the overall population will include approximately 18 000 randomized patients who have either died or have been followed for a minimum of 24 months, possibly supplemented with an additional subset of Chinese patients (~600) who may be followed for less than 24 months.

2 STUDY PLAN

The following figure presents graphically the study design:

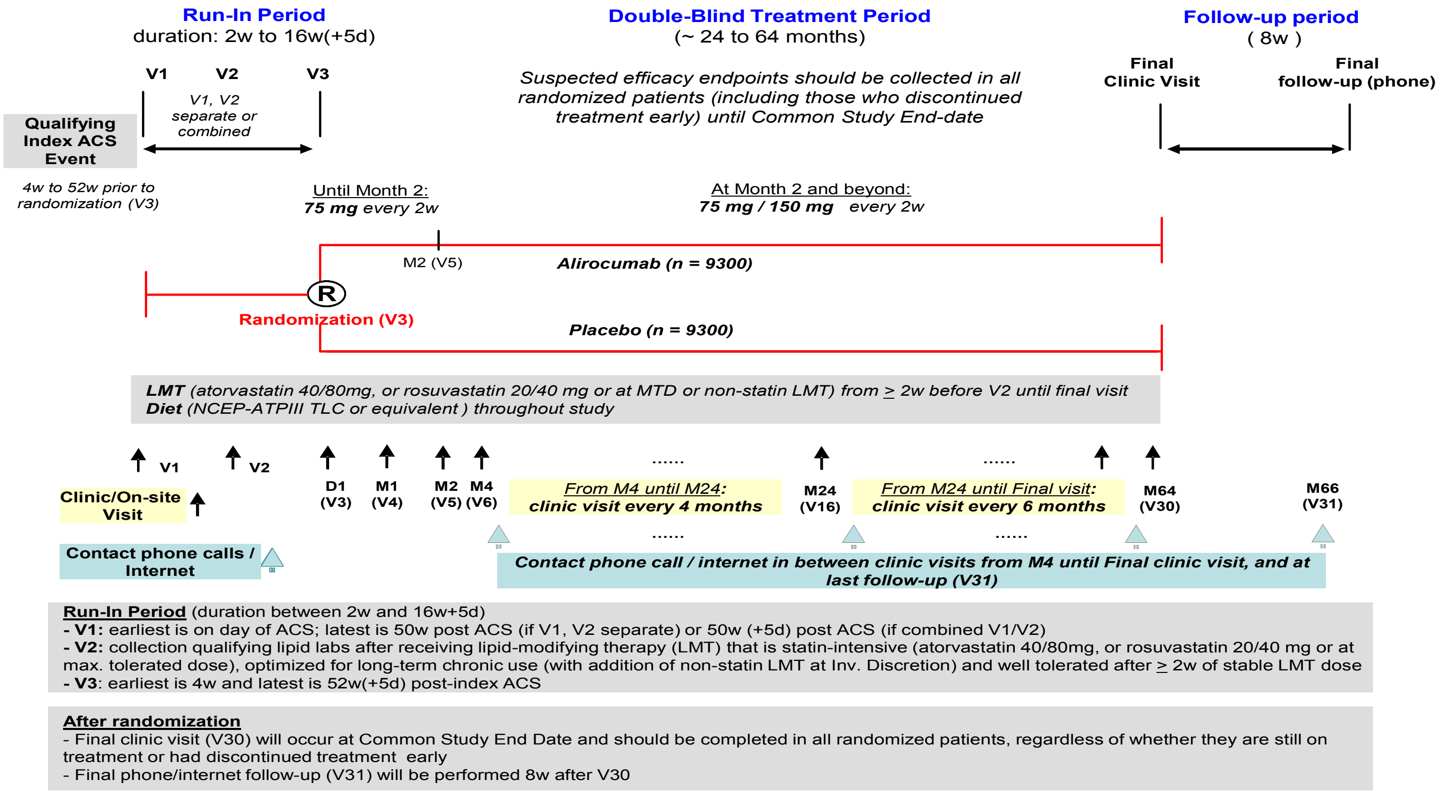

ACS: acute coronary syndrome, LMT: lipid-modifying therapy, MTD: maximal tolerated dose

The study flow chart is detailed in the protocol.

2.1 MODIFICATIONS TO THE STATISTICAL SECTION OF THE PROTOCOL

This section summarizes major changes to the protocol statistical section with emphasis on changes made after study start (after the first patient was enrolled).

The protocol history table below gives the timing, rationale, and key details of major changes to the protocol statistical section.

The first patient was randomized on 02 November 2012.

Table 2 - Protocol amendment statistical changes

Amendment Number	Date approved	Rationale	Description of statistical changes
6	05-Dec-2013	Clarification of definition of secondary endpoints in the protocol	The secondary endpoint of “Any hospitalization for unanticipated coronary revascularization procedure” was replaced with “Any ischemia-driven coronary revascularization procedure”
6	05-Dec-2013	Exclusion criteria in the protocol modified to allow inclusion of patients with a qualifying index ACS event occurring more than 16 weeks and less than 52 weeks prior to randomization	“Time from ACS event to randomization” in categorical variable (eg, 4-24 weeks, >24 weeks) is added as a subgroup factor in the Subgroups analyses of the primary efficacy endpoint
6	05-Dec-2013	The endpoint “Cardiovascular events of interest (other than efficacy endpoints)” has been added in the protocol.	The analysis of cardiovascular events of interest (other than efficacy endpoints) is added.
8	16-Apr-2015	Sample size may be increased up to approximately 18 600 patients to allow the inclusion of 600 Chinese patients because of the local delayed study start in China, and definition of the CSED updated	CSED definition modified to “when 1613 patients have experienced at least one primary efficacy event or 24 months after the closing of randomization for all countries except China, whichever comes last”
8	16-Apr-2015	Neurologic events (including neurocognitive events) are added as AESI	Neurologic events (including neurocognitive events) added in the safety analysis

ACS = acute coronary syndrome; AESI = adverse event of special interest; CSED = common study end date

2.2 STATISTICAL MODIFICATIONS MADE IN THE STATISTICAL ANALYSIS PLAN

The statistical analysis plan history table below gives the timing, rationale, and key details for major changes to the statistical analysis features

- in the first version of the statistical analysis plan dated on 25 January 2016 compared to the protocol amendment 8 (version currently in effect at this time)
- in the amended versions of the statistical analysis plan (versions 2 and 3) compared to the first version of 25 January 2016.

Changes already incorporated in a protocol amendment are listed only in Table 2.

With regard to the study timelines, the first patient was enrolled on 02 November 2012. The first interim analysis occurred in April 2016 with the data cut-off for the interim analysis on 30 November 2015. The second interim analysis occurred when approximately 1210 patients had at least one positively-adjudicated primary efficacy endpoint, with review of the data by the DMC in November 2016.

Table 3 - Statistical analysis plan statistical changes

SAP version number	Date approved	Rationale	Description of statistical changes
1	25-Jan-2016	Imputation method not planned for missing lipid values	For patients without lipid values in the time window analyzed, multiple imputation will be used with different imputation strategies depending on the time of those missing values (during the treatment period or after treatment discontinuation) (see Section 3.4.7.4.1)
1	25-Jan-2016	End of analysis period for the analysis of TEAEs for patients with 2 consecutives low LDL-C did not take into account down-titrations from 150 mg to 75 mg	In the specific analysis of TEAEs for the subgroup of patients with two consecutive LDL-C <25 mg/dL (respectively 15 mg/dL) within the alirocumab treatment group, the upper limit of the analysis period for patients down-titrated from 150 mg to 75 mg will end at the date of last injection of 150 mg +70 days (see Section 3.4.5.1).
1	25-Jan-2016	Selection of patients with 2 consecutive LDL-C <25 mg/dL (respectively 15 mg/dL) modified consistently with the other studies of the program.	The patients will be considered as having 2 consecutive LDL-C <25 mg/dL (respectively 15 mg/dL) if these values are spaced out by at least 21 days (see Section 3.1.5.4).
2	28-Jul-2016	Category of patients without diabetes at baseline divided in two sub-categories, consistently with the ODYSSEY Phase 3a program.	The category of patients without diabetes at baseline will be sub-divided in two-sub-categories: “Pre-diabetes” and “normoglyceamic” (see Section 3.1.1). The transition to new onset of diabetes in these two subgroups will be described separately (see Section 3.4.5.3). The primary efficacy endpoint will also be assessed in these subgroups (see Section 3.4.4.1)

SAP version number	Date approved	Rationale	Description of statistical changes
2	28-Jul-2016	Per FDA's recommendation, definition of "New onset of diabetes" revised.	The fasting glucose criterion is included in the definition of "New onset of diabetes" (see Section 3.4.5.3). Details about handling of specific data situations are also added.
3	This version	To improve the identification of the diabetes status at baseline and during the course of the study (new onset of diabetes) for patients selected by anti-diabetic medications.	Outcome of the review by external diabetes experts considered in the definition of diabetes status for patients identified with anti-diabetic medications at baseline (see Section 3.1.1) and during the course of the study (see Section 3.4.5.3)
3	This version	Improve derivation of the censoring date for vital status	Additional dates are considered to derive the censoring date for CHD deaths, CV deaths and all causes of deaths endpoints (see Section 3.1.3.2).
3	This version	Include CHD death and CV death endpoints in the hierarchical testing to have a more robust assessment on these efficacy endpoints.	CHD death endpoint is moved from other secondary efficacy endpoints to main secondary efficacy endpoints. In addition CV death endpoint is added in the main secondary endpoints (see Section 3.1.3.2.1).
3	This version	Provide comprehensive assessment of diabetes.	"New onset of diabetes" added in the list of events of interest (see Section 3.1.4.1)
3	This version	Clarification about the list of patients who will have an additional ADA sample after the end of the study.	See Section 3.1.5.5
3	This version	Explore treatment effects on CV events before and after defined timepoints.	Analysis with Cox Proportional hazard models, exploring if the treatment HR varies over time, added in additional analyses on CV events (see Section 3.4.4.4).
3	This version	Avoid overlap between alirocumab period and placebo period for patients switched to placebo with incomplete/missing last active injection date.	Imputation of last active injection if incomplete/missing changed from the day to the day before the first injection of placebo following the switch to placebo in IVRS (see Section 3.5.3)

SAP = statistical analysis plan; TEAE = treatment-emergent adverse event;

3 STATISTICAL AND ANALYTICAL PROCEDURES

3.1 ANALYSIS ENDPOINTS

3.1.1 Demographic and baseline characteristics

Unless otherwise specified, the baseline value is defined as the last available value obtained up to the first double-blind IMP injection.

For patients randomized and not treated, the baseline value will be the last available value on or before the day of randomization.

All baseline safety and efficacy parameters (apart from those listed below) will be presented along with the summary statistics in the safety and efficacy sections (Section 3.4.5 and Section 3.4.4).

Demographic characteristics

Demographic variables are:

- Age in years (quantitative and qualitative variable: <65, ≥65 to <75, and ≥75 years; and <65, ≥65 years);
- Gender (Male, Female);
- Race (White, Black or African American, Asian, American Indian or Alaska Native, Native Hawaiian or other Pacific Island, other);
- Ethnicity (Hispanic or Latino, Not Hispanic or Latino);
- Region (North America, South America, Western Europe, Eastern Europe, Asia, Rest of the world as defined in Section 3.5.6).

Medical or surgical history

This information will be coded using the version of Medical Dictionary for Regulatory Activities (MedDRA) currently in effect at Sanofi at the time of database lock.

Medical or surgical history includes:

- Medical history of allergies;
- Patient's family medical allergic history;
- Other relevant medical or surgical history;
- Smoking habits;
- Alcohol habits.

Medical history of specific interest includes:

- Coronary artery disease history prior to the index ACS event;
- Cardiovascular risk factors including:
  - Dyslipidemia;
  - Hypertension;
  - Family history of coronary artery disease;
  - Type 1 or Type 2 diabetes mellitus.
- Other cardiovascular disease, including:
  - Congestive heart failure;
  - Peripheral arterial disease;
  - Cerebrovascular disease (carotid endarterectomy/carotid stenting, prior stroke, transient ischemic attack).

In addition, the status of diabetes mellitus at baseline and pre-diabetes at baseline will be derived using the following definitions:

<u>Diabetes mellitus:</u>

- Type 1 or Type 2 diabetes reported in medical history or as an adverse event before baseline (ie, before the first IMP intake or randomization for non-treated patients) (using company MedDRA query [CMQ] "Type 1 or Type 2 diabetes" as detailed in Appendix B, Table 11)
- And/or HbA_{1c} ≥6.5% at baseline (V3) (or at V1 if V3 is not available)
- And/or 2 values of fasting blood glucose ≥126 mg/dL (7.0 mmol/L) (at V1 and V3)
- And/or Use of anti-diabetic medication before baseline with a confirmed diagnosis per the external diabetes experts* (in case a partial start date for a given medication precludes determining whether it started prior or after baseline, the diabetes status will not consider anti-diabetic medications for the concerned patients).

  * Patients classified as diabetic based only on the use of anti-diabetic medication before baseline will be reviewed in a blinded manner by external experts in diabetology. The individual Diabetes cases will be reviewed based on available information (e.g., medical history, concomitant medications, concomitant AEs, laboratory data, including complementary investigations as relevant). If the diabetic mellitus status is not confirmed, the patients will be classified as pre-diabetes or normoglyceamic according to the definitions below.

Pre- diabetes:

- Specific terms (CMQ "impaired glucose control" as detailed in Appendix B Table 12) reported in the medical history or as an adverse event before baseline (ie, before the first IMP intake or randomization for non-treated patients);
- And/or HbA1c ≥5.7% and <6.5% at baseline (V3) (or at V1 if V3 is not available)
- And/or two values of fasting glucose (at V1 and V3) ≥100 mg/dL (5.6 mmol/L) but no more than one ≥126 mg/dL (7.0 mmol/L).

Normoglyceamic

Patients not fulfilling either of the above criteria, will be classified as normoglyceamic at baseline.

Disease characteristics at baseline

Specific disease history includes:

- Information on index qualifying ACS event: number (%) of patients with elevated cardiac biomarkers (troponin I or T, or CKMB); with resting ECG changes consistent with ischemia or infarction (ST depression, ST elevation, T wave inversion, pathological Q waves, new tall R wave) and additional evidence of obstructive coronary disease (evidence of myocardial or infarction by perfusion imaging, regional wall motion abnormality, epicardial coronary artery stenosis ≥70%, need for revascularization [percutaneous coronary intervention (PCI) or coronary artery bypass graft surgery (CABG)] related to index ACS event);
- The index ACS subtype:
  - STEMI: elevated cardiac biomarkers (Troponin I or T, and/or CKMB) and new or presumed new ST elevation
  - NSTEMI: elevated cardiac biomarkers (Troponin I or T, and/or CKMB) and No new or presumed new ST elevation
  - UA: No Troponin I or T elevated, and No CKMB elevated, and "Resting ECG changed consistent with ischemia or infarction AND additional evidence of obstructive coronary disease" (ie, any combination of responses to questions "B" on the electronic case report form [e-CRF])
- Time from index ACS event to randomization (in weeks and in months), quantitatively and in category <2, ≥2 to <4, ≥4 to <6, ≥ 6 months
- Revascularization procedure associated with the index ACS event (PCI or CABG)
- Time from the revascularization procedure to randomization (in weeks and in months)
- New cardiovascular events occurring during the run-in period, selected using a list of preferred terms (PTs) from CMQ or standardized MedDRA query (SMQ).

Other baseline characteristics

Other baseline characteristics include weight in kilograms (quantitative variable), and body mass index (BMI) in kg/m² (quantitative and qualitative variable: <30, ≥30).

Lipid parameters, HbA_{1c} (quantitative and qualitative variable: <5.7 %, ≥5.7 to <6.5%, ≥6.5%) and hs-CRP (quantitative and qualitative variable: <2, ≥2 mg/L) at baseline will be also summarized by treatment group. Lipid parameters are total cholesterol (TC), LDL-C, non HDL-C, HDL-C, fasting triglycerides (TG), Apo A-1, Apo B, and lipoprotein (a) (Lp (a)).

For lipid parameters, both quantitative and qualitative variables will be considered, with the following categories:

- LDL-C: <70, ≥70 to <80, ≥80 to <100, ≥100 to <130, ≥130 to <160, ≥160 to <190, ≥190 mg/dL (ie, <1.81, ≥1.81 to <2.07, ≥2.07 to <2.59, ≥2.59 to <3.37, ≥3.37 to <4.14, ≥4.14 to <4.91, ≥4.91 mmol/L)
- HDL-C: <40, ≥40 mg/dL (ie, <1.04, ≥1.04 mmol/L),
- Non-HDL-C: <100, ≥100 to <110, ≥110 to <130, ≥130 to <160, ≥160 to <190, ≥190 to <220, ≥220 mg/dL (ie, <2.59, ≥2.59 to 2.84, ≥2.84 to <3.37, ≥3.37 to <4.14, ≥4.14 to <4.91, ≥4.91 to 5.69, ≥5.69 mmol/L)
- Fasting TG: <150, ≥150 to <200, ≥200 mg/dL (ie, <1.7, ≥1.7 to <2.3, ≥2.3 mmol/L), category ≥150 mg/dL (ie, ≥1.7 mmol/L [mixed dyslipidemia]) will be also displayed,
- Lp (a): <30, ≥30 to <50, ≥50 mg/dL (ie, <0.3, ≥0.3 to <0.5, ≥0.5g/L), category ≥30 mg/dL (ie, ≥0.3 g/L) will be also displayed
- Apo B: <75, ≥75 to <90, ≥90 mg/dL (ie, <0.75, ≥0.75 to <0.9, ≥0.9 g/L)

In addition the number (%) of patients not adequately controlled at baseline as per protocol definition will be described:

- LDL-C ≥70 mg/dL (1.81 mmol/L) or Apo B ≥80 mg/dL (0.8 g/L) or non-HDL-C ≥100 mg/dL (2.59 mmol/L)
- LDL-C ≥70 mg/dL (1.81 mmol/L)
- Apo B ≥80 mg/dL (0.8 g/L)
- Non-HDL-C ≥100 mg/dL (2.59 mmol/L)

Any technical details related to computation, dates, and imputation for missing dates are described in Section 3.5.

3.1.2 Prior or concomitant medications

All LMTs taken within 1 month before screening visit V1 and until the end of the study, are to be reported in 1 of the following specific case report form pages:

- Previous and concomitant statin drugs;
- Previous and concomitant medications lipid lowering drugs (other than statins);

Patients on chronic use of statin (ie, on any statin for at least 3 months prior to the index ACS event) and the reasons for any modification in the statin regimen post-randomization are to be reported on the following specific page:

- Additional statin information

Other concomitant medications taken since informed consent, including cardiovascular medications are to be reported on the following specific page:

- Concomitant medications (all other than statin and other than lipid lowering drugs).

All medications will be coded using the World Health Organization-Drug Dictionary (WHO-DD) using the version currently in effect at Sanofi at the time of database lock.

- Prior medications are those the patient used within 1 month before screening visit V1 and prior to first investigational medicinal product (IMP) injection. Prior medications can be discontinued before first administration or can be ongoing during treatment phase;
- Concomitant medications are any treatments received by the patient concomitantly to the IMP, from first IMP injection to the last double-blind injection +70 days. A given medication can be classified both as a prior medication and as a concomitant medication. Concomitant medications do not include medications started during the post-treatment period (as defined in the observation period in Section 3.1.4);
- Post-treatment medications are those the patient took in the period starting the day after the concomitant medication period up to the end of the study.

For patients randomized but not treated, medications will be categorized as prior medications or post-treatment medications according to the intake dates in relation to the date of randomization.

The following medications of specific interest will be also selected using specific coding's list:

- Aspirin or oral ADP receptor antagonists
- Injectable anticoagulants (UFH or LMWH or Bivalirudin or Selective Factor Xa inhibitor)
- Thrombolytic
- Specific oral anticoagulant
- Anti-diabetic drugs (insulin, oral anti-diabetic, other non-oral anti-diabetic)
- ACE-inhibitor or angiotensin receptor blocker
- Beta blocker

- Calcium channel blocker
- Diuretics
- Nitrates
- NSAIDs (excluding aspirin)

Any technical details related to computation, dates, imputation for missing dates are described in Section 3.5.

3.1.3 Efficacy endpoints

3.1.3.1 Primary efficacy endpoint(s)

The primary efficacy endpoint is the time from randomization to first occurrence of 1 of the following clinical events, as determined by the CEC:

- CHD death (including "undetermined causes of death" as per the CEC);
- Any non-fatal MI;
- Fatal and non-fatal ischemic stroke (including "stroke not otherwise specified" as per the CEC);
- Unstable angina requiring hospitalization.

The rules to determine the components and the date of the event that will be considered in the analyses of the primary efficacy endpoint are detailed in Appendix C.

If none of these events is observed at the time of the analysis cut-off date (final or interim, depending on the timing of the analysis, see Section 3.4.4.1 and Section 4 for details), the patient will be right-censored at the date of last contact when information on efficacy endpoints (presence or absence) has been retrieved, or at the date of death, or at the cut-off date/CSED, whichever comes first.

The last information on efficacy endpoint (presence or absence)" will be the latest date among:

- The date of last visit performed with "endpoints events" e-CRF page completed.
- The "Date of last information on efficacy endpoint (presence or absence)" reported in the visit e-CRF pages during the course of the study.
- The "Date of last information on efficacy endpoint (presence or absence)" reported at the end of the study for all patients (this information can also be completed during the course of the study for "lost-to-follow-up" patients, patients who discontinued the follow-up, and patients who died).

In case of no information on the presence or absence of efficacy endpoint, nor on death at time of database extraction for the interim analyses, the censoring date will be the randomization date (eg, for patients randomized but having not reached the Month 1 visit yet).

Handling of missing or incomplete dates

In the exceptional circumstances when the exact date of occurrence of the outcome event has not been established (day and/or month missing), the date will be imputed as follows:

Table 4 - Imputation of incomplete date of events composing the primary efficacy outcome

Type of event	Imputed date
	**Only the day is missing**
Death	The latter of the last contact date + 1 day and the 1st day of the month.
Other primary endpoint component (CHD event or ischemic stroke)	The latter of the randomization date and the 1st day of the month.
	**Day and month are missing**
Death	The latter of the last contact date + 1 day and January 1st of the year.
Other primary endpoint component (CHD event or ischemic stroke)	The latter of the randomization date and January 1st of the year.

CHD = coronary heart disease

3.1.3.2 Secondary efficacy endpoint(s)

Clinical events assessed in the analyses are those determined by the CEC. Events suspected by the investigator but not confirmed by the CEC will not be part of the outcomes. CHD death outcome will include deaths for causes that could not be determined by the CEC (adjudicated as "Undetermined cause of death"). Similarly, fatal and non-fatal ischemic stroke outcomes will include fatal and non-fatal strokes for causes that couldn't be determined by the CEC (adjudicated as "Stroke not otherwise specified").

Rules for censoring date and for imputation of incomplete dates of events will be the same as for the primary efficacy endpoint, for all secondary efficacy endpoints with the exception of the analyses of the time to CHD death, the time to CV death and of the time to all-cause mortality for which the censoring date will be defined as the earliest date among the "date of last known alive or Date of death" and the cut-off date/CSED.

The "date of last known alive or Date of death" will be the latest date among:

- The date of last visit performed;
- The "Date of last known alive or Date of death" reported in the visit e-CRF pages during the course of the study;
- The "Date of last known alive or Date of death" reported at the end of the study for all patients (this information can also be completed during the course of the study for "lost-to-follow-up" patients, patients who discontinued the follow-up and patients who died);

(electronic 3.0)

- The latest date among these dates, the last IMP injection date, the date of adverse events, the date of CV events, the date of death and the date of laboratory samples will be considered.

In case at time of database extraction for the interim analyses, only the randomization date is available, then the censoring date will be the randomization date.

3.1.3.2.1 Main secondary endpoint(s)

- Time from randomization to first occurrence of any CHD event (major CHD event, unstable angina requiring hospitalization, ischemia-driven coronary revascularization procedure);
- Time from randomization to first occurrence of any major CHD event (CHD death, nonfatal MI);
- Time from randomization to first occurrence of any CV event (any non-fatal CHD event, any CV death, and non-fatal ischemic stroke);
- Time from randomization to first occurrence of all-cause mortality, non-fatal MI, non-fatal ischemic stroke;
- Time from randomization to CHD death;
- Time from randomization to CV death;
- Time from randomization to death (all-cause mortality).

3.1.3.2.2 Other secondary efficacy endpoint(s)

- Component of the primary endpoint considered individually:
  - Time from randomization to first occurrence of any non-fatal MI;
  - Time from randomization to first occurrence of fatal or any non-fatal ischemic stroke;
  - Time from randomization to first occurrence of any unstable angina requiring hospitalization.
- Time from randomization to first occurrence of any ischemia-driven coronary revascularization procedure;
- Time from randomization to first occurrence of any congestive heart failure requiring hospitalization.

3.1.4 Safety endpoints

Following injections are considered as double-blind IMP injections:

- For patients randomized in the placebo group: any injection from double-blind kits;

- For patients randomized in the alirocumab group: any injection from double-blind kits excepted placebo injections given to maintain the blind in case of 2 consecutive LDL-C values <15 mg/dL.

Of note potential additional training injections after randomization using training injection kits will not be taken into account.

The period of safety observation starts from the time when the patient gives informed consent and is divided into three periods:

- PRE-TREATMENT period: defined from the signed informed consent up to the first dose of double-blind IMP injection;
- Treatment-emergent adverse event (TEAE) period: defined as the time from the first dose of double-blind IMP injection to the last dose of double-blind IMP injection +70 days (10 weeks), (as residual effect of treatment is expected until 10 weeks after the stop of double-blind IMP).

  The TEAE period will include:

  - The TREATMENT period defined as the time from the first dose of double-blind IMP up to the day of last dose of double-blind IMP injection +21 days, as serum concentration of alirocumab >10 µg/mL is expected for approximately 21 days following administration of 150 mg, and because throughout the previous studies it was observed that when alirocumab concentrations declined below this concentration, decrease in effect on LDL-C is observed.
  - The RESIDUAL TREATMENT defined as the time from the day of last dose of double-blind IMP injection +22 days up to the day of last dose of double-blind IMP injection +70 days (10 weeks).
- POST-TREATMENT period: defined as the time starting the day after the end of the TEAE period.

3.1.4.1 Adverse events variables

Occurrence of adverse events (including serious adverse events [SAEs], and adverse events of special interest [AESIs]) are recorded from the time of signed informed consent until the end of study.

All adverse events will be coded to a “Lowest Level Term (LLT)”, “Preferred Term (PT)”, “High Level Term (HLT)”, “High Level Group Term (HLGT)”, and associated primary “System Organ Class (SOC)” using the version of MedDRA currently in effect at Sanofi at the time of the database lock.

Adverse event observation periods

- Pre-treatment adverse events are adverse events that developed or worsened or became serious during the pre-treatment period;

- Treatment-emergent adverse events are adverse events that developed or worsened or became serious during the treatment-emergent adverse event period;
- Post-treatment adverse events are adverse events that developed or worsened or became serious during the post-treatment period.

Adverse events of special interest

Adverse events of special interest (AESIs) are adverse events (serious or non-serious) that need to be monitored, documented, and managed in a pre-specified manner described in the protocol. In this study, AESI are the following (their complete descriptions are provided in the protocol):

- General allergic events, selected using SMQ “hypersensitivity” (broad and narrow) excluding the following preferred terms linked to local injection site reactions (“infusion site dermatitis”, “infusion site hypersensitivity”, “infusion site rash”, “infusion site urticaria”, “injection site dermatitis”, “injection site hypersensitivity”, “injection site rash”, “injection site urticaria”, and “injection site vasculitis”)
- Local injection site reactions, selected using e-CRF specific tick box on the adverse event page
- ALT ≥3 ULN (if baseline ALT <ULN) or ALT ≥2 times the baseline value (if baseline ALT ≥ULN), selected using laboratory data
- Hemolytic anemia, selected using e-CRF specific tick box on the adverse event page and confirmed final diagnosis provided in the adverse event complementary form
- Neurologic events selected using a CMQ, based on SMQs “demyelination” (broad and narrow), “peripheral neuropathy” (broad and narrow), and “Guillain-Barre syndrome” (broad and narrow) excluding the following preferred terms “acute respiratory distress syndrome”, “asthenia”, “respiratory arrest” and “respiratory failure” and including selected PTs from SMQ "optic nerve disorders" (see Appendix B Table 13 for the list of terms)
- Neurocognitive events:
  - Selected using a CMQ, based on the following 5 HLGTs: “deliria (including confusion)”, “cognitive and attention disorders and disturbances”, “dementia and amnestic conditions”, “disturbances in thinking and perception”, and “mental impairment disorders”
  - A second grouping of terms for neurocognitive events was defined based on Regulatory Agency request (see Appendix B Table 14 for the list of terms)
- Overdose of IMP (symptomatic or asymptomatic), selected using appropriate MedDRA codes and the tick box “Overdose with IMP” in the adverse event complementary e-CRF form
- Pregnancy (including partner of a randomized male subject) selected using appropriate MedDRA codes

In addition the additional grouping of events will be provided:

- Hepatic disorder events using SMQ “Hepatic disorder”
- Diabetes mellitus or diabetic complications using HLGT “diabetes complications” (including PTs pertaining to the secondary SOC included in the HLGT), HLT “diabetes mellitus”, and HLT “carbohydrate tolerance analyses (incl diabetes)” excluding PTs “blood glucose decreased” and “Glycosylated haemoglobin decreased” and including the PTs “hyperglycaemia”, “Hyperglycaemic unconsciousness” and “Hyperglycaemic seizure” from the HLT "Hyperglyceamic conditions NEC"
- New onset of diabetes (in the subgroup of patients not having diabetes at baseline) (see definition in Section 3.4.5.3)
- Cataract using HLT “Cataract conditions”

3.1.4.2 Deaths

The deaths observation period are per the observation periods defined below.

- Deaths from first IMP injection until CSED:
  - Treatment-emergent deaths: deaths occurring during the TEAE period
  - Post-treatment emergent deaths
- Deaths post-CSED

3.1.4.3 Laboratory safety variables

Clinical laboratory data consists of blood analysis, including hematology, clinical chemistry, and urinalysis. Clinical laboratory values after conversion will be analyzed into standard international units. International units will be used in all listings and tables. Clinical laboratory values converted into conventional (US) units will be also available in the database. Analyses can be provided upon request.

Blood samples for clinical laboratories will be taken as described in the study flow chart in the protocol.

The laboratory parameters will be classified as follows:

- Hematology
  - **Red blood cells and platelets**: hemoglobin, hematocrit, red blood cell count, platelet count;
  - **White blood cells:** white blood cell count, neutrophils, lymphocytes, monocytes, basophils, eosinophils.

- Clinical chemistry
  - **Metabolism:** fasting plasma glucose, total protein, albumin, creatine phosphokinase (CPK);
  - **Electrolytes**: sodium, potassium, chloride, calcium, phosphorus, bicarbonate;
  - **Renal function**: creatinine, eGFR, blood urea nitrogen, uric acid;
  - **Liver function**: alanine aminotransferase (ALT), aspartate aminotransferase (AST), alkaline phosphatase (ALP), gamma glutamyl transferase (γGT), lactate hydrogenase (LDH), total bilirubin, and in case of total bilirubin values above the normal range, must include direct and indirect bilirubin (used for describing individual cases only);
  - **Hepatitis screen:** anti-hepatitis-C antibody.

Technical formulas are described in Section 3.5.1.

3.1.4.4 Vital signs variables

Vital signs include: weight, heart rate, systolic and diastolic blood pressure in sitting position.

3.1.4.5 Electrocardiogram variables

Electrocardiograms were recorded automatically by the device at the Investigator site.

Electrocardiogram assessments will be described as normal or abnormal.

3.1.5 Other endpoints

Other assessment endpoints defined below are exploratory.

3.1.5.1 Lipid parameters

The lipid parameters include values (in conventional [US] and international units), percent change from baseline, and/or when appropriate absolute change from baseline (in conventional and international units) over time for the following parameters: LDL-C, TC, HDL-C, fasting TGs, non-HDL-C, Apo A-1, Apo B, ratio Apo B/Apo A-1, Lp (a), ratio TC/HDL.

All these parameters are measured or calculated by a central laboratory, for both scheduled and unscheduled time points. For LDL-C analysis, both calculated and measured LDL-C values will be taken into account. In case both calculated and measured LDL-C are provided for the same sampling, the measured LDL-C will be considered. Calculated LDL-C is obtained using the Friedewald formula. Non-HDL-C is calculated by subtracting HDL-C from the TC.

Unless otherwise specified, all central measurements (scheduled or unscheduled, fasting or not fasting) may be used to provide a value for each timepoint, even if assessed after treatment discontinuation (intent-to-treat [ITT] approach). The analysis windows used to allocate a measurement to a time point are defined in Section 3.5.4. For TG, only fasting measurements will be used. Measurements with missing fasting status will be excluded from the analyses.

For all time points post-baseline, the value used for the analyses at a given time point (eg, at Month 24) is the value obtained within the corresponding analysis window. The baseline value is the last available measurement obtained up to the date and time of the first double-blind IMP injection. For patients randomized and not treated, the baseline value is defined as the last available value obtained up to the date and time of randomization.

Percent changes from baseline to a timepoint are defined as: 100 x (parameter value at the time point – parameter value at baseline) / parameter value at baseline.

Data handling conventions for other endpoints are described in Section 3.5.

3.1.5.2 hs-CRP

The percent change in hs-CRP from baseline over time is defined using same definitions and rules as for LDL-C, when applicable (see section above). hs-CRP values greater or equal to 10 mg/L will be excluded from analyses in a second approach, since these are suggestive of concurrent infections, MI, or other events provoking an acute phase response (2).

PCSA criteria for hs-CRP are defined in Appendix A.

3.1.5.3 HbA_{1C}

The absolute change in HbA_{1c} (%) from baseline over time: same definitions and rules as for LDL-C (see Section 3.1.5.1).

3.1.5.4 Patients with LDL-C <25 mg/dL (0.65 mmol/L)

The assessment will include:

- The proportion of patients with two consecutive results, spaced out by at least 21 days, of LDL-C <25 mg/dL (<0.65 mmol/L) (respectively LDL-C <15 mg/dL, ie, <0.39 mmol/L) during the double-blind treatment period
- The time to the first LDL-C <25 mg/dL (respectively LDL-C <15 mg/dL) for these patients.

In case both calculated and measured LDL-C are provided for the same sampling, the measured LDL-C will be considered.

3.1.5.5 Anti-alirocumab antibodies assessed throughout the study.

Anti-alirocumab antibodies (ADAs) are assessed at Visit 3 (Month 0), Visit 5 (Month 2), Visit 6 (Month 4), and Visit 10 (Month 12) for the first year, then every year and at the final on-treatment visit (CSED visit for completers, or early end of treatment visit for patients who discontinued the treatment).

ADA measurements will be assigned to analysis windows as defined in Section 3.5.4.

The following variables will be described:

- ADA response (Positive or Negative). For ADA positive:
  - Titer levels
  - Neutralizing status (Positive or Negative)
- Pre-existing positive ADA defined as patients with positive ADA response at baseline with less than 4-fold increase in titer in the post-baseline period
- Treatment-emergent positive ADA response defined as 1) Patients with no ADA positive response at baseline but with any positive response in the post-baseline period (up to follow-up visit) or 2) Patients with a positive ADA response at baseline and at least a 4-fold increase in titer in the post-baseline period (up to follow-up visit). For treatment-emergent positive ADA, the following categories for ADA duration will be applied:
  - A persistent positive response is a treatment-emergent ADA positive response detected in at least 2 consecutive post-baseline samples separated by at least a 16-week period
  - An indeterminate duration positive response is defined as ADA present only at the last sampling time point
  - A transient positive response is defined as any treatment-emergent positive ADA response that is neither considered persistent nor indeterminate

In addition, potential ADA samples that are to be collected after CSED visit for patients with titer ≥240 at CSED visit will be listed.

3.1.5.6 Quality-of-life parameters

EQ-5D™ is a standardized and generic instrument for measuring the health status and health related quality of life for clinical and economic assessment (3). EQ-5D instrument includes 5 items corresponding to the following dimensions: mobility, self-care, usual activities, pain/discomfort, anxiety/depression (Appendix D). Each item can take 1 of 3 responses: (1.) "no problem", (2.) "some problems", and (3.) "severe problems". Overall health status is defined as a 5-digit number and will be converted into a standard utility score ranging between -0.594 (representing severe problems) and 1 (representing no problem): the single index utility score, using a regression model (4) (Appendix E). If response to one or more dimension is missing, the utility score will be missing.

Quality of life parameters include response to each EQ-5D items and change in utility score over time from baseline.

3.1.5.7 Cardiovascular events of interest (other than efficacy endpoints)

Cardiovascular events of interest (other than efficacy endpoints) include clinically significant complications or procedures (not planned at the time of randomization), related to peripheral arterial disease and venous thromboembolic events as listed below:

- Venous thromboembolic events of interest:
  - Deep vein thrombosis (DVT)
  - Pulmonary embolism (PE)
- Lower extremity peripheral arterial disease events of interest:
  - Peripheral lower limb revascularization (endovascular revascularization, surgical revascularization)
  - Critical limb ischemia (including ischemic imputation of lower limb for the event)

The following variables will be analyzed:

- Time from randomization to first occurrence of any other CV events of interest (venous thromboembolic events or lower extremity peripheral arterial disease events)
- Time from randomization to first occurrence of any venous thromboembolic events (DVT or PE)
- Time from randomization to first occurrence of any lower extremity peripheral arterial disease events (peripheral lower limb revascularization or critical limb ischemia)

3.1.6 Pharmacokinetic variables

Not applicable.

3.1.7 Pharmacogenomics endpoints

Pharmacogenetics endpoints and analyses will be detailed in a separate SAP.

3.2 DISPOSITION OF PATIENTS

This section describes patient disposition for both patient study status and the patient analysis populations.

Screened patients are defined as any patient who met the ACS inclusion criteria and signed the informed consent (ie, patients entering the run-in phase).

Randomized patients consist of all screened patients, with a double-blind treatment kit number allocated and recorded in the interactive voice response system (IVRS)/ interactive web response system (IWRS) database, regardless of whether the treatment kit was used or not. Patients treated without being randomized or treated with a double-blind treatment kit before the randomization will not be considered as randomized and will not be included in any analysis population.

For patient study status, the total number of patients in each of the following categories will be presented in the clinical study report using a flowchart diagram or summary table:

- Screened patients;
- Screened failure patients and reasons for screen failure;
- Non-randomized but treated patients, if any;
- Randomized patients;
- Randomized but not treated patients and reason for not being treated;
- Randomized and treated patients;
- Patients who completed the double-blind study treatment period as per protocol (as per e-CRF end-of-treatment form);
- Patients who did not complete the double-blind study treatment period as per protocol (as per e-CRF end-of-treatment form);
- Patients who discontinued the double-blind study treatment by main reason for permanent treatment discontinuation (as per e-CRF end-of-treatment form);
- Status at last study contact.

For all categories of patients (except for the screened and non-randomized categories) percentages will be calculated using the number of randomized patients as the denominator.

Reasons for treatment discontinuation will be supplied in tables giving numbers and percentages by treatment group. For blinded discontinuation due to low LDL-C level (patients who switched to placebo in blinded manner), the reason reported by the investigator will be reclassified as "2 consecutive LDL-C <15mg/dL (<0.39 mmol/L)".

Number (%) of patients who discontinued the follow-up for CV events will be summarized over time. The main reason for study discontinuation will be summarized overall and according to whether or not the patients had a primary efficacy endpoint confirmed by CEC prior study discontinuation.

A patient will be considered as having discontinued the follow-up for CV events if the date of the last information on efficacy endpoints (presence or absence) is before the common study end date.

Kaplan-Meier plots/estimates of the cumulative incidence of premature IMP treatment discontinuation due to any reason, or due to adverse event will be provided on randomized population. Not treated patient will be considered with event at Day 1 (day of randomization). All completers will be considered as right-censored observations. Time to premature IMP treatment discontinuation and censoring time will be defined as: Date of last IMP injection – Date of randomization + 14 days.

All major deviations potentially impacting efficacy analyses, randomization, and drug-dispensing irregularities, and other major deviations will be summarized in tables giving numbers and percentages of deviations by treatment group. These deviations are listed in the data review and surveillance plan.

Additionally, the following populations will be summarized by treatment group.

- Randomized population;
- Efficacy population: intent-to-treat (ITT) population;
- Safety population;
- Anti-alirocumab antibody population.

Definition of the study populations are provided in Section 3.3.

3.2.1 Randomization and drug dispensing irregularities

Randomization and drug-dispensing irregularities occur whenever:

1. A randomization is not in accordance with the protocol-defined randomization method, such as a) a patient is randomized based on an incorrect stratum, or b) a patient is randomized twice.

OR

2. A patient is dispensed an IMP kit not allocated by the protocol-defined randomization, such as a) a patient at any time in the study is dispensed a different treatment kit than as randomized (which may or may not contain the correct-as-randomized IMP), or b) a non-randomized patient is treated with IMP reserved for randomized patients.

Randomization and drug-dispensing irregularities will be monitored throughout the study and reviewed on an ongoing basis.

All randomization and drug-dispensing irregularities will be documented in the clinical study report. These irregularities will be summarized by treatment group on the randomized population. Non-randomized, treated patients will be described separately.

Randomization and drug-dispensing irregularities to be prospectively identified include but are not limited to:

Table 5 - Randomization and drug allocation irregularities

Kit dispensation without IVRS/IWRS transaction
Erroneous kit dispensation
Patient randomized twice
Stratification error
A kit allocated at Day 1 or any unscheduled replacement before the up-titration visit (it may be the Month 2 or the Month 4 visit) is administered to the patient after the up-titration visit[a]
A kit allocated at any visit or unscheduled replacement from the up-titration visit, is administered to the patient after the next scheduled reallocation visit[a]

a Only if dose received is different from the one expected as per IVRS/IWRS allocation

3.3 ANALYSIS POPULATIONS

Patients treated without or before being randomized will not be considered randomized and will not be included in any analysis populations. The safety experience and CV events of patients treated and not randomized will be reported separately.

Randomized population: includes all randomized patients as defined in Section 3.2.

For any patient randomized more than once, only the data associated with the first randomization will be used in any analysis population. The safety experience associated with any later randomization will be assessed separately.

3.3.1 Efficacy populations

3.3.1.1 Intent–to-treat population

The primary efficacy analysis population will be the intent-to-treat (ITT) population, consisting of all randomized patients. Patients in the ITT population will be analyzed according to the treatment group allocated by randomization.

3.3.2 Safety population

The Safety population considered for safety analyses will be the randomized patients who actually received at least 1 dose or part of a dose of the double-blind IMP injection. Patients will be analyzed according to the treatment actually received (ie, as-treated treatment group, placebo or alirocumab).

In addition:

- Randomized patients for whom it is unclear whether they took the study medication will be included in the safety population in the treatment group as randomized;
- For patients receiving double-blind IMP injection from more than one treatment group during the trial (cases reported as protocol deviation), the treatment group used for as-treated analysis will be the one to which the patient was treated with the highest number of injections; in case of the same number of injections of each treatment is received, the as-treated treatment group will be the as-randomized group.

3.3.3 Anti-alirocumab antibody population

The ADA analysis will be performed on all randomized and treated patients (safety population) with an available ADA sample at Day 1 (baseline) and at least 1 available ADA sample post first double-blind IMP injection.

3.4 STATISTICAL METHODS

3.4.1 Demographics and baseline characteristics

Parameters described in Section 3.1.1 will be summarized by treatment group and overall using descriptive statistics.

Continuous data will be summarized using the number of available data, mean, standard deviation (SD), median, minimum, and maximum for each treatment group. First quartile (Q1) and third quartile (Q3) will be also provided for baseline lipid parameters, HbA_{1c}, and hs-CRP. Categorical and ordinal data will be summarized using the number and percentage of patients in each treatment group.

Unless otherwise specified, parameters will be summarized on the randomized population analyzed in the treatment group to which they were randomized. Similar analyses will be done on the safety population and will be included in the appendices if the size of the safety population is different (>10%) from the size of the randomized population for any treatment group. In the randomized population, parameters will also be summarized within each region.

All reported patient's medical and surgical history will be presented by primary SOC and HLT. The tables will be sorted by SOC internationally agreed order and decreasing frequency of HLT based on the overall incidence across treatment groups. In addition all medical history of specific interest will be presented by treatment group.

The diagnosis of diabetes mellitus at baseline (see also Section 3.1.1 for definition), as well as the source of diagnosis will be summarized in the randomized and safety populations by treatment group and overall, using the following mutually exclusive categories:

- From medical history or pre-treatment adverse events
- From anti-diabetic medications regardless of laboratory data (if no medical history of diabetes)
- From laboratory data only (if no medical history of diabetes and no anti-diabetic medications)

No specific description of the safety parameters will be provided at baseline. If relevant, the baseline values will be described along with each safety analysis.

3.4.2 Prior, concomitant or post-treatment medications

The prior, concomitant, and post-treatment medications will be presented for the randomized population.

Medications will be summarized by treatment group according to the WHO-DD dictionary, considering the first digit of the anatomical therapeutic chemical (ATC) class (anatomical category) and the first 3 digits of the ATC class (therapeutic category). All ATC codes corresponding to a medication will be summarized, and patients will be counted once in each ATC category (anatomical or therapeutic) linked to the medication. Therefore patients may be counted in several categories for the same medication.

The table for prior medications will be sorted by decreasing frequency of ATC followed by all other therapeutic classes based on the overall incidence across treatment groups. In case of equal frequency regarding ATCs (anatomical or therapeutic categories), alphabetical order will be used.

The tables for concomitant and post-treatment medications will be sorted by decreasing frequency of ATC followed by all other therapeutic classes based on the incidence in the alirocumab group. In case of equal frequency regarding ATCs (anatomical or therapeutic categories), alphabetical order will be used. In addition all medications of specific interest will be presented by treatment group.

The number and percentage of patients that took any statin for at least 3 months prior to the index ACS event (as reported on the e-CRF page "Additional statin information") will be displayed by treatment group.

The background lipid modifying therapy regimen at randomization will be summarized using the following categories:

- High dose atorvastatin/rosuvastatin (defined as daily atorvastatin 40 to 80 mg, or rosuvastatin 20 to 40 mg)
- Low/moderate dose atorvastatin/rosuvastatin (defined as daily atorvastatin <40 mg, or rosuvastatin <20 mg)
- Statin other than atorvastatin or rosuvastatin, at any dose
- Only LMT other than statin
- No LMT

The reason for not being on high dose at randomization will be supplied in tables giving numbers and percentages by treatment group.

Details (ie, statin names, doses) for patients who had received at least 2 statins the day of randomization (if any) will be listed.

For atorvastatin and rosuvastatin, the dose (in mg) will be also displayed by treatment group.

- Atorvastatin daily dose in mg (10, 20, 40, 80, Other);
- Rosuvastatin daily dose in mg (5, 10, 20, 40, Other);

The LMT other than statins will be summarized by pre-specified categories, chemical class or therapeutic class, and standardized medication name.

In addition the number (%) of patients in the following background LMT categories at randomization will be displayed:

- Ezetimibe and high dose atorvastatin/rosuvastatin
- Ezetimibe and low/moderate dose atorvastatin/rosuvastatin
- Ezetimibe and statin other than atorvastatin or rosuvastatin
- Ezetimibe and other LMT other than statin
- Only ezetimibe

LMT (statins and other LMTs) used after randomization during the study will be summarized over time graphically by treatment group and LMTs intensity at randomization using the following mutually exclusive categories:

- High dose atorvastatin/rosuvastatin
- Low/moderate dose atorvastatin/rosuvastastin
- Statin other than atorvastatin or rosuvastatin
- Only LMT other than statin
- No LMT

The reason for first modification in statin regimen after randomization will be also described.

The use of ezetimibe after randomization during the study will be also summarized over time graphically by treatment group.

The number (%) of patients initiating ezetimibe after randomization will be also displayed according to background LMT status at randomization.

3.4.3 Extent of investigational medicinal product exposure and compliance

The extent of IMP exposure and compliance will be assessed and summarized by actual treatment received within the safety population.

Double-blind IMP kits contain the following:

- Placebo for the ones administered to patients randomized in the placebo group
- 75 or 150 mg of alirocumab

Placebo injections administered to patients randomized in the alirocumab following 2 consecutive LDL-C <15 mg/dL will not be considered as double-blind IMP injections in the statistical analyses.

3.4.3.1 Extent of investigational medicinal product exposure

The total exposure will be assessed by:

- Duration of IMP exposure in months defined as: (last dose of double-blind IMP injection date + 14 – first dose of double-blind IMP injection date) / 30.4375, regardless of intermittent discontinuations (see Section 3.5.3 for calculation in case of missing or incomplete data). Non-integer values will be rounded to one decimal place;
- The total number of double-blind IMP injections by patient;

In addition the duration of the observation period in months will be analyzed. The duration of observation period is defined as: (last contact date – randomization date+1)/30.4375. Non-integer values will be rounded to one decimal place.

These parameters will be summarized descriptively as a quantitative variable (number, mean, SD, median, minimum, and maximum). In addition, the durations of treatment exposure and observation period will be presented according to the following categories: <2, ≥2 to <6, ≥6 to <12, ≥12 to <24, ≥24 to <36, ≥36 to <48, ≥48 to <60, ≥60 months. The total number of double-blind IMP injections by patient will be presented similarly using the following categories: <4, ≥4 to <13, ≥13 to <26, ≥26 to <39, ≥39 to <52, ≥52 to <65, ≥65 to <78, ≥78 to <104, ≥104 to <120, ≥120.

Additionally, the cumulative exposure will be provided in patient years.

Titration

The following summaries will be provided in the alirocumab group:

- The number (%) of patients with an up-titration to 150 mg, overall and according to the time of up-titration (ie, Month 2 and Month 4)
- The number (%) of patients with an up-titration followed by a down-titration to 75 mg
- The number (%) of patients with a switch to placebo
- The number (%) of patients on 75 mg, 150 mg, placebo over time (intermittent discontinuations won't be taken into account)

Cumulative exposure in patient-year by dose level (75 mg, 150 mg, placebo) will be provided, not taking into account intermittent discontinuations.

3.4.3.2 Compliance

Compliance will be assessed using the injection frequency that will be defined for each patient as the average number of days between 2 injections, that is: (last dose date – first dose date) / (number of injections – 1).

Cases of overdose (ie, 2 or more injections from the double-blind treatment kit are administered in <7 calendar days) will be summarized by treatment group. More generally, dosing irregularities are defined in Section 3.2.1.

3.4.4 Analyses of efficacy endpoints

All efficacy analyses will be performed based on ITT approach that will include events occurring from randomization to the analysis cut-off date for interim analysis or CSED for the final analysis, even after the patient has discontinued the study treatment. Any CV endpoint events occurring after the cut-off date/CSED will not be included in the analyses, regardless of the adjudication status. These events, if any, will be reported in a listing separately.

3.4.4.1 Analysis of primary efficacy endpoint(s)

The analysis of the primary efficacy endpoint will be the comparison between the two treatments using a log-rank test stratified by region (North America, South America, Western Europe, Eastern Europe, Asian, Other). The randomization is stratified by country but since the number of events per individual country is expected to be low (about 50 countries), the analysis will be stratified according to a grouping of countries into regions.

This primary comparison will be the 1-sided test (at 0.0249 type 1 error for the final analysis) of the following hypotheses at the final analysis:

$$H_0: HR \geq 1 \text{ versus } H_1: HR < 1$$

The estimates of the HR and corresponding confidence interval (CI) at (1-2α)% level (α being the 1-sided significance level: α=0.0249 at final analysis, α=0.0001 at second interim analysis) will be provided using a Cox Proportional Hazard model stratified by region. The underlying assumption of proportional hazards for Cox model will be checked by visual inspection of Kaplan-Meier plots. If proportionality is not observed, sensitivity analyses will be performed. In particular, the results will be presented by yearly intervals: the number of events per 100 patient-years for each yearly interval will be provided for each treatment group as well as the ratio of the two event rates. In addition, between-treatment cumulative rate ratios based on the Kaplan-Meier estimates and the corresponding 95% CIs) will be provided at yearly interval.

The cumulative incidence rate over time (at 6 months and by year) together with appropriate interval will be estimated by treatment group using Kaplan-Meier estimates.

Reasons for censoring (including patient who died before the cut-off date/CSED for other reason than CHD, lost to follow-up) will be summarized. For patients censored before the cut-off date/CSED, time from last contact when information on efficacy endpoints has been retrieved to cut-off date will be summarized.

Interim analyses

Two interim analyses will be performed. See Section 4 for description of these analyses. The cut-off dates of final (ie, CSED) and interim analyses are expected to be:

- First interim analysis date (futility): when 807 patients have experienced at least 1 primary efficacy event (50% fraction information);
- Second interim analysis date (futility and overwhelming efficacy): when 1210 patients have experienced at least 1 primary efficacy event (75% fraction information);
- Final analysis date: when 1613 patients have experienced at least 1 primary efficacy event or 24 months after the last date of randomization ex-China, whichever comes last.

Sensitivity analyses

The following sensitivity analyses will be performed:

<u>Primary efficacy endpoint as per investigator</u>

A sensitivity analysis of the primary efficacy endpoint will be performed including any events up to the CSED with final diagnosis by the investigator confirming the event, whether or not confirmed by the CEC. The statistical methodology used will be the same as defined for the primary efficacy analysis.

Since Investigators are requested to report any UA regardless of whether the event fulfils the stricter protocol definition or not, a subset of the reported UA will be selected using information reported on the e-CRF on hospitalization forms, additional information reported on the UA form related to ECG findings, need for revascularization procedure, and/or the concomitance of elevation of cardiac biomarkers (see Appendix C for full details). Since the categorization of deaths as CHD death was not requested from the Investigator, CHD deaths as per Investigator will include all deaths with primary cause of death reported as "Acute myocardial infarction", "Sudden cardiac death", "Heart failure or cardiogenic shock" as per investigator. The category "Undetermined cause of death" as per Investigator will be also included in this endpoint.

The concordance rate between Investigator opinion and adjudication by the CEC will be provided for all CV events adjudicated, by CV event's type.

Primary endpoint analysis excluding undetermined causes of death and undetermined causes of stroke

The primary efficacy analysis will be also performed excluding deaths adjudicated as "undetermined causes of death" and strokes adjudicated as "undetermined causes of stroke" by the CEC.

Supportive analyses

The primary efficacy outcome will be analyzed on randomized and treated patients considering only events that occurred during the treatment period (ie, from the first double-blind IMP injections to the last double-blind IMP injections +21 days, or up to the date of the CSED, whichever comes first), using the same statistical methodology as for the primary efficacy analysis. If a patient does not have a primary endpoint during the treatment period, the patient will be right-censored at the date of last contact when information on efficacy endpoints (presence or absence) has been retrieved, or at the date of death, or at the CSED or at the date of last double-blind IMP injection +21 days, whichever comes first.

Analysis on all events (ie, including recurrent events after the primary efficacy endpoints) will be also performed. Risk ratios between treatments groups will be estimated by Andersen-Gill (4) mean intensity model and the robust sandwich estimate of Lin and Wei (5) for the covariance matrix. Cumulative mean function and 95% CI in each treatment group will be calculated using Nelson-Aalen estimate.

Subgroup analyses

The consistency of the treatment effect on primary efficacy outcome will be evaluated with respect to the following demographic/baseline characteristic and prognostic factors:

- Gender;
- Age group (<65, ≥65);
- Race (Caucasian, Black, Asian/Oriental, and Other, as appropriate);
- Country (IVRS stratum, depending of the size of subgroups);

- Region (USA/Non-USA, and North America/South America/Eastern Europe/Western Europe/Asia/Rest of world);
- Time from ACS event to randomization (eg, ≤24 weeks, >24 weeks).

For each factor except the region, a Cox proportional hazard model will be used, including the treatment, the region, the factor, and the treatment-by-factor interaction terms as covariates. Within each selected factor, the treatment effect hazard ratio and its CI will be estimated from this Cox model. P-values of interaction will be also provided. Results will be plotted using forests plot. The treatment effect by region will be estimated using the similar, Cox proportional model with treatment, region, and treatment-by-region interaction as the covariates.

In addition, Kaplan-Meier curves and summary statistics showing number of patients, number (%) of primary efficacy outcome events, cumulative incidence of events at 6 months and by year, and appropriate CI may be provided for each treatment arm in previously selected subgroups defined above.

In addition, the effect of the time from ACS to randomization (in weeks) will be assessed using a Cox Proportional Hazard model including the time from ACS event (continuous) as a covariate, the treatment group and the interaction.

In addition, homogeneity of treatment effect in the following subgroups will be explored (providing sufficient number of events per subgroups):

- BMI (<30, ≥ 30 kg/m²)
- Age (<65, ≥65 to <75, ≥75)
- Ethnicity (hispanic or latino/not hispanic nor latino)
- Statin treatment at randomization in three categories (high dose atorvastatin/rosuvastatin; any other statin [ie, low/moderate doses of atorvastatin/rosuvastatin, any dose of other statins]; no statin)
- Diabetes mellitus status at baseline (diabetes, pre-diabetes/ normoglyceamic)
- Diabetes mellitus status at baseline (diabetes, pre-diabetes, normoglyceamic)
- Baseline LDL-C (<80, ≥80 to <100, ≥100 mg/dL)
- Index ACS event (STEMI, NSTEMI, UA)
- Prior stroke
- Baseline non-HDL-C (<110, ≥110 to <130, ≥130 mg/dL)
- Baseline Apo B (<75, ≥75 to <90, ≥90 mg/dL)
- Baseline Lp (a) (<50, ≥50 mg/dL)
- Baseline hs-CRP (<2, ≥2 mg/L)

3.4.4.2 Analyses of secondary efficacy endpoints

Method for controlling the overall type-I error rate when testing the main secondary efficacy endpoints is described in Section 3.4.4.3.

Secondary endpoints will be analyzed using the same statistical methodology as for the primary endpoint.

3.4.4.3 Multiplicity issues

In order to handle multiple main secondary endpoints, the overall type-I error will be controlled by the use of a sequential inferential approach. Statistical significance of the primary endpoint is required before drawing inferential conclusions about first main secondary endpoint (at the 0.0001 1-sided alpha level at the second interim analysis or at the 0.0249 1-sided alpha level at the final analysis). Inferential conclusions about successive main secondary parameters require statistical significance of the prior one. The order of tests is detailed in Section 3.1.3.2.1.

This fixed hierarchical approach will ensure a strong control of the overall type-I error rate at the required 1-sided level (0.0001 for the second interim analysis and 0.0249 at the final analysis).

No further adjustments will be made for other secondary endpoints for which p-values will be provided for descriptive purpose only.

3.4.4.4 Additional efficacy analyses

Additional efficacy analyses, endorsed by the steering committee, may be defined in an exploratory SAP, before the unblinding of the treatment code. In particular the relationship between lipid lowering effects and the outcome of cardiovascular efficacy endpoints will be assessed.

Additional analyses will be performed to explore if the randomized treatment effects on cardiovascular efficacy endpoints are statistically different before and after a specified timepoint (e.g. 0-1 year vs. >1 year). These analyses will be performed with Cox proportional hazard models. Specifically, for a given endpoint, the model fit that allows the treatment HR to vary before and after the specified timepoint (with adjustment for region) will be compared to the corresponding model where the treatment HR is not allowed to vary over time.

3.4.5 Analyses of safety data

The summary of safety results will be presented by treatment group.

General common rules

All safety analyses will be performed on the safety population as defined in Section 3.3.2, unless otherwise specified, using the following common rules:

- Safety data in patients who do not belong to the safety population (ie, exposed but not randomized) will be listed separately;
- The baseline value is defined as the last available value before first double-blind IMP injection, except otherwise specified;
- The potentially clinically significant abnormality (PCSA) values are defined as abnormal values considered medically important by the Sponsor according to predefined criteria/thresholds based on literature review and defined by the Sponsor for clinical laboratory tests, vital signs, and ECG (see Appendix A). In case the PCSA threshold is within the normal laboratory ranges, the analysis will be done using "<LLN" or ">ULN" threshold instead of "<PCSA threshold" or ">PCSA threshold" respectively. Of note, for HbA_{1c}, usual PCSA criteria will not be applied as specific analysis for the incidence of diabetes during the TEAE period will be provided, combining information from adverse event, laboratory parameters, and antidiabetic medications (see detail in Section 3.4.5.3).
- PCSA criteria will determine which patients had at least 1 PCSA during the TEAE period, taking into account all evaluations performed during this period, including nonscheduled, local or repeated evaluations.
- The treatment-emergent PCSA denominator by group for a given parameter will be based on the number of patients assessed for that given parameter in the TEAE period by treatment group on the safety population.
- All measurements, scheduled or unscheduled, fasting or not fasting, will be assigned to analysis windows defined in Section 3.5.4 Table 8.
- For quantitative safety parameters based on central laboratory/reading measurements, descriptive statistics will be used to summarize results and change from baseline values by visit (using analysis windows defined in Section 3.5.4 Table 8) and treatment group. Summaries will include the last on-treatment value and the worst on-treatment value.
- The worst value is defined as the nadir and/or the peak value during the treatment period according to the direction (minimum or maximum) of the abnormality as defined in the PCSA list.
- For exploratory purpose, safety analyses could also be provided according to up-titration status, ie, according to whether the patients remained on the 75 mg dose or whether they were up-titrated to 150 mg. These analyses will be exploratory and descriptive (no formal comparison per dose) as it is expected that there could be inherent differences in the baseline characteristics between those patients titrating to 150 mg and those remaining on 75 mg. In order to reduce the bias of this analysis, the period before the up-titration for patients up-titrated and the period before the first up-titration time point (ie, Month 2) for patients not up-titrated will not be included in the analysis since only the dose 75 mg is proposed for this time period and consequently the early events can only be attributed to

this dose. Therefore the descriptive analysis per dose will include any safety events occurring from the first injection post up-titration time point IVRS/IWRS transaction to the end of the TEAE period or to 70 days after down-titration to 75 mg (if any), whichever comes first. Event-rate per patient-year will also be provided after up-titration time point to take into account variable duration of exposure.

- Analyses performed according to diabetes mellitus status at baseline will be done using the definition provided in Section 3.1.1.

3.4.5.1 Analyses of adverse events

Generalities

The primary focus of adverse event reporting will be on TEAEs. Pre- and post-treatment adverse events will be described separately.

If an adverse event date/time of onset (occurrence, worsening, or becoming serious) is incomplete, an imputation algorithm will be used to classify the adverse event as pre-treatment, treatment-emergent, or post-treatment. The algorithm for imputing date/time of onset will be conservative and will classify an adverse event as treatment emergent unless there is definitive information to determine it is pre-treatment or post-treatment. Details on classification of adverse events with missing or partial onset dates are provided in Section 3.5.3.

Adverse event incidence tables will present by SOC, HLGT, HLT, and PT, sorted in alphabetical order for each treatment group, the number (n) and percentage (%) of patients experiencing an adverse event. Multiple occurrences of the same event in the same patient will be counted only once in the tables within a treatment phase. The denominator for computation of percentages is the safety population within each treatment group.

Sorting within tables should ensure the same presentation for the set of all adverse events within the observation period (pre-treatment, treatment-emergent, and post-treatment). For that purpose, the table of all TEAEs presented by SOC and PT sorted by the internationally agreed SOC order and decreasing frequency of PTs within SOCs will define the presentation order for all other tables unless otherwise specified. Sorting will be based on results for the alirocumab group.

As recommended by the safety planning, evaluation, and reporting team (SPERT, [6]) the analysis of all TEAEs will be split into 3 tiers for signal detection and analysis of adverse events.

- Tier 1: TEAEs with pre-specified detailed analysis: TEAEs for which hypothesis and comprehensive analytical approach are prospectively defined.
- Tier 2: signal detection among common TEAE: (not prespecified).
- Tier 3: descriptive analysis of infrequent TEAEs: TEAEs which are infrequent. This could include some AESI predefined in the protocol (eg, pregnancy, hemolytic anemia) to ensure close monitoring but which are expected to be so rare that statistical analysis is not meaningful. For those events, medical judgment should prevail.

Prospective analysis for Tier 1 events

Tier 1 events will include the AESIs and grouping of adverse events as defined in Section 3.1.4.1. Some of these events may be analyzed as Tier 3 in case their occurrence is infrequent. For each selected Tier 1 event, comprehensive analytic approach will be conducted, as described below, in order to evaluate whether the incidence is higher in the alirocumab group versus the placebo group.

Descriptive summaries of Tier 1 events

The number (%) of patients with an event in the TEAE period will be summarized in each treatment group: 95% CIs of the incidence rate (%) will be provided (CI calculated using the mid-p method) for each Tier 1 grouping of terms. The event rate per 100 patient-years (the number of patients with an event in question divided by total 100 patient-years), as well as 95% CI will be also provided. For a patient with an event, patient year is censored at time of first event; for patient without event, it corresponds to the length of the TEAE period. If the event is defined as a grouping of terms, the table will be presented by SMQ/CMQ and PT (when selection is based on SMQ/CMQ) and by PT (when selection is based on the e-CRF tick box or HLGT/HLT), showing the number (%) of each PT included in the grouping of terms. The summaries will be sorted by decreasing incidence of PT within each SMQ/CMQ (in the alirocumab group). In addition, above description will be provided according to diabetes mellitus status at baseline for the diabetes mellitus or diabetic complications grouping.

An overview of each Tier 1 TEAE will be also provided in each treatment group: number (%) of TEAE, of treatment-emergent SAE, of TEAE leading to death and of TEAE leading to permanent treatment discontinuation. In addition, a summary of the following characteristics at grouping level will be provided: the severity grade (mild, moderate, severe), the outcome (Recovered/Resolved, Recovering/Resolving, Unknown, Recovered/Resolved with sequelae, Stabilized, Not recovered/Not Resolved, Fatal), the seriousness, the outcome status of Tier 1 TEAE leading to premature treatment discontinuation. In addition, summary by SMQ/CMQ and PT will be provided for serious TEAEs and TEAEs leading to permanent treatment discontinuation.

Time to liver-related treatment discontinuation and time to liver death may also be provided using hepatic disorder SMQ.

Additional statistical analyses for Tier 1

In order to compare treatment groups, the hazard ratio (HR) will be provided together with the corresponding 95% CI. Hazard ratio will be calculated using a Cox model. Patient without any event will be censored at the end of the TEAE period.

Kaplan-Meier curves for time from first dose of double-blind IMP to the first occurrence of Tier 1 TEAE will be provided for each Tier 1 (grouping of terms). Patient without any event will be censored at the end of the TEAE period.

In addition, HR and Kaplan-Meier curves and estimates will be provided according to diabetes mellitus status at baseline for the diabetes mellitus or diabetic complications grouping.

To assess the homogeneity of the treatment effect across age groups (<65 years versus ≥65 years, and <75 years versus ≥75 years), the treatment-by-age interaction will be tested in a Cox model including the age factor term and the treatment-by-age interaction term. Hazard ratio and the corresponding 95% CI within each age subgroup (calculated using a Cox model), as well as the significance level of the treatment-by-age interaction term will be also provided for descriptive purpose.

Additional summaries for local injection site reaction

The following description of local injection site reaction will be tabulated:

- Number of local injection site reaction per patient: 1, >1;
- Mean duration;
- Number of events divided by the number of double-blind IMP injections received;
- Time from first double-blind IMP injection to first local injection site reaction;
- Number of double-blind IMP injections received up to the first event;
- Intensity of the event (mild, moderate, severe);
- Description of the highest intensity of each symptom recorded in the specific e-CRF page.

Analysis of all "common" TEAE(s) - Tier 2 events

"Common" events are defined as those for which there are more (>) than n patients with an event overall in the safety population. This threshold will be defined as the number of patients with events (n) observed overall (whatever the treatment group) for which the extreme case scenario (n for alirocumab versus 0 for the placebo) doesn't allow the p-value to be less than 0.05.

All common TEAEs (by HLT and PT), showing the number (%) of TEAE, the event rate per 100 patient-years, HR (estimated using a Cox model) with the corresponding 95% CI, sorted by decreasing incidence rate in alirocumab group in one table, and by decreasing HR in the another table, will be analyzed.

If any clinically significant signal is detected and need further characterization, additional analyses similar to Tier 1 analyses, will be provided.

Descriptive analysis of infrequent adverse events – Tier 3 events

All infrequent TEAEs (Tier 3) will be reported with descriptive statistics (n, %) and event rate per 100 patient year, without comparative statistics since with so rare event statistical comparisons are not meaningful and medical judgment should prevail.

Analysis of all treatment-emergent adverse events

The following TEAE summaries, including all TEAEs (common or not, Tier 1 or not) will be generated for the safety population.

- Overview of TEAEs, summarizing number (%) of patients with any:
    - TEAE;
    - Serious TEAE;
    - TEAE leading to death;
    - TEAE leading to permanent treatment discontinuation.
- All TEAEs by primary SOC, HLGT, HLT, and PT, sorted by the SOC internationally agreed order. The other levels (HLGT, HLT, PT) will be presented in alphabetical order;
- All TEAEs regardless of relationship and related to IMP by primary SOC, HLGT, HLT, and PT, sorted by the internationally agreed SOC order. The other levels (HLGT, HLT, PT) will be presented in alphabetical order;
- All TEAEs regardless of relationship and related to statin/other lipid lowering drug by primary SOC, HLGT, HLT, and PT, sorted by the internationally agreed SOC order. The other levels (HLGT, HLT, PT) will be presented in alphabetical order;
- All TEAEs by primary SOC and PT, sorted by the internationally agreed SOC order and by decreasing incidence of PTs within each SOC (in the alirocumab group). This sorting order will be applied to all other tables by SOC and PT of TEAEs, unless otherwise specified. The event rate per 100 patient-years (the number of patients with an event in question divided by total patient-years) will be provided for all TEAEs by SOC and PT. For a patient with event, patient-year is censored at time of first event; for patient without event, it corresponds to length of TEAE period;
- All TEAEs that occurred with HLT and PT incidence ≥2% in alirocumab group and at incidence at least 0.5% higher in alirocumab than placebo, by primary SOC, HLT, and PT;
- All TEAEs that occurred with incidence ≥5% in any treatment group, by primary SOC and PT, with event rate per 100 patient-years;
- All TEAEs by maximal severity (ie, mild, moderate, or severe), presented by primary SOC and PT.

Subgroup of patients with 2 consecutive LDL-C <25 mg/dL (<0.65 mmol/L)

A 2-step approach will be used to analyze the safety in relation to low LDL-C.

The first step will screen events for potential signal using 2 approaches. The first approach will be a direct comparison of patients with 2 consecutive LDL-C <25 mg/dL versus ≥25 mg/dL (ie, patients without 2 consecutive LDL-C <25 mg/dL) within the alirocumab treatment group. Since these 2 groups are based on post-randomization data with a potential for bias, a second approach will compare the alirocumab effect versus placebo according to categories based on baseline LDL-C. The frequency of patients with 2 consecutive LDL-C <25 mg/dL is expected to be the largest in the category with the lowest baseline LDL-C and be lower and lower in the categories with higher baseline LDL-C. Therefore, an event induced by low LDL-C should be associated with a higher alirocumab effect versus placebo (ie, higher HR) in the first baseline LDL-C category(ies) than in the subsequent categories. Details of these 2 approaches are provided below.

The second step will consist in the comparison of patients with 2 consecutive LDL-C <25 mg/dL versus ≥25 mg/dL within the alirocumab group for the events detected in the first step, as well as for pre-specified events.

Similar analyses will be provided considering 15 mg/dL (0.39 mmol/L) as threshold instead of 25 mg/dL.

First step

First screening approach

TEAE summary by primary SOC, HLGT, HLT, and PT as well as groupings of events (ie, cataract, neurological events, neurocognitive disorders and "new onset of diabetes" [see Section 3.4.5.3 for the definition]) will be provided on the safety population in the groups below:

- Placebo group
- Alirocumab group
- Alirocumab LDL-C ≥25 mg/dL (ie, alirocumab patients without 2 consecutive LDL-C <25 mg/dL)
- Alirocumab patients with 2 consecutive LDL-C <25 mg/dL

For patients with 2 consecutive LDL-C <25 mg/dL, analyses will be done on the period starting from the first of the 2 consecutive LDL-C lower than 25 mg/dL to the upper limit of the TEAE period excepted for patients down-titrated from 150 mg to 75 mg for whom the analysis period will end at the date of last injection of 150 mg +70 days (as patients down-titrated from 150 mg to 75 mg are likely to come back above 25 mg/dL).

The time to the first TEAE/event will be compared for patients with 2 consecutive LDL-C <25 mg/dL versus ≥25 mg/dL, within the alirocumab group, using a Cox model, including the covariate for 2 consecutive LDL C <25 mg/dL (Yes/No). Hazard ratio (and 95% CI) from this model will be provided. The event rate per 100 patient-years (the number of patients with an event in question divided by total 100 patient-years) will also be provided.

Second screening approach

TEAE summary by primary SOC, HLGT, HLT, and PT as well as groupings of events (ie, cataract, neurological events, neurocognitive disorders and "new onset of diabetes" will be provided on the safety population according to baseline LDL-C categories (eg, <70, ≥70 to <90, ≥90 to <110, ≥110 mg/dL).

Hazard ratio (and 95% CI) for alirocumab effect versus placebo within each baseline LDL-C subgroup will be provided using a Cox model with baseline LDL-C (in categories), treatment group and the treatment-by-baseline LDL-C interaction term.

To assess the impact of baseline LDL-C on hazard ratio, a Cox model including the baseline LDL-C (as continuous factor), treatment group and treatment-by-baseline LDL-C interaction term will be used. The p-value from the interaction term will be provided for descriptive purpose to evaluate if there is a potential relationship between baseline LDL-C and hazard ratio.

Second step

For each pre-specified event (cataract, neurological events, neurocognitive disorders, new onset of diabetes) as well as for each event with a potential signal detected in the first step, the time to the first TEAE/event will be compared for patients with 2 consecutive LDL-C <25 mg/dL versus ≥25 mg/dL, within the alirocumab group, using a Cox model, including the covariate for 2 consecutive LDL-C <25 mg/dL (Yes/No) and prognostic factors of the event analyzed. Adjusted HR (and 95% CI) from this model will be provided.

The list of prognostic factors will be established based on the literature and on study data (as applicable).

Subgroups of patients with treatment-emergent ADA positive response

All TEAEs by primary SOC, HLGT, HLT, and PT as well as local injection site reactions will be described in the alirocumab group according to the following ADA parameters:

- Treatment-emergent ADA positive response (yes/no)
- Persistent/transient/indeterminate treatment-emergent ADA positive response.

Analysis of all treatment-emergent serious adverse event(s)

- All treatment-emergent SAEs by primary SOC, HLGT, HLT, and PT, sorted by the internationally agreed SOC order. The other levels (HLGT, HLT, PT) will be presented in alphabetical order;
- All treatment-emergent SAEs regardless of relationship and related to IMP, by primary SOC, HLGT, HLT, and PT, sorted by the internationally agreed SOC order. The other levels (HLGT, HLT, PT) will be presented in alphabetical order;
- All treatment-emergent SAEs by primary SOC and PT.

Analysis of all treatment-emergent adverse event(s) leading to permanent treatment discontinuation

- All treatment-emergent adverse events leading to treatment discontinuation, by primary SOC, HLGT, HLT, and PT, sorted by the internationally agreed SOC order. The other levels (HLGT, HLT, PT) will be presented in alphabetical order;
- All TEAEs leading to treatment discontinuation by primary SOC and PT.

Analysis of pre-treatment and post-treatment adverse events

- All pre-treatment adverse events by primary SOC and PT sorted by the internationally agreed SOC order and decreasing incidence of PTs within each SOC;
- All pre-treatment adverse events leading to treatment discontinuation (if any) by primary SOC and PT, sorted by the internationally agreed SOC order and decreasing incidence of PTs within each SOC;
- All post-treatment adverse events by primary SOC and PT, sorted by the internationally agreed SOC order and decreasing incidence of PTs within each SOC;
- All post-treatment SAEs by primary SOC and PT, sorted by the internationally agreed SOC order and decreasing incidence of PTs within each SOC.

3.4.5.2 Deaths

The following summaries of deaths will be generated for the safety population.

- Number (%) of patients who died from the first IMP injection until CSED and reasons for death as adjudicated by the CEC;
- Deaths occurring after CSED (adjudicated or not);
- Treatment-emergent adverse events leading to death (death as an outcome on the adverse event case report form page as reported by the Investigator) by primary SOC, HLGT, HLT, and PT sorted by internationally agreed SOC order, with HLGT, HLT, and PT presented in alphabetical order within each SOC;

- All post-treatment adverse events leading to death by primary SOC and PT, sorted by the internationally agreed SOC order and decreasing incidence of PTs within each SOC;
- In addition, deaths in non-randomized patients or randomized but not treated patients will be displayed.

3.4.5.3 Analyses of laboratory variables

Descriptive statistics over time

The summary statistics (including number, mean, median, Q1, Q3 standard deviation, minimum, and maximum) of all laboratory variables (central laboratory values and changes from baseline) will be calculated for each visit or study assessment (baseline, each post-baseline time point, last on-treatment and worst on-treatment value) by treatment group during the treatment period.

For glucose, this summary will be also provided according to the diabetes status at baseline. Only fasting samples will be summarized.

In addition, for some parameters of interest, mean changes from baseline with the corresponding standard error could be plotted over time in each treatment group.

Potentially clinically significant abnormalities

The incidence of PCSAs (list provided in Appendix A) as well as ALT increase as defined as AESI and hemoglobin decrease from baseline ≥15 g/L at any time during the TEAE period will be summarized by biological function and treatment group whatever the baseline level and/or according to the following baseline status categories:

- Normal/missing;
- Abnormal according to PCSA criterion or criteria.

For glucose, this summary will also be provided according to the diabetes status at baseline. Only fasting samples will be summarized.

For parameters for which no PCSA criteria are defined, similar table(s) using the normal range will be provided.

PCSA summaries will also be provided in patients from alirocumab group with 2 consecutive LDL-C <25 mg/dL in case a signal detected in the adverse events analyses (see Section 3.4.5.1) warrants further investigations. Only PCSA occurring after the first occurrence of LDL-C <25 mg/dL will be considered (see Section 3.4.5.1, sub-section “Subgroup of patients with two consecutive LDL-C <25 mg/dL (<0.65 mmol/L)” for the definition of the analysis period).

Analysis of new onset of diabetes (NOD)

The incidence of new onset of diabetes during the TEAE period will be analyzed in the subgroup of patients not having diabetes at baseline as well as in the subgroups of patients with pre-diabetes and normal glycemic at baseline (see Section 3.1.1). New onset diabetes will be defined as follows, combining information from adverse events, medication, and laboratory parameters:

- Type 1 or 2 diabetes TEAE (CMQ "Type 1 or type 2 diabetes", Table 11).
- And/or anti-diabetic medication initiated during the TEA E period with a confirmed diagnosis per the external diabetes experts *.

  * Patients classified as NOD based only on the use of anti-diabetic medication during the TEAE period will be reviewed in a blinded manner by external experts in diabetology. If the diabetic mellitus status is not confirmed, the patients will not be classified as NOD.
- And/or at least 2 HbA_{1c} ≥6.5% during the TEAE period
  - For patients with a single measurement available during the TEAE period, a single value ≥6.5% will be considered and qualify the patient as NOD by default
  - For patients with several HbA1c measurements but only with the last one ≥6.5%, this single value ≥6.5% will be considered and qualify the patient as NOD by default.
- And/or at least two fasting glucose measurements ≥126 mg/dL (7.0 mmol/L):
  - For patients with only a single measurement available during the TEAE period, a single value ≥126 mg/dL (7.0 mmol/L) will NOT be considered and will NOT qualify the patient as NOD.
  - For patients with several fasting glucose measurements but only with the last one ≥126 mg/dL (7.0 mmol/L), this single value ≥ 126 mg/dL (7.0 mmol/L) will NOT be considered and will NOT qualify the patient as NOD.

Hepatitis C antibody

The number and percentage of patients with a post-baseline seroconversion for hepatitis C test will be provided by treatment group in post-baseline (including the TEAE and post TEAE periods). Post-baseline seroconversion is defined for patients with a negative baseline status who had either a “positive ribonucleic acid” (RNA) or a “confirmed positive antibody with negative RNA” post-baseline status as defined in the table below. Other situations require case by case evaluation and will be described individually if relevant.

The status as regards to hepatitis C virus (HCV) for a patient will be defined as follows for all evaluations (baseline and post-baseline).

Table 6 - Definition of the patient status regarding hepatitis C virus

	Hepatitis C Antibody (Ab) test result				
	Negative		Positive		
Reflexive test[a] – hepatitis C RNA test	Not available or HCV RNA not detected	HCV RNA detected	HCV RNA not detected[b]	HCV RNA detected	Not available
Hepatitis C status - label	Negative	Positive RNA	Negative[b]	Positive RNA	Positive Ab – no RNA available

a Test performed at the same time or after the antibody test in the pre-treatment period (for baseline evaluation), or post-baseline, respectively

b For post-baseline evaluation, a second antibody test with a different type of assay is to be done at the same date or after the first antibody test. The result of this test will modify the final hepatitis C status of the patient in some cases (see details in the text below the table)

The baseline evaluation will be based on tests performed during the pre-treatment period.

In case of multiple hepatitis C tests available for the post-baseline evaluation, the positive status of the patient will be defined as follows:

- “Positive RNA” status if at least 1 post-baseline positive RNA is detected, regardless of status of the patient at the end of treatment.
- Else “Positive Ab – no RNA available” status if no post-baseline reflexive RNA test is available for at least 1 post-baseline positive antibody test.

If no antibody test is available or with “indeterminate” as result pre-treatment or post-baseline, respectively, the RNA test (if available) will be used alone to determine the status of the patient. If no RNA is available then the hepatitis C status of the patient will be missing.

The post-baseline status “confirmed positive antibody with negative RNA” will replace “Negative” status as defined above in the case where no RNA was detected post-baseline and the 2 antibody tests surrounding the same visit (from 2 different types of assay) are positive.

For a conservative approach, the post-baseline status “Positive Ab – no RNA available” will not be modified by the availability of a second antibody test from a different assay.

Possible drug-induced liver injury

The liver function tests, namely AST, ALT, alkaline phosphatase, and total bilirubin, are used to assess possible drug-induced liver toxicity. The proportion of patients with PCSA values at any post-baseline visit by baseline status will be displayed by treatment group for each parameter.

A graph of distribution of peak values of ALT versus peak values of total bilirubin will also be presented. Note that the ALT and total bilirubin values are presented on a logarithmic scale. The graph will be divided into 4 quadrants with a vertical line corresponding to 3 x ULN for ALT and a horizontal line corresponding to 2 x ULN for total bilirubin.

Graph and listing of possible Hy's law cases identified by treatment group (eg, patients with any elevated ALT>3 x ULN, and associated with an increase in bilirubin ≥2 x ULN) with ALT, AST, alkaline phosphatase, total bilirubin, and, if available, direct and indirect bilirubin, will be provided.

3.4.5.4 Analyses of vital sign variables

The summary statistics (including number, mean, median, Q1, Q3, standard deviation, minimum, and maximum) of all vital signs variables (values and changes from baseline) will be calculated for each visit or study assessment (baseline, each post-baseline time point, last on-treatment and worst on-treatment value) by treatment group during the treatment period. In addition, for some parameters of interest, mean changes from baseline with the corresponding standard error could be plotted over time in each treatment group.

The incidence of PCSAs at any time during the TEAE period will be summarized by treatment group irrespective of the baseline level and/or according to the following baseline status categories:

- Normal/missing;
- Abnormal according to PCSA criterion or criteria.

3.4.6 Analyses of other safety parameters

Events initially suspected by investigators, and reported as such in endpoints forms, may finally not be confirmed by investigators and possibly classified into another category that is not a component of the primary efficacy endpoint. In addition, per protocol all suspected UAs have to be sent for adjudication regardless of whether the event fulfils the stricter protocol definition or not, therefore a high proportion of the reported UAs are expected to be finally not retained as an endpoint.

Since those events are not reported as adverse events either, they will be described separately as follows:

The number (%) of patients with events below during the TEAE period will be displayed by treatment group on the safety population:

- With final diagnosis as per investigator of stable coronary disease,
- With final diagnosis as per investigator of unstable angina regardless of whether the event fulfils the stricter protocol definition or not;
- With final diagnoses as per investigator of hemorrhagic stroke, transient ischemic attack (TIA), and subdural hematoma.

The number (%) of patients with hemorrhagic strokes or silent MI as per CEC during the TEAE period will also be summarized on the safety population

3.4.7 Analyses of other endpoints

All measurements, scheduled or unscheduled, fasting or not fasting, will be assigned to analysis windows defined in Section 3.5.4, in order to provide an assessment for Month 1 to Month 64 time points.

3.4.7.1 Analyses of hs-CRP

hs-CRP parameter will be summarized on the safety population by analysis visit using number of available data, mean, SD, median, Q1, Q3, minimum, and maximum for each treatment group during the treatment period. The time profile will be plotted by treatment group with the medians, Q1 and Q3. The incidence of PCSA at any time during the TEAE period will be summarized by treatment group using descriptive statistics.

hs-CRP values greater or equal to 10 mg/L will be excluded from analyses in a second approach, since these are suggestive of concurrent infections.

3.4.7.2 Analyses of HbA_{1c}

HbA_{1c} parameter will be summarized on the safety population by analysis visit using number of available data, mean, SD, median, minimum, and maximum for each treatment during the treatment period. Summary will be also provided according to the diabetes mellitus status at baseline (see Section 3.1.1). The time profile will be plotted by treatment group with the means and the corresponding standard errors (SEs).

In case the proportion of initiation of anti-diabetic medications is different between the 2 treatment groups, further analysis of HbA_{1c} over time would be performed.

3.4.7.3 Analyses of patients with LDL-C <25 mg/dL(<0.65 mmol/L)

The number and percentage of patients with 2 consecutive LDL-C <25 mg/dL (respectively, LDL-C <15 mg/dL, ie, 0.39 mmol/L) will be provided by treatment group on the safety population. Kaplan-Meir curves will be provided for the time to the first LDL-C <25 mg/dL (respectively 15 mg/dL) for these patients. For this analysis, patients without post-baseline LDL-C result or with only 1 post-baseline LDL-C result will not be included.

3.4.7.4 Analyses of lipid parameters

The lipids variables (see Section 3.1.5.1) will be analyzed using an ITT approach (based on the ITT population) including all lipid values, regardless of whether the patient was continuing therapy or not. In addition, analyses will also be conducted using an on-treatment approach (based on the randomized and treated population) only including lipid data collected during the treatment period.

3.4.7.4.1 ITT analyses

A pattern-mixture model approach (see Appendix F) will be used with a different imputation strategy applied for missing lipid values during the treatment period (ie, within the time period from the first double-blind IMP injection up to the day of the last double-blind injection +21 days) and missing lipid values after treatment discontinuation (ie, after the day of last injection +21 days) based on the following assumptions:

- Patients within 21 days of their last double-blind IMP injection would continue to show benefit from treatment similar to that observed at the scheduled time point. Therefore, lipid values missing during the treatment period (samples obtained outside the specified window, no blood sample available although visit was performed, etc) should be considered "Missing At Random" and imputed based on other on-treatment measurements;
- Patients who stopped taking their study treatment no longer benefited from it after discontinuation and thus tended to have lipid values returning to baseline. Thus lipid values missing after treatment discontinuation will be imputed based on patient's own baseline value.

Missing lipid values will be imputed 10 times using the MI SAS® procedure, to generate 10 complete data sets. The percent change from baseline and/or the absolute change from baseline at a pre-specified time point will be derived from observed and imputed lipid value at this time point. Imputed values for time points after CSED will be discarded.

TGs and Lp (a) data will be log-transformed before imputation process and then back-transformed to create the imputed data sets.

The completed data sets will be analyzed using an analysis of covariance (ANCOVA) model for continuous lipid variables other than Lp (a) and TGs or a robust regression (6) model for Lp (a) and TGs continuous variables with treatment group as fixed effect, and the baseline lipid value as continuous covariate. The MIANALYZE procedure will be used to generate valid statistical inferences by combining results from the 10 analyses using Rubin's formula.

The number of imputations (10) will be informally verified by replicating sets of 10 imputations and checking whether the combined results are stable. If not stable, the number of imputations will be increased and informally checked as above, and thus until stable estimates are obtained.

The value at the CSED will be the value obtained at the CSED visit. For patients without CSED visit, the last lipid value observed or imputed up to CSED will be taken into account.

Throughout the ANCOVA and robust regression models, the alirocumab group will be compared to placebo using appropriate contrasts tested at the two-sided 0.05 level, and providing the 95% CI of the difference, for the different time points as well as at the CSED.

No adjustment will be made for lipid variables for which p-values will be provided for descriptive purpose.

3.4.7.4.2 On-treatment analyses

Analysis of lipid variables will be conducted during the treatment period. Post-treatment data will not be considered.

The lipid variables other than Lp (a) and TGs will be analyzed in the randomized and treated population using a mixed-effect model with repeated measures (MMRM) approach. All post-baseline on-treatment data available within Month 1 to Month 64 analysis windows will be used and missing data will be accounted for by the MMRM model.

The model will include the fixed categorical effects of treatment group (placebo, alirocumab), planned time point (Month 1 to Month 64), treatment-by-time point interaction, as well as, the continuous fixed covariate of baseline lipid value and baseline lipid value-by-time point interaction. This model will be run using SAS Mixed procedure with an unstructured correlation matrix to model the within-patient errors. Parameters will be estimated using restricted maximum likelihood method with the Newton-Raphson algorithm. Denominator degrees of freedom will be estimated using Satterthwaite's approximation. This model will provide baseline adjusted least-squares means estimates over time for both treatment groups with their corresponding SEs and 95% CI. To compare the alirocumab group to the placebo group, appropriate contrasts statement will be used to test the differences of these estimates, at the 2-sided 0.05 level, for the different time points.

Note: in case of computation issue for this approach, multiple imputation under missing-at-random (MAR) assumption will be conducted, followed by an ANCOVA model.

The Lp (a) and TGs will be analyzed in the randomized and treated population using multiple imputation (same imputations as in Section 3.4.7.4.1 without discarding imputations of missing values during the post-treatment period (see Appendix F).

3.4.7.5 Analyses of anti-alirocumab antibody variables

The following summaries will be performed on the ADA population, taking into account all samples regardless of timing in relation to injections. ADA results will be summarized by treatment group and up-titration status (see Section 3.4.5).

- ADA results (negative or positive) by time point;
- Neutralizing status (negative or positive) by time point for positive ADA;
- ADA titers using descriptive statistics (median, minimum, and maximum) for positive ADA by time point;
- Number (%) of patients with pre-existing ADA and number (%) of patients with treatment-emergent ADA positive response;
- Number (%) of patients with persistent/transient/indeterminate treatment-emergent ADA positive response;
- Time to onset of treatment-emergent ADA positive response using descriptive statistics;

- Number (%) of patients with at least 1 neutralizing ADA.

Correlations between ADA parameters (eg, titers, treatment-emergent ADA positive status, neutralizing status), safety and/or efficacy endpoints will be also explored (eg, scatter plot).

3.4.7.6 Analyses of quality of life/health economics variables

The analysis of data from EQ-5D instrument will be performed on the ITT population.

Baseline is defined as the Visit 3 (Day 1) evaluation. Analysis window will be used to assign the measurements to time points (see Section 3.5.4).

Individual EQ-5D items

Response for each one of the 5 EQ-5D items will be summarized by time point for each treatment group with number (%) of patients reporting level 1 (no problems), level 2 (some problems), and level 3 (extreme problems) by item.

EQ-5D utility score

The raw value and the change from baseline of the utility score will be summarized using mean, median, Q1, Q3, SD, minimum, and maximum for each post-baseline visit. Cumulative distribution functions for the change in utility score from baseline will be displayed by treatment groups over time.

The change from baseline in utility score over time will be analyzed using a MMRM model with fixed categorical effects of treatment group, planned time points up to the CSED, treatment-by-time point interaction, as well as, the continuous fixed covariates of baseline value and baseline value-by-time point interaction.

Note: In case of computation issue for this approach, multiple imputation under MAR assumption will be conducted, followed by an ANCOVA model.

3.4.7.7 Analysis of cardiovascular events of interest (other than efficacy endpoint)

Other cardiovascular events of interest (see Section 3.1.5.7) will be analyzed using a time-to-event approach (Kaplan-Meier methodology) in the ITT population. Patients without any event will be censored using the same methodology as for the primary efficacy endpoint.

3.4.8 Analyses of pharmacokinetic and pharmacodynamic variables

Not applicable.

3.5 DATA HANDLING CONVENTIONS

3.5.1 General conventions

The following definitions/formulas will be used for computation of parameters.

Common study end date

The common study end date is defined as the date when 1613 patients have experienced at least 1 primary efficacy event or 24 months after the last date of randomization ex-China, whichever comes last.

Date of last dose of investigational medicinal product

The date of the last dose of IMP is equal to the last date of administration reported on the IMP administration case report form page, or missing if the last administration date is unknown. For patients on the alirocumab arm who will switch to placebo injection due to blinded treatment discontinuation, the date of last administration reported associated to an active injection will be considered.

Renal function formulas

eGFR value will be derived using the Modification of the Diet in Renal Disease (MDRD) equation:

175 x (creatinine in µmol/L / 88.4)$^{-1.154}$ x (age in years)$^{-0.203}$ (x 0.742 if female, x 1.212 if race is "black or african american").

Lipids variables, laboratory safety variables, hs-CRP

For data below the lower limit of quantification (LLOQ)/limit of linearity, half of the lower limit value (ie, LLOQ/2) will be used for quantitative analyses. For data above the upper limit of quantification (ULOQ) / limit of linearity, the upper limit value (ie, ULOQ) will be used for quantitative analyses.

The above rules won't be applied for the calculated LDL-C and non-HDL-C when HDL-C value is below the LLOQ. The value of LLOQ/2 for HDL-C will be used to obtain the non-HDL-C and calculated LDL-C used for quantitative analyses.

Below is an example of data for a dummy patient reported in the database, with the values that will be used in quantitative analyses for each parameters.

Table 7 - Example of lipid data for a dummy patient

Parameter	Value reported in the database	Value used in the analysis
TC	255 mg/dL	255 mg/dL
HDL-C	<10 mg/dL	5 mg/dL
Calculated LDL-C[a]	<221 mg/dL	216 mg/dL
NON-HDL-C	<255 mg/dL	250 mg/dL
TRIG	172 mg/dL	172 mg/dL

a Friedewald formula for calculated LDL-C (when lipid expressed in mg/dL: LDL-C=NON-HDL-C-0.2*TG)

3.5.2 Data handling conventions for secondary efficacy variables

Rules defined for the primary efficacy variable will apply to time-to-event secondary efficacy variables.

3.5.3 Missing data

For categorical variables, patients with missing data are not included in calculations of percentages unless otherwise specified. When relevant, the number of patients with missing data is presented.

Handling of missing or incomplete dates of primary endpoint events

Rules for imputations are detailed in Table 4.

Handling of baseline definition if time of first double-blind injection or time of assessment at visit 3 is missing

If the time of the first double-blind injection or the time of assessment at Visit 3 is missing then the baseline value is defined as the last available value obtained before or on the day of the first double-blind IMP injection.

Handling of computation of treatment duration and compliance if IMP first or end of treatment date is missing

If the IMP first or end of treatment date is missing, the exposure duration and compliance will be left as missing.

Handling of treatment/TEAE analysis periods and survival analysis if IMP end of treatment date is unknown

If the last injection (last active injection for patients randomized in the alirocumab group, last injection from a double-blind kit for patients randomized in the placebo group) date is missing or incomplete, this date will be imputed to the earliest of the dates below to define the upper bound of the treatment/TEAE analysis periods and to define the censoring date for survival analyses performed on these periods:

- The last day of the month and year, when only the day is missing, or the 31st of December of the year, when only the year is known;
- The date of the end of treatment visit (CSED visit for completer, early end of treatment visit for patients who prematurely discontinued the IMP);
- The date of the last contact;
- The date of death (if any);
- The day before the date of first injection of placebo following the switch to placebo in IVRS (if any).

Exception: In case the last active injection for a patient allocated to the alirocumab group was inadvertently received after the first injection of placebo following the switch to placebo in IVRS, the last active injection date (missing or incomplete) will be imputed to the earliest of the dates below:

- The last day of the month and year, when only the day is missing, or the 31st of December of the year, when only the year is known;
- The date of the end of treatment visit (CSED visit for completer, early end of treatment visit for patients who prematurely discontinued the IMP);
- The date of the last contact;
- The date of death (if any);
- The day before the date of the placebo injection (if any) following the last active injection.

Handling of medication missing/partial dates

No imputation of medication start/end dates or times will be performed. If a medication date or time is missing or partially missing and it cannot be determined whether it was taken prior or concomitantly, it will be considered a prior, concomitant, and post-treatment medication, unless otherwise specified.

Handling of adverse events with missing or partial date/time of onset

Missing or partial adverse event onset dates and times will be imputed so that if the partial adverse event onset date/time information does not indicate that the adverse event started prior to treatment or after the TEAE period, the adverse event will be classified as treatment-emergent. No imputation of adverse event end dates/times will be performed. These data imputations are for categorization purpose only and will not be used in listings. No imputation is planned for date/time of adverse event resolution.

Handling of adverse events when date and time of first IMP administration is missing

When the date and time of the first IMP administration is missing, all adverse events that occurred on or after the day of randomization will be considered as TEAEs.

Handling of missing assessment of relationship of adverse events to IMP

If the assessment of the relationship to IMP is missing for an adverse event, the adverse event will be considered as related to the IMP in the tables of possibly related adverse events, but no imputation will be done at the data level

Handling of potentially clinically significant abnormalities

If a patient has a missing baseline value he/she will be grouped in the category "normal/missing at baseline."

For PCSAs with 2 conditions, one based on a change from baseline value or a normal range and the other on a threshold value, with the first condition being missing, the PCSA will be based only on the second condition.

For a PCSA defined on a threshold and/or a normal range, this PCSA will be derived using this threshold if the normal range is missing (eg, ">0.5 GIGA/L" criterion will be used for eosinophils for the PCSA ">0.5 GIGA/L or >ULN if ULN ≥0.5 GIGA/L" when ULN is missing.

Measurements flagged as invalid by the laboratory will not be summarized or taken into account in the computation of PCSA values.

3.5.4 Windows for time points

Data analyzed by time point (including lipid data, laboratory safety data, vital signs, ECG, ADA, EQ-5D) will be summarized using the time windows given in Table 8 below. These time windows will be applicable for all analyses, and they are defined to provide more homogeneous data for time point-specific analyses.

Table 8 - Time windows definitions

Time point	Targeted study days	Time windows
Month 1	30	16 to 44
Month 2	60	45 to 75
Month 4	122	107 to 137
Month 8	244	229 to 259
Month 12 to Month 24	Number of months of the planned visit x 30.4375 and rounded to the nearest entire number of days	Targeted study day ±21 days
Beyond Month 24	Number of months of the planned visit x 30.4375 and rounded to the nearest entire number of days	Targeted study day ±28 days

If multiple valid values of a variable exist within a time window, the nearest from the targeted study day will be selected for the statistical analysis by time point. If the difference is a tie, the value after the targeted study day will be used. If multiple valid values of a variable exist within a same day, then the first value of the day will be selected.

3.5.5 Unscheduled visits

For lipid data, safety laboratory data, ECG, or vital signs, unscheduled visit measurements may be used to provide a measurement for a time window, a baseline, a time point, or a worst value, if appropriate according to their definition. The measurements may also be used to determine abnormal/PCSA values.

3.5.6 Pooling of centers for statistical analyses

The randomization scheme was not stratified by center to avoid risk of unbalance between treatment groups within a country induced by the large number of centers that will participate to the study. Nevertheless, as the primary efficacy and main secondary endpoints are centrally adjudicated by the CEC, these outcomes are not expected to be influenced by the center. Therefore, the center will not be added as factor in the primary analysis model.

Centers will be pooled into region (see Table 9) to describe the study population and to perform the primary efficacy analysis and subgroup analyses.

Table 9 - Definition of geographic regions

North America	South America	Western Europe	Eastern Europe	Asia	Rest of the World
Canada	Argentina	Austria	Bosnia Herzegovia	Hong Kong	Australia
United States	Brazil	Belgium	Bulgaria	India	Israel
	Chile	Denmark	Croatia	Japan	New Zealand
	Colombia	Finland	Czech Republic	Korea	Republic of South Africa
	Mexico	France	Estonia	Malaysia	
	Peru	Germany	Georgia	Philippines	
	Guatemala	Italy	Hungary	Singapore	
		Netherlands	Latvia	Sri Lanka	
		Norway	Lithuania	Thailand	
		Portugal	Macedonia	Taiwan	
		Spain	Poland	China	
		Sweden	Romania		
		Switzerland	Russian Federation		
		United Kingdom	Serbia		
		Greece	Slovakia		
			Slovenia		
			Turkey		
			Ukraine		

3.5.7 Statistical technical issues

Not applicable.

4 INTERIM ANALYSIS

Two interim analyses (IA) are planned, when 50% and 75% of the total number of expected events have occurred:

- Interim analysis for futility will be conducted, when approximately 807 events (50% of the targeted number of primary endpoint events) have occurred;
- Interim analysis for futility and overwhelming efficacy will be conducted, when approximately 1210 events (75% of the targeted number of primary endpoint events) have occurred.

Both IAs will be performed by the external independent CV DMC Statistician and will be reviewed under the supervision of the CV DMC. The CV DMC will also review secondary efficacy endpoints and safety data (adverse events, laboratory data, vital signs) available at the time of the IA.

Control of the type I and type II error will be ensured using gamma (-5) spending function for type II error (futility) and Gamma (-22) for type I error (efficacy) at each IA (the type I error spending function is also applied at the first IA, even if the objective of this first IA is only futility). It has to be noted that, in order to protect the global type I error in case the decision is taken to overrule the futility rule, non-binding boundaries were used.

The following table shows the stopping rules at each interim analysis (using the sample size assumptions described in Section 1.3):

Table 10 - Interim analyses stopping boundaries corresponding to Gamma (-22) type I error and Gamma (-5) type II error spending functions

Timing of analyses	Stopping boundaries (1-sided p-value and hazard ratio)	
	Futility	Overwhelming efficacy
First IA: 50% of targeted events	p >0.548<br>(⇔ HR >1.008)	NA
Second IA: 75% of targeted events	p >0.19<br>(⇔ HR >0.951)	p <0.0001[a]<br>(⇔ HR <0.802)

HR = hazard ratio; IA = interim analysis; NA = not applicable

a Should the second interim analysis be triggered just before or after 1210 events have been reached, the exact nominal significance level to be used at the second IA would be re-computed based on a Gamma(-22) spending function.

Calculations done using EAST® 5.4

The CV data monitoring committee (DMC) could consider early stopping of the study for overwhelming efficacy at the second IA, if the following conditions are met:

- Stopping boundaries for overwhelming efficacy are crossed;
- Positive trend observed for secondary efficacy endpoints, including all cause mortality, and no excess of non-CV mortality;
- Consistency of the treatment effect on the primary efficacy endpoint across the following subgroups: gender, age, race, country (depending on the size of subgroups), time from index ACS event to randomization, and regions (see Section 3.4.4.1).

(electronic 3.0)

5 DATABASE LOCK

The final database is planned to be locked approximately 3 months after the last patient last visit.

6 SOFTWARE DOCUMENTATION

All summaries and statistical analyses will be generated using SAS version 9.1 or higher.

Sample size calculations were done using EAST® 5.4 version.

7 REFERENCES

1. Cannon CP, Braunwald E, McCabe CH, Rader DJ, Rouleau JL, Berlder R, et al. Intensive versus moderate lipid lowering with statins after acute coronary syndromes. N Engl J Med. 2004 Apr 8;350(15):1495-504.

2. Pearson TA, Mensah GA, Alexander RW, Anderson JL, Cannon RO 3rd, Criqui M, et al. Markers of inflammation and cardiovascular disease: application to clinical and public health practice: A statement for healthcare professionals from the Centers for Disease Control and Prevention and the American Heart Association. Circulation. 2003 Jan 28;107(3):499-511.

3. Dolan P. Modeling valuations for EuroQol health states. Med Care. 1997 Nov;35(11):1095-108.

4. Andersen PK, Gill RD. Cox's Regression Model for Counting Processes: a large sample study. The Annals of Statistics 1982 Dec;10(4):1100-20.

5. Lin DY, Wei LJ. The Robust Inference for the Cox Proportional Hazards Model. Journal of the American Statistical Association. 1989 Dec;84(408):1074-8.

6. Crowe BJ, Xia HA, Berlin JA, Watson DJ, Shi H, Lin SL, et al. Recommendations for safety planning, data collection, evaluation and reporting during drug, biologic and vaccine development: a report of the safety planning, evaluation, and reporting team. Clin Trials. 2009 Oct;6(5):430-40.

8 LIST OF APPENDICES

Appendix A: Potentially clinically significant abnormalities (PCSA) criteria

Appendix B: List of MedDRA terms for CMQs

Appendix C: Derivation of the efficacy endpoints/components

Appendix D: EQ-5D Patient Questionnaire

Appendix E: EQ-5D utility score algorithm

Appendix F: Detailed statistical methodology for pattern-mixture model

Appendix A Potentially clinically significant abnormalities (PCSA) criteria

CRITERIA for POTENTIALLY CLINICALLY SIGNIFICANT ABNORMALITIES
for phase 2/3 studies (oncology excepted)

Parameter	PCSA	Comments
**Clinical Chemistry**		
ALT	By distribution analysis :<br>>3 ULN<br>>5 ULN<br>>10 ULN<br>>20 ULN	Enzymes activities must be expressed in ULN, not in IU/L.<br>Concept paper on DILI – FDA draft Guidance Oct 2007.<br>Internal DILI WG Oct 2008.<br>Categories are cumulative.<br>First row is mandatory. Rows following one mentioning zero can be deleted.
AST	By distribution analysis :<br>>3 ULN<br>>5 ULN<br>>10 ULN<br>>20 ULN	Enzymes activities must be expressed in ULN, not in IU/L.<br>Concept paper on DILI – FDA draft Guidance Oct 2007.<br>Internal DILI WG Oct 2008.<br>Categories are cumulative.<br>First row is mandatory. Rows following one mentioning zero can be deleted.
Alkaline Phosphatase	>1.5 ULN	Enzymes activities must be expressed in ULN, not in IU/L.<br>Concept paper on DILI – FDA draft Guidance Oct 2007.<br>Internal DILI WG Oct 2008.
Total Bilirubin	>1.5 ULN<br>>2 ULN	Must be expressed in ULN, not in µmol/L or mg/L. Categories are cumulative.<br>Concept paper on DILI – FDA draft Guidance Oct 2007.<br>Internal DILI WG Oct 2008.
Conjugated Bilirubin	>35% Total Bilirubin and TBILI>1.5 ULN	Conjugated bilirubin dosed on a case-by-case basis.
ALT and Total Bilirubin	ALT>3 ULN and TBILI>2 ULN	Concept paper on DILI – FDA draft Guidance Oct 2007.<br>Internal DILI WG Oct 2008.<br>To be counted within a same treatment phase, whatever the interval between measurement.
CPK	>3 ULN<br>>10 ULN	FDA Feb 2005.<br>Am J Cardiol April 2006.<br>Categories are cumulative.<br>First row is mandatory. Rows following one mentioning zero can be deleted.

CRITERIA for POTENTIALLY CLINICALLY SIGNIFICANT ABNORMALITIES
for phase 2/3 studies (oncology excepted)

Parameter	PCSA	Comments
CLcr (mL/min)<br>(Estimated creatinine clearance based on the Cokcroft-Gault equation)	<15 (end stage renal disease)<br>≥15 - <30 (severe decrease in GFR)<br>≥30 - < 60 (moderate decrease in GFR)<br>≥60 - <90 (mild decrease in GFR)<br>≥ 90 (normal GFR)	FDA draft Guidance 2010<br>Pharmacokinetics in patients with impaired renal function-study design, data analysis, and impact on dosing and labeling
eGFR (mL/min/1.73m2)<br>(Estimate of GFR based on an MDRD equation)	<15 (end stage renal disease)<br>≥15 - <30 (severe decrease in GFR)<br>≥30 - < 60 (moderate decrease in GFR)<br>≥60 - <90 (mild decrease in GFR)<br>≥ 90 (normal GFR)	FDA draft Guidance 2010<br>Pharmacokinetics in patients with impaired renal function-study design, data analysis, and impact on dosing and labeling
Creatinine	≥150 µmol/L (Adults)<br>≥30% change from baseline<br>≥100% change from baseline	Benichou C., 1994.
Uric Acid<br>Hyperuricemia<br>Hypouricemia	 >408 µmol/L<br><120 µmol/L	Harrison- Principles of internal Medicine 17th Ed., 2008.
Blood Urea Nitrogen	≥17 mmol/L	
Chloride	<80 mmol/L<br>>115 mmol/L	
Sodium	≤129 mmol/L<br>≥160 mmol/L	
Potassium	<3 mmol/L<br>≥5.5 mmol/L	FDA Feb 2005.
Total Cholesterol	≥7.74 mmol/L	Threshold for therapeutic intervention.
Triglycerides	≥4.6 mmol/L	Threshold for therapeutic intervention.
Lipasemia	≥3 ULN	
Amylasemia	≥3 ULN	
Glucose<br>Hypoglycaemia<br>Hyperglycaemia	 ≤3.9 mmol/L and <LLN<br>≥11.1 mmol/L (unfasted); ≥7 mmol/L (fasted)	 ADA May 2005.<br>ADA Jan 2008.
HbA1c	>8%	
Albumin	≤25 g/L	

CRITERIA for POTENTIALLY CLINICALLY SIGNIFICANT ABNORMALITIES
for phase 2/3 studies (oncology excepted)

Parameter	PCSA	Comments
CRP	>2 ULN or >10 mg/L (if ULN not provided)	FDA Sept 2005.
**Hematology**		
WBC	<3.0 Giga/L (Non-Black); <2.0 Giga/L (Black)<br>≥16.0 Giga/L	Increase in WBC: not relevant.<br>To be interpreted only if no differential count available.
Lymphocytes	>4.0 Giga/L	
Neutrophils	<1.5 Giga/L (Non-Black);<1.0 Giga/L (Black)	International Consensus meeting on drug-induced blood cytopenias, 1991.<br>FDA criteria.
Monocytes	>0.7 Giga/L	
Basophils	>0.1 Giga/L	
Eosinophils	>0.5 Giga/L or >ULN (if ULN≥0.5 Giga/L)	Harrison- Principles of internal Medicine 17th Ed., 2008.
Hemoglobin	≤115 g/L (Male); ≤95 g/L (Female)<br>≥185 g/L (Male); ≥165 g/L (Female)<br>Decrease from Baseline ≥20 g/L	Criteria based upon decrease from baseline are more relevant than based on absolute value. Other categories for decrease from baseline can be used (≥30 g/L, ≥40 g/L, ≥50 g/L).
Hematocrit	≤0.37 v/v (Male) ; ≤0.32 v/v (Female)<br>≥0.55 v/v (Male) ; ≥0.5 v/v (Female)	
RBC	≥6 Tera/L	Unless specifically required for particular drug development, the analysis is redundant with that of Hb.<br>Otherwise, consider FDA criteria.
Platelets	<100 Giga/L<br>≥700 Giga/L	International Consensus meeting on drug-induced blood cytopenias, 1991.
**Urinalysis**		
pH	≤4.6<br>≥8	
**Vital signs**		
HR	≤50 bpm and decrease from baseline ≥20 bpm<br>≥120 bpm and increase from baseline≥20 bpm	To be applied for all positions (including missing) except STANDING.
SBP	≤95 mmHg and decrease from baseline ≥20mmHg<br>≥160 mmHg and increase from baseline ≥20 mmHg	To be applied for all positions (including missing) except STANDING.
DBP	≤45 mmHg and decrease from baseline ≥10 mmHg<br>≥110 mmHg and increase from baseline ≥10 mmHg	To be applied for all positions (including missing) except STANDING.

CRITERIA for POTENTIALLY CLINICALLY SIGNIFICANT ABNORMALITIES
for phase 2/3 studies (oncology excepted)

Parameter	PCSA	Comments
Orthostatic Hypotension		
Orthostatic SDB	≤-20 mmHg	
Orthostatic DBP	≤-10 mmHg	
Weight	≥5% increase from baseline<br>≥5% decrease from baseline	FDA Feb 2007.
**ECG**		Ref.: ICH E14 guidance (2005) and E14 Q&A (2012), and Cardiac Safety Research Consortium White Paper on PR and QRS (Nada et al. Am Heart J. 2013; 165(4) : 489-500)
HR	<50 bpm<br><50 bpm and decrease from baseline ≥20 bpm<br><40 bpm<br><40 bpm and decrease from baseline ≥20 bpm<br><30 bpm<br><30 bpm and decrease from baseline ≥20 bpm	Categories are cumulative
	>90 bpm<br>>90 bpm and increase from baseline ≥20bpm<br>>100 bpm<br>>100 bpm and increase from baseline ≥20bpm<br>>120 bpm<br>>120 bpm and increase from baseline ≥20 bpm	Categories are cumulative
PR	>200 ms<br>>200 ms and increase from baseline ≥25%<br>> 220 ms<br>>220 ms and increase from baseline ≥25%<br>> 240 ms<br>> 240 ms and increase from baseline ≥25%	Categories are cumulative
QRS	>110 ms<br>>110 msec and increase from baseline ≥25%<br>>120 ms<br>>120 ms and increase from baseline ≥25%	Categories are cumulative
QT	>500 ms	

CRITERIA for POTENTIALLY CLINICALLY SIGNIFICANT ABNORMALITIES
for phase 2/3 studies (oncology excepted)

Parameter	PCSA	Comments
QTc	Absolute values (ms)<br><br>>450 ms<br>>480 ms<br>>500 ms<br><br>Increase from baseline<br>Increase from baseline]30-60] ms<br>Increase from baseline >60 ms	To be applied to any kind of QT correction formula.<br>Absolute values categories are cumulative<br><br>QTc >480 ms and ΔQTc>60 ms are the 2 PCSA categories to be identified in individual subjects/patients listings.

Appendix B List of MedDRA terms for CMQs

Table 11 - CMQ “Type 1 or Type 2 diabetes”

MedDRA Term Label	Preferred Term Code
Diabetes mellitus	10012601
Diabetes mellitus inadequate control	10012607
Insulin resistant diabetes	10022491
Diabetes mellitus malnutrition-related	10050197
Diabetes mellitus management	10051599
Insulin-requiring type 2 diabetes mellitus	10053247
Type 1 diabetes mellitus	10067584
Type 2 diabetes mellitus	10067585
Fulminant type 1 diabetes mellitus	10072628

Table 12 - CMQ “Impaired Glucose Control”

MedDRA Term Label	Preferred Term Code
Glucose tolerance impaired	10018429
Insulin resistance	10022489
Insulin resistance syndrome	10022490
Impaired fasting glucose	10056997

Table 13 - Selected PTs from SMQ “Optic nerve disorders” including in the CMQ for neurologic events

MedDRA Term Label	Preferred Term Code
Benign neoplasm of optic nerve	10057424
Optic atrophy	10030910
Optic discs blurred	10030923
Optic nerve disorder	10061322
Optic nerve injury	10030938
Optic nerve neoplasm	10053645
Optic nerve operation	10053272
Optic neuropathy	10061323
Papillitis	10033708
Pseudopapilloedema	10037141

MedDRA Term Label	Preferred Term Code
Subacute myelo-opticoneuropathy	10058009
Toxic optic neuropathy	10044245
Visual evoked potentials abnormal	10047549
Amaurosis fugax	10001903
Blindness	10005169
Blindness unilateral	10005186
Colour blindness acquired	10010051
Colour vision tests abnormal	10010056
Cranial nerve injury	10061094
Delayed myelination	10076456
Fundoscopy abnormal	10017520
Hemianopia	10019452
Hemianopia heteronymous	10019455
Hemianopia homonymous	10019456
Loss of visual contrast sensitivity	10064133
Neuro-ophthalmological test abnormal	10029256
Night blindness	10029404
Ophthalmological examination abnormal	10056836
Optic pathway injury	10030949
Optical coherence tomography abnormal	10073561
Quadranopia	10075427
Visual acuity reduced	10047531
Visual acuity reduced transiently	10047532
Visual acuity tests abnormal	10047534
Visual field defect	10047555
Visual field tests abnormal	10047567
Visual impairment	10047571
Visual pathway disorder	10061411

Table 14 - CMQ “Neurocognitive disorders – FDA’s recommendation”

MedDRA level	MedDRA Code	MedDRA Term Label
PTCD	10001949	Amnesia
PTCD	10061423	Amnestic disorder
PTCD	10002711	Anterograde Amnesia
PTCD	10066842	Behavioural and Psychiatric Symptoms of Dementia
PTCD	10008398	Change in sustained attention
LLTCD	10009843	Cognitive Deterioration
PTCD	10057668	Cognitive Disorder
LLTCD	10010300	Confusion
LLTCD	10048321	Confusion Aggravated
PTCD	10010305	Confusional State
PTCD	10012218	Delirium
PTCD	10012267	Dementia
PTCD	10012271	Dementia Alzheimer's type
LLTCD	10012290	Dementia Nos
LLTCD	10012291	Dementia Nos Aggravated
LLTCD	10012292	Dementia of the Alzheimer's type NOS
PTCD	10067889	Dementia with Lewy Bodies
PTCD	10013395	Disorientation
PTCD	10013496	Disturbance in attention
PTCD	10070246	Executive dysfunction
PTCD	10068968	Frontotemporal Dementia
LLTCD	10058669	Global Amnesia
PTCD	10021402	Illogical Thinking
PTCD	10071176	Impaired reasoning
PTCD	10021630	Incoherent
PTCD	10023236	Judgement impaired
PTCD	10027175	Memory Impairment
PTCD	10027374	Mental Impairment
LLTCD	10027376	Mental Impairment Nos
LLTCD	10048345	Mental State Abnormal Aggravated
PTCD	10048294	Mental Status Changes
PTCD	10065424	Mini Mental Status Examination Abnormal
PTCD	10036631	Presenile Dementia
PTCD	10038965	Retrograde Amnesia
PTCD	10039966	Senile Dementia

MedDRA level	MedDRA Code	MedDRA Term Label
LLTCD	10039967	Senile Dementia Nos
LLTCD	10040602	Short-term Memory Loss
PTCD	10043431	Thinking Abnormal
LLTCD	10043438	Thinking Slowed
PTCD	10044380	Transient Global Amnesia
PTCD	10057678	Vascular Dementia

Appendix C Derivation of the efficacy endpoints/components

The objective of this appendix is to detail the rules to derive the efficacy endpoints/components as per CEC and as per investigator. Rules describe the derivation of the type of the events, but also the date of the events that will be used for analyses using time to event approach.

A) DERIVATION OF CARDIOVASCULAR EVENTS AS PER CEC

1. Event "Non-fatal MI"

"Non-fatal MI" includes non-fatal MI or unstable angina adjudicated as MI (excluding events adjudicated as silent MI).

For patients who died with acute MI as primary cause of death as per adjudication, the last MI (excluding silent MIs) among those confirmed by adjudication and that occurred within 30 days before the death will not be considered as a "non-fatal MI", but as a "fatal MI" included in the "CHD death" category.

The date of event that will be considered in all analyses is the onset date of the MI.

2. Event "Non-fatal ischemic stroke and fatal ischemic stroke"

"Non-fatal ischemic stroke and fatal ischemic stroke" includes strokes adjudicated as ischemic stroke or, as stroke "not otherwise specified".

The date of event that will be considered in the analysis is the onset date of the stroke.

3. Event "Unstable angina requiring hospitalization"

"Unstable angina requiring hospitalization" includes events adjudicated as UA requiring hospitalization.

The date of the event that will be considered in the analyses is the onset date of the UA.

4. Event "CHD Death"

"CHD death" includes deaths adjudicated as due to Coronary Heart Disease and deaths adjudicated with an "Undetermined" primary cause of death.

The date of event that will be considered in the analysis of "Time to CHD death" is the date of death.

In the analysis of the primary efficacy endpoint, fatal MIs will be included in the category "CHD death", therefore the date of the CHD death that will be considered to determine the first event among the events included in the composite is:

- The date of onset of the "fatal MI" (see above in section A.1.) if the primary cause of death is "Acute myocardial infarction"
- The date of death otherwise

The same date for CHD death will be used for the analyses on "Time to any CHD event" and "Time to any major CHD event".

5. Event "Ischemia-driven coronary revascularization procedure"

"Ischemia-driven coronary revascularization procedure" includes PCI and CABG procedures with "Acute coronary syndrome" or "New-progressive symptoms of chronic ischemia or new progressive functional test abnormality that occurred since randomization" as reason for revascularization AND "De-novo lesion" and/or "Stent thrombosis" as type of lesions that required revascularization (ie, procedures performed only to treat restenosis lesion(s) are excluded from the endpoint).

The date of the event that will be considered in the analyses is the date of the revascularization procedure.

6. Event "CV Death"

"CV death" includes deaths with primary cause of death adjudicated as "Cardiovascular" or "undetermined".

The date of event that will be considered in the analysis of "Time to CV death" is the date of death.

In the analysis of "Time to any CV event" (any non-fatal CHD event, any CV death, or non-fatal ischemic stroke), fatal MIs and fatal strokes will be included in the category "CV death", therefore the date of the CV death that will be considered to determine the first event among the events included in the composite is:

- The date of onset of the "fatal MI" (see above in section A.1.) if the primary cause of death is "Acute myocardial infarction"
- The date of onset of the fatal ischemic stroke if the primary cause of death is "Stroke – ischemic" or "Stroke – Undetermined". The fatal ischemic stroke will be the last stroke among those confirmed by adjudication that occurred within 30 days before the death
- The date of death for other causes of death

7. Event “Congestive heart failure requiring hospitalization”

“CHF requiring hospitalization” includes events confirmed by adjudication.

The date of the event that will be considered in the analyses will be the onset date of the CHF event.

8. Event “All-cause mortality”

“All-cause mortality” includes all adjudicated deaths.

The date of event that will be considered in the analysis of “Time from randomization to death (all-cause mortality)” is the date of death.

In the analysis of “Time to all-cause mortality, non-fatal MI, non-fatal ischemic stroke”, fatal MIs and fatal strokes will be included in the category “all-cause mortality”, therefore the date of all-cause mortality that will be considered to determine the first event among the events included in the composite is:

- The date of onset of the “fatal MI” (see above in section A.1.) if the primary cause of death is “Acute myocardial infarction”
- The date of onset of the fatal ischemic stroke (see above in section A.2.) if the primary cause of death is “Stroke – ischemic” or “Stroke – Undetermined”
- The date of death for other causes of death

B) DERIVATION OF CARDIOVASCULAR EVENTS AS PER INVESTIGATOR

Endpoints as per investigator’s opinion will be derived for the sensitivity analysis of the primary efficacy endpoint.

1. Event “Non-fatal MI”

“Non-fatal MI” includes all events reported, with a final diagnosis as per investigator of “Spontaneous, non-procedural MI”, “peri-PCI MI”, or “peri-CABG MI.

For patients who died with acute MI as primary cause of death as per investigator, the last MI reported and that occurred within 30 days before the death will not be considered as a “non-fatal MI”, but is a “fatal MI” included in the “CHD death” category.

The date of event that will be considered in the analysis is the date of onset of ischemic symptoms that caused the subject to seek medical attention, reported by the investigator in the e-CRF specific form (could be slightly different from the date as per CEC).

2. Event "Non-fatal ischemic stroke and Fatal ischemic stroke"

"Non-fatal ischemic stroke and fatal ischemic stroke" includes all cerebrovascular events reported, with a final diagnosis as per investigator of "ischemic stroke", "ischemic stroke with hemorrhagic conversion", or "undetermined stroke".

The date of event that will be considered in the analysis is the date of new or worsening neurological symptoms, reported by the investigator in the e-CRF specific form (could be slightly different from the date as per CEC).

3. Event "Unstable angina requiring hospitalization"

The definition of "Unstable angina requiring hospitalization" in the protocol of the Outcomes study is more restrictive than the unstable angina the investigators had to report in the e-CRF to allow all potential UA to be adjudicated. Therefore a subset of all the UA reported by the investigator will be selected for the analysis, as follows:

"Unstable angina requiring hospitalization" includes all events reported by the investigator, with a final diagnosis of "unstable angina", and in addition with:

- The presence of an hospitalization (with discharge not before the next calendar day) that may correspond to the event, and;
- New ECG findings (Persistent ST elevation, ST depression, T wave inversion, New LBBB, Q waves, or Other findings), and;
- Contemporary evidence of angiographically significant coronary disease (PCI/CABG performed to treat at least one significant coronary lesion, or Diagnostic catheterization with at least one lesion >70% that is not a restenosis at previous PCI site)

In the absence of those criteria, since an elevation of cardiac biomarkers observed within 48H of the event may be the sign of a possible MI (STEMI or non STEMI), the event will be also considered as a possible composite from the primary endpoint.

The date of the event that will be considered in the analysis is the date of onset of ischemic symptoms that caused the subject to seek medical attention, reported by the investigator in the e-CRF specific form (could be slightly different from the date as per CEC).

4. Event "CHD Death"

Since the categorization of deaths as CHD death was not requested to the Investigator, we will apply the convention below for "CHD death" as per investigator's opinion:

"CHD deaths" as per investigator include deaths due to "Acute myocardial infarction", "Sudden cardiac death", "Heart failure or cardiogenic shock", and deaths with an "Undetermined" primary cause of death.

In the sensitive analysis of the primary efficacy endpoint as per investigator, fatal MIs will be included in the category "CHD death", therefore the date of the CHD death that will be considered to determine the first event among the events included in the composite is:

- The date of onset of ischemic symptoms that caused the subject to seek medical attention of the "fatal MI" (see above in section B.1.) if the primary cause of death as per investigator is "Acute myocardial infarction"
- The date of death otherwise.

Appendix D EQ-5D Patient Questionnaire

[redacted]

2

© 1998 EuroQol Group EQ-5D™ is a trade mark of the EuroQol Group

Appendix E EQ-5D utility score algorithm

[redacted]

Appendix F Detailed statistical methodology for pattern-mixture model

A pattern-mixture model approach will be used for change in LDL_C at each timepoint, with a different imputation strategy applied for missing LDL-C values during the treatment period (ie, within the time period from the first double-blind investigational medicinal product [IMP] injection up to the day of the last double-blind injection +21 days) and missing LDL C values after treatment discontinuation (ie, after the day of last double-blind injection +21 days) based on the following assumptions:

- Patients within 21 days of their last IMP injection would continue to show benefit from treatment similar to that observed at the scheduled time point. Therefore, LDL C values missing during the treatment period will be considered "Missing At Random" and imputed using a model estimated using all samples collected on treatment.
- Patients who stopped taking their study treatment no longer benefited from it in the future, and thus tended to have LDL-C values returning to baseline. Thus LDL-C values missing after treatment discontinuation will be imputed based on patient's own baseline value.

The assumptions for this approach were based on the following considerations:

- Missing values during the treatment period are mostly consecutive to:
  - Visits performed outside of the pre-specified time-window;
  - No blood sample available although visit was done;
  - LDL-C not measurable due to technical reasons.

  In addition, these missing data were often intermittent, ie, followed by LDL-C values collected at subsequent visits. It was therefore considered reasonable to assume that these missing data were "At Random".
- Phase 2 studies DFI11565 and R727-CL-1003 included a prospective assessment of calculated LDL-C during the follow-up period after a 12-week treatment period. These studies showed that after treatment discontinuation, the average calculated LDL-C returned to baseline level within 4 weeks after ceasing alirocumab treatment (see Figure 1 and Figure 2).

Figure 1 - Study DFI11565: calculated LDL-C mean (+/- SE) percent change from baseline

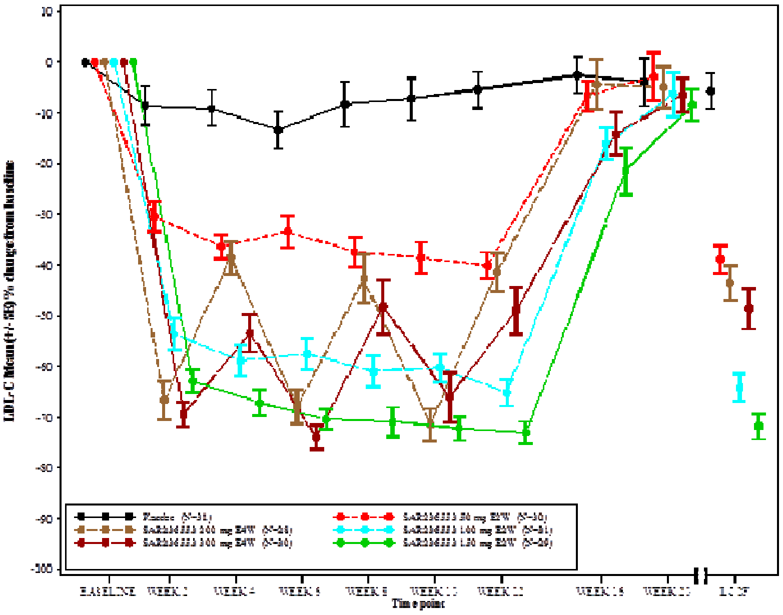

Figure 2 - Study R-727-CL-1003: calculated LDL-C mean (+/- SE) percent change from baseline

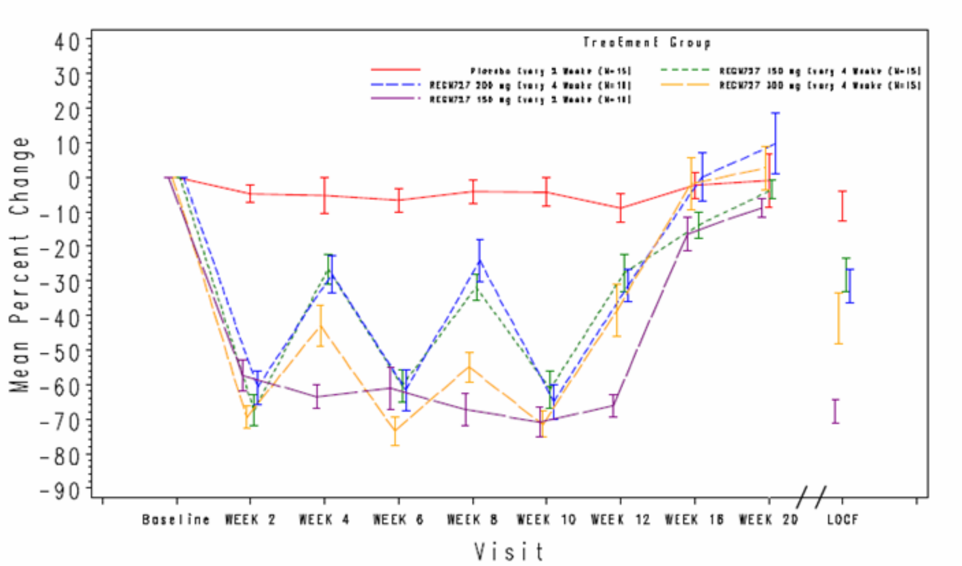

Missing LDL-C values will be imputed 10 times to generate 10 complete data sets. For each planned time point, the percent changes from baseline will be derived from observed and imputed LDL-C at this time point. The completed data sets will be analyzed using an ANCOVA model with treatment group as fixed effect, and the baseline LDL-C value as continuous covariate. The results from the 10 analyses will be combined using Rubin's formulae. If necessary, the number of imputations (10) will be increased until stable estimates are obtained.

Imputation of missing data during the treatment period

Missing LDL-C values during the treatment period will be imputed from other on-treatment measurements assuming Missing At Random, using SAS® MI procedure.

Only LDL-C values collected during the treatment period will be included in the imputation model. This way, missing LDL-C values during the treatment period will be imputed based solely on observed on-treatment LDL-C values.

The imputation model will be estimated within each treatment arm and include baseline LDL-C value, and all LDL-C values at pre-specified visits. Since the pattern of missing data will necessarily be non-monotone, a Monte-Carlo Markov Chain (MCMC) method will be used. A minimum value of 0 will be specified in order to avoid negative imputed LDL-C values.

A sample SAS code is provided below:

```
proc mi data=DATAIN out=DATAOUT nimpute=10 minimum=0;
  var LDL_BASE LDL_M1 LDL_M2 LDL_M4 LDL_M8 …;
by ARM;
run;
```

As stated above, the input dataset DATAIN will include only LDL-C values collected during the treatment period. Any LDL-C values collected during the post-treatment period will be excluded from the input dataset. In practice, the MI procedure will generate imputed values for all missing values (whether on-treatment or post-treatment), but only imputed values during the treatment period will be kept in the final datasets that will be analyzed. Imputed values during the post-treatment period will be discarded and replaced by imputed values described in the next paragraph.

Imputation of missing data after treatment discontinuation

Missing LDL-C values during the post-treatment period will be imputed assuming LDL C values would on average return to baseline values.

For each patient, missing post-treatment LDL-C values will be imputed 10 times, using a random draw from a normal distribution, with mean equal to patient's own baseline value and variance equal to the conditional variance at the specific time-point, given the baseline value.

Let Y_0 and Y_1 denote the LDL-C at baseline and at the specific time-point respectively. Since Y_0 and Y_1 are assumed to have a bivariate normal distribution, the conditional variance of Y_1 given Y_0 is:

$$Var(Y_1 | Y_0 = y_0) = \sigma_1^2 (1 - \rho^2)$$

Where σ_1^2 denotes the variance of Y1 and ρ the coefficient of correlation between Y_0 and Y_1.

The conditional variance will be estimated from observed data within the same treatment arm at the specific time-point.

During the random generation process, a minimum value of 0 will also be applied in order to avoid negative imputed LDL-C values.

The same methodology will be applied to all lipid parameters, using log-transformation of variables for TGs and Lp (a).

EFC11570 16.1.9 Statistical analysis plan

ELECTRONIC SIGNATURES

Signed by	Meaning of Signature	Server Date (dd-MMM-yyyy HH:mm)
[redacted]		
