# Supplementary material for: Effect of alirocumab on cataracts in patients with acute coronary syndromes
Source: BMC Ophthalmol. 2023 Jun 16;23:279. doi: 10.1186/s12886-023-03012-1 (PMC10276414; doi:10.1186/s12886-023-03012-1)
Supplement: Supplementary file 2 — Additional file 2. [file 12886_2023_3012_MOESM2_ESM.docx]

**Effect of alirocumab on cataracts in patients with acute coronary syndromes**

Contents

[SUPPLEMENTARY METHODS 2](#_Toc137455020)

[STUDY ENROLMENT CRITERIA 2](#_Toc137455021)

[STUDY TREATMENTS 2](#_Toc137455022)

[Run-in period and adjustment of background lipid-modifying therapy 2](#_Toc137455023)

[Patients from china 3](#_Toc137455024)

[Main secondary efficacy end points 3](#_Toc137455025)

[Other secondary efficacy end points 3](#_Toc137455026)

[Patients with blinded adjustment in alirocumab dose 3](#_Toc137455027)

[Sample size estimation and statistical methods for primary analysis 4](#_Toc137455028)

[Appendix Table 1 Participating countries and definitions of geographic regions^2^ 5](#_Toc137455029)

[ODYSSEY OUTCOMES committees and investigators 6](#_Toc137455030)

[Institutional Review Board (IRB)/Independent Ethics Committee (IEC) list 28](#_Toc137455031)

[References 142](#_Toc137455032)

# SUPPLEMENTARY METHODS

The following information on the ODYSSEY OUTCOMES study design and methods and the primary results has been published elsewhere.^1,2^ The protocol is available at https://www.nejm.org/.

## STUDY ENROLMENT CRITERIA

Inclusion criteria

- Hospitalization for acute coronary syndrome, defined by symptoms of myocardial ischemia with an unstable pattern, occurring at rest or with minimal exertion, within 72 hours of an unscheduled hospital admission due to presumed or proven obstructive coronary disease and at least one of the following:
- Elevated cardiac biomarkers
- Resting electrocardiographic changes consistent with ischemia or infarction, plus additional evidence of obstructive coronary disease from regional wall motion or perfusion abnormality, 70% or more epicardial coronary stenosis by angiography, or need for coronary revascularization procedure.
- Lipid levels inadequately controlled by atorvastatin 40 to 80 mg, rosuvastatin 20 to 40 mg daily, or the maximum tolerated dose of one of these agents, defined by at least one of the following:
  - Low-density lipoprotein (LDL) cholesterol 70 mg per deciliter (1.81 mmol per liter) or more
  - Non−high-density lipoprotein (non–HDL) cholesterol 100 mg per deciliter (2.59 mmol per liter) or more
  - Apolipoprotein B 80 mg per deciliter or more.

Principal exclusion criteria

- Age less than 40 years
- Qualifying index ACS event less than 4 weeks or more than 52 weeks before randomization
- Not on stable lipid-modifying therapy for at least 2 weeks before randomization
- Uncontrolled hypertension (greater than 180 mm Hg systolic and/or greater than 110 mm Hg diastolic at randomization visit)
- New York Heart Association class III or IV congestive heart failure persisting despite treatment or left ventricular ejection fraction less than 25% if measured
- History of hemorrhagic stroke
- Fasting triglycerides greater than 400 mg per deciliter (4.52 mmol per liter) at qualifying laboratory visit
- Recurrent acute coronary syndrome event within 2 weeks prior to randomization visit
- Coronary revascularization procedure performed within 2 weeks prior to randomization visit or planned after randomization
- Liver transaminases more than three times upper limit of normal; laboratory evidence of current hepatitis B or C infection; creatine kinase more than three times upper limit of normal; estimated glomerular filtration rate less than 30 ml/(min⋅1.73 m^2^); positive urine or serum pregnancy test
- Use of fibrates other than fenofibrate or fenofibric acid.

## STUDY TREATMENTS

## Run-in period and adjustment of background lipid-modifying therapy

The protocol specified a run-in period of 2−16 weeks. During this period, patients were trained in the use of the autoinjector to administer study medication and intensive or maximum tolerated atorvastatin or rosuvastatin treatment was initiated, continued, or adjusted up to 2 weeks prior to the qualifying visit. If treatment with high-dose atorvastatin or rosuvastatin was not tolerated due to side effects or laboratory abnormalities, lower doses of atorvastatin (10 to 20 mg daily) or rosuvastatin (5 to 10 mg daily) were utilized, or the patient was switched from atorvastatin to rosuvastatin or vice versa. In the absence of tolerability issues, low or moderate doses of atorvastatin or rosuvastatin could be used for valid medical reasons documented on case report forms, including advanced age, low body mass, or interaction of statin with another required medication. Statin-intolerance was defined in the protocol as intolerance to at least two statins. A determination of statin intolerance required investigator review of the medical history, discussion with patient, family, and/or treating physician, and documentation on case report forms. Patients with documented statin intolerance could be randomized without any background statin therapy. Treatment with non-statin lipid modifying agents was permissible, with or without concurrent statin therapy, except that fibrates other than fenofibrate or fenofibric acid were prohibited.

The run-in period concluded with a qualifying visit with laboratory testing, performed after a minimum of 2 weeks on stable, tolerated background lipid-modifying therapy with no intent to make further changes to lipid-modifying therapy. Patients who met lipid and other inclusion criteria at the qualifying visit could proceed to randomization if no exclusion criteria were fulfilled. After randomization, if a statin safety or tolerability concern arose the protocol allowed investigators to decrease the dose of atorvastatin or rosuvastatin, switch from atorvastatin to rosuvastatin or vice versa, prescribe a statin other than atorvastatin or rosuvastatin, or prescribe a non-statin lipid-modifying agent. Changes to lipid-modifying therapy after randomization were recorded on a case report form.

## Patients from china

To comply with a request from Chinese regulatory authorities, a minimum of 600 patients were included in the trial from sites in the People’s Republic of China. Randomization of the main study cohort occurred between November 2012 and November 2015. Because of the time required to complete administrative procedures, patients from China were randomized after the main cohort, from May 2016 to February 2017.

## Main secondary efficacy end points

The main secondary efficacy end points were tested sequentially, using the order defined below. The hierarchy was revised during the course of the trial,* and was prespecified in the final statistical analysis plan, available at www.nejm.org.

- Time from randomization to first occurrence of any coronary heart disease event (major coronary heart disease event, unstable angina requiring hospitalization, ischemia-driven coronary revascularization procedure);
- Time from randomization to first occurrence of any major coronary heart disease event (coronary heart disease death, nonfatal myocardial infarction);
- Time from randomization to first occurrence of any cardiovascular event (any nonfatal coronary heart disease event, any cardiovascular death, and nonfatal ischemic stroke);
- Time from randomization to first occurrence of all-cause mortality, nonfatal myocardial infarction, nonfatal ischemic stroke;
- Time from randomization to coronary heart disease death;
- Time from randomization to cardiovascular death;
- Time from randomization to death (all-cause mortality).

* In the initial statistical analysis plan for the study, the pre-specified testing hierarchy for secondary endpoints placed all-cause mortality directly after the composite endpoints of any coronary heart disease event, major coronary heart disease event, any cardiovascular event, and all-cause death, nonfatal myocardial infarction, or nonfatal ischemic stroke; cause-specific death did not appear in the hierarchy.^1^ In a subsequent amendment to the analysis plan that was implemented prior to unblinding of the study database, coronary heart disease death and cardiovascular death were placed ahead of all-cause death in the testing hierarchy, as reflected in the list of main secondary efficacy end points above.

##

## Other secondary efficacy end points

- Components of the primary end point considered individually:
- Time from randomization to first occurrence of any nonfatal myocardial infarction;
- Time from randomization to first occurrence of fatal or any nonfatal ischemic stroke;
- Time from randomization to first occurrence of any unstable angina requiring hospitalization.
- Time from randomization to first occurrence of any ischemia-driven coronary revascularization procedure;
- Time from randomization to first occurrence of any congestive heart failure requiring hospitalization.

## Patients with blinded adjustment in alirocumab dose

The ODYSSEY OUTCOMES trial was designed to test the efficacy and safety of alirocumab by targeting achieved LDL cholesterol levels of 25 to 50 mg per deciliter (0.65 to 1.29 mmol per liter), allowing levels of 15 to 25 mg per deciliter (0.39 to 0.65 mmol per liter), but avoiding sustained levels below 15 mg per deciliter. Protocol-specified, blinded dose adjustment algorithms were used for this purpose.

Results of the application of these algorithms are as follows: At first assessment following randomization, 2615 (27.6%) patients treated with alirocumab had an LDL cholesterol level of 50 mg per deciliter or higher and subsequently underwent blinded up titration of alirocumab from 75 mg to 150 mg. Of these 2615 patients, 805 later underwent blinded down titration back to 75 mg after two consecutive LDL cholesterol measurements below 25 mg per deciliter. In the alirocumab group, 730 (7.7%) patients were blindly switched to placebo at a median 8.3 months from randomization after two consecutive LDL cholesterol measurements below 15 mg per deciliter while on the 75 mg dose. These patients, whose median baseline LDL cholesterol was 71 (IQR, 58 to 83) mg per deciliter, were included in the alirocumab group in intention-to-treat analyses. Of the total time on treatment with alirocumab, 77.8% and 22.2% were at the 75 mg and 150 mg doses, respectively.

## Sample size estimation and statistical methods for primary analysis

Efficacy was determined by time to first occurrence of any component of the primary end point by intention-to-treat, including all patients and events from randomization to the common trial end date. Design assumptions included a primary end point incidence of 11.4% at 4 years in the placebo group and median baseline LDL cholesterol of 90 mg per deciliter, with an anticipated reduction of 50% with alirocumab treatment, resulting in an expected 15% hazard reduction. It was estimated that 1613 primary end point events occurring in 18,000 patients over a median follow-up of approximately 3 years would provide 90% power to detect the expected hazard reduction at a 5% significance level. In China, 614 patients were randomized after the main trial cohort (as discussed in the Supplementary Appendix). The protocol specified that the trial continue until at least 1613 primary end point events occurred and all evaluable patients were followed for at least 2 years (except those from China), assuring a minimum observation time to assess safety and efficacy. Treatment hazard ratios and 95% confidence intervals (CIs) were estimated by Cox proportional hazards models, stratified by geographic region; P values were determined by stratified log rank tests. To control for multiplicity, if the primary end point hazard was significantly lower in the alirocumab group, main secondary end points were tested in hierarchical fashion in the sequence listed above. Two prespecified interim analyses were performed when approximately 50% and 75% of the planned primary end point events had occurred; neither led to early stopping of the trial. To account for the two interim analyses, a two-sided P value less than 0.0498 was required for statistical significance of the primary end point at the final analysis. Absolute treatment effects in subgroups were compared with the Gail−Simon test.^3^ The Statistical Analysis Plan and Supplementary Appendix contain additional descriptive safety analyses and analytic methods.

# Appendix Table 1 Participating countries and definitions of geographic regions^2^

| Canada/USA | Latin America | Western Europe | Central and Eastern Europe | East/South Asia | Rest of the World |
| --- | --- | --- | --- | --- | --- |
| Canada  United States | Argentina  Brazil  Chile  Colombia  Guatemala  Mexico  Peru | Austria  Belgium  Denmark  Finland  France  Germany  Greece  Italy  Netherlands  Norway  Portugal  Spain  Sweden  Switzerland  United Kingdom | Bosnia– Herzegovina  Bulgaria  Croatia  Czech Republic  Estonia  Georgia  Hungary  Latvia  Lithuania  Macedonia  Poland  Romania  Russian Federation  Serbia  Slovakia  Slovenia  Turkey  Ukraine | China  Hong Kong  India  Japan  Korea  Malaysia  Philippines  Singapore  Sri Lanka  Thailand  Taiwan | Australia  Israel  New Zealand  Republic of South Africa |

# ODYSSEY OUTCOMES committees and investigators

**Executive Steering Committee**

**Co-Chairs: Gregory G. Schwartz** (Division of Cardiology, University of Colorado School of Medicine, Aurora, CO, USA) **and Ph. Gabriel Steg** (Université de Paris, Assistance Publique-Hôpitaux de Paris, Hôpital Bichat, Paris, and INSERM-1148, Paris, France; National Heart and Lung Institute, Imperial College, Royal Brompton Hospital, London, UK)

Deepak L. Bhatt (Mount Sinai Heart, Icahn School of Medicine at Mount Sinai Health System, New York, NY, USA; previously at Brigham and Women's Hospital Heart & Vascular Center and Harvard Medical School, Boston, MA, USA), Vera A. Bittner (University of Alabama at Birmingham, Birmingham, AL, USA), Rafael Diaz (Estudios Cardiológicos Latinoamérica, Instituto Cardiovascular de Rosario, Rosario, Argentina), Shaun G. Goodman (Canadian VIGOUR Centre, University of Alberta, Edmonton, Alberta, Canada, and St. Michael’s Hospital, University of Toronto, Toronto, Ontario, Canada), Robert A. Harrington (Stanford Center for Clinical Research, Department of Medicine, Stanford University, Stanford, CA, USA), J. Wouter Jukema (Leiden University Medical Center, Leiden, The Netherlands), Michael Szarek (State University of New York, Downstate School of Public Health, Brooklyn, NY, USA), Harvey D. White (Green Lane Cardiovascular Research Unit, Te Whatu Ora – Health New Zealand, Te Toka Tumai, and University of Auckland, Auckland, New Zealand), Andreas M. Zeiher (Department of Medicine III, Goethe University, Frankfurt am Main, Germany).

***Nonvoting members:***

*Ex officio:* Pierluigi Tricoci (Duke Clinical Research Institute, Duke University Medical Center, Durham, NC, USA), Matthew T. Roe (Duke Clinical Research Institute, Duke University Medical Center, Durham, NC, USA), Kenneth W. Mahaffey (Stanford Center for Clinical Research, Department of Medicine, Stanford University, Stanford, CA).

*Sponsor representatives*: Jay M. Edelberg (Sanofi), Corinne Hanotin (Sanofi), Guillaume Lecorps (Sanofi), Angèle Moryusef (Sanofi), Robert Pordy (Regeneron Pharmacauticals), William J. Sasiela (Regeneron Pharmacauticals), Jean-François Tamby (Sanofi).

**National Leaders**

**Argentina**: Rafael Diaz (Estudios Cardiológicos Latinoamérica, Rosario); **Australia**: Philip E Aylward (South Australian Health and Medical Research Institute, Flinders University and Medical Centre, Adelaide); **Austria**: Heinz Drexel (Landeskrankenhaus Feldkirch, Feldkirch); **Belgium**: Peter Sinnaeve (UZ Leuven, Leuven); **Bosnia and Herzegovina**: Mirza Dilic (University Clinical Center Sarajevo, Clinic of Cardiovascular Diseases and Rheumatism, Sarajevo, Bosnia and Herzegovina); **Brazil**: Renato D. Lopes (Duke University Medical Center, Duke Clinical Research Institute, Durham, NC); **Bulgaria**: Nina N Gotcheva (MHAT “National Cardiology Hospital” EAD, Sofia); **Canada**: Shaun G. Goodman (Canadian VIGOUR Centre, University of Alberta, Edmonton); **Chile**: Juan-Carlos Prieto (Hospital Clinico Universidad De Chile, Santiago); **China**: Huo Yong (Peking University First Hospital, Beijing); **Colombia**: Patricio López-Jaramillo (Masira Research Institute, Medical School (UDES), Bucaramanga); **Croatia**: Ivan Pećin (University of Zagreb, Zagreb school of medicine, University hospital center Zagreb; Zagreb); Zeljko Reiner (University Hospital Center Zagreb, School of Medicine, University of Zagreb, Zagreb); **Czech Republic**: Petr Ostadal (Na Homolce Hospital, Prague); **Denmark**: Steen Hvitfeldt Poulsen (Aarhus Universitetshospital Skejby, Aarhus N); **Estonia**: Margus Viigimaa (North Estonia Medical Centre, Tallinn); **Finland**: Markku S Nieminen (Division of Cardiology, Heart and Lung Center, HUCH); **France**: Nicolas Danchin (Hôpital Européen Georges Pompidou, FACT, an F-CRIN network, Paris); **Georgia**: Vakhtang Chumburidze (Chapidze Emergency Cardiology Center, Tbilisi); **Germany**: Nikolaus Marx (Universitätsklinikum Aachen, Aachen); **Greece**: Evangelos Liberopoulos (School of Medicine, National and Kapodistrian University of Athens, Athens, Greece). **Guatemala**: Pablo Carlos Montenegro Valdovinos (Clínica privada, Unidad de Diagnóstico Cardiológico, Guatemala City); **Hong Kong**: Hung-Fat Tse (Queen Mary Hospital, The University of Hong Kong, Hong Kong); **Hungary**: Robert Gabor Kiss (Military Hospital, Budapest); **India**: Denis Xavier (St John's Medical College, Bangalore); **Israel**: Doron Zahger (Soroka University Medical Center, Faculty of Health Sciences, Ben Gurion University of the Negev, Beer Sheva); **Italy**: Marco Valgimigli (Cardiocentro Ticino Institute, Ente Ospedaliero Cantonale, Lugano, Switzerland; and University of Bern, Bern, Switzerland); **Japan**: Takeshi Kimura (Kyoto University Graduate School of Medicine, Kyoto-shi, Kyoto); **Korea**: Hyo Soo Kim (Seoul National University Hospital, Seoul, Republic of Korea); **Sang-Hyun Kim** (SMG Seoul National University Boramae Medical Center, Seoul); **Latvia**: Andrejs Erglis (Pauls Stradins Clinical University Hospital, University of Latvia, Riga); **Lithuania**: Aleksandras Laucevicius (Vilnius University Hospital Santaros klinikos, Vilnius). **Macedonia**: Sasko Kedev (University Clinic of Cardiology, Skopje); **Malaysia**: Khalid Yusoff (Universiti Teknologi MARA (UiTM) Selayang Campus, Batu Caves and UCSI University); **Mexico**: Gabriel Arturo Ramos López (Medical Office, Guadalajara, Jalisco); **Netherlands**: Marco Alings (Amphia Ziekenhuis Molengracht, Breda); **New Zealand**: Harvey D. White (Green Lane Cardiovascular Research Unit, Te Whatu Ora – Health New Zealand, Te Toka Tumai, and University of Auckland, Auckland, New Zealand); **Norway**: Sigrun Halvorsen (Oslo Universitetssykehus HF, Oslo); **Peru**: Roger M Correa Flores (Hospital Nacional IV Alberto Sabogal Sologuren, Callao); **Philippines**: Rody G. Sy (Cardinal Santos Medical Center, San Juan); **Poland**: Andrzej Budaj (Centre of Postgraduate Medical Education, Grochowski Hospital, Warsaw, Poland); **Portugal**: Joao Morais (Leiria Hospital Center, Leiria, Portugal; ciTechCare, Polytechnic of Leiria, Leiria, Portugal); **Romania**: Maria Dorobantu (Clinical Emergency Hospital of Bucharest, Bucharest); **Russian Federation**: Yuri Karpov (National Medical Research Centre of Cardiology); **Serbia**: Arsen D. Ristic (Department of Cardiology of the Clinical Center of Serbia, Belgrade University School of Medicine, Belgrade); **Singapore**: Terrance Chua (National Heart Centre, Singapore); **Slovakia**: Jan Murin (I. Interna klinika, Univerzitna nemocnica Bratislava, Bratislava); **Slovenia**: Zlatko Fras (Preventive Cardiology Unit, Department of Vascular Medicine, Division of Medicine, University Medical Centre Ljubljana, Ljubljana; Faculty of Medicine, University of Ljubljana, Ljubljana); **Republic of South Africa:** Anthony J Dalby (Life Fourways Hospital, Randburg [previously at Milpark Hospital, Johannesburg]); **Spain**: José Tuñón (Fundación Jiménez Díaz, Madrid); **Sri Lanka**: H. Asita de Silva (Clinical Trials Unit, Department of Pharmacology, Faculty of Medicine, University of Kelaniya); **Sweden**: Emil Hagström (Department of Medical Sciences, and Uppsala Clinical Research Centre, Uppsala University, Uppsala); **Switzerland**: Ulf Landmesser (Universitätsspital Zürich) and Christian Müller (Cardiovascular Research Institute Basel (CRIB) and Department of Cardiology, University Hospital Basel, University of Basel, Basel); **Taiwan**: Chern-En Chiang (General Clinical Research Center, Taipei Veterans General Hospital and National Yang-Ming University, Taipei); **Thailand**: Piyamitr Sritara (Ramathibodi Hospital, Bangkok); **Turkey**: Sema Guneri (Dokuz Eylul Universitesi Tip Fakultesi, Izmir); **Ukraine**: Alexander Parkhomenko (Institute of Cardiology, Kyiv, Ukraine); **United Kingdom**: Kausik K. Ray (Imperial College London, London); **United States**: Patrick M. Moriarty (Clinical Pharmacology – University of Kansas Medical Center, Kansas City, Kansas), Matthew T. Roe (Duke Clinical Research Institute, Durham, NC), Robert Vogel (University of Colorado Denver, Denver, Colorado).

**Data Safety Monitoring Board**

Bernard Chaitman (Chair) (Saint Louis University Center for Health Outcomes Research, Saint Louis University, St. Louis, MO, USA), Sheryl F. Kelsey (Graduate School of Public Health, University of Pittsburgh, PA), Anders G. Olsson (Department of Medicine and Health, Linköping University, Linköping, Sweden), Jean-Lucien Rouleau (Institut de Cardiologie, Université de Montréal, Montréal, Quebec, Canada), Maarten L. Simoons (Erasmus MC, Rotterdam, The Netherlands).

**Monitoring of Safety in Patients with Low Low-Density Lipoprotein Values**

Karen Alexander (Duke University, Duke Clinical Research Institute, Durham, NC, USA), Chiara Meloni (Duke Clinical Research Institute, Duke University Medical Center, Durham, NC, USA), Robert Rosenson (Mt. Sinai School of Medicine, New York, NY, USA), Eric J.G. Sijbrands (Erasmus MC, Rotterdam, The Netherlands)

**Clinical Events Committee**

Pierluigi Tricoci (Chair) (Duke University, Durham, NC, USA), John H. Alexander (Duke University, Durham, NC, USA), Luciana Armaganijan (Brazilian Clinical Research Institute, São Paulo, Brazil), Akshay Bagai (St. Michael’s Hospital, University of Toronto, Toronto, Canada), Maria Cecilia Bahit (Brazilian Clinical Research Institute, São Paulo, Brazil), J. Matthew Brennan (Duke University, Durham, NC, USA), Shaun Clifton (Duke University, Durham, NC, USA), Adam D. DeVore (Duke University, Durham, NC, USA), Shalonda Deloatch (Duke University, Durham, NC, USA), Sheila Dickey (Duke University, Durham, NC, USA), Keith Dombrowski (Duke University, Durham, NC, USA), Grégory Ducrocq (Hôpital Bichat, Assistance Publique – Hôpitaux de Paris, Paris, France), Zubin Eapen (Duke University, Durham, NC, USA), Patricia Endsley (Duke University, Durham, NC, USA), Arleen Eppinger (Duke University, Durham, NC, USA), Robert W. Harrison (Duke University, Durham, NC, USA), Connie Ng Hess (University of Colorado Denver, Denver, CO, USA), Mark A. Hlatky (Stanford University, Stanford, CA, USA), Joseph Dedrick Jordan (University of North Carolina, NC, USA), Joshua W. Knowles (Stanford University, Stanford, CA, USA), Bradley J. Kolls (Duke University, Durham, NC, USA), David F. Kong (Duke University, Durham, NC, USA), Sergio Leonardi (Fondazione IRCCS Policlinico S. Matteo, Pavia, Italy), Linda Lillis (Duke University, Durham, NC, USA), Renato D. Lopes (Duke University, Durham, NC, USA), David J. Maron (Stanford University, Stanford, CA, USA), Kenneth W. Mahaffey (Stanford University, Stanford, CA, USA), Jill Marcus (Duke University, Durham, NC, USA), Robin Mathews (Duke University, Durham, NC, USA), Rajendra H. Mehta (Duke University, Durham, NC, USA), Robert J. Mentz (Duke University, Durham, NC, USA), Humberto Graner Moreira (Brazilian Clinical Research Institute, São Paulo, Brazil), Chetan B. Patel (Duke University, Durham, NC, USA), Sabrina Bernardez Pereira (Brazilian Clinical Research Institute, São Paulo, Brazil), Lynn Perkins (Duke University, Durham, NC, USA), Thomas J. Povsic (Duke University, Durham, NC, USA), Etienne Puymirat (European Hospital Georges Pompidou, Paris, France), Matthew T. Roe (Duke University, Durham, NC, USA), William Schuyler Jones (Duke University, Durham, NC, USA), Bimal R. Shah (Duke University, Durham, NC, USA), Matthew W. Sherwood (Duke University, Durham, NC, USA), Kenya Stringfellow (Duke University, Durham, NC, USA), Darin Sujjavanich (Duke University, Durham, NC, USA), Mustafa Toma (St. Paul's Hospital, University of British Columbia, Vancouver, BC, Canada), Charlene Trotter (Duke University, Durham, NC, USA), Sean F.P. van Diepen (Canadian VIGOUR Centre, University of Alberta, Edmonton, Alberta, Canada), Matthew D. Wilson (Duke University, Durham, NC, USA), Andrew Tze-Kay Yan (St. Michael’s Hospital, University of Toronto, Toronto, Canada).

**Investigators who enrolled at least 1 patient (in descending order of number of patients enrolled) (18,927 patients enrolled overall)**

**Argentina (592 patients enrolled)** Lilia B Schiavi (Clinica Del Prado, Cordoba, Córdoba); Marcelo Garrido (Clinica Privada Provincial, Merlo, Buenos Aires Province); Andrés F Alvarisqueta (Centro de Investigaciones Medicas Mar del Plata, Mar del Plata, Buenos Aires Province); Sonia A Sassone (Consultorios Asoc. de Endocrinologia e Inv. Clin. Aplicada, CABA, Buenos Aires); Anselmo P Bordonava (Clinica FUSAVIM Privada, Villa Maria, Córdoba); Alberto E Alves De Lima (Instituto Cardiovascular De Buenos Aires, Ciudad de Buenos Aires, Buenos Aires); Jorge M Schmidberg (Instituto Medico Aguero, Moron, Buenos Aires); Ernesto A Duronto (Fundacion Favaloro para la Docencia e Investigacion Médica, CABA, Buenos Aires); Orlando C Caruso (Hospital Central Mendoza, Mendoza, Mendoza); Leonardo P Novaretto (Sanatorio Santa Rosa, Santa Rosa, La Pampa); Miguel Angel Hominal (Centro de Investigaciones Clinicas del litoral, Santa Fe, Santa Fe); Oscar R Montaña (Dim Clinica Privada, Ramos Mejia, Buenos Aires Province); Alberto Caccavo (Clinica Coronel Suarez, Coronel Suarez, Buenos Aires Province); Oscar A Gomez Vilamajo (Sanatorio San Martin SA, Venado Tuerto, Santa Fe); Alberto J Lorenzatti (Instituto Medico DAMIC, Córdoba, Córdoba); Luis R Cartasegna (Hospital Italiano De La Plata, La Plata, Buenos Aires Province); Gustavo A Paterlini (Investigaciones Clinicas Tucuman, San Miguel De Tucuman, Tucumán); Ignacio J Mackinnon (Instituto De Investigaciones Clínicas, Mar Del Plata, Buenos Aires Province); Guillermo D Caime (Instituto de Diagnostico Cardiovascular La Plata, La Plata, Buenos Aires Province); Marcos Amuchastegui and Oscar Salomone (Hospital Privado Centro Medico De Cordoba, Cordoba, Córdoba); Oscar R Codutti (Cordis Instituto del Corazón, Resistencia, Chaco); Horacio O Jure (Clinica Chutro S.R.L, Cordoba, Córdoba); Julio OE Bono (Sanatorio Allende, Córdoba, Córdoba); Adrian D Hrabar (Instituto de Investigaciones Clinicas Quilmes, Quilmes, Buenos Aires Province); Julio A Vallejos (Instituto De Cardiología De Corrientes, Corrientes, Corrientes); Rodolfo A Ahuad Guerrero (Corporacion Medica San Martin, San Martin, Buenos Aires Province); Federico Novoa (Consultorio Privado, San Isidro, Buenos Aires); Cristian A Patocchi (Hospital Regional Español Bahia Blanca, Bahia Blanca, Buenos Aires Province); Cesar J Zaidman (Centro de Investigacion y Prevencion Cardiovascular, CABA, Buenos Aires); Maria E Giuliano (Instituto Medico de la Fundacion de Estudios Clinicos, Rosario, Santa Fe); Ricardo D Dran (Sanatorio Mariano Pelliza, Olivos, Buenos Aires Province); Marisa L Vico (Instituto de Investigaciones Clinicas de Zarate, Zarate, Buenos Aires Province); Gabriela S Carnero (I.M.E.P. Instituto Médico Elsa Perez, Ciudadela, Buenos Aires Province); Pablo N Guzman (Sanatorio San Francisco, Santiago del Estero, Santiago del Estero); Juan C Medrano Allende (Clínica y Maternidad Suizo Argentina, CABA, Buenos Aires); Daniela F Garcia Brasca (Hospital Italiano, Cordoba, Córdoba); Miguel H Bustamante Labarta (Centro Médico Lebensohn, Junin, Buenos Aires Province); Sebastian Nani (Clinica Olivos, Olivos, Buenos Aires Province); Eduardo DS Blumberg (Medeos, CABA, Buenos Aires); Hugo R Colombo (Clinica Colombo, Cordoba, Córdoba); Alberto Liberman and Victorino Fuentealba (Instituto Privado de Investigaciones Clinicas de Cordoba, Cordoba, Córdoba); Hector L Luciardi (Centro Modelo De Cardiología, San Miguel de Tucuman, Tucumán); Gabriel D Waisman (Hospital Italiano De Buenos Aires, Buenos Aires, Buenos Aires); Mario A Berli (Hospital "provincial" Dr Jose Maria Cullen, Santa Fe, Santa Fe); Ruben O Garcia Duran (Instituto de Investigaciones Clínicas San Nicolás, San Nicolas, Buenos Aires Province); Horacio G Cestari (Sanatorio Guemes, CABA, Buenos Aires); Hugo A Luquez (Centro Medico Luquez, Cordoba, Cordoba); Jorge A Giordano (Clinica Instituto Medico Adrogue, Adrogue, Buenos Aires Province); Silvia S Saavedra (Sanatorio Parque S.A., Salta, Salta); Gerardo Zapata (Instituto Cardiovascular de Rosario, Rosario, Santa Fe); Osvaldo Costamagna (Clinica Parra Centro de Investigaciones Clinicas de Rafaela, Rafaela, Santa Fe); Susana Llois (Hospital Interzonal [General] de Agudos Eva Peron, San Martin, Buenos Aires Province).

**Australia (216 patients enrolled)** Jonathon H Waites (Coffs Harbour Health Campus, Coffs Harbour, New South Wales); Nicholas Collins (John Hunter Hospital, New Lambton Heights, New South Wales); Allan Soward (Mildura Cardiology, Mildura, Victoria); Philip E Aylward (South Australian Health and Medical Research Institute, Flinders University and Medical Centre, Adelaide South Australia); Chris LS Hii (Calvary Health Care, Bruce, Australian Capital Territory); Philip E Aylward (Heart and Vascular Institute, Fullarton, South Australia); James Shaw (The Alfred Hospital, Prahran, Victoria); Margaret A Arstall (Lyell McEwin Hospital Elizabeth Vale, South Australia); John Horowitz and Daniel Ninio (The Queen Elizabeth Hospital, Woodville South, South Australia); James F Rogers (Gosford Hospital, Gosford, New South Wales); David Colquhoun (Core Research Group Pty Ltd, Milton, Queensland); Romulo E Oqueli Flores (Ballarat Health Services, Ballarat, Victoria); Philip Roberts-Thomson (Royal Hobart Hospital, Hobart, Tasmania); Owen Raffel (Prince Charles Hospital, Chermside, Queensland); Sam J Lehman (Adelaide Medical Research, Ashford, South Australia); Constantine Aroney (Holy Spirit Northside Hospital, Chermside, Queensland); Steven GM Coverdale (Nambour General Hospital, Nambour, Queensland); Paul J Garrahy (Princess Alexandra Hospital, Woolloongabba, Queensland); Gregory Starmer (The Cairns Hospital, Cairns, Queensland); Mark Sader (St George Hospital, Kogarah, New South Wales); Patrick A Carroll (Redcliffe Hospital, Redcliffe, Queensland); Ronald Dick (Epworth Healthcare, Richmond, Victoria).

**Austria (58 patients enrolled)** Robert Zweiker (Universitätsklinikum Graz, Graz); Uta Hoppe (Universitaetsklinikum Salzburg, Salzburg); Heinz Drexel (Vorarlberg Institut für Vaskuläre Forschung (VIVIT), Feldkirch); Kurt Huber (Wilhelminenspital der Stadt Wien, Wien); Rudolf Berger and Georg Delle-Karth and Bernhard Frey (Universitätsklinik für Innere Medizin II Währinger Gürtel 18-20; Franz Weidinger (Krankenanstalt Rudolfstiftung der Stadt Wien, Wien).

**Belgium (197 patients enrolled)** Dirk Faes (Mariaziekenhuis vzw, Overpelt); Kurt Hermans (AZ St. Lucas, Gent); Bruno Pirenne (Clinique Saint-Pierre, Ottignies); Attilio Leone (CHU Tivoli, La Louviere); Etienne Hoffer (CHR De La Citadelle, Liège); Peter Sinnaeve (UZ Leuven, Leuven); Mathias CM Vrolix (ZOL, Genk); Luc De Wolf (Private Practice, Tienen); Bart Wollaert (ZNA Stuivenberg, Antwerpen); Marc Castadot (Clinique Saint Jean, Bruxelles); Karl Dujardin (AZ Delta, Roeselare); Christophe Beauloye (UCL Saint Luc, Bruxelles); Geert Vervoort (AZ St.Maarten, Mechelen); Harry Striekwold (Heilig Hart Ziekenhuis, Mol); Carl Convens (ZNA Middelheim, Antwerpen); John Roosen (Imelda Hospital, Bonheiden); Emanuele Barbato (O.L Vrouwziekenhuis Campus Aalst, Aalst); Marc Claeys (UZ Antwerpen, Edegem); Frank Cools (AZ Klina, Brasschaat).

**Bosnia and Herzegovina (156 patients enrolled)** Ibrahim Terzic (Heart Center BH, Tuzla); Fahir Barakovic (University Medical Center Tuzla, Tuzla); Zlatko Midzic (Cantonal Hospital "Dr. Irfan Ljubijankic" Bihac, Bihac); Belma Pojskic (Cantonal Hospital Zenica, Zenica); Emir Fazlibegovic (University Clinical Hospital Mostar, Mostar); Mirza Dilic (University Clinical Center Sarajevo, Sarajevo); Azra Durak-Nalbantic and Mehmed Kulić (University Clinical Center of Sarajevo, Sarajevo); Dusko Vulic (Clinical Center Banja Luka, Banja Luka); Adis Muslibegovic (Regional Medical Center „Dr. Safet Mujic', Mostar); Boris Goronja (Clinical Center Banja Luka, Banja Luka).

**Brazil (928 patients enrolled)** Gilmar Reis (CARDRESEARCH - Cardiologia Assistencial e de Pesquisa, Belo Horizonte, Minas Gerais); Luciano Sousa (Hospital do Coracao do Brasil, Brasilia, Distrito Federal); Jose C Nicolau (INCOR - Instituto do Coracao, Sao Paulo, Sao Paulo); Flavio E Giorgeto (Hospital Sao Francisco De Assis, Belo Horizonte, Minas Gerais); Ricardo P Silva (Hospital Universitário Walter Cantídio, Fortaleza); Lilia Nigro Maia (Hospital De Base de São José do Rio Preto, Sao Jose Do Rio Preto, Sao Paulo); Rafael Rech (Hospital Universitário Ulbra, Canoas); Paulo RF Rossi (Hospital Evangelico De Curitiba, Curitiba, Parana); Maria José AG Cerqueira (Instituto de Ensino e Pesquisa Clínica do Ceara Ltda., Fortaleza, Ceara); Norberto Duda (Hospital São Vicente De Paulo, Passo Fundo, Rio Grande do Sul); Renato Kalil (Instituto De Cardiologia Do Rio Grande Do Sul, Porto Alegre, Rio Grande do Sul); Adrian Kormann (Sociedade Divina Providência (Hospital Santa Isabel), Blumenau, Santa Catarina); José Antonio M Abrantes (Santa Casa de Misericórdia de Pelotas, Pelotas, Rio Grande do Sul); Pedro Pimentel Filho (Hospital Nossa Senhora da Conceicao, Porto Alegre, Rio Grande do Sul); Ana Priscila Soggia (Sociedade Beneficiente de Senhoras Hospital Sírio Libanês, Sao Paulo); Mayler ON de Santos (Centro de Cardiologia e Radiologia Intervencionista LTDA, Aparecida de Goiania); Fernando Neuenschwander (Hospital Vera Cruz - NUPEC, Belo Horizonte); Luiz C Bodanese (Hospital Sao Lucas - PUCRS, Porto Alegre, Rio Grande do Sul); Yorghos L Michalaros (Instituto de Ensino, Consultoria e Pesquisa Clinica LTDA-ME, Belo Horizonte, Minas Gerais); Freddy G Eliaschewitz (CPCLIN - Centro de Pesquisas Clinicas, Sao Paulo, Sao Paulo); Maria H Vidotti (Clínica LOEMA, Campinas, Sao Paulo); Paulo E Leaes (Santa Casa De Porto Alegre, Porto Alegre, Rio Grande do Sul); Roberto V Botelho (Instituto Do Coracao Do Triangulo, Uberlandia, Minas Gerais); Sergio Kaiser (CCBR Brasil, Rio De Janeiro, Rio de Janeiro); Euler Roberto F Fernandes Manenti (IMV - Instituto De Medicina Vascular Hospital Mae de Deus, Porto Alegre, Rio Grande do Sul); Dalton B Precoma (Sociedade Hospitalar Angelina Caron, Campina Grande do Sul, Parana); Jose C Moura Jorge (Santa Casa De Curitiba, Curitiba, Parana); Pedro Silva (Esho Empresa de Serviço Hospitalares S.A, Sao Paulo, Sao Paulo); Jose A Silveira (Faculdade de Medicina do ABC / CEMEC, Sao Bernardo do Campo, Sao Paulo); Wladmir Saporito (Instituto de Moléstias Cardiovasculares Tatuí (IMC), Tatuí, Sao Paulo; and Hospital Estadual Mario Covas, Santo Andre); Jose A Marin Neto (HCFMRP, Riberao Preto); Gilson S Feitosa (Hospital Santa Izabel / Santa Casa de Misericordia da Bahia, Salvador, Bahia); Luiz Eduardo F Ritt (Hospital Cardio Pulmonar, Salvador; Hospital Cardio Pulmonar at Salvador); Juliana A de Souza (Centro de Cardiologia Clinica e Pesquisa Dra. Juliana Souza, Brasilia, Distrito Federal); Fernando Costa (FGM-Clinica Paulista de Doencas Cardiovasculares, Sao Paulo); Weimar KSB Souza (Barroso e Sebba Ltda, Goiania); Helder JL Reis (Hospital De Clinicas Gaspar Vianna, Belem, Para); Renato D. Lopes (Universidade Federal de São Paulo - UNIFESP/EPM); Leandro Machado (Hospital Estadual Mario Covas, Santo Andre); José Carlos Aidar Ayoub (Instituto de Moléstias Cardiovasculares/IMC, São José do Rio Preto).

**Bulgaria (333 patients enrolled)** Georgi V Todorov (2nd MHAT, Sofia); Fedya P Nikolov (Univ Multiprofile Hosp for Active Treatment "Sveti Georgi", Plovdiv); Elena S Velcheva (Specialized Hospital for Active Treatment in Cardiology, Pleven); Maria L Tzekova (UMHAT dr Georgi Stranski, Pleven); Haralambi O Benov (Multiprof Distr Hosp for Active Treatment "Dr.St.Cherkezov", Veliko Tarnovo); Stanislav L Petranov (Multiprofile Hospital for Active Treatment - Burgas AD, Burgas); Haralin S Tumbev (Specialized Hosp. for Active Cardio. Treatment–“Cardiolife”, Varna); Nina S Shehova-Yankova (MHAT Bratan Shukerov AD, Cardio. Dept. with Inrtensive Unite, Smoljan); Dimitar T Markov (UMHAT Tzaritza Yoanna – ISUL EAD, Sofia); Dimitar H Raev (MHAT 'Sveti Vrach' EOOD, Sandanski); Mihail N Mollov (First Multiprofile Hospital for Active Treatment - Sofia EAD, Sofia); Kostadin N Kichukov ("MHAT Lyulin" EAD. Department of Internal Diseases, Sofia); Katya A Ilieva-Pandeva (MHAT Blagoevgrad AD, Blagoevgrad); Nina N Gotcheva (MHAT “National Cardiology Hospital” EAD, Sofia); Raya Ivanova and Maryana Gospodinov (UMHAT Alexandrovska EAD, Sofia); Valentina M Mincheva (NMT Hospital "Tsar Boris III", Sofia); Petar V Lazov (Multiprofile Hospital for Active Treatment - Pazardzhik AD, Pazardzhik); Bojidar I Dimov (Fifth MHAT-Sofia EAD, Sofia).

**Canada (361 patients enrolled)** Manohara Senaratne (Dr. M. P. J Senaratne Professional Corporation, Edmonton, Alberta); James Stone (TotalCardiology, Calgary, Alberta); Jan Kornder (deceased), Stephen Pearce (Surrey Memorial Hospital, Surrey, British Columbia); Danielle Dion (Centre Hospitalier Beauce-Etchemin, St. Georges de Beauce, Quebec); Daniel Savard (CardioVasc HR, St. Jean sur Richelieu, Quebec); Yves Pesant (Saint Jerome Medical Research, Inc., St. Jerome); Amritanshu Pandey (Cambridge Cardiac Care Center, Cambridge, Ontario); Simon Robinson (Victoria Heart Institute Foundation, Victoria, British Columbia); Gilbert Gosselin (Centre Hospitalier Pierre Le Gardeur, Terrebonne, Quebec); Saul Vizel (Vizel Cardiac Research, Cambridge, Ontario); Gordon Hoag (Discovery Clinical Services, Victoria, British Columbia); Ronald Bourgeois (G.A. Research Associates, Ltd., Moncton, New Brunswick); Anne Morisset (Centre de Santé et de Services Sociaux de la Haute-Yamaska, Granby, Quebec); Eric Sabbah (Centre de Depistage et Recherche Cardiovasculaire Rive-Sud, Longueuil, Quebec); Bruce Sussex (General Hospital Health Sciences Center, St. John’s, Newfoundland and Labrador); Simon Kouz (Centre Hospitalier Régional de Lanaudière, Joliette, Quebec); Paul MacDonald (Cape Breton Regional Hospital, Sydney, Nova Scotia); Ariel Diaz (Centre Hospitalier Régional de Trois-Rivières, Trois-Rivières, Quebec); Nicolas Michaud (Clinique de Cardiologie de Lévis, Lévis, Quebec); David Fell (Newmarket Cardiology Research Group, Newmarket, Ontario); Raymond Leung and Raymond Leung Royal Alexandra Hospital, Edmonton, Alberta); Tycho Vuurmans (Royal Columbian Hospital, New Westminster, British Columbia); Christopher Lai, Frank Nigro (current PI) (Thunder Bay Regional Health Science Center, Thunder Bay, Ontario); Richard Davies (Ottawa Heart Institute, Ottawa, Ontario); Gustavo Nogareda (Red Deer Regional Hospital Centre, Red Deer, Alberta); Ram Vijayaraghavan (Scarborough Cardiology Research, Scarborough, Ontario); John Ducas (St. Boniface Hospital, Winnipeg, Manitoba); Serge Lepage (Hôtel-Dieu du Centre Hospitalier Universitaire de Sherbrooke, Sherbrooke, Quebec); Shamir Mehta (Hamilton General Hospital, Hamilton, Ontario); James Cha (Private Practice affiliated Lakeridge Health of Oshawa, Oshawa, Ontario); Robert Dupuis (Centre de Santé et de Services Sociaux de la Région de Thetford (Recherche), Thetford Mines, Quebec); Peter Fong and Sohrab Lutchmedial (Saint John Regional Hospital, Saint John, New Brunswick); Josep Rodes-Cabau (Quebec Heart Institute, Laval Hospital, Sainte-Foy, Quebec City, Quebec); Hussein Fadlallah (Recherche GCP Research, Montreal, Quebec); David Cleveland (Penticton Regional Hospital, Penticton, British Columbia); Thao Huynh (Montreal General Hospital, Montreal, Quebec); Iqbal Bata (Queen Elizabeth II Health Sciences Center, Halifax, Nova Scotia); Adnan Hameed (Medical Professional Corporation, St. Catherines, Ontario).

**Chile (132 patients enrolled)** Cristian Pincetti (Centro de Investigacion Clinica del Sur Cics, Temuco, IX Region de la Araucanía); Sergio Potthoff (Corporacion de Beneficencia Osorno, Osorno, X Region los Lagos); Juan C Prieto (Hospital Clinico Universidad De Chile, Santiago); Monica Acevedo (Centro de Investigaciones Clínicas de la UniversidadCatólica, Santiago, RM Region Metropolitan); Arnoldo Aguirre (Hospital Barros Luco Trudeau, Santiago, RM Region Metropolitan); Margarita Vejar (Hospital del Salvador, Santiago); Mario Yañez (Hospital Naval Almirante Nef de Viña del Mar, Viña Del Mar, V Region de Valparaiso); Guillermo Araneda (Hospital Clinico Magallanes, Punta Arenas, XII Region Magallanes); Mauricio Fernandez (Clinica Alemana de Santiago, Santiago, RM Region Metropolitan); Luis Perez (Hospital Clinico Regional Dr. Guillermo Grant Benavente, Concepcion, VIII Region del Bio-Bio); Paola Varleta (Psicomedica Clinical and Research Group, Santiago, RM Region Metropolitan); Fernando Florenzano (Biomedica Research Group, Santiago, RM Region Metropolitan); Laura Huidobro (Clinica Universidad Catolica del Maule, Talca, VII Region del Maule); Carlos A Raffo (Estudios clinicos V Region Ltda, Viña del Mar); Claudia Olivares (Hospital y CRS El Pino, Santiago, RM Region Metropolitan); Leonardo Nahuelpan (Hospital Base Valdivia, Valdivia XIV, Region de los Rios); Humberto Montecinos (Complejo Asistencial Dr. Sotero del Rio, Santiago).

**China (614 patients enrolled)** Jiyan Chen (Guangdong General Hospital, Guangzhou); Yugang Dong (The First Affiliated Hospital, Sun Yat-Sen University, Guangzhou); Weijian Huang (The First Affiliated Hospital of Wenzhou Medical University, Wenzhou); Jianzhong Wang (Siping Central People's Hospital, Siping); Shi'An Huang (Affiliated Hospital of Guangdong Medical University, Zhanjiang); Zhuhua Yao (Tianjin Union Medical Center, Tianjin); Xiang Li and Lan Cui (Yanbian University Hospital, Yanji); Wenhua Lin (TEDA International Cardiovascular Hospital, Tianjin); Yuemin Sun (Tianjin Medical University General Hospital,); Jingfeng Wang (Sun Yat-Sen Memorial Hospital, Sun Yat-Sen University, Guangzhou); Jianping Li (Peking University First Hospital, Beijing); Xuelian Zhang (People's Hospital, Changchun); Hong Zhu (The Affiliated Hospital of Xuzhou Medical College, Xuzhou); Dandan Chen (Guizhou Provincial People’s Hospital, Guiyang); Lan Huang (Xinqiao Hospital of Third Military Medical University, Chongqing); Shaohong Dong (Shenzhen People’s Hospital, Shenzhen); Guohai Su (Jinan Central Hospital Affiliated to Shandong University, Jinan); Biao Xu (Nanjing Drum Tower Hospital, The Affiliated Hospital of NJMU, Nanjing); Xi Su (Wuhan Asian Heart Hospital, Wuhan); Xiaoshu Cheng (The Second Affiliated Hospital to Nanchang University, Nanchang); Jinxiu Lin (The First Affiliated Hospital of Fujian Medical University, Fuzhou); Wenxia Zong (The Third People’s Hospital of Hubei Province, Wuhan); Huanming Li (Tianjin 4th Centre Hospital, Tianjin); Yi Feng (Zhongda Hospital, Southeast University, Nanjing); Dingli Xu (Nanfang hospital, Southern Medical University, Guangzhou); Xinchun Yang (Beijing Chaoyang Hospital, Beijing); Yuannan Ke (China-Japan Friendship Hospital, Beijing); Xuefeng Lin (The First Affiliated Hospital of Baotou Medical College, Baotou); Zheng Zhang (The First Hospital of Lanzhou University, Lanzhou); Zeqi Zheng (The First Affiliated Hospital of Nanchang University, Nanchang); Zhurong Luo (Fuzhou General Hospital of Nanjing Military Command, Fuzhou); Yundai Chen (Chinese PLA General Hospital, Beijing); Chunhua Ding and Yi Zhong (The Central Hospital of China Aerospace Corporation, Beijing); Yang Zheng (First Hospital of Jilin University, Changchun); Xiaodong Li (Shengjing Hospital of China Medical University, Shenyang); Daoquan Peng and Shuiping Zhao (The Second Xiangya Hospital of Central South University, Changsha); Ying Li and Xuebo Liu (Shanghai East Hospital, Shanghai); Meng Wei (Shanghai Sixth People's Hospital, Shanghai); Shaowen Liu (Shanghai First People's Hospital, Shanghai); Yihua Yu (Zhejiang Hospital, Hangzhou); Baiming Qu (Zhejiang Provincial People’s Hospital, Hangzhou); Weihong Jiang (The Third Xiangya Hospital Of Central South University, Changsha); Yujie Zhou (Beijing Anzhen Hospital, Capital Medical University, Beijing); Xingsheng Zhao (Inner Mongolia People's Hospital, Hohhot); Zuyi Yuan (The First Affiliated Hospital of Xi'an JiaoTong University, Xi'An); Ying Guo (Hunan Provincial People's Hospital, Changsha); Xiping Xu (The First People's Hospital of Yueyang, Yueyang); Xubo Shi (Beijing Tongren Hospital, Capital Medical University, Beijing); Junbo Ge (Zhongshan Hospital Fudan University, Shanghai); Guosheng Fu (Sir Run Run Shaw Hospital of Zhejiang University, Hangzhou); Feng Bai (Lanzhou University Second Hospital, Lanzhou); Weiyi Fang (Shanghai Chest Hospital Shanghai Jiaotong University, Shanghai); Xiling Shou (Shaanxi Provincial People's Hospital, Xi'an); Xiangjun Yang (First Affiliated Hospital Of Soochow University, Suzhou); Jian'An Wang and Meixiang Xiang (The Second Affiliated Hospital of Zhejiang University, Hangzhou); Yingxian Sun (First Hospital of China Medical University, Shenyang); Qinghua Lu (Second Hospital of Shandong University, Jinan); Ruiyan Zhang (Ruijin Hospital Affiliated to Shanghai Jiaotong University, Shanghai); Jianhua Zhu (First Affiliated Hospital of Zhejiang University, Hangzhou); Yizhou Xu (Hangzhou First People's Hospital, Hangzhou); Zhongcai Fan (Affiliated Hospital of Luzhou Medical College, Luzhou); Tianchang Li (Navy General Hospital, Cardiology Center, Beijing); Chun Wu (Peking University Shenzhen Hospital, Shenzhen).

**Colombia (354 patients enrolled)** Nicolas Jaramillo (Centro de Medicina del Ejercicio y Rehabilitacion Cardiaca, Medellin); Gregorio Sanchez Vallejo (Fundación Cardiomet CEQUIN, Armenia, Quindio ); Diana C Luna Botia (Hospital Santa Clara, Bogota); Rodrigo Botero Lopez (IPS Rodrigo Botero SAS, Medellin); Dora I Molina De Salazar (Asociacion IPS Medicos Internistas de Manizales, Caldas); Alberto J Cadena Bonfanti and Carlos Cotes Aroca (Clinica de la Costa ltda, Barranquilla); Juan Diego Higuera and Marco Blanquicett (Fundación Oftalmológica FOSCAL,); Sandra I Barrera Silva (Caja de Compensacion Familiar CAFAM, Bogota); Henry J Garcia Lozada (Fundacion Centro de Investigaciones Biomedicas Riescard, Espinal - Tolima); Julian A Coronel Arroyo (IPS Centro Medico Julian Coronel S.A., Cali, Valle); Jose L Accini Mendoza (IPS Centro cientifico asistencial SAS, Barranquilla); Ricardo L Fernandez Ruiz and Alvaro M. Quintero Ossa (Centro Cardiovascular Colombiano Clinica Santa Maria CARDIOVID Medellin, Antioquia); Fernando G Manzur Jatin (Centro de diagnostico cardiologico, Cartagena); Aristides Sotomayor Herazo (Centro Cardiovascular Santa Lucia, Cartagena, Bolivar); Jeffrey Castellanos Parada and Rafael Suarez Arambula (SIMEDICS IPS S.A.S, Bogota D.C.); Miguel A Urina Triana (Fundacion del Caribe para la Investigacion Biomedica, Barranquilla); Angela M Fernandez Trujillo (Centro Medico Imbanaco de Cali S.A, Cali, Valle).

**Croatia (70 patients enrolled)** Maja Strozzi (Clinical Hospital Center Zagreb, Zagreb); Siniša Car (General Hospital Varazdin, Varazdin); Melita Jerić (General Hospital Varazdin, Varazdin), Davor Miličić (University Hospital Center Zagreb, Zagreb); Martina Lovrić Benčić (Clinical Hospital Center Zagreb, Zagreb); Hrvoje Pintarić (University Hospital Centre „Sestre Milosrdnice“, Zagreb); Đeiti Prvulović (General Hospital Slavonski Brod, Slavonski brod); Jozica Šikić (Clinical Hospital Sveti Duh, Zagreb); Viktor Peršić (Thalassotherapia-Opatija, Opatija); Dean Mileta (Clinical Hospital Merkur, Zagreb); Kresimir Štambuk (Special Hosp. for Med. Rehab.Krapinske Toplice, Krapinske Toplice); Zdravko Babić (Clinical Hospital Center, 'Sestre Milosrdnice', Zagreb); Vjekoslav Tomulic (Clinical Hospital Center Rijeka, Rijeka); Josip Lukenda and Stanka Mejic-Krstulovic (General Hospital Dubrovnik, General Hospital Dubrovnik, Dubrovnik); Boris Starcevic (Clinical Hospital Dubrava, Clinical Hospital Dubrava, Zagreb).

**Czech Republic (381 patients enrolled)** Jindrich Spinar (FN Brno, Brno); David Horak and Zdenek Velicka (Krajska Nemocnice Liberec a.s., Liberec); Josef Stasek (Fakultni Nemocnice Hradec Kralove, Hradec Kralove); David Alan (Fakultni Nemocnice v Motole, Praha); Vilma Machova (Interni a kardiologicka ambulance, InterkardioML, s.r.o., Marianske Lazne); Ales Linhart (Vseobecna Fakultni Nemocnice V Praze, Praha); Vojtech Novotny (Nemocnice Pardubickeho kraje, a.s., Pardubice); Vladimir Kaucak (Mestska Nemocnice Ostrava, Ostrava); Richard Rokyta (Fakultni Nemocnice Plzen, Plzen); Robert Naplava (Centrum pro choroby srdce a cev, Kromeriz); Zdenek Coufal (Krajska Nemocnice Tomase Bati a.s., Zlin); Vera Adamkova (Institut klinicke a experimentalni mediciny, Praha); Ivo Podpera (Oblastni Nemocnice Kladno a.s., Kladno); Jiri Zizka (Thomayerova nemocnice, Praha); Zuzana Motovska (Fakultni Nemocnice Kralovske Vinohrady, Praha); Ivana Marusincova (Kardio Sever, r.o., Ceska Lipa); Premysl Svab (Kardiologicka ambulance, Rakovnik); Petr Ostadal (Nemocnice Na Homolce, Praha); Petr Heinc (Fakultni Nemocnice Olomouc, Olomouc); Jiri Kuchar (Kardiologie s.r.o., Cesky Krumlov); Petr Povolny (Cardiocentrum Kladno s.r.o., P-P klinika Kladno, Kladno); Jiri Matuska (Interni a cevni ambulance, Hodonin).

**Denmark (352 patients enrolled)** Steen H Poulsen (Aarhus Universitetshospital Skejby, Aarhus N); Bent Raungaard (Dep Cardiology, Aalborg); Peter Clemmensen; Lia E Bang (Rigshospitalet Hjertecentret, Copenhagen); Ole May; Morten Bøttcher (Region Hospital Herning, Herning); Jens D Hove (Hvidovre Hospital, Hvidovre); Lars Frost (Regionshospitalet Silkeborg, Silkeborg); Gunnar Gislason (Gentofte Hospital, Hellerup); John Larsen (Næstved Sygehus, Næstved);Peter Betton Johansen; Flemming Hald and Peter Johansen (Sygehus Lillebælt, Vejle Sygehus, Vejle); Jørgen Jeppesen (Glostrup Hospital, Glostrup); Tonny Nielsen (Sjællands Universitetshospital Køge, Køge); Kjeld S Kristensen; Piotr Maria Walichiewicz (Holbaek Sygehus, Holbaek); Jens D Lomholdt (Slagelse Sygehus, Slagelse); Ib C Klausen (Regionshospitalet Viborg, Viborg); Peter Kaiser Nielsen (Hillerød Hospital, Hillerød); Flemming Davidsen (Sygehus Sønderjylland, Aabenraa); Lars Videbaek (Odense Universitetshospital, Odense C).

**Estonia (216 patients enrolled)** Margus Viigimaa (North Estonia Medical Centre, Tallinn); Mai Soots (SA Viljandi Haigla, Viljandimaa); Veiko Vahula (SA Pärnu Haigla, Pärnu); Anu Hedman (AS Ida-Tallinna Keskhaigla, Tallinn); Üllar Soopõld (SA Tartu Ülikooli Kliinikum, Tartu); Kaja Märtsin and Tiina Jurgenson (Mustamäe Tervisekeskus OÜ, Tallinn); Arved Kristjan (Mahe Perearst OU, Tallinn).

**Finland (116 patients enrolled) Markku S Nieminen**, (Biomedicum and Division of Cardiology, Helsinki University Hospital, Juhani K Airaksinen); Saila Vikman Heart Center, Tampere University Hospital; Heikki Huikuri (University Hospital of Oulu), Juhani Airaksinen (Turun Yliopistollinen sairaala Sydänkeskus)**France (185 patients enrolled)** Pierre Coste (Hôpital Cardiologique du Haut-Leveque, Pessac); Emile Ferrari (Hôpital Pasteur, Nice); Nicolas Danchin (Hôpital Europeen Georges Pompidou, Paris); Olivier Morel (Nouvel Hôpital Civil, Strasbourg); Gilles Montalescot (Hôpital Pitié Salpêtrière, Paris); Jacques Machecourt and Gilles Barone-Rochette (CHU Albert Michallon, Grenoble); Jacques Mansourati (CHU De Brest Hôpital de la Cavale Blanche, Brest); Yves Cottin (CHU de Dijon Le Bocage Central, Dijon); Ph. Gabriel Steg (Hôpital Bichat, Paris); Florence Leclercq (Hôpital Arnaud de Villeneuve, Montpellier); Abdelkader Belhassane (Centre Hospitalier de Cambrai, Cambrai); Nicolas Delarche (Centre Hospitalier Général, Pau); Franck Boccara (Hôpital Saint-Antoine, Paris); Franck Paganelli (Hôpital Nord, Marseille); Jérôme Clerc (CH Compiègne - Noyon, Compiegne); Francois Schiele (CHU Jean Minjoz, Besancon); Victor Aboyans (CHU Dupuytren, Limoges); Vincent Probst (CHU de Nantes, Nantes); Jacques Berland (Clinique Saint Hilaire, Rouen); Thierry Lefèvre (Institut Hospitalier Jacques Cartier, Massy); Bernard Citron (CHU Gabriel Montpied, Clermont-Ferrand).

**Georgia (131 patients enrolled)** Vakhtang Chumburidze (Chapidze Emergency Cardiology Center, Tbilisi); Irakli Khintibidze ("Aleksandr Aladashvili Clinic" LLC, Tbilisi); Tamaz Shaburishvili (Tbilisi Heart and Vascular Clinic, Ltd, Tbilisi); Zurab Pagava (Center of vascular and heart diseases, Tbilisi); Ramaz Ghlonti (Unimed Ajara Batumi Refferal Hospital, Batumi); Zaza Lominadze (LTD "Clinic-LJ", Kutaisi); George Khabeishvili (Tbilisi Heart and Vascular Clinic, Ltd, Tbilisi).

**Germany (509 patients enrolled)** Rayyan Hemetsberger and Kemala Edward (St. Johannes Hospital, Dortmund); Ursula Rauch-Kröhnert (Charité - Universitätsmedizin Campus Benjamin Franklin, Berlin); Matthias Stratmann (Kardiologische Gemeinschaftspraxis, Dortmund); Karl-Friedrich Appel (Studienzentrum Dr. Appel, Kassel); Ekkehard Schmidt (Cardiologicum Hamburg, Hamburg); Heyder Omran (GFO Kliniken Bonn, Bonn); Christoph Stellbrink (Klinikum Bielefeld Mitte, Bielefeld); Thomas Dorsel (Joseph-Hospital Warendorf, Warendorf); Emmanouil Lianopoulos and Hans Friedrich Vöhringer (DRK Kliniken Berlin-Köpenick, Berlin); Roger Marx (MediClin Fachklinik Rhein/Ruhr, Essen); Andreas Zirlik (Universitätsherzzentrum Freiburg, Freiburg); Detlev Schellenberg (Institut für Klinische Forschung, Rotenburg an der Fulda); Thomas Heitzer (Klinikum Dortmund, Dortmund); Ulrich Laufs and Christian Werner (Universitätsklinikum des Saarlandes, Homburg/Saar); Nikolaus Marx (Universitätsklinikum Aachen, Aachen); Stephan Gielen and Sebastian Nuding (Universitätsklinikum Halle/Saale, Halle (Saale); Bernhard Winkelmann (ClinPhenomics GmbH & Co. KG, Frankfurt am Main); Steffen Behrens (Vivantes Humboldt-Klinikum, Berlin); Karsten Sydow and Mahir Karakas (Universitäres Herzzentrum Hamburg GmbH, Hamburg); Gregor Simonis (FAZ Dresden-Neustadt GbR, Dresden); Thomas Muenzel (Universitätsklinikum Mainz, Mainz); Nikos Werner (Universitätsklinikum Bonn, Bonn); Stefan Leggewie (Universitäts-Herzzentrum Freiburg-Bad Krozingen, Bad Krozingen); Dirk Böcker (St. Marien-Hospital Hamm, Hamm); Rüdiger Braun-Dullaeus (Universitätsklinikum Magdeburg, Magdeburg); Nicole Toursarkissian (Praxis Dr. Nicole Toursarkissian, Berlin); Michael Jeserich (GP Dr. Haggenmiller, PD Dr. Michael Jeserich, Nürnberg); Matthias Weißbrodt (Gemeinschaftspraxis Dr. M. Löbe, Dr. M. Weißbrodt, Leipzig); Tim Schaeufele (Robert-Bosch-Krankenhaus GmbH, Stuttgart); Joachim Weil (Sana Kliniken Lübeck, Lübeck); Heinz Völler (Klinik am See, Rüdersdorf bei Berlin); Johannes Waltenberger (Universitätsklinikum Münster, Münster); Mohammed Natour (HPK-Heidelberger Privatklinik für Kardiologie, Heidelberg); Susanne Schmitt and Dirk Müller-Wieland (Asklepios Klinik St. Georg., Allgemeine Innere Medizin, Hamburg); Stephan Steiner (Krankenhausgesellschaft St. Vincenz mbH, Limburg); Lothar Heidenreich and Elmar Offers (Dreifaltigkeits-Hospital Lippstadt, Lippstadt); Uwe Gremmler (MVZ Ambulantes Kardiologisches Zentrum Peine GbR, Peine); Holger Killat (Praxis Dr. Holger Killat, Haßloch); Werner Rieker (Studienzentrum Rankestrasse, Berlin).

**Greece (70 patients enrolled)** Sotiris Patsilinakos ("Konstantopouleio” General Hospital of Nea Ionia, Athens); Athanasios Kartalis (“Skylitseio” General Hopsital of Chios, Chios); Athanassios Manolis (“Asklipeio” General Hospital, Athens); Dimitrios Sionis and Geargios Chachalis (“Sismanogleio” General Hospital of Attica, Marousi); Evangelos Liberopoulos (School of Medicine, National and Kapodistrian University of Athens, Athens, Greece ); Ioannis Skoumas (“Ippokrateio” General Hospital, Athens); Vasilios Athyros (“Ippokrateio” General Hospital, Thessaloniki); Panagiotis Vardas and Frangkiskos Parthenakis (University General Hospital of Heraklion, Crete); previous PI Dimitrios Alexopoulos / current PI Georgios Hahalis (University General Hospital of Patra, Patra); John Lekakis (“Attikon” University General Hospital, Athens); Apostolos Hatzitolios (AHEPA General Hospital of Thessaloniki, Thessaloniki).

**Guatemala (25 patients enrolled)** Sergio R Fausto Ovando (Cilinical Research Center, Guatemala); Pablo Carlos Montenegro Valdovinos (Clinica Privada, Guatemala); Juan L Arango Benecke (Clinicas Medicas; Dr. Arango, Guatemala); Edgar R Rodriguez De Leon (Unidad de Diagnostico Cardiologico, Guatemala).

**Hong Kong (17 patients enrolled)** Bryan PY Yan (Prince of Wales Hospital, Shatin, NT); David CW Siu (Queen Mary Hospital).

**Hungary (224 patients enrolled)** Tibor Turi (Fejér Megyei Szent György Egyetemi Oktató Kórház, Székesfehérvár); Bela Merkely (Semmelweis Egyetem Városmajori Szív-és Érgyógyászati Klinika, Budapest); Robert Gabor Kiss (Military Hospital, Budapest); Imre Ungi (Szegedi Tudományegyetem, Szeged); Geza Lupkovics (Zala Megyei Szent Rafael Kórház, Zalaegerszeg); Lajos Nagy (Markusovszky Egyetemi Oktatókórház, Szombathely); András Katona (Békés Megyei Központi Kórház, Gyula); István Édes (Debreceni Egyetem Klinikai Központ, Debrecen); Gábor Müller (Markhot Ferenc Oktatókórház és Rendelointézet, Eger); Iván Horvath (Pécsi Tudományegyetem Klinikai Központ, Pécs); Tibor Kapin and Zsolt Szigeti (Bajcsy-Zsilinszky KórházRendelöintézet, Budapest); József Faluközy (Állami Szívkórház Balatonfüred, Balatonfüred).

**India (521 patients enrolled)** Mukund Kumbla (Omega Hospital, Mangalore); Manjinder Sandhu (Artemis Health Institute, Gurgaon); Sharath Annam (Sunshine Hospital, Secunderabad); Naveen Reddy Proddutur and Reddy Regella (Mediciti Hospitals, Hyderabad); Rajendra K Premchand (Krishna Institute Of Medical Sciences, Secunderabad); Ajaykumar Mahajan and Sudhir Pawar (Lokmanya Tilak Municipal Medical College & General Hospital, Mumbai); Atul D Abhyanakar (Nirmal Hospital Pvt Ltd, Surat); Prafulla Kerkar (Seth GS Medical College & KEM Hospital, Mumbai); Ravishankar A Govinda (K.R.Hospital, Mysore); Abraham Oomman (Apollo Hospital, Chennai); Dhurjati Sinha (ICVS at IPGME&R, SSKM Hospital, Kolkata); Sachin N Patil (Aster Aadhar Hospital, Kolhapur); Dhiman Kahali (B. M. Birla Heart Research Centre, Kolkata); Jitendra Sawhney (Dharma Vira Heart Center, Sir Ganga Ram Hospital, Delhi); Abhijeet B Joshi (Niramay Hospital, Pune); Sanjeev Chaudhary (Fortis Hospital, Gurgaon); Pankaj Harkut (Meditrina Institute of Medical Sciences, Nagpur); Santanu Guha (Medical College and Hospital, Kolkata); Sanjay Porwal (KLEs Dr Prabhakar Kore Hospital & Medical Research Centre, Belgaum); Srimannarayana Jujjuru (Andhra Hospital, Vijaywada); Ramesh B Pothineni (Dr. Ramesh Cardiac & Multispeciality Hospital Ltd., Vijayawada); Minguel R Monteiro (UGH Vintage Hospital & Medical Research Centre Pvt Ltd, Panaji); Aziz Khan (Crescent hospital and heart clinic, Nagpur); Shamanna S Iyengar (Manipal Hospital, Bangalore, Karnataka); Jasprakash Singh Grewal (Satguru Pratap Singh Apollo Hospitals, Ludhiana); Manoj Chopda (Magnum Heart Institute, Nashik); Mahesh C Fulwani (Shrikrishna Hrudayalay and Critical Care Centre, Nagpur); Dr. Aparna Patange and Patil Sachin (Krishna Institute of Medical Sciences Deemed University, Karad, Maharashtra); Vijay K Chopra (Medanta - The Medicity, New Delhi); Naresh K Goyal (Max Super Speciality Hospital, Delhi, Delhi); Rituparna Shinde (Sanjeevan Hospital, Pune); Gajendra V Manakshe (Datta Meghe Institute of Medical Sciences (Deemed University, Wardha); Nitin Patki (Maharashtra Medical Foundation Joshi Hospital, Pune, Maharashtra); Sumeet Sethi (Max Heart & Vascular Institute, New Delhi); Vengatesh Munusamy (SRM Medical College Hospital And Research Centre, Chennai); Sunil Karnaand Sunil Thanvi (Bahnubhai And Madhuben Patel Cardiac Centre, Karamsad); Srilakshmi Adhyapak and Chandrakant Patil (St John's Medical College & Hospital, Bangalore); Ulhas Pandurangi (Madras Medical Mission, Chennai); Rishabh Mathur and Jugal Gupta (S. R. Kalla Memorial Gastro & General Hospital, Jaipur); Suhas Kalashetti (K. E .M Hospital, Pune, Maharashtra); Ajit Bhagwat (Kamal Nayan Bajaj Hospital, Aurangabad); Bagirath Raghuraman (Chinmaya Narayana superspecility hospital, Bangalore); Shiv Kumar Yerra (Mahavir Hospital And Research Centre, Hyderabad); Prasant Bhansali (AMC-MET medical College and Sheth L G General Hospital, Ahmedabad); Rohidas Borse and Patil Rahul (B.J. Government Med. College and Sassoon General Hospitals, Pune); Srihari Das and Vinay Kumar (NH-Narayana Multispecialty hospital, Bangalore); Jabir Abdullakutty (Lisie Hospital, Cochin); Shireesh Saathe (Deenanath Mangeshkar Hospital, Pune); Priya Palimkar (Jehangir Clinical Development centre, and Jehangir Hospital, Pune); Jabir Abdullkutty (Lisie Hospital, Kochi); Shireesh Sathe (Dmangeshkar Hospital, Pune).

**Israel (582 patients enrolled)** Shaul Atar (Western Galilee Medical Center, Nahariya); Michael Shechter (Sheba MC, Tel HaShomer); Morris Mosseri (Meir Medical Center, Kfar Saba); Yaron Arbel, Chorin Ehud and Havakuk Ofer (Tel Aviv Sourasky MC, Tel Aviv); Chaim Lotan (Hadassah Ein Kerem MC, Jerusalem); Uri Rosenschein (Bnai Zion Medical Center, Haifa); Amos Katz (Barzilai Medical Center, Ashkelon); Yaakov Henkin (Soroka Medical Center, Beer Sheva); Adi Francis (Holy Family Hospital, Nazareth); Marc Klutstein (Shaare Zedek Medical Center, Jerusalem); Eugenia Nikolsky and Robert Zukermann (Rambam MC, Haifa); Yoav Turgeman (Haemek Medical Center, Afula); Majdi Halabi and Alon Marmor (Ziv Medical Center, Safed); Ran Kornowski (Rabin Mc Belinson Campus, Petah-Tikva); Michael Jonas (Kaplan Medical Center, Rehovot); Offer Amir and Yonathan Hasin (The Baruch Padeh Medical Center, Poriya, Tiberias); Yoseph Rozenman (Wolfson Mc, Holon); Shmuel Fuchs and Vered Zvi (Rabin Mc Belinson Campus, Petah-Tikva); Osamah Hussein (Ziv Medical Center, Safed); Dov Gavish (Wolfson Mc, Holon); Zvi Vered (Assaf Harofe Medical Center, Tzrifin); Yoseph Caraco (Hadassah Ein Kerem MC, Jerusalem); Mazen Elias (Haemek Medical Center, Afula); Naveh Tov and Efrat Wolfovitz (Bnai Zion Medical Center, Haifa); Michael Lishner (Meir Medical Center, Kfar Saba); Nizar Elias (Bnei Zion MC, Haifa).

**Italy (275 patients enrolled)** Giancarlo Piovaccari (Ospedale Degli Infermi Rimini, Rimini); Annamaria De Pellegrin and Raffaella Garbelotto (UO Medicina Generale Ospedale di Vittorio Veneto, Vittorio Veneto); Gabriele Guardigli and Valgimigli Marco (Azienda Ospedaliera S. Anna, Ferrara); Giovanni Licciardello (Ospedale E. Moscatello, Augusta, Salerno); Carla Auguadro and Filippo Scalise (Policlinico di Monza, Monza); Claudio Cuccia (Fondazione Poliambulanza - Istituto Ospedaliero, Brescia); Alessandro Salvioni (Centro Cardiologico Fondazione Monzino, Milano); Giuseppe Musumeci and Michelle Senni (ASST-Papa Giovanni XXII, Bergamo); Paolo Calabrò (Azienda Ospedaliera Monaldi, Napoli); Salvatore Novo (A O Universitaria Policlinico P Giaccone, Palermo); Pompilio Faggiano and Marco Metra (Azienda Ospedaliera Spedali Civili di Brescia, Brescia); Nicoletta B De Cesare (Policlinico San Marco - Istituti Ospedalieri Bergamaschi, Osio Sotto, Bergamo); Sergio Berti (Fond.Toscana G.Monasterio-Presidio di Massa-Osped. del Cuore, Massa, Massa-Carrara); Claudio Cavallini (Ospedale S Maria Della Misericordia, Perugia); Enrico Puccioni (Ospedale Civile di Livorno - Divisione Cardiologia-UTIC, Livorno); Marcello Galvani (Ospedale Morgagni-Pierantoni, Forlì, Forlì-Cesena); Maurizio Tespili (Ospedale Bolognini, Seriate, Bergamo); Piermarco Piatti (Ospedale San Raffaele, Milano); Michela Palvarini (A.O. Guido Salvini - P.O. di Passirana, Rho, Milano); Giuseppe De Luca (Azienda Ospedaliera Maggiore Della Carità, Novara); Roberto Violini (Azienda Ospedaliera San Camillo-Forlanini, Roma); Alessandro De Leo and Zoran Olivari (Ospedale Civile Treviso Presidio Ospedaliero Ca' Foncello, Treviso); Pasquale Perrone Filardi (Az. Ospedaliera Univ. Federico II, Napoli); Maurizio Ferratini (IRCCS Fondazione "Don Gnocchi" Centro "S. Maria Nacente", Milano); Vittorio Racca (IRCCS Fondazione "Don Gnocchi" Centro "S, Maria Nacente" – Cardiologia, Milano).

**Japan (204 patients enrolled)** Kazuoki Dai and Yuji Shimatani (Hiroshima City Hospital, Hiroshima-shi, Hiroshima); Haruo Kamiya (Japanese Red Cross Nagoya Daiichi Hospital, Nagoya-shi, Aichi); Kenji Ando (Kokura Memorial Hospital, Kitakyushu-shi, Fukuoka); Yoshihiro Takeda (Rinku General Medical Center, Izumisano-shi, Osaka); Yoshihiro Morino (Iwate Medical University Hospital, Morioka-shi, Iwate); Yoshiki Hata (Minamino Cardiovascular Hospital, Hachioji-shi, Tokyo); Kazuo Kimura (Yokohama City University Medical Center, Yokohama-shi, Kanagawa); Koichi Kishi (Tokushima Red Cross Hospital, Komatsushima-shi, Tokushima); Ichiro Michishita (Yokohama Sakae Kyosai Hospital, Yokohama-shi, Kanagawa); Hiroki Uehara (Urasoe General Hospital, Urasoe-shi, Okinawa); Toshinori Higashikata (National Health Insurance Komatsu Municipal Hospital, Komatsu-shi, Ishikawa); Atsushi Hirayama (Nihon University Itabashi Hospital, Itabashi-ku, Tokyo); Keiji Hirooka and Yasuji Doi (Saiseikai Senri Hospital, Suita-shi, Osaka); Satoru Sakagami (Kanazawa Medical Center, Kanazawa-shi, Ishikawa); Shuichi Taguchi (Mito Medical Center, Higashiibaraki-gun, Ibaraki); Akihiro Koike (Fukuoka-Higashi Medical Center, Koga-shi, Fukuoka); Hiroyuki Fujinaga (Tokushima Prefectural Central Hospital, Tokushima-shi, Tokushima); Shinji Koba (Showa University Hospital, Shinagawa-ku, Tokyo); Ken Kozuma (Teikyo University Hospital, Itabashi-ku, Tokyo); Tomohiro Kawasaki (Shin-Koga Hospital, Kurume-shi, Fukuoka); Yujiro Ono (Higashi-Hiroshima Medical Center, Higashihiroshima-shi, Hiroshima); Masatoshi Shimizu (Kobe Medical Center, Kobe-shi, Hyogo); Yousuke Katsuda (Fukuoka City Medical Association Hospital, Fukuoka-shi, Fukuoka); Atsuyuki Wada (Kusatsu General Hospital, Kusatsu-shi, Shiga); Toshiro Shinke (Kobe University Hospital, Kobe-shi, Hyogo); Takeshi Kimura (Kyoto University Hospital, Kyoto-shi, Kyoto); Junya Ako (Kitasato University Hospital, Sagamihara-shi, Kanagawa); Kenshi Fujii (Sakurabashi Watanabe Hospital, Osaka-shi, Osaka); Toshiyuki Takahashi (Tokyo Saiseikai Central Hospital, Minato-ku, Tokyo); Tomohiro Sakamoto and Koichi Nakao (Saiseikai Kumamoto Hospital, Kumamoto-shi, Kumamoto); Yutaka Furukawa (Kobe City Medical Center General Hospital, Kobe-shi, Hyogo); Hiroshi Sugino and Ritsu Tamura (Kure Medical Center and Chugoku Cancer Center, Kure-shi, Hiroshima); Toshiaki Mano and Masaaki Uematsu (Kansai Rosai Hospital, Amagasaki-shi, Hyogo); Noriaki Utsu (Minami Osaka Hospital, Osaka-shi, Osaka); Kashima Ito (Nerima Hospital, Nerima-ku, Tokyo); Takuya Haraguchi and Katsuhiko Sato (Caress Sapporo Tokeidai Memorial Clinic, Sapporo-shi, Hokkaido); Yasunori Ueda (Osaka National Hospital, Osaka-shi, Osaka); Akira Nishibe (Amagasaki New Town Hospital, Amagasaki-shi, Hyogo); Kazuteru Fujimoto (Kumamoto Medical Center, Kumamoto-shi, Kumamoto); Motomaru Masutani and Akira Nishibe (Human Medical Amagasaki New Town Hospital, Amagasaki-shi); Kazuteru Fujimoto (National Hospital Organization Kumamoto Medical Center, Kumamoto-shi).

**Korea, Republic of (94 patients enrolled)** Jung Han Yoon (Yonsei University Wonju Severance Christian Hospital, Wonju, Gangwon-do); **Sang-Hyun Kim and Hack-Lyoung Kim** (SMG Seoul National University Boramae Medical Center, Seoul, Seoul); Hun Sik Park (Kyungpook National University Hospital, Daegu); In-Ho Chae (Seoul National University Bundang Hospital, Seongnam, Gyeonggi-do); Moo Hyun Kim (Dong-A University Medical Center, Busan); Myung Ho Jeong (Chonnam National University Hospital, Gwangju, Gwangju); Seungwoon Rha (Korea University Guro Hospital, Seoul); Chongjin Kim (KyungHee University Hospital at Gangdong, Seoul, Seoul); Hyo-Soo Kim and Hae Young Kim (Seoul National University Hospital, Seoul, Seoul); Taekjong Hong (Pusan University Hospital, Busan, Busan); Seung-Jea Tahk (Ajou University Hospital, Suwon, Gyeonggi-Do); Youngkwon Kim (Dongguk University Medical Center, Goyang-si, Gyeonggi-Do).

**Latvia (80 patients enrolled)** Arija Busmane (Adoria, Sabiedriba ar ierobežotu atbildibu, Riga); Natalija Pontaga (SIA "Daugavpils regionala slimnica, Daugavpils); Aldis Strelnieks (Rigas Austrumu kliniska universitates slimnica, SIA, Riga); Iveta Mintale (Pauls Stradins Clinical University Hospital, Riga); Iveta Sime (Regional Hospital of Liepaja, Liepaja).

**Lithuania (188 patients enrolled)** Zaneta Petrulioniene (Vilnius University Hospital Santaros klinikos, Vilnius); Roma Kavaliauskiene (Clinic of Cardiology and Rehabilitation, Klaipeda); Ruta Jurgaitiene (Hospital of Lithuanian University of Health Sciences, Kaunas); Gintare Sakalyte and Rimvydas Slapikas (Hospital of Lithuanian University of Health Sciences, Kaunas); Sigute Norkiene (Klaipeda Seamen's hospital, Klaipeda); Nerijus Misonis (Private Medicine Center Kardivita, Vilnius); Aleksandras Kibarskis (Private Cardiologic Clinic Sirdies Namai, Vilnius); Raimondas Kubilius (Elite Medicale, Kaunas).

**Macedonia, The Former Yugoslav Republic of (132 patients enrolled)** Stojko Bojovski (Cardiological Diagnostic Center, Tetovo); Sasko Kedev (University Clinic of Cardiology, Skopje); Nensi Lozance (Clinical hospital dr. Trifun Panovski, Bitola, Bitola); Aleksandar Kjovkaroski (Military medical Center, Skopje); Snezana Doncovska (PHI Clinical hospital Shtip, Shtip).

**Malaysia (110 patients enrolled)** Tiong Kiam Ong (Sarawak General Hospital, Kuching); Sazzli Kasim (Universiti Teknologi MARA (UiTM), Sungai Buloh Campus, Sungai Buloh, Selangor); Oteh Maskon (Hospital Universiti Kebangsaan Malaysia, Kuala Lumpur); Balachandran Kandasamy (Institut Jantung Negara, Kuala Lumpur, Kuala Lumpur); Khalid Yusoff (Universiti Teknologi MARA (UiTM) Selayang Campus, Batu Caves and UCSI University); Houng B Liew (Queen Elizabeth Hospital II, Kota Kinabalu); Wan Mohd Izani Wan Mohamed (Hospital Universiti Sains Malaysia, Kota Bharu).

**Mexico (349 patients enrolled)** Armando García Castillo (Cardiolink Clin Trials S.C., Monterrey, Nuevo León); Gabriel Arturo Ramos López (Medical Office, Guadalajara, Jalisco); Jorge Carrillo Calvillo (Hospital Central "Dr. Ignacio Morones Prieto", San Luis Potosi, San Luis Potosi); Pedro Fajardo Campos (CINCAME Centro de Investigación Cardiovascular y Metabólica, Tijuana, Baja California Norte); Juan Carlos Núñez Fragoso (Lahoja Asociación para la Investigación y la Farmacovigilancia S.C., Durango, Durango); Edmundo Alfredo Bayram Llamas (Fundación Cardiovascular de Aguascalientes A.C., Aguascalientes, Aguascalientes); Marco Antonio Alcocer Gamba (Centro de Estudios Clínicos de Querétaro, S.C., Querétaro, Querétaro); Jaime Carranza Madrigal (Unidad de Prevención y Atención Metabólica, Morelia, Michoacan); Luis Gerardo González Salas (Centro para el Desarrollo de la Medicina y de Asistencia Médica Especializada, S.C., Culiacán, Sinaloa); Enrique López Rosas (Centro de Alta Especialidad "Dr. Rafael Lucio", Jalapa, Veracruz); Belinda González Díaz (UMAE Hospital de Cardiología del Centro Médico Nacional Siglo XXI., Ciudad de México, México); Eduardo Salcido Vázquez (Antiguo Hospital Civil de Guadalajara "Fray Antonio Alcalde", Guadalajara, Jalisco); Alfredo Nacoud Ackar (Hospital Universitario "Dr. José Eleuterio González", Monterrey, Nuevo León); Guillermo Antonio Llamas Esperón (Hospital Cardiológica Aguascalientes, Aguascalientes, Aguascalientes); Carlos Rodolfo Martínez Sánchez (Instituto Nacional de Cardiología "Ignacio Chávez", Ciudad de México, México); María Guerrero De Leon (Avix Investigación Clínica, S.C., Monterrey, Nuevo León); Rodrigo Suarez Otero (INBIOMEDYC Toluca, Toluca, México); Guillermo Fanghänel Salmón (Clínica Integral del Paciente Diabético y Obeso, Ciudad de México, México); Jesús Antonio Pérez Ríos (OSMO Oaxaca Site Management Organization S.C., Oaxaca, Oaxaca); José Angel Garza Ruíz (IMED Internal Medicine Clin Trials, Monterrey, Nuevo León).

**Netherlands (686 patients enrolled)** Marco Alings (Amphia Ziekenhuis Molengracht, Breda); Robert W Breedveld, Margriet Feenema-Aardema and Alida Borger-Van Der Burg (Medisch Centrum Leeuwarden, Leeuwarden); Pieter AM Hoogslag (Diaconessenhuis Meppel, Meppel); Harry Suryapranata (Radboud University Medical Center, Nijmegen); Antonius Oomen, Paulus Van Haelst and Margriet Feenema-Aradema (D & A Research, Sneek); Jacobijne J Wiersma and Dirk Basart (Vasculair Onderzoeks Centrum BV, Hoorn); Ruud MA Van Der Wal and Peter Zwart (Ziekenhuis Bernhoven, Uden); Pascalle Monraats and Henricus Van Kesteren (Admiraal De Ruiter Ziekenhuis, Goes); Ioannis Karalis and Johan Jukema (Leiden University Medical Center, Leiden); Gerardus JE Verdel (Spaarne Gasthuis, Haarlem); Bart RG Brueren (Catharina Ziekenhuis, Eindhoven); Roland PTh Troquay (VieCuri Medisch Centrum Voor Noord-Limburg, Venlo); Eric P Viergever (Groene Hart Ziekenhuis, Gouda); Nadea YY Al-Windy (Gelre Ziekenhuis Zutphen, Zutphen); Gerard L Bartels (Martini Ziekenhuis, Groningen); Jan H Cornel (Noordwest Ziekenhuisgroep, locatie Alkmaar, Alkmaar); Walter RM Hermans (St Elisabeth Ziekenhuis, Tilburg); Johannes PR Herrman (OLVG locatie Oost, Amsterdam); Robert J Bos (Bravis Ziekenhuis, Roosendaal); Reginald GEJ Groutars (OLVG locatie Oost, Amsterdam); Coenraad C Van Der Zwaan (Ziekenhuis Rivierenland, Tiel); Refik Kaplan and Raymond Lionarons (Röpcke-Zweers Ziekenhuis, Hardenberg); Eelko Ronner (Reinier De Graaf Gasthuis, Delft); Bjorn E Groenemeijer (Gelre Diagnostisch Centrum, Apeldoorn); Patrick NA Bronzwaer (Zaans Medisch Centrum, Zaandam); Anho AH Liem (Sint Franciscus Gasthuis, Rotterdam); Bernard JWM Rensing (St Antonius Ziekenhuis, Nieuwegein); Marcel JJA Bokern (Waterlandziekenhuis, Purmerend); Remco Nijmeijer (Tjongerschans Hospital Heerenveen, Heerenveen); Ferry MRJ Hersbach (Maasstad ziekenhuis, Rotterdam); Frank F Willems (Rijnstate Ziekenhuis, Arnhem); Antonius TM Gosselink and Saman Rasoul (Isala Klinieken, Zwolle).

**New Zealand (257 patients enrolled)** John Elliott (Christchurch Hospital, Christchurch, Canterbury); Gerard Wilkins (Dunedin Hospital, Dunedin, Otago); Raewyn Fisher (Waikato Hospital, Hamilton, Waikato); Douglas Scott (Middlemore Hospital, Otahuhu, Auckland); Hamish Hart (North Shore Hospital, Takapuna, Auckland); Ralph Stewart (Auckland City Hospital, Grafton, Auckland); Scott Harding (Wellington Hospital, Newtown, Wellington); Ian Ternouth (Taranaki Base Hospital, New Plymouth, Taranaki); Nicholas Fisher and Samuel Wilson (Nelson Hospital, Nelson, Nelson); Denise Aitken (Lakes District Health Board, Rotorua, Bay of Plenty); Russell Anscombe (Hutt Valley District Health Board, Lower Hutt, Wellington); Laura Davidson (Palmerston North Hospital, Palmerston North, Manawatu-Wanganui).

**Norway (97 patients enrolled)** Tadeusz Tomala (Svelvik Legesenter, Svelvik); Ottar Nygård (Helse Bergen HF, Bergen); Jon Arne Sparby (Sykehuset innlandet HF, Kongsvinger, Kongsvinger); Kjell Andersen (Sykehuset Innlandet HF, Hamar, Hamar); Lars Gullestad (Oslo Universitetssykehus HF, Oslo); Jarle Jortveit (Sørlandet sykehus HF Arendal, Arendal); Peter S Munk and Erlend gyllensten Singsaas (Stavanger Helseforskning AS, Stavanger); Sigrun Halvorsen (Oslo Universitetssykehus HF, Oslo); Ulf Hurtig (Sykehuset Innlandet HF, Tynset).

**Peru (208 patients enrolled)** Roger M Correa Flores (Hospital Nacional IV Alberto Sabogal Sologuren, Callao); Jorge R Calderon Ticona (Novocardio Centro de Investigacion y Atencion Cardiovascular, Lima); Julio R Durand Velasquez (Centro de Invesgaciones Médicas - Hospital Maria Auxiliadora, Lima); Sandra A Negron Miguel (Clínica Internacional Sede San Borja, Lima); Enrique S Sanabria Perez (Clinica Javier Prado, Lima); Jesus M Carrion Chambilla (Hospital Nacional Arzobispo Loayza, Lima); Carlos A Chavez Ayala (Hospital Nacional Daniel Alcides Carrión, Callao); Reynaldo P Castillo Leon (Clínica San Gabriel - Consultorio 316, Lima); Rolando J Vargas Gonzales (Clínica Virgen María Auxiliadora, Piura); Jose D Hernandez Zuniga (Clinica Medica San Martin E.I.R.L, Ica); Luis A Camacho Cosavalente (Clínica Peruano Americana, Trujillo); Jorge E Bravo Mannucci (Clínica Divino Niño Jesus Orden de Malta,); Javier Heredia Landeo (Clinica Angloamericana, Clinica Angloamericana, Lima); Nassip C Llerena Navarro (Hospital Nacional Carlos Alberto Seguín Escobedo, Arequipa); Yudy M Roldan Concha (Hospital Nacional Hipolito Unanue, Lima); Víctor E Rodriguez Chavez (Hospital Central de la Fuerza Aerea Peruana, Lima); Henry A Anchante Hernandez (Hospital Nacional Cayetano Heredia, Lima); Carlos A Zea Nunez (Hospital Nivel IV Adolfo Guevara Velasco, Cuzco); Walter Mogrovejo Ramos (Instituto Neuro Cardiovascular de las Americas, Lima).

**Philippines (116 patients enrolled)** Arthur Ferrolino (Philippine Heart Center, Quezon City); Rosa Allyn G Sy (Ospital ng Makati Diabetes Clinic, Makati City); Louie Tirador (St. Paul's Hospital, Iloilo City); Rody G. Sy (Cardinal Santos Medical Center, San Juan); Generoso Matiga (Perpetual Succour Hospital/Cebu Heart Institute, Cebu City); Raul Martin Coching (Davao Doctors Hospital, Davao); Alisa Bernan (Community Health and Development Cooperative Hospital., Davao City); Gregorio Rogelio (St. Luke's Medical Center, Quezon); Dante D. Morales (Manila Doctors Hospital, Manila); Edgar Tan (Cebu Doctors University Hospital, Cebu City); Dennis Jose Sulit (Quirino Memorial Medical Center, Quezon City).

**Poland (926 patients enrolled)** Adrian Wlodarczak (Miedziowe Centrum Zdrowia, Lubin,); Krystyna Jaworska, Grzegorz Skonieczny (Wojewodzki Szpital Zespolony im. L. Rydygiera, Torun,); Lidia Pawlowicz (Prywatne Centrum Kardiologii, Torun); Pawel Wojewoda (Poradnia Kardiologiczna Centrum Medyczne Ogrodowa, Skierniewice,); Benita Busz-Papiez (Indywidualna Specjalistyczna Praktyka Lekarska, Szczecin); Janusz Bednarski (Samodzielny Pub. Spec. Szp. Zachodni im. Jana Pawla II, Grodzisk Mazowiecki); Aleksander Goch (10 Wojskowy Szpital Kliniczny z Poliklinika, Bydgoszcz); Pawel Staneta (NZOZ Specjalistyczna Przychodnia Lekarska „Medikard”, Plock); Elzbieta Dulak (NZOZ "Srodmiescie" Sp. z o.o., Gdynia); Andrzej Budaj (Centre of Postgraduate Medical Education, Grochowski Hospital, Warsaw, Poland); Krzysztof Saminski (Centrum Kardiologiczne "Pro Corde" Sp. z o.o., Wroclaw); Wlodzimierz Krasowski (COPERNICUS Podmiot Leczniczy Sp. z o.o. Szpital Sw. Wojciecha Gdansk); Wanda Sudnik (Centrum Medyczne Dr Sudnik s.c. W&J Sudnik, Sokolka); Aleksander Zurakowski (Malopolskie Centrum Sercowo-Naczyniowe, Chrzanow); Marcin Skorski (SPZOZ w Lecznej, Leczna); Roman Lysek (Przychodnia Lecznice CITOMED Sp. z o.o., Torun); Beata Miklaszewicz (B.Miklaszewicz & D.Dabrowski "CARDIAMED" Spolka Jawna, Legnica); Jacek Kubica (Szpital Uniwersytecki im. dr A. Jurasza, Bydgoszcz); Jan Andrzej Lipko (5 Wojskowy Szpital Kliniczny z Poliklinika SPZOZ, Krakow); Edyta Kostarska-Srokosz (Centrum Medyczne „Nasze Zdrowie”, Warszawa); Marek Piepiorka (Gabinet Kardiologiczno-Internistyczny, Gdynia); Anna Drzewiecka (NZOZ "Centrum-Med" s.c., Czeladz); Ryszard Sciborski (SPZOZ, Olawa); Arkadiusz Stasiewski (NZOZ Neuro-Kard Ilkowski i Partnerzy, Poznan); Tomasz Blicharski (Lubelskie Centrum Diagnostyczne, Swidnik); Leszek Bystryk (Wojewodzkie Centrum Szpitalne Kotliny Jeleniogorskiej, Jelenia Gora); Michal Szpajer (Poliklinika evi-, Gdynia); Marek Korol (Osrodek Medycyny Rodzinnej Sp. z o.o., Sobotka); Tomasz Czerski (SPZOZ w Wegrowie, Wegrów); Ewa Mirek-Bryniarska (Szpital Specjalistyczny im. J. Dietla, Krakow); Jacek Gniot (SPZOZ w Pulawach, Pulawy); Andrzej Lubinski (Indywidualna Specjalistyczna Praktyka Lekarska A. Lubinski, Lodz); Jerzy Gorny (Wojewodzki Szpital Specjalistyczny, Olsztyn); Edward Franek (Clinical Research Group Sp. z o.o., Warszawa); Grzegorz Raczak (Centrum Kardiologii Uniwersyteckie Centrum Kliniczne, Gdansk); Hanna Szwed (Instytut Kardiologii, Klinika Choroby Wiencowej, Warsaw).

**Portugal (174 patients enrolled)** Pedro Monteiro (Centro Hosp e Univ de Coimbra - Hosp da Univ de Coimbra, Coimbra); Jose Mesquita Bastos (Centro Hospitalar de Baixo Vouga, Aveiro); Helder H Pereira (Hospital Garcia De Orta, Almada); Dinis Martins (Hospital Do Divino Espírito Santo, Ponta Delgada); Joao Morais (Leiria Hospital Center, Leiria; ciTechCare, Polytechnic of Leiria, Leiria); Filipe Seixo (Centro Hospitalar De Setubal EPE, Setubal); Carlos Mendonça (Hospital de Vila Franca de Xira, Vila Franca de Xira); Ana Botelho and Francisca Caetano (Centro Hosp e Univ de Coimbra - Hosp da Univ de Coimbra, Coimbra).

**Romania (145 patients enrolled)** Bogdan Minescu (Spitalul Judetean De Urgenta Braila, Braila); Octavian Istratoaie (Spitalul Clinic Judetean De Urgenta, Craiova); Dan N Tesloianu (S.C. CARDIOMED SRL, Iasi); Maria Dorobantu (Spitalul Clinic De Urgenta Bucuresti, Bucuresti); Gabriel Cristian and Silviu Dumitrescu (SCUMC "dr. Carol Davila", Bucharest); Cristian GC Podoleanu (Podoleanu G. Cristian Gheorghe Calin, Targu Mures); Mircea CA Constantinescu (S.C. Medcon S.R.L., Buzau); Cristina M Bengus (Spitalul Judetean de Urgenta ”Sf. Pantelimon” Focsani, Focsani); Constantin Militaru (Cardiomed SRL, Craiova); Doina Rosu (Spitalul Clinic Judetean de Urgenta "Pius Branzeu", Timisoara); Irinel R Parepa (Spitalul Clinic Judetean de Urgenta Constanta, Constanta); Adrian V Matei (Institutul de Urgenta pentru Boli Cardiovasculare si Transpl, Targu Mures); Tom M Alexandru and Mihaela Malis (Impatients SRL, Codlea); Ioan Coman (Alcor Med Srl, Bucuresti); Rodica Stanescu-Cioranu (Clinica Angiomed SRL, Bucuresti); Doina Dimulescu (Spitalul Clinic De Urgenta "elias", Bucuresti).

**Russian Federation (1109 patients enrolled)** Yury Shvarts (Faculty Therapy Department of Clinical Hospital n.a. Mirotvo, Saratov); Olga Orlikova (Regional Clinical Cardiology Dispensary, Saratov); Zhanna Kobalava (City Clinical Hospital #64, Moscow); Olga L Barbarash (Scientific & Research Institute of Complex Problems of Cardi, Kemerovo); Valentin Markov (Research Institute Of Cardiology, Tomsk); Nadezhda Lyamina (Saratov Scientific Research Institute Of Cardiology, Saratov); Alexander Gordienko (Medical Military Academy n.a.S.M.Kirov, Saint - Petersburg); Konstantin Zrazhevsky (City Hospital #38 named after Semashko N.A, Saint Petersburg); Alexander Y Vishnevsky (Pokrovskaya City Hospital, St-Petersburg); Victor Gurevich (Central Medical Unit #122, St-Petersburg); Raisa Stryuk (City Clinical Hospital No. 71, Moscow); Nikita V Lomakin (Central Clinical Hospital with Polyclinic, Moscow); Igor Bokarev and Tatiana Khlevchuk (City Clinical Hospital n.a. Eramishantsev, Moscow); Sergey Shalaev (State Autonomous Institution of Healthcare of Tyumen region, Tyumen); Larisa Khaisheva (City Emergency Hospital #2, Rostov-On-Don); Petr Chizhov (Therapeutic Department of Clinical Hospital n.a. N.A. Semash, Yaroslavl); Inna Viktorova (Omsk City Clinical Hospital 1, Omsk); Natalya Osokina and Vladimir Shchekotov (State Budgetary Healthcare Institution Clinical Medical Sani, Perm); Evgenia Akatova (City Clinical Hospital#40of the Department of Healthcare of, Moscow); Galina Chumakova (Altay Regional Cardiology Dispensary, Barnaul); Igor Libov (City Clinical Hospital n.a.Botkin ofMoscow Healthcare depart, Moscow); Mikhail I Voevoda (Institute of Therapy and preventive medicine, Novosibirsk); Tatyana V Tretyakova (City Clinical Hospital #25, Novosibirsk); Evgeny Baranov (City Clin. Hosp. #5 of Nizhni Novgorod district, Nizhny Novgorod); Sergey Shustov (Medical Military Academy n.a.S.M.Kirov, Saint - Petersburg); Sergey Yakushin (Ryazan Regional Clinical Cardiology Dispensary, Ryazan); Ivan Gordeev (City Clinical Hospital #15 n.a. Filatov, Moscow); Niiaz Khasanov (State Med. Uni. based on MSHI City Clinical Hospital #7, Kazan); Olga Reshetko (Regional Clinical Hospital, Saratov); Tatiana Sotnikova (City Clinical Hospital n.a.Botkin ofMoscow Healthcare depart, Moscow); Olga Molchanova (National Research Center For Preventive Medicine, Moscow); Konstantin Nikolaev (City Clinical Hospital #19, Novosibirsk); Liudmila Gapon (Tyumen Cardiology Center, Tyumen); Elena Baranova (St. Petersburg State Med. Univ. named for I. P. Pavlov, Saint-Petersburg); Zaur Shogenov (City Clinical Hospital n.a. Veresaev, Moscow); Elena Kosmachova (Regional Clinical Hospital #1, Krasnodar); Yuriy Karpov (Voronezh City Clin. Hosp. of Emergency Medical Care #1, Voronezh); Yuri Karpov (National Medical Research Centre of Cardiology, Moscow); Anton Povzun (Saint-Petersburg I.I. Dzhanelidze Research Institute of Emer, Saint-Petersburg); Liudmila Egorova (Saint Luke's Clinical Hospital, Saint Petersburg); Vadim V Tyrenko (Military Medical Academy n.a. Kirov, St-Petersburg); Igor G Ivanov and Masterov Ilya (St. George the Martyr City Hospital, Saint-Petersburg); Sergey Kanorsky (Krasnodar City Clinical Urgent Care Hospital, Krasnodar).

**Serbia (255 patients enrolled)** Dragan Simic (Clinical Centre of Serbia, Belgrade); Nikola Ivanovic (General Hospital Sremska Mitrovica, Sremska Mitrovica); Goran Davidovic (Clinical Centre Kragujevac, Kragujevac); Nebojsa Tasic (Institute for Cardiovascular Diseases Dedinje, Belgrade); Milika R. Asanin (Clinical Centre of Serbia, Belgrade); Stevo Stojic (General Hospital Pancevo, Pancevo); Svetlana R. Apostolovic (Clinical Center Nis, Nis); Stevan Ilic (Institute for treatment and rehabilitation “Niska Banja”, Nis); Biljana Putnikovic Tosic (Clinical Hospital Centre Zemun, Belgrade); Aleksandar Stankovic (General Hospital Leskovac, Leskovac); Aleksandra Arandjelovic (Clinical Hospital Centre Zvezdara, Belgrade); Slavica Radovanovic and Branislava Todic (Clinical-Hospital Center Bezanijska Kosa, Belgrade); Arsen D. Ristic (Clinical Centre of Serbia, Belgrade); Jovan Balinovac (General Hospital Valjevo, Valjevo); Dragan V. Dincic (Military Medical Academy, Belgrade); Petar Seferovic and Ana Karadzic (Clinical Center of Serbia, Belgrade); Slobodan Dodic (Institute of Cardiovascular Diseases of Vojvodina, Sremska Kamenica); Sinisa Dimkovic and Tamara Jakimov (Clinical Hospital Center Bezanijska Kosa, Belgrade).

**Singapore (49 patients enrolled)** Terrance Chua (National Heart Centre, Singapore); Kian-Keong Poh (National University Hospital, Singapore); Hean Yee Ong and Justin Tang I-Shing (Khoo Teck Puat Hospital, Singapore).

**Slovakia (340 patients enrolled)** Karol Micko (Interna a kardiologicka klinika, Kardiomed s.r.o., Lucenec); Jan Nociar (Interna a kardiologicka ambulancia KARDIO1, s.r.o., Lucenec); Daniel Pella (Cardio D&R s.r.o., Kosice); Peter Fulop (Kardiologicka a interna ambulancia Interna SK, s.r.o., Svidnik); Marian Hranai (Kardiologicka ambulancia, Nitra); Juraj Palka (Kardiologicka a interna ambulancia CORP, s.r.o., Kosice); Juraj Mazur (Kardiologicka a interna ambulancia Kardio-onkologia, s.r.o., Dolny Kubin); Ivan Majercák (Sukromna interna a kardiologicka ambulancia, Kosice); Andrej Dzupina (Alian, s.r.o., Bardejov); František Fazekas (Kardiologicka ambulancia Medicentrum Heart, s.r.o., Kralovsky Chlmec); Jozef Gonsorcik (Nestatna interna a kardiologicka ambulancia, Kosice); Viliam Bugan (Medivasa s.r.o., Zilina); Jan Murin (I. Interna klinika, Univerzitna nemocnica Bratislava, Bratislava); Juraj Selecky (Sciens, s.r.o., Trebisov); Gabriel Kamensky (V. Interna klinika Univerzitna nemocnica Bratislava Ruzinov, Bratislava); Jaroslava Strbova (Kardiologicka, interna a sonograficka amb. Kardio-Sanus, sro, Bratislava); Rudolf Smik (Interna, kardiologicka, angiologicka ambul. Medikard, s.r.o., Presov); Andrej Dukat (Univerzitna nemocnica Bratislava, Bratislava); Peter Olexa (Topcare s.r.o, Kosice).

**Slovenia (36 patients enrolled)** Ivan Žuran (General Hospital Celje, Celje); Janez Poklukar (General Hospital Jesenice, Jesenice); Nataša Černič Šuligoj (General Hospital Izola, Izola); Matija Cevc (Department of Vascular Medicine, Division of Medicine, University Medical Centre Ljubljana, Ljubljana); Zlatko Fras (Preventive Cardiology Unit, Department of Vascular Medicine, Division of Medicine, University Medical Centre Ljubljana, Ljubljana; Faculty of Medicine, University of Ljubljana, Ljubljana).

**Republic of South Africa (505 patients enrolled)** Henry P Cyster (TREAD Research CC, Parow); Naresh Ranjith (Nash Ranjith Research Centre, Durban); Clive Corbett (Panorma Medi-Clinic, Western Cape); Junaid Bayat (Suite 502, Durban Medical Centre, Durban); Ellen Makoali Makotoko and Hendrik du Toit Theron (Cardiology Research, Bloemfontein); Ilse E Kapp (Cardiology Clinical Research, Alberton); Matthys M de V Basson (Tiervlei Trial Centre, Bellville); Hanlie Lottering and Dina Van Aswegen (SCION Clinical Research, Pretoria); Louis J Van Zyl (Clinical Projects Research SA (Pty) Ltd, Worcester); Peter J Sebastian (Dr PJ Sebastian Private Practice, Chatsworth); Thayabran Pillay (Vincent Palotti Hospital, Cape Town); Jan A Saaiman (Kuils River Private Hospital, Kuilsrivier, Western Cape); Patrick J Commerford (Groote Schuur Hospital, Cape Town); Soraya Cassimjee and Garda Riaz (Dr Garda Cardiology Practice, Johannesburg); Iftikhar O Ebrahim (Unitas Hospital, Pretoria); Mahomed Sarvan (Drs MI Sarvan, R Moodley & Partners incorporated, Tongaat); Joseph H Mynhardt (Dr. J Mynhardt, Private Practice, Kimberley); Anthony J Dalby (Life Fourways Hospital, Randburg [previously at Milpark Hospital, Johannesburg]); Helmuth Reuter (Winelands Medical Research Centre, Stellenbosch); Rajendran Moodley (Netcare Umhlanga Medical Centre, Durban).

**Spain (826 patients enrolled)** Manuel Vida (Hospital Virgen del Mar, Almería, Almería); Angel R. Cequier Fillat (Hospital Universitari de Bellvitge, Hospitalet de Llobregat, Barcelona); Vicente Bodí Peris (Hospital Clínico Universitario, Valencia, Valencia); Francisco Fuentes Jimenez (Hospital Universitario Reina Sofía, Córdoba, Córdoba); Francisco Marín (Hospital Virgen De La Arrixaca, Murcia, Murcia); Jose M Cruz Fernández and Rafael Jesus Hidalgo Urbano (Hospital Universitario Virgen Macarena, Sevilla, Sevilla); Blas Gil-Extremera and Pablo Toledo (Complejo Hospitalario Universitario de Granada, Granada); Fernando Worner Diz (Hospital Universitari Arnau de Vilanova, Lleida, Lérida); David Garcia-Dorado (Hospital Vall d'Hebron, Barcelona, Barcelona); Andres Iñiguez (Hospital Alvaro Cunqueiro, Vigo); José Tuñón Fernández (Fundación Jiménez Díaz, Madrid, Madrid); Jose R Gonzalez-Juanatey (Hospital Clínico Universitario de Santiago, Santiago de Compostela); Javier Fernandez Portales (Hospital San Pedro de Alcántara, Cáceres, Cáceres); Fernando Civeira Murillo (Hospital Miguel Servet, Zaragoza, Zaragoza); Laia Matas Pericas (Hospital de la Santa Creu i Sant Pau, Barcelona, Barcelona); Jose Luis Zamorano (Hospital Universitario Ramon y Cajal, Madrid, Madrid); Manuel De Mora Martin (Hospital Carlos Haya, Málaga, Málaga); Jordi Bruguera Cortada (Hospital Del Mar, Barcelona, Barcelona); Joaquin J Alonso Martin and Jose Maria Serrano Antolin (Hospital Universitario De Fuenlabrada, Fuenlabrada); José R De Berrazueta Fernández and José Antonio Vázquez de Prada (Hospital Universitario Marques De Valdecilla, Santander, Santander); Jose Francisco Díaz Fernández (Hospital Juan Ramón Jiménez, Huelva, Huelva); José Alberto García Lledó (Hospital Príncipe de Asturias, Alcalá de Henares, Madrid); Juan Cosín Sales (Hospital Arnau De Vilanova, Valencia, Valencia); Javier Botas Rodriguez (Hospital Universitario Fundación Alcorcón, Madrid, Madrid); Gabriel Gusi Tragant (Hospital de Sabadell - Corporacio Sanitaria Parc Tauli, Sabadell, Barcelona); Amparo Benedicto (Hospital Universitario la Princesa, Madrid, Madrid); Carlos Gonzalez-Juanatey (Hospital Universitario Lucus Augusti, Lugo, Lugo); Mercedes Camprubí Potau (Hospital Joan XXIII, Tarragona, Tarragona); Ignacio Plaza Perez (Hospital Universitario Infanta Sofia, San Sebastian de los Reyes); César Morís De La Tassa (Hospital Universitario Central de Asturias, Oviedo, Asturias); Pablo Loma-Osorio Rincon (Hospital Universitari de Girona Dr. Josep Trueta, Girona); Javier Balaguer Recena (Hospital General Universitario de Guadalajara, Guadalajara, Guadalajara); Juan M Escudier (Hospital Universitario Puerta De Hierro Majadahonda, Majadahonda); Antonio Coca Payeras (Hospital Clinic i Provincial, Barcelona); Norberto Alonso Orcajo (Complejo asistencial de Leon, Leon); Pedro Valdivielso (Hospital Virgen de la Victoria, Malaga).

**Sri Lanka (314 patients enrolled)** Godwin Constantine (University of Colombo, Colombo); Ruvaiz Haniffa (University of Colombo, Colombo); Nirmali Tissera (National Hospital of Sri Lanka, Colombo); Stanley Amarasekera and Chandrike Ponnamperuma (Colombo South Teaching Hospital, Kalubowila); Nimali Fernando and Kaputella Fernando (North Colombo Teaching Hospital, Ragama); Jayanthimala Jayawardena (Institute of Cardiology, National Hospital of Sri Lanka, Colombo); Santharaj Wijeyasingam and Gotabhaya Ranasinghe (Institute of Cardiology, National Hospital of Sri Lanka, Colombo); Ruvan Ekanayaka and Sepalika Mendis (Institute of Cardiology, National Hospital of Sri Lanka, Colombo); Vajira Senaratne (Institute of Cardiology, National Hospital of Sri Lanka, Colombo ); Gnanamoorthy Mayurathan (Kandy Teaching Hospital, Kandy); Ajantha Rajapaksha and Thilak Sirisena (Kurunegala Teaching Hospital, Kurunegala); Jagath I Herath (Sri Jayawardenapura General Hospital, Nugegoda); Naomali Amarasena (Sri Jayawardenapura General Hospital, Nugegoda).

**Sweden (250 patients enrolled)** Stefan Berglund (Falu Lasarett, Falun); Gundars Rasmanis (Karolinska University Hospital Huddinge, Stockholm); Emil Hagström and Ola Vedin (Dept of Medical Sciences, and Uppsala Clinical Research Centre, Uppsala University, Uppsala); Nils Witt (Södersjukhuset, Stockholm); Georgios Mourtzinis (Sahlgrenska University Hospital, Mölndal); Peter Nicol (Närsjukhuset Köping, Köping); Ole Hansen (Skåne University Hospital, Malmö); Stefano Romeo (Sahlgrenska University Hospital, Göteborg); Steen Agergaard Jensen (Blekingesjukhuset Karlshamn, Karlshamn); Ingemar Torstensson (Centralsjukhuset Kristianstad, Kristianstad); Ulf Ahremark (Hallands sjukhus Halmstad, Halmstad); Torbjörn Sundelin (Länssjukhuset Sundsvall-Härnösand, Sundsvall).

**Switzerland (88 patients enrolled)** Tiziano Moccetti (Cardiocentro Ticino, Lugano); Christian Müller (Cardiovascular Research Institute Basel (CRIB) and Department of Cardiology, University Hospital Basel, University of Basel, Basel); Francois Mach (Hopitaux Universitaires de Geneve, Geneve); Ronald Binde, Ulf Landmesser and Oliver Gämperli (Universitätsspital Zürich, Zürich).

**Taiwan (93 patients enrolled)** Chern-En Chiang (Taipei Veterans General Hospital, Taipei); Wei-Chuan Tsai (National Cheng Kung University Hospital, Tainan); Kwo-Chang Ueng (Chung-Shan Medical University Hospital, Taichung); Wen-Ter Lai (Kaohsiung Medical University Chung-Ho Memorial Hospital, Kaohsiung); Ming-En Liu (HsinChu Mackay Memorial Hospital, Hsinchu); Juey-Jen Hwang (National Taiwan University Hospital, Taipei); Wei-Hsian Yin (Cheng-Hsin General Hospital, Taipei); I-Chang Hsieh and Ming-Jer Hsieh (Chang Gung Memorial Hospital - Linkou, Taoyuan Hsien); Wei Hsiang Lin (Tri-Service General Hospital - Taipei, Taipei); Jen-Yuan Kuo (Mackay Memorial Hospital, Taipei); Tsuei-Yuan Huang (Chi-Mei Medical Center, Tainan); Chih-Yuan Fang (Kaohsiung Chang Gung Memorial Hospital, Kaohsiung).

**Thailand (161 patients enrolled)** Pinij Kaewsuwanna (Maharat Nakhonratchasima Hospital, Moung); Wasant Soonfuang (Police General Hospital, Pratumwan); Woravut Jintapakorn (Songklanagarind Hospital, Hat Yai); Apichard Sukonthasarn (Chiang Mai University, Muang, Chiang Mai); Piyamitr Sritara (Ramathibodi Hospital, Bangkok); Nattawut Wongpraparut (Siriraj Hospital, Mahidol University, Bangkok-Noi); Krisada Sastravaha (Bhumibol Adulyadej Hospital, Bangkok); Nakarin Sansanayudh (Phramongkutklao Hospital, Bangkok); Wirash Kehasukcharoen (Central Chest Institute, Nonthaburi); Dilok Piyayotai (Thammasat University Hospital, Klong Luang); Paiboon Chotnoparatpat (Vajira Bangkok University Hospital, Bangkok).

**Turkey (78 patients enrolled)** Ahmet Camsari (Mersin Universty School Of Medicine Department Of Cardiology, Mersin); Hakan Kultursay (Ege University Medical Faculty Department of Cardiyology, IZMIR); Sema Guneri (Dokuz Eylul Universitesi Tip Fakultesi, Izmir); Bulent Mutlu (Marmara Universitesi Pendik Egitim ve Arastirma Hastanesi, Istanbul); Murat Ersanli (Istanbul Universitesi Kardiyoloji Enstitusu, Istanbul); Mustafa Demirtas (Cukurova Universitesi Tip Fakultesi, Adana); Cevat Kirma (Kartal Kosuyolu Yuksek Ihtisas Egitim ve Arastirma Hastanesi, Istanbul); Ertan Ural (Kocaeli Universitesi Tip Fakultesi, Kocaeli); Lale Koldas (Istanbul Universitesi Cerrahpasa Tip Fakultesi, Istanbul).

**Ukraine (639 patients enrolled)** Oleksandr Karpenko (City Clinical Hospital No 1, Kiev); Alexander Prokhorov (City Clinical Hospital #27, Kharkiv); Ihor Vakaluyk (Ivano-Frankivsk Regional Clinical Cardiology Center, Ivano-Frankivsk); Halyna Myshanych (Railway Clinical Hospital #2, "Health Center", Kyiv); Dmytro Reshotko (Kyiv City Oleksandrivska Clinical Hospital, Kyiv); Valeriy Batushkin (Kyiv City Clinical Hospital #5, Cardiology Department, Kyiv); Leonid Rudenko (Kyiv Emergency Care Hospital, Kiev); Ihor Kovalskyi (City Hospital #1, Mykolaiv); Mykola Kushnir (Zhytomyr regional clinical hospital n.a. O.F. Gerbachevskyy, Zhytomyr); Vira Tseluyko (City Clinical Hospital #8, Kharkiv); Yuriy Mostovoy (Vinnitsya City Clinical Hospital 1, Vinnitsya); Mykola Stanislavchuk (Vinnytsia M.I. Pyrohov Regional Clinical Hospital, Vinnytsia); Yulian Kyiak (Lviv Danylo Halytskyi National Medical University, Lviv); Yuriy Karpenko (Odesa Regional Clinical Hospital, Odesa); Yaroslav Malynovsky (Regional Medical Center for Cardiovascular Diseases, Zaporizhia); Andriy Klantsa (Khmelnytskyi Regional Hospital, Khmelnytskyi); Oles Kutniy (Public Institution: Central City Hospital #2, Zhytomyr); Ekaterina Amosova (Kyiv City Oleksandrivska Clinical Hospital, Kyiv); Viktor Tashchuk (Chernivtsi Regional Clinical Cardiology Center, Chernivtsi); Oleh Leshchuk (Lviv Regional Clinical Hospital, Dept. of Internal Medicine, Lviv); Alexander Parkhomenko (Institute of Cardiology, Kyiv, Ukraine); Mykola Rishko (Zakarpattya Regional cardiology Dispensary, Uzhgorod); Mykola Kopytsya (State Institution LT Malaya Inst. of Ther. of AMS of Ukraine, kharkiv); Andriy Yagensky (Lutsk City Clinical Hospital, Lutsk); Mykola Vatutin (Donetsk National Medical University named after M. Gorkiy, Donetsk); Andriy Bagriy (Donetsk National Medical University named after M. Gorkiy, Donetsk); Olga M Barna (Policlinic ofadm of med services and rehabilitation of ARTEM, Kyiv); Olexiy Ushakov (City Clinical Hospital #6 of Emergency Medical Care, Simferopol); Georgiy Dzyak (Dnipropetrovsk Regional Clinical Center of Cardiology, Dnipropetrovsk); Borys Goloborodko (City Clinical Hospital # 3, Odessa); Anatolii Rudenko (M.M. Amosov National Institute of Cardiovascular Surgery, Kyiv); Volodymyr Zheleznyy (Sumy City Clinical Hospital #1, Sumy).

**United Kingdom (292 patients enrolled)** Jasper Trevelyan (Worcestershire Royal Hospital, Worcester, Worcestershire); Azfar Zaman (Freeman Hospital, Newcastle Upon Tyne, Tyne and Wear); Kaeng Lee (Birmingham Heartlands Hospital, Birmingham); Andrew Moriarty (Craigavon Area Hospital, Portadown, Armagh); Rajesh K Aggarwal (Basildon and Thurrock University Hospitals, Basildon, Essex); Piers Clifford (Wycombe General Hospital, High Wycombe, Buckinghamshire); Yuk-Ki Wong (St Richard's Hospital, Chichester, West Sussex); Syed MR Iqbal (Surrey and Sussex Healthcare NHS Trust, Redhill); Eduardas Subkovas (Glan Clwyd Hospital, Rhyl, Denbighshire); Denise Braganza (Peterborough and Stamford Hospitals NHS Foundation Trust, Peterborough); David Sarkar (Plymouth Hospitals NHS Trust, Plymouth, Devon); Robert Storey (University of Sheffield, Sheffield, South Yorkshire); Huw Griffiths (Queen Alexandra Hospital, Portsmouth, Hampshire); Sam Mcclure (City Hospitals Sunderland NHS Foundation Trust, Sunderland); Rangasamy Muthusamy and Simon Smith (The Rotherham NHS Foundation Trust, Rotherham, South Yorkshire); John Kurian (Bradford Teaching Hospitals NHS Foundation Trust, Bradford); Terry Levy (Royal Bournemouth Hospital, Bournemouth, Dorset); Craig Barr (The Dudley Group of Hospitals NHS Foundation Trust, Dudley, West Midlands); Honer Kadr (Queen's Hospital, Romford, Essex); Robert Gerber (Conquest Hospital, St Leonards, East Sussex); Audrius Simaitis (Royal Cornwall Hospitals NHS Trust, Truro, Cornwall); Handrean Soran (St Marys Hospital, Manchester, Greater Manchester); Anthony Mathur (London Chest Hospital, London); Adrian Brodison, Mohammad Ayaz and Muhammad Cheema (University Hospitals of Morecambe Bay NHS Foundation Trust, Cumbria, Cumbria); Richard Oliver and Simon Thackray (Castle Hill Hospital Hull And East Yorkshire Trust, Hull, East Yorkshire); Telal Mudawi, Gohar Rahman, and Ayyaz Sultan (Wrightington, Wigan and Leigh NHS Foundation Trust, Wigan, Greater Manchester); Timothy Reynolds (Queen's Hospital, Burton On Trent, Staffordshire); David Sharman and david Sprigings(Northampton General Hospital, Northampton, Northamptonshire); Rob Butler (University Hospital Of North Staffordshire, Stoke-On-Trent, Staffordshire); Peter Wilkinson (Ashford & St Peter's Hospitals NHS Foundation Trust, Chertsey); Gregory YH Lip (City Hospital, Birmingham, West Midlands); Julian Halcox, Sean Gallagher and Nicholas Ossei-Gerning(University Hospital Of Wales, Cardiff, South Glamorgan).

**United States (2511 patients enrolled)** Gil Vardi (St. Louis Heart and Vascular, St. Louis, Missouri); Duccio Baldari (Cardiology Partners Clinical Research Institute, Wellington, Florida); David Brabham (PharmaTex Research, Amarillo, Texas); Charles Treasure II (Cardiovascular Research of Knoxville, Knoxville); Charles Dahl (Aspen Clinical Research, Orem, Utah); Bruce Palmer (Wichita Falls Heart Clinic, Wichita Falls, Texas); Alan Wiseman (EMMC Northeast Cardiology Associates, Bangor); Abul Khan (The Iowa Clinic, West Des Moines, Iowa); Sanjeev Puri (Quad City Heart Center, Moline, Illinois); Ann Elizabeth Mohart (Mercy Health Research, Washington, Missouri); Carlos Ince (Maryland Cardiovascular Specialists, Baltimore, Maryland); Enrique Flores (Georgia Heart Specialists, LLC, Covington, Georgia); Scott Wright (Cardiovascular Associates of East Texas, Tyler, Texas); Shi-Chi Cheng (The Heart Center, PC, Huntsville, Alabama); Michael Rosenberg (Heart and Vascular Center/Holy Family Memorial, Manitowoc, Wisconsin); William Rogers, Jr (University of Alabama at Birmingham Medical Center, Birmingham); Edward Kosinski (Connecticut Clinical Research, LLC, Bridgeport); Les Forgosh (HealthEast Medical Research Institute, St. Paul); Jonathan Waltman (Saint Joseph's Healthcare Research Center, Lexington, Kentucky); Misal Khan (Springfield Medical Center, Panama City); Mohammad Shoukfeh (Caprock Cardiac Center Research Institute, Lubbock); Georges Dagher and Patrick Cambier (Morton Plant Mease Health Care, Inc., Clearwater, Florida); Ira Lieber (TCR Institute, LLC, Kingwood, Texas); Priya Kumar (Advanced Research Associates, Greenwood, South Carolina); Cara East (Baylor University Medical Center, Dallas, Texas); Perry Krichmar and Mian Hasan (Research Physicians Network Alliance, Pembroke Pines, Florida); Lindsey White (Eastern Carolina Cardiovascular Associates, Elizabeth City, North Carolina); Thomas Knickelbine (Minneapolis Heart Institute Foundation, Minneapolis, Minnesota); Thomas Haldis (Sanford-Fargo, Fargo, North Dakota); Eve Gillespie and Thomas Amidon (Glacier View Cardiology PC, Kalispell, Montana); David Suh (Atlanta Heart Specialists LLC, Tucker); Imran Arif and mouhamad Abdallah (University of Cincinnati Hospital, Cincinnati); Faiq Akhter (Apex Medical Research, AMR Inc., Springfield, Ohio); Eric Carlson (Eastern Cardiology, P.A., Greenville, North Carolina); Michael D'Urso (Black Hills Cardiovascular Research, Rapid City, South Dakota); Fadi El-Ahdab (The Cardiovascular Group Centra, Lynchburg, Virginia); William Nelson and Katie Moriarty (Cardiology Research, St. Paul, Minnesota); Barry Harris (Integrative Research Associates Inc, Fort Lauderdale, Florida); Steven Cohen (The Cardiology Center, Delray Beach); Luther Carter and Daniel Doty (Cardiology Consultants, Pensacola); Kenneth Sabatino (Clearwater Cardiovascular Consultants, Safety Harbor, Florida); Tariq Haddad (Virginia Heart, Leesburg, Virginia); Amir Malik (Plaza Medical Center of Fort Worth, Fort Worth); Sunder Rao (Butler Medical Providers, Butler); Angel Mulkay (Mulkay Cardiology Consultants PC, Hackensack, New Jersey); Ion Jovin (McGuire V.A. Medical Center, Richmond, Virginia); Kim Klancke (Daytona Heart Group, Daytona Beach); Vinay Malhotra (MultiCare Institute for Research and Innovation, Puyallup); Sai K Devarapalli (IU Health Ball Memorial Hospital Physicians, Inc., Muncie, Indiana); Michael Koren (Jacksonville Center for Clinical Research, Jacksonville); Harish Chandna (Victoria Heart and Vascular Center, Victoria, Texas); George Dodds III, Tauqir Goraya and James Bengston (Michigan Heart, PC, Ypsilanti, Michigan); Matthew Janik (PMG Research of Wilmington, Wilmington, North Carolina); Joseph Moran (Piedmont Healthcare - Research, Statesville, North Carolina); Andrew Sumner (Lehigh Valley Hospital/Lehigh Valley Health Network, Allentown, Pennsylvania); John Kobayashi (Beacon Medical Group, South Bend, Indiana); William Davis (Advanced Cardiovascular, LLC, Auburn, Alabama); Shahram Yazdani (Inova Cardiology Ambulatory Research(ICARE-1), Manassas, Virginia); John Pasquini (Novant Health Heart and Vascular Institute, Charlotte, North Carolina); Maitreya Thakkar (PMG Research of Rocky Mount, LLC, Rocky Mount, North Carolina); Amarnath Vedere (Cardiology Partners Clinical Research Institute, Palm Beach Gardens); Wayne Leimbach (Oklahoma Heart Institute, Tulsa); James Rider and Sarah fenton (Cardiology Associates of Bellin Health, Green Bay); Narendra Singh (Atlanta Heart Specialists, Cumming); Anil V Shah (Coastal Multi-Specialty Research, Santa Ana, California); Patrick M. Moriarty (Clinical Pharmacology – University of Kansas Medical Center, Kansas City, Kansas); Denise Janosik (Saint John's Mercy Cardiovascular Research, St. Louis, Missouri); Carl Pepine (University of Florida, Gainesville, Florida); Brett Berman (Brett J. Berman, MD, APC, Chula Vista Cardiac Center, California); Joseph Gelormini (Trinity Medical WNY, PC, Buffalo, New York); Christopher Daniels and Kerensky Richard (John D. Archbold Memorial Hospital, Thomasville, Georgia); Friederike Keating (The University of Vermont - Fletcher Allen Health Care, Burlington, Vermont); Nicholas I Kondo (Saratoga Cardiology Associates, Saratoga Springs, New York); Sanjay Shetty and Howard Levite (AtlantiCare Regional Medical Center, Pomona); Winfried Waider (Long Beach Memorial Medical Center, Long Beach, California); Theodore Takata (Texas Health Research and Education Institute, Fort Worth, Texas); Mazen Abu-Fadel (University of Oklahoma Health Sciences Center, Oklahoma City); Vipul Shah (Carolina Heart Specialists, LLC, Lancaster, South Carolina); Rahul Aggarwal (Tenet Florida Physician Services, Jupiter); Mark Izzo (St. Vincent Consultants in Cardiovascular LLC, Erie, Pennsylvania); Anil Kumar (Kumar Medical Corporation, Lancaster, California); Brack Hattler and Rose Do (Denver V.A. Medical Center, Denver); Chad Link (Sparrow Clinical Research Institute, Lansing); Anna Bortnick (Montefiore Medical Center, Bronx, New York); George Kinzfogl III (Metrowest Medical Center, Framingham, Massachusetts); Arnold Ghitis (Heart and Health Institute Westside, Plantation, Florida); John Larry (Ohio State University Hospital East, Columbus, Ohio); Edward Teufel (Main Medical Partners Maine Health Cardiology, Scarborough, Maine); Peter Kuhlman (Saint Vincent's Cardiology, Jacksonville); Brent Mclaurin (AnMed Health Medical Center, Anderson); Wenwu Zhang (Cardiovascular Solutions, LLC, Shreveport, Louisiana); Stephen Thew (Heart Clinics Northwest, Spokane, Washington); Jalal Abbas (Clinical Research Institute of Arizona, Surprise); Matthew White, Othman Islam, and Sumeet Subherwal (Raleigh Cardiology Associates, P.A., Raleigh); Nandkishore Ranadive and Babak Vakili (Orlando Heart Specialists, Altamonte Springs); Christian Gring (NC Heart and Vascular Research, Clayton, North Carolina); David Henderson (Cardiology Research Associates, Daytona Beach); Timothy Schuchard (CentraCare Heart & Vas Center at St. Cloud Hospital, St. Cloud, Minnesota); Naim Farhat (North Ohio Research, Ltd., Elyria, Ohio); Geoffrey Kline (U of N Texas Health Science Ctr-Infectious Disease, Fort Worth, Texas); Sharan Mahal (Advanced Heart Care, LLC, Bridgewater, New Jersey); Jack Whitaker (Wellmont CVA Heart Institute, Greenville, Tennessee); Shawn Speirs (Eastern Idaho Regional Medical Center, Idaho Falls); Rolf Andersen (Lancaster Heart Foundation, Lancaster, Pennsylvania); Nizar Daboul (Advanced Medical Research, Maumee); Phillip Horwitz and Firas Zahr (University of Iowa, Iowa City); George Ponce (Spectrum Clinical Research Institute Inc, Moreno Valley, California); Zubair Jafar (Hudson Valley Heart Center, Poughkeepsie); Joseph Mcgarvey, Jr (Doylestown Cardiology Associates-VIAA, Doylestown, Pennsylvania); Vipul Panchal (Norton Cardiovascular Associates, Louisville, Kentucky); Stephen Voyce (Geisinger Cardiology-Scranton, Scranton, Pennsylvania); Thomas Blok (Advanced Cardiac Healthcare, Kalamazoo); William Sheldon (North Ohio Research Center - Sandusky, Sandusky, Ohio); Masoud M Azizad (Valley Clinical Trials, Inc., Northridge); Carsten Schmalfuss (Malcom Randall V.A. Medical Center, Gainesville); Mark Picone and Robert Pederson (Austin Heart, PLLC, Austin, Texas); William Herzog, Jr and Keith Friedman (CV Specialists of Central Maryland/Johns Hopkins University, Columbia, Maryland); Jason Lindsey (Saint Luke's Hospital, Kansas City, Missouri); Rosemary Nowins, Eichenlaub Timothy and Parilak Leonard (CIMA Medical Resarch Inc, Las Vegas, Nevada); Norman Lepor (Westside Medical Associates of Los Angeles, Beverly Hills); Mahfouz El Shahawy (Cardiovascular Center of Sarasota, Sarasota, Florida); Howard Weintraub (New York University Medical Center, New York); Anand Irimpen and Alvaro Alonso (Tulane University Medical Center, Dept. of Medicine, New Orleans); Wade May and Daniels Christopher (Cardiovascular Institute of the South, Lafayette, Louisiana); Thomas Galski (Virtua the Cardiology Group, Moorestown, New Jersey); Alan Chu (Amarillo Heart Clinical Research Institute, Inc., Amarillo, Texas); Freny Mody and Ebrahimi Ramin (V.A. Greater Los Angeles Health Care System at West LA, Los Angeles, California); Zachary Hodes and Joseph Rossi (The Care Group LLC, Indianapolis, Indiana); Gregory Rose (Wake Heart Research, Raleigh, North Carolina); James Fairlamb (Missouri Cardiovascular Specialists, Columbia, Missouri); Charles Lambert, Jr (Florida Hospital Pepin Heart Institute, Tampa, Florida); Ajit Raisinghani (University of California at San Diego, San Diego, California); Antonio Abbate and George Vetrovec (Virginia Commonwealth University Medical Center, Richmond); Marilyn King (Clearwater CV and Interventional Consultants- Bardmoor, Largo, Florida); Charles Carey (Saint Anthony's Medical Center, St. Louis, Missouri); Jaime Gerber (Yale New Haven Hospital Heart and Vascular Research, North Haven, Connecticut); Liwa Younis (Gateway Cardiovascular Research, St. Louis, Missouri); Hyeun (Tom) Park (Lakeland Cardiology, Mountain Lakes, New Jersey); Mladen Vidovich (University of Illinois at Chicago, Chicago, Illinois); Thomas Knutson (Prevea Clinic, Inc., Green Bay); Dennis Friedman (Cardiac Associates, PC, Rockville, Maryland); Fred Chaleff (Infinity Clinical Research LLC, Sunrise, Florida); Arthur Loussararian (St. Jude Hospital Yorba Linda DBA St. Joseph Heritage Health, Mission Viejo, California); Phillip Rozeman (Clinical Trials of America, Minden, Louisiana); Carey Kimmelstiel and Jeffrey Kuvin (Tufts-New England Medical Center, Boston); Kevin Silver (Summa Health, Akron, Ohio); Malcolm Foster (Turkey Creek Medical Center, Knoxville, Tennessee); Glen Tonnessen and Andrey Espinoza (Hunterdon Cardiovascular Associates, Flemington, New Jersey); Mohamadali Amlani (Mid-Michigan Cardiology Associates, Flint, Michigan); Andreas Wali (Holy Spirit Cardiology, Camp Hill, Pennsylvania); Christopher Malozzi, Geert T Jong and Clara Massey (University of South Alabama Medical Center, Mobile, Alabama); Keattiyoat Wattanakit (Heartcare Midwest, Peoria); Philip J. O'Donnell (Selma Medical Associates, Winchester); Dinesh Singal (Cardio Metabolic Institute, Somerset); Naseem Jaffrani (Alexandria Cardiology Clinic, Alexandria); Sridhar Banuru (The Heart Group, Newburgh); Daniel Fisher (Capitol Interventional Cardiology, Carmichael, California); Mark Xenakis (Cardiovascular Associates of Virginia, Midlothian, Virginia); Neal Perlmutter (Overlake Medical Clinics, Bellevue, Washington); Ravi Bhagwat (Cardiovascular Research of Northwest Indiana, Munster, Indiana); James Strader, Jr and Ronald Blonder (Colorado Health Medical Group CV and Thoracic Care, Colorado Springs, Colorado); Ayim Akyea-Djamson (Metropolitan Cardiovascular Consultants, Beltsville, Maryland); Ajay Labroo (Advanced CardioVascular Consultants, Rock Island); Kwan Lee (University of Arizona Medical Center, Tucson, Arizona); H. John Marais (Beaver Medical Clinic, Banning, California); Edmund Claxton, Jr, Robert Weiss and Rohr Kathryn (Maine Research Associates, Auburn); Martin Berk (CV Research Institute of Dallas, Inc., Dallas, Texas); Peter Rossi (Pasco Cardiology Center, Hudson); Parag Joshi and Amit Khera (UT Southwestern Medical Center, Dallas); Ajit S Khaira (Bala and Khaira Medical Research Center, Fresno); Greg Kumkumian (Maryland Heart, PC, Bethesda, Maryland); Steven Lupovitch (Northwest Heart Clinical Research, Arlington Heights, Illinois); Joshua Purow (Holy Cross Hospital Inc, Fort Lauderdale, Florida); Stephen Welka (Aurora Memorial Hospital Burlington, Burlington, Wisconsin); David Hoffman (St. Elizabeth Health Center, Youngstown, Ohio); Stuart Fischer (Los Alamitos Cardiovascular, Los Alamitos); Eugene Soroka (Saviers Medical Group, Port Hueneme, California); Donald Eagerton (Carolina Health Specialists, Myrtle Beach, South Carolina); Samir Pancholy (Northeast Clinical Trials Group, Scranton); Michael Ray and Norman Erenrich (Cardiology Associates of Palm Beach, West Palm Beach); Michael Farrar (Northland Cardiology, North Kansas City); Stewart Pollock (Harrisonburg Medical Association, Harrisonburg, Virginia); William J French (Harbor University of California Los Angeles Medical Center, Torrance, California); Steve Diamantis and Douglas Guy (Methodist Physicians Clinic – Heart Consultants, Omaha); Lawrence Gimple (University of Virginia Health System, Charlottesville, Virginia); Mark Neustel (St. Mary's/Duluth Clinic, Duluth, Minnesota); Steven Schwartz (Foundation Cardiology, Nashua, New Hampshire); Edward Pereira and Seals Albert (East Coast Institute for Research, Jacksonville); Douglas Spriggs (Clearwater Cardiovascular Consultants, Clearwater); Janet Strain and Suneet Mittal (Valley Health System, Ridgewood, New Jersey); Anthony Vo (Long Beach V.A. Healthcare System, Long Beach, California); Majed Chane (California Heart Specialists, Huntington Beach); Jason Hall (Medicore Cardiology, Bridgewater, New Jersey); Nampalli Vijay (Aurora Denver Cardiology Associates, PC, Denver); Kapildeo Lotun (University of Arizona Sarver Heart Center, Tucson, Arizona); F. Martin Lester (IMC Diagnostic and Medical Clinic, Mobile, Alabama); Ahed Nahhas (Toledo Clinic, Toledo); Theodore Pope (Midwest Heart and Vascular Specialists, Overland Park, Kansas); Paul Nager (Midwest Heart & Vascular Specialists, Independence, Missouri); Rakesh Vohra and Mukesh Sharma (Parkway Cardiology Associates, PC, Oak Ridge); Riyaz Bashir (Temple University Hospital, Philadelphia); Hinan Ahmed (The University of Texas Health Science Center at San Antonio, San Antonio, Texas); Michael Berlowitz (Tampa General Hospital, Tampa, Florida); Robert Fishberg (Associates in Cardiovascular Disease, LLC, Springfield, New Jersey); Robert Barrucco (Virtua Cardiology Group, Voorhees, New Jersey); Eric Yang (University of California at Los Angeles, Los Angeles, California); Michael Radin (Radin Cardiovascular Medical Group, Inc, Newport Beach); Daniel Sporn and Dwight Stapleton (Guthrie Clinic, Ltd., Sayre, Pennsylvania); Steven Eisenberg (Cardiovascular Specialists, PC, Atlanta, Georgia); Joel Landzberg (Westwood Cardiology, Westwood, New Jersey); Martin Mcgough (Academic Cardiology Associates, Rochester Hills, Michigan); Samir Turk (Trinity Medical Group, Minot); Michael Schwartz (DuPage Medical Group Cardiology, Winfield, Illinois); P. Sandy Sundram (Advanced Heart Group, Harvey); Diwakar Jain (Westchester Medical Center, Valhalla); Mark Zainea (McLaren Macomb, Mount Clemens, Michigan); Carlos Bayron (Interventional Cardiac Consultants, Trinity, Florida); Ronald Karlsberg and Suhail Dohad (Cardiovascular Research Foundation of Southern California, Beverly Hill, California); Henry Lui (Research Associates of Jackson, Jackson); William Keen (Kaiser Permanente Viewridge Medical Offices, San Diego, California); Donald Westerhausen, Jr (Midwest Cardiovascular Research, Elkhart, Indiana); Sandeep Khurana (Healthy Heart Cardiology, Grandville); Himanshu Agarwal (Allegent Health Heart and Vascular Specialsts, Omaha, Nebraska); Jessica Birchem (Mercy Medical Research Institute, Springfield); William Penny, Jr (V.A. San Diego Health Care System, San Diego, California); Mark Chang (Wellmont CVA Heart Institute, Johnson City); Sherrill Murphy and John Henry (Heart Consultants PC, Omaha, Nebraska); Branislav Schifferdecker (Oklahoma Heart Hospital, Oklahoma City, Oklahoma City);John M Gilbert (Saint Jude Heritage Medical Group, Fullerton, California); Gopal Chalavarya (Florida Cardiology Group, Hudson, Florida); Charles Eaton (Memorial Hospital of Rhode Island, Pawtucket, Rhode Island); John F Schmedtje, Jr (Roanoke Heart Institute, Roanoke); Stuart Christenson and Imran Dotani (McFarland Clinic PC, Ames, Iowa); Douglas Denham (Clinical Trials of Texas, Inc., San Antonio); Alexander Macdonell (University Cardiology Associates, Augusta, Georgia); Paul Gibson (Cardiology Research Associates, Saint Louis); Aref Rahman (V.A. Pittsburgh Healthcare System, Pittsburgh); Tammam Al Joundi and Nizar Assi (Gateway Cardiovascular Research Center, Jerseyville, Illinois); Gary Conrad (Huntington Memorial Hospital, Pasadena, California); Purushotham Kotha (Purushotham and Akther Kotha MD, Inc, La Mesa, California); Michael Love (Erlanger Health System, Chattanooga, Tennessee); Gregory Giesler (Huntington Hospital, Pasadena, California); Howard Rubenstein and Dawood Gamil (Port City Clinical Research, Saraland, Alabama); Laura Akright (Northeast Clinical Research of San Antonio, Schertz, Texas); Justine Krawczyk and Joanne Cobler (Buffalo Medical Group, Williamsville, New York); Terry Wells (Angelina Medical Research LLC, Lufkin, TX); James Welker (Anne Arundel Health System, Annapolis, MD); Robert Foster (Birmingham Heart Clinic, PC, Birmingham, AL); Richard Gilmore (Lake Charles Medical and Surgical Clinic, Lake Charles, LA); Jay Anderson (Nebraska Medicine - Internal Medical Associates, Grand Island, NE); Douglas Jacoby (Pennsylvania Presbyterian Medical Center, Philadelphia, PA); Bill Harris (Pikeville Medical Center, Pikeville, KY); Geraldine Gardner (Providence Everett Medical Center, Everett, WA); Ramprasad Dandillaya (Ram Dandillaya, M.D., Inc, Beverly Hills, CA); Kishor Vora (Research Integrity, Owensboro, KY); John Kostis (Rutgers University - Robert Wood Johnson Medical School, New Brunswick, NJ; UMDNJ - Robert Wood Johnson Medical Center, New Brunswick, New Jersey); John Hunter (Santa Rosa Sebastopol Cardiology Group, Santa Rosa, CA); David Laxson (University of Minnesota Heart Care, Edina, MN); Eric Ball (Walla Walla Clinic, Walla Walla, WA); Jay Anderson (Internal Medical Association, Grand Island, Nebraska); Terry Wells (TAD Clinical Research, Lufkin,Texas); Kishor Vora (Research Integrity, Owensboro, Kentucky); Eric Ball (Walla Walla Clinic, Walla Walla, Washington); James Welker (Anne Arundel Health System, Annapolis, Maryland).

**Academic Research Organizations and Contract Research Organizations**

***Brazilian Clinical Research Institute, São Paulo, Brazil***

Renato D. Lopes, Flavia Egydio, Anelise Kawakami, Janaina Oliveira.

***Canadian VIGOUR Centre, University of Alberta, Edmonton, Canada***

Shaun G. Goodman, Julianna Wozniak.

***Covance, Marlow, Buckinghamshire, UK***

Alexander Matthews, Caroline Ratky, Janine Valiris.

***Duke Clinical Research Institute, Durham, NC, USA***

Lisa Berdan, Anita Hepditch, Kirby Quintero, Matthew T. Roe, Tyrus Rorick, Melissa Westbrook.

***Estudios Clínicos Latino America, Rosario, Santa Fe, Argentina***

Rafael Diaz, Andrea Pascual, Carla Rovito.

***French Alliance for Cardiovascular Trials (FACT), an F-CRIN network, Paris, France***

Nicolas Danchin, Madeleine Bezault, Elodie Drouet, Tabassome Simon.

***Green Lane Coordinating Centre, Kingsland, Auckland, New Zealand***

Harvey D. White, Caroline Alsweiler.

***Leuven Klinisch Coördinatiecentrum, Leuven, Belgium***

Peter Sinnaeve, Anne Luyten.

***South Australian Health & Medical Research Institute***

Philip E Aylward, Julie Butters, Liddy Griffith, Michelle Shaw.

***Uppsala kliniska forskningscentrum, Uppsala, Sweden***

Emil Hagström, Lena Grunberg.

**Independent Academic Statisticians: SUNY Downstate School of Public Health**

Michael Szarek, Shahidul Islam.

**Sponsors**

***Medical Operations***

Marie-France Brégeault, Nathalie Bougon, Douglas Faustino, Sylvie Fontecave, Judith Murphy, Jean-Francois Tamby, Melanie Verrier (Sanofi, France).

***Trial Operations****

Veronique Agnetti, Dorthe Andersen, Emmy Badreddine, Mhamed Bekkouche, Cecile Bouancheau, Imane Brigui, Maddy Brocklehurst, Joseph Cianciarulo, Dawn Devaul, Szilvia Domokos, Cecile Gache, Caroline Gobillot, Severine Guillou, Jan Healy, Megan Heath, Gayatri Jaiwal, Carine Javierre, Julien Labeirie, Myriam Monier, Ulises Morales, Asmaa Mrabti, Bicky Mthombeni, Betim Okan, Lucile Smith, Jennifer Sheller, Sebastien Sopena, Valerie Pellan (*Sanofi, Paris, France*).

**Excluding North American countries, which were managed by Duke Clinical Research Institute (see above).*

***Pharmacovigilance***

Fadela Benbernou, Nafissa Bengrait, Maud Lamoureux, Katarina Kralova, Michel Scemama (Sanofi, Paris, France).

***Statisticians***

Raphael Bejuit, Anthony Coulange, Christelle Berthou, Jérôme Repincay, Christelle Lorenzato, Alexis Etienne, Valerie Gouet, Guillaume Lecorps, Virginie Loizeau, Mickael Normand, Anne Ourliac, Christelle Rondel (Sanofi, Paris, France).

***Investigational Product Management***

Antony Adamo, Pascale Beltran, Pauline Barraud, Helene Dubois-Gache, Benjamin Halle, Lamia Metwally, Maxime Mourgues, Marc Sotty, Marion Vincendet (Sanofi, Paris, France).

***Clinical Documentation***

Raluca Cotruta, Zhu Chengyue, Dominique Fournie-Lloret, Christine Morrello, Aurelie Perthuis, Patrick Picault, Isabelle Zobouyan (Sanofi, France).

***Diabetes Expert Review Committee***

Helen M. Colhoun, University of Edinburgh, Edinburgh, Scotland, UK.

Michael A. Dempsey, Endocrine & Metabolic Consultants, Rockville, MD, USA.

Mark A. McClanahan, Diabetes and Thyroid Associates, PC, Fredericksburg, USA.

# Institutional Review Board (IRB)/Independent Ethics Committee (IEC) list

| **Local IRB/IEC** | **National IRB/IEC** |
| --- | --- |
| CEI Hospital Italiano de La Plata  Avenida 51 Entre 29 Y 30 S / N  La Plata  B1900AXI  Buenos Aires Province  ARGENTINA |  |
| Comite de Etica en Investigacion CEIC  Larrea 1381 3° A  Caba  C1117ABK  Buenos Aires  ARGENTINA |  |
| Comite de Etica en Investigacion CEIC  Larrea 1381 3° A  Caba  C1117ABK  Buenos Aires  ARGENTINA |  |
| CECIC - Comité de Ética de CER Investigaciones Clínicas  Vicente Lopez 1441  Quilmes  B1878DVB  Buenos Aires Province  ARGENTINA |  |
| Comité de Ética y Revisión Institucional - CEYRI  Lebensohn 31  Junin  B6000BHA  Buenos Aires Province  ARGENTINA |  |
| Comité de Ética en Investigación Fundación Oncosalud  Siria 16  Pergamino  B2700GOB  Buenos Aires Province  ARGENTINA |  |
| Comite de Etica en Investigacion Clínica Olivos  Maipu 1660  Olivos  B1602ABQ  Buenos Aires Province  ARGENTINA |  |
| Comite Institucional de Etica de la salud Clinica Colombo  Duarte Quiros 1948  Cordoba  X5002AOQ  Córdoba  ARGENTINA |  |
| Comite de Etica en Investigacion CEIC  Larrea 1381 3° A  Caba  C1117ABK  Buenos Aires  ARGENTINA |  |
| Comite de Etica independiente ICBA  Blando Encalada 1525  Caba  C1428DCO  Buenos Aires  ARGENTINA |  |
| Comite de Etica en Investigacion CEIC  Larrea 1381 3° A  Caba  C1117ABK  Buenos Aires  ARGENTINA |  |
| CECIC - Comité de Ética de CER Investigaciones Clínicas  Vicente Lopez 1441  Quilmes  B1878DVB  Buenos Aires Province  ARGENTINA |  |
| CESI - Comite de Etica San Isidro  Avenida del Libertador 16958  San Isidro  B1643CRO  Buenos Aires Province  ARGENTINA |  |
| CEI Instituto de Investigaciones Clínicas  Avda. Colón 3364  Mar Del Plata  B7600FZN  Buenos Aires Province  ARGENTINA |  |
| CESI - Comite de Etica San Isidro  Avenida del Libertador 16958  San Isidro  B1643CRO  Buenos Aires Province  ARGENTINA |  |
| Comite de Etica en Investigacion CEIC  Larrea 1381 3° A  Caba  C1117ABK  Buenos Aires  ARGENTINA |  |
| CEI Instituto de Investigaciones Clínicas  Avda. Colón 3364  Mar Del Plata  B7600FZN  Buenos Aires Province  ARGENTINA |  |
| CIB Hospital Municipal De Agudos Leonidas Lucero  Estomba 968  Bahia Blanca  B8000ABA  Buenos Aires Province  ARGENTINA |  |
| Comité Independiente de Ética Clinica Chutro  Avenida Colón 788  Córdoba  X5000EPU  Córdoba  ARGENTINA |  |
| Comité de Ética e Investigación Fundación Sanatorio Guemes  Acuña de Figueroa 1240  Caba  C1180AAX  Buenos Aires  ARGENTINA |  |
| Comité Independiente de Ética Clinica Chutro  Avenida Colón 788  Córdoba  X5000EPU  Córdoba  ARGENTINA |  |
| Comité Independiente de Ética Clinica Chutro  Avenida Colón 788  Córdoba  X5000EPU  Córdoba  ARGENTINA |  |
| Comite de Etica en Investigacion CEIC  Larrea 1381 3° A  Caba  C1117ABK  Buenos Aires  ARGENTINA |  |
| Comite de Etica en Investigacion CEIC  Larrea 1381 3° A  Caba  C1117ABK  Buenos Aires  ARGENTINA |  |
| Comite de Etica en Investigacion CEIC  Larrea 1381 3° A  Caba  C1117ABK  Buenos Aires  ARGENTINA |  |
| Comite Inst. de Etica en Inv. en salud del Sanatorio Allende  Hipólito Yrigoyen 384  Córdoba  X5000JHQ  Córdoba  ARGENTINA |  |
| Comité de Ética Independientes Consultorios Integrados  Italia 424  Rosario  S2000DEJ  Santa Fe  ARGENTINA |  |
| Comite de Etica en Investigacion CEIC  Larrea 1381 3° A  Caba  C1117ABK  Buenos Aires  ARGENTINA |  |
| Comité de ética Instituto Medico Elsa Pérez  Tte. Gral. Ricchieri 4794  Ciudadela  B1702BML  Buenos Aires Province  ARGENTINA |  |
| CIEIS Hospital Privado de Cordoba  Friuli 2786  Cordoba  X5016KEH  Córdoba  ARGENTINA |  |
| Comité Independiente de Ética Clinica Chutro  Avenida Colón 788  Córdoba  X5000EPU  Córdoba  ARGENTINA |  |
| CEI Instituto de Investigaciones Clínicas  Avda. Colón 3364  Mar Del Plata  B7600FZN  Buenos Aires Province  ARGENTINA |  |
| Comite de Etica en Investigacion CEIC  Larrea 1381 3° A  Caba  C1117ABK  Buenos Aires  ARGENTINA |  |
| Comite de Etica DIM Cinica Privada  Av. Belgrano 136  Ramos Mejia  B1704ETD  Buenos Aires Province  ARGENTINA |  |
| CEI - H. Zonal Gral de Agudos Dr. R. Carrillo  Hipolito Yrigoyen 1051  Ciudadela  B1702FWM  Buenos Aires Province  ARGENTINA |  |
| CESI - Comite de Etica San Isidro  Avenida del Libertador 16958  San Isidro  B1643CRO  Buenos Aires Province  ARGENTINA |  |
| Comité Independiente de Ética Fundacion Rusculleda  Av. Colon 2057  Cordoba  X5003DCE  Córdoba  ARGENTINA |  |
| Comite de Etica en Investigacion CEIC  Larrea 1381 3° A  Caba  C1117ABK  Buenos Aires  ARGENTINA |  |
| Comité de Ética Instituto Médico Platense CEDIMP  Boulevard 51 Nº 315  B1900AVG  ARGENTINA |  |
| Hospital Italiano Bs.As. CEPI  Juan D. Peron 4190  Caba  C1181ACH  Buenos Aires  ARGENTINA |  |
| CIEIS - Hospital Italiano de Cordoba  Roma 550 Barrio General Paz  X5004BAL  Córdoba  ARGENTINA |  |
| Comite de Etica en Investigacion CEIC  Larrea 1381 3° A  Caba  C1117ABK  Buenos Aires  ARGENTINA |  |
| CESI - Comite de Etica San Isidro  Avenida del Libertador 16958  San Isidro  B1643CRO  Buenos Aires Province  ARGENTINA |  |
| Comite de Etica en Investigacion CEIC  Larrea 1381 3° A  Caba  C1117ABK  Buenos Aires  ARGENTINA |  |
| Comité de ética Instituto Medico Elsa Pérez  Tte. Gral. Ricchieri 4794  Ciudadela  B1702BML  Buenos Aires Province  ARGENTINA |  |
| CIEIS - Santiago del Estero  Belgrano 1912  G4200ABT  Santiago del Estero  ARGENTINA |  |
| CEI Clinica y Maternidad Suizo Argentina  Av. Pueyrredon 1486  C1414CED  Buenos Aires  ARGENTINA |  |
| Comité Independiente de Ética Fundacion Rusculleda  Av. Colon 2057  Cordoba  X5003DCE  Córdoba  ARGENTINA |  |
| Comite de Bioetica Hospital Universitario Fundacion Favaloro  Av Belgrano 1746  Caba  C1093AAS  Buenos Aires  ARGENTINA |  |
| Comite de Etica en Investigacion CEIC  Larrea 1381 3° A  Caba  C1117ABK  Buenos Aires  ARGENTINA |  |
| Comité de Bioética HIGA "Eva Perón"  Ricardo Balbin 900  San Martin  B1650NBN  Buenos Aires Province  ARGENTINA |  |
| Comite de Etica en Investigacion CEIC  Larrea 1381 3° A  Caba C1117ABK  Buenos Aires  ARGENTINA |  |
| Bellberry Limited  129 Glen Osmond Road  Eastwood  5063  South Australia  AUSTRALIA |  |
| Concord Repatriation General Hospital  Hospital Rd  Concord 2139  New South Wales  AUSTRALIA |  |
| Hunter New England Human Research Ethics Committee  Lookout Road  New Lambton Heights  2305  New South Wales  AUSTRALIA |  |
| St Vincent’s Aged and Health Care HREC  48 Montpeller Road  Bowen Hills  Queensland  AUSTRALIA |  |
| Bellberry Limited  129 Glen Osmond Road  Eastwood  5063  South Australia  AUSTRALIA |  |
| Hunter New England Human Research Ethics Committee  Lookout Road  New Lambton Heights  2305  New South Wales  AUSTRALIA |  |
| Hunter New England Human Research Ethics Committee  Lookout Road  New Lambton Heights  2305  New South Wales  AUSTRALIA |  |
| Hunter New England Human Research Ethics Committee  Lookout Road  New Lambton Heights  2305  New South Wales  AUSTRALIA |  |
| Hunter New England Human Research Ethics Committee  Lookout Road  New Lambton Heights  2305  New South Wales  AUSTRALIA |  |
| Bellberry Limited  129 Glen Osmond Road  Eastwood  5063  South Australia  AUSTRALIA |  |
| Hunter New England Human Research Ethics Committee  Lookout Road  New Lambton Heights  2305  New South Wales  AUSTRALIA |  |
| The Alfred Research & Ethics Unit  Commercial Road  Prahran  3181  Victoria  AUSTRALIA |  |
| Calvary Hospital  Calvary Health Care Human Research and Ethics Committee, PO Box 254  Jamison Centre  2614 Australian Capital Territory  AUSTRALIA |  |
| Southern Adelaide Clincial Human Research Ethics Committee  Flinders Drive  Flinders Medical Centre  Bedford Park5042  South Australia  AUSTRALIA |  |
| Hunter New England Human Research Ethics Committee  Lookout Road  New Lambton Heights  2305  New South Wales  AUSTRALIA |  |
| Hunter New England Human Research Ethics Committee  Lookout Road  New Lambton Heights  2305 New South Wales  AUSTRALIA |  |
| Southern Adelaide Clincial Human Research Ethics Committee  Flinders Drive  Flinders Medical Centre  Bedford Park  5042 South Australia  AUSTRALIA |  |
| Hunter New England Human Research Ethics Committee  Lookout Road  New Lambton Heights  2305  New South Wales  AUSTRALIA |  |
| Epworth Human Research Ethics Committee  Clinical Trials and Research Centre  185-187 Hoddle Street  Richmond  3121 Victoria  AUSTRALIA |  |
| Bellberry Limited  129 Glen Osmond Road  Eastwood  5063 South Australia  AUSTRALIA |  |
| Hunter New England Human Research Ethics Committee  Lookout Road  New Lambton Heights  2305  New South Wales  AUSTRALIA |  |
| Southern Adelaide Clincial Human Research Ethics Committee  Flinders Drive  Flinders Medical Centre  Bedford Park  5042  South Australia  AUSTRALIA |  |
| Hunter New England Human Research Ethics Committee  Lookout Road  New Lambton Heights  2305 New South Wales  AUSTRALIA |  |
| Human Research Ethics Committee (Tasmania) Network  University of Tasmania  Private Bag 1  Hobart  7001  Tasmania  AUSTRALIA |  |
| Hunter New England Human Research Ethics Committee  Lookout Road  New Lambton Heights  2305  New South Wales  AUSTRALIA |  |
|  | Ethik-Kommission für das Bundesland Salzburg  Sebastian-Stief-Gasse 2  Salzburg  5020  AUSTRIA |
|  | Ethik-Kommission für das Bundesland Salzburg  Sebastian-Stief-Gasse 2  Salzburg  5020  AUSTRIA |
|  | Ethik-Kommission für das Bundesland Salzburg  Sebastian-Stief-Gasse 2  Salzburg  5020  AUSTRIA |
|  | Ethik-Kommission für das Bundesland Salzburg  Sebastian-Stief-Gasse 2  Salzburg  5020  AUSTRIA |
|  | Ethik-Kommission für das Bundesland Salzburg  Sebastian-Stief-Gasse 2  Salzburg  5020  AUSTRIA |
|  | Ethik-Kommission für das Bundesland Salzburg  Sebastian-Stief-Gasse 2  Salzburg  5020  AUSTRIA |
|  | Commissie voor Medische Ethiek/Klinisch onderzoek  Herestraat 49  Leuven  3000  BELGIUM |
|  | Commissie voor Medische Ethiek/Klinisch onderzoek  Herestraat 49  Leuven  3000  BELGIUM |
|  | Commissie voor Medische Ethiek/Klinisch onderzoek  Herestraat 49  Leuven  3000  BELGIUM |
|  | Commissie voor Medische Ethiek/Klinisch onderzoek  Herestraat 49  Leuven  3000  BELGIUM |
|  | Commissie voor Medische Ethiek/Klinisch onderzoek  Herestraat 49  Leuven  3000  BELGIUM |
|  | Commissie voor Medische Ethiek/Klinisch onderzoek  Herestraat 49  Leuven  3000  BELGIUM |
|  | Commissie voor Medische Ethiek/Klinisch onderzoek  Herestraat 49  Leuven  3000  BELGIUM |
|  | Commissie voor Medische Ethiek/Klinisch onderzoek  Herestraat 49  Leuven  3000  BELGIUM |
|  | Commissie voor Medische Ethiek/Klinisch onderzoek  Herestraat 49  Leuven  3000  BELGIUM |
|  | Commissie voor Medische Ethiek/Klinisch onderzoek  Herestraat 49  Leuven  3000  BELGIUM |
|  | Commissie voor Medische Ethiek/Klinisch onderzoek  Herestraat 49  Leuven  3000  BELGIUM |
|  | Commissie voor Medische Ethiek/Klinisch onderzoek  Herestraat 49  Leuven  3000  BELGIUM |
|  | Commissie voor Medische Ethiek/Klinisch onderzoek  Herestraat 49  Leuven  3000  BELGIUM |
|  | Commissie voor Medische Ethiek/Klinisch onderzoek  Herestraat 49  Leuven  3000  BELGIUM |
|  | Commissie voor Medische Ethiek/Klinisch onderzoek  Herestraat 49  Leuven  3000  BELGIUM |
|  | Commissie voor Medische Ethiek/Klinisch onderzoek  Herestraat 49  Leuven  3000  BELGIUM |
|  | Commissie voor Medische Ethiek/Klinisch onderzoek  Herestraat 49  Leuven  3000  BELGIUM |
|  | Commissie voor Medische Ethiek/Klinisch onderzoek  Herestraat 49  Leuven  3000  BELGIUM |
|  | Commissie voor Medische Ethiek/Klinisch onderzoek  Herestraat 49  Leuven  3000  BELGIUM |
|  | Commissie voor Medische Ethiek/Klinisch onderzoek  Herestraat 49  Leuven  3000  BELGIUM |
|  | Commissie voor Medische Ethiek/Klinisch onderzoek  Herestraat 49  Leuven  3000  BELGIUM |
| Clinical Center Banja Luka  Ethics Committee  12 Beba bb  Banja Luka  78000  BOSNIA AND HERZEGOVINA |  |
| DEAMEDICA PJ Medicinska Elektronika  Ethics Committee Vuka Karadžića 6  Banja Luka  78000  BOSNIA AND HERZEGOVINA |  |
| Ethics Commitee Cantonal Hospital "Dr. Safet Mujic"  Marsala Tita 294  Mostar  8000  BOSNIA AND HERZEGOVINA |  |
| Hospital Mostar  University Clinical  Bijeli brijeg bb  Mostar  8000  BOSNIA AND HERZEGOVINA |  |
| Ethics Committee  University Clinical Center Sarajevo  Bolnicka 25  Sarajevo  71000  BOSNIA AND HERZEGOVINA |  |
| Ethics Committee Cantonal Hospital Zenica  Crkvice 67  Zenica  72000  BOSNIA AND HERZEGOVINA |  |
| Ethics Committee  University Clinical Center Sarajevo  Bolnicka 25  Sarajevo  71000  BOSNIA AND HERZEGOVINA |  |
| Ethics Committee  University Medical Center Tuzla  Trnovac bb  Tuzla  75000  BOSNIA AND HERZEGOVINA |  |
| Ethics Committee Heart Center BH  Heart Center BH  Alekse Santica 8  Tuzla  75000  BOSNIA AND HERZEGOVINA |  |
| Ethics Committee State Hospital'Abdulah Nakas'  Kranjceviceva 12  Sarajevo  71000  BOSNIA AND HERZEGOVINA |  |
| Cantonal Hospital "Dr. Irfan Ljubijankic" Bihac  Darovalaca krvi 67  Bihac  77000  BOSNIA AND HERZEGOVINA |  |
| Instituto de Cardiologia do Rio Grande do Sul/Fundacao Unive  Av. Princesa Isabel, 370  Porto Alegre  90620-001  Rio Grande do Sul  BRAZIL | Comissão Nacional de Ética em Pesquisa - CONEP  Edifício Ex-INAN - Unidade II - Ministério da Saúde  SEPN 510 NORTE, BLOCO A, 3º Andar  Brasilia  70750-521  BRAZIL |
| EC of Universidade Regional de Blumenau / FURB  Rua Antonio da Veiga, 140  Bairro Victor Konder  Blumenau  89010-971  Santa Catarina  BRAZIL | Comissão Nacional de Ética em Pesquisa - CONEP  Edifício Ex-INAN - Unidade II - Ministério da Saúde  SEPN 510 NORTE, BLOCO A, 3º Andar  Brasilia  70750-521  BRAZIL |
| EC of Hospital Maternidade Angelina Caron  Rodovia do Caqui, 1150 km 1  Campina Grande Do Sul  83430-000  Parana  BRAZIL | Comissão Nacional de Ética em Pesquisa - CONEP  Edifício Ex-INAN - Unidade II - Ministério da Saúde  SEPN 510 NORTE, BLOCO A, 3º Andar  Brasilia  70750-521  BRAZIL |
| EC of Hospital Vera Cruz  Av. Barbacena, 653  Barro Preto  Belo Horizonte  Minas Gerais  BRAZIL | Comissão Nacional de Ética em Pesquisa - CONEP  Edifício Ex-INAN - Unidade II - Ministério da Saúde  SEPN 510 NORTE, BLOCO A, 3º Andar  Brasilia  70750-521  BRAZIL |
| EC of Sociedade Evangelica Beneficente de Curitiba  Rua Padre Anchieta, 2770  Curitiba  80730-150  Parana  BRAZIL | Comissão Nacional de Ética em Pesquisa - CONEP  Edifício Ex-INAN - Unidade II - Ministério da Saúde  SEPN 510 NORTE, BLOCO A, 3º Andar  Brasilia  70750-521  BRAZIL |
| EC of Pesquisa do Investiga - Instituto de Pesquisas  Avenida Romeu Tórtima, 739 - Cidade Universitária – Campinas/SP, CEP: 13084-791  Sao Paulo  BRAZIL | Comissão Nacional de Ética em Pesquisa - CONEP  Edifício Ex-INAN - Unidade II - Ministério da Saúde  SEPN 510 NORTE, BLOCO A, 3º Andar  Brasilia  70750-521  BRAZIL |
| EC of Centro Universitario de Brasilia  SEPN 707/907, Campus do UniCEUB, Bloco 6  Brasilia  70790-075  Distrito Federal  BRAZIL | Comissão Nacional de Ética em Pesquisa - CONEP  Edifício Ex-INAN - Unidade II - Ministério da Saúde  SEPN 510 NORTE, BLOCO A, 3º Andar  Brasilia  70750-521  BRAZIL |
| EC of Hospital Universitario Walter Cantidio / UFCE  Rua Capitao Francisco Pedro, 1290  Rodolfo Teofilo  Fortaleza  60430-370  Ceara  BRAZIL | Comissão Nacional de Ética em Pesquisa - CONEP  Edifício Ex-INAN - Unidade II - Ministério da Saúde  SEPN 510 NORTE, BLOCO A, 3º Andar  Brasilia  70750-521  BRAZIL |
| EC of Hospital Universitario Walter Cantidio / UFCE  Rua Capitao Francisco Pedro, 1290  Rodolfo Teofilo  Fortaleza  60430-370  Ceara  BRAZIL | Comissão Nacional de Ética em Pesquisa - CONEP  Edifício Ex-INAN - Unidade II - Ministério da Saúde  SEPN 510 NORTE, BLOCO A, 3º Andar  Brasilia  70750-521  BRAZIL |
| EC of Hospital Pro-Cardiaco Pronto Socorro Cardiologico  Rua Paulo Barreto, 86  Botafogo  Rio De Janeiro  22280-010  Rio de Janeiro  BRAZIL | Comissão Nacional de Ética em Pesquisa - CONEP  Edifício Ex-INAN - Unidade II - Ministério da Saúde  SEPN 510 NORTE, BLOCO A, 3º Andar  Brasilia  70750-521  BRAZIL |
| EC of Pesquisa do Inst de Cardiologia do Distrito Federal (ICDF)  Brasilia  70658700  Distrito Federal  BRAZIL | Comissão Nacional de Ética em Pesquisa - CONEP  Edifício Ex-INAN - Unidade II - Ministério da Saúde  SEPN 510 NORTE, BLOCO A, 3º Andar  Brasilia  70750-521  BRAZIL |
| EC of Hospital de Clinicas da Universidade Federal de Goias  Primeira Avenida, 545 Quadra 68  Setor Universitario  Goiania  74605-050  Goias  BRAZIL | Comissão Nacional de Ética em Pesquisa - CONEP  Edifício Ex-INAN - Unidade II - Ministério da Saúde  SEPN 510 NORTE, BLOCO A, 3º Andar  Brasilia  70750-521  BRAZIL |
| EC of Hospital Pro-Cardiaco Pronto Socorro Cardiologico  Rua Paulo Barreto, 86  Botafogo  Rio De Janeiro  22280-010  Rio de Janeiro  BRAZIL | Comissão Nacional de Ética em Pesquisa - CONEP  Edifício Ex-INAN - Unidade II - Ministério da Saúde  SEPN 510 NORTE, BLOCO A, 3º Andar  Brasilia  70750-521  BRAZIL |
| EC of Pontifícia Universidade Católica do Rio Grande do Sul  Av. Ipiranga, 6681  sala 703, predio 50  Jd. Botanico  Porto Alegre  90619-900  Rio Grande do Sul  BRAZIL | Comissão Nacional de Ética em Pesquisa - CONEP  Edifício Ex-INAN - Unidade II - Ministério da Saúde  SEPN 510 NORTE, BLOCO A, 3º Andar  Brasilia  70750-521  BRAZIL |
| EC Professor Dr. Celso Figueiroa - Hospital Santa Izabel  Praca Conselheiro Almeida Couto, 500  Bairro Nazare  Salvador  40050-410  Bahia  BRAZIL | Comissão Nacional de Ética em Pesquisa - CONEP  Edifício Ex-INAN - Unidade II - Ministério da Saúde  SEPN 510 NORTE, BLOCO A, 3º Andar  Brasilia  70750-521  BRAZIL |
| EC of Faculdade de Medicina do ABC  Avenida Príncipe de Gales, 821  Bairro Príncipe de Gales  Santo Andre  09060-650  Sao Paulo  BRAZIL | Comissão Nacional de Ética em Pesquisa - CONEP  Edifício Ex-INAN - Unidade II - Ministério da Saúde  SEPN 510 NORTE, BLOCO A, 3º Andar  Brasilia  70750-521  BRAZIL |
| EC of Hospital Vera Cruz  Av. Barbacena, 653  Barro Preto  Belo Horizonte  Minas Gerais  BRAZIL | Comissão Nacional de Ética em Pesquisa - CONEP  Edifício Ex-INAN - Unidade II - Ministério da Saúde  SEPN 510 NORTE, BLOCO A, 3º Andar  Brasilia  70750-521  BRAZIL |
| EC of Santa Casa de Misericordia de Pelotas  Praca Piratinino de Almeida, 53  Pelotas  96015-290  Rio Grande do Sul  BRAZIL | Comissão Nacional de Ética em Pesquisa - CONEP  Edifício Ex-INAN - Unidade II - Ministério da Saúde  SEPN 510 NORTE, BLOCO A, 3º Andar  Brasilia  70750-521  BRAZIL |
| Ethics Committee of Universidade de Passo Fundo  Campus I  Km171 - BR285  Bairro Sao Jose  Passo Fundo  99001-970  Rio Grande do Sul  BRAZIL | Comissão Nacional de Ética em Pesquisa - CONEP  Edifício Ex-INAN - Unidade II - Ministério da Saúde  SEPN 510 NORTE, BLOCO A, 3º Andar  Brasilia  70750-521  BRAZIL |
| EC of Faculdade de Medicina de Sao Jose do Rio Preto  Av. Brigadeiro Faria Lima, - 5416 - Vila São Pedro  Sao José Do Rio Preto  15090-000  Sao Paulo  BRAZIL | Comissão Nacional de Ética em Pesquisa - CONEP  Edifício Ex-INAN - Unidade II - Ministério da Saúde  SEPN 510 NORTE, BLOCO A, 3º Andar  Brasilia  70750-521  BRAZIL |
| EC of Hospital de Clinicas da Universidade Federal de Goias  Primeira Avenida, 545 Quadra 68  Setor Universitario  Goiania  74605-050  Goias  BRAZIL | Comissão Nacional de Ética em Pesquisa - CONEP  Edifício Ex-INAN - Unidade II - Ministério da Saúde  SEPN 510 NORTE, BLOCO A, 3º Andar  Brasilia  70750-521  BRAZIL |
| EC of Hospital Vera Cruz  Av. Barbacena, 653  Barro Preto  Belo Horizonte  Minas Gerais  BRAZIL | Comissão Nacional de Ética em Pesquisa - CONEP  Edifício Ex-INAN - Unidade II - Ministério da Saúde  SEPN 510 NORTE, BLOCO A, 3º Andar  Brasilia  70750-521  BRAZIL |
| Ethics Committee of Universidade Federal de Sao Paulo  Rua Botucatu, 572  1 Andar - Conjunto 14  Sao Paulo  04023-061  Sao Paulo  BRAZIL | Comissão Nacional de Ética em Pesquisa - CONEP  Edifício Ex-INAN - Unidade II - Ministério da Saúde  SEPN 510 NORTE, BLOCO A, 3º Andar  Brasilia  70750-521  BRAZIL |
| Ethics Committee of Irm. Santa Casa de Mis. de Porto Alegre  Rua Professor Annes Dias, 295  Porto Alegre  90020-090  Rio Grande do Sul  BRAZIL | Comissão Nacional de Ética em Pesquisa - CONEP  Edifício Ex-INAN - Unidade II - Ministério da Saúde  SEPN 510 NORTE, BLOCO A, 3º Andar  Brasilia  70750-521  BRAZIL |
| EC of Irmandade Santa Casa de Misericordia de Sao Paulo  R. Dr. Cesario Mota Jr.,112  Sao Paulo  01277-900  Sao Paulo  BRAZIL | Comissão Nacional de Ética em Pesquisa - CONEP  Edifício Ex-INAN - Unidade II - Ministério da Saúde  SEPN 510 NORTE, BLOCO A, 3º Andar  Brasilia  70750-521  BRAZIL |
| EC of Associacao Educadora Sao Carlos - H. Mae de Deus  Rua José de Alencar, 286  Bairro Menino Jesus  Porto Alegre  90880-480  Rio Grande do Sul  BRAZIL | Comissão Nacional de Ética em Pesquisa - CONEP  Edifício Ex-INAN - Unidade II - Ministério da Saúde  SEPN 510 NORTE, BLOCO A, 3º Andar  Brasilia  70750-521  BRAZIL |
| EC of Grupo Hospitalar Conceicao  Av. Francisco Trein, 596 bloco H 3 andar  Cristo Redentor  Porto Alegre  91350-200  Rio Grande do Sul  BRAZIL | Comissão Nacional de Ética em Pesquisa - CONEP  Edifício Ex-INAN - Unidade II - Ministério da Saúde  SEPN 510 NORTE, BLOCO A, 3º Andar  Brasilia  70750-521  BRAZIL |
| EC of Analise de Projetos de Pesquisa-CAPPesq-CEP HCFMUSP  EC of Analise de Projetos de Pesquisa-CAPPesq-CEP HCFMUSP  Rua Dr. Ovídio Pires de Campos, 225 - Cerqueira César  5o andar  Sao Paulo  05403-010  Sao Paulo BRAZIL | Comissão Nacional de Ética em Pesquisa - CONEP  Edifício Ex-INAN - Unidade II - Ministério da Saúde  SEPN 510 NORTE, BLOCO A, 3º Andar  Brasilia  70750-521  BRAZIL |
| Ethics Committee of Hospital Geral de Goiania  Av. Anhanguera, 6479  Setor Oeste  Goiania  74110-010  Goias  BRAZIL | Comissão Nacional de Ética em Pesquisa - CONEP  Edifício Ex-INAN - Unidade II - Ministério da Saúde  SEPN 510 NORTE, BLOCO A, 3º Andar  Brasilia  70750-521  BRAZIL |
| EC of Instituto de Molestias Cardiovasculares SJRP  R. Castelo D'Agua, 3030  Bairro Redentora  Sao Jose Do Rio Preto  15015-210  Sao Paulo  BRAZIL | Comissão Nacional de Ética em Pesquisa - CONEP  Edifício Ex-INAN - Unidade II - Ministério da Saúde  SEPN 510 NORTE, BLOCO A, 3º Andar  Brasilia  70750-521  BRAZIL |
| EC of Faculdade de Medicina do ABC  Avenida Príncipe de Gales, 821  Bairro Príncipe de Gales  Santo Andre  09060-650  Sao Paulo  BRAZIL | Comissão Nacional de Ética em Pesquisa - CONEP  Edifício Ex-INAN - Unidade II - Ministério da Saúde  SEPN 510 NORTE, BLOCO A, 3º Andar  Brasilia  70750-521  BRAZIL |
| EC of Fund. Pub. Estadual Hospital de Clinicas Gaspar Vianna  Travessa Alferes Costa s/n - Bairro Pedreira  Belem  66087-660  Para  BRAZIL | Comissão Nacional de Ética em Pesquisa - CONEP  Edifício Ex-INAN - Unidade II - Ministério da Saúde  SEPN 510 NORTE, BLOCO A, 3º Andar  Brasilia  70750-521  BRAZIL |
| EC of Pontificia Universidade Catolica do Parana  Rua Imaculada Conceição, 1155  3 andar - Predio Administrativo  Curitiba  80215-901  Parana  BRAZIL | Comissão Nacional de Ética em Pesquisa - CONEP  Edifício Ex-INAN - Unidade II - Ministério da Saúde  SEPN 510 NORTE, BLOCO A, 3º Andar  Brasilia  70750-521  BRAZIL |
| Ethics Committee of Universidade Federal de Uberlandia  Av. Joao Naves de Avila, 2121  Bairro Santa Monica  Uberlandia  38400-098  Minas Gerais  BRAZIL | Comissão Nacional de Ética em Pesquisa - CONEP  Edifício Ex-INAN - Unidade II - Ministério da Saúde  SEPN 510 NORTE, BLOCO A, 3º Andar  Brasilia  70750-521  BRAZIL |
| EC of Hosp. de Clinicas Faculd. Medicina USP Ribeirao Preto  Campus Universitario Monte Alegre  Ribeirao Preto  14048-900  Sao Paulo  BRAZIL | Comissão Nacional de Ética em Pesquisa - CONEP  Edifício Ex-INAN - Unidade II - Ministério da Saúde  SEPN 510 NORTE, BLOCO A, 3º Andar  Brasilia  70750-521  BRAZIL |
| EC of Faculdade de Medicina do ABC  Avenida Príncipe de Gales, 821  Bairro Príncipe de Gales  Santo Andre  09060-650  Sao Paulo  BRAZIL | Comissão Nacional de Ética em Pesquisa - CONEP  Edifício Ex-INAN - Unidade II - Ministério da Saúde  SEPN 510 NORTE, BLOCO A, 3º Andar  Brasilia  70750-521  BRAZIL |
| EC of Fundação Hospitalar São Francisco de Assis  Rua Itamaracá, 535  Belo Horizonte  31110580  Minas Gerais  BRAZIL | Comissão Nacional de Ética em Pesquisa - CONEP  Edifício Ex-INAN - Unidade II - Ministério da Saúde  SEPN 510 NORTE, BLOCO A, 3º Andar  Brasilia  70750-521  BRAZIL |
| EC of Pequisa da Sociedade Hosp. Samaritano (CEP-HS)  R. Conselheiro Brotero, 1486 - Higienópolis  Sao Paulo  01232-010  BRAZIL | Comissão Nacional de Ética em Pesquisa - CONEP  Edifício Ex-INAN - Unidade II - Ministério da Saúde  SEPN 510 NORTE, BLOCO A, 3º Andar  Brasilia  70750-521  BRAZIL |
| EC of Associacao Educadora Sao Carlos - H. Mae de Deus  Rua José de Alencar, 286  Bairro Menino Jesus  Porto Alegre  90880-480  Rio Grande do Sul  BRAZIL | Comissão Nacional de Ética em Pesquisa - CONEP  Edifício Ex-INAN - Unidade II - Ministério da Saúde  SEPN 510 NORTE, BLOCO A, 3º Andar  Brasilia  70750-521  BRAZIL |
| EC Professor Dr. Celso Figueiroa - Hospital Santa Izabel  Praca Conselheiro Almeida Couto, 500  Bairro Nazare  Salvador  40050-410  Bahia  BRAZIL | Comissão Nacional de Ética em Pesquisa - CONEP  Edifício Ex-INAN - Unidade II - Ministério da Saúde  SEPN 510 NORTE, BLOCO A, 3º Andar  Brasilia  70750-521  BRAZIL |
| EC of Hospital Sirio Libanes  Rua Peixoto Gomide, 316  11 andar  Sao Paulo  01409-000  Sao Paulo  BRAZIL | Comissão Nacional de Ética em Pesquisa - CONEP  Edifício Ex-INAN - Unidade II - Ministério da Saúde  SEPN 510 NORTE, BLOCO A, 3º Andar  Brasilia  70750-521  BRAZIL |
| Fifth MHAT-Sofia EAD  Department of Cardiology  67A “Gen. Nikolay G. Stoletov” Blvd.  Sofia  1233  BULGARIA | Ethics Committee Multicenter Trials  5 Sveta Nedelya Square  Sofia  1000  BULGARIA |
| UMHAT Tzaritza Yoanna – ISUL EAD  UMHAT “Tsaritsa Yoanna”- ISUL, Clinic of Cardiology  8 Byalo more Str.  Sofia  1527  BULGARIA | Ethics Committee Multicenter Trials  5 Sveta Nedelya Square  Sofia  1000  BULGARIA |
| NMT Hospital "Tsar Boris III"  Clinic of Cardiology, 104 “Knyaginya Maria Luiza”Blvd  Sofia  1233  BULGARIA | Ethics Committee Multicenter Trials  5 Sveta Nedelya Square  Sofia  1000  BULGARIA |
| Univ Multiprofile Hosp for Active Treatment "Sveti Georgi"  Clinic of cardiology - EAD  66 Peshtersko shose Blvd.  Plovdiv  4002  BULGARIA | Ethics Committee Multicenter Trials  5 Sveta Nedelya Square  Sofia  1000  BULGARIA |
| 2nd MHAT  Department of Intensive Cardiology treatment  120, "hristo Botev" Blvd.  Sofia  1202  BULGARIA | Ethics Committee Multicenter Trials  5 Sveta Nedelya Square  Sofia  1000  BULGARIA |
| MHAT “National Cardiology Hospital” EAD  EAD Clinic of Cardiology,  65” Konyovitsa” Str.  Sofia  1309  BULGARIA | Ethics Committee Multicenter Trials  5 Sveta Nedelya Square  Sofia  1000  BULGARIA |
| Multiprofile Hospital for Active Treatment - Pazardzhik AD  Department of Cardiology  15 Bolnichna Str.  Pazardzhik 4400  BULGARIA | Ethics Committee Multicenter Trials  5 Sveta Nedelya Square  Sofia  1000  BULGARIA |
| Multiprofile Hospital for Active Treatment - Burgas AD  Department of Cardiology - Multiprofile Hospital for Active Treatment - Burgas AD  73 Stefan Stambolov Blvd.  Burgas  8000  BULGARIA | Ethics Committee Multicenter Trials  5 Sveta Nedelya Square  Sofia  1000  BULGARIA |
| UMHAT dr Georgi Stranski  Clinic of Cardiology  8A Georgi Kochev Str  Pleven  5800  BULGARIA | Ethics Committee Multicenter Trials  5 Sveta Nedelya Square  Sofia  1000  BULGARIA |
| Multiprof Distr Hosp for Active Treatment "Dr.St.Cherkezov"  Cardiology department -Multiprofile District Hospital for Active Treatment "Dr. St. Cherkezov" AD  1 Nish Str.  Veliko Tarnovo  5000  BULGARIA | Ethics Committee Multicenter Trials  5 Sveta Nedelya Square  Sofia  1000  BULGARIA |
| MHAT ‘Sv. Vrach’ EOOD  Park area  Sandanski  2800  BULGARIA | Ethics Committee Multicenter Trials  5 Sveta Nedelya Square  Sofia  1000  BULGARIA |
| UMHAT "Aleksandrovska"  1 Sveti Georgi Sofiyski str.  Sofia  1431  BULGARIA | Ethics Committee Multicenter Trials  5 Sveta Nedelya Square  Sofia  1000  BULGARIA |
| First Multiprofile Hospital for Active Treatment - Sofia EAD  Department of Cardiology- First 37” Patriarh Evtimiy” Blvd.  Sofia  1142  BULGARIA | Ethics Committee Multicenter Trials  5 Sveta Nedelya Square  Sofia  1000  BULGARIA |
| Local Ethical Committee of Specialized Hosp for Active Treatment in Cardiology EAD  91 Gen. Vladimir Vazov str. Pleven 5800  BULGARIA | Ethics Committee Multicenter Trials  5 Sveta Nedelya Square  Sofia  1000  BULGARIA |
| "MHAT Lyulin" EAD. Department of Internal Diseases  Luylin 6, 81 Dr Petar Dertliev Str.  Sofia  1336  BULGARIA | Ethics Committee Multicenter Trials  5 Sveta Nedelya Square  Sofia  1000  BULGARIA |
| MHAT Blagoevgrad AD - Cardiology Department  60'' Slavyanska'' str.  Blagoevgrad  2700  BULGARIA | Ethics Committee Multicenter Trials  5 Sveta Nedelya Square  Sofia  1000  BULGARIA |
| Specialized Hospital for Active Cardiology Treatment – “Cardiolife” - Cardiology Department  Blvd. Republika 15  Varna  9000  BULGARIA | Ethics Committee Multicenter Trials  5 Sveta Nedelya Square  Sofia  1000  BULGARIA |
| MHAT Bratan Shukerov AD, Cardio. Dept. with Inrtensive Unite  MHAT Bratan Shukerov AD, Cardiology Department  2 Bulgaria Blvd.  Smoljan  4700  BULGARIA | Ethics Committee Multicenter Trials  5 Sveta Nedelya Square  Sofia  1000  BULGARIA |
| Nova Scotia Heath Authority Research Ethics Board, Centre for Clinical Research, 5790 University Ave., Rm 118, Halifax, NS B3H 1V7, CANADA |  |
|  | Institutional Review Board Services, 372 Hollandview Trail, Ste. 300, Aurora, ON L4G 0A5, CANADA |
|  | Institutional Review Board Services, 372 Hollandview Trail, Ste. 300, Aurora, ON L4G 0A5, CANADA |
| Interior Health Research Ethics Board, 1815 Kirschner Rd., Ste. 104, Kelowna, BC V1Y 4N7, CANADA |  |
| Ottawa Health Science Network Research Ethics Board, 725 Parkdale Ave., Civic Box 411 Loeb Bldg., Ottawa, ON K1Y 4E9, CANADA |  |
|  | Institutional Review Board Services, 372 Hollandview Trail, Ste. 300, Aurora, ON L4G 0A5, CANADA |
|  | Institutional Review Board Services, 372 Hollandview Trail, Ste. 300, Aurora, ON L4G 0A5, CANADA |
| Health Research Board of Alberta - Clinical Trials Committee, 10104 103rd Ave., Rm 1500, Edmonton, AB T5J 4A7, CANADA |  |
|  | Institutional Review Board Services, 372 Hollandview Trail, Ste. 300, Aurora, ON L4G 0A5, CANADA |
|  | Institutional Review Board Services, 372 Hollandview Trail, Ste. 300, Aurora, ON L4G 0A5, CANADA |
| University of Alberta Health Research Ethics Board, , 308 Campus Tower, 8625- 112th St., Edmonton, AB T6G 1K8, CANADA |  |
|  | Institutional Review Board Services, 372 Hollandview Trail, Ste. 300, Aurora, ON L4G 0A5, CANADA |
| Horizon Health Network IRB, Saint John Regional Hospital, 400 University Ave., PO BOX 2100, Saint John, NB E2L 4L2,  CANADA |  |
| Nova Scotia Heath Authority Research Ethics Board, Centre for Clinical Research, 5790 University Ave., Rm 118, Halifax, NS B3H 1V7, CANADA |  |
| Vancouver Island Health Authority Research Ethics  Room 003-3970 Haro Rd, Pearkes Bldg. Queen Alexandra Centre  Victoria, BC V8N 4A9 CANADA |  |
| Comite d’ethique de la Recherche Institute Universitaire de Cardiologie et de Pneumologie de Quebec IRB, Hopital Laval IRB, 2725 Chemin Sainte-Foy, Quebec QC G1V 4G5,  CANADA |  |
| Comite d’ethique de la recherche du Centre integre de sante et de services sociaux de chaudiere-Appalaches 143 Rue Wolfe, Levis,QC G6W 3Z1 CANADA |  |
| University of Alberta Health Research Ethics Board, 308 Campus Tower, 8625 – 112th St., Edmonton, AB T6G 1K8,  CANADA |  |
| Centre intégré universitaire de santé et de services sociaux de l'Estrie - Centre hospitalier universitaire de Sherbrooke, 375 Argyll, Sherbrooke, QC J1J 3H5,  CANADA |  |
|  | Institutional Review Board Services, 372 Hollandview Trail, Ste. 300, Aurora, ON L4G 0A5, CANADA |
|  | Institutional Review Board Services, 372 Hollandview Trail, Ste. 300, Aurora, ON L4G 0A5, CANADA |
| Health Research Board of Alberta , 10104 103rd Ave., Rm 1500, Edmonton, AB T5J 4A7, CANADA |  |
| Health Research Ethics Authority, 2nd Floor, Eastern Trust Bldg 95, Bonaventure Ave., St. John’s, ND A1B 2X5, CANADA |  |
|  | Institutional Review Board Services, 372 Hollandview Trail, Ste. 300, Aurora, ON L4G 0A5, CANADA |
|  | Institutional Review Board Services, 372 Hollandview Trail, Ste. 300, Aurora, ON L4G 0A5, CANADA |
| Thunder Bay Regional Health Sciences Centre REB, 908 Oliver Road, Thunder Bay, ON P7B 6V4, CANADA | Institutional Review Board Services, 372 Hollandview Trail, Ste. 300, Aurora, ON L4G 0A5, CANADA |
| University of Manitoba Bannatyne Campus Biomedical Research Ethics Board, 770 Bannatyne Ave., Winnipeg, MB R3E 0W3, CANADA |  |
| CSSS du Nord de Lanaudiere - Centre Hospitalier Regional de Lanaudiere IRB, 911 Montee Des Pionniers, Terrebonne, QC J6V 2H2, CANADA |  |
|  | Institutional Review Board Services, 372 Hollandview Trail, Ste. 300, Aurora, ON L4G 0A5, CANADA |
| Research Ethics Board of the McGill University Health Centre, 1650 Cedar Ave., Ste. C10.148.2, Montreal, QC H3G 1A4, CANADA |  |
| SSS du Nord de Lanaudiere-Centre hospitalier regional de lanaudiere, Comte d’ethique a la recherche, 1000 Boul Sainte Anne, Saint-Charles Borromee, QC J6E 6J2,  CANADA |  |
| Niagara Health System Research Ethics Board,260 Sugar Loaf St., Port Colborne, ON L3K 2N7, CANADA |  |
| Comite d’ethique de la recherche en sante chez l’humain du CHUS – CIUSSS de I’Estrie – CHUS – Hopital Fleurimont ,CRCHUS, Hopital Fleurimont, 3001 12 e Ave. Nord., Sherbrooke, QC J1H 5N4, CANADA |  |
| Comite d’ethique de la recherche du Centre integre de sante et de services sociaux de chaudiere-Appalaches 143 Rue Wolfe, Levis,QC G6W 3Z1 CANADA |  |
| Centre integre universitaire de sante et de services sociaux de la Mauricie-et-du-Centre-du-Quebec Comite D’Ethique de la Recherche, 1991 Boul Du Carmel, Trois-Rivieres, QC G8Z 3R9, CANADA |  |
| Hamilton Integrated Research Ethics Board, 293 Wellington St., N, Ste.102, Hamilton, ON L8L 8E7,  CANADA |  |
|  | Institutional Review Board Services, 372 Hollandview Trail, Ste. 300, Aurora, ON L4G 0A5, CANADA |
| Centre De Sante Et De Services Sociaux de Saint-Jerome, 290 Montigny, St. Jerome, QC J7Z 5T3,  CANADA |  |
| University of Kelaniya - EC  Faculty of Medicine,  P.O.BOX 6. Thalagolla Road  Ragama  11600  SRI LANKA | University of Kelaniya - EC  Faculty of Medicine,  P.O.BOX 6. Thalagolla Road  Ragama  11600  SRI LANKA |
| University of Kelaniya - EC  Faculty of Medicine,  P.O.BOX 6. Thalagolla Road  Ragama  11600  SRI LANKA | University of Kelaniya - EC  Faculty of Medicine,  P.O.BOX 6. Thalagolla Road  Ragama  11600  SRI LANKA |
| University of Kelaniya - EC  Faculty of Medicine,  P.O.BOX 6. Thalagolla Road  Ragama  11600  SRI LANKA | University of Kelaniya - EC  Faculty of Medicine,  P.O.BOX 6. Thalagolla Road  Ragama  11600  SRI LANKA |
| University of Kelaniya - EC  Faculty of Medicine,  P.O.BOX 6. Thalagolla Road  Ragama  11600  SRI LANKA | University of Kelaniya - EC  Faculty of Medicine,  P.O.BOX 6. Thalagolla Road  Ragama  11600  SRI LANKA |
| University of Kelaniya - EC  Faculty of Medicine,  P.O.BOX 6. Thalagolla Road  Ragama  11600  SRI LANKA | University of Kelaniya - EC  Faculty of Medicine,  P.O.BOX 6. Thalagolla Road  Ragama  11600  SRI LANKA |
| University of Kelaniya - EC  Faculty of Medicine,  P.O.BOX 6. Thalagolla Road  Ragama  11600  SRI LANKA | University of Kelaniya - EC  Faculty of Medicine,  P.O.BOX 6. Thalagolla Road  Ragama  11600  SRI LANKA |
| University of Kelaniya - EC  Faculty of Medicine,  P.O.BOX 6. Thalagolla Road  Ragama  11600  SRI LANKA | University of Kelaniya - EC  Faculty of Medicine,  P.O.BOX 6. Thalagolla Road  Ragama  11600  SRI LANKA |
| University of Kelaniya - EC  Faculty of Medicine,  P.O.BOX 6. Thalagolla Road  Ragama  11600  SRI LANKA | University of Kelaniya - EC  Faculty of Medicine,  P.O.BOX 6. Thalagolla Road  Ragama  11600  SRI LANKA |
| University of Kelaniya - EC  Faculty of Medicine,  P.O.BOX 6. Thalagolla Road  Ragama  11600  SRI LANKA | University of Kelaniya - EC  Faculty of Medicine,  P.O.BOX 6. Thalagolla Road  Ragama  11600  SRI LANKA |
| University of Kelaniya - EC  Faculty of Medicine,  P.O.BOX 6. Thalagolla Road  Ragama  11600  SRI LANKA | University of Kelaniya - EC  Faculty of Medicine,  P.O.BOX 6. Thalagolla Road  Ragama  11600  SRI LANKA |
| Comité de Ética Científico S.S. M Oriente  Av . Salvador # 364  Providencia  Santiago  7500922  RM Region Metropolitan  CHILE |  |
| Comite de Etica de la Investigacion S.S Araucania Sur  Andres Bello 636  Temuco  IX Region de la Araucanía  CHILE |  |
| Comite Etico de Investigacion Servicio de Salud Valdivia  Vicente Perez Rosales #560 of 307  Edificio Prales  Valdivia 5110537  XIV Region de los Rios  CHILE |  |
| Comité de Ética Científico S.S. M Oriente  Av . Salvador # 364  Providencia  Santiago 7500922  RM Region Metropolitan  CHILE |  |
| Comite de Etica de la Investigacion S.S.M. Norte  San Jose # 1053  Independencia  Santiago  8380755  RM Region Metropolitan  CHILE |  |
| Comité de Ética Científico S.S. M Oriente  Av . Salvador # 364  Providencia  Santiago 7500922  RM Region Metropolitan  CHILE |  |
| Comite Etico de Investigacion Servicio de Salud Valdivia  Vicente Perez Rosales #560 of 307  Edificio Prales  Valdivia 5110537  XIV Region de los Rios  CHILE |  |
| comite de etica cientifica del servicio de salud del maule  1 Norte 963 Edificio centro 2000, 4° piso  Talca  VII Region del Maule  CHILE |  |
| Comite de Etica de la Investigacion S.S. Viña Quillota  Limache # 1307  Viña del Mar  2520563  V Region de Valparaiso  CHILE |  |
| Comite de Etica de la Investigacion S.S.M Sur Oriente  Av. Concha y Toro # 3459  Puente Alto  Santiago  7710000  RM Region Metropolitan  CHILE |  |
| Comite de Etica de la Investigacion S.S.M. Sur  Av. Santa Rosa # 3453  Santiago8900390  RM Region Metropolitan  CHILE |  |
| SECT REG MINISTERIAL DE SALUD COMITE ETICO CIENTIFICO CLINIC  Caupolican # 518 Piso 5 oficina 510  Concepcion  VIII Region del Bio-Bio  CHILE |  |
| Comité de Ética Científico S.S. M Oriente  Av . Salvador # 364  Providencia  Santiago 7500922  RM Region Metropolitan  CHILE |  |
| Comite de Etica de la Investigacion S.S.M Central  Victoria subercaseaux 381, 5° piso, Santiago Centro  Santiago 8360160  RM Region Metropolitan  CHILE |  |
| Comite de Etica de la Investigacion S.S.M Sur Oriente  Av. Concha y Toro # 3459  Puente Alto  Santiago  7710000  RM Region Metropolitan  CHILE |  |
| Comite Etico de Investigacion Servicio de Salud Valdivia  Vicente Perez Rosales #560 of 307  Edificio Prales  Valdivia  5110537  XIV Region de los Rios  CHILE |  |
| Comite de Etica de la Investigacion S.S. Viña Quillota  Limache # 1307  Viña del Mar  Vina Del Mar  2520563  V Region de Valparaiso  CHILE |  |
| Comité de Ética Científico S.S. M Oriente  Av . Salvador # 364  Providencia  Santiago 7500922  RM Region Metropolitan  CHILE |  |
| IEC of Peking University First Hospital  No 8, Xishiku Street,Xicheng District  Beijing 100034  CHINA |  |
| IEC of Chinese PLA General Hospital  No.28, Fuxing Road  Beijing 100853  CHINA |  |
| IEC of Beijing Tongren Hospital  No. 1, Dongjiaominxiang  Beijing 100730  CHINA |  |
| EIC of Beijing Chaoyang Hospital  No 8,Gongti nanlu,Chaoyang District,  Beijing100020  CHINA |  |
| IEC of Chinese PLA General Hospital  No.28, Fuxing Road  Beijing 100853  CHINA |  |
| IEC of Beijing Anzhen Hospital of Capital Medical University  No. 2, Anzhen road, Beijing 100029  CHINA |  |
| IEC of The Central Hospital of China Aerospace Corporation  No.15, Yuquan Road, Haidian District  Beijing 100049  CHINA |  |
| IEC of First Hospital, Jilin University  No.71, Xinmin Street  Changchun 130021  CHINA |  |
| IEC of Yanbian University Hospital  No.1327, Juzi Street,  Jilin Province  Yanji 133000  CHINA |  |
| IEC of First Hospital of China Medical University  No.155, Najing Street(north)  Shenyang 110001  CHINA |  |
| IEC of Shengjing Hospital of China Medical University  No.36 Sanhao Street  Shenyang 110004  CHINA |  |
| IEC of Inner Mongolia People's Hospital  No.20, Zhaowuda Road, Hohhot  Inner Mongolia  Hohhot 010017  CHINA |  |
| The First Affiliated Hospital of Baotou Medical College  No.41, Linyin Road, Kundulun District  Baotou 014010  CHINA |  |
| IEC of TEDA International Cardiovascular Hospital  NO.61 Third Road, Economic and Technological Development Zone  Tianjin 300457  CHINA |  |
| IEC of The First Hospital of Lanzhou University  No.1,Donggang West Road, Chengguan District  Lanzhou  730000  CHINA |  |
| IEC of 1st Affiliated Hospital of Xi'an Jiaotong University  No.277, Yanta West Road, Yanta District, Xi'an City 710061  CHINA |  |
| IEC of Second Hospital of Shandong University  No.247, Beiyuan Street  Jinan 250033  CHINA |  |
| IEC of Jinan Central Hospital  No.105 Jiefang road，Shandong  Jinan 250013  CHINA |  |
| IEC of DrumTower Hospital of Nanjing University Medical School  No.321, Zhongshan Road  Nanjing 210008  CHINA |  |
| IEC of Zhongda Hospital, Southeast University  IEC of Zhongda Hospital  No.87 Dingjiaqiao, Jiangsu province  Nanjing  210009  CHINA |  |
| IEC of The First Affiliated Hospital of Soochow University  No.188,Shizi Street  Suzhou  215006  CHINA |  |
| IEC of Shanghai East Hospital  No1800,Yuntai Road,Pudong New Area  Shanghai 200120  CHINA |  |
| IEC of Ruijin Hospital Affiliated to Shanghai Jiaotong University  No.197,Ruijin 2nd Road  Shanghai 200020  CHINA |  |
| Independent Ethics Committee of Shanghai Sixth People's Hosp  No.600, Yishan Road  Shanghai  200233  CHINA |  |
| IEC of Shanghai First People's Hospital  No. 100, Haining Road  Shanghai  200080  CHINA |  |
| IEC of Zhongshan Hospital Fudan University  No.180, Fenglin Road  Shanghai  200032  CHINA |  |
| IEC of Sir Run Run Shaw Hospital  No.3,Qingchun Road (East)  Hangzhou  310016  CHINA |  |
| IEC of First Affiliated Hospital of Zhejiang University  No.79,Qingchun Road  Hangzhou  310003  CHINA |  |
| IEC of Second Affiliated Hospital of Zhejiang University  No.88,Jiefang Road  Hangzhou  310009  CHINA |  |
| IEC of Wuhan Asia Heart Hosp.  No.753, Jinghan Avenue, Wuhan, Hubei Province  Wuhan  430022  CHINA |  |
| IEC of Hunan Provincial People's Hospital  No.61, West Jiefang Road  Changsha  410005  CHINA |  |
| IEC of Second Xiangya Hospital of Central South University  No.139,Renmin Road (middle)  Changsha 410011  CHINA |  |
| IEC of 1st Hosp. Nanchang Univ  No.17, Yongwaizheng Street, Nanchang, Jiangxi Province  Nanchang 330006  CHINA |  |
| IEC of The Second Affiliated Hospital to Nanchang University  IEC of 2nd Hosp. Nanchang Univ  No. 1 Minde Road, Nanchang, Jiangxi Province  Nanchang  330006  CHINA |  |
| IEC of Xinqiao Hospital of Third Military Medical University  Xinqiaozheng Street  Chongqing 400037  CHINA |  |
| IEC of the First Hospital of Sun Yat-Sen University  Room 110, Longzhu Building, No 5, Zhusigang Erma Road  Guangzhou  510080  CHINA |  |
| IEC of Guangdong General Hospital  No. 106, Zhongshan Er Road  Guangzhou  510080  CHINA |  |
| IEC of Nanfang Hospital  No.1838,Guangzhou Avenue (North)  Guangzhou 510515  CHINA |  |
| IEC of Xuzhou Medical College Hospital  No.99, West Huaihai Road, Xuzhou, Jiangsu Province; Xuzhou  221002  CHINA |  |
| IEC of Siping Central People's Hospital  No.89, South Yingbin Street, Tiexie Dsitrict  Siping136000  CHINA |  |
| IEC of Jilin Province People's Hospital  No.1183, Gongnong Street  Jilin Province  Changchun 130021  CHINA |  |
| IEC of First Affiliated Hospital of Wenzhou Medical College  NanBaixiang, Ouhai District  Wenzhou, Zhejiang Province  Wenzhou 325000  CHINA |  |
| IEC of Tianjin Medical University General Hospital  No 154,Anshan Road, Heping district, tianjin  Tianjin  300052  CHINA |  |
| IEC of Tianjin Union Medicine Centre  No.190, Jieyuan Road, HongqiaoDistrict  Tianjin  300121  CHINA |  |
| IEC of Zhejiang Hospital  Floor 2, Building 11, No. 12, Lingyin Road  Hangzhou  310013  CHINA |  |
| IEC First Affiliated Hospital of Fujian Medical University  No.20 Chazhong Road, Taijiang District, Fuzhou, Fujian Province  Fuzhou  350005  CHINA |  |
| IEC of Sun Yat-Sen Memorial Hospital  No.107,Yanjiang Road (West)  Guangzhou  510120  CHINA |  |
| IEC of Guizhou Provincial People‘s Hospital  No. 83, Zhongshan East Road, Guiyang, Guizhou Province  Guiyang 550002  CHINA |  |
| IEC of Peking University Shenzhen Hospital  No.1120, Lianhua Road, Futian District  Shenzhen 518036  CHINA |  |
| IEC for Clinical Drug Trials of Lanzhou University Second Hospital  No.82,Cuiyingmen, Linxia Road, Chengguan District  Lanzhou  730030  CHINA |  |
| IEC of First People's Hospital of Yueyang  No.39 Dongmaoling Road  Yueyang 414000  CHINA |  |
| IEC of Shanghai Chest Hospital  No 241, West Huaihai Road  Shanghai 200030  CHINA |  |
| IEC of Hangzhou First People's Hospital  No.261, Huansha Road, Shangcheng District  Hangzhou, Zhejiang Province  Hangzhou  310006  CHINA |  |
| IEC of Zhejiang Provincial People’s Hospital  No 158, Shangtang Road, Zhejiang Province  Hangzhou  310014  CHINA |  |
| IEC of Fuzhou General Hospital of Nanjing Military Command  No.156, Xierhuan Road (North)  Fuzhou  350025  CHINA |  |
| IEC of Affiliated Hospital of Guangdong Medical University  No.57, South Renming Avenue, Xiashan District  Zhanjiang  524001  CHINA |  |
| IEC of Affiliated Hospital of Southwest Medical University  No.25, Taiping Street, Jiangyang District  Luzhou, Sichuan Province  Luzhou  646000  CHINA |  |
| IEC Navy General Hospital  No. 6, Fu Cheng road, Haidian District  Beijing  100048  CHINA |  |
| IEC of Tianjin 4th Centre Hospital  No.1 Zhongshan Road, Hebei District  Tianjin  300140  CHINA |  |
| IEC of Peking University Shenzhen Hospital  No.1120, Lianhua Road, Futian District  Shenzhen  518036  CHINA |  |
| IEC of Shaanxi Provincial People's Hospital  No.256 West Youyi Road  Xi'an, Shaanxi Province  Xi'An  710068  CHINA |  |
| IEC of The Third People’s Hospital of Hubei Province  No.26, Zhongshan Avenue  Wuhan, Hubei Province  Wuhan  430033 CHINA |  |
| IEC of Third Xiangya Hospital of Central South University  No.138, Tongzipo Road  Changsha  410013  CHINA |  |
| Veterans General Hospital-Taipei(Institutional Review Board)  Medical Research & Education  3F, Chih-The Medical Research Building  No. 201, Sec. 2, Shih-Pai Road  Taipei  11217  TAIWAN, PROVINCE OF CHINA |  |
| National Taiwan University Hospital - Yunlin  No. 579, Sec2, Yunlin Raod  Yunlin  TAIWAN, PROVINCE OF CHINA |  |
| Changhua Christian Hospital  135, Nan-Hsiao Street  Changhua  500  TAIWAN, PROVINCE OF CHINA |  |
| Chung-Shan Medical University Hospital  Department of Cardiology  No. 110, Sec.1, Chien-Kuo North Road  2f Cardiac Catherization Room Taichung  402 TAIWAN, PROVINCE OF CHINA |  |
| Mackay Memorial Hospital Institutional Review Board  No. 92, Sec. 2, Zhongshan N Rd., Zhongshan Dist.  Taipei  10449  TAIWAN, PROVINCE OF CHINA |  |
| Cheng Hsin General Hospital (Institutional Review Board)  No.45, Cheng Hsin St.,Pai-Tou  Taipei  TAIWAN, PROVINCE OF CHINA |  |
| Chang Gung Memorial Hospital (Institutional Review Board)  No5, Fu-Hsing street, Guei Shan Street  Taoyuan  TAIWAN, PROVINCE OF CHINA |  |
| National Cheng Kung University Hospital  138 Sheng-Li Road  Tainan  704  TAIWAN, PROVINCE OF CHINA |  |
| Tri Service General Hospital (IRB)  No.325, Sec.2 Cheng-Kung Road  Taipei  11490  TAIWAN, PROVINCE OF CHINA |  |
| Chi-Mei  No. 901 Chung-Hwa Rd. Yung-Kang City  TAIWAN, PROVINCE OF CHINA |  |
| Chang Gung Memorial Hospital (Institutional Review Board)  No5, Fu-Hsing street, Guei Shan Street  Taoyuan  TAIWAN, PROVINCE OF CHINA |  |
| IRB, Kaohsiung Medical University Chung-Ho Memorial Hospital  8F, Building A, No. 100 Tzyou 1st Rd  Kaohsiung  807  TAIWAN, PROVINCE OF CHINA |  |
| Mackay Memorial Hospital Institutional Review Board  No. 92, Sec. 2, Zhongshan N Rd., Zhongshan Dist.  Taipei  10449  TAIWAN, PROVINCE OF CHINA |  |
| C.de E. en Inv. Biomédica Centro Medico Julián Coronel  Carrera 59 N° 1E - 21  Cali-Valle  COLOMBIA |  |
| Comité de Ética en la Investigación CAIMED  Carrera 42 A No. 17 – 50  Bogotá  111611  COLOMBIA |  |
| Comite de etica en investigacion clinica de la costa ltda  Carrera 50 # 80-90 Barranquilla, Atlántico - Colombia  COLOMBIA |  |
| Centro de diagnostico cardiologico LTDA  Calle 5 No 6-47 Castillo Grande  Cartagena NA  COLOMBIA |  |
| Comite de Etica en Investigación FOSCAL  Cll 49 No. 27a-74  Floridablanca  COLOMBIA |  |
| Comité de Ética en la Investigación CAIMED  Carrera 42 A No. 17 – 50  Bogotá  111611  COLOMBIA |  |
| Comité de Revisión de Estudios de Investigación CREI  Carrera 43A# 34-155 Piso 6  Medellin  050015  COLOMBIA |  |
| Comité de Ética en Investigación de RIESCARD  Carrera 5 No.18-70 Barrio Libertador  Espinal  COLOMBIA |  |
| Comité de Ética e Investigación de CEMDE  Calle 33A #70A-175  Medellín  COLOMBIA |  |
| Comite de Etica en Investigacion del Centro Medico Imbanaco  Carrera 38 #5a -100  Torre B - Piso 11  Cali  COLOMBIA |  |
| CEI de la Fundación del Caribe para la Investigación  Cr. 50 No. 80-216  Barranquilla COLOMBIA |  |
| CEI de la Fundación del Caribe para la Investigación  Cr. 50 No. 80-216  Barranquilla  COLOMBIA |  |
| Comité Institucional de Ética de Investigación de CAFAM  Av. Cra. 68 # 90 – 88 Bloque 5, piso 4  Bogotá  COLOMBIA |  |
| Comité de Ética Centro Cardiovascular Santa Lucia  Carrera 71 N° 71 - 31 Cartagena-Bolívar  COLOMBIA |  |
| CE en Investigacion del Hospital Santa Clara E.S.E.  Carrera 15 No. 1 - 59 Sur Bogotá  COLOMBIA |  |
| Comité de Ética en Investigación, Clínica CARDIO VID  Calle 78 B # 75 – 21, Medellín  COLOMBIA |  |
| C. de E. de la Inv. - Riesgo de Fractura- SA  Carrera 13 # 97 – 25, Bogotá  110221037  COLOMBIA |  |
|  | Central Ethics Committee  Agencija za lijekove i medicinske proizvode  Ksaverska cesta 4  Zagreb  10000  CROATIA |
|  | Central Ethics Committee  Agencija za lijekove i medicinske proizvode  Ksaverska cesta 4  Zagreb  10000  CROATIA |
|  | Central Ethics Committee  Agencija za lijekove i medicinske proizvode  Ksaverska cesta 4  Zagreb  10000  CROATIA |
|  | Central Ethics Committee  Agencija za lijekove i medicinske proizvode  Ksaverska cesta 4  Zagreb  10000  CROATIA |
|  | Central Ethics Committee  Agencija za lijekove i medicinske proizvode  Ksaverska cesta 4  Zagreb  10000  CROATIA |
|  | Central Ethics Committee  Agencija za lijekove i medicinske proizvode  Ksaverska cesta 4  Zagreb  10000  CROATIA |
|  | Central Ethics Committee  Agencija za lijekove i medicinske proizvode  Ksaverska cesta 4  Zagreb  10000  CROATIA |
|  | Central Ethics Committee  Agencija za lijekove i medicinske proizvode  Ksaverska cesta 4  Zagreb  10000  CROATIA |
|  | Central Ethics Committee  Agencija za lijekove i medicinske proizvode  Ksaverska cesta 4  Zagreb  10000  CROATIA |
|  | Central Ethics Committee  Agencija za lijekove i medicinske proizvode  Ksaverska cesta 4  Zagreb  10000  CROATIA |
|  | Central Ethics Committee  Agencija za lijekove i medicinske proizvode  Ksaverska cesta 4  Zagreb  10000  CROATIA |
|  | Central Ethics Committee  Agencija za lijekove i medicinske proizvode  Ksaverska cesta 4  Zagreb  10000  CROATIA |
|  | Central Ethics Committee  Agencija za lijekove i medicinske proizvode  Ksaverska cesta 4  Zagreb  10000  CROATIA |
|  | Central Ethics Committee  Agencija za lijekove i medicinske proizvode  Ksaverska cesta 4  Zagreb  10000  CROATIA |
|  | Central Ethics Committee  Agencija za lijekove i medicinske proizvode  Ksaverska cesta 4  Zagreb  10000  CROATIA |
|  | Central Ethics Committee  Agencija za lijekove i medicinske proizvode  Ksaverska cesta 4  Zagreb  10000  CROATIA |
| Eticka komise FN Brno  Jihlavska 20  Brno  62500  CZECH REPUBLIC | EK pro multicentrické klinické hodnocení FN v Motole  V uvalu 84  Praha 5  15006  CZECH REPUBLIC |
| EK při IKEM a Thomayerově nemocnici  Videnska 800  Praha 4 - Krc  14059  CZECH REPUBLIC | EK pro multicentrické klinické hodnocení FN v Motole  V uvalu 84  Praha 5  15006  CZECH REPUBLIC |
| EK pro multicentrické klinické hodnocení FN v Motole  V uvalu 84  Praha 5  15006  CZECH REPUBLIC | EK pro multicentrické klinické hodnocení FN v Motole  V uvalu 84  Praha 5  15006  CZECH REPUBLIC |
| Eticka komise Fakultni nemocnice Hradec Kralove  Sokolska 581  Hradec Kralove  500 05  CZECH REPUBLIC | EK pro multicentrické klinické hodnocení FN v Motole  V uvalu 84  Praha 5  15006  CZECH REPUBLIC |
| EK pro multicentrické klinické hodnocení FN v Motole  V uvalu 84  Praha 5  15006  CZECH REPUBLIC | EK pro multicentrické klinické hodnocení FN v Motole  V uvalu 84  Praha 5  15006  CZECH REPUBLIC |
| Eticka komise Nemocnice Kromeriz, a.s.  Havlickova 660/69  Kromeriz  767 55  CZECH REPUBLIC | EK pro multicentrické klinické hodnocení FN v Motole  V uvalu 84  Praha 5  15006  CZECH REPUBLIC |
| EK pro multicentrické klinické hodnocení FN v Motole  V uvalu 84  Praha 5  15006  CZECH REPUBLIC | EK pro multicentrické klinické hodnocení FN v Motole  V uvalu 84  Praha 5  15006  CZECH REPUBLIC |
| Eticka komise Krajske nemocnice T. Bati, a.s.  Havlickovo nabrezi. 600  Zlin  76275  CZECH REPUBLIC | EK pro multicentrické klinické hodnocení FN v Motole  V uvalu 84  Praha 5  15006  CZECH REPUBLIC |
| EK FN Královské Vinohrady  FN Kralovske Vinohrady  Srobarova 1150/50  Praha 10  10034  CZECH REPUBLIC | EK pro multicentrické klinické hodnocení FN v Motole  V uvalu 84  Praha 5  15006  CZECH REPUBLIC |
| Eticka komise Nemocnice Na Homolce  Roentgenova 2  Praha 5  15030  CZECH REPUBLIC | EK pro multicentrické klinické hodnocení FN v Motole  V uvalu 84  Praha 5  15006  CZECH REPUBLIC |
| EK FN Olomouc a Lékařské fakutly UP v Olomouci  I. P. Pavlova 6  Olomouc  77520  CZECH REPUBLIC | EK pro multicentrické klinické hodnocení FN v Motole  V uvalu 84  Praha 5  15006  CZECH REPUBLIC |
| Etická komise Nemocnice Pardubického kraje, a.s.  Pardubicka nemocnice  Kyjevska 44  Pardubice  532 03  CZECH REPUBLIC | EK pro multicentrické klinické hodnocení FN v Motole  V uvalu 84  Praha 5  15006  CZECH REPUBLIC |
| EK pro multicentrická hodnocení KN Liberec, a.s.  Husova 10  Liberec  46063  CZECH REPUBLIC | EK pro multicentrické klinické hodnocení FN v Motole  V uvalu 84  Praha 5  15006  CZECH REPUBLIC |
| EK Mestska nemocnice Ostrava  Nemocnicni 20  Ostrava  72880  CZECH REPUBLIC | EK pro multicentrické klinické hodnocení FN v Motole  V uvalu 84  Praha 5  15006  CZECH REPUBLIC |
| Eticka komise Vseobecne FN v Praze  Na Bojisti 1  Praha 2  12808  CZECH REPUBLIC | EK pro multicentrické klinické hodnocení FN v Motole  V uvalu 84  Praha 5  15006  CZECH REPUBLIC |
| Eticka komise FN Plzen  E. Benese 13  Plzen  30599  CZECH REPUBLIC | EK pro multicentrické klinické hodnocení FN v Motole  V uvalu 84  Praha 5  15006  CZECH REPUBLIC |
| Eticka komise Oblastni nemocnice Kladno  Vancurova 1548  Kladno  27201  CZECH REPUBLIC | EK pro multicentrické klinické hodnocení FN v Motole  V uvalu 84  Praha 5  15006  CZECH REPUBLIC |
| EK pro multicentrické klinické hodnocení FN v Motole  V uvalu 84  Praha 5  15006  CZECH REPUBLIC | EK pro multicentrické klinické hodnocení FN v Motole  V uvalu 84  Praha 5  15006  CZECH REPUBLIC |
| EK při IKEM a Thomayerově nemocnici  Videnska 800  Praha 4 - Krc  14059  CZECH REPUBLIC | EK pro multicentrické klinické hodnocení FN v Motole  V uvalu 84  Praha 5  15006  CZECH REPUBLIC |
| EK pro multicentrické klinické hodnocení FN v Motole  V uvalu 84  Praha 5  15006  CZECH REPUBLIC | EK pro multicentrické klinické hodnocení FN v Motole  V uvalu 84  Praha 5  15006  CZECH REPUBLIC |
| EK pro multicentrické klinické hodnocení FN v Motole  V uvalu 84  Praha 5  15006  CZECH REPUBLIC | EK pro multicentrické klinické hodnocení FN v Motole  V uvalu 84  Praha 5  15006  CZECH REPUBLIC |
| EK pro multicentrické klinické hodnocení FN v Motole  V uvalu 84  Praha 5  15006  CZECH REPUBLIC | EK pro multicentrické klinické hodnocení FN v Motole  V uvalu 84  Praha 5  15006  CZECH REPUBLIC |
| De Videnskabsetiske Komitéer for Region Hovedstaden  Regionsgården  Kongens Vænge 2  Hillerød  3400  DENMARK | De Videnskabsetiske Komitéer for Region Hovedstaden  Regionsgården  Kongens Vænge 2  Hillerød  3400  DENMARK |
| De Videnskabsetiske Komitéer for Region Hovedstaden  Regionsgården  Kongens Vænge 2  Hillerød  3400  DENMARK | De Videnskabsetiske Komitéer for Region Hovedstaden  Regionsgården  Kongens Vænge 2  Hillerød  3400  DENMARK |
| De Videnskabsetiske Komitéer for Region Hovedstaden  Regionsgården  Kongens Vænge 2  Hillerød  3400  DENMARK | De Videnskabsetiske Komitéer for Region Hovedstaden  Regionsgården  Kongens Vænge 2  Hillerød  3400  DENMARK |
| De Videnskabsetiske Komitéer for Region Hovedstaden  Regionsgården  Kongens Vænge 2  Hillerød  3400  DENMARK | De Videnskabsetiske Komitéer for Region Hovedstaden  Regionsgården  Kongens Vænge 2  Hillerød  3400  DENMARK |
| De Videnskabsetiske Komitéer for Region Hovedstaden  Regionsgården  Kongens Vænge 2  Hillerød  3400  DENMARK | De Videnskabsetiske Komitéer for Region Hovedstaden  Regionsgården  Kongens Vænge 2  Hillerød  3400  DENMARK |
| De Videnskabsetiske Komitéer for Region Hovedstaden  Regionsgården  Kongens Vænge 2  Hillerød  3400  DENMARK | De Videnskabsetiske Komitéer for Region Hovedstaden  Regionsgården  Kongens Vænge 2  Hillerød  3400  DENMARK |
| De Videnskabsetiske Komitéer for Region Hovedstaden  Regionsgården  Kongens Vænge 2  Hillerød  3400  DENMARK | De Videnskabsetiske Komitéer for Region Hovedstaden  Regionsgården  Kongens Vænge 2  Hillerød  3400  DENMARK |
| De Videnskabsetiske Komitéer for Region Hovedstaden  Regionsgården  Kongens Vænge 2  Hillerød  3400  DENMARK | De Videnskabsetiske Komitéer for Region Hovedstaden  Regionsgården  Kongens Vænge 2  Hillerød  3400  DENMARK |
| De Videnskabsetiske Komitéer for Region Hovedstaden  Regionsgården  Kongens Vænge 2  Hillerød  3400  DENMARK | De Videnskabsetiske Komitéer for Region Hovedstaden  Regionsgården  Kongens Vænge 2  Hillerød  3400  DENMARK |
| De Videnskabsetiske Komitéer for Region Hovedstaden  Regionsgården  Kongens Vænge 2  Hillerød  3400  DENMARK | De Videnskabsetiske Komitéer for Region Hovedstaden  Regionsgården  Kongens Vænge 2  Hillerød  3400  DENMARK |
| De Videnskabsetiske Komitéer for Region Hovedstaden  Regionsgården  Kongens Vænge 2  Hillerød  3400  DENMARK | De Videnskabsetiske Komitéer for Region Hovedstaden  Regionsgården  Kongens Vænge 2  Hillerød  3400  DENMARK |
| De Videnskabsetiske Komitéer for Region Hovedstaden  Regionsgården  Kongens Vænge 2  Hillerød  3400  DENMARK | De Videnskabsetiske Komitéer for Region Hovedstaden  Regionsgården  Kongens Vænge 2  Hillerød  3400  DENMARK |
| De Videnskabsetiske Komitéer for Region Hovedstaden  Regionsgården  Kongens Vænge 2  Hillerød  3400  DENMARK | De Videnskabsetiske Komitéer for Region Hovedstaden  Regionsgården  Kongens Vænge 2  Hillerød  3400  DENMARK |
| De Videnskabsetiske Komitéer for Region Hovedstaden  Regionsgården  Kongens Vænge 2  Hillerød  3400  DENMARK | De Videnskabsetiske Komitéer for Region Hovedstaden  Regionsgården  Kongens Vænge 2  Hillerød  3400  DENMARK |
| De Videnskabsetiske Komitéer for Region Hovedstaden  Regionsgården  Kongens Vænge 2  Hillerød  3400  DENMARK | De Videnskabsetiske Komitéer for Region Hovedstaden  Regionsgården  Kongens Vænge 2  Hillerød  3400  DENMARK |
| De Videnskabsetiske Komitéer for Region Hovedstaden  Regionsgården  Kongens Vænge 2  Hillerød  3400  DENMARK | De Videnskabsetiske Komitéer for Region Hovedstaden  Regionsgården  Kongens Vænge 2  Hillerød  3400  DENMARK |
| De Videnskabsetiske Komitéer for Region Hovedstaden  Regionsgården  Kongens Vænge 2  Hillerød  3400  DENMARK | De Videnskabsetiske Komitéer for Region Hovedstaden  Regionsgården  Kongens Vænge 2  Hillerød  3400  DENMARK |
| Tallinn Medical Research Ethics Committee  Hiiu 42  Tallinn  11619  ESTONIA | Tallinn Medical Research Ethics Committee  Hiiu 42  Tallinn  11619  ESTONIA |
| Tallinn Medical Research Ethics Committee  Hiiu 42  Tallinn  11619  ESTONIA | Tallinn Medical Research Ethics Committee  Hiiu 42  Tallinn  11619  ESTONIA |
| Tallinn Medical Research Ethics Committee  Hiiu 42  Tallinn  11619  ESTONIA | Tallinn Medical Research Ethics Committee  Hiiu 42  Tallinn  11619  ESTONIA |
| Tallinn Medical Research Ethics Committee  Hiiu 42  Tallinn  11619  ESTONIA | Tallinn Medical Research Ethics Committee  Hiiu 42  Tallinn  11619  ESTONIA |
| Tallinn Medical Research Ethics Committee  Hiiu 42  Tallinn  11619  ESTONIA | Tallinn Medical Research Ethics Committee  Hiiu 42  Tallinn  11619  ESTONIA |
| Tallinn Medical Research Ethics Committee  Hiiu 42  Tallinn  11619  ESTONIA | Tallinn Medical Research Ethics Committee  Hiiu 42  Tallinn  11619  ESTONIA |
| Tallinn Medical Research Ethics Committee  Hiiu 42  Tallinn  11619  ESTONIA | Tallinn Medical Research Ethics Committee  Hiiu 42  Tallinn  11619  ESTONIA |
| ETENE/TUKIJA, Sosiaali- ja terveysministeriö  Lintulahdenkuja 4  Helsinki  00530  FINLAND | ETENE/TUKIJA, Sosiaali- ja terveysministeriö  Lintulahdenkuja 4  Helsinki  00530  FINLAND |
| ETENE/TUKIJA, Sosiaali- ja terveysministeriö  Lintulahdenkuja 4  Helsinki  00530  FINLAND | ETENE/TUKIJA, Sosiaali- ja terveysministeriö  Lintulahdenkuja 4  Helsinki  00530  FINLAND |
| ETENE/TUKIJA, Sosiaali- ja terveysministeriö  Lintulahdenkuja 4  Helsinki  00530  FINLAND | ETENE/TUKIJA, Sosiaali- ja terveysministeriö  Lintulahdenkuja 4  Helsinki  00530  FINLAND |
| ETENE/TUKIJA, Sosiaali- ja terveysministeriö  Lintulahdenkuja 4  Helsinki  00530  FINLAND | ETENE/TUKIJA, Sosiaali- ja terveysministeriö  Lintulahdenkuja 4  Helsinki  00530  FINLAND |
|  | CPP IDF 10  Hôpital Robert Ballanger  Batiment central n°8 (3ème étage)  boulevard Robert Ballanger  Aulnay-Sous-Bois Cedex  93602  FRANCE |
|  | CPP IDF 10  Hôpital Robert Ballanger  Batiment central n°8 (3ème étage)  boulevard Robert Ballanger  Aulnay-Sous-Bois Cedex  93602  FRANCE |
|  | CPP IDF 10  Hôpital Robert Ballanger  Batiment central n°8 (3ème étage)  boulevard Robert Ballanger  Aulnay-Sous-Bois Cedex  93602  FRANCE |
|  | CPP IDF 10  Hôpital Robert Ballanger  Batiment central n°8 (3ème étage)  boulevard Robert Ballanger  Aulnay-Sous-Bois Cedex  93602  FRANCE |
|  | CPP IDF 10  Hôpital Robert Ballanger  Batiment central n°8 (3ème étage)  boulevard Robert Ballanger  Aulnay-Sous-Bois Cedex  93602  FRANCE |
|  | CPP IDF 10  Hôpital Robert Ballanger  Batiment central n°8 (3ème étage)  boulevard Robert Ballanger  Aulnay-Sous-Bois Cedex  93602  FRANCE |
|  | CPP IDF 10  Hôpital Robert Ballanger  Batiment central n°8 (3ème étage)  boulevard Robert Ballanger  Aulnay-Sous-Bois Cedex  93602  FRANCE |
|  | CPP IDF 10  Hôpital Robert Ballanger  Batiment central n°8 (3ème étage)  boulevard Robert Ballanger  Aulnay-Sous-Bois Cedex  93602  FRANCE |
|  | CPP IDF 10  Hôpital Robert Ballanger  Batiment central n°8 (3ème étage)  boulevard Robert Ballanger  Aulnay-Sous-Bois Cedex  93602  FRANCE |
|  | CPP IDF 10  Hôpital Robert Ballanger  Batiment central n°8 (3ème étage)  boulevard Robert Ballanger  Aulnay-Sous-Bois Cedex  93602  FRANCE |
|  | CPP IDF 10  Hôpital Robert Ballanger  Batiment central n°8 (3ème étage)  boulevard Robert Ballanger  Aulnay-Sous-Bois Cedex  93602  FRANCE |
|  | CPP IDF 10  Hôpital Robert Ballanger  Batiment central n°8 (3ème étage)  boulevard Robert Ballanger  Aulnay-Sous-Bois Cedex  93602  FRANCE |
|  | CPP IDF 10  Hôpital Robert Ballanger  Batiment central n°8 (3ème étage)  boulevard Robert Ballanger  Aulnay-Sous-Bois Cedex  93602  FRANCE |
|  | CPP IDF 10  Hôpital Robert Ballanger  Batiment central n°8 (3ème étage)  boulevard Robert Ballanger  Aulnay-Sous-Bois Cedex  93602  FRANCE |
|  | CPP IDF 10  Hôpital Robert Ballanger  Batiment central n°8 (3ème étage)  boulevard Robert Ballanger  Aulnay-Sous-Bois Cedex  93602  FRANCE |
|  | CPP IDF 10  Hôpital Robert Ballanger  Batiment central n°8 (3ème étage)  boulevard Robert Ballanger  Aulnay-Sous-Bois Cedex  93602  FRANCE |
|  | CPP IDF 10  Hôpital Robert Ballanger  Batiment central n°8 (3ème étage)  boulevard Robert Ballanger  Aulnay-Sous-Bois Cedex  93602  FRANCE |
|  | CPP IDF 10  Hôpital Robert Ballanger  Batiment central n°8 (3ème étage)  boulevard Robert Ballanger  Aulnay-Sous-Bois Cedex  93602  FRANCE |
|  | CPP IDF 10  Hôpital Robert Ballanger  Batiment central n°8 (3ème étage)  boulevard Robert Ballanger  Aulnay-Sous-Bois Cedex  93602  FRANCE |
|  | CPP IDF 10  Hôpital Robert Ballanger  Batiment central n°8 (3ème étage)  boulevard Robert Ballanger  Aulnay-Sous-Bois Cedex  93602  FRANCE |
|  | CPP IDF 10  Hôpital Robert Ballanger  Batiment central n°8 (3ème étage)  boulevard Robert Ballanger  Aulnay-Sous-Bois Cedex  93602  FRANCE |
|  | CPP IDF 10  Hôpital Robert Ballanger  Batiment central n°8 (3ème étage)  boulevard Robert Ballanger  Aulnay-Sous-Bois Cedex  93602  FRANCE |
| LEC of "Clinic-LJ" LTD  6A, Chechelashvili str.  Kutaisi  4600  GEORGIA |  |
| LEC of Emergency Cardiology Center by Acad.G.Chapidze  4, Lubliana  Tbilisi  0159  GEORGIA |  |
| LEC of Tbilisi Heart and Vascular Clinic  36, Ljubljana str.  Tbilisi  159  GEORGIA |  |
| “Unimed Ajara” Batumi Referral Hospital Local Ethics Commiss  125, Bagrationi  Batumi6010  GEORGIA |  |
| LEC of Aleksandr Aladashvili Clinic  103, Uznadze str.,  Tbilisi  102  GEORGIA |  |
| LEC of Tbilisi Heart and Vascular Clinic  36, Ljubljana str.  Tbilisi  159  GEORGIA |  |
| Center of vascular and heart diseases  5, Lyublyana street  Tbilisi  159 GEORGIA |  |
|  | Ethik-Kommission an der  Medizinischen Fakultät der RWTH Aachen  Pauwelsstraße 30  Aachen  52074  GERMANY |
|  | Ethik-Kommission an der  Medizinischen Fakultät der RWTH Aachen  Pauwelsstraße 30  Aachen  52074  GERMANY |
|  | Ethik-Kommission an der  Medizinischen Fakultät der RWTH Aachen  Pauwelsstraße 30  Aachen  52074  GERMANY |
|  | Ethik-Kommission an der  Medizinischen Fakultät der RWTH Aachen  Pauwelsstraße 30  Aachen  52074  GERMANY |
|  | Ethik-Kommission an der  Medizinischen Fakultät der RWTH Aachen  Pauwelsstraße 30  Aachen  52074  GERMANY |
|  | Ethik-Kommission an der  Medizinischen Fakultät der RWTH Aachen  Pauwelsstraße 30  Aachen  52074  GERMANY |
|  | Ethik-Kommission an der  Medizinischen Fakultät der RWTH Aachen  Pauwelsstraße 30  Aachen  52074  GERMANY |
|  | Ethik-Kommission an der  Medizinischen Fakultät der RWTH Aachen  Pauwelsstraße 30  Aachen  52074  GERMANY |
|  | Ethik-Kommission an der  Medizinischen Fakultät der RWTH Aachen  Pauwelsstraße 30  Aachen  52074  GERMANY |
|  | Ethik-Kommission an der  Medizinischen Fakultät der RWTH Aachen  Pauwelsstraße 30  Aachen  52074  GERMANY |
|  | Ethik-Kommission an der  Medizinischen Fakultät der RWTH Aachen  Pauwelsstraße 30  Aachen  52074  GERMANY |
|  | Ethik-Kommission an der  Medizinischen Fakultät der RWTH Aachen  Pauwelsstraße 30  Aachen  52074  GERMANY |
|  | Ethik-Kommission an der  Medizinischen Fakultät der RWTH Aachen  Pauwelsstraße 30  Aachen  52074  GERMANY |
|  | Ethik-Kommission an der  Medizinischen Fakultät der RWTH Aachen  Pauwelsstraße 30  Aachen  52074  GERMANY |
|  | Ethik-Kommission an der  Medizinischen Fakultät der RWTH Aachen  Pauwelsstraße 30  Aachen  52074  GERMANY |
|  | Ethik-Kommission an der  Medizinischen Fakultät der RWTH Aachen  Pauwelsstraße 30  Aachen  52074  GERMANY |
|  | Ethik-Kommission an der  Medizinischen Fakultät der RWTH Aachen  Pauwelsstraße 30  Aachen  52074  GERMANY |
|  | Ethik-Kommission an der  Medizinischen Fakultät der RWTH Aachen  Pauwelsstraße 30  Aachen  52074  GERMANY |
|  | Ethik-Kommission an der  Medizinischen Fakultät der RWTH Aachen  Pauwelsstraße 30  Aachen  52074  GERMANY |
|  | Ethik-Kommission an der  Medizinischen Fakultät der RWTH Aachen  Pauwelsstraße 30  Aachen  52074  GERMANY |
|  | Ethik-Kommission an der  Medizinischen Fakultät der RWTH Aachen  Pauwelsstraße 30  Aachen  52074  GERMANY |
|  | Ethik-Kommission an der  Medizinischen Fakultät der RWTH Aachen  Pauwelsstraße 30  Aachen  52074  GERMANY |
|  | Ethik-Kommission an der  Medizinischen Fakultät der RWTH Aachen  Pauwelsstraße 30  Aachen  52074  GERMANY |
|  | Ethik-Kommission an der  Medizinischen Fakultät der RWTH Aachen  Pauwelsstraße 30  Aachen  52074  GERMANY |
|  | Ethik-Kommission an der  Medizinischen Fakultät der RWTH Aachen  Pauwelsstraße 30  Aachen  52074  GERMANY |
|  | Ethik-Kommission an der  Medizinischen Fakultät der RWTH Aachen  Pauwelsstraße 30  Aachen  52074  GERMANY |
|  | Ethik-Kommission an der  Medizinischen Fakultät der RWTH Aachen  Pauwelsstraße 30  Aachen  52074  GERMANY |
|  | Ethik-Kommission an der  Medizinischen Fakultät der RWTH Aachen  Pauwelsstraße 30  Aachen  52074  GERMANY |
|  | Ethik-Kommission an der  Medizinischen Fakultät der RWTH Aachen  Pauwelsstraße 30  Aachen  52074  GERMANY |
|  | Ethik-Kommission an der  Medizinischen Fakultät der RWTH Aachen  Pauwelsstraße 30  Aachen  52074  GERMANY |
|  | Ethik-Kommission an der  Medizinischen Fakultät der RWTH Aachen  Pauwelsstraße 30  Aachen  52074  GERMANY |
|  | Ethik-Kommission an der  Medizinischen Fakultät der RWTH Aachen  Pauwelsstraße 30  Aachen  52074  GERMANY |
|  | Ethik-Kommission an der  Medizinischen Fakultät der RWTH Aachen  Pauwelsstraße 30  Aachen  52074  GERMANY |
|  | Ethik-Kommission an der  Medizinischen Fakultät der RWTH Aachen  Pauwelsstraße 30  Aachen  52074  GERMANY |
|  | Ethik-Kommission an der  Medizinischen Fakultät der RWTH Aachen  Pauwelsstraße 30  Aachen  52074  GERMANY |
|  | Ethik-Kommission an der  Medizinischen Fakultät der RWTH Aachen  Pauwelsstraße 30  Aachen  52074  GERMANY |
|  | Ethik-Kommission an der  Medizinischen Fakultät der RWTH Aachen  Pauwelsstraße 30  Aachen  52074  GERMANY |
|  | Ethik-Kommission an der  Medizinischen Fakultät der RWTH Aachen  Pauwelsstraße 30  Aachen  52074  GERMANY |
|  | Ethik-Kommission an der  Medizinischen Fakultät der RWTH Aachen  Pauwelsstraße 30  Aachen  52074  GERMANY |
|  | Ethik-Kommission an der  Medizinischen Fakultät der RWTH Aachen  Pauwelsstraße 30  Aachen  52074  GERMANY |
|  | Ethik-Kommission an der  Medizinischen Fakultät der RWTH Aachen  Pauwelsstraße 30  Aachen  52074  GERMANY |
|  | Ethik-Kommission an der  Medizinischen Fakultät der RWTH Aachen  Pauwelsstraße 30  Aachen  52074  GERMANY |
|  | National Ethics Committee  284, Messoghion Avenue  Cholargos  Athens  15562  GREECE |
|  | National Ethics Committee  284, Messoghion Avenue  Cholargos  Athens  15562  GREECE |
|  | National Ethics Committee  284, Messoghion Avenue  Cholargos  Athens  15562  GREECE |
|  | National Ethics Committee  284, Messoghion Avenue  Cholargos  Athens  15562  GREECE |
|  | National Ethics Committee  284, Messoghion Avenue  Cholargos  Athens  15562  GREECE |
|  | National Ethics Committee  284, Messoghion Avenue  Cholargos  Athens  15562  GREECE |
|  | National Ethics Committee  284, Messoghion Avenue  Cholargos  Athens  15562  GREECE |
|  | National Ethics Committee  284, Messoghion Avenue  Cholargos  Athens  15562  GREECE |
|  | National Ethics Committee  284, Messoghion Avenue  Cholargos  Athens  15562  GREECE |
|  | National Ethics Committee  284, Messoghion Avenue  Cholargos  Athens  15562  GREECE |
|  | National Ethics Committee  284, Messoghion Avenue  Cholargos  Athens  15562  GREECE |
|  | National Ethics Committee  284, Messoghion Avenue  Cholargos  Athens  15562  GREECE |
| Comité Independiente de Etica Latin Ethics  14 Calle 7-13 Zona 9, Edificio Torre Blanca , Nivel 10  01009  GUATEMALA |  |
| Comité Independiente de Etica Latin Ethics  14 Calle 7-13 Zona 9, Edificio Torre Blanca , Nivel 10  01009  GUATEMALA |  |
| Comité Independiente de Etica Latin Ethics  14 Calle 7-13 Zona 9, Edificio Torre Blanca , Nivel 10  01009  GUATEMALA |  |
| Comité Independiente de Etica Latin Ethics  14 Calle 7-13 Zona 9, Edificio Torre Blanca , Nivel 10  01009  GUATEMALA |  |
| Joint CUHK-NTEC Clinical Research Ethics Committee  8th Floor  Lui Che Woo Clinical Sciences Building  Prince of Wales Hospital  Shatin HONG KONG |  |
| IRB of the HKU /Hospital Authority Hong Kong West Cluster  Rm 901, Administration Block  102 Pokfulam Road  HONG KONG |  |
|  | Medical Research Council, Ethics Committee for Clin. Pharm.  Klin. Farm. Etikai Bizottság, Arany János u. 6-8  Budapest  1051  HUNGARY |
|  | Medical Research Council, Ethics Committee for Clin. Pharm.  Klin. Farm. Etikai Bizottság, Arany János u. 6-8  Budapest 1051  HUNGARY |
|  | Medical Research Council, Ethics Committee for Clin. Pharm.  Klin. Farm. Etikai Bizottság, Arany János u. 6-8  Budapest 1051  HUNGARY |
|  | Medical Research Council, Ethics Committee for Clin. Pharm.  Klin. Farm. Etikai Bizottság, Arany János u. 6-8  Budapest 1051  HUNGARY |
|  | Medical Research Council, Ethics Committee for Clin. Pharm.  Klin. Farm. Etikai Bizottság, Arany János u. 6-8  Budapest  1051  HUNGARY |
|  | Medical Research Council, Ethics Committee for Clin. Pharm.  Klin. Farm. Etikai Bizottság, Arany János u. 6-8  Budapest  1051  HUNGARY |
|  | Medical Research Council, Ethics Committee for Clin. Pharm.  Klin. Farm. Etikai Bizottság, Arany János u. 6-8  Budapest  1051  HUNGARY |
|  | Medical Research Council, Ethics Committee for Clin. Pharm.  Klin. Farm. Etikai Bizottság, Arany János u. 6-8  Budapest  1051  HUNGARY |
|  | Medical Research Council, Ethics Committee for Clin. Pharm.  Klin. Farm. Etikai Bizottság, Arany János u. 6-8  Budapest  1051  HUNGARY |
|  | Medical Research Council, Ethics Committee for Clin. Pharm.  Klin. Farm. Etikai Bizottság, Arany János u. 6-8  Budapest  1051  HUNGARY |
|  | Medical Research Council, Ethics Committee for Clin. Pharm.  Klin. Farm. Etikai Bizottság, Arany János u. 6-8  Budapest  1051  HUNGARY |
|  | Medical Research Council, Ethics Committee for Clin. Pharm.  Klin. Farm. Etikai Bizottság, Arany János u. 6-8  Budapest  1051  HUNGARY |
|  | Medical Research Council, Ethics Committee for Clin. Pharm.  Klin. Farm. Etikai Bizottság, Arany János u. 6-8  Budapest  1051  HUNGARY |
| Dr. Ramesh Cardiac and Multispeciality Hospital P Ltd.,  Ring Road, Near ITI College, Vijaywada-520 008  Vijaywada  520 008  INDIA |  |
| Ethics Committee Mysore Medical College  and Research Institute and Associated Hospitals  Irwin Road ,Mysore-570021  Mysore  570021  INDIA |  |
| KIMS Institutional Ethics Committee  1-8-31/1, Minister Road, Secunderabad  500003  INDIA |  |
| Departement of cardiology  Apollo hospital Educational Education  21 Greams lane  Off Greams road  Chennai, Tamil Nadu 600006  INDIA |  |
| The Madras Medical Mission,  Institutional Ethics Committee,  #4-A, Dr. J. J. Nagar, Mogappair, Chennai-600 037,Chennai  600037  INDIA |  |
| S.R.Kalla Memorial Ethical Committee for Human Research  78,Dhulleshwar Garden ,Behind HSBC Bank  Sardar Patel Marg, C-Scheme  Jaipur  302001  INDIA |  |
| Fortis Hospital Ethics Committee  154/9,Bannerghatta Road  opp Indian Institute of Management Bangalore 560076  Bangalore  560076  INDIA |  |
| Ethics Committee S.P. Medical College and A.G. Hospitals  S.P. Medical College and A.G. Hospitals, Bikaner, 334 003  Bikaner  334 003  INDIA |  |
| IEC Lisie Hospital  P.B # 3053, Kochi - 682018  Kochi  682018  INDIA |  |
| Ethics Committee of Manipal Hospital  , 98, HAL Airport Road  State - Karnataka  Bangalore  560017  INDIA |  |
| M.S Ramaiah medical college and hospitalethical review board  MSRIT post,  New BEL road  Bangalore  560054  INDIA |  |
| St. John's Medical College and Hospital, IRB Board  Sarjapur Road  Koramangala, II Block  Bangalore  560034  Karnataka  INDIA |  |
| Medanta Independent Ethics Committee,  Medanta - The Medicity,  Sector- 38, Gurgaon, Haryana.  Gurgoan122001  INDIA |  |
| Institutional Ethics Committee - Sunshine Hospitals  Penderghast Road, Opposite Parsi Dharamsala, Behind Paradise Hotel,  Secunderabad,  Telangana  Secunderabad  500003  INDIA |  |
| EC  Indus Hospital  KGM Down Road, Maharanipeta,  Visakhapatanam- 530002  Visakhapatanam  530002  INDIA |  |
| Bhagwan Mahavir Medical Research Centre,  10-1-1 Bhagwan Mahavir Marg, A C Guard,  Hyderabad-500004  Hyderabad  500004  INDIA |  |
| Max Super Speciality Hospital  (East Block)  A unit of Devki Devi Foundation  2 Press Enclave Road Saket  Delhi  110117  INDIA |  |
| QUEEN'S NRI HOSPITAL.  Gurudwara Lane  Visakhapatnam  530013  INDIA |  |
| Ethics Committee  Vintage Hospital & Medical Research Centre Pvt Ltd,  Caculo Enclave, St Inez, Panaji, Goa - 403001  Goa  403001  INDIA |  |
| CARE Hospital,Care Convergence Centre  H.No: 8-2-595/2/B,Road No 10  Banjara Hills  Hyderabad  500034  INDIA |  |
| KEM Hospital Research Centre  Sardar Moodliar Road, Rasta Peth  Pune  411011  INDIA |  |
| Institutional Ethics committee - Noble Hospital  Pvt. Ltd.153,  Magarpatta City Road Pune 411013  INDIA |  |
| Dinanath Mangeshkar Hospital Ethics committee  Erandawane  Pune  411004  INDIA |  |
| Institutional Ethics Committee - Joshi Hospital  Maharashtra Medical Research Society,  778, Shivaji Nagar,  Pune  411004  INDIA |  |
| Ethics committee - Sanjeevan Hospital  Plot no 23, off Karve road, Erandawane,  Erandawane, Pune- 411004, Maharashtra, India  Pune  411004  INDIA |  |
| Krishna Institute of Medical Sciences Deemed University  Karad, Dist. Satara  Maharashtra  Satara  415110  INDIA |  |
| Superspeciality Hospital  Max Super Speciality Hospital ( A unit Of Devki Devi Foundation)  2, Press Enclave Road, Saket, NewDelhi, 110017  Delhi  110017  INDIA |  |
| Kamalnayan Bajaj Hospital  Gut No.43, Satara Parisar, Bajaj Marg  Beed Bypass Road, Aurangabad-431005  Maharashtra  INDIA |  |
| Ethics committee for research on Human subject  Seth GS Medical College and KEM Hospital  Room#46, Old Hospital Bldg,Next to Medicine Seminar Hall  2nd Floor, Parel  Mumbai  400012  INDIA |  |
| AMC MET Ethics Committee  5th floor, Department of Pharmacology,  Sheth L.G. Hospital and AMC MET Medical College,  Maninagar  Ahmedabad  380008 INDIA |  |
| IPGMER SSKM Hospital,  244 Acharya Jagadish Chandra Bose Road,  Kolkata 700020  INDIA |  |
| Kolkata Medical College - IEC for Human Research  88 college Street  Kolkata 700073  INDIA |  |
| EC Lokmanya Tilak Municipal Medical College & General Hospital  2nd floor, college building,  Dr.B.R.Ambedkar Road, Sion, Mumbai -400022  INDIA |  |
| Ethics Committee of B J Govt. Medical College and Sassoon General Hospital  Sassoon Road, Somwarpeth,  Pune-411001  INDIA |  |
| Ethics committee of B.M Birla Heart Research Centre  1/1, National Library Road  Kolkata  700027  INDIA |  |
| SRM Medical College Hospital & Research Centre  SRM Nagar, Potheri, Kattankulathur-603203,  Kancheepuram District, Tamilnadu  Kancheepuram  603203  INDIA |  |
| Mediciti Ethics Committee  Mediciti Hopsitals, Mediciti Institute of Medical Sciences  5-9-22, Near Sarovar Hotel  Secretariat Road, Hyderabad  500063  INDIA |  |
| Narayana Health Medical Ethics Committee  #258/A,Bommasandra Industrial Area,Anekal(Taluk)  Bangalore  Karnataka  Bangalore  560099  INDIA |  |
| Narayana Health Medical Ethics Committee  #258/A,Bommasandra Industrial Area,Anekal(Taluk)  Bangalore  Karnataka  Bangalore  560099  INDIA |  |
| Meditrina Institute Ethics Committee  Meditrina Institute of Medical Sciences, 278,  Central Bazar Road, Ramdaspeth, Nagpur-440010  INDIA |  |
| Satguru Pratap Singh Apollo Hospitals  Sherpur Chowk, G.T.Road  Ludhiana  141003  INDIA |  |
| Nirmal Hospital Private Limited Ethics Committee  Ring road, Surat-395002, Gujarat.  Surat  395002  INDIA |  |
| Ethics Committee  Sir Gangaram Hospital  Dept. of Cardiology, 5th Floor  Rajinder Nagar  110060  New Delhi  INDIA |  |
| Niramaya Hospital Ethics Committee  S.No 4742  Behind Jai Hind Petrol Pump  Next to chinchwad post office  Pune 411019  INDIA |  |
| Omega Ethical Committee  Mahaveer Circle, Kankanady  Mangalore  575002  INDIA |  |
| Magna-Care Ethics Committee  Chopda Medicare & Research Centre Pvt. Ltd,  Magnum Heart Institute, Plot no. 3/5, Patil Lane no.1, Laxmi Nagar,  Near K.B.H Vidyalaya, Canada Corner,  Nashik  422005  INDIA |  |
| EC - Crescent Hospital & Heart Centre, Near Lokmat Square,  Dhantoli, Nagpur - 440012  INDIA |  |
| Virtuous Institutional Medical Research Ethics Committee  IVth Floor  Tikekar Road  Congress Nagar Square  Dhantoli  Nagpur  440012  INDIA |  |
| Jehagir Hospital premises  32,Sassoon Road  Pune  411001  INDIA |  |
| Fortis Memorial Research Institutev-EC  Meeting Room, Lower Ground Floor  Sector 44  Gurgaon  122002  INDIA |  |
| Artemis Health Sciences Institutional Ethics Committee/ IRB  Sec 51  Gurgaon  122001  INDIA |  |
| Human Research Ethics Committee  H.M. Patel Center for Medical Care & Education  Shri Krishna Hospital & Medical Reasearch Center,  Managed by Charutar Arogya Mandal  Gokal Nagar  Karamsad  388325  INDIA |  |
| Ethics Committee of KLE University,  JNMC Campus, Nehru Nagar,  Karnataka - Belgaum  590010  INDIA |  |
| Datta Meghe Institute of Medical Sciences  Sawangi (Meghe), Wardha-442004  Wardha  442004  INDIA |  |
| Andhra Hospital - Institutional Ethics Committee  C.V.R Complex, Prakasam Road,  Vijayawada - 520 002, Andhra Pradesh, India  Vijayawada  520002  INDIA |  |
| Ethics Committee of Rabin Medical Center  39 Jabotinsky Street  Petach Tikva  49100  ISRAEL |  |
| Ethics Committee of Meir Medical Center  59 Tchernickovski Street  Kfar Saba  44281  ISRAEL |  |
| Ethics Committee of Ziv Medical Center  PO Box 1008  Safed  13100  ISRAEL |  |
| Ethics Committee of Western Galilee Medical Center  PO Box 21  Nahariya  22100  ISRAEL |  |
| Ethics Committee of Hadassah Medical Center  POB 12000  Jerusalem  91120  ISRAEL |  |
| Ethics Committee of HaEmek Medical Center  Afula  ISRAEL |  |
| Ethics Committee of Wolfson Medical Center  62 HaLochamim Street  Holon  58100  ISRAEL |  |
| Ethics Committee of Ziv Medical Center  PO Box 1008  Safed  13100  ISRAEL |  |
| Ethics Committee of Barzilai Medical Center  3 HaHistadrut Street  Ashkelon  78278  ISRAEL |  |
| Ethics Committee of Tel Aviv Sourasky Medical Center  6 Weizman Street  Tel Aviv  64239  ISRAEL |  |
| Ethics Committee of Bnei Zion Medical Center  Golumb Street 47  POB 4940  Haifa  31048  ISRAEL |  |
| Ethics Committee of Soroka Medical Center  PO Box 151  Beer Sheva  85101  ISRAEL |  |
| Ethics Committee of Meir Medical Center  59 Tchernickovski Street  Kfar Saba  44281  ISRAEL |  |
| Ethics Committee of Tel Aviv Sourasky Medical Center  6 Weizman Street  Tel Aviv  64239  ISRAEL |  |
| Ethics Committee of Holy Family Hospital  PO Box 8  Nazareth  16100  ISRAEL |  |
| Ethics Committee of Shaare Zedek Medical Center  PO Box 3235  Jerusalem  91031  ISRAEL |  |
| Ethics Committee of Bnei Zion Medical Center  Golumb Street 47  POB 4940  Haifa  31048  ISRAEL |  |
| Ethics Committee of Rambam Medical Center  PO Box 9602  HaAliya Street 8, Bat Galim  Haifa  31096  ISRAEL |  |
| Ethics Committee of Rabin Medical Center  39 Jabotinsky Street  Petach Tikva  49100  ISRAEL |  |
| Ethics Committee of Kaplan Medical Center  PO Box 1  Rehovot  76100  ISRAEL |  |
| Ethics Committee of Wolfson Medical Center  62 HaLochamim Street  Holon  58100  ISRAEL |  |
| EC of Sheba Medical Center  The Chaim Sheba Medical Center  Tel-Hashomer  52621  ISRAEL |  |
| EC of Baruch Padeh Medical Center, Poriya  EC of Baruch Padeh MC, Poriya  Tiberias  ISRAEL |  |
| Ethics Committee of HaEmek Medical Center  Afula  ISRAEL |  |
| Ethics Committee of Bnei Zion Medical Center  Golumb Street 47  POB 4940  Haifa  31048  ISRAEL |  |
| Ethics Comittee of Assaf HaRofe Medical Center  D.N. Beer Yaakov  Tzrifin  70300  ISRAEL |  |
| Ethics Committee of Hadassah Medical Center  POB 12000  Jerusalem  91120  ISRAEL |  |
| Comitato Etico Seconda universita'degli studi di napoli, aou sun - aorn ospedali dei colli  Via s. Maria di costantinopoli, 104  Napoli 80138  ITALY |  |
| Comitato Etico DELL'UNIVERSITA' FEDERICO II  Via Pansini, 5  Napoli 80131  ITALY |  |
| IRCCS Fondazione Don Carlo Gnocchi di Milano  Comitato per la Sperimentazione Clinica del Medicinali  Via Alfonso Capecelatro, 66  Milano 20148  ITALY |  |
| Cardiologico Monzino  Cardiologico Monzino  VIA RIPAMONTI 435  C/O ISTITUTO EUROPEO DI ONCOLOGIA  Milano 20141  ITALY |  |
| AOU Policlinico Paolo Giaccone  Comitato per la Sperimentazione clinica dei Medicinali  Via del Vespro, 127  Palermo 90127  ITALY |  |
| Provincia di Brescia  Comitato per la sperimentazione Clinica dei Medicinali  Piazzale Spedali Civili, 1  Brescia 25124  ITALY |  |
| Comitato Etico della Provincia di Bergamo  PIAZZA OMS,1  Bergamo 24127  ITALY |  |
| Comitato Unico della Provincia di Ferrara  VIA ALDO MORO, 8  Ferrara 44124  ITALY |  |
| CEAS Comitato Etico Aziende Sanitarie dell'Umbria  Via della Rivoluzione, 16  Ellera Di Corciano  06070  Perugia  ITALY |  |
| IRST IRCCS e Area Vasta Romagna  Comitato per la sperimentazione clinica del medicinali  Via P. Maroncelli, 40  Meldola  47014  Forlì-Cesena  ITALY |  |
| Area Vasta Nord Ovest _AOUP  Comitato per la sperimentazione clinica dei Medicinali  Via Roma, 67  Pisa 56126  ITALY |  |
| Comitato Etico della Provincia di Bergamo  PIAZZA OMS,1  Bergamo  24127  ITALY |  |
| Ufficio Protocollo Generale  Comitato per la Sperimentazione Clinica del Medicinali  Piazza S.M. di Gesù, 5  Catania 95124  ITALY |  |
| CE Spermentazi CLINICA (CESC) - PROV.TREVISO E BELLUNO  VIA SANT'AMBROGIO DI FIERA,37  Treviso  ITALY |  |
| Comitato Etico Interaziendale di Novara  Corso Mazzini, 18  Ospedale Maggiore della Carità  Novara 28100  ITALY |  |
| Comitato Etico Lazio 1  Farmacia Circonvallazione Gianicolense, 87  Roma 00152  ITALY |  |
| Provincia Monza Brianza  Comitato per la sperimentazione clinica dei Medicinali  Via Pergolesi, 33  20052  Monza e Brianza  ITALY |  |
| CE Spedali Civili di Brescia  Piazza Spedali Civili, 1  Brescia 25100  ITALY |  |
| IRST IRCCS e Area Vasta Romagna  Comitato per la sperimentazione clinica del medicinali  Via P. Maroncelli, 40  Meldola  47014  Forlì-Cesena  ITALY |  |
| Comitato Etico della Provincia di Bergamo  PIAZZA OMS,1  Bergamo  24127  ITALY |  |
| IRCCS - OSP.San Raffaele  Comitato Etico  Via Olgettina, 60  Milano  20132  ITALY |  |
| Comitato etico milano area C  pza Ospedale maggiore 3  Milano  20162  ITALY |  |
| Ce sperimentazione clinica (cesc) - prov.treviso e belluno  Via sant'ambrogio di fiera,37  Treviso  ITALY |  |
| Area Vasta Nord Ovest _AOUP  Comitato per la sperimentazione clinica dei Medicinali  Via Roma, 67  Pisa 56126  ITALY |  |
| Institutional Review Board of Kyoto University Hospital  54 Shogoin Kawahara-cho, Sakyo-ku  Kyoto-Shi  606-8507  Kyoto  JAPAN |  |
| IRB of Teikyo University Hospital  2-11-1 Kaga  Itabashi-Ku  173-8606  Tokyo JAPAN |  |
| Hiroshima City Hiroshima Citizens Hospital IRB  7-33 Motomachi, Naka-Ku  Hiroshima-Shi  730-8518  JAPAN |  |
| Kitasato University Sagamihara Institutional Review Board  1-15-1 Kitasato, Minami-ku Sagamihara-Shi  252-0375  Kanagawa  JAPAN |  |
| Nagoya Daiichi Hospital Institutional Review Board  3-35 Michishita-Cho, Nakamura-Ku  Nagoya-Shi  453-8511  Aichi  JAPAN |  |
| Yokohama Sakae Kyosai Hospital Institutional Review Board  132 Katsura-Cho, Sakae-Ku  Yokohama-Shi  247-0005  Kanagawa  JAPAN |  |
| Urasoe General Hospital Institutional Review Board  4-16-1 Iso  Urasoe-Shi  901-2132  Okinawa  JAPAN |  |
| CPC Clinical Trial Hospital Institutional Review Board  4-18-38 Toso  Kagoshima-Shi  890-0081  Kagoshima JAPAN |  |
| Sone Clinic Institutional Review Board  3-31-1 Shinjuku  Shinjuku-Ku  160-0022  Tokyo  JAPAN |  |
| Institutional Review Board of Sakurabashi Watanabe Hospital  2-4-32 Umeda, Kita-ku  Osaka-Shi  530-0001  Osaka  JAPAN |  |
| Shin-Koga Hospital Institutional Review Board  120 Tenjin-cho  Kurume-Shi  830-8577  Fukuoka  JAPAN |  |
| Sone Clinic Institutional Review Board  3-31-1 Shinjuku  Shinjuku-Ku  160-0022  Tokyo  JAPAN |  |
| Nihon University Hospitals' Joint Institutional Review Board  30-1, Oyaguchi,. Kami-cho  Itabashi-Ku  173-8610  Tokyo  JAPAN |  |
| Kokura Memorial Hospital Institutional Review Board  3-2-1 Asano, Kokurakita-ku Kitakyushu-Shi  802-8555  Fukuoka  JAPAN |  |
| Iwate Medical University Institutional Review Board  19-1 Uchimaru  Morioka-Shi  020-8505  Iwate  JAPAN |  |
| Kobe City Medical Center General Hospital IRB  2-1-1 Minatojimaminamimachi Chuo-ku  Kobe-Shi  650-0047  Hyogo JAPAN |  |
| CPC Clinical Trial Hospital Institutional Review Board  4-18-38 Toso  Kagoshima-Shi  890-0081  Kagoshima  JAPAN |  |
| Clinical Research Hospital Tokyo Institutional Review Board  3-87-4 Hara-machi  Shinjuku-Ku  162-0053  Tokyo  JAPAN |  |
| National Hospital Organization Central Review Board  2-5-21 Higashigaoka  Meguro-Ku  152-8621  Tokyo  JAPAN |  |
| Yokohama City University Medical Center IRB  4-57 Urafune-cho, Minami-ku  Yokohama-Shi  232-0024  Kanagawa JAPAN |  |
| Sone Clinic Institutional Review Board  3-31-1 Shinjuku  Shinjuku-Ku  160-0022  Tokyo  JAPAN |  |
| National Hospital Organization Central Review Board  2-5-21 Higashigaoka  Meguro-Ku  152-8621  Tokyo  JAPAN |  |
| National Hospital Organization Central Review Board  2-5-21 Higashigaoka  Meguro-Ku  152-8621  Tokyo  JAPAN |  |
| National Hospital Organization Central Review Board  2-5-21 Higashigaoka  Meguro-Ku  152-8621  Tokyo  JAPAN |  |
| National Hospital Organization Central Review Board  2-5-21 Higashigaoka  Meguro-Ku  152-8621  Tokyo  JAPAN |  |
| Tokyo Saiseikai Central Hospital Institutional Review Board  1-4-17 Mita  Minato-Ku  108-0073  JAPAN |  |
| National Hospital Organization Central Review Board  2-5-21 Higashigaoka  Meguro-Ku  152-8621  Tokyo  JAPAN |  |
| Koyasu Neurosurgical Clinic Institutional Review Board  1-6-1 Futamatagawa, Asahi-ku  Yokohama-Shi  241-0821  Kanagawa  JAPAN |  |
| Tokushima Pref. Central Hospital Institutional Review Board  1-10-3 Kuramoto-cho  Tokushima-Shi  770-8539  Tokushima JAPAN |  |
| Saiseikai Kumamoto Hospital Institutional Review Board  5-3-1 Chikami, Minami-ku  Kumamoto-Shi  861-4193  Kumamoto  JAPAN |  |
| Kusatsu General Hospital Institutional Review Board  1660 Yabase-cho  Kusatsu-Shi  525-8585  Shiga  JAPAN |  |
| Rinku General Medical Center Institutional Review Board  2-23 Rinkuouraikita  Izumisano-Shi  598-8577  Osaka  JAPAN |  |
| Instutution Review Board of Showa University Hospital  1-5-8 Hatanodai  Shinagawa-Ku  142-8666  Tokyo  JAPAN |  |
| Tokushima Red Cross Hospital Institutional Review Board  Komatsushima-cho  Irinokuchi103  Komatsushima-Shi  773-8502  Tokushima JAPAN |  |
| Clinical Research Hospital Tokyo Institutional Review Board  3-87-4 Hara-machi  Shinjuku-Ku  162-0053  Tokyo JAPAN |  |
| Instutution Review Board of Kobe university Hospital  7-5-2 Kusunoki-cho, Chuo-ku  Kobe-Shi  650-0017  Hyogo  JAPAN |  |
| National Hospital Organization Central Review Board  2-5-21 Higashigaoka  Meguro-Ku  152-8621  Tokyo  JAPAN |  |
| National Hospital Organization Central Review Board  2-5-21 Higashigaoka  Meguro-Ku  152-8621  Tokyo JAPAN |  |
| Kansai Rosai Hospital IRB  3-1-69 Inabaso  Amagasaki-Shi  660-8511  Hyogo  JAPAN |  |
| IRB of Chonnam National University Hospital  410 Jebong-Ro, Dong-gu,  Gwangju  501-757  Gwangju  KOREA, REPUBLIC OF |  |
| Institutional Review Board of Kyungpook Nat'l Univ. Hospital  50, 2-ga, Samdeok-dong  Jung-gu  Daegu  700-721  KOREA, REPUBLIC OF |  |
| Pusan National University Hospital  10 1-ga Ami-dong Seo-gu  Busan  KOREA, REPUBLIC OF |  |
| IRB of Seoul St. Mary's hospital  222, Banpo-Daero, Seocho-gu  137-701  Seoul  KOREA, REPUBLIC OF |  |
| IRB of Wonju Christian Hospital  162, Ilsan-dong, Wonju, Gangwon-do, Republic of Korea  Wonju  220-701  Gangwon-do  KOREA, REPUBLIC OF |  |
| IRB of Korea University Guro Hospital  148, Gurodong-Ro, Guro-Gu  Seoul  152-703  KOREA, REPUBLIC OF |  |
| IRB of SMG-SNH Boramae Medical Center  20 Boramae-ro 5-gil, Dongjak-Gu  Seoul  156-707  KOREA, REPUBLIC OF |  |
| IRB of Seoul National University Bundang Hospital  173-82 Gumi-ro, Bundang-gu, Seoungnam, Gyeonggi-do, 463-707, Republic of Korea  Seoungnam  Gyeonggi-do  KOREA, REPUBLIC OF |  |
| Ajou University Hospital  San 5, Woncheon-dong, Yeongtong-gu,Suwon  443-721  Gyeonggi-do  KOREA, REPUBLIC OF |  |
| IRB of Seoul National University Hospital  101, Daehak-ro, Jongno-gu  03080  Seoul  KOREA, REPUBLIC OF |  |
| IRB of KyungHee University Hospital at Gangdong  149, Sangil-Dong, Gangdong-Gu  134-727  Seoul  KOREA, REPUBLIC OF |  |
| IRB of Dongguk University Ilsan Hospital  814 Siksa-Dong  Ilsandong-Gu  Goyang  Gyeonggi-Do  410-773  Gyeonggi-do  KOREA, REPUBLIC OF |  |
| Institutional Review Board of Dong-A Univ. Medical Center  1, 3-ga, Dongdaeshin-dong  Seo-gu  Busan  602-715  KOREA, REPUBLIC OF |  |
| Ethics Committee for Clinical Trials  Aizkraukles Street 21-113  Riga  LV-1006  LATVIA | Ethics Committee for Clinical Trials  Aizkraukles Street 21-113  Riga  LV-1006  LATVIA |
| Ethics Committee for Clinical Trials  Aizkraukles Street 21-113  Riga  LV-1006  LATVIA | Ethics Committee for Clinical Trials  Aizkraukles Street 21-113  Riga  LV-1006  LATVIA |
| Ethics Committee for Clinical Trials  Aizkraukles Street 21-113  Riga  LV-1006  LATVIA | Ethics Committee for Clinical Trials  Aizkraukles Street 21-113  Riga  LV-1006  LATVIA |
| Ethics Committee for Clinical Trials  Aizkraukles Street 21-113  Riga  LV-1006  LATVIA | Ethics Committee for Clinical Trials  Aizkraukles Street 21-113  Riga  LV-1006  LATVIA |
| Ethics Committee for Clinical Trials  Aizkraukles Street 21-113  Riga  LV-1006  LATVIA | Ethics Committee for Clinical Trials  Aizkraukles Street 21-113  Riga  LV-1006  LATVIA |
| Ethics Committee for Clinical Trials  Aizkraukles Street 21-113  Riga  LV-1006  LATVIA | Ethics Committee for Clinical Trials  Aizkraukles Street 21-113  Riga  LV-1006  LATVIA |
| Lithuanian Bioethics Committee  Vilniaus str. 16  Vilnius  LT-01402  LITHUANIA | Lithuanian Bioethics Committee  Vilniaus str. 16  Vilnius  LT-01402  LITHUANIA |
| Lithuanian Bioethics Committee  Vilniaus str. 16  Vilnius  LT-01402  LITHUANIA | Lithuanian Bioethics Committee  Vilniaus str. 16  Vilnius  LT-01402  LITHUANIA |
| Lithuanian Bioethics Committee  Vilniaus str. 16  Vilnius  LT-01402  LITHUANIA | Lithuanian Bioethics Committee  Vilniaus str. 16  Vilnius  LT-01402  LITHUANIA |
| Lithuanian Bioethics Committee  Vilniaus str. 16  Vilnius  LT-01402  LITHUANIA | Lithuanian Bioethics Committee  Vilniaus str. 16  Vilnius  LT-01402  LITHUANIA |
| Lithuanian Bioethics Committee  Vilniaus str. 16  Vilnius  LT-01402  LITHUANIA | Lithuanian Bioethics Committee  Vilniaus str. 16  Vilnius  LT-01402  LITHUANIA |
| Lithuanian Bioethics Committee  Vilniaus str. 16  Vilnius  LT-01402  LITHUANIA | Lithuanian Bioethics Committee  Vilniaus str. 16  Vilnius  LT-01402  LITHUANIA |
| Lithuanian Bioethics Committee  Vilniaus str. 16  Vilnius  LT-01402  LITHUANIA | Lithuanian Bioethics Committee  Vilniaus str. 16  Vilnius  LT-01402  LITHUANIA |
| Lithuanian Bioethics Committee  Vilniaus str. 16  Vilnius  LT-01402  LITHUANIA | Lithuanian Bioethics Committee  Vilniaus str. 16  Vilnius  LT-01402  LITHUANIA |
| Lithuanian Bioethics Committee  Vilniaus str. 16  Vilnius  LT-01402  LITHUANIA | Lithuanian Bioethics Committee  Vilniaus str. 16  Vilnius  LT-01402  LITHUANIA |
| "Universiti Teknologi MARA (UiTM), Sungai Buloh Campus  Jalan Hospital 4700  Sungai Buloh Selangor  4700  MALAYSIA |  |
| Ethics Committee  Institut Jantung Negara  145 Jalan Tun Razak  Kuala Lumpur  50400  MALAYSIA |  |
| Medical Research & Ethics Committee  C/o Institute for Health Management  Jalan Rumah Sakit, Bangsar  Kuala Lumpur  59000  MALAYSIA |  |
| UiTM Research Ethics Committee  University Teknology MARA, Sungai Buloh Campus, Jalan Hospital  47000 Sungai Buloh  Selangor  MALAYSIA |  |
| Research Ethics Committee (UKM)  Universiti Kebangsaan Malaysia  Faculty of Medicine  Jalan Yaakob Latif  Bandar Tun Razak Cheras  Kuala Lumpur  56000  MALAYSIA |  |
| Medical Research & Ethics Committee  C/o Institute for Health Management  Jalan Rumah Sakit, Bangsar  Kuala Lumpur  59000  MALAYSIA |  |
| Research Ethics Committee (Human) / USM  Universiti Sains Malaysia  Kubang Kerian, Kota Bahru  Kelantan  16150  MALAYSIA |  |
| Clinical Research Institute  Blvd. Manuel Avila Camacho 1994 - 1103  San Lucas Tepetlacalco  Tlalnepantla  54055  Estado de México  MEXICO |  |
| CEI Hospital General de Durango  Av. 5 de febrero, esquina Norman F. S/N  Durango  34000  MEXICO |  |
| CEI Sociedad Médica del Hospital Ángeles Culiacán, S.C.  Blvd. Alfonso G. Calderón 2193, Col. Desarrollo Urbano Tres Ríos Culiacán  80020 Sinaloa  MEXICO |  |
| CEI Mexico Centre for Clinical Research, S.A de C.V.  Amores 709 Col. Del Valle  D.F.  03100  México, D.F.  MEXICO |  |
| CEI Instituto Jalisciense de Investigación Clínica S.AdeC.V.  Penitenciaria No. 20 Col. Centro  Guadalajara  44100  Jalisco  MEXICO |  |
| CEI Escuela de Medicina del ITESM  Av. Morones Prieto 3000 Pte.  Col. Los Doctores  Monterrey  64710  MEXICO |  |
| CEI Centro. de Esp. Méd. del Edo. de Ver., Dr. Rafael Lucio  Avenida Ruiz Cortines 2903, Col. Unidad Magisterial  Xalapa 91020  Veracruz MEXICO |  |
| CEI Hospital Central Dr. Ignacio Morones Prieto  Av.Venustiano Carranza 2395  Col. Zona Universitaria  San Luis Potosi  72240  San Luis Potosi  MEXICO |  |
| CEI Hospital Civil de Guadalajara Fray Antonio Alcalde  Hospital 278, Col. El retiro  Guadalajara  44280 Jalisco  MEXICO |  |
| CEI de la Facultad de Medicina y HU de la UANL  Av. Madero y Gonzalitos S/N, Col Mitras Centro  Monterrey  64460 Nuevo Leon  MEXICO |  |
| CEI Oaxaca Site Management Organization S.C.  Humboldt 302, Col. Centro  Oaxaca  68000  MEXICO |  |
| CEI Winsett Rethman, S.A. de C.V. (CEIIC)  Capitán Aguilar Sur No. 669, Col. Obispado  Monterrey  64060 Nuevo Leon  MEXICO |  |
| CEI Instituto de Corazón de Querétaro, S. A. de C.V.  Prol. Priv. Ignacio Zaragoza, 16-B 2° piso. Col. Centro  76000 Queretaro  MEXICO |  |
| CEI Promt. M  éd. Ags. SA de CV (Hospital Cardiológica de Ags)  República del Ecuador 200, Fracc. Las Américas  Aguascalientes  20230  MEXICO |  |
| CEI Hospital General Dr. Miguel Silva, SSA Michoacán  Samuel Ramos S/N Col. Centro,  Morelia 58000  Michoacan  MEXICO |  |
| CEI Cardiolink Clintrials  Hidalgo 1813, Col. Obispado  Monterrey  64060  Nuevo Leon  MEXICO |  |
| Instituto Nacional De Cardiología  Juan Badiano No. 1 Intersección Perif. S  Col. Sección Xv.  D.F.  14080  México, D.F.  MEXICO |  |
| CEI Mexico Centre for Clinical Research, S.A de C.V.  Amores 709 Col. Del Valle  D.F.03100  México, D.F.  MEXICO |  |
| CEI Op. de Hosp. Ángeles, S.A. de C.V. Hosp. Ángeles Mocel  Gelati No. 29, Col. San Miguel Chapultepec  D.F.11850  México, D.F.  MEXICO |  |
| CEI IMSS, Coordinación de Investigación en Salud  Av. Cuauhtémoc 330, Col. Doctores  D.F.06720  México, D.F.  MEXICO |  |
|  | Catharina Ziekenhuis METC  Michelangelolaan 2  Eindhoven 5623 EJ  NETHERLANDS |
|  | Catharina Ziekenhuis METC  Michelangelolaan 2  Eindhoven  5623 EJ  NETHERLANDS |
|  | Catharina Ziekenhuis METC  Michelangelolaan 2  Eindhoven  5623 EJ  NETHERLANDS |
|  | Catharina Ziekenhuis METC  Michelangelolaan 2  Eindhoven  5623 EJ  NETHERLANDS |
|  | Catharina Ziekenhuis METC  Michelangelolaan 2  Eindhoven  5623 EJ  NETHERLANDS |
|  | Catharina Ziekenhuis METC  Michelangelolaan 2  Eindhoven  5623 EJ  NETHERLANDS |
|  | Catharina Ziekenhuis METC  Michelangelolaan 2  Eindhoven  5623 EJ  NETHERLANDS |
|  | Catharina Ziekenhuis METC  Michelangelolaan 2  Eindhoven  5623 EJ  NETHERLANDS |
|  | Catharina Ziekenhuis METC  Michelangelolaan 2  Eindhoven  5623 EJ  NETHERLANDS |
|  | Catharina Ziekenhuis METC  Michelangelolaan 2  Eindhoven  5623 EJ  NETHERLANDS |
|  | Catharina Ziekenhuis METC  Michelangelolaan 2  Eindhoven  5623 EJ  NETHERLANDS |
|  | Catharina Ziekenhuis METC  Michelangelolaan 2  Eindhoven  5623 EJ  NETHERLANDS |
|  | Catharina Ziekenhuis METC  Michelangelolaan 2  Eindhoven  5623 EJ  NETHERLANDS |
|  | Catharina Ziekenhuis METC  Michelangelolaan 2  Eindhoven  5623 EJ  NETHERLANDS |
|  | Catharina Ziekenhuis METC  Michelangelolaan 2  Eindhoven  5623 EJ  NETHERLANDS |
|  | Catharina Ziekenhuis METC  Michelangelolaan 2  Eindhoven  5623 EJ  NETHERLANDS |
|  | Catharina Ziekenhuis METC  Michelangelolaan 2  Eindhoven  5623 EJ  NETHERLANDS |
|  | Catharina Ziekenhuis METC  Michelangelolaan 2  Eindhoven  5623 EJ  NETHERLANDS |
|  | Catharina Ziekenhuis METC  Michelangelolaan 2  Eindhoven  5623 EJ  NETHERLANDS |
|  | Catharina Ziekenhuis METC  Michelangelolaan 2  Eindhoven  5623 EJ  NETHERLANDS |
|  | Catharina Ziekenhuis METC  Michelangelolaan 2  Eindhoven  5623 EJ  NETHERLANDS |
|  | Catharina Ziekenhuis METC  Michelangelolaan 2  Eindhoven  5623 EJ  NETHERLANDS |
|  | Catharina Ziekenhuis METC  Michelangelolaan 2  Eindhoven  5623 EJ  NETHERLANDS |
|  | Catharina Ziekenhuis METC  Michelangelolaan 2  Eindhoven  5623 EJ  NETHERLANDS |
|  | Catharina Ziekenhuis METC  Michelangelolaan 2  Eindhoven  5623 EJ  NETHERLANDS |
|  | Catharina Ziekenhuis METC  Michelangelolaan 2  Eindhoven  5623 EJ  NETHERLANDS |
|  | Catharina Ziekenhuis METC  Michelangelolaan 2  Eindhoven  5623 EJ  NETHERLANDS |
|  | Catharina Ziekenhuis METC  Michelangelolaan 2  Eindhoven  5623 EJ  NETHERLANDS |
|  | Catharina Ziekenhuis METC  Michelangelolaan 2  Eindhoven  5623 EJ  NETHERLANDS |
|  | Catharina Ziekenhuis METC  Michelangelolaan 2  Eindhoven  5623 EJ  NETHERLANDS |
|  | Catharina Ziekenhuis METC  Michelangelolaan 2  Eindhoven  5623 EJ  NETHERLANDS |
|  | Catharina Ziekenhuis METC  Michelangelolaan 2  Eindhoven  5623 EJ  NETHERLANDS |
| Multi-region Ethics Committee  Level 1, 1-3 The Terrace  PO BOX 5013  Wellington  6011  Wellington  NEW ZEALAND | Health and Disability Ethics Committee  Ethics Department  20 Aitken St  Thorndon  Wellington  NEW ZEALAND |
| Multi-region Ethics Committee  Level 1, 1-3 The Terrace  PO BOX 5013  Wellington  6011  Wellington  NEW ZEALAND | Health and Disability Ethics Committee  Ethics Department  20 Aitken St  Thorndon  Wellington  NEW ZEALAND |
| Multi-region Ethics Committee  Level 1, 1-3 The Terrace  PO BOX 5013  Wellington  6011  Wellington  NEW ZEALAND | Health and Disability Ethics Committee  Ethics Department  20 Aitken St  Thorndon  Wellington  NEW ZEALAND |
| Multi-region Ethics Committee  Level 1, 1-3 The Terrace  PO BOX 5013  Wellington  6011  Wellington  NEW ZEALAND | Health and Disability Ethics Committee  Ethics Department  20 Aitken St  Thorndon  Wellington  NEW ZEALAND |
| Multi-region Ethics Committee  Level 1, 1-3 The Terrace  PO BOX 5013  Wellington  6011  Wellington  NEW ZEALAND | Health and Disability Ethics Committee  Ethics Department  20 Aitken St  Thorndon  Wellington  NEW ZEALAND |
| Multi-region Ethics Committee  Level 1, 1-3 The Terrace  PO BOX 5013  Wellington  6011  Wellington  NEW ZEALAND | Health and Disability Ethics Committee  Ethics Department  20 Aitken St  Thorndon  Wellington  NEW ZEALAND |
| Multi-region Ethics Committee  Level 1, 1-3 The Terrace  PO BOX 5013  Wellington  6011  Wellington  NEW ZEALAND | Health and Disability Ethics Committee  Ethics Department  20 Aitken St  Thorndon  Wellington  NEW ZEALAND |
| Multi-region Ethics Committee  Level 1, 1-3 The Terrace  PO BOX 5013  Wellington  6011  Wellington  NEW ZEALAND | Health and Disability Ethics Committee  Ethics Department  20 Aitken St  Thorndon  Wellington  NEW ZEALAND |
| Multi-region Ethics Committee  Level 1, 1-3 The Terrace  PO BOX 5013  Wellington  6011  Wellington  NEW ZEALAND | Health and Disability Ethics Committee  Ethics Department  20 Aitken St  Thorndon  Wellington  NEW ZEALAND |
| Multi-region Ethics Committee  Level 1, 1-3 The Terrace  PO BOX 5013  Wellington  6011  Wellington  NEW ZEALAND | Health and Disability Ethics Committee  Ethics Department  20 Aitken St  Thorndon  Wellington  NEW ZEALAND |
| Multi-region Ethics Committee  Level 1, 1-3 The Terrace  PO BOX 5013  Wellington  6011  Wellington  NEW ZEALAND | Health and Disability Ethics Committee  Ethics Department  20 Aitken St  Thorndon  Wellington  NEW ZEALAND |
| Regional komité for medisinsk og helsefaglig forskningsetikk  REK Sør-Øst A  Postboks 1130, Blindern  Oslo  0318  NORWAY | Regional komité for medisinsk og helsefaglig forskningsetikk  REK Sør-Øst A  Postboks 1130, Blindern  Oslo  0318  NORWAY |
| Regional komité for medisinsk og helsefaglig forskningsetikk  REK Sør-Øst A  Postboks 1130, Blindern  Oslo  0318  NORWAY | Regional komité for medisinsk og helsefaglig forskningsetikk  REK Sør-Øst A  Postboks 1130, Blindern  Oslo  0318  NORWAY |
| Regional komité for medisinsk og helsefaglig forskningsetikk  REK Sør-Øst A  Postboks 1130, Blindern  Oslo  0318  NORWAY | Regional komité for medisinsk og helsefaglig forskningsetikk  REK Sør-Øst A  Postboks 1130, Blindern  Oslo  0318  NORWAY |
| Regional komité for medisinsk og helsefaglig forskningsetikk  REK Sør-Øst A  Postboks 1130, Blindern  Oslo 0318  NORWAY | Regional komité for medisinsk og helsefaglig forskningsetikk  REK Sør-Øst A  Postboks 1130, Blindern  Oslo 0318  NORWAY |
| Regional komité for medisinsk og helsefaglig forskningsetikk  REK Sør-Øst A  Postboks 1130, Blindern  Oslo  0318  NORWAY | Regional komité for medisinsk og helsefaglig forskningsetikk  REK Sør-Øst A  Postboks 1130, Blindern  Oslo  0318  NORWAY |
| Regional komité for medisinsk og helsefaglig forskningsetikk  REK Sør-Øst A  Postboks 1130, Blindern  Oslo  0318  NORWAY | Regional komité for medisinsk og helsefaglig forskningsetikk  REK Sør-Øst A  Postboks 1130, Blindern  Oslo  0318  NORWAY |
| Regional komité for medisinsk og helsefaglig forskningsetikk  REK Sør-Øst A  Postboks 1130, Blindern  Oslo  0318  NORWAY | Regional komité for medisinsk og helsefaglig forskningsetikk  REK Sør-Øst A  Postboks 1130, Blindern  Oslo  0318  NORWAY |
| Regional komité for medisinsk og helsefaglig forskningsetikk  REK Sør-Øst A  Postboks 1130, Blindern  Oslo  0318  NORWAY | Regional komité for medisinsk og helsefaglig forskningsetikk  REK Sør-Øst A  Postboks 1130, Blindern  Oslo  0318  NORWAY |
| Regional komité for medisinsk og helsefaglig forskningsetikk  REK Sør-Øst A  Postboks 1130, Blindern  Oslo  0318  NORWAY | Regional komité for medisinsk og helsefaglig forskningsetikk  REK Sør-Øst A  Postboks 1130, Blindern  Oslo  0318  NORWAY |
| Comite Institu. de Etica en Inv. Hosp. Nac. Arzobispo Loayza  Av. Alfonso Ugarte 848  Lima 1  PERU |  |
| CE de la asociación benéfica PRISMA  CARLOS GONZALES N° 251.URB MARANGA  Lima 32  PERU |  |
| Comité de Bioetca de la Red Asistencial Sabogal ESSALUD  Jr.Colina 1081, Bellavista, Callao –Peru  CALLAO 2  PERU |  |
| Comite Inst. de Etica en Inv. Hospital Hipolito Unanue  Av. César Vallejo 1390, El Agustino LIMA 10  PERU |  |
| CE de la asociación benéfica PRISMA  CARLOS GONZALES N° 251.URB MARANGA  Lima 32  PERU |  |
| CE de la asociación benéfica PRISMA  CARLOS GONZALES N° 251.URB MARANGA  Lima 32  PERU |  |
| CE de la asociación benéfica Prisma  Carlos Gonzales N° 251.Urb Maranga  Lima 32  PERU |  |
| CE de la asociación benéfica prisma  Carlos gonzales n° 251.urb maranga  Lima 32  PERU |  |
| CE de la asociación benéfica prisma  Carlos gonzales n° 251.urb maranga  Lima 32  PERU |  |
| CE de la asociación Benéfica prisma  Carlos gonzales N° 251.urb maranga  Lima 32  PERU |  |
| CE de la asociación benéfica prisma  Carlos gonzales n° 251.urb maranga  Lima 32  PERU |  |
| CE de la asociación benéfica Prisma  Carlos Gonzales N° 251.Urb Maranga  Lima 32  PERU |  |
| CE de la asociación benéfica prisma  Carlos gonzales n° 251.urb maranga  Lima 32  PERU |  |
| Hospital Nacional Carlos Seguin Escobedo  Esquina del Peral y Filtro s/n.  Arequipa  051  PERU |  |
| Clínica Angloamericana  Av. Alfredo Salazar Cdra 3  Lima 27  PERU |  |
| CE de la asociación benéfica prisma  Carlos gonzales n° 251.urb maranga  Lima 32  PERU |  |
| CE de la asociación Benéfica Prisma  Carlos Gonzales N° 251.Urb Maranga  Lima 32  PERU |  |
| CE de la asociación Benéfica Prisma  Carlos Gonzales N° 251.Urb Maranga  Lima 32  PERU |  |
| Comite de Etica del Hospital Nacional Cayetano Heredia  Av. Honorio Delgado 262 -San Martin de Porres Lima 31  PERU |  |
| CE de la asociación Benéfica Prisma  Carlos Gonzales N° 251.Urb Maranga  Lima 32  PERU |  |
| Philippine Heart Center (Ethics Review Committee)  East Avenue  Quezon  1100  PHILIPPINES |  |
| Ospital ng Makati Ethics Committee  Sampaguita Street  Brgy. Pembo, Makati City  PHILIPPINES |  |
| CDU-CDUH Institutional Ethics Review Committee  Cebu Doctors' University-Cebu Doctor's University Hospital  1 Dr. P. V. Larrazabal Jr. Avenue, North Reclamation  Mandaue City, Cebu  6014  PHILIPPINES |  |
| Medical Center Manila  1122 Gen. Luna Street, Ermita  Manila  1000  PHILIPPINES |  |
| St. Luke's Medical Center Instiutional Ethics Review Board  Research and Biotechnology Division  Main Building, St. Luke's Medical Center  279 E. Rodriguez Sr. Blvd.  Quezon City  1102  PHILIPPINES |  |
| St. Paul’s Hospital of IloIlo Institutional Review Board  General Luna Street  Iloilo 5000  PHILIPPINES |  |
| Medical Center Manila (Ethics Review Committee)  UN Avenue cor Taft Avenue, Ermita Manila  1000  PHILIPPINES |  |
| Quirino Memorial Medical Center Hospital Ethics Committee  Katipunan Road Extension  Project 4  Quezon  PHILIPPINES |  |
| Davao Doctor Hospital Institutional Ethics Review Board  118 E. Quirino Avenue,  Davao  PHILIPPINES |  |
| Community Health and Development Cooperative Hospital IRB  Room 200, Anda Riverside, Magallanes Interior, Davao City  8000  PHILIPPINES |  |
| De La Salle Health Sciences Institute Independent Ethics Com.  Rm 6301, De La Salle Angelo King Medical Research Center,  Congressional Avenue, Ground Floor  Cavite  PHILIPPINES |  |
| Perpetual Succour Hospital Inst. Ethics and Review Board  Room 501, SPC-Medical Specialty Center  Gorordo Avenue  Cebu  PHILIPPINES |  |
| Manila Doctors Hospital Institutional Review Board  Manila Doctors Hospital  667 United Nations Avenue  Ermita Manila  1000  PHILIPPINES |  |
|  | Komisja Bioetyczna przy Centrum Medycznego Ksztalcenia Podyp  ul. Marymoncka 99/103  Warszawa  01-813  Mazowieckie  POLAND |
|  | Komisja Bioetyczna przy Centrum Medycznego Ksztalcenia Podyp  ul. Marymoncka 99/103  Warszawa  01-813  Mazowieckie  POLAND |
|  | Komisja Bioetyczna przy Centrum Medycznego Ksztalcenia Podyp  ul. Marymoncka 99/103  Warszawa  01-813  Mazowieckie  POLAND |
|  | Komisja Bioetyczna przy Centrum Medycznego Ksztalcenia Podyp  ul. Marymoncka 99/103  Warszawa  01-813  Mazowieckie  POLAND |
|  | Komisja Bioetyczna przy Centrum Medycznego Ksztalcenia Podyp  ul. Marymoncka 99/103  Warszawa  01-813  Mazowieckie  POLAND |
|  | Komisja Bioetyczna przy Centrum Medycznego Ksztalcenia Podyp  ul. Marymoncka 99/103  Warszawa  01-813  Mazowieckie  POLAND |
|  | Komisja Bioetyczna przy Centrum Medycznego Ksztalcenia Podyp  ul. Marymoncka 99/103  Warszawa  01-813  Mazowieckie  POLAND |
|  | Komisja Bioetyczna przy Centrum Medycznego Ksztalcenia Podyp  ul. Marymoncka 99/103  Warszawa  01-813  Mazowieckie  POLAND |
|  | Komisja Bioetyczna przy Centrum Medycznego Ksztalcenia Podyp  ul. Marymoncka 99/103  Warszawa  01-813  Mazowieckie  POLAND |
|  | Komisja Bioetyczna przy Centrum Medycznego Ksztalcenia Podyp  ul. Marymoncka 99/103  Warszawa  01-813  Mazowieckie  POLAND |
|  | Komisja Bioetyczna przy Centrum Medycznego Ksztalcenia Podyp  ul. Marymoncka 99/103  Warszawa  01-813  Mazowieckie  POLAND |
|  | Komisja Bioetyczna przy Centrum Medycznego Ksztalcenia Podyp  ul. Marymoncka 99/103  Warszawa  01-813  Mazowieckie  POLAND |
|  | Komisja Bioetyczna przy Centrum Medycznego Ksztalcenia Podyp  ul. Marymoncka 99/103  Warszawa  01-813  Mazowieckie  POLAND |
|  | Komisja Bioetyczna przy Centrum Medycznego Ksztalcenia Podyp  ul. Marymoncka 99/103  Warszawa  01-813  Mazowieckie  POLAND |
|  | Komisja Bioetyczna przy Centrum Medycznego Ksztalcenia Podyp  ul. Marymoncka 99/103  Warszawa  01-813  Mazowieckie  POLAND |
|  | Komisja Bioetyczna przy Centrum Medycznego Ksztalcenia Podyp  ul. Marymoncka 99/103  Warszawa  01-813  Mazowieckie  POLAND |
|  | Komisja Bioetyczna przy Centrum Medycznego Ksztalcenia Podyp  ul. Marymoncka 99/103  Warszawa  01-813  Mazowieckie  POLAND |
|  | Komisja Bioetyczna przy Centrum Medycznego Ksztalcenia Podyp  ul. Marymoncka 99/103  Warszawa  01-813  Mazowieckie  POLAND |
|  | Komisja Bioetyczna przy Centrum Medycznego Ksztalcenia Podyp  ul. Marymoncka 99/103  Warszawa  01-813  Mazowieckie  POLAND |
|  | Komisja Bioetyczna przy Centrum Medycznego Ksztalcenia Podyp  ul. Marymoncka 99/103  Warszawa  01-813  Mazowieckie  POLAND |
|  | Komisja Bioetyczna przy Centrum Medycznego Ksztalcenia Podyp  ul. Marymoncka 99/103  Warszawa  01-813  Mazowieckie  POLAND |
|  | Komisja Bioetyczna przy Centrum Medycznego Ksztalcenia Podyp  ul. Marymoncka 99/103  Warszawa  01-813  Mazowieckie  POLAND |
|  | Komisja Bioetyczna przy Centrum Medycznego Ksztalcenia Podyp  ul. Marymoncka 99/103  Warszawa  01-813  Mazowieckie  POLAND |
|  | Komisja Bioetyczna przy Centrum Medycznego Ksztalcenia Podyp  ul. Marymoncka 99/103  Warszawa  01-813  Mazowieckie  POLAND |
|  | Komisja Bioetyczna przy Centrum Medycznego Ksztalcenia Podyp  ul. Marymoncka 99/103  Warszawa  01-813  Mazowieckie  POLAND |
|  | Komisja Bioetyczna przy Centrum Medycznego Ksztalcenia Podyp  ul. Marymoncka 99/103  Warszawa  01-813  Mazowieckie  POLAND |
|  | Komisja Bioetyczna przy Centrum Medycznego Ksztalcenia Podyp  ul. Marymoncka 99/103  Warszawa  01-813  Mazowieckie  POLAND |
|  | Komisja Bioetyczna przy Centrum Medycznego Ksztalcenia Podyp  ul. Marymoncka 99/103  Warszawa  01-813  Mazowieckie  POLAND |
|  | Komisja Bioetyczna przy Centrum Medycznego Ksztalcenia Podyp  ul. Marymoncka 99/103  Warszawa  01-813  Mazowieckie  POLAND |
|  | Komisja Bioetyczna przy Centrum Medycznego Ksztalcenia Podyp  ul. Marymoncka 99/103  Warszawa  01-813  Mazowieckie  POLAND |
|  | Komisja Bioetyczna przy Centrum Medycznego Ksztalcenia Podyp  ul. Marymoncka 99/103  Warszawa  01-813  Mazowieckie  POLAND |
|  | Komisja Bioetyczna przy Centrum Medycznego Ksztalcenia Podyp  ul. Marymoncka 99/103  Warszawa  01-813  Mazowieckie  POLAND |
|  | Komisja Bioetyczna przy Centrum Medycznego Ksztalcenia Podyp  ul. Marymoncka 99/103  Warszawa  01-813  Mazowieckie  POLAND |
|  | Komisja Bioetyczna przy Centrum Medycznego Ksztalcenia Podyp  ul. Marymoncka 99/103  Warszawa  01-813  Mazowieckie  POLAND |
|  | Komisja Bioetyczna przy Centrum Medycznego Ksztalcenia Podyp  ul. Marymoncka 99/103  Warszawa  01-813  Mazowieckie  POLAND |
|  | Komisja Bioetyczna przy Centrum Medycznego Ksztalcenia Podyp  ul. Marymoncka 99/103  Warszawa  01-813  Mazowieckie  POLAND |
|  | CEIC - Comissão de Ética para a Investigação Clínica  Parque da Saúde de Lisboa, Pav. 17-A  Avenida do Brasil nº 53  Lisboa 1749-004  PORTUGAL |
|  | CEIC - Comissão de Ética para a Investigação Clínica  Parque da Saúde de Lisboa, Pav. 17-A  Avenida do Brasil nº 53  Lisboa  1749-004  PORTUGAL |
|  | CEIC - Comissão de Ética para a Investigação Clínica  Parque da Saúde de Lisboa, Pav. 17-A  Avenida do Brasil nº 53  Lisboa 1749-004  PORTUGAL |
|  | CEIC - Comissão de Ética para a Investigação Clínica  Parque da Saúde de Lisboa, Pav. 17-A  Avenida do Brasil nº 53  Lisboa 1749-004  PORTUGAL |
|  | CEIC - Comissão de Ética para a Investigação Clínica  Parque da Saúde de Lisboa, Pav. 17-A  Avenida do Brasil nº 53  Lisboa  1749-004  PORTUGAL |
|  | CEIC - Comissão de Ética para a Investigação Clínica  Parque da Saúde de Lisboa, Pav. 17-A  Avenida do Brasil nº 53  Lisboa 1749-004  PORTUGAL |
|  | CEIC - Comissão de Ética para a Investigação Clínica  Parque da Saúde de Lisboa, Pav. 17-A  Avenida do Brasil nº 53  Lisboa 1749-004  PORTUGAL |
|  | CEIC - Comissão de Ética para a Investigação Clínica  Parque da Saúde de Lisboa, Pav. 17-A  Avenida do Brasil nº 53  Lisboa 1749-004  PORTUGAL |
|  | CEIC - Comissão de Ética para a Investigação Clínica  Parque da Saúde de Lisboa, Pav. 17-A  Avenida do Brasil nº 53  Lisboa 1749-004  PORTUGAL |
|  | National bioethics committee of medical product and medical devices  19-21 Stefan cel mare street  District 2  Bucharest 020125  ROMANIA |
|  | National bioethics committee of medical product and medical devices  19-21 Stefan cel mare street District 2  Bucharest 020125  ROMANIA |
|  | National bioethics committee of medical product and medical devices  19-21 Stefan cel mare street District 2  Bucharest 020125  ROMANIA |
|  | National bioethics committee of medical product and medical devices  19-21 Stefan cel mare street District 2  Bucharest 020125  ROMANIA |
|  | National bioethics committee of medical product and medical devices  19-21 Stefan cel mare street District 2  Bucharest 020125  ROMANIA |
|  | National bioethics committee of medical product and medical devices  19-21 Stefan cel mare street District 2  Bucharest 020125  ROMANIA |
|  | National bioethics committee of medical product and medical devices  19-21 Stefan cel mare street District 2  Bucharest 020125  ROMANIA |
|  | National bioethics committee of medical product and medical devices  19-21 Stefan cel mare street District 2  Bucharest 020125  ROMANIA |
|  | National bioethics committee of medical product and medical devices  19-21 Stefan cel mare street District 2  Bucharest 020125  ROMANIA |
|  | National bioethics committee of medical product and medical devices  19-21 Stefan cel mare street District 2  Bucharest 020125  ROMANIA |
|  | National bioethics committee of medical product and medical devices  19-21 Stefan cel mare street District 2  Bucharest 020125  ROMANIA |
|  | National bioethics committee of medical product and medical devices  19-21 Stefan cel mare street District 2  Bucharest 020125  ROMANIA |
|  | National bioethics committee of medical product and medical devices  19-21 Stefan cel mare street District 2  Bucharest 020125  ROMANIA |
|  | National bioethics committee of medical product and medical devices  19-21 Stefan cel mare street District 2  Bucharest 020125  ROMANIA |
|  | National bioethics committee of medical product and medical devices  19-21 Stefan cel mare street District 2  Bucharest 020125  ROMANIA |
|  | National bioethics committee of medical product and medical devices  19-21 Stefan cel mare street District 2  Bucharest 020125  ROMANIA |
|  | National bioethics committee of medical product and medical devices  19-21 Stefan cel mare street District 2  Bucharest 020125  ROMANIA |
| LEC of St-Petersburg State Medical University  10, Rentgena str.  St. Petersburg  197101  RUSSIAN FEDERATION | Ethics Board at Ministry of Health of the Russian Federation  3, Rakhmanovskiy per.,  Moscow  127994  RUSSIAN FEDERATION |
| LEC of the City Clinical Hospital #15  23, Veshnyakovskaya st.,  Moscow  111539  RUSSIAN FEDERATION | Ethics Board at Ministry of Health of the Russian Federation  3, Rakhmanovskiy per.,  Moscow  127994  RUSSIAN FEDERATION |
| LEC of Regional Clinical Hospital  1, Smirnovskoe uschelie  Saratov  410053  RUSSIAN FEDERATION | Ethics Board at Ministry of Health of the Russian Federation  3, Rakhmanovskiy per.,  Moscow  127994  RUSSIAN FEDERATION |
| The Independent Interdisciplinary Ethics Committee  51, Leningradsky ave.,  Moscow  125468  RUSSIAN FEDERATION | Ethics Board at Ministry of Health of the Russian Federation  3, Rakhmanovskiy per.,  Moscow  127994  RUSSIAN FEDERATION |
| LEC of the Kirov Military Medical Adademy  6, lit. Zh, Academika Lebedeva str.,  Saint-Petersburg  194044  RUSSIAN FEDERATION | Ethics Board at Ministry of Health of the Russian Federation  3, Rakhmanovskiy per.,  Moscow  127994  RUSSIAN FEDERATION |
| LEC of the City Hospital #38  7/2, bld. A, Hospitalnaya str., Pushkin District  Saint-Petersburg  196601  RUSSIAN FEDERATION | Ethics Board at Ministry of Health of the Russian Federation  3, Rakhmanovskiy per.,  Moscow  127994  RUSSIAN FEDERATION |
| Local EC of the Emergency Hospital#2  88/35, Bodraya str.,  Rostov-On-Don  344068  RUSSIAN FEDERATION | Ethics Board at Ministry of Health of the Russian Federation  3, Rakhmanovskiy per.,  Moscow  127994  RUSSIAN FEDERATION |
| LEC of the City Clinical Hospital n.a. N.A. Semashko  5, 8 Marta str.,  Yaroslavl  150002  RUSSIAN FEDERATION | Ethics Board at Ministry of Health of the Russian Federation  3, Rakhmanovskiy per.,  Moscow  127994  RUSSIAN FEDERATION |
| LEC of the Clinical Hospital №122  4, Prospect Kultury  Saint –Petersburg  194291  RUSSIAN FEDERATION | Ethics Board at Ministry of Health of the Russian Federation  3, Rakhmanovskiy per.,  Moscow  127994  RUSSIAN FEDERATION |
| LEC of the City Clinical Hospital #64  61, Vavilova Str.  Moscow  117292  RUSSIAN FEDERATION | Ethics Board at Ministry of Health of the Russian Federation  3, Rakhmanovskiy per.,  Moscow  127994  RUSSIAN FEDERATION |
| LEC of the Saratov State Medical University  B.Kazachya str.,  Saratov  410012  RUSSIAN FEDERATION | Ethics Board at Ministry of Health of the Russian Federation  3, Rakhmanovskiy per.,  Moscow  127994  RUSSIAN FEDERATION |
| LEC of the Kemerovo Cardiological Dispensary  6, Sosnovy boulevard, Kemerovo  650002  RUSSIAN FEDERATION | Ethics Board at Ministry of Health of the Russian Federation  3, Rakhmanovskiy per.,  Moscow  127994  RUSSIAN FEDERATION |
| LEC of the Saratov State Medical University  112, B. Kazachjya  Saratov  410012  RUSSIAN FEDERATION | Ethics Board at Ministry of Health of the Russian Federation  3, Rakhmanovskiy per.,  Moscow  127994  RUSSIAN FEDERATION |
| LEC of the Kirov Military Medical Adademy  6, lit. Zh, Academika Lebedeva str.,  Saint-Petersburg  194044  RUSSIAN FEDERATION | Ethics Board at Ministry of Health of the Russian Federation  3, Rakhmanovskiy per.,  Moscow  127994  RUSSIAN FEDERATION |
| The Independent Interdisciplinary Ethics Committee  51 Leningradskiy ave.  Moscow  125468  RUSSIAN FEDERATION | Ethics Board at Ministry of Health of the Russian Federation  3, Rakhmanovskiy per.,  Moscow  127994  RUSSIAN FEDERATION |
| LEC of the «Ryazan Regional Clinical Cardiology Dispensary»  96, Strojkova str.,  Ryazan  390026  RUSSIAN FEDERATION | Ethics Board at Ministry of Health of the Russian Federation  3, Rakhmanovskiy per.,  Moscow  127994  RUSSIAN FEDERATION |
| Ethics Committee at the Institution of Healthcare of Tyumen  build. 1, 7, 4th km Chervyshevskogo trakta  Tyumen  625000  RUSSIAN FEDERATION | Ethics Board at Ministry of Health of the Russian Federation  3, Rakhmanovskiy per.,  Moscow  127994  RUSSIAN FEDERATION |
| LEC of Kuban State Medical University  Sedina str., 4  Krasnodar  350063  RUSSIAN FEDERATION | Ethics Board at Ministry of Health of the Russian Federation  3, Rakhmanovskiy per.,  Moscow  127994  RUSSIAN FEDERATION |
| Ethics Committee of the Regional Clinical Cardiolgy Dispensa  15, Krymskaya street  Saratov  410039  RUSSIAN FEDERATION | Ethics Board at Ministry of Health of the Russian Federation  3, Rakhmanovskiy per.,  Moscow  127994  RUSSIAN FEDERATION |
| The Independent Interdisciplinary Ethics Committee  51 Leningradskiy ave.  Moscow  125468  RUSSIAN FEDERATION | Ethics Board at Ministry of Health of the Russian Federation  3, Rakhmanovskiy per.,  Moscow  127994  RUSSIAN FEDERATION |
| The Independent Interdisciplinary Ethics Committee  51 Leningradskiy ave.  Moscow  125468  RUSSIAN FEDERATION | Ethics Board at Ministry of Health of the Russian Federation  3, Rakhmanovskiy per.,  Moscow  127994  RUSSIAN FEDERATION |
| LEC of the Tyumen Cardiology Center  111a, Melnikaite str.,  Tyumen  625026  RUSSIAN FEDERATION | Ethics Board at Ministry of Health of the Russian Federation  3, Rakhmanovskiy per.,  Moscow  127994  RUSSIAN FEDERATION |
| LEC of the Altay Regional Cardiology Disensary  46, Malakhova str.,  Barnaul  656055  RUSSIAN FEDERATION | Ethics Board at Ministry of Health of the Russian Federation  3, Rakhmanovskiy per.,  Moscow  127994  RUSSIAN FEDERATION |
| LEC of the Russian Cardiological Research-Productive Complex  15A 3rd Cherepkovskaya str.  Moscow  121552  RUSSIAN FEDERATION | Ethics Board at Ministry of Health of the Russian Federation  3, Rakhmanovskiy per.,  Moscow  127994  RUSSIAN FEDERATION |
| LEC of the Central Clinical Hospital with outpatient departm  15, Marshala Timoshenko str.  Moscow121359  RUSSIAN FEDERATION | Ethics Board at Ministry of Health of the Russian Federation  3, Rakhmanovskiy per.,  Moscow  127994  RUSSIAN FEDERATION |
| LEC of the Kirov Military Medical Adademy  6, lit. Zh, Academika Lebedeva str.,  Saint-Petersburg  194044  RUSSIAN FEDERATION | Ethics Board at Ministry of Health of the Russian Federation  3, Rakhmanovskiy per.,  Moscow  127994  RUSSIAN FEDERATION |
| LEC of the City Clinical Hospital n.a. Eramishantsev  15, Lenskaya str.,  Moscow  129327  RUSSIAN FEDERATION | Ethics Board at Ministry of Health of the Russian Federation  3, Rakhmanovskiy per.,  Moscow  127994  RUSSIAN FEDERATION |
| LEC of the Research Center for Preventive Medicine  10, Petroverigskiy lane  Moscow  101990  RUSSIAN FEDERATION | Ethics Board at Ministry of Health of the Russian Federation  3, Rakhmanovskiy per.,  Moscow  127994  RUSSIAN FEDERATION |
| LEC of the City Clinical Hospital #25  1a, Alexandra Nevskogo str.  Novosibirsk  630075  RUSSIAN FEDERATION | Ethics Board at Ministry of Health of the Russian Federation  3, Rakhmanovskiy per.,  Moscow  127994  RUSSIAN FEDERATION |
| LEC of the Omsk City Clinical Hospital № 1  7, Perelуeta str., Omsk, 644112,  RUSSIAN FEDERATION | Ethics Board at Ministry of Health of the Russian Federation  3, Rakhmanovskiy per.,  Moscow  127994  RUSSIAN FEDERATION |
| LEC of the Kazan Medical Univercity  49, Butlerova str.,  Kazan 420012  RUSSIAN FEDERATION | Ethics Board at Ministry of Health of the Russian Federation  3, Rakhmanovskiy per.,  Moscow  127994  RUSSIAN FEDERATION |
| LEC of the Clinical Medical Sanitary  Part # 1  42, Soldatova str, Perm, 614111 Russia  68, Gagarina blvd, Perm, 614077  RUSSIAN FEDERATION | Ethics Board at Ministry of Health of the Russian Federation  3, Rakhmanovskiy per.,  Moscow  127994  RUSSIAN FEDERATION |
| LEC of the Pavlov Medical University  10, Rentgna str,  St-Petersburg  197022  RUSSIAN FEDERATION | Ethics Board at Ministry of Health of the Russian Federation  3, Rakhmanovskiy per.,  Moscow127994  RUSSIAN FEDERATION |
| LEC of the St. George the Martyr City Hospital  1, Severnyi avenue  Saint-Petersburg  194354  RUSSIAN FEDERATION | Ethics Board at Ministry of Health of the Russian Federation  3, Rakhmanovskiy per.,  Moscow  127994  RUSSIAN FEDERATION |
| LEC of the City Clinical Hospital #5  34, Nesterova str.,  Nizhniy Novgorod  603005  RUSSIAN FEDERATION | Ethics Board at Ministry of Health of the Russian Federation  3, Rakhmanovskiy per.,  Moscow  127994  RUSSIAN FEDERATION |
| LEC of the City Clinical Hospital n.a. Veresaev  10, Lobnenskaya str.  Moscow  127644  RUSSIAN FEDERATION | Ethics Board at Ministry of Health of the Russian Federation  3, Rakhmanovskiy per.,  Moscow  127994  RUSSIAN FEDERATION |
| LEC of the Emergency hospital  23, prospect Patriotov  Voronezh  394065  RUSSIAN FEDERATION | Ethics Board at Ministry of Health of the Russian Federation  3, Rakhmanovskiy per.,  Moscow  127994  RUSSIAN FEDERATION |
| LEC of Research Institute of Cardiology Siberia Brunch of RA  Kievskaya str. 111 A  Tomsk  634012  RUSSIAN FEDERATION | Ethics Board at Ministry of Health of the Russian Federation  3, Rakhmanovskiy per.,  Moscow  127994  RUSSIAN FEDERATION |
| LEC of the City Clinical Hospital #19  3, Shukshina str.,  Novosibirsk 630089  RUSSIAN FEDERATION | Ethics Board at Ministry of Health of the Russian Federation  3, Rakhmanovskiy per.,  Moscow  127994  RUSSIAN FEDERATION |
| LEC of the SAINT-PETERSBURG I.I. DZHANELIDZE RESEARCH INSTIT  3, Budapeshtskaya str.,  Saint-Petersburg  192242  RUSSIAN FEDERATION | Ethics Board at Ministry of Health of the Russian Federation  3, Rakhmanovskiy per.,  Moscow  127994  RUSSIAN FEDERATION |
| LEC of the Institute of Therapy and Preventive medicine  175/1, Borisa Bogatkova str.,  Novosibirsk 630089  RUSSIAN FEDERATION | Ethics Board at Ministry of Health of the Russian Federation  3, Rakhmanovskiy per.,  Moscow  127994  RUSSIAN FEDERATION |
| LEC of the Krasnodar Regional Clinical Hospital #1  167, Maya str.  Krasnodar 350086  RUSSIAN FEDERATION | Ethics Board at Ministry of Health of the Russian Federation  3, Rakhmanovskiy per.,  Moscow127994  RUSSIAN FEDERATION |
| LEC of the Pokrovskaya City Hospital  85, Bolshoi prosp., B.O.  St.-Petersburg  199106  RUSSIAN FEDERATION | Ethics Board at Ministry of Health of the Russian Federation  3, Rakhmanovskiy per.,  Moscow  127994  RUSSIAN FEDERATION |
| LEC Institute for Treatment and Rehabilitation „Niška Banja“  Srpskih Junaka 2  Niska Banja  18205  SERBIA |  |
| LEC of Clinical Hospital Centre Zemun  Vukova 9  Belgrade  11000  SERBIA |  |
| LEC of Clinical Hospital Center Bežanijska kosa  Bežanijska kosa b.b  11000  SERBIA |  |
| LEC of Clinical Center Nis  Bulevar Zorana Djindjica 48 Nis  18000  SERBIA |  |
| LEC of Military Medical Academy  Crnotravska 17  Belgrade  11000  SERBIA |  |
| LEC of Clinical Hospital Center Bežanijska kosa  Bežanijska kosa b.b  11000  SERBIA |  |
| LEC of Clinical Center of Serbia  Pasterova 2  Belgrade  11000  SERBIA |  |
| LEC of Clinical Center of Serbia  Pasterova 2  Belgrade  11000  SERBIA |  |
| LEC of Institute for Cardiovascular Diseases of Vojvodina  Put Dr. Goldmana 4  Sremska Kamenica  21204  SERBIA |  |
| LEC of Clinical Center of Serbia  Pasterova 2  Belgrade  11000  SERBIA |  |
| LEC of Dedinje Cardiovascular Institute  Heroja Milana Tepica 1  Belgrade  SERBIA |  |
| LEC of Clinical Center Kragujevac  Zmaj Jovina 30  Kragujevac  34000  SERBIA |  |
| LEC of Clinical Center of Serbia  Pasterova 2  Belgrade  11000  SERBIA |  |
| LEC of General Hospital Valjevo  Sinđelićeva 62  Valjevo  14000  SERBIA |  |
| LEC of Clinical Center Zvezdara  Dimitrija Tucovica 161  Belgrade  SERBIA |  |
| LEC of General Hospital Leskovac  Rade Končara 9  Leskovac  16000  SERBIA |  |
| LEC of General Hospital Sremska Mitrovica  Stari šor 65  Sremska Mitrovica  22000  SERBIA |  |
| LEC General Hospital Pancevo  Miloša Trebinjca 11  Pancevo 26000  SERBIA |  |
| Centralised Institutional Review Board  CIRB Board C  Singapore Health Service Pte Ltd. 31 Third Hospital Avenue  #03-03 Bowyer Block C  168753  SINGAPORE |  |
| NHG Domain Specific Review Board  c/o NHG HQ, Clinical Project Management & Planning Division  Research and Development Office  6 Commonwealth Lane  GMTI Bldg., #04-01/02  149547  SINGAPORE |  |
| NHG Domain Specific Review Board  c/o NHG HQ, Clinical Project Management & Planning Division  Research and Development Office  6 Commonwealth Lane  GMTI Bldg., #04-01/02  149547  SINGAPORE |  |
| EK Presovsky samospravny kraj  Namestie mieru 2  Presov  08001  SLOVAKIA | EK Univerzitnej nemocnice Bratislava - Stare Mesto  Mickiewiczova 13  Bratislava  81369  SLOVAKIA |
| Eticka komisia Kosickeho samospravneho kraja  Namestie Maratonu mieru 1  Kosice 04266  SLOVAKIA | EK Univerzitnej nemocnice Bratislava - Stare Mesto  Mickiewiczova 13  Bratislava  81369  SLOVAKIA |
| Eticka komisia Nitrianskeho samospravneho kraja  Stefanikova trieda 69  Nitra  94901  SLOVAKIA | EK Univerzitnej nemocnice Bratislava - Stare Mesto  Mickiewiczova 13  Bratislava  81369  SLOVAKIA |
| EK Presovsky samospravny kraj  Namestie mieru 2  Presov  08001  SLOVAKIA | EK Univerzitnej nemocnice Bratislava - Stare Mesto  Mickiewiczova 13  Bratislava  81369  SLOVAKIA |
| Eticka komisia Zilinskeho samospravneho kraja  Ul. Komenskeho 48  Zilina  01109  SLOVAKIA | EK Univerzitnej nemocnice Bratislava - Stare Mesto  Mickiewiczova 13  Bratislava  81369  SLOVAKIA |
| EK Banskobystrickeho samospravneho kraja  Namestie SNP 23  Banska Bystrica  974 01  SLOVAKIA | EK Univerzitnej nemocnice Bratislava - Stare Mesto  Mickiewiczova 13  Bratislava  81369  SLOVAKIA |
| Eticka komisia Kosickeho samospravneho kraja  Namestie Maratonu mieru 1  Kosice  04266  SLOVAKIA | EK Univerzitnej nemocnice Bratislava - Stare Mesto  Mickiewiczova 13  Bratislava  81369  SLOVAKIA |
| EK Univerzitnej nemocnice Bratislava - Stare Mesto  Mickiewiczova 13  Bratislava  81369  SLOVAKIA | EK Univerzitnej nemocnice Bratislava - Stare Mesto  Mickiewiczova 13  Bratislava  81369  SLOVAKIA |
| Eticka komisia Kosickeho samospravneho kraja  Namestie Maratonu mieru 1  Kosice  04266  SLOVAKIA | EK Univerzitnej nemocnice Bratislava - Stare Mesto  Mickiewiczova 13  Bratislava  81369  SLOVAKIA |
| EK Banskobystrickeho samospravneho kraja  Namestie SNP 23  Banska Bystrica  974 01  SLOVAKIA | EK Univerzitnej nemocnice Bratislava - Stare Mesto  Mickiewiczova 13  Bratislava  81369  SLOVAKIA |
| Eticka komisia Kosickeho samospravneho kraja  Namestie Maratonu mieru 1  Kosice  04266  SLOVAKIA | EK Univerzitnej nemocnice Bratislava - Stare Mesto  Mickiewiczova 13  Bratislava  81369  SLOVAKIA |
| Eticka komisia Bratislavskeho samospravneho kraja  Sabinovska 16  Bratislava  82005  SLOVAKIA | EK Univerzitnej nemocnice Bratislava - Stare Mesto  Mickiewiczova 13  Bratislava  81369  SLOVAKIA |
| Eticka komisia Univerzitnej nemocnice Bratislava  Nemocnica Ruzinov  Ruzinovska 6  Bratislava  82606  SLOVAKIA | EK Univerzitnej nemocnice Bratislava - Stare Mesto  Mickiewiczova 13  Bratislava  81369  SLOVAKIA |
| Eticka komisia Kosickeho samospravneho kraja  Namestie Maratonu mieru 1  Kosice  04266  SLOVAKIA | EK Univerzitnej nemocnice Bratislava - Stare Mesto  Mickiewiczova 13  Bratislava  81369  SLOVAKIA |
| EK Presovsky samospravny kraj  Namestie mieru 2  Presov  08001  SLOVAKIA | EK Univerzitnej nemocnice Bratislava - Stare Mesto  Mickiewiczova 13  Bratislava  81369  SLOVAKIA |
| Eticka komisia Kosickeho samospravneho kraja  Namestie Maratonu mieru 1  Kosice  04266  SLOVAKIA | EK Univerzitnej nemocnice Bratislava - Stare Mesto  Mickiewiczova 13  Bratislava  81369  SLOVAKIA |
| EK Univerzitnej nemocnice Bratislava - Stare Mesto  Mickiewiczova 13  Bratislava  81369  SLOVAKIA | EK Univerzitnej nemocnice Bratislava - Stare Mesto  Mickiewiczova 13  Bratislava  81369  SLOVAKIA |
| Eticka komisia Kosickeho samospravneho kraja  Namestie Maratonu mieru 1 Kosice  04266 SLOVAKIA | EK Univerzitnej nemocnice Bratislava - Stare Mesto  Mickiewiczova 13  Bratislava  81369  SLOVAKIA |
| Eticka komisia Zilinskeho samospravneho kraja  Ul. Komenskeho 48  Zilina 01109  SLOVAKIA | EK Univerzitnej nemocnice Bratislava - Stare Mesto  Mickiewiczova 13  Bratislava  81369  SLOVAKIA |
| Republic of Slovenia National Medical Ethics Committee  Zaloska 7, SI  University Medical Centre Ljubljana  Ljubljana  1525 SLOVENIA | Republic of Slovenia National Medical Ethics Committee  Zaloska 7, SI  University Medical Centre Ljubljana  Ljubljana 1525  SLOVENIA |
| Republic of Slovenia National Medical Ethics Committee  Zaloska 7, SI  University Medical Centre Ljubljana  Ljubljana  1525  SLOVENIA | Republic of Slovenia National Medical Ethics Committee  Zaloska 7, SI  University Medical Centre Ljubljana  Ljubljana  1525  SLOVENIA |
| Republic of Slovenia National Medical Ethics Committee  Zaloska 7, SI  University Medical Centre Ljubljana  Ljubljana  1525  SLOVENIA | Republic of Slovenia National Medical Ethics Committee  Zaloska 7, SI  University Medical Centre Ljubljana  Ljubljana  1525  SLOVENIA |
| Republic of Slovenia National Medical Ethics Committee  Zaloska 7, SI  University Medical Centre Ljubljana  Ljubljana  1525  SLOVENIA | Republic of Slovenia National Medical Ethics Committee  Zaloska 7, SI  University Medical Centre Ljubljana  Ljubljana  1525  SLOVENIA |
| Republic of Slovenia National Medical Ethics Committee  Zaloska 7, SI  University Medical Centre Ljubljana  Ljubljana  1525  SLOVENIA | Republic of Slovenia National Medical Ethics Committee  Zaloska 7, SI  University Medical Centre Ljubljana  Ljubljana  1525  SLOVENIA |
| South African Medical Association Research Ethics Committee  Castle Walk Corporate Park, Block F  Nossob Street  Erasmuskloof Ext.3  Pretoria  0153  SOUTH AFRICA | South African Medical Association Research Ethics Committee  Castle Walk Corporate Park, Block F  Nossob Street  Erasmuskloof Ext.3  Pretoria  0153  SOUTH AFRICA |
| South African Medical Association Research Ethics Committee  Castle Walk Corporate Park, Block F  Nossob Street  Erasmuskloof Ext.3  Pretoria  0153  SOUTH AFRICA | South African Medical Association Research Ethics Committee  Castle Walk Corporate Park, Block F  Nossob Street  Erasmuskloof Ext.3  Pretoria  0153  SOUTH AFRICA |
| South African Medical Association Research Ethics Committee  Castle Walk Corporate Park, Block F  Nossob Street  Erasmuskloof Ext.3  Pretoria  0153  SOUTH AFRICA | South African Medical Association Research Ethics Committee  Castle Walk Corporate Park, Block F  Nossob Street  Erasmuskloof Ext.3  Pretoria  0153  SOUTH AFRICA |
| South African Medical Association Research Ethics Committee  Castle Walk Corporate Park, Block F  Nossob Street  Erasmuskloof Ext.3  Pretoria  0153  SOUTH AFRICA | South African Medical Association Research Ethics Committee  Castle Walk Corporate Park, Block F  Nossob Street  Erasmuskloof Ext.3  Pretoria  0153  SOUTH AFRICA |
| South African Medical Association Research Ethics Committee  Castle Walk Corporate Park, Block F  Nossob Street  Erasmuskloof Ext.3  Pretoria  0153  SOUTH AFRICA | South African Medical Association Research Ethics Committee  Castle Walk Corporate Park, Block F  Nossob Street  Erasmuskloof Ext.3  Pretoria  0153  SOUTH AFRICA |
| South African Medical Association Research Ethics Committee  Castle Walk Corporate Park, Block F  Nossob Street  Erasmuskloof Ext.3  Pretoria  0153  SOUTH AFRICA | South African Medical Association Research Ethics Committee  Castle Walk Corporate Park, Block F  Nossob Street  Erasmuskloof Ext.3  Pretoria  0153  SOUTH AFRICA |
| South African Medical Association Research Ethics Committee  Castle Walk Corporate Park, Block F  Nossob Street  Erasmuskloof Ext.3  Pretoria  0153  SOUTH AFRICA | South African Medical Association Research Ethics Committee  Castle Walk Corporate Park, Block F  Nossob Street  Erasmuskloof Ext.3  Pretoria  0153  SOUTH AFRICA |
| South African Medical Association Research Ethics Committee  Castle Walk Corporate Park, Block F  Nossob Street  Erasmuskloof Ext.3  Pretoria  0153  SOUTH AFRICA | South African Medical Association Research Ethics Committee  Castle Walk Corporate Park, Block F  Nossob Street  Erasmuskloof Ext.3  Pretoria  0153  SOUTH AFRICA |
| UCT Human Research Ethics Committee  E53- 46 Old Main Building  Groote Schuur Hospital  Observatory  Cape Town  7925  SOUTH AFRICA | South African Medical Association Research Ethics Committee  Castle Walk Corporate Park, Block F  Nossob Street  Erasmuskloof Ext.3  Pretoria  0153  SOUTH AFRICA |
| South African Medical Association Research Ethics Committee  Castle Walk Corporate Park, Block F  Nossob Street  Erasmuskloof Ext.3  Pretoria  0153  SOUTH AFRICA | South African Medical Association Research Ethics Committee  Castle Walk Corporate Park, Block F  Nossob Street  Erasmuskloof Ext.3  Pretoria  0153  SOUTH AFRICA |
| South African Medical Association Research Ethics Committee  Castle Walk Corporate Park, Block F  Nossob Street  Erasmuskloof Ext.3  Pretoria  0153  SOUTH AFRICA | South African Medical Association Research Ethics Committee  Castle Walk Corporate Park, Block F  Nossob Street  Erasmuskloof Ext.3  Pretoria  0153  SOUTH AFRICA |
| South African Medical Association Research Ethics Committee  Castle Walk Corporate Park, Block F  Nossob Street  Erasmuskloof Ext.3  Pretoria  0153  SOUTH AFRICA | South African Medical Association Research Ethics Committee  Castle Walk Corporate Park, Block F  Nossob Street  Erasmuskloof Ext.3  Pretoria  0153  SOUTH AFRICA |
| South African Medical Association Research Ethics Committee  Castle Walk Corporate Park, Block F  Nossob Street  Erasmuskloof Ext.3  Pretoria  0153  SOUTH AFRICA | South African Medical Association Research Ethics Committee  Castle Walk Corporate Park, Block F  Nossob Street  Erasmuskloof Ext.3  Pretoria  0153  SOUTH AFRICA |
| University of the Free State Research Division  University of the Free Sate Research Division  205 Nelson Mandela Drive  Park West  Bloemfontein  9301  SOUTH AFRICA | South African Medical Association Research Ethics Committee  Castle Walk Corporate Park, Block F  Nossob Street  Erasmuskloof Ext.3  Pretoria  0153  SOUTH AFRICA |
| South African Medical Association Research Ethics Committee  Castle Walk Corporate Park, Block F  Nossob Street  Erasmuskloof Ext.3  Pretoria  0153  SOUTH AFRICA | South African Medical Association Research Ethics Committee  Castle Walk Corporate Park, Block F  Nossob Street  Erasmuskloof Ext.3  Pretoria  0153  SOUTH AFRICA |
| South African Medical Association Research Ethics Committee  Castle Walk Corporate Park, Block F  Nossob Street  Erasmuskloof Ext.3  Pretoria  0153  SOUTH AFRICA | South African Medical Association Research Ethics Committee  Castle Walk Corporate Park, Block F  Nossob Street  Erasmuskloof Ext.3  Pretoria  0153  SOUTH AFRICA |
| Stellenbosch University HREC1  Faculty of Health Sciences, Stellenbosch University  4th Floor Teaching Block  Francie van Zijl Drive  Parow  Cape Town  7500  SOUTH AFRICA | South African Medical Association Research Ethics Committee  Castle Walk Corporate Park, Block F  Nossob Street  Erasmuskloof Ext.3  Pretoria  0153  SOUTH AFRICA |
| South African Medical Association Research Ethics Committee  Castle Walk Corporate Park, Block F  Nossob Street  Erasmuskloof Ext.3  Pretoria  0153  SOUTH AFRICA | South African Medical Association Research Ethics Committee  Castle Walk Corporate Park, Block F  Nossob Street  Erasmuskloof Ext.3  Pretoria  0153  SOUTH AFRICA |
| South African Medical Association Research Ethics Committee  Castle Walk Corporate Park, Block F  Nossob Street  Erasmuskloof Ext.3  Pretoria  0153  SOUTH AFRICA | South African Medical Association Research Ethics Committee  Castle Walk Corporate Park, Block F  Nossob Street  Erasmuskloof Ext.3  Pretoria  0153  SOUTH AFRICA |
| South African Medical Association Research Ethics Committee  Castle Walk Corporate Park, Block F  Nossob Street  Erasmuskloof Ext.3  Pretoria  0153  SOUTH AFRICA | South African Medical Association Research Ethics Committee  Castle Walk Corporate Park, Block F  Nossob Street  Erasmuskloof Ext.3  Pretoria  0153  SOUTH AFRICA |
| South African Medical Association Research Ethics Committee  Castle Walk Corporate Park, Block F  Nossob Street  Erasmuskloof Ext.3  Pretoria  0153  SOUTH AFRICA | South African Medical Association Research Ethics Committee  Castle Walk Corporate Park, Block F  Nossob Street  Erasmuskloof Ext.3  Pretoria  0153  SOUTH AFRICA |
| Comité etico regional Comunidad de Madrid  Aduana,29  3ª planta  28013  Madrid  SPAIN | Comité etico regional Comunidad de Madrid  Aduana,29  3ª planta  28013  Madrid  SPAIN |
| Comité etico regional Comunidad de Madrid  Aduana,29  3ª planta  28013  Madrid  SPAIN | Comité etico regional Comunidad de Madrid  Aduana,29  3ª planta  28013  Madrid  SPAIN |
| Comité etico regional Comunidad de Madrid  Aduana,29  3ª planta  28013  Madrid  SPAIN | Comité etico regional Comunidad de Madrid  Aduana,29  3ª planta  28013  Madrid  SPAIN |
| Comité etico regional Comunidad de Madrid  Aduana,29  3ª planta  28013  Madrid  SPAIN | Comité etico regional Comunidad de Madrid  Aduana,29  3ª planta  28013  Madrid  SPAIN |
| Comité etico regional Comunidad de Madrid  Aduana,29  3ª planta  28013  Madrid  SPAIN | Comité etico regional Comunidad de Madrid  Aduana,29  3ª planta  28013  Madrid  SPAIN |
| Comité etico regional Comunidad de Madrid  Aduana,29  3ª planta  Madrid  28013  SPAIN | Comité etico regional Comunidad de Madrid  Aduana,29  3ª planta  Madrid  28013  SPAIN |
| Comité etico regional Comunidad de Madrid  Aduana,29  3ª planta  Madrid  28013  SPAIN | Comité etico regional Comunidad de Madrid  Aduana,29  3ª planta  Madrid  28013  SPAIN |
| Comité etico regional Comunidad de Madrid  Aduana,29  3ª planta  Madrid  28013  SPAIN | Comité etico regional Comunidad de Madrid  Aduana,29  3ª planta  Madrid  28013  SPAIN |
| Comité etico regional Comunidad de Madrid  Aduana,29  3ª planta  Madrid  28013  SPAIN | Comité etico regional Comunidad de Madrid  Aduana,29  3ª planta  Madrid  28013  SPAIN |
| Comité etico regional Comunidad de Madrid  Aduana,29  3ª planta  Madrid  28013  SPAIN | Comité etico regional Comunidad de Madrid  Aduana,29  3ª planta  Madrid  28013  SPAIN |
| Comité etico regional Comunidad de Madrid  Aduana,29  3ª planta  Madrid  28013  SPAIN | Comité etico regional Comunidad de Madrid  Aduana,29  3ª planta  Madrid  28013  SPAIN |
| Comité etico regional Comunidad de Madrid  Aduana,29  3ª planta  Madrid  28013  SPAIN | Comité etico regional Comunidad de Madrid  Aduana,29  3ª planta  Madrid  28013  SPAIN |
| Comité etico regional Comunidad de Madrid  Aduana,29  3ª planta  Madrid  28013  SPAIN | Comité etico regional Comunidad de Madrid  Aduana,29  3ª planta  Madrid  28013  SPAIN |
| Comité etico regional Comunidad de Madrid  Aduana,29  3ª planta  28013  Madrid  SPAIN | Comité etico regional Comunidad de Madrid  Aduana,29  3ª planta  28013  Madrid  SPAIN |
| Comité etico regional Comunidad de Madrid  Aduana,29  3ª planta  Madrid  28013  SPAIN | Comité etico regional Comunidad de Madrid  Aduana,29  3ª planta  Madrid  28013  SPAIN |
| Comité etico regional Comunidad de Madrid  Aduana,29  3ª planta  Madrid  28013  SPAIN | Comité etico regional Comunidad de Madrid  Aduana,29  3ª planta  Madrid  28013  SPAIN |
| Comité etico regional Comunidad de Madrid  Aduana,29  3ª planta  Madrid  28013  SPAIN | Comité etico regional Comunidad de Madrid  Aduana,29  3ª planta  Madrid  28013  SPAIN |
| Comité etico regional Comunidad de Madrid  Aduana,29  3ª planta  Madrid  28013  SPAIN | Comité etico regional Comunidad de Madrid  Aduana,29  3ª planta  Madrid  28013  SPAIN |
| Comité etico regional Comunidad de Madrid  Aduana,29  3ª planta  Madrid  28013  SPAIN | Comité etico regional Comunidad de Madrid  Aduana,29  3ª planta  Madrid  28013  SPAIN |
| Comité etico regional Comunidad de Madrid  Aduana,29  3ª planta  Madrid  28013  SPAIN | Comité etico regional Comunidad de Madrid  Aduana,29  3ª planta  Madrid  28013  SPAIN |
| Comité etico regional Comunidad de Madrid  Aduana,29  3ª planta  Madrid  28013  SPAIN | Comité etico regional Comunidad de Madrid  Aduana,29  3ª planta  Madrid  28013  SPAIN |
| Comité etico regional Comunidad de Madrid  Aduana,29  3ª planta  Madrid  28013  SPAIN | Comité etico regional Comunidad de Madrid  Aduana,29  3ª planta  Madrid  28013  SPAIN |
| Comité etico regional Comunidad de Madrid  Aduana,29  3ª planta  Madrid  28013  SPAIN | Comité etico regional Comunidad de Madrid  Aduana,29  3ª planta  Madrid  28013  SPAIN |
| Comité etico regional Comunidad de Madrid  Aduana,29  3ª planta  Madrid  28013  SPAIN | Comité etico regional Comunidad de Madrid  Aduana,29  3ª planta  Madrid  28013  SPAIN |
| Comité etico regional Comunidad de Madrid  Aduana,29  3ª planta  28013  Madrid  SPAIN | Comité etico regional Comunidad de Madrid  Aduana,29  3ª planta  28013  Madrid  SPAIN |
| Comité etico regional Comunidad de Madrid  Aduana,29  3ª planta  Madrid  28013  SPAIN | Comité etico regional Comunidad de Madrid  Aduana,29  3ª planta  Madrid  28013  SPAIN |
| Comité etico regional Comunidad de Madrid  Aduana,29  3ª planta  Madrid  28013  SPAIN | Comité etico regional Comunidad de Madrid  Aduana,29  3ª planta  Madrid  28013  SPAIN |
| Comité etico regional Comunidad de Madrid  Aduana,29  3ª planta  Madrid  28013  SPAIN | Comité etico regional Comunidad de Madrid  Aduana,29  3ª planta  Madrid  28013  SPAIN |
| Comité etico regional Comunidad de Madrid  Aduana,29  3ª planta  Madrid  28013  SPAIN | Comité etico regional Comunidad de Madrid  Aduana,29  3ª planta  Madrid  28013  SPAIN |
| Comité etico regional Comunidad de Madrid  Aduana,29  3ª planta  Madrid  28013  SPAIN | Comité etico regional Comunidad de Madrid  Aduana,29  3ª planta  Madrid  28013  SPAIN |
| Comité etico regional Comunidad de Madrid  Aduana,29  3ª planta  Madrid  28013  SPAIN | Comité etico regional Comunidad de Madrid  Aduana,29  3ª planta  Madrid  28013  SPAIN |
| Comité etico regional Comunidad de Madrid  Aduana,29  3ª planta  Madrid  28013  SPAIN | Comité etico regional Comunidad de Madrid  Aduana,29  3ª planta  Madrid  28013  SPAIN |
| Comité etico regional Comunidad de Madrid  Aduana,29  3ª planta  Madrid  28013  SPAIN | Comité etico regional Comunidad de Madrid  Aduana,29  3ª planta  Madrid  28013  SPAIN |
| Comité etico regional Comunidad de Madrid  Aduana,29  3ª planta  Madrid  28013  SPAIN | Comité etico regional Comunidad de Madrid  Aduana,29  3ª planta  Madrid  28013  SPAIN |
| Comité etico regional Comunidad de Madrid  Aduana,29  3ª planta  Madrid  28013  SPAIN | Comité etico regional Comunidad de Madrid  Aduana,29  3ª planta  Madrid  28013  SPAIN |
| Comité etico regional Comunidad de Madrid  Aduana,29  3ª planta  Madrid  28013  SPAIN | Comité etico regional Comunidad de Madrid  Aduana,29  3ª planta  Madrid  28013  SPAIN |
| Comité etico regional Comunidad de Madrid  Aduana,29  3ª planta  Madrid  28013  SPAIN | Comité etico regional Comunidad de Madrid  Aduana,29  3ª planta  Madrid  28013  SPAIN |
| Comité etico regional Comunidad de Madrid  Aduana,29  3ª planta  Madrid  28013  SPAIN | Comité etico regional Comunidad de Madrid  Aduana,29  3ª planta  Madrid  28013  SPAIN |
| Comité etico regional Comunidad de Madrid  Aduana,29  3ª planta  Madrid  28013  SPAIN | Comité etico regional Comunidad de Madrid  Aduana,29  3ª planta  Madrid  28013  SPAIN |
| Comité etico regional Comunidad de Madrid  Aduana,29  3ª planta  Madrid  28013  SPAIN | Comité etico regional Comunidad de Madrid  Aduana,29  3ª planta  Madrid  28013  SPAIN |
| Regionala Etikprövningsnämnden  Box 1964  Uppsala  751 49  SWEDEN | Regionala Etikprövningsnämnden  Box 1964  Uppsala  751 49  SWEDEN |
| Regionala Etikprövningsnämnden  Box 1964  Uppsala  751 49 SWEDEN | Regionala Etikprövningsnämnden  Box 1964  Uppsala  751 49 SWEDEN |
| Regionala Etikprövningsnämnden  Box 1964  Uppsala  751 49 SWEDEN | Regionala Etikprövningsnämnden  Box 1964  Uppsala  751 49 SWEDEN |
| Regionala Etikprövningsnämnden  Box 1964  Uppsala  751 49 SWEDEN | Regionala Etikprövningsnämnden  Box 1964  Uppsala  751 49 SWEDEN |
| Regionala Etikprövningsnämnden  Box 1964  Uppsala  751 49 SWEDEN | Regionala Etikprövningsnämnden  Box 1964  Uppsala  751 49 SWEDEN |
| Regionala Etikprövningsnämnden  Box 1964  Uppsala  751 49 SWEDEN | Regionala Etikprövningsnämnden  Box 1964  Uppsala  751 49 SWEDEN |
| Regionala Etikprövningsnämnden  Box 1964  Uppsala  751 49 SWEDEN | Regionala Etikprövningsnämnden  Box 1964  Uppsala  751 49 SWEDEN |
| Regionala Etikprövningsnämnden  Box 1964  Uppsala  751 49 SWEDEN | Regionala Etikprövningsnämnden  Box 1964  Uppsala  751 49 SWEDEN |
| Regionala Etikprövningsnämnden  Box 1964  Uppsala  751 49 SWEDEN | Regionala Etikprövningsnämnden  Box 1964  Uppsala  751 49 SWEDEN |
| Regionala Etikprövningsnämnden  Box 1964  Uppsala  751 49 SWEDEN | Regionala Etikprövningsnämnden  Box 1964  Uppsala  751 49 SWEDEN |
| Regionala Etikprövningsnämnden  Box 1964  Uppsala  751 49 SWEDEN | Regionala Etikprövningsnämnden  Box 1964  Uppsala  751 49 SWEDEN |
| Regionala Etikprövningsnämnden  Box 1964  Uppsala  751 49 SWEDEN | Regionala Etikprövningsnämnden  Box 1964  Uppsala  751 49 SWEDEN |
| Regionala Etikprövningsnämnden  Box 1964  Uppsala  751 49 SWEDEN | Regionala Etikprövningsnämnden  Box 1964  Uppsala  751 49 SWEDEN |
| Kantonale Ethikkommission Zürich  Stampfenbachstraße 121  Zürich 8090  SWITZERLAND |  |
| Commission cantonale d'éthique de la recherche CCER  Secrétariat de la CCER  Rue Adrien-Lachenal 8  Genève 1207  SWITZERLAND |  |
| Comitato Etico cantonale (Ticino)  c/o Ufficio di sanità  Via Orico 5  Bellinzona  6501  SWITZERLAND |  |
| Ethikkommission Nordwest- und Zentralschweiz (EKNZ)  Hebelstraße 53  Basel  4056  SWITZERLAND |  |
| Institute for the Development of Human Research Protections  Building 8, Floor 7, Room 702  Ministry of Public Health, Nonthaburi  Thailand  Nonthaburi  11000 THAILAND | Institute for the Development of Human Research Protections  Building 8, Floor 7, Room 702  Ministry of Public Health, Nonthaburi  Thailand  Nonthaburi  11000 THAILAND |
| Institute for the Development of Human Research Protections  Building 8, Floor 7, Room 702  Ministry of Public Health, Nonthaburi  11000  THAILAND | Institute for the Development of Human Research Protections  Building 8, Floor 7, Room 702  Ministry of Public Health, Nonthaburi  11000  THAILAND |
| Research Ethics Committee 2, Faculty of Medicine, Chiang Mai  Faculty of Medicine, Chiang Mai University,  110 Intavaroros Rd., Amphoe Muang,  Chiang Mai  50200  THAILAND | Institute for the Development of Human Research Protections  Building 8, Floor 7, Room 702  Ministry of Public Health, Nonthaburi  11000  THAILAND |
| Prince of Songkla University  Office of Human Research Ethics Committee, Faculty of Medicine, Prince of Songkla University  15 Karnjanavanit Rd. Hat Yai, Songkla 90110 THAILAND | Institute for the Development of Human Research Protections  Building 8, Floor 7, Room 702  Ministry of Public Health, Nonthaburi  THAILAND |
| Institute for the Development of Human Research Protections  Building 8, Floor 7, Room 702  Ministry of Public Health, Nonthaburi THAILAND | Institute for the Development of Human Research Protections  Building 8, Floor 7, Room 702  Ministry of Public Health, NonthaburiTHAILAND |
| Ethical Clearance Committee on Human Rights - Involving Huma  Ramathibodi Hospital, Mahidol University, Rama VI Road  10400 Bangkok  THAILAND | Institute for the Development of Human Research Protections  Building 8, Floor 7, Room 702  Ministry of Public Health, Nonthaburi 11000  THAILAND |
| Institute for the Development of Human Research Protections  Building 8, Floor 7, Room 702  Ministry of Public Health, Nonthaburi 11000  THAILAND | Institute for the Development of Human Research Protections  Building 8, Floor 7, Room 702  Ministry of Public Health, Nonthaburi 11000  THAILAND |
| Siriraj Institutional Review Board  2 Wanglang Road, Bangkoknoi  10700  THAILAND | Institute for the Development of Human Research Protections  Building 8, Floor 7, Room 702  Ministry of Public Health, Nonthaburi 11000  THAILAND |
| Institute for the Development of Human Research Protections  Building 8, Floor 7, Room 702  Ministry of Public Health,  Nonthaburi  11000  THAILAND | Institute for the Development of Human Research Protections  Building 8, Floor 7, Room 702  Ministry of Public Health,  Nonthaburi  11000  THAILAND |
| Institute for the Development of Human Research Protections  Building 8, Floor 7, Room 702  Ministry of Public Health, Nonthaburi  11000  THAILAND | Institute for the Development of Human Research Protections  Building 8, Floor 7, Room 702  Ministry of Public Health, Nonthaburi  11000  THAILAND |
| Ethical Review Committee of Royal Thai Army Medical Departme  315 Rajavithi Road  Rajathavee  10400  Bangkok  THAILAND | Institute for the Development of Human Research Protections  Building 8, Floor 7, Room 702  Ministry of Public Health, Nonthaburi  11000  THAILAND |
| Ethics Committee  Dokuz Eylül University School of Medicine Dean's Office  Inciralti  35340  IZMIR  TURKEY | Ethics Committee  Dokuz Eylül University School of Medicine Dean's Office  Inciralti  35340  IZMIR  TURKEY |
| Ethics Committee  Dokuz Eylül University School of Medicine Dean's Office  Inciralti  35340  IZMIR  TURKEY | Ethics Committee  Dokuz Eylül University School of Medicine Dean's Office  Inciralti  35340  IZMIR  TURKEY |
| Ethics Committee  Dokuz Eylül University School of Medicine Dean's Office  Inciralti  35340  IZMIR  TURKEY | Ethics Committee  Dokuz Eylül University School of Medicine Dean's Office  Inciralti  35340  IZMIR  TURKEY |
| Dokuz Eylül University School of Medicine Ethics Committee  Dean's Office  Inciralti  35340  IZMIR TURKEY | Dokuz Eylül University School of Medicine Ethics Committee  Dean's Office  Inciralti  35340  IZMIR TURKEY |
| Ethics Committee  Dokuz Eylül University School of Medicine Dean's Office  Inciralti  35340  IZMIR  TURKEY | Ethics Committee  Dokuz Eylül University School of Medicine Dean's Office  Inciralti  35340  IZMIR  TURKEY |
| Ethics Committee  Dokuz Eylül University School of Medicine Dean's Office  Inciralti  35340  IZMIR  TURKEY | Ethics Committee  Dokuz Eylül University School of Medicine Dean's Office  Inciralti  35340  IZMIR  TURKEY |
| Ethics Committee  Dokuz Eylül University School of Medicine Dean's Office  Inciralti  35340  IZMIR  TURKEY | Ethics Committee  Dokuz Eylül University School of Medicine Dean's Office  Inciralti  35340  IZMIR  TURKEY |
| Ethics Committee  Dokuz Eylül University School of Medicine Dean's Office  Inciralti  35340  IZMIR  TURKEY | Ethics Committee  Dokuz Eylül University School of Medicine Dean's Office  Inciralti  35340  IZMIR  TURKEY |
| Ethics Committee  Dokuz Eylül University School of Medicine Dean's Office  Inciralti  35340  IZMIR  TURKEY | Ethics Committee  Dokuz Eylül University School of Medicine Dean's Office  Inciralti  35340  IZMIR  TURKEY |
| Ethics Committee  Dokuz Eylül University School of Medicine Dean's Office  Inciralti  35340  IZMIR  TURKEY | Ethics Committee  Dokuz Eylül University School of Medicine Dean's Office  Inciralti  35340  IZMIR  TURKEY |
| Ethics Committee  Dokuz Eylül University School of Medicine Dean's Office  Inciralti  35340  IZMIR  TURKEY | Ethics Committee  Dokuz Eylül University School of Medicine Dean's Office  Inciralti  35340  IZMIR  TURKEY |
| Ethics Committee  Dokuz Eylül University School of Medicine Dean's Office  Inciralti  35340  IZMIR  TURKEY | Ethics Committee  Dokuz Eylül University School of Medicine Dean's Office  Inciralti  35340  IZMIR  TURKEY |
| LEC of City Clinical Hospital #1  121 Kharkivske Shose  Kyiv  02091  UKRAINE |  |
| LEC of Kyiv City Clinical Hospital of emergency medical care  3, Bratyslavska str  Kyiv  02660  UKRAINE |  |
| LEC of Kharkiv City Clinical Hospital #8  266-G Saltyvske shosse  Kharkiv  61176  UKRAINE |  |
| LEC of City clinical hospital #3  11, Lidersivsky ave  Odesa  65014  UKRAINE |  |
| LEC of City Clinical Hospital #1  96, Khmelnytske shosse  Vynnytsa  21029  UKRAINE |  |
| LEC of Kharkiv City Clinical Hospital #27  41 Pushkinska str.  Kharkiv  61002  UKRAINE |  |
| LEC of Zakarpatsky Regional Clinical Cardiology Dispensary  15A, Timiriazeva str.  Uzhorod 88000  UKRAINE |  |
| LEC of the Regional clinical hospital n.a. N.I. Pyrogov  46, Pyrogova Str.,  Vinnytsia  21018  UKRAINE |  |
| LEC of Institute of therapy n.a. L.T. Maloy  2-A Postusheva ave  Kharkiv  61039  UKRAINE |  |
| LEC of the Regional Clinical Cardiology Dispensary  114 Hetman Mazepa Str.,  Ivano-Frankivsk  76018  UKRAINE |  |
| LEC of the Central municipal clinical hospital  39/1, Shovkovychna str.,  Kyiv  01601  UKRAINE |  |
| LEC of the Institute of urgent and recovery surgery  47 Lenynsky ave  Donetsk  83045  UKRAINE |  |
| LEC of Odesa regional clinical hospital  26, Acad. Zabolotnogo str.  Odesa  65025  UKRAINE |  |
| LEC of the Central municipal clinical hospital  39/1, Shovkovychna str.,  Kyiv  01601  UKRAINE |  |
| LEC of Chernyvtsy Regional Clinical Cardiology Dispensary  230, Chervonoarmyska str.  Chernyvtsy  58013  UKRAINE |  |
| LEC of Institute of Cardiology n.a. M.D. Strazeska  5, Narodnogo Opolchennia str.  Kyiv  03151  UKRAINE |  |
| LEC of the Crimean State Medical University n.a.Georgievskiy  5/7, Lenina str.,  Simferopol  295006  UKRAINE |  |
| LEC of Dnipropetrovsk Regional Clinical Center of Cardiology  28, Plekhanova str.  Dnipropetrovsk  49060  UKRAINE |  |
| LEC of Khmelnytsky regional hospital  1 Pilotska str.  Khmelnytsky  29000  UKRAINE |  |
| LEc of Regional Clinical Hospital n.a. O.F.Gorbachevskogo  3, Chervonogo Khresta str.  Zhytomyr  10002  UKRAINE |  |
| LEC of Road Clinical Hospital #2  9, Povitroflotskyy ave.  Kyiv  03049  UKRAINE |  |
| LEC of Regional Medical Center of cardiovascular disease  78, Peremogy str.  Zhaporizzya  69005  UKRAINE |  |
| LEC of Central City Clinical Hospital #1  52a Rozy Liuksemburg str.  Donetsk  UKRAINE |  |
| LEC of Lutsk City Clinical Hospital  13, Vidrodzennia str.  Lutsk  43024  UKRAINE |  |
| LEC of City Hospital #1  4, Volodarskogo Str.  Mykolaiv  54003  UKRAINE |  |
| LEC of the Administration of medical services and rehabilita  17-A, Belorusskaia str.  Kyiv  UKRAINE |  |
| LEC of City Clinical Hospital of Emergency Medical Care  9 Mykolaichuka str.  Lviv  79059  UKRAINE |  |
| LEC of Central City Hospital #2  2-A Saburova str.  Zhytomyr  10004  UKRAINE |  |
| LEC of Lviv Regional Clinical Hospital  7 Chernihivska str.  Lviv  79010  UKRAINE |  |
| LEC of National Institute of Cardiovascular Surgery  6, Amosova str.  Kyiv  03110  UKRAINE |  |
| LEC of City Clinical Hospital #1  13, 20 Rokiv Peremohy str.  Sumu  40021  UKRAINE |  |
| LEC of Kyiv City Clinical Hospital #5  11 Vidpochynku str.  Kyiv  03115  UKRAINE |  |
|  | IEC Macedonian Agency for Medicines and Medical Devices  Sv. Kiril i Metodij No 54  Skopje  1000  MACEDONIA, FMR YUGOSLAV REP. |
|  | IEC Macedonian Agency for Medicines and Medical Devices  Sv. Kiril i Metodij No 54  Skopje  1000  MACEDONIA, FMR YUGOSLAV REP. |
|  | IEC Macedonian Agency for Medicines and Medical Devices  Sv. Kiril i Metodij No 54  Skopje  1000  MACEDONIA, FMR YUGOSLAV REP. |
|  | IEC Macedonian Agency for Medicines and Medical Devices  Sv. Kiril i Metodij No 54  Skopje  1000  MACEDONIA, FMR YUGOSLAV REP. |
|  | IEC Macedonian Agency for Medicines and Medical Devices  Sv. Kiril i Metodij No 54  Skopje  1000  MACEDONIA, FMR YUGOSLAV REP. |
|  | West of Scotland REC 1  Ground Floor  Tennant Institute  Western Infirmary  38 Church Street  Glasgow  G11 6NT  Strathclyde  UNITED KINGDOM |
|  | West of Scotland REC 1  Ground Floor  Tennant Institute  Western Infirmary  38 Church Street  Glasgow  G11 6NT  Strathclyde  UNITED KINGDOM |
|  | West of Scotland REC 1  Ground Floor  Tennant Institute  Western Infirmary  38 Church Street  Glasgow  G11 6NT  Strathclyde  UNITED KINGDOM |
|  | West of Scotland REC 1  Ground Floor  Tennant Institute  Western Infirmary  38 Church Street  Glasgow  G11 6NT  Strathclyde  UNITED KINGDOM |
|  | West of Scotland REC 1  Ground Floor  Tennant Institute  Western Infirmary  38 Church Street  Glasgow  G11 6NT  Strathclyde  UNITED KINGDOM |
|  | West of Scotland REC 1  Ground Floor  Tennant Institute  Western Infirmary  38 Church Street  Glasgow  G11 6NT  Strathclyde  UNITED KINGDOM |
|  | West of Scotland REC 1  Ground Floor  Tennant Institute  Western Infirmary  38 Church Street  Glasgow  G11 6NT  Strathclyde  UNITED KINGDOM |
|  | West of Scotland REC 1  Ground Floor  Tennant Institute  Western Infirmary  38 Church Street  Glasgow  G11 6NT  Strathclyde  UNITED KINGDOM |
|  | West of Scotland REC 1  Ground Floor  Tennant Institute  Western Infirmary  38 Church Street  Glasgow  G11 6NT  Strathclyde  UNITED KINGDOM |
|  | West of Scotland REC 1  Ground Floor  Tennant Institute  Western Infirmary  38 Church Street  Glasgow  G11 6NT  Strathclyde  UNITED KINGDOM |
|  | West of Scotland REC 1  Ground Floor  Tennant Institute  Western Infirmary  38 Church Street  Glasgow  G11 6NT  Strathclyde  UNITED KINGDOM |
|  | West of Scotland REC 1  Ground Floor  Tennant Institute  Western Infirmary  38 Church Street  Glasgow  G11 6NT  Strathclyde  UNITED KINGDOM |
|  | West of Scotland REC 1  Ground Floor  Tennant Institute  Western Infirmary  38 Church Street  Glasgow  G11 6NT  Strathclyde  UNITED KINGDOM |
|  | West of Scotland REC 1  Ground Floor  Tennant Institute  Western Infirmary  38 Church Street  Glasgow  G11 6NT  Strathclyde  UNITED KINGDOM |
|  | West of Scotland REC 1  Ground Floor  Tennant Institute  Western Infirmary  38 Church Street  Glasgow  G11 6NT  Strathclyde  UNITED KINGDOM |
|  | West of Scotland REC 1  Ground Floor  Tennant Institute  Western Infirmary  38 Church Street  Glasgow  G11 6NT  Strathclyde  UNITED KINGDOM |
|  | West of Scotland REC 1  Ground Floor  Tennant Institute  Western Infirmary  38 Church Street  Glasgow  G11 6NT  Strathclyde  UNITED KINGDOM |
|  | West of Scotland REC 1  Ground Floor  Tennant Institute  Western Infirmary  38 Church Street  Glasgow  G11 6NT  Strathclyde  UNITED KINGDOM |
|  | West of Scotland REC 1  Ground Floor  Tennant Institute  Western Infirmary  38 Church Street  Glasgow  G11 6NT  Strathclyde  UNITED KINGDOM |
|  | West of Scotland REC 1  Ground Floor  Tennant Institute  Western Infirmary  38 Church Street  Glasgow  G11 6NT  Strathclyde  UNITED KINGDOM |
|  | West of Scotland REC 1  Ground Floor  Tennant Institute  Western Infirmary  38 Church Street  Glasgow  G11 6NT  Strathclyde  UNITED KINGDOM |
|  | West of Scotland REC 1  Ground Floor  Tennant Institute  Western Infirmary  38 Church Street  Glasgow  G11 6NT  Strathclyde  UNITED KINGDOM |
|  | West of Scotland REC 1  Ground Floor  Tennant Institute  Western Infirmary  38 Church Street  Glasgow  G11 6NT  Strathclyde  UNITED KINGDOM |
|  | West of Scotland REC 1  Ground Floor  Tennant Institute  Western Infirmary  38 Church Street  Glasgow  G11 6NT  Strathclyde  UNITED KINGDOM |
|  | West of Scotland REC 1  Ground Floor  Tennant Institute  Western Infirmary  38 Church Street  Glasgow  G11 6NT  Strathclyde  UNITED KINGDOM |
|  | West of Scotland REC 1  Ground Floor  Tennant Institute  Western Infirmary  38 Church Street  Glasgow  G11 6NT  Strathclyde  UNITED KINGDOM |
|  | West of Scotland REC 1  Ground Floor  Tennant Institute  Western Infirmary  38 Church Street  Glasgow  G11 6NT  Strathclyde  UNITED KINGDOM |
|  | West of Scotland REC 1  Ground Floor  Tennant Institute  Western Infirmary  38 Church Street  Glasgow  G11 6NT  Strathclyde  UNITED KINGDOM |
|  | West of Scotland REC 1  Ground Floor  Tennant Institute  Western Infirmary  38 Church Street  Glasgow  G11 6NT  Strathclyde  UNITED KINGDOM |
|  | West of Scotland REC 1  Ground Floor  Tennant Institute  Western Infirmary  38 Church Street  Glasgow  G11 6NT  Strathclyde  UNITED KINGDOM |
|  | West of Scotland REC 1  Ground Floor  Tennant Institute  Western Infirmary  38 Church Street  Glasgow G11 6NT  Strathclyde  UNITED KINGDOM |
|  | West of Scotland REC 1  Ground Floor  Tennant Institute  Western Infirmary  38 Church Street  Glasgow G11 6NT  Strathclyde  UNITED KINGDOM |
|  | West of Scotland REC 1  Ground Floor  Tennant Institute  Western Infirmary  38 Church Street  Glasgow G11 6NT  Strathclyde  UNITED KINGDOM |
|  | West of Scotland REC 1  Ground Floor  Tennant Institute  Western Infirmary  38 Church Street  Glasgow  G11 6NT  Strathclyde  UNITED KINGDOM |
|  | West of Scotland REC 1  Ground Floor  Tennant Institute  Western Infirmary  38 Church Street  Glasgow  G11 6NT  Strathclyde  UNITED KINGDOM |
|  | Copernicus IRB Group, PO BOX 110605, Research Triangle Park, NC 27709, USA |
|  | Copernicus IRB Group, PO BOX 110605, Research Triangle Park, NC 27709, USA |
| Western Institutional Review Board, 1019 39th Ave., SE, Ste. 120, Puyallip, WA 98374, USA |  |
| Western Institutional Review Board, 3535 7th Ave., SW, PO Box 12029, Olympia, WA 98508, USA |  |
|  | Copernicus IRB Group, PO BOX 110605, Research Triangle Park, NC 27709, USA |
| V.A. Greater Los Angeles Healthcare System IRB, 11301 Wilshire Blvd., Bldg 114, Los Angeles, CA 90073, USA |  |
|  | Copernicus IRB Group, PO BOX 110605, Research Triangle Park, NC 27709, USA |
| Western Institutional Review Board, 3535 7th Ave., SW, PO Box 12029, Olympia, WA 98508, USA |  |
|  | Copernicus IRB Group, PO BOX 110605, Research Triangle Park, NC 27709, USA |
|  | Copernicus IRB Group, PO BOX 110605, Research Triangle Park, NC 27709, USA |
|  | Copernicus IRB Group, PO BOX 110605, Research Triangle Park, NC 27709, USA |
|  | Copernicus IRB Group, PO BOX 110605, Research Triangle Park, NC 27709, USA |
|  | Copernicus IRB Group, PO BOX 110605, Research Triangle Park, NC 27709, USA |
|  | Copernicus IRB Group, PO BOX 110605, Research Triangle Park, NC 27709, USA |
|  | Copernicus IRB Group, PO BOX 110605, Research Triangle Park, NC 27709, USA |
|  | Copernicus IRB Group, PO BOX 110605, Research Triangle Park, NC 27709, USA |
| Bon Secours Richmond Health System IRB, 8580 Magellan Pkwy., Richmond, VA 23227, USA |  |
| Western Institutional Review Board, 3535 7th Ave., SW, PO Box 12029, Olympia, WA 98508, USA |  |
| University of California, San Diego Human Research Protections Program, VA San Diego Healthcare System, 3350 La Jolla Village Dr., Mail Code 151A, San Diego, CA 92161, USA |  |
| Western Institutional Review Board, 1019 39th Ave., SE, Ste. 120, Puyallip, WA 98374, USA |  |
| Saint Vincent Indianapolis Hospital IRB, Saint Vincent Indianapolis Reseach and Regulatory Affairs, 8402 Harcourt Rd., Ste. 120, Indianapolis, IN 46260, USA |  |
|  | Copernicus IRB Group, PO BOX 110605, Research Triangle Park, NC 27709, USA |
| Guthrie Healthcare System IRB, One Guthrie Sq., Sayre, PA 18840, USA |  |
| Western Institutional Review Board, 3535 7th Ave., SW, PO Box 12029, Olympia, WA 98508, USA |  |
|  | Copernicus IRB Group, PO BOX 110605, Research Triangle Park, NC 27709, USA |
|  | Copernicus IRB Group, PO BOX 110605, Research Triangle Park, NC 27709, USA |
| Saint Joseph Mercy Hospital IRB, 5301 E. Huron River Dr., RHB 2018, Ann Arbor, MI, 48106, USA |  |
|  | Copernicus IRB Group, PO BOX 110605, Research Triangle Park, NC 27709, USA |
|  | Copernicus IRB Group, PO BOX 110605, Research Triangle Park, NC 27709, USA |
| Wellmont Health System IRB, 105 W Stone Dr., Ste. 6A, Kingsport, TN 37660, USA |  |
|  | Copernicus IRB Group, PO BOX 110605, Research Triangle Park, NC 27709, USA |
| Baptist Hospital IRB, 1000 W Moreno St., Pensacola, FL 32501, USA |  |
|  | Copernicus IRB Group, PO BOX 110605, Research Triangle Park, NC 27709, USA |
|  | Copernicus IRB Group, PO BOX 110605, Research Triangle Park, NC 27709, USA |
|  | Copernicus IRB Group, PO BOX 110605, Research Triangle Park, NC 27709, USA |
| University of Virginia IRB for Health Sciences Research, One Morton Dr., Ste. 400, Box 5, Charlottesville, VA 22903, USA |  |
|  | Copernicus IRB Group, PO BOX 110605, Research Triangle Park, NC 27709, USA |
|  | Copernicus IRB Group, PO BOX 110605, Research Triangle Park, NC 27709, USA |
| University of Vermont Committees on Human Research IRB, 85 S Prospect St., Burlington, VT, 05405, USA |  |
|  | Copernicus IRB Group, PO BOX 110605, Research Triangle Park, NC 27709, USA |
|  | Copernicus IRB Group, PO BOX 110605, Research Triangle Park, NC 27709, USA |
| Tufts Medical Center IRB, 800 Washington St., Box 817, Boston, MA 02111, USA |  |
| Corporate Institutional Review Board of Bellin Helth Sysem, 744 S. Webster Ave., PO BOX 23400, Green Bay, WI 54305, USA |  |
| Western Institutional Review Board, 3535 7th Ave., SW, PO Box 12029, Olympia, WA 98508, USA |  |
|  | Copernicus IRB Group, PO BOX 110605, Research Triangle Park, NC 27709, USA |
|  | Copernicus IRB Group, PO BOX 110605, Research Triangle Park, NC 27709, USA |
|  | Copernicus IRB Group, PO BOX 110605, Research Triangle Park, NC 27709, USA |
| Summa Health System Institutional Review Board, 55 Arch St., Ste. G1, Akron, OH 44304, USA |  |
|  | Copernicus IRB Group, PO BOX 110605, Research Triangle Park, NC 27709, USA |
|  | Copernicus IRB Group, PO BOX 110605, Research Triangle Park, NC 27709, USA |
| The Valley Hospital IRB, One Valley Health Plaza, Paramus, NJ 07432, USA |  |
| Southern California Institute for Research and Education, 5901 East 7th St., Long Beach, CA 90822, USA |  |
|  | Copernicus IRB Group, PO BOX 110605, Research Triangle Park, NC 27709, USA |
|  | Copernicus IRB Group, PO BOX 110605, Research Triangle Park, NC 27709, USA |
|  | Copernicus IRB Group, PO BOX 110605, Research Triangle Park, NC 27709, USA |
|  | Copernicus IRB Group, PO BOX 110605, Research Triangle Park, NC 27709, USA |
|  | Copernicus IRB Group, PO BOX 110605, Research Triangle Park, NC 27709, USA |
| Saint Joseph's Hospital of Atlanta IRB, 5673 Peachtree Dunwoody Rd., Ste. 550, Atlanta,GA 30342, USA |  |
|  | Copernicus IRB Group, PO BOX 110605, Research Triangle Park, NC 27709, USA |
|  | Copernicus IRB Group, PO BOX 110605, Research Triangle Park, NC 27709, USA |
| McGuire IRB, 1201 Broad Rock Blvd., Richmond, VA 23249, USA |  |
|  | Copernicus IRB Group, PO BOX 110605, Research Triangle Park, NC 27709, USA |
|  | Copernicus IRB Group, PO BOX 110605, Research Triangle Park, NC 27709, USA |
| Florida Hospital Tampa Bay Division IRB, Pepin Heart Institute, 3100 E Fletcher Ave., Tampa, FL 33613, USA |  |
|  | Copernicus IRB Group, PO BOX 110605, Research Triangle Park, NC 27709, USA |
|  | Copernicus IRB Group, PO BOX 110605, Research Triangle Park, NC 27709, USA |
|  | Copernicus IRB Group, PO BOX 110605, Research Triangle Park, NC 27709, USA |
|  | Copernicus IRB Group, PO BOX 110605, Research Triangle Park, NC 27709, USA |
|  | Copernicus IRB Group, PO BOX 110605, Research Triangle Park, NC 27709, USA |
| Human Subjects Committee Institutional Review Board, 3901 Rainbow Blvd., Kansas City, KS, 66160, USA |  |
| Trinity Medical Center IRB, 2701 17th St., Rock Island, IL 61201, USA |  |
| University of California, San Diego Human Research Protections Program, 9500 Gilman Dr., La Jolla, CA 92307, USA |  |
|  | Copernicus IRB Group, PO BOX 110605, Research Triangle Park, NC 27709, USA |
| Saint Cloud Hospital IRB, 1406 6th Ave. N., St. Cloud, MN 56303, USA |  |
| Trinity Hospital Institutional Review Board, One Burdick Expy.,W, Minot, ND 58701, USA |  |
|  | Copernicus IRB Group, PO BOX 110605, Research Triangle Park, NC 27709, USA |
|  | Copernicus IRB Group, PO BOX 110605, Research Triangle Park, NC 27709, USA |
|  | Copernicus IRB Group, PO BOX 110605, Research Triangle Park, NC 27709, USA |
|  | Copernicus IRB Group, PO BOX 110605, Research Triangle Park, NC 27709, USA |
|  | Copernicus IRB Group, PO BOX 110605, Research Triangle Park, NC 27709, USA |
|  | Copernicus IRB Group, PO BOX 110605, Research Triangle Park, NC 27709, USA |
| Western Institutional Review Board, 3535 7th Ave., SW, PO Box 12029, Olympia, WA 98508,USA |  |
|  | Copernicus IRB Group, PO BOX 110605, Research Triangle Park, NC 27709, USA |
|  | Copernicus IRB Group, PO BOX 110605, Research Triangle Park, NC 27709, USA |
|  | Copernicus IRB Group, PO BOX 110605, Research Triangle Park, NC 27709, USA |
|  | Copernicus IRB Group, PO BOX 110605, Research Triangle Park, NC 27709, USA |
| University of Minnesota Research Subjects Protection Programs, 420 Delaware St., SE., MMC 820, Minneapolis, MN 55455, USA |  |
| Huntsville Hospital IRB, 101 Sivley Rd., Huntsville, AL 35801, USA |  |
| AnMed Health IRB, 800 N. Fant St., Anderson, SC 29621, USA |  |
|  | Copernicus IRB Group, PO BOX 110605, Research Triangle Park, NC 27709, USA |
| UNTHSC at Fort Worth IRB, 3400 Camp Bowie Blvd, Fort Worth, TX 76107, USA |  |
|  | Copernicus IRB Group, PO BOX 110605, Research Triangle Park, NC 27709, USA |
|  | Copernicus IRB Group, PO BOX 110605, Research Triangle Park, NC 27709, USA |
|  | Copernicus IRB Group, PO BOX 110605, Research Triangle Park, NC 27709, USA |
| Mercy Hospital Saint Louis IRB, 621 S New Ballas Rd., Ste. 6002B, St. Louis, MO 63141, USA |  |
|  | Copernicus IRB Group, PO BOX 110605, Research Triangle Park, NC 27709, USA |
| Hunterdon Medical Center IRB, 2100 Wescott Dr., Flemington, NJ 08822, USA |  |
| Western Institutional Review Board, 3535 7th Ave., SW, PO Box 12029, Olympia, WA 98508, US |  |
| Memorial Hospital of Rhode Island IRB, 111 Brewer St., Pawtucket, RI 02860, USA |  |
| Western Institutional Review Board, 3535 7th Ave., SW, PO Box 12029, Olympia, WA 98508, USA |  |
|  | Copernicus IRB Group, PO BOX 110605, Research Triangle Park, NC 27709, USA |
| Research Participant Protection Office, Lehigh Valley Health Network, 1255 S Cedar Crest Blvd., Ste. 3500, Allentown, PA 18103, USA |  |
| University of Florida Health Center IRB, PO BOX 100173, Gainesville, FL 32610, USA |  |
|  | Copernicus IRB Group, PO BOX 110605, Research Triangle Park, NC 27709, USA |
|  | Copernicus IRB Group, PO BOX 110605, Research Triangle Park, NC 27709, USA |
|  | Copernicus IRB Group, PO BOX 110605, Research Triangle Park, NC 27709, USA |
|  | Copernicus IRB Group, PO BOX 110605, Research Triangle Park, NC 27709, USA |
| Western Institutional Review Board, 3535 7th Ave., SW, PO Box 12029, Olympia, WA 98508, USA |  |
|  | Copernicus IRB Group, PO BOX 110605, Research Triangle Park, NC 27709, USA |
| McLaren Health Care Human Research Protection Program, 2701 Cambridge Ct., Ste. 110, Auburn Hills, MI 48326, USA |  |
|  | Copernicus IRB Group, PO BOX 110605, Research Triangle Park, NC 27709, USA |
|  | Copernicus IRB Group, PO BOX 110605, Research Triangle Park, NC 27709, USA |
|  | Copernicus IRB Group, PO BOX 110605, Research Triangle Park, NC 27709, USA |
|  | Copernicus IRB Group, PO BOX 110605, Research Triangle Park, NC 27709, USA |
|  | Copernicus IRB Group, PO BOX 110605, Research Triangle Park, NC 27709, USA |
|  | Copernicus IRB Group, PO BOX 110605, Research Triangle Park, NC 27709, USA |
|  | Copernicus IRB Group, PO BOX 110605, Research Triangle Park, NC 27709, USA |
|  | Copernicus IRB Group, PO BOX 110605, Research Triangle Park, NC 27709, USA |
|  | Copernicus IRB Group, PO BOX 110605, Research Triangle Park, NC 27709, USA |
|  | Copernicus IRB Group, PO BOX 110605, Research Triangle Park, NC 27709, USA |
|  | Copernicus IRB Group, PO BOX 110605, Research Triangle Park, NC 27709, USA |
|  | Copernicus IRB Group, PO BOX 110605, Research Triangle Park, NC 27709, USA |
|  | Copernicus IRB Group, PO BOX 110605, Research Triangle Park, NC 27709, USA |
|  | Copernicus IRB Group, PO BOX 110605, Research Triangle Park, NC 27709, USA |
| New York Medical College IRB, Office of Research Adminstration, 40 Sunshine Cottage Rd., Valhalla, NY 10595, USA |  |
| Geisinger Health System IRB, 100 N. Academy Ave., Danville, PA 17822, USA |  |
| Saint Anthonys Medical Center, 10010 Kennerly Rd., St. Louis, MO 63128, USA |  |
| Holy Spirit Health System IRB, Heart Center, 3rd Floor, 503 North 21st St., Camp Hill, PA 17011, USA |  |
|  | Copernicus IRB Group, PO BOX 110605, Research Triangle Park, NC 27709, USA |
|  | Copernicus IRB Group, PO BOX 110605, Research Triangle Park, NC 27709, USA |
|  | Copernicus IRB Group, PO BOX 110605, Research Triangle Park, NC 27709, USA |
| University Hospital IRB, 1350 Walton Way, Augusta, GA, 30901, USA |  |
| Mayo Clinic Foundation IRB, 201 Bldg., Rm 4-60, 200 First St., SW, Rochester, MN 55905, USA |  |
|  | Copernicus IRB Group, PO BOX 110605, Research Triangle Park, NC 27709, USA |
|  | Copernicus IRB Group, PO BOX 110605, Research Triangle Park, NC 27709, USA |
| HealthEast IRB, 1700 University Ave., W, St. Paul, MD 55104, USA |  |
| Wellmont Health System IRB, 105 W. Stone Dr., Ste. 6A, Kingsport, TN 37660, USA |  |
| Oregon Health and Science University - Research Integrity Office, IRB L106-RI, 3181 SW Sam Jackson Park Rd., Portland, OR 97239, USA |  |
|  | Copernicus IRB Group, PO BOX 110605, Research Triangle Park, NC 27709, USA |
| Colorado Multiple IRB, Bldg 500, Rm N3214, 13001 East 17th Place, Aurora, CO 80045, USA |  |
|  | Copernicus IRB Group, PO BOX 110605, Research Triangle Park, NC 27709, USA |
| University of South Alabama Medical Science Foundation IRB, 307 N. University Blvd., CSAB 138, Mobile, AL 36688, USA |  |
| Suburban Hospital IRB, 8600 Old Georgetown Road, Attn. Executive Offices, Bethesda, MD 20814, USA |  |
|  | Copernicus IRB Group, PO BOX 110605, Research Triangle Park, NC 27709, USA |
| Peoria Institutional Review Board, One Illini Dr, Peoria, IL 61605, USA |  |
|  | Copernicus IRB Group, PO BOX 110605, Research Triangle Park, NC 27709, USA |
| Essentia Health IRB, Essentia Institute of Rural Health, 502 E 2nd St., 5AV-2, Duluth, MN 55805, USA |  |
| Johns Hopkins University IRB, 1620 McElderry St, Read Hall-B130, Baltimore, MD 21205, USA |  |
|  | Copernicus IRB Group, PO BOX 110605, Research Triangle Park, NC 27709, USA |
|  | Copernicus IRB Group, PO BOX 110605, Research Triangle Park, NC 27709, USA |
|  | Copernicus IRB Group, PO BOX 110605, Research Triangle Park, NC 27709, USA |
| MemorialCare Health System IRB, Long Beach Memorial Medical Center, 2801 Atlantic Ave, Long Beach, CA 90805, USA |  |
| McLaren Health Care Human Research Protection Program, 2701 Cambridge Ct., Ste 110, Auburn Hills, MI 48346, USA |  |
|  | Copernicus IRB Group, PO BOX 110605, Research Triangle Park, NC 27709, USA |
|  | Copernicus IRB Group, PO BOX 110605, Research Triangle Park, NC 27709, USA |
|  | Copernicus IRB Group, PO BOX 110605, Research Triangle Park, NC 27709, USA |
|  | Copernicus IRB Group, PO BOX 110605, Research Triangle Park, NC 27709, USA |
| Schulman Associates IRB, Inc., 4445 Lake Forest Dr., Ste. 300, Cincinnati, OH 45242, USA |  |
| Yale New Haven Hospital, West Pavilion 493, 20 York St., New Haven, CT 06510, USA |  |
|  | Copernicus IRB Group, PO BOX 110605, Research Triangle Park, NC 27709, USA |
| Biomedical Research Alliance of New York, LLC IRB, 1981 Marcus Ave., Ste. 210, Lake Success, NY 11042, USA |  |
|  | Copernicus IRB Group, PO BOX 110605, Research Triangle Park, NC 27709, USA |
|  | Copernicus IRB Group, PO BOX 110605, Research Triangle Park, NC 27709, USA |
|  | Copernicus IRB Group, PO BOX 110605, Research Triangle Park, NC 27709, USA |
|  | Copernicus IRB Group, PO BOX 110605, Research Triangle Park, NC 27709, USA |
|  | Copernicus IRB Group, PO BOX 110605, Research Triangle Park, NC 27709, USA |
| V.A. Pittsburgh Healthcare System, University Dr C 151CT-U, Pittsburgh, PA 15240, USA |  |
| Western Institutional Review Board, 3535 7th Ave., SW, PO Box 12029, Olympia, WA 98508, USA |  |
| University of Texas Health Science Center San Antonio IRB, 7703 Floyd Curl Dr., Rm 3114, Mail Code 7830, San Antonio, TX 78229, USA |  |
|  | Copernicus IRB Group, PO BOX 110605, Research Triangle Park, NC 27709, USA |
| MetroWest Medical Center Institutional Review Board, 85 Lincoln St.,MAB305, Framingham, MA 01702, USA |  |
|  | Copernicus IRB Group, PO BOX 110605, Research Triangle Park, NC 27709, USA |
|  | Copernicus IRB Group, PO BOX 110605, Research Triangle Park, NC 27709, USA |
|  | Copernicus IRB Group, PO BOX 110605, Research Triangle Park, NC 27709, USA |
|  | Copernicus IRB Group, PO BOX 110605, Research Triangle Park, NC 27709, USA |
|  | Copernicus IRB Group, PO BOX 110605, Research Triangle Park, NC 27709, USA |
|  | Copernicus IRB Group, PO BOX 110605, Research Triangle Park, NC 27709, USA |
|  | Copernicus IRB Group, PO BOX 110605, Research Triangle Park, NC 27709, USA |
|  | Copernicus IRB Group, PO BOX 110605, Research Triangle Park, NC 27709, USA |
|  | Copernicus IRB Group, PO BOX 110605, Research Triangle Park, NC 27709, USA |
|  | Copernicus IRB Group, PO BOX 110605, Research Triangle Park, NC 27709, USA |
|  | Copernicus IRB Group, PO BOX 110605, Research Triangle Park, NC 27709, USA |
| NYU Medical Center/Bellvue Hospital Institutional Review Board, 1 Park Ave., 6th Floor, New York, NY 10016, USA |  |
| Lancaster General Health IRB, 555 N Duke St., Lancaster, PA 17604, USA |  |
|  | Copernicus IRB Group, PO BOX 110605, Research Triangle Park, NC 27709, USA |
| Quorum Review, 1601 Fifth Ave., Ste. 100, Seattle, WA 98101, USA |  |
|  | Copernicus IRB Group, PO BOX 110605, Research Triangle Park, NC 27709, USA |
|  | Copernicus IRB Group, PO BOX 110605, Research Triangle Park, NC 27709, USA |
|  | Copernicus IRB Group, PO BOX 110605, Research Triangle Park, NC 27709, USA |
|  | Copernicus IRB Group, PO BOX 110605, Research Triangle Park, NC 27709, USA |
| Texas Health Resources IRB, 612 E Lamar Blvd, Ste 1212, Arlington, TX 76011, USA |  |
| Presbyterian Healthcare IRB, 200 Hawthorne Lane, Charlotte, NC 28204, USA |  |
|  | Copernicus IRB Group, PO BOX 110605, Research Triangle Park, NC 27709, USA |
| Aurora Health Care Subject Protection Program, Aurora IRB, 945 North 12th St., PO BOX 342 W310, Milwaukee, WI 53201, USA |  |
|  | Copernicus IRB Group, PO BOX 110605, Research Triangle Park, NC 27709, USA |
| Western Institutional Review Board, 1019 39th Ave., SE, Ste. 120, Puyallip, WA 98374, USA |  |
| Western Institutional Review Board, 1019 39th Ave., SE, Ste. 120, Puyallip, WA 98374, USA |  |
| Atlantic Health System IRB, 475 South St., Morristown, NJ 07960, USA |  |
|  | Copernicus IRB Group, PO BOX 110605, Research Triangle Park, NC 27709, USA |
|  | Copernicus IRB Group, PO BOX 110605, Research Triangle Park, NC 27709, USA |
|  | Copernicus IRB Group, PO BOX 110605, Research Triangle Park, NC 27709, USA |
|  | Copernicus IRB Group, PO BOX 110605, Research Triangle Park, NC 27709, USA |
| Chesapeake IRB, 7063 Columbia Gateway Dr., Ste 110, Columbia, MD 21046, USA |  |
| Virtua IRB, 175 Madison Ave., Mount Holly, NJ 08060, USA |  |
| Tulane University Human Research Protection Office IRB, 1440 Cannal St., Ste. 1705, TW-36, New Orleans, LA 70112, USA |  |
|  | Copernicus IRB Group, PO BOX 110605, Research Triangle Park, NC 27709, USA |
|  | Copernicus IRB Group, PO BOX 110605, Research Triangle Park, NC 27709, USA |
| Quorum Review, 1601 Fifth Ave., Ste. 110, Seattle, WA 98101, USA |  |
| University of California Los Angeles Office for Protection of Research Subjects, 11000 Kinross Ave, Ste. 102, Los Angeles, CA 90095, USA |  |
|  | Copernicus IRB Group, PO BOX 110605, Research Triangle Park, NC 27709, USA |
| University of Tennessee College of Medicine IRB, 960 E Third St., Ste. 102, Chattanooga, TN 37403, USA |  |
|  | Copernicus IRB Group, PO BOX 110605, Research Triangle Park, NC 27709, USA |
|  | Copernicus IRB Group, PO BOX 110605, Research Triangle Park, NC 27709, USA |
| Quorum Review, 1601 Fifth Ave., Ste. 100, Seattle, WA 98101, USA |  |
|  | Copernicus IRB Group, PO BOX 110605, Research Triangle Park, NC 27709, USA |
|  | Copernicus IRB Group, PO BOX 110605, Research Triangle Park, NC 27709, USA |
| University of Illinois at Chicago IRB, 1737 W Polk St., 203 Administrative Office Bldg-M/C 672, Chicago, IL 60612, USA |  |
|  | Copernicus IRB Group, PO BOX 110605, Research Triangle Park, NC 27709, USA |
|  | Copernicus IRB Group, PO BOX 110605, Research Triangle Park, NC 27709, USA |
|  | Copernicus IRB Group, PO BOX 110605, Research Triangle Park, NC 27709, USA |
|  | Copernicus IRB Group, PO BOX 110605, Research Triangle Park, NC 27709, USA |
|  | Copernicus IRB Group, PO BOX 110605, Research Triangle Park, NC 27709, USA |
|  | Copernicus IRB Group, PO BOX 110605, Research Triangle Park, NC 27709, USA |
|  | Copernicus IRB Group, PO BOX 110605, Research Triangle Park, NC 27709, USA |
| Baylor Research Institute IRB, 3310 Live Oak, Ste. 501, Dallas, TX 75204, USA |  |
|  | Copernicus IRB Group, PO BOX 110605, Research Triangle Park, NC 27709, USA |
|  | Copernicus IRB Group, PO BOX 110605, Research Triangle Park, NC 27709, USA |
|  | Copernicus IRB Group, PO BOX 110605, Research Triangle Park, NC 27709, USA |
|  | Copernicus IRB Group, PO BOX 110605, Research Triangle Park, NC 27709, USA |
| Kaiser Permanente of Southern California IRB, 393 E Walnut St., 4th Floor, Pasadena, CA 91188, USA |  |
|  | Copernicus IRB Group, PO BOX 110605, Research Triangle Park, NC 27709, USA |
|  | Copernicus IRB Group, PO BOX 110605, Research Triangle Park, NC 27709, USA |
|  | Copernicus IRB Group, PO BOX 110605, Research Triangle Park, NC 27709, USA |
|  | Copernicus IRB Group, PO BOX 110605, Research Triangle Park, NC 27709, USA |
|  | Copernicus IRB Group, PO BOX 110605, Research Triangle Park, NC 27709, USA |
|  | Copernicus IRB Group, PO BOX 110605, Research Triangle Park, NC 27709, USA |
|  | Copernicus IRB Group, PO BOX 110605, Research Triangle Park, NC 27709, USA |
| Western Institutional Review Board, 1019 39th Ave., SE, Ste. 120, Puyallip, WA 98374, USA |  |
|  | Copernicus IRB Group, PO BOX 110605, Research Triangle Park, NC 27709, USA |
|  | Copernicus IRB Group, PO BOX 110605, Research Triangle Park, NC 27709, USA |
| HealthPartners IRB, 640 Jackson St., St Paul, MN 55101, USA |  |
|  | Copernicus IRB Group, PO BOX 110605, Research Triangle Park, NC 27709, USA |
|  | Copernicus IRB Group, PO BOX 110605, Research Triangle Park, NC 27709, USA |
|  | Copernicus IRB Group, PO BOX 110605, Research Triangle Park, NC 27709, USA |
|  | Copernicus IRB Group, PO BOX 110605, Research Triangle Park, NC 27709, USA |
|  | Copernicus IRB Group, PO BOX 110605, Research Triangle Park, NC 27709, USA |
|  | Copernicus IRB Group, PO BOX 110605, Research Triangle Park, NC 27709, USA |
| University of Oklahoma Health Sciences Center IRB, 1000 Stanton L. Young Blvd., Rm 176, Oklahoma City, OK 73117, USA |  |
| North Kansas City Hospital IRB, 2800 Clay Edwards Dr., North Kansas City, MO 64116, USA |  |
|  | Copernicus IRB Group, PO BOX 110605, Research Triangle Park, NC 27709, USA |
|  | Copernicus IRB Group, PO BOX 110605, Research Triangle Park, NC 27709, USA |
| University of Pennsylvania Office of Regulatory Affairs IRB, Office of Regulatory Affairs, IRB #6, 3624 Market St., Ste 301S, Philadelphia, PA 19104, USA |  |
| Presence Saint Joseph Medical Center IRB, 333 N Madison St., Joliet, IL 60435, USA |  |
|  | Copernicus IRB Group, PO BOX 110605, Research Triangle Park, NC 27709, USA |
|  | Copernicus IRB Group, PO BOX 110605, Research Triangle Park, NC 27709, USA |
| Western Institutional Review Board, 1019 39th Ave., SE, Ste. 120, Puyallip, WA 98374, USA |  |
| Western Institutional Review Board, 3535 7th Ave., SW, PO Box 12029, Olympia, WA 98508, USA |  |
|  | Copernicus IRB Group, PO BOX 110605, Research Triangle Park, NC 27709, USA |
| Virtua IRB, 175 Madison Ave., Mount Holly, NJ 08060, USA |  |
| Shady Grove Adventist Hospital IRB, 9901 Medical Center Dr., Rockville, MD 20850, USA |  |
| Rockingham Memorial Hospital IRB, 2010 Health Campus Drive, Harrisonburg, VA 22801, USA |  |
|  | Copernicus IRB Group, PO BOX 110605, Research Triangle Park, NC 27709, USA |
| Western Institutional Review Board, 1019 39th Ave., SE, Ste. 120, Puyallip, WA 98374, USA |  |
| Western Institutional Review Board, 1019 39th Ave., SE, Ste. 120, Puyallip, WA 98374, USA |  |
|  | Copernicus IRB Group, PO BOX 110605, Research Triangle Park, NC 27709, USA |
|  | Copernicus IRB Group, PO BOX 110605, Research Triangle Park, NC 27709, USA |
| Thomas Jefferson University IRB, Beacon Health System, 600 East Blvd, Elkhart, IN 46514, USA |  |
| Western Institutional Review Board, 1019 39th Ave., SE, Ste. 120, Puyallip, WA 98374, USA |  |
|  | Copernicus IRB Group, PO BOX 110605, Research Triangle Park, NC 27709, USA |
|  | Copernicus IRB Group, PO BOX 110605, Research Triangle Park, NC 27709, USA |
|  | Copernicus IRB Group, PO BOX 110605, Research Triangle Park, NC 27709, USA |
|  | Copernicus IRB Group, PO BOX 110605, Research Triangle Park, NC 27709, USA |
|  | Copernicus IRB Group, PO BOX 110605, Research Triangle Park, NC 27709, USA |
| Comité de Bioética del IIC de Rosario  Paraguay 160  Rosario  S2000CVD  Santa Fe  ARGENTINA |  |
| Bellberry Limited  129 Glen Osmond Road  Eastwood  5063  South Australia  AUSTRALIA |  |
| Hunter New England Human Research Ethics Committee  Lookout Road  New Lambton Heights  2305  New South Wales  AUSTRALIA |  |
| Hunter New England Human Research Ethics Committee  Lookout Road  New Lambton Heights  2305  New South Wales  AUSTRALIA |  |
|  | Ethik-Kommission für das Bundesland Salzburg  Sebastian-Stief-Gasse 2  Salzburg  5020  AUSTRIA |
|  | Commissie voor Medische Ethiek/Klinisch onderzoek  Herestraat 49  Leuven 3000  BELGIUM |
|  | Commissie voor Medische Ethiek/Klinisch onderzoek  Herestraat 49  Leuven 3000  BELGIUM |
|  | Ethics Committee Multicenter Trials  5 Sveta Nedelya Square  Sofia 1000  BULGARIA |
| Saint Michael's Hospital Research Ethics Board, 30 Bond St., Victoria Wing, 7th Floor, Rm7008, Toronto, ON M5B 1W8,  CANADA |  |
|  | Institutional Review Board Services, 372 Hollandview Trail, Ste. 300, Aurora, ON L4G 0A5, CANADA |
|  | Institutional Review Board Services, 372 Hollandview Trail, Ste. 300, Aurora, ON L4G 0A5, CANADA |
| University of Alberta Health Research Ethics Board, 308 Campus Tower, 8625-112th St., Edmonton, AB T6G 1K8, CANADA |  |
|  | Institutional Review Board Services, 372 Hollandview Trail, Ste. 300, Aurora, ON L4G 0A5, CANADA |
| University of British Columbia Clinical Ethics Research Board, 828 W 10th Ave., Vancouver, BC V5Z 1L8, CANADA |  |
| Tri-Hospital Research Ethics Board, Grand River Hospital  835 King Street West, Kaufman BLDG K 503  Kitchener, ON N2G 1G3 CANADA |  |
| Comite d'ethique de la recherche IRB, Hopital du Sacre-Cœur de Montreal, 5400 Boul Gouin Quest, Montreal, QC H4J 1C5, CANADA |  |
| Western University Health Science Research Ethics Board, Office of Research Ethics, University of Western Ontario, Rm 5150 Support Services Bldg, London, ON N6A 3K7,  CANADA |  |
| Shin-Kong Wu Ho-Su Memorial Hospital  No. 95, Wen Chang Road, Shih Lin District  Taipei  111  TAIWAN, PROVINCE OF CHINA |  |
| Comité de Etica En Investigación Clínica (CEI) Clínica Marly Calle 50 No. 9-67 (off 611)  COLOMBIA |  |
| Comité de Bioética en Investigación Clínica Farallones  Calle 9C N° 50 - 25 Cali-Valle  COLOMBIA |  |
| Eticka komise Ustredni vojenske nemocnice Praha  U Vojenske nemocnice 1200  Praha 6  16902  CZECH REPUBLIC | EK pro multicentrické klinické hodnocení FN v Motole  V uvalu 84  Praha 5  15006  CZECH REPUBLIC |
|  | CPP IDF 10  Hôpital Robert Ballanger  Batiment central n°8 (3ème étage)  boulevard Robert Ballanger  Aulnay-Sous-Bois Cedex  93602  FRANCE |
|  | CPP IDF 10  Hôpital Robert Ballanger  Batiment central n°8 (3ème étage)  boulevard Robert Ballanger  Aulnay-Sous-Bois Cedex  93602  FRANCE |
|  | CPP IDF 10  Hôpital Robert Ballanger  Batiment central n°8 (3ème étage)  boulevard Robert Ballanger  Aulnay-Sous-Bois Cedex  93602  FRANCE |
| LEC of LTD cardiologic clinic GULI  9, Tsinandali str.,  Tbilisi 144  GEORGIA |  |
|  | Ethik-Kommission an der  Medizinischen Fakultät der RWTH Aachen  Pauwelsstraße 30  Aachen  52074  GERMANY |
|  | Ethik-Kommission an der  Medizinischen Fakultät der RWTH Aachen  Pauwelsstraße 30  Aachen  52074  GERMANY |
|  | Ethik-Kommission an der  Medizinischen Fakultät der RWTH Aachen  Pauwelsstraße 30  Aachen  52074  GERMANY |
|  | Ethik-Kommission an der  Medizinischen Fakultät der RWTH Aachen  Pauwelsstraße 30  Aachen  52074  GERMANY |
| Dr. Ramesh Cardiac and Multispeciality Hospital P Ltd.,  Ring Road, Near ITI College, Vijaywada-520 008  INDIA |  |
| Fortis Escorts Hospital  JLN Marg, Malviya Nagar  Jaipur 302017  INDIA |  |
| Westfort Hi-Tech Hospital Ltd  P.B. No. 930, Punkunnam,  Thrissur- 680 002  INDIA |  |
| EC Lokmanya Tilak Municipal Medical College & General Hospital  2nd floor, college building,  Dr.B.R.Ambedkar Road, Sion,Mumbai -400022  INDIA |  |
| EC Lokmanya Tilak Municipal Medical College & General Hospital  2nd floor, college building,  Dr.B.R.Ambedkar Road, Sion,Mumbai -400022  INDIA |  |
| Karnataka Institute of Medical Sciences  Office of Principal,  Hubali  580021 INDIA |  |
| Aster Aadhar Ethics Committee (AAEC)  KMT Workshop, Kolhapur – 416012  Maharashtra  INDIA |  |
| Comitato Bioetico Sperim. del Farmaco A. O.Cannizzaro  Via Messina, 829  95126 Catania  ITALY |  |
| Comitato Etico Area Vasta Sud Est  Via Senese, 161  Sede Ospedale Misericordia  58100 Grosseto  ITALY |  |
| Comitato Etico Interaziendale M.  Corso Spezia, 60  Torino 10100  ITALY |  |
| Comitato Etico della ASL TO/2  Corso Svizzera, 185 bis  Torino  10149  ITALY |  |
| Comitato Etico Interaziendale A.O.“Città della Salute  e della Scienza" di Torino - A.O. ordine Mauriziano di Torino  ASL TO1 Corso Bramante, 88  Torino  10126  ITALY |  |
| Università Campus Bio-Medico  Comitato Etico  VIA ALVARO DEL PORTILLO, 200  00128  Roma  ITALY |  |
| Institutional Review Board of Gachon University Gil Hospital  1198, Guwol-dong,  Namdong-gu,  Incheon  405-760  Incheon  KOREA, REPUBLIC OF |  |
| Comité de Etica, Investigación y Bioseguridad Hospital Bernardette, S.C.  Av. Hidalgo 930 Col. Artesanos C.P. 44200 Guadalajara, Jalisco  MEXICO |  |
| Comité de Etica e Investigación Christus Mugerza del Parque  Pedro Leal Rodríguez 1802 Col. Centro C.P. 31000 Chihuahua, Chihuahua  MEXICO |  |
| Comité de Etica Hospital General León  20 de Enero No. 927 Col. Obregón. C.P. 37320 León Guanajuato  MEXICO |  |
| Comite de Etica del Hospital 1o de Octubre ISSSTE  Av. Insituto Politecnico Nacional # 1669  Col. Lindavista  Mexico City  07300  MEXICO |  |
| Comite de Etica del Hospital 1o de Octubre ISSSTE  Av. Insituto Politecnico Nacional # 1669  Col. Lindavista  Mexico City  07300  MEXICO |  |
|  | Catharina Ziekenhuis METC  Michelangelolaan 2  Eindhoven 5623 EJ NETHERLANDS |
| Multi-region Ethics Committee  NZ Multi-region EC  Level 1, 1-3 The Terrace  PO BOX 5013  Wellington  6011  Wellington  NEW ZEALAND | Health and Disability Ethics Committee  Ethics Department  20 Aitken St  Thorndon  Wellington  NEW ZEALAND |
| Regional komité for medisinsk og helsefaglig forskningsetikk  REK Sør-Øst A  Postboks 1130, Blindern  Oslo  0318  NORWAY | Regional komité for medisinsk og helsefaglig forskningsetikk  REK Sør-Øst A  Postboks 1130, Blindern  Oslo  0318  NORWAY |
| Comité Institucional de ética en investigación PRISMA  PERU |  |
| Comité Institucional de Ética en investigación de la Universidad de San Martín de Porres –Clínica Cada Mujer  PERU |  |
| HOSPITAL NACIONAL ALMANZOR AGUINAGA ASENJO  Pza. Plaza de la Segurida Nro. S/n - CHICLAYO -  Lambayeque  PERU |  |
|  | Komisja Bioetyczna przy Centrum Medycznego Ksztalcenia Podyp  ul. Marymoncka 99/103  Warszawa01-813  Mazowieckie  POLAND |
|  | Nat bioethics committee of medical product - medical devices  19-21 Stefan cel mare street District 2  Bucharest  020125  ROMANIA |
|  | National bioethics committee of medical product and medical devices  19-21 Stefan cel mare street District 2  Bucharest  020125  ROMANIA |
|  | Nat bioethics committee of medical product - medical devices  19-21 Stefan cel mare street  District 2  Bucharest 020125  ROMANIA |
| LEC of the 1st Moscow State Medical University n.a.Sechenov  8, build 2, Trubetskaya str.,  Moscow  119992  RUSSIAN FEDERATION | Ethics Board at Ministry of Health of the Russian Federation  3, Rakhmanovskiy per.,  Moscow  127994  RUSSIAN FEDERATION |
| LEC of the Burdenko Main Military Hospital  3, Gospitalnaya sq.,  Moscow  105229  RUSSIAN FEDERATION | Ethics Board at Ministry of Health of the Russian Federation  3, Rakhmanovskiy per.,  Moscow  127994  RUSSIAN FEDERATION |
| Eticka komisia Bratislavskeho samospravneho kraja  Sabinovska 16  Bratislava  82005  SLOVAKIA | EK Univerzitnej nemocnice Bratislava - Stare Mesto  Mickiewiczova 13  Bratislava  81369  SLOVAKIA |
| Eticka komisia Nitrianskeho samospravneho kraja  Stefanikova trieda 69  Nitra  94901  SLOVAKIA | EK Univerzitnej nemocnice Bratislava - Stare Mesto  Mickiewiczova 13  Bratislava  81369  SLOVAKIA |
| Comité etico regional Comunidad de Madrid  Aduana,29  3ª planta  28013  Madrid  SPAIN | Comité etico regional Comunidad de Madrid  Aduana,29  3ª planta  28013  Madrid  SPAIN |
| Comité etico regional Comunidad de Madrid  Aduana,29  3ª planta  28013  Madrid  SPAIN | Comité etico regional Comunidad de Madrid  Aduana,29  3ª planta  28013  Madrid  SPAIN |
| Kantonale Ethikkommission Thurgau  Spitalcampus 1  Münsterlingen 8596  SWITZERLAND |  |
| Commission cantonale (VD) d'ethique de la recherche sur l'et  Commission cantonale (VD) d'ethique de la recherche sur l'etre human  Secretariat administratif  Avenue de Chailly 23  Lausanne 1012  SWITZERLAND |  |
| Ethics Committee  Dokuz Eylül University School of Medicine Dean's Office  Inciralti  35340 IZMIR  TURKEY | Ethics Committee  Dokuz Eylül University School of Medicine Dean's Office  Inciralti  35340 IZMIR  TURKEY |
| Ethics Committee  Dokuz Eylül University School of Medicine Dean's Office  Inciralti  35340 IZMIR  TURKEY | Ethics Committee  Dokuz Eylül University School of Medicine Dean's Office  Inciralti  35340 IZMIR  TURKEY |
| LEC of Khmelnytsky regional hospital  1 Pilotska str.  Khmelnytsky 29000  UKRAINE |  |
| LEC of Khmelnytsky regional hospital  1 Pilotska str.  Khmelnytsky 29000  UKRAINE |  |
|  | West of Scotland REC 1  Ground Floor  Tennant Institute  Western Infirmary  38 Church Street  Glasgow G11 6NT  Strathclyde  UNITED KINGDOM |
| Genesis Health Care IRB, 800 Forest Ave., Zanesville, OH 43701, USA |  |
|  | Copernicus IRB Group, PO BOX 110605, Research Triangle Park, NC 27709, USA |
|  | Copernicus IRB Group, PO BOX 110605, Research Triangle Park, NC 27709, USA |
| Integris Health Inc., 3400 NW Expressway, Bldg C, Ste 806, Oklahoma City, OK 73112, USA |  |
|  | Copernicus IRB Group, PO BOX 110605, Research Triangle Park, NC 27709, USA |
| University of California Davis Medical Center IRB, IRB Administration, CTSC Bldg., Rm. 1429, 2921 Stockton Blvd., Ste. 1400, Sacramento, CA 95817, USA |  |
|  | Copernicus IRB Group, PO BOX 110605, Research Triangle Park, NC 27709, USA |
|  | Copernicus IRB Group, PO BOX 110605, Research Triangle Park, NC 27709, USA |
|  | Copernicus IRB Group, PO BOX 110605, Research Triangle Park, NC 27709, USA |
|  | Copernicus IRB Group, PO BOX 110605, Research Triangle Park, NC 27709, USA |
|  | Copernicus IRB Group, PO BOX 110605, Research Triangle Park, NC 27709, USA |
| University Medical Center of Southern Nevada IRB, 1800 W Charleston Blvd., Las Vegas, NV 89102, USA |  |
|  | Copernicus IRB Group, PO BOX 110605, Research Triangle Park, NC 27709, USA |
|  | Copernicus IRB Group, PO BOX 110605, Research Triangle Park, NC 27709, USA |
|  | Copernicus IRB Group, PO BOX 110605, Research Triangle Park, NC 27709, USA |
| Catholic Medical Center IRB, 100 McGregor St., Manchester, NH 03102, USA |  |
|  | Copernicus IRB Group, PO BOX 110605, Research Triangle Park, NC 27709, USA |
| Chesapeake Research Review, LLC, 7063 Columbia Gateway Dr., Ste 110, Columbia,MD 21046, USA |  |
|  | Copernicus IRB Group, PO BOX 110605, Research Triangle Park, NC 27709, USA |
|  | Copernicus IRB Group, PO BOX 110605, Research Triangle Park, NC 27709, USA |
| Elmhurst Hospital Institutional Review Board, 155 E Brush Hill Rd., Elmhurst, IL 60126, USA |  |
| Ochsner IRB, 1514 Jefferson Hwy., New Orleans, LA 70121, USA |  |
| Advocate Health and Hosptials IRB, 3705 Highland Parkway, Rm 4867-N, Downers Grove, IL 60515, USA |  |
| Western Institutional Review Board, 3535 7th Ave., SW, PO Box 12029, Olympia, WA 98508, USA |  |
|  | Copernicus IRB Group, PO BOX 110605, Research Triangle Park, NC 27709, USA |
|  | Copernicus IRB Group, PO BOX 110605, Research Triangle Park, NC 27709, USA |
| Thomas Jefferson University, 1015 Chestnut St., Ste. 313, Philadelphia, PA 19107, USA |  |
|  | Copernicus IRB Group, PO BOX 110605, Research Triangle Park, NC 27709, USA |

# References

1. Schwartz GG, Bessac L, Berdan LG, et al. Effect of alirocumab, a monoclonal antibody to PCSK9, on long-term cardiovascular outcomes following acute coronary syndromes: rationale and design of the ODYSSEY outcomes trial. *Am Heart J* 2014; 168: 682-9

2. Schwartz GG, Steg PG, Szarek M, et al. Alirocumab and Cardiovascular Outcomes after Acute Coronary Syndrome. *N Engl J Med* 2018; 379: 2097-107

3. Gail MH, Simon R. Tests for Qualitative Interactions between Treatment Effects and Patient Subsets. *Biometrics* 1985; 41: 361–72
